# Whole gut virome analysis of 476 Japanese revealed a link between phage and autoimmune disease

**DOI:** 10.1136/annrheumdis-2021-221267

**Published:** 2021-12-08

**Authors:** Yoshihiko Tomofuji, Toshihiro Kishikawa, Yuichi Maeda, Kotaro Ogawa, Takuro Nii, Tatsusada Okuno, Eri Oguro-Igashira, Makoto Kinoshita, Kenichi Yamamoto, Kyuto Sonehara, Mayu Yagita, Akiko Hosokawa, Daisuke Motooka, Yuki Matsumoto, Hidetoshi Matsuoka, Maiko Yoshimura, Shiro Ohshima, Shota Nakamura, Hidenori Inohara, Hideki Mochizuki, Kiyoshi Takeda, Atsushi Kumanogoh, Yukinori Okada

**Affiliations:** 1 Department of Statistical Genetics, Osaka University Graduate School of Medicine, Suita, Osaka, Japan; 2 Department of Otorhinolaryngology-Head and Neck Surgery, Osaka University Graduate School of Medicine, Suita, Japan; 3 Department of Head and Neck Surgery, Aichi Cancer Center Hospital, Nagoya, Japan; 4 Department of Respiratory Medicine and Clinical Immunology, Osaka University Graduate School of Medicine, Suita, Japan; 5 Laboratory of Immune Regulation, Department of Microbiology and Immunology, Osaka University Graduate School of Medicine, Suita, Japan; 6 Integrated Frontier Research for Medical Science Division, Institute for Open and Transdisciplinary Research Initiatives, Osaka University, Suita, Japan; 7 Department of Neurology, Osaka University Graduate School of Medicine, Suita, Japan; 8 Department of Pediatrics, Osaka University Graduate School of Medicine, Suita, Osaka, Japan; 9 Laboratory of Statistical Immunology, Immunology Frontier Research Center (WPI-IFReC), Osaka University, Suita, Japan; 10 Department of Neurology, Suita Municipal Hospital, Suita, Japan; 11 Department of Infection Metagenomics, Research Institute for Microbial Diseases, Osaka University, Suita, Japan; 12 Rheumatology and Allergology, NHO Osaka Minami Medical Center, Kawachinaga, Japan; 13 WPI Immunology Frontier Research Center, Osaka University, Suita, Japan; 14 Department of Immunopathology, Immunology Frontier Research Center, Osaka University, Suita, Japan; 15 Laboratory for Systems Genetics, RIKEN Center for Integrative Medical Sciences, Yokohama, Japan

**Keywords:** rheumatoid arthritis, systemic lupus erythematosus, autoimmunity

## Abstract

**Objective:**

The relationship between autoimmune diseases and the gut microbiome has been intensively studied, and several autoimmunity-associated bacterial taxa have been identified. However, much less is known about the roles of the gut virome in autoimmune diseases.

**Methods:**

Here, we performed a whole gut virome analysis based on the shotgun sequencing of 476 Japanese which included patients with rheumatoid arthritis (RA), systemic lupus erythematosus (SLE), multiple sclerosis and healthy control subjects.

**Results:**

Our case–control comparison of the viral abundance revealed that crAss-like phages, which are one of the main components of a healthy gut virome, significantly decreased in the gut of the patients with autoimmune disease, specifically the patients with RA and SLE. In addition, *Podoviridae* significantly decreased in the gut of the patients with SLE. To understand how these viruses affected the bacteriome, we performed a quantitative virus–bacterium association analysis and clustered regularly interspaced short palindromic repeat-based virus–bacterium interaction analysis. We identified a symbiosis between *Podovirida*e and *Faecalibacterium*. In addition, multiple bacterial targets of crAss-like phages were identified (eg, *Ruminococcus* spp).

**Conclusion:**

Our data suggest that the gut virome can affect our body either directly or via bacteria. Our analyses have elucidated a previously missing part of the autoimmunity-associated gut microbiome and presented new candidates that contribute to the development of autoimmune diseases.

**Key messages**

**What is already known about this subject?**

- Alteration of the gut microbiome has been linked to the pathogenesis of autoimmune diseases such as rheumatoid arthritis (RA), systemic lupus erythematosus (SLE) and multiple sclerosis. However, much less is known about the roles of the gut virome in the development of these diseases.

**What does this study add?**

- Our case–control comparison of the viral abundance revealed that crAss-like phages, which are one of the main components of a healthy gut virome, significantly decreased in the gut of the patients with autoimmune disease, mainly in the patients with RA and SLE.
- Multiple bacterial targets of crAss-like phages were identified (eg, *Ruminococcus* spp) from the quantitative virus–bacterium association analysis and clustered regularly interspaced short palindromic repeat based virus–bacterium interaction analysis.
- *Podoviridae*, which has a symbiotic relationship to *Faecalibacterium*, significantly decreased in the gut of the patients with SLE.

**How might this impact on clinical practice or future developments?**

- Our analyses have elucidated a previously missing part of the autoimmunity-associated gut microbiome and presented new candidates that contribute to the development of autoimmune diseases.

## Introduction

Despite the recent advancements in medicine, autoimmune diseases are increasing in prevalence and cause significant chronic morbidity and disability.^1 2^ Thus, their prevention and treatment are considered important goals for modern medicine. Many autoimmune diseases are caused by complex genetic and environmental factors and their interactions. Genetic factors of autoimmune diseases are being revealed through various studies, such as the genome-wide association study.^3 4^ Meanwhile, environmental factors have also been identified; however, much remain to be elucidated.

The gut microbiome, which refers to the microbial communities inhabiting our gut, substantially influences our health via the immune and metabolic systems.^5^ Accompanied by advances in analytical methods, many studies have been conducted to reveal the complex relationships between the gut microbiome and human diseases such as metabolic disease,^6^ cancer,^7^ and intestinal disease.^8^ As for autoimmune diseases, the gut microbiome has been considered one of the most significant environmental contributors in their development. The reduced diversity of the gut microbiome has been reported in various autoimmune diseases, including rheumatoid arthritis (RA) and systemic lupus erythematosus (SLE).^9^ In addition, *Prevotella copri* contributes to the pathogenesis of RA by activating the immune response via Th17 cells.^10^

A large proportion of the gut microbiome is composed of viruses.^11^ The most predominant component of the gut virome is bacteriophage. Bacteriophages infect bacteria and regulate the gut bacteriome by either lysing their hosts or altering their physiological function.^12^ In the patients with *Clostridium difficile* infection, reconstruction of a healthy gut microbiome through faecal transplantation was associated with the viral composition of the donor’s faeces,^13–15^ supporting the significant role of bacteriophages in the maintenance of the gut microbiome. In addition to their mediating effects, recent studies have suggested that bacteriophages can directly affect our body via the immune system.^16 17^ Although it is evident that the gut virome has a significant impact on the host’s physiology, many previous metagenomic studies have ignored the viral component of the gut microbiome due to technical difficulties.

As viruses require a host for growth and only a limited fraction of the gut bacteriome can be cultured, most of the gut virome cannot be evaluated through conventional laboratory approaches. Although high-throughput sequencing technologies have enabled us to analyse unculturable microbes, its application in virome analysis is not as straightforward. As viruses do not have universal taxonomic markers such as bacterial 16S ribosomal RNA (rRNA), gut virome studies have used virus-like particle (VLP) sequencing or whole-metagenome shotgun sequencing that require complex data processing and computational resources. In addition, the quantification methods used in bacterial analysis are not transferable to virome analysis because the current reference viral genome databases cover only a limited fraction of the actual gut virome populations.^11^ Although previous studies have quantified the gut viral abundance by nucleotide-level alignment of individual reads or assembled contigs to viral sequence databases, they could only evaluate only a minority of the gut virome populations.^18 19^ Owing to the extensive efforts in the development of computational methods and algorithms optimised for viruses, a comprehensive view of the gut virome in the physiological condition started to be revealed.^20–23^ However, the utilisation of such viral-optimised analysis methods remains limited.

In addition to the quantification of the gut viral abundance, the identification of the viral host is necessary to reveal the effect of bacteriophages on the gut microbial community. Although the coabundance analysis was useful in conferring virus–host interaction, it was not sufficient to show the evidence of viral infections.^24^ The whole-metagenome shotgun sequencing technology has enabled us to identify infectious targets of the bacteriophages using clustered regularly interspaced short palindromic repeat (CRISPR) sequences in the bacterial contigs. CRISPR and CRISPR-associated (Cas) proteins comprise the CRISPR–Cas system, a bacterial adaptive immune system against predators such as bacteriophages.^25^ The CRISPR–Cas system intakes virus-derived sequences as CRISPR spacers to efficiently eject the virus during a subsequent infection. Thus, spacer sequences in the CRISPR loci are the immune memory of bacteria and footprints of a viral infection. Although the CRISPR analysis can provide direct evidence of a viral infection, its application in a large-scale virome analysis remains limited,^21 22^ possibly due to the high sequencing and computational cost of a whole-metagenome shotgun sequencing analysis.

A case–control discrepancy of the gut virome was reported for only a limited number of intestinal,^19 26^ and metabolic diseases,^27–29^ which included inflammatory bowel diseases (IBD).^18 30^ In IBD, the individual diversity of the gut virome and gut bacteriome increased and decreased compared with those of healthy subjects, respectively, suggesting the contribution of the gut virome to the disease pathogenesis. Most of the current virome case–control studies were conducted in Western countries,^18 30 31^ or China.^26–28^ Given the differences in the metagenomic landscape among different populations, the association between the gut virome and various diseases in diverse populations should be studied. In addition, limited numbers of studied diseases have hindered us from understanding the virus-associated disease aetiologies and the role of bacteriophages as healthy components of the gut microbiome. Although the importance of the gut microbial environment in systemic autoimmune diseases such as RA, SLE and multiple sclerosis (MS) has been well established,^9 32 33^ there are few studies that have identified case–control discrepancies of the gut virome in these diseases.^31^ Therefore, a whole-virome analysis to reveal the associations between bacteriophages and autoimmune diseases is warranted.

Here, we performed a whole gut virome analysis of 476 Japanese which included the patients with RA (*N* = 111), SLE (*N* = 47) and MS (*N* = 29) and healthy control (HC) subjects (*N* = 289). This is the largest case–control comparison of the viral abundance to date. We evaluated changes in the gut virome and whether it was specific to each autoimmune disease or shared across multiple autoimmune diseases. In addition, we performed a virus–bacterium association analysis based on the abundance data and a CRISPR-based virus–bacterium interaction analysis to reveal the virus–bacterium interaction mediated by disease-associated viruses identified in this study.

## Methods

### Patient participation

The study included 112 patients with RA, 48 patients with SLE, 29 patients with MS, and 296 HC subjects. Most of these subjects were derived either from previous studies,^32 33^ or a recently conducted SLE metagenome study.^34^ The patients with RA were enrolled at the Osaka University Hospital, National Hospital Organization Osaka Minami Medical Center, and Daini Osaka Police Hospital. The patients with SLE were enrolled at the Osaka University Hospital and National Hospital Organization Osaka Minami Medical Center. The patients with MS were enrolled at the Osaka University Hospital. The HC subjects were enrolled at the Osaka University Graduate School of Medicine, Osaka University Hospital, and National Hospital Organization Osaka Minami Medical Center. The patients with RA were diagnosed by physicians according to the American College of Rheumatology and the European League Against Rheumatism 2010 criteria for RA.^35^ The patients with SLE were diagnosed by physicians according to the Systemic Lupus International Collaborating Clinics classification criteria.^36^ The patients with MS were diagnosed by physicians according to the McDonald 2010 criteria.^37^ The Disease Activity Score 28 using C reactive protein,^38^ SLE Disease Activity Index^39^ and Expanded Disability Status Scale,^40^ were calculated to evaluate the activity of each disease. The HC subjects had no personal history of the immune-related diseases.

Participants with extreme diets (eg, strict vegetarians) were not included in the dataset. All subjects provided written informed consent before participation. Those who took antibiotics within a month was reported as the patients treated with antibiotics. The characteristics of the subjects are shown in online supplemental table 1.

10.1136/annrheumdis-2021-221267.supp1 Supplementary data

### Sample collection and DNA extraction

Faecal samples were collected in tubes containing RNAlater (Ambion). After the weights of the samples were measured, RNAlater was added to produce 10-fold dilutions of homogenates. Faecal samples were stored at −80°C within 24 hours after collection. Bacterial DNA was extracted according to a previously described method. Briefly, 0.3 g glass beads (diameter: 0.1 mm) (BioSpec) and 500 µL EDTA-Tris-saturated phenol were added to the suspension, and the mixture was vortexed vigorously using a FastPrep-24 (MP Biomedicals) at 5.0 power level for 30 s. After centrifugation at 20 000 g for 5 min at 4°C, 400 µL of supernatant was collected. Subsequently, phenol–chloroform extraction was performed, and 250 µL of supernatant was subjected to isopropanol precipitation. Finally, DNAs were suspended in 100 µL EDTA-Tris buffer and stored at −20°C.

### Whole-genome shotgun sequencing

A shotgun sequencing library was constructed using the KAPA Hyper Prep Kit (KAPA Biosystems), and 150 bp paired-end reads were generated on HiSeq 3000 and NovaSeq 6000 for sequencing batches 1–4 and batch 5, respectively. The sequencing data from the patients with RA in sequencing batch 3 were newly obtained for this study, while other sequencing data were derived from previous studies,^32 33^ or a recently conducted SLE metagenome study.^34^ The sequence reads were converted to FASTQ format using bcl2fastq (V.2.19).

### Quality control of sequencing reads

We followed a series of steps to maximise the quality of the datasets. The main steps in the quality control (QC) process were as follows: (1) trimming of low-quality bases, (2) identification and masking of human reads and (3) removal of duplicated reads. We marked duplicate reads using PRINSEQ-lite (V.0.20.4; -derep 1). We trimmed the raw reads to clip Illumina adapters and cut-off low-quality bases at both ends using the Trimmomatic (V.0.39; parameters: ILLUMINACLIP:TruSeq3-PE-2.fa:2:30:10:8:true LEADING:20 TRAILING:20 SLIDINGWINDOW:3:15 MINLEN:60). We discarded reads less than 60 bp in length after trimming. Next, we performed duplicate removal by retaining only the longest read among the duplicates with the same sequences. As a final QC step, we aligned the quality-filtered reads to the human reference genome (hg38) using bowtie2 (V.2.3.5) with default parameters and BMTagger (V.3.101). We kept only the reads of which both paired ends failed to align in either tool. The average QC-passed total sequencing base pairs were 25.5, 7.8, 6.7, 7.0 and 8.3 Gb for sequencing batches 1–5.

### Viral contig assembly and identification

The de novo assembly of the filtered paired-end reads into contigs was conducted using MEGAHIT (V.1.2.9; parameters: -min-contig-len 1500). Following assembly, contigs whose 5’ and 3’ terminals have ≥50 bp overlap were marked as circular contigs. Linear contigs of ≥5 kbp and circular contigs of ≥1.5 kbp were subjected to VirSorter (V.1.0.6),^41^ and VirFinder (V.1.1).^42^ VirSorter was performed using both RefSeqABVir (–db 1) and Viromes (–db 2) databases, and sequences sorted as viruses with the ‘most confident’ prediction (category 1) or ’ikely’ prediction (category 2) were extracted for further analysis. Candidate viral sequences which were sorted as viruses with ‘possible’ prediction (category 3) by VirSorter were extracted for further analysis if they had a VirFinder score of ≥0.7. The remaining contigs were extracted for further analysis if they had a VirFinder score of ≥0.9. To minimise the contamination of bacterial sequences, we assessed the level of bacterial and viral gene enrichments as previously described by Gregory *et al*.^22^ We used bacterial single-copy orthologs v4 (BUSCOv4; V.4.1.2),^43^ to search the 124 bacterial single-copy orthologs registered in BUSCO’s database and then used the BUSCO provided HMM score cutoffs to filter our results. Viral gene enrichment was assessed using hmmsearch (V.3.1b2) of viral contigs against the curated viral protein family (VPF) modules (https://portal.nersc.gov/dna/microbial/prokpubs/EarthVirome_DP/),^44^ and any matches with E-value of <0.05 were defined as hits. We set a threshold for contamination of bacterial genome at BUSCO hit/number of VPF hits of >0.05. These procedures resulted in 93 254 total viral populations with an average length of 21.1 kbp.

### Taxonomic annotation

We first classified viral contigs using complete viral RefSeq genomes (downloaded in June 2020 containing 12 696 genomes; https://www.ncbi.nlm.nih.gov/labs/virus/vssi/#/) as reference. Although the taxonomic information of viral genomes was based on NCBI taxonomy data, family-level classification of *crAssphage* (NC_024711.1) was modified to crAss-like phage according to recent publications.^24^ The viral contig sequences were searched against the reference genome using megablast (ncbi-blast-plus V.2.10.1) with E-value of <10^−10^, nucleotide identity of >95%, and coverage of contigs of ≥85%. Viral contigs were assigned to higher than or equal to species level taxonomy based on the megablast hit with the highest bit score. Contigs not assigned taxonomy in the previous step based on the nucleotide-level comparison proceeded to protein-level comparison for annotation of family-level taxonomy. First, we predicted open reading frames (ORFs) in viral contigs using MetaProdigal (V.2.6.3) with -p meta option. To detect crAss-like phages, we compared ORFs in viral contigs and crAss-like phage signature genes.^21 45^ The protein sequences of the polymerase (UGP_018) and terminase (UGP_092) in *crAssphage* (NC_024711.1) were queried against the ORFs in viral contigs using blastp with E-value of <10^−5^ and alignment length of ≥350. Viral contigs with blastp hits were assigned family-level taxonomic annotation as crAss-like phage. Then, the remaining unclassified contigs were proceed to taxonomic annotation by a voting system.^21 23^ The ORFs in the viral contigs were searched against the RefSeq protein database (downloaded in June 2020, containing 420 609 proteins) using DIAMOND blastp (V.0.9.32.133) with E-value of <10^−5^. Based on the DIAMOND blastp hits with the highest bit score, the ORFs were assigned taxonomic annotation. We then summarised all the taxonomic assignments of the ORFs for each contig, and assigned taxonomic information higher than or equal to family level based on the majority taxonomic assignment among the annotated ORFs. Viral contigs without a majority taxonomic assignment were regarded as unclassified viruses. Viral contigs with less than two annotated ORFs were also regarded as unclassified viruses.

### Abundance quantification of viruses

To calculate the raw abundances of the different viral populations in each sample, the quality-checked reads from each sample were mapped to the viral contigs recovered from the sample using bowtie2 with default parameter. On average, 2.8% of the reads were mapped to viral contigs. CoverM filter (V.0.4.0) was used to remove the reads mapped at <95% nucleotide identity to the contigs. Then, CoverM trimmed_mean (parameters: --trim-min 0.05 --trim-max 0.95) was used to calculate the average read depth across the viral populations. The read depth of each viral population was divided by the total sequenced length of each sample for normalisation. The normalised read depth of each viral population was summed up by each sample, and the normalised abundance of each clade was calculated at different taxonomic ranks. Then, we detected outlier samples using principal component analysis (PCA).

### Abundance quantification of bacteria

After viral quantification, we extracted nonviral reads to obtain bacterial abundance. We used the previously constructed bacterial reference dataset composed of curated 7881 genomes.^32^ The filtered paired-end reads were aligned to the reference genome dataset using bowtie2 with default parameters. For the multiple-mapped reads, only the best possible alignment was selected by the alignment scores. The number of reads that was mapped to each genome was divided by the length of the genome. The value of each genome was summed up by each sample, and the relative abundance of each clade was calculated at six levels (L2: phylum, L3: class, L4: order, L5: family, L6: genus, L7: species). Then, we detected outlier samples using PCA. We removed one RA sample, one SLE sample, and seven HC samples on the basis of the PCA of the viral or bacterial raw abundance. The samples that passed QC, including those derived from the 111 patients with RA, 47 patients with SLE, 29 patients with MS, and 289 HC subjects, were used in the subsequent analysis.

### Association test of the viral abundance for age and sex

We normalised the viral abundances using log transformation. For family-level analysis, we retained only those clades that were detected in all the sequencing batches and ≥10% of the samples. The detection ratios of the QC-passed viral clades are indicated in online supplemental table 2. An association test of the viral abundance for age and sex was performed using the following formula: abundance of the virus ~ age + sex+phenotype + sequencing batch +total sequenced length. The lm() function in the R (V.4.0.1) was used for linear regression, and the effect size of the age and sex was evaluated.

### Case–control comparison of the viral abundance

Case–control association tests were performed separately for each standardised clade abundance using the glm() function in the R (V.4.0.1), and the effect size of the viral abundance was evaluated. Sequencing batches 1, 2 and 3 were used in the analysis for RA (*N*_case_=111 and *N*_control_=111). Sequencing batches 2, 3 and 5 were used in the analysis for SLE (*N*_case_=201 and *N*_control_=47). Sequencing batch 4 was used in the analysis for MS (*N*_case_=29 and *N*_control_=74). We adopted sex, age, age^2^, sequencing batch, and total sequenced length as covariates. To evaluate the effect of potential confounding factors, we performed sub-analysis with datasets from which (1) males were removed, (2) non-new onset patients were removed, (3) those treated with antibiotics were removed, (4) those treated with proton pump inhibitors were removed, (5) those treated with steroids were removed or (6) those treated for autoimmune diseases were removed. Case–control comparisons per sequencing batch were performed to evaluate the consistency of the results between the sequencing batches. In addition, comparisons between (1) the treated patients and the untreated patients, (2) the new onset patients and the non-new onset patients, and (3) those with high vs low disease activity were performed. For the meta-analysis, we used the metafor (V.3.0–2) package for R. As the case–control analyses for RA and SLE had significant overlap in control samples, we used only subjects from sequencing batch five for SLE case–control comparison. As for the random-effect model, ‘REML’ option was used for fitting parameters with restricted maximum likelihood estimation.

### Virus–bacterium association analysis

We normalised the bacterial relative abundance profiles using log transformation. We only retained the clades that were detected in all the sequencing batches, in ≥20% of the samples, and with an average relative abundance of more than 0.001% of the total abundance. After selection, we assessed 802 clades (12 phyla, 24 classes, 35 orders, 74 families, 188 genera and 469 species).

Virus–bacterium association tests were performed separately for each virus–bacterium pair using the lm function in the R, and the effect size of the viral abundance was evaluated. We evaluated the association between eight viral families that passed clade QC described above and 802 bacterial clades. We adopted sex, age, age^2^, sequencing batch, total sequenced length and the top 10 principal components of the normalised bacterial abundance as covariates. The false discovery ratio was calculated using the Benjamini-Hochberg procedure. To evaluate the effect of potential confounding factors, we performed subanalysis with datasets from which (1) male subjects were removed, (2) non-new-onset patients were removed, (3) only HC subjects were retained, (4) those treated with antibiotics were removed, (5) those treated with proton pump inhibitors were removed, (6) those treated with steroids were removed and (7) those treated for autoimmune diseases were removed. In addition, disease-specific virus–bacterium associations were evaluated by adding and evaluating virus × RA, virus × SLE, or virus × MS terms in the regression formula. The sample sets were defined as in the case–control comparison for each disease.

### Virus–bacterium interaction analysis based on CRISPR spacers

We extracted contigs of ≥5 kbp that were not classified as viral ones. Then, we predicted the CRISPR sequences in these contigs with MinCED (V.0.4.2),^46^ using the ‘-minNR 2’ parameter as previously described.^22^ Contigs with CRISPR sequences were queried against the bacterial reference genome using megablast with E-value of <10^−10^, nucleotide identity of >95%, and coverage of contigs of ≥90%. Based on the megablast hit with the highest bit score, bacterial taxonomy was assigned to the contigs. Spacers within the predicted CRISPR sequences were queried against the viral contigs recovered from the same sample using blastn with E-value of <10^−5^, nucleotide identity of >95%, and coverage of spacers of ≥90%. Based on the blastn hit with the highest bit score, viral taxonomy was assigned to the spacers. Then, we summarised the virus–bacterium pair based on taxonomy annotated to the spacers and the contigs, collapsed within each sample, and summarised across all the samples.

### Patient and public involvement

This research was done without patient and public involvement. Patients and public were not invited to comment on the study design and were not consulted to develop patient relevant outcomes or interpret the results.

## Results

### Obtaining viral abundance data from 476 individuals

**Figure 1:**
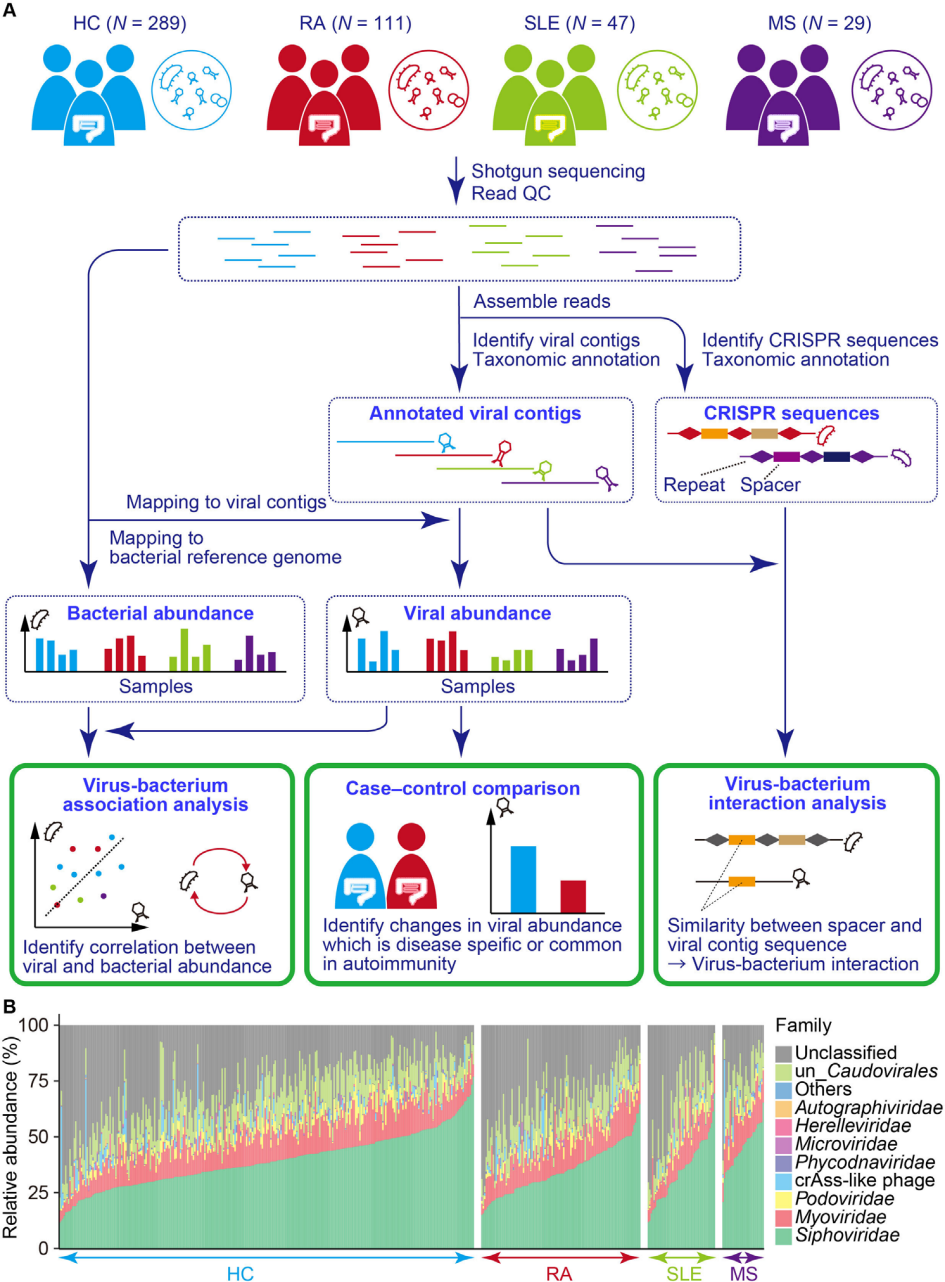
Overview of the whole gut virome analysis of the autoimmune diseases. (A) Schematic illustration of the study design. Shotgun sequencing data from HC subjects and patients with RA, SLE and MS were quality-checked and used for recovering viral contigs. The quality-checked reads were then mapped to the viral contigs recovered from the same sample to obtain the per sample viral abundance, followed by case–control comparison. Non-viral reads were mapped to a bacterial reference genome to obtain the per sample bacterial abundance. The viral and bacterial abundance data were integrated for virus–bacterium association analysis. CRISPR sequences in nonviral contigs were identified, and sequence similarity between spacer sequences and viral contigs recovered from the same sample was evaluated to identify the bacterial targets of the viruses. (B) Viral relative abundance at the family level. Relative abundance profiles were constructed using whole-genome shotgun sequencing (*N*_HC_=289, *N*_RA_=111, *N*_SLE_=47, *N*_MS_=29). CRISPR, clustered regularly interspaced short palindromic repeat; HC, healthy control; MS, multiple sclerosis; RA, rheumatoid arthritis; SLE, systemic lupus erythematosus; QC, quality control.

An overview of our study is shown in figure 1A. We obtained the gut viral abundance from the shotgun sequencing data of 476 faecal DNA samples (289 HC subjects, 111 patients with RA, 47 patients with SLE, and 29 patients with MS), which passed stringent QC for sequencing reads and samples (online supplemental figure 1). Detailed characteristics of the participants are shown in online supplemental table 1). Contigs were assembled from the extracted QC sequencing reads and viral contigs. To extract the viral contigs from the whole-genome shotgun sequencing data, we first extracted candidate viral contigs using VirSorter^41^ and VirFinder.^42^ Subsequently, we removed potential bacterial contaminations using BUSCO,^43^ and VPF,^44^ which resulted in 93,254 viral contigs (on average, 195.9 viral contigs per sample). We then performed taxonomic annotation and quantification of each viral contig. Among the viral contigs, 65,201 (69.9%) were classified at the family level. Consistent with previous reports,^21–23^ *Caudovirales* including *Siphoviridae*, *Myoviridae*, *Podoviridae,* and unclassified *Caudovirales* were highly abundant among the gut virome (average: 38.2%, 12.4%, 2.9% and 12.2%, respectively, figure 1B), suggesting that our pipeline successfully reconstructed the viral abundance from shotgun sequencing data. We performed QC of the family-level viral clades for subsequent case–control comparison. After clade QC, eight viral families were included in the association test. We evaluated the effects of age and sex on the viral abundance (online supplemental table 3). We found no significant association between age and sex, and the viral abundances (*P* > 0.05 / 8 = 0.0063).

### Case–control comparison of the viral abundance for RA, SLE and MS

**Figure 2:**
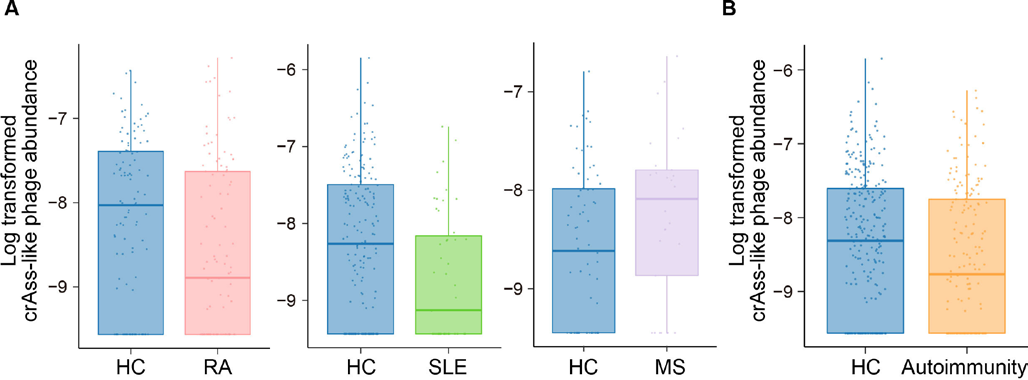
Case–control comparison of the crAss-like phage abundance. (A) Case–control comparison of the crAss-like phage abundance for RA (left), SLE (middle) and MS (right). Boxplots indicate the median values (centre lines) and IQRs (box edges), with the whiskers extending to the most extreme points within the range between (lower quantile−(1.5×IQR)) and (upper quantile+(1.5×IQR)). (B) Case–control comparison of the crAss-like phage abundance for autoimmunity. Boxplots indicate the median values (centre lines) and IQRs (box edges), with the whiskers extending to the most extreme points within the range between (lower quantile−(1.5×IQR)) and (upper quantile+(1.5×IQR)). HC, healthy control; MS, multiple sclerosis; RA, rheumatoid arthritis; SLE, systemic lupus erythematosus.

We performed case–control comparison for the eight QC-passed viral families in each autoimmune disease (table 1) using logistic regression with adjustments for age, age^2^, sex, sequencing batch and total sequencing length. We found that crAss-like phages significantly decreased in the metagenome of the patients with RA (*P* < 0.05 / 8 = 0.0063; figure 2A). As the medications of the patients with RA and male–female imbalance due to sex-biased prevalence could be confounding factors, we performed sub-analysis (online supplemental table 4). Even after removing the male subjects, non-new onset patients, or those who took medications such as proton pump inhibitors, steroids, and any therapeutics for RA, crAss-like phages still decreased in the gut metagenome of the patients with RA (effect size = −0.568, –0.385, −0.441, –0.475 and −0.520, respectively). A comparison within the case revealed that the abundance of crAss-like phages was not significantly affected by the treatment, timing of the onset, disease activity of RA, and an inflammation marker (effect size = −0.008, 0.313, 0.018, and 0.291; *P* = 0.97, 0.25, 0.93 and 0.20, respectively; online supplemental table 4). In the gut metagenome of the patients with SLE, *Podoviridae* significantly decreased (*P* < 0.05 / 8=0.0063; table 1), and crAss-like phages nominally decreased (*P* < 0.05; figure 2A, table 1). Similar to the patients with RA, we performed subanalysis. Although the p values increased in the subanalysis, which was possibly due to the decreased sample size, the effect size was consistent (online supplemental table 5). Abundances of crAss-like phages and *Podoviridae* in the gut metagenome of the patients with SLE were not significantly affected by the treatment, timing of the onset, and disease activity of SLE (effect size = −0.806, –0.593, and −1.147; *P* = 0.15, 0.31 and 0.070 for crAss-like phages; effect size = −0.289,–0.165 and 0.313; *P* = 0.63, 0.77 and 0.67 for *Podoviridae*, respectively; online supplemental table 5). None of the viral families, including crAss-like phages that decreased in the metagenome of both RA and SLE, were associated with MS (figure 2A, table 1).

**Table 1:** Result of the case–control comparison of the viral abundance for RA, SLE and MS

Viruses	RA (*N*_case_=111 and *N*_control_=111)			SLE (*N*_case_=47 and *N*_control_=201)			MS (*N*_case_=29 and *N*_control_=74)		
	Effect size	SE	*P*	Effect size	SE	*P*	Effect size	SE	*P*
*Autographiviridae*	−0.051	0.293	0.86	0.257	0.338	0.45	−0.380	1.052	0.72
crAss-like phage	−0.476	0.173	0.0060**	−0.514	0.206	0.012*	0.425	0.488	0.38
*Herelleviridae*	−0.579	0.320	0.070	−0.109	0.311	0.73	1.730	1.278	0.18
*Microviridae*	0.155	0.231	0.50	0.265	0.279	0.34	0.729	0.781	0.35
*Myoviridae*	0.657	0.672	0.33	−0.753	0.858	0.38	−1.908	2.108	0.37
*Phycodnaviridae*	0.049	0.256	0.85	−0.513	0.273	0.060	−0.004	0.567	0.99
*Podoviridae*	−0.147	0.325	0.65	−0.947	0.330	0.0041**	0.893	1.041	0.39
*Siphoviridae*	−0.986	1.437	0.49	−3.710	1.487	0.013*	−0.667	3.417	0.85

*P<0.05, **P<0.01.

MS, multiple sclerosis; RA, rheumatoid arthritis; SLE, systemic lupus erythematosus.

### crAss-like phages decreased in the gut metagenome of combined autoimmune diseases (RA, SLE and MS)

Autoimmune diseases are known to have a shared aetiology, and a combined analysis of multiple autoimmune diseases is useful to reveal the hidden aetiology with increased statistical power.^47^ A shared gut microbial component of the autoimmune diseases had been inferred from previous studies,^9^ suggesting that there could also be a virus-related and shared aetiology among autoimmune diseases. Thus, we performed a combined case–control comparison of the viral abundance in RA, SLE and MS.

Through logistic regression of all the HC subjects and the combined autoimmune disease patients (RA, SLE and MS), we found that the abundance of crAss-like phages was significantly decreased in the combined autoimmune diseases (figure 2B, table 2). In addition, *Siphoviridae* nominally decreased in the combined autoimmune disease patients. We performed a sub-analysis for certifying the significant association between crAss-like phages and combined autoimmune diseases. Even after removing male subjects, non-new-onset patients, or those who took medications such as antibiotics, proton pump inhibitors, steroids, and any therapeutics for combined autoimmune diseases, crAss-like phages decreased with the consistent effect sizes in the gut metagenome of the combined autoimmune diseases patients (effect size = −0.464, –0.534, −0.387, –0.417, −0.406 and −0.549, respectively; online supplemental table 6). When sequencing batch 4 was removed, which was composed of the HC subjects and the patients with MS, the association became more significant compared with that of the analysis with all the sequencing batches (effect size = −0.499, *P* = 2.6 × 10^−4^), suggesting that the significant decrease in crAss-like phages was driven by RA and SLE. A comparison between the treated patients and the untreated patients, or the new-onset patients and the non-new onset patients showed no significant difference (effect size = −0.087 and −0.007; *P* = 0.63 and 0.97, respectively; online supplemental table 6). We also performed a stratified analysis for each sequencing batch (sequencing batch 1–5). Although the p values became less significant due to the decreased sample size, the directions of the effect were consistent in all the batches, except for the batch 4 (online supplemental table 6), suggesting that the detected association was not solely dependent on a specific dataset. To take into account of the heterogeneity among the patients with RA, SLE and MS, we performed a random-effect meta-analysis (online supplemental table 7). Again, we detected a significant association between the combined autoimmune diseases in a random-effect model (effect size = −0.392, *P* = 0.0047) with non-significant heterogeneity among the diseases (*Q* = 3.03 and *P* for *Q* = 0.22). The Association between crAss-like phages and combined autoimmune diseases was detected even when the viral abundance was transformed to the absence/presence state (effect size = −0.734, *P* = 0.0046; online supplemental table 8), which was independent of the quantification or normalisation method.

**Table 2:** Results of the case–control comparison of the viral abundance for autoimmunity

Viruses	Effect size	SE	*P*
*Autographiviridae*	−0.048	0.216	0.82
crAss-like phage	−0.429	0.126	6.5×10^−4^**
*Herelleviridae*	−0.253	0.220	0.25
*Microviridae*	0.193	0.176	0.27
*Myoviridae*	0.057	0.555	0.92
*Phycodnaviridae*	−0.101	0.163	0.53
*Podoviridae*	−0.304	0.219	0.17
*Siphoviridae*	−1.956	0.959	0.041*

*P<0.05, **P<0.01.

In our study, some crAss-like phages, which had nucleotide-level similarity to NC_024711.1, were identified. We performed a case–control comparison of NC_024711.1 and other crAss-like phages in combined autoimmune diseases, which revealed that both NC_024711.1 and other crAss-like phages were significantly associated with combined autoimmune diseases (*P* = 0.0036 and *P =* 0.019, respectively; online supplemental table 9). crAss-like phages are bacteriophages that were first identified in 2014 by the cross-assembly of metagenomic sequencing reads.^24^ crAss-like phages occupy a significant part of the healthy gut virome, however, associations between crAss-like phages and human diseases have never been identified before.^48^

### Virus–bacterium coabundance between *Podoviridae* and *Faecalibacterium*

Bacteriophages impact our health via virus–bacterium interactions by modifying the abundance or function of their bacterial hosts. Thus, a comprehensive study of virus–bacterium interactions is useful for revealing the virus-related pathologies of autoimmune diseases. We performed a virus–bacterium association analysis based on the abundance of viruses and bacteria of all samples. We first quantified bacterial abundance using nonviral reads (figure 1A, online supplemental figure 2). For the virus–bacterium association analysis, we performed bacterial clade QC. Afterward, 802 bacterial clades were included in the association test.

**Figure 3:**
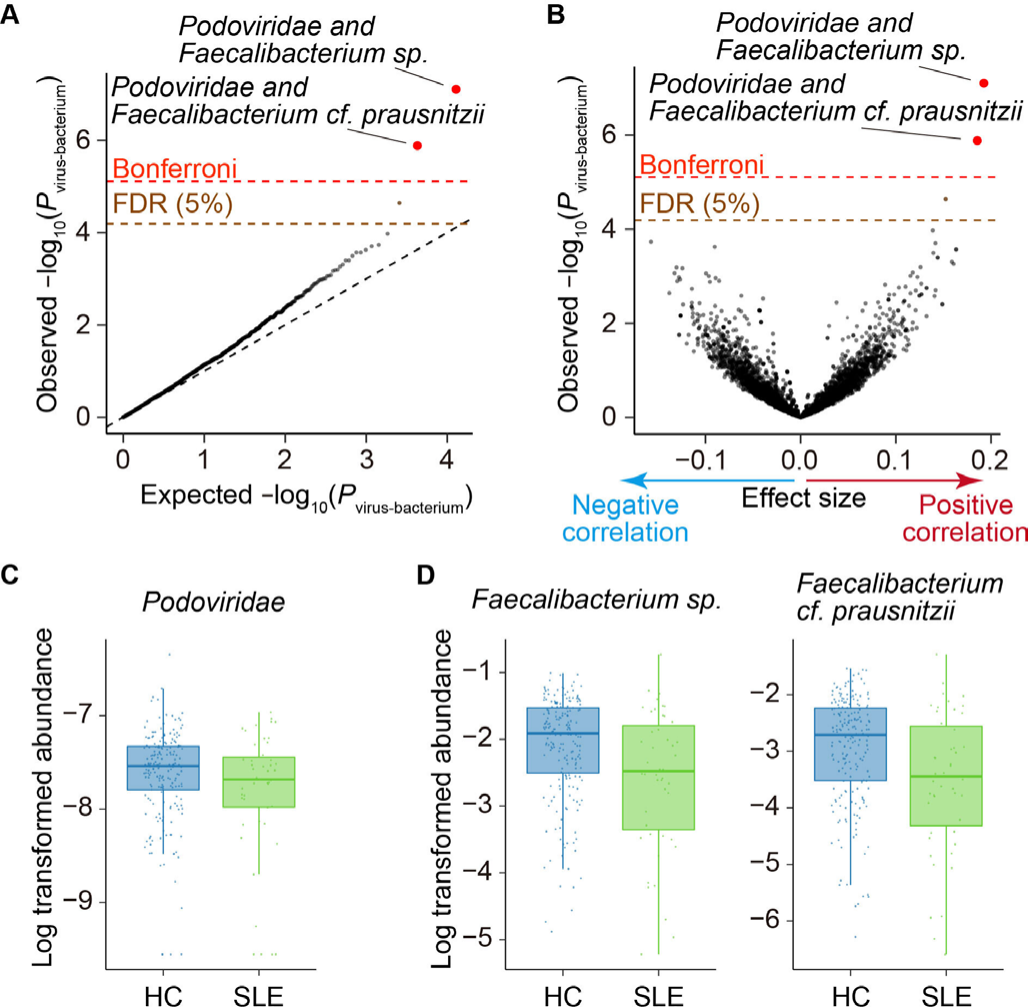
Virus–bacterium coabundances in the gut microbiome. (A) A quantile–quantile plot of the p values from the virus–bacterium association analysis (*P*_virus-bacterium_). The x-axis indicates −log_10_(*P*_virus-bacterium_) expected from uniform distribution. The y-axis indicates the observed −log_10_(*P*_virus-bacterium_). The diagonal dashed line represents *y=x*, which corresponds to the null hypothesis. The horizontal red dashed line indicates the Bonferroni-corrected threshold (*α*=0.05), and the brown dashed line indicates the FDR threshold (FDR=0.05) calculated using the Benjamini–Hochberg method. The virus–bacterium pairs with p value of less than the Bonferroni thresholds are plotted as red dots. The virus–bacterium pairs with FDR of <0.05 are plotted as brown dots, and other virus–bacterium pairs are plotted as black dots. (B) A volcano plot. The x-axis indicates the effect sizes in linear regression. The y-axis, horizontal dashed lines and dot colours are the same as in (A). (C) Case–control comparison of the *Podoviridae* abundance for SLE. Boxplots indicate the median values (centre lines) and IQRs (box edges), with the whiskers extending to the most extreme points within the range between (lower quantile−(1.5×IQR)) and (upper quantile+(1.5×IQR)). (D Case–control comparison of the *Faecalibacterium* spp and *Faecalibacterium cf. prausnitzii* abundance for SLE. Boxplots indicate the median values (centre lines) and IQRs (box edges), with the whiskers extending to the most extreme points within the range between (lower quantile−(1.5×IQR)) and (upper quantile+(1.5×IQR)). FDR, false discovery rate; HC, healthy control; SLE, systemic lupus erythematosus.

We then evaluated the association between 8 viral families and 802 bacterial clades. The abundances of two bacterial clades, *Faecalibacterium* spp and *Faecalibacterium* cf. *prausnitzii*, were positively correlated with the abundance of *Podoviridae* (effect size = 0.192, *P*_virus-bacterium_ = 7.9 × 10^−8^ for *Faecalibacterium* spp; effect size = 0.186, *P*_virus-bacterium_ = 1.3 × 10^−6^ for *Faecalibacterium*. cf. *prausnitzii*; figure 3A, B, online supplemental table 10, online supplemental file, satisfying a Bonferroni threshold (*α* < 0.05; *P*_virus-bacterium_ < 7.8 × 10^−6^). Even after removing male subjects, non-new onset patients, patients with combined autoimmune disease, or those who took medications such as antibiotics, proton pump inhibitors, steroids and any therapeutics for autoimmune diseases, the effect sizes of the association between *Faecalibacterium* and *Podoviridae* were consistent (online supplemental table 11). We performed a virus–bacterium association analysis with interaction term between diseases and viruses (online supplemental figure 3) but could not find significant disease-specific virus–bacterium associations.

10.1136/annrheumdis-2021-221267.supp2 Supplementary data

In our case–control comparison of the viral abundance, *Podoviridae* significantly decreased in the gut metagenome of the patients with SLE (figure 3C, table 1). *Faecalibacterium* spp and *Faecalibacterium cf. prausnitzii*, which were positively associated with *Podoviridae*, also tended to decrease in the gut metagenome of the patients with SLE (figure 3D). In the bacterial microbiome-wide association study of the patients with SLE conducted with subsets of dataset,^34^ (sequencing batch 1, 2 and 5), nonsignificant decreases of *Faecalibacterium* spp and *Faecalibacterium cf. prausnitzii* were observed (effect size = −0.04, *P* = 0.79; effect size = −0.11, *P* = 0.50, respectively). *Faecalibacterium* is a bacterial genus that produces short-chain fatty acids, which have anti-inflammatory activity.^49^ Changes in the abundance of *Faecalibacterium* were frequently reported in immune-related diseases or conditions.^49–51^ Thus, our results suggest that the symbiotic network of *Podoviridae* and *Faecalibacterium* is important to keep the homeostasis of the immune system.

### Viral host detection based on the CRISPR spacer sequences

To further elucidate the virus–bacterium interaction, we analysed the CRISPR loci of bacterial contigs from all samples to identify infected viruses. We detected 28,347 CRISPR spacer sequences in total (601.6 CRISPR spacer sequences per sample) by MinCED,^46^ and identified 834 unique taxonomically annotated virus–host interactions from 476 gut metagenomes (online supplemental figure 4).

Consistent to a previous report of healthy Japanese subjects,^21^ virus–host interactions involving *Caudovirales* such as *Siphoviridae*, *Myoviridae* and *Podoviridae* were frequently observed. In addition, the preferred hosts for each viral family (eg, *Podoviridae* preferentially infected to *Actinobacteria*, including *Bifidobacterium*, while *Myoviridae* did not; *Microviridae* preferentially infected *Bacteroidetes*, including *Bacteroides* compared with other viral families.) were also consistent with the previous report.

To reveal the autoimmunity-associated virus–bacterium interaction, we searched the hosts of autoimmunity-associated viruses, crAss-like phages and *Podoviridae* (table 3). As for *Podoviridae,* interactions with *Faecalibacteriums* spp via the CRISPR–Cas system were identified from a sample. Manual inspection of a contig supporting this interaction revealed that this contig aligned to a common region across *Faecalibacterium* (*Faecalibacterium* spp, *Faecalibacterium cf. prausnitzii and Faecalibacterium prausnitzii*). This result supported the symbiotic relationship between *Podoviridae* and *Faecalibacterium*, which was suggested from the quantitative virus–bacterium association analysis.

**Table 3:** Infectious targets of crAss-like phages and *Podoviridae* based on CRISPR spacers

Viruses	Bacteria
crAss-like phage	*Anaerobutyricum hallii,* *Bacteroides vulgatus* , *Blautia* spp, *Dorea longicatena, Eubacterium limosum,* *Ruminococcus* spp
*Podoviridae*	*Akkermansia muciniphila, Akkermansia* spp, *Bacteroides plebeius, Bacteroides* spp, *Bacteroides uniformis, Bifidobacterium adolescentis, Bifidobacterium bifidum, Bifidobacterium dentium, Bifidobacterium longum, Bifidobacterium pseudocatenulatum, Bifidobacterium* spp, *Blautia obeum, Blautia* spp, *Burkholderiales bacterium, Clostridium clostridioforme, Clostridium spp, Coprobacillus* spp, *Dialister invisus, Eubacterium rectale, Eubacterium ventriosum,* *Faecalibacterium* spp, *Firmicutes bacterium, Gemmiger formicilis, Holdemanella biformis, Parabacteroides distasonis, Parabacteroides merdae, Porphyromonas* spp, *Ruminococcus bromii, Ruminococcus* spp, *Streptococcus salivarius, Sutterella wadsworthensis*

The underlined bacteria have the following characteristics (see DISCUSSION): (1) *Bacteroides vulgatus* belongs to phylum *Bacteroidetes* which is a predicted host of crAss-like phages.^24^ (2) *Ruminococcus* spp belongs to genus *Ruminococcus* which is reported to be associated with RA,^55^ and SLE.^54^ (3) *Faecalibacterium* spp is also associated with *Podoviridae* also in the quantitative virus–bacterium association analysis and reported to be associated with autoimmune diseases.^50 51^

CRISPR, clustered regularly interspaced short palindromic repeat.

It was reported that crAss-like phages, especially those closely related to the first identified *crAssphage* (NC_024711.1), infected to *Bacteroidetes* from multiple lines of evidence.^24 52^ In our results, a predicted host of NC_024711.1 was *Bacteroides vulgatus*. Fujimoto *et al* reported that crAss-like phages, which had nucleotide-level similarity to NC_024711.1 dominantly infected to *Bacteroidetes*, while other crAss-like phages could also infect to *Firmicutes*.^21^ In line with those results, crAss-like phages other than NC_024711.1 infected to various *Firmicutes* in our analysis. Among the virus–host interaction involving crAss-like phages, *Ruminococcus* spp infection was detected in three independent samples. Therefore, we detected several hosts of the bacteriophages which decreased in autoimmune diseases.

## Discussion

In this study, we revealed the disease-associated changes in the gut virome of Japanese patients with autoimmune disease. We discovered that crAss-like phages in the gut of the patients with RA and SLE decreased. In addition, *Podoviridae* also decreased in the gut of the patients with SLE. Our quantitative virus–bacterium association analyses revealed a positive correlation between *Podoviridae* and *Faecalibacterium* abundance, and a direct evidence of phage infection was provided by CRISPR-based virus–bacterium interaction analyses. *Bacteroidetes* and *Firmicutes*, including *Ruminococcus* spp, were detected as hosts of crAss-like phages, which were replicated in multiple samples.

We obtained the gut virome abundance data from the whole-metagenome shotgun sequencing reads. The resulting viral composition, characterised by highly abundant *Caudovirales* that included *Siphoviridae*, *Myoviridae* and *Podoviridae*, was mostly consistent with previous reports,^21–23^ indicating that our pipeline successfully worked. This result indicated the advantage of whole-metagenome shotgun sequencing, which could be applied for both the bacteriome and virome analyses. Although the abundance of *Microviridae* was lower than that in previous studies performed with VLP-sequencing,^21 23^ it could have been due to the difference in the sequencing and library preparation methods. In the gut, bacteriophages can present as both free viral particles and infecting phages, which include prophages that are integrated in the bacterial genome. Although VLP-sequencing mainly captures free viral particles, whole-metagenome shotgun sequencing mainly captures actively infecting viruses and integrated prophages.^11^ In addition, the quantification of single-stranded DNA (ssDNA) viruses including *Microviridae* was strongly dependent on the library preparation method, specifically the multiple displacement amplification technique that is frequently used in VLP-sequencing and is known to show an upward bias of the ssDNA viral abundance.^11^ Gregory *et al* compared the VLP-sequencing data with the shotgun sequencing data and revealed that there was a large difference in the reconstructed viral populations, and the composition of ssDNA viruses recovered from shotgun sequencing data was consistently lower than that of the VLP-sequencing data.^22^ In this study, we used the whole-metagenome shotgun sequencing method to focus on actively infecting phages and prophages which occupied the majority of the gut virome and actively participated in virus–bacterium interaction. However, future integrative analysis with VLP-sequencing might result in a more comprehensive understanding of the gut virome.

*crAssphage* was first identified in 2014 through the cross-assembly of shotgun sequencing data from multiple faecal samples.^24^ Although *crAssphage* frequently existed in the healthy gut virome, a large fraction of its proteins showed no similarity to the already existing viral proteins. Thus, *crAssphage* was considered as comprising a new taxonomic group (crAss-like phage). Since crAss-like phages were discovered, its biological features including taxonomy and infective hosts,^52 53^ were extensively studied. Although the identification of the association between *crAssphage* and disease states have been warranted, there had been no reports on the relationships between *crAssphage* and diseases.^48^ In this study, the case–control comparison of the viral abundance revealed that the amount of crAss-like phages decreased in the gut metagenome of the patients with autoimmune diseases, specifically RA and SLE. In our dataset, the sample size of the patients with MS was relatively smaller than those with RA and SLE due to the rarity of MS in Japan. Therefore, future larger-scale analyses are warranted to analyse the relationship between viruses and MS with more statistical power.

In our CRISPR-based virus–bacterium interaction analysis, *Bacteroides vulgatus* and various *Firmicutes* were detected as hosts of crAss-like phages. *Bacteroidetes* was originally predicted as a host of crAss-like phages by read co-occurrence analysis and CRISPR-based analysis.^24^ Thus, the detection of *Bacteroides vulgatus* as a host of *crAssphage* (NC_024711.1) was reasonable. Although not detected in our CRISPR-based analysis, *Bacteroides intestinalis* was also a host of crAss-like phages, which was validated in vitro.^53^ In our quantitative virus–bacterium interaction analysis, the abundance of crAss-like phages and *Bacteroides intestinalis* was nominally correlated (effect size = 0.083 and *P*_virus-bacterium_ = 0.0078), suggesting that the virus–host relationship between the two existed in our dataset. In previous bacterial case–control comparisons,^32 34^ there was a nominally significant negative association between *Bacteroides intestinalis* and SLE (effect size = −0.412, *P* = 0.033), although the association was not significant for RA (effect size = 0.056, *P* = 0.76).

Among the virus–host interactions involving crAss-like phages, infection of *Ruminococcus* spp was detected in three independent samples. The 16S rRNA sequencing study of the patients with SLE,^54^ and autoimmune arthritis model mice,^55^ suggested that the genus *Ruminococcus* was related to the SLE and RA pathology.

Recently, Yutin *et al* performed a CRISPR-based virus–host association analysis for crAss-like phages,^56^ and *Prevotella copri*, which associated with the pathogenesis of RA,^48^ was also detected as a host of crAss-like phages. Collectively, these results have suggested that crAss-like phages are associated with the autoimmune diseases, possibly via the interaction with the host’s immune system or the modulation of the biological properties of various bacteria. As the abundance of crAss-like phages is highly variable across different areas,^57^ studies in other areas are warranted.

*Podoviridae* significantly decreased in the gut metagenome of the patients with SLE. Both the quantitative and CRISPR-based virus–bacterium interaction analyses suggested the symbiosis of *Podoviridae and Faecalibacterium. Faecalibacterium* is known beneficial bacteria as they produce short-chain fatty acids.^58^ Among the *Faecalibacterium, Faecalibacterium prausnitzii* was the most intensively studied and is associated with many conditions, including autoimmune diseases.^50 51^ While there is the possibility that the decreased *Podoviridae* affected *Faecalibacterium* and vice versa, the effect of *Podoviridae* on the biological property of *Faecalibacterium* should be experimentally tested in the future.

In summary, our whole gut virome case–control study revealed a previously unknown part of the link between the gut microbiome and autoimmune diseases. A comprehensive understating of the gut microbiome, including its virome, deepens the insights of the pathogenesis of autoimmune diseases.

### URLs

bcl2fastq, https://support.illumina.com/sequencing/sequencing_software/bcl2fastq-conversion-software/downloads.html

Trimmomatic, http://www.usadellab.org/cms/?page=trimmomatic bowtie2, http://bowtie-bio.sourceforge.net/bowtie2/index.shtml

BMTagger, ftp://ftp.ncbi.nlm.nih.gov/pub/agarwala/bmtagger/

PRINSEQ, http://prinseq.sourceforge.net/

MEGAHIT, https://github.com/voutcn/megahit

VirSorter, https://github.com/simroux/VirSorter

VirFinder, https://github.com/jessieren/VirFinder

BUSCO, https://busco.ezlab.org hmmer, http://hmmer.org/download.html ncbi-blast-plus, https://blast.ncbi.nlm.nih.gov/Blast.cgi?PAGE_TYPE=BlastDocs&DOC_TYPE=Download

DIAMOND, https://github.com/bbuchfink/diamond coverM, https://github.com/wwood/CoverM

Prodigal, https://github.com/hyattpd/Prodigal

MinCED, https://github.com/ctSkennerton/minced

Samtools, http://www.htslib.org/download/ bedtools, https://github.com/arq5x/bedtools2

R, https://www.r-project.org

Python, https://www.python.org/downloads/release/python-376/

Seqkit, https://bioinf.shenwei.me/seqkit/download/

RefSeq (Virus), https://www.ncbi.nlm.nih.gov/labs/virus/vssi/#/

VPF, https://portal.nersc.gov/dna/microbial/prokpubs/EarthVirome_DP/

## Data Availability

Data are available in a public, open access repository. The whole-genome shotgun sequencing data are deposited in National Bioscience Database Center (NBDC) Human Database (http://humandbs.biosciencedbc.jp/) with the accession number of hum0197.

## References

[1] Bach J-F . The effect of infections on susceptibility to autoimmune and allergic diseases. N Engl J Med 2002;347:911–20. 10.1056/NEJMra020100 12239261

[2] Dinse GE , Parks CG , Weinberg CR , et al . Increasing prevalence of antinuclear antibodies in the United States. Arthritis Rheumatol 2020;72:1026–35. 10.1002/art.41214 32266792 PMC7255943

[3] Okada Y , Wu D , Trynka G , et al . Genetics of rheumatoid arthritis contributes to biology and drug discovery. Nature 2014;506:376–81. 10.1038/nature12873 24390342 PMC3944098

[4] Gutierrez-Arcelus M , Rich SS , Raychaudhuri S . Autoimmune diseases - connecting risk alleles with molecular traits of the immune system. Nat Rev Genet 2016;17:160–74. 10.1038/nrg.2015.33 26907721 PMC4896831

[5] Holmes E , Li JV , Marchesi JR , et al . Gut microbiota composition and activity in relation to host metabolic phenotype and disease risk. Cell Metab 2012;16:559–64. 10.1016/j.cmet.2012.10.007 23140640

[6] Qin J , Li Y , Cai Z , et al . A metagenome-wide association study of gut microbiota in type 2 diabetes. Nature 2012;490:55–60. 10.1038/nature11450 23023125

[7] Yachida S , Mizutani S , Shiroma H , et al . Metagenomic and metabolomic analyses reveal distinct stage-specific phenotypes of the gut microbiota in colorectal cancer. Nat Med 2019;25:968–76. 10.1038/s41591-019-0458-7 31171880

[8] Lloyd-Price J , Arze C , Ananthakrishnan AN , et al . Multi-Omics of the gut microbial ecosystem in inflammatory bowel diseases. Nature 2019;569:655–62. 10.1038/s41586-019-1237-9 31142855 PMC6650278

[9] Zhang X , Chen B-di , Zhao L-D , et al . The gut microbiota: emerging evidence in autoimmune diseases. Trends Mol Med 2020;26:862–73. 10.1016/j.molmed.2020.04.001 32402849

[10] Maeda Y , Kurakawa T , Umemoto E , et al . Dysbiosis contributes to arthritis development via activation of autoreactive T cells in the intestine. Arthritis Rheumatol 2016;68:2646–61. 10.1002/art.39783 27333153

[11] Shkoporov AN , Hill C . Bacteriophages of the Human Gut: The "Known Unknown" of the Microbiome. Cell Host Microbe 2019;25:195–209. 10.1016/j.chom.2019.01.017 30763534

[12] Silveira CB , Rohwer FL . Piggyback-the-Winner in host-associated microbial communities. NPJ Biofilms Microbiomes 2016;2:16010. 10.1038/npjbiofilms.2016.10 28721247 PMC5515262

[13] Ott SJ , Waetzig GH , Rehman A , et al . Efficacy of sterile fecal filtrate transfer for treating patients with Clostridium difficile infection. Gastroenterology 2017;152:799–811. 10.1053/j.gastro.2016.11.010 27866880

[14] Zuo T , Wong SH , Lam K . Bacteriophage transfer during faecal microbiota transplantation in Clostridium difficile infection is associated with treatment outcome. Gut 2018;67:634–43. 10.1136/gutjnl-2017-313952 28539351 PMC5868238

[15] Draper LA , Ryan FJ , Smith MK , et al . Long-Term colonisation with donor bacteriophages following successful faecal microbial transplantation. Microbiome 2018;6:220. 10.1186/s40168-018-0598-x 30526683 PMC6288847

[16] Keen EC , Dantas G . Close encounters of three kinds: bacteriophages, commensal bacteria, and host immunity. Trends Microbiol 2018;26:943–54. 10.1016/j.tim.2018.05.009 29909042 PMC6436384

[17] Guerin E , Hill C . Shining light on human gut bacteriophages. Front Cell Infect Microbiol 2020;10:481. 10.3389/fcimb.2020.00481 33014897 PMC7511551

[18] Norman JM , Handley SA , Baldridge MT , et al . Disease-Specific alterations in the enteric virome in inflammatory bowel disease. Cell 2015;160:447–60. 10.1016/j.cell.2015.01.002 25619688 PMC4312520

[19] Ansari MH , Ebrahimi M , Fattahi MR , et al . Viral metagenomic analysis of fecal samples reveals an enteric virome signature in irritable bowel syndrome. BMC Microbiol 2020;20:123. 10.1186/s12866-020-01817-4 32429898 PMC7236503

[20] Shkoporov AN , Clooney AG , Sutton TDS , et al . The human gut Virome is highly diverse, stable, and individual specific. Cell Host Microbe 2019;26:527–41. 10.1016/j.chom.2019.09.009 31600503

[21] Fujimoto K , Kimura Y , Shimohigoshi M , et al . Metagenome data on intestinal Phage-Bacteria associations AIDS the development of phage therapy against Pathobionts. Cell Host Microbe 2020;28:380–9. 10.1016/j.chom.2020.06.005 32652061

[22] Gregory AC , Zablocki O , Zayed AA , et al . The gut Virome database reveals age-dependent patterns of Virome diversity in the human gut. Cell Host Microbe 2020;28:724–40. 10.1016/j.chom.2020.08.003 32841606 PMC7443397

[23] Zuo T , Sun Y , Wan Y , et al . Human-Gut-DNA Virome variations across geography, ethnicity, and urbanization. Cell Host Microbe 2020;28:741–51. 10.1016/j.chom.2020.08.005 32910902

[24] Dutilh BE , Cassman N , McNair K , et al . A highly abundant bacteriophage discovered in the unknown sequences of human faecal metagenomes. Nat Commun 2014;5:4498. 10.1038/ncomms5498 25058116 PMC4111155

[25] Deveau H , Garneau JE , Moineau S . Crispr/Cas system and its role in phage-bacteria interactions. Annu Rev Microbiol 2010;64:475–93. 10.1146/annurev.micro.112408.134123 20528693

[26] Nakatsu G , Zhou H , Wu WKK , et al . Alterations in enteric Virome are associated with colorectal cancer and survival outcomes. Gastroenterology 2018;155:529–41. 10.1053/j.gastro.2018.04.018 29689266

[27] Ma Y , You X , Mai G , et al . A human gut phage catalog correlates the gut phageome with type 2 diabetes. Microbiome 2018;6:24. 10.1186/s40168-018-0410-y 29391057 PMC5796561

[28] Han M , Yang P , Zhong C , et al . The human gut Virome in hypertension. Front Microbiol 2018;9:3150. 10.3389/fmicb.2018.03150 30619215 PMC6305721

[29] Zhao G , Vatanen T , Droit L , et al . Intestinal virome changes precede autoimmunity in type I diabetes-susceptible children. Proc Natl Acad Sci U S A 2017;114:E6166–75. 10.1073/pnas.1706359114 28696303 PMC5544325

[30] Clooney AG , Sutton TDS , Shkoporov AN , et al . Whole-Virome analysis sheds light on viral dark matter in inflammatory bowel disease. Cell Host Microbe 2019;26:764–78. 10.1016/j.chom.2019.10.009 31757768

[31] Mangalea MR , Paez-Espino D , Kieft K , et al . Individuals at risk for rheumatoid arthritis harbor differential intestinal bacteriophage communities with distinct metabolic potential. Cell Host Microbe 2021;29:726–39. 10.1016/j.chom.2021.03.020 33957082 PMC8186507

[32] Kishikawa T , Maeda Y , Nii T , et al . Metagenome-wide association study of gut microbiome revealed novel aetiology of rheumatoid arthritis in the Japanese population. Ann Rheum Dis 2020;79:103–11. 10.1136/annrheumdis-2019-215743 31699813 PMC6937407

[33] Kishikawa T , Ogawa K , Motooka D , et al . A Metagenome-Wide association study of gut microbiome in patients with multiple sclerosis revealed novel disease pathology. Front Cell Infect Microbiol 2020;10:585973. 10.3389/fcimb.2020.585973 33363050 PMC7759502

[34] Tomofuji Y , Maeda Y , Oguro-Igashira E , et al . Metagenome-wide association study revealed disease-specific landscape of the gut microbiome of systemic lupus erythematosus in Japanese. Ann Rheum Dis 2021;80:1575–83. 10.1136/annrheumdis-2021-220687 34426398 PMC8600607

[35] Aletaha D , Neogi T , Silman AJ , et al . 2010 rheumatoid arthritis classification criteria: an American College of Rheumatology/European League against rheumatism collaborative initiative. Arthritis Rheum 2010;62:2569–81. 10.1002/art.27584 20872595

[36] Petri M , Orbai A-M , Alarcón GS , et al . Derivation and validation of the systemic lupus international collaborating clinics classification criteria for systemic lupus erythematosus. Arthritis Rheum 2012;64:2677–86. 10.1002/art.34473 22553077 PMC3409311

[37] Polman CH , Reingold SC , Banwell B , et al . Diagnostic criteria for multiple sclerosis: 2010 revisions to the McDonald criteria. Ann Neurol 2011;69:292–302. 10.1002/ana.22366 21387374 PMC3084507

[38] Matsui T , Kuga Y , Kaneko A , et al . Disease activity score 28 (DAS28) using C-reactive protein underestimates disease activity and overestimates EULAR response criteria compared with DAS28 using erythrocyte sedimentation rate in a large observational cohort of rheumatoid arthritis patients in Japan. Ann Rheum Dis 2007;66:1221–6. 10.1136/ard.2006.063834 17369281 PMC1955164

[39] Petri M , Kim MY , Kalunian KC , et al . Combined oral contraceptives in women with systemic lupus erythematosus. N Engl J Med 2005;353:2550–8. 10.1056/NEJMoa051135 16354891

[40] Kurtzke JF . Rating neurologic impairment in multiple sclerosis: an expanded disability status scale (EDSS). Neurology 1983;33:1444–52. 10.1212/WNL.33.11.1444 6685237

[41] Roux S , Enault F , Hurwitz BL , et al . VirSorter: mining viral signal from microbial genomic data. PeerJ 2015;3:e985. 10.7717/peerj.985 26038737 PMC4451026

[42] Ren J , Ahlgren NA , Lu YY , et al . VirFinder: a novel k-mer based tool for identifying viral sequences from assembled metagenomic data. Microbiome 2017;5:69. 10.1186/s40168-017-0283-5 28683828 PMC5501583

[43] Simão FA , Waterhouse RM , Ioannidis P , et al . BUSCO: assessing genome assembly and annotation completeness with single-copy orthologs. Bioinformatics 2015;31:3210–2. 10.1093/bioinformatics/btv351 26059717

[44] Paez-Espino D , Pavlopoulos GA , Ivanova NN , et al . Nontargeted virus sequence discovery pipeline and virus clustering for metagenomic data. Nat Protoc 2017;12:1673–82. 10.1038/nprot.2017.063 28749930

[45] Yutin N , Makarova KS , Gussow AB , et al . Discovery of an expansive bacteriophage family that includes the most abundant viruses from the human gut. Nat Microbiol 2018;3:38–46. 10.1038/s41564-017-0053-y 29133882 PMC5736458

[46] Bland C , Ramsey TL , Sabree F , et al . Crispr recognition tool (crt): a tool for automatic detection of clustered regularly interspaced palindromic repeats. BMC Bioinformatics 2007;8:209. 10.1186/1471-2105-8-209 17577412 PMC1924867

[47] Acosta-Herrera M , Kerick M , González-Serna D , et al . Genome-wide meta-analysis reveals shared new loci in systemic seropositive rheumatic diseases. Ann Rheum Dis 2019;78:311–9. 10.1136/annrheumdis-2018-214127 30573655 PMC6800208

[48] Koonin EV , Yutin N . The crAss-like phage group: how Metagenomics reshaped the human Virome. Trends Microbiol 2020;28:349–59. 10.1016/j.tim.2020.01.010 32298613

[49] Ferreira-Halder CV , Faria AVdeS , Andrade SS . Action and function of Faecalibacterium prausnitzii in health and disease. Best Pract Res Clin Gastroenterol 2017;31:643–8. 10.1016/j.bpg.2017.09.011 29566907

[50] Mandl T , Marsal J , Olsson P , et al . Severe intestinal dysbiosis is prevalent in primary Sjögren's syndrome and is associated with systemic disease activity. Arthritis Res Ther 2017;19:237. 10.1186/s13075-017-1446-2 29065905 PMC5655865

[51] Yin J , Sternes PR , Wang M , et al . Shotgun metagenomics reveals an enrichment of potentially cross-reactive bacterial epitopes in ankylosing spondylitis patients, as well as the effects of TNFi therapy upon microbiome composition. Ann Rheum Dis 2020;79:132–40. 10.1136/annrheumdis-2019-215763 31662318

[52] Jonge PAde , Meijenfeldt FABvon , Rooijen LEvan , et al . Evolution of bacon domain tandem repeats in crAssphage and novel gut bacteriophage lineages. Viruses 2019;11:1085. 10.3390/v11121085 PMC6949934 31766550

[53] Shkoporov AN , Khokhlova EV , Fitzgerald CB , et al . ΦCrAss001 represents the most abundant bacteriophage family in the human gut and infects Bacteroides intestinalis. Nat Commun 2018;9:4781. 10.1038/s41467-018-07225-7 30429469 PMC6235969

[54] Azzouz D , Omarbekova A , Heguy A , et al . Lupus nephritis is linked to disease-activity associated expansions and immunity to a gut commensal. Ann Rheum Dis 2019;78:947–56. 10.1136/annrheumdis-2018-214856 30782585 PMC6585303

[55] Rogier R , Ederveen THA , Boekhorst J , et al . Aberrant intestinal microbiota due to IL-1 receptor antagonist deficiency promotes IL-17- and TLR4-dependent arthritis. Microbiome 2017;5:63. 10.1186/s40168-017-0278-2 28645307 PMC5481968

[56] Yutin N , Benler S , Shmakov SA , et al . Analysis of metagenome-assembled viral genomes from the human gut reveals diverse putative CrAss-like phages with unique genomic features. Nat Commun 2021;12:1044. 10.1038/s41467-021-21350-w 33594055 PMC7886860

[57] Guerin E , Shkoporov A , Stockdale SR , et al . Biology and taxonomy of crAss-like bacteriophages, the most abundant virus in the human gut. Cell Host Microbe 2018;24:653–64. 10.1016/j.chom.2018.10.002 30449316

[58] Ríos-Covián D , Ruas-Madiedo P , Margolles A , et al . Intestinal short chain fatty acids and their link with diet and human health. Front Microbiol 2016;7:185. 10.3389/fmicb.2016.00185 26925050 PMC4756104

