# Supplementary material for: Whole gut virome analysis of 476 Japanese revealed a link between phage and autoimmune disease
Source: Ann Rheum Dis. 2021 Dec 8;81(2):278–88. doi: 10.1136/annrheumdis-2021-221267 (PMC8761997; doi:10.1136/annrheumdis-2021-221267)
Supplement: Supplementary data [file annrheumdis-2021-221267supp001.pdf]

# Supplementary information

## A whole gut virome analysis of 476 Japanese revealed a link between phage and autoimmune disease

Tomofuji Y et al.

Corresponding to Yukinori Okada (yokada@sg.med.osaka-u.ac.jp)

### Table of contents

**■Supplementary Figures ........ 1**

1. Pipeline for virome analysis from shotgun sequencing data ........ 1
2. Bacterial relative abundance at the genus level (L6) ........ 2
3. Association tests for disease-specific virus-bacterium interaction ........ 3
4. Analysis of the bacterial target of viruses based on the CRISPR spacers ........ 4

**■Supplementary Tables ........ 5**

1. Characteristics of the study population ........ 5
2. Detection ratio of the viral clades ........ 8
3. Association of age and sex to the viral abundances ........ 9
4. Sub-analysis of the crAss-like phage abundance for RA ........ 10
5. Sub-analysis of the viral abundance for SLE ........ 11
6. Sub-analysis of the crAss-like phage abundance for combined autoimmune diseases (RA, SLE, and MS) ........ 12
7. Meta-analysis for the association between combined autoimmune diseases (RA, SLE, and MS) and the viral abundances ........ 13
8. Case–control comparison of the absence/presence state of crAss-like phages for combined autoimmune diseases (RA, SLE, and MS) ........ 14

9. Case–control comparison of *crAssphage* (NC_024711.1) and other crAss-like phages for combined autoimmune diseases (RA, SLE, and MS) ............................................. 15

10. Virus-bacterium pairs with significant association ................................................. 16

11. Sub-analysis for the virus-bacterium association analysis .................................... 17

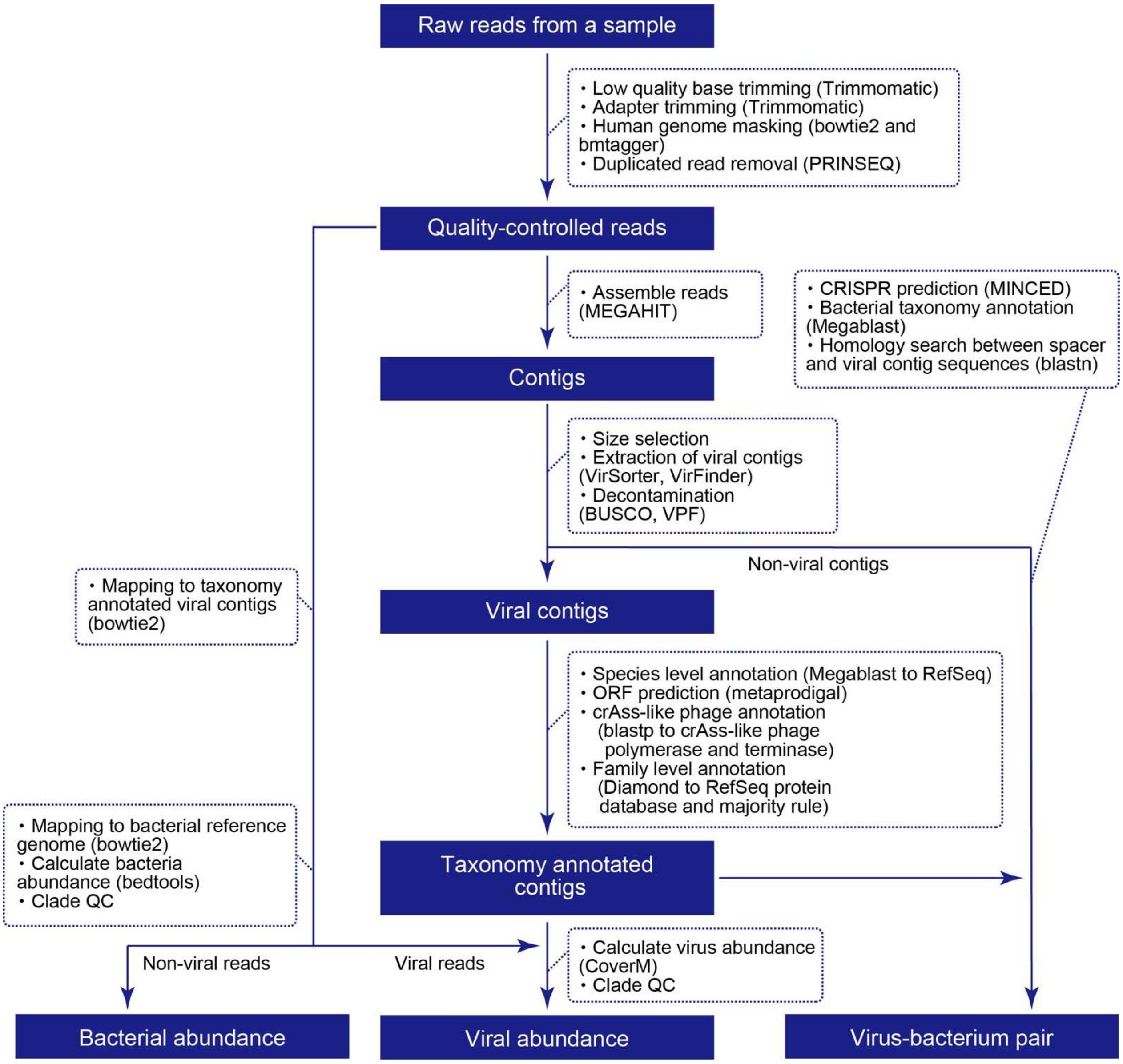


**Supplementary Figure 1. Pipeline for virome analysis from shotgun sequencing data.** Whole-genome shotgun sequencing reads of the gut microbiome were processed following this pipeline. This pipeline produces three outputs (bacterial abundance, viral abundance, and virus-bacterium pair). CRISPR, clustered regularly interspaced short palindromic repeat; ORF, open reading frame; QC, quality check; RefSeq, NCBI Reference Sequence Database.

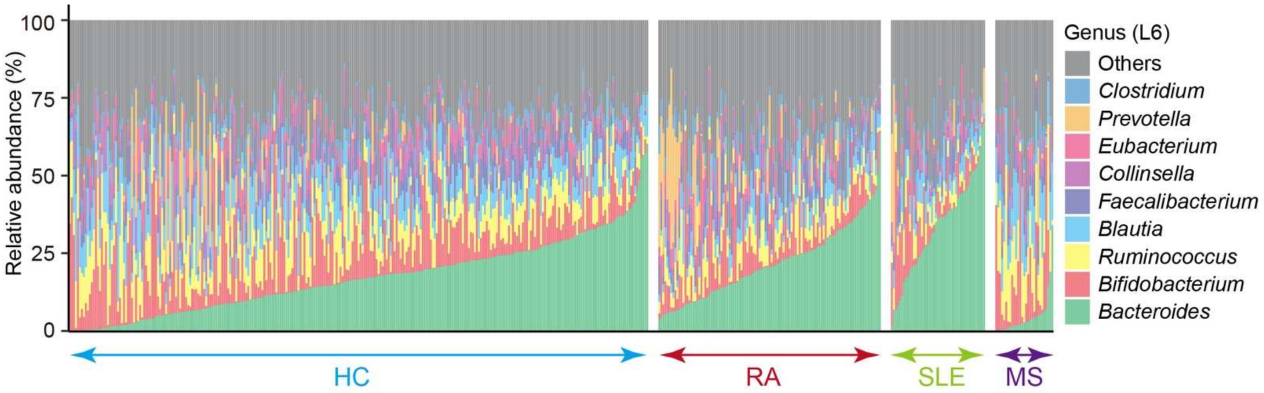


**Supplementary Figure 2. Bacterial relative abundance at the genus level (L6).**

HC, healthy control; MS, multiple sclerosis; RA, rheumatoid arthritis; SLE, systemic lupus erythematosus.



Tomofuji Y, *et al*. *Ann Rheum Dis* 2022; 781:278–288. doi: 10.1136/annrheumdis-2021-221267

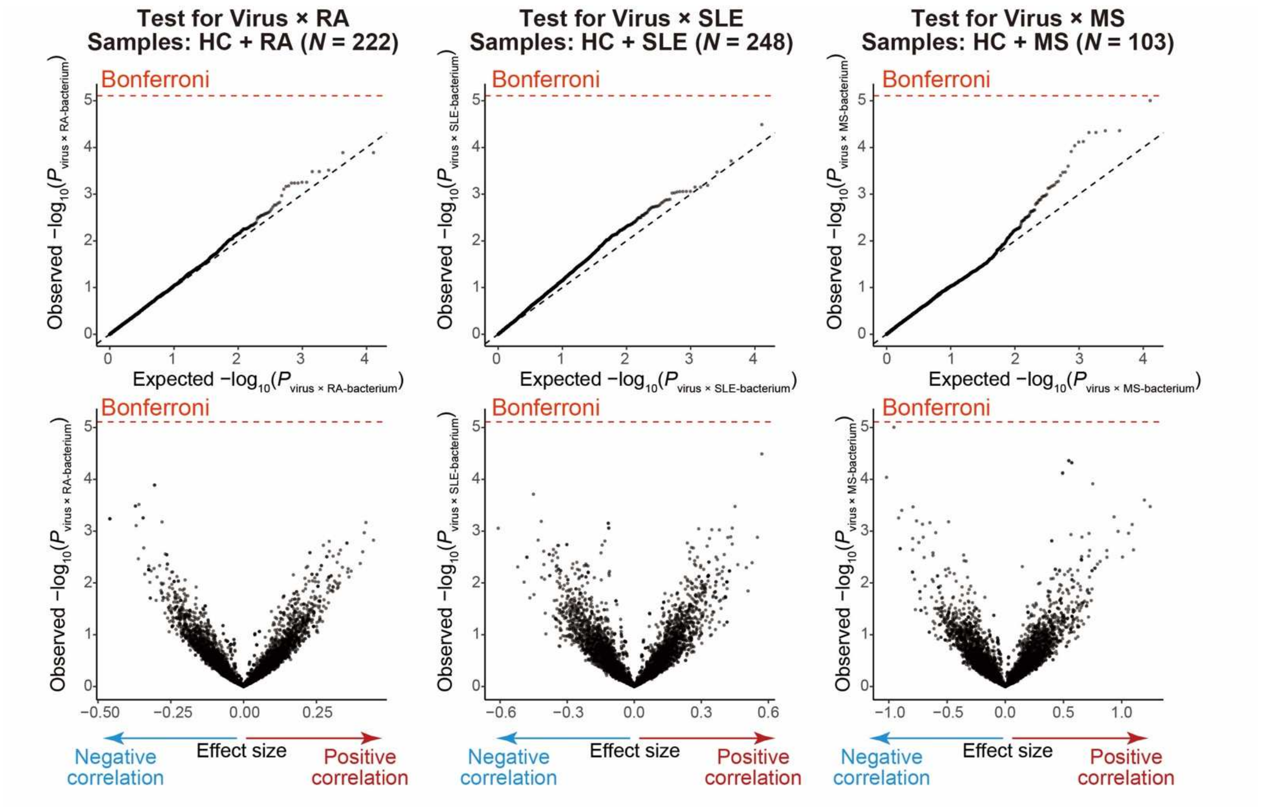


**Supplementary Figure 3. Association tests for disease-specific virus-bacterium interaction. (Top),** A quantile–quantile plot of the p-values from the disease specific virus-bacterium association analysis (left RA, middle SLE, right MS). The x-axis indicates $-\log_{10}(P_{\text{virus × disease-bacterium}})$ expected from uniform distribution. The y-axis indicates observed $-\log_{10}(P_{\text{virus × disease-bacterium}})$. The diagonal dashed line represents $y = x$, which corresponds to the null hypothesis. The horizontal red dashed line indicates the Bonferroni-corrected threshold ($\alpha = 0.05$). **(Bottom)**, A volcano plot. The x-axis indicates the effect sizes of virus × diseases term in linear regression. The y-axis, horizontal dashed lines are the same as in (top). HC, healthy control; MS, multiple sclerosis; RA, rheumatoid arthritis; SLE, systemic lupus erythematosus.



Tomofuji Y, *et al. Ann Rheum Dis* 2022; 781:278–288. doi: 10.1136/annrheumdis-2021-221267

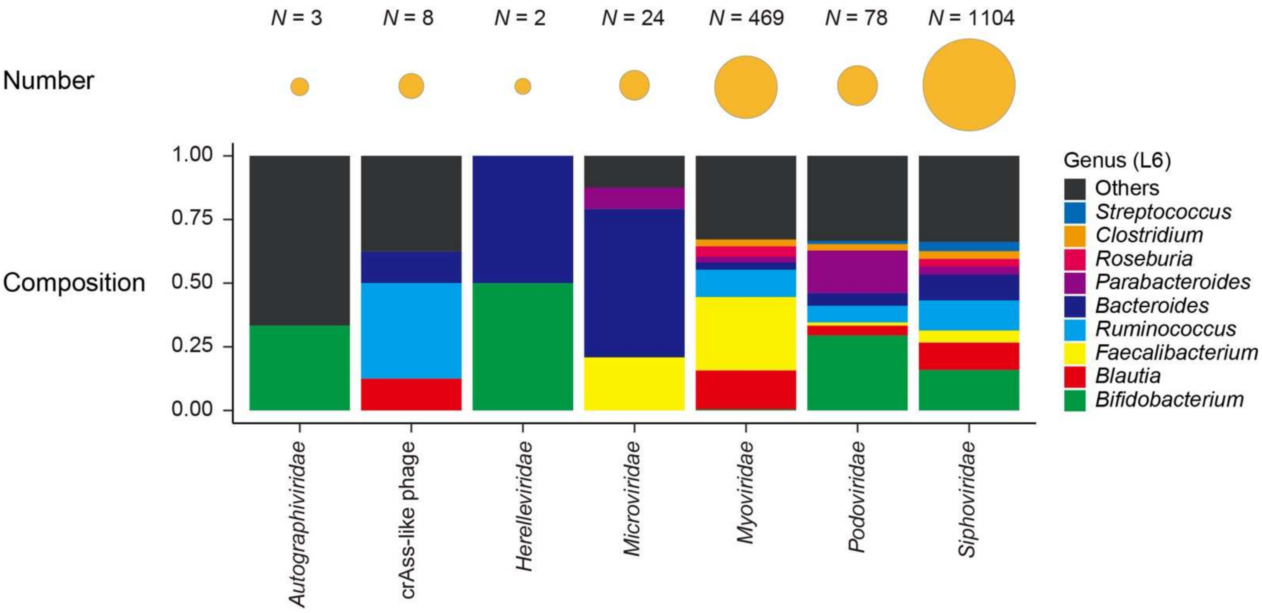


**Supplementary Figure 4. Analysis of the bacterial target of viruses based on the CRISPR spacers.**

Composition of bacterial genus with CRISPR spacers related to each viral family is represented as bar plot. Numbers of bacterium-virus pairs detected in this analysis are indicated above the bar. CRISPR, clustered regularly interspaced short palindromic repeat; HC, healthy control; MS, multiple sclerosis; RA, rheumatoid arthritis; SLE, systemic lupus erythematosus.



Tomofuji Y, *et al. Ann Rheum Dis* 2022; 781:278–288. doi: 10.1136/annrheumdis-2021-221267

**Supplementary Table 1. Characteristics of the study population.**

| | RA (*N* = 111) | SLE (*N* = 47) | MS (*N* = 29) | Control (*N* = 289) |
|---|---|---|---|---|
| Mean age (sd) | 61.4 (15.0) | 42.9 (15.9) | 45.6 (9.0) | 33.2 (9.0) |
| Sequencing batch 1 | 15 (13.5%) | 0 | 0 | 14 (4.8%) |
| Sequencing batch 2 | 67 (60.4%) | 5 (10.6%) | 0 | 27 (9.3%) |
| Sequencing batch 3 | 29 (26.1%) | 10 (21.3%) | 0 | 70 (24.2%) |
| Sequencing batch 4 | 0 | 0 | 29 (100%) | 74 (25.6%) |
| Sequencing batch 5 | 0 | 32 (68.1%) | 0 | 104 (36.0%) |
| Female | 88 (79.3%) | 43 (91.5%) | 24 (82.8%) | 140 (46.2%) |
| New onset | 70 (63.1%) | 18 (38.3%) | 2 (6.9%) | - |
| Not new onset | 24 (21.6%) | 29 (61.7%) | 27 (93.1%) | - |
| Antibiotics use | 0 | 11 (23.4%) | 0 | 0 |
| Proton pump inhibitor use | 13 (11.7%) | 27 (57.4%) | 4 (13.8%) | - |
| No treatment | 63 (56.8%) | 14 (29.8%) | 2 (6.9%) | - |
| With treatment | 43 (38.7%) | 33 (70.2%) | 27 (93.1%) | - |
| Comorbidity | | | | |
| Diabetes | 7 (6.3%) | 1 (2.1%) | 0 | - |
| Hypertension | 21 (18.9%) | 4 (8.5%) | 3 (10.3%) | - |
| Dyslipidemia | 19 (17.1%) | 5 (10.6%) | 3 (10.3%) | - |
| Cardiovascular disorders | 4 (3.6%) | 1 (2.1%) | 0 | - |
| Not evaluated | 6 (5.4%) | 0 | 0 | - |
| Treatment for RA | | | | |
| Steroid | 15 (13.5%) | - | - | - |
| Methotrexate | 26 (23.4%) | - | - | - |
| Salazosulfapyridine | 11 (9.9%) | - | - | - |
| Iguratimod | 1 (0.9%) | - | - | - |
| Tacrolimus | 1 (0.9%) | - | - | - |
| Certolizumab pegol | 1 (0.9%) | - | - | - |
| Abatacept | 1 (0.9%) | - | - | - |
| Atlizumab | 1 (0.9%) | - | - | - |
| DAS-C, mean (sd) | 4.1 (1.3) | - | - | - |
| DAS-C > 4.1 | 48 (43.2%) | - | - | - |
| Rheumatoid factor | | | | |
| Strong positive | 60 (54.1%) | - | - | - |
| Positive | 19 (17.1%) | - | - | - |
| Negative | 27 (24.3%) | - | - | - |
| Not evaluated | 5 (4.5%) | - | - | - |
| ACPA | | | | |
| Strong positive | 62 (55.9%) | - | - | - |
| Positive | 10 (9.0%) | - | - | - |
| Negative | 31 (27.9%) | - | - | - |

| | | | | |
|---|---|---|---|---|
| Not evaluated | 8 (7.2%) | - | - | - |
| Sero-positivity | | | | |
| Positive | 85 (76.6%) | - | - | - |
| Negative | 26 (23.4%) | - | - | - |
| Steinbrocker stage | | | | |
| I | 58 (52.2%) | - | - | - |
| II | 18 (16.2%) | - | - | - |
| III | 3 (2.7%) | - | - | - |
| IV | 3 (2.7%) | - | - | - |
| Not evaluated | 29 (26.1%) | - | - | - |
| Interstitial lung disease | 6 (5.4%) | - | - | - |
| Treatment for SLE | | | | |
| Steroid | - | 32 (68.1%) | - | - |
| Hydroxychloroquine | - | 7 (14.9%) | - | - |
| Tacrolimus | - | 5 (10.6%) | - | - |
| Ciclosporin | - | 1 (2.1%) | - | - |
| Mycophenolate Mofetil | - | 8 (17%) | - | - |
| Cyclophosphamide | - | 1 (2.1%) | - | - |
| SLE-DAI, mean (sd) | - | 11.1 (8.7) | - | - |
| SLE-DAI ≥ 8 | - | 28 (59.6%) | - | - |
| Manifestation of SLE | | | | |
| Psychosis | - | 1 (2.1%) | - | - |
| Vision impairment | - | 1 (2.1%) | - | - |
| Headache | - | 4 (8.5%) | - | - |
| Cerebrovascular disease | - | 1 (2.1%) | - | - |
| Vasculitis | - | 1 (2.1%) | - | - |
| Arthritis | - | 14 (29.8%) | - | - |
| Myositis | - | 2 (4.3%) | - | - |
| Urinary cast | - | 9 (19.1%) | - | - |
| Hematuria | - | 5 (10.6%) | - | - |
| Proteinuria | - | 13 (27.7%) | - | - |
| Pyuria | - | 7 (14.9%) | - | - |
| Rash | - | 15 (31.9%) | - | - |
| Alopecia | - | 7 (14.9%) | - | - |
| Mucosal ulcer | - | 5 (10.6%) | - | - |
| Pleurisy | - | 5 (10.6%) | - | - |
| Pericarditis | - | 2 (4.3%) | - | - |
| Low complement | - | 34 (72.3%) | - | - |
| Increased DNA binding | - | 30 (63.8%) | - | - |
| Fever | - | 14 (29.8%) | - | - |
| Thombocytopenia | - | 2 (4.3%) | - | - |

| | | | | |
|---|---|---|---|---|
| Leucopenia | - | 12 (25.5%) | - | - |
| Seizure | - | 0 | - | - |
| Organic brain syndrome | - | 0 | - | - |
| Cranial nerve disorder | - | 0 | - | - |
| Lupus nephritis | - | 20 (42.6 %) | - | - |
| Treatment for MS | | | | |
| Steroid | - | - | 6 (20.7%) | - |
| Dimethyl fumarate | - | - | 9 (31.0%) | - |
| Fingolimod | - | - | 7 (24.1%) | - |
| Interferon | - | - | 4 (13.8%) | - |
| Glatiramer acetate | - | - | 6 (20.7%) | - |
| EDSS, mean (sd) | - | - | 2.6 (2.0) | - |
| EDSS ≥ 4.5 | - | - | 8 (27.6%) | - |
| Spinal cord involvement | - | - | 18 (62.1%) | - |

ACPA, anti-citrullinated protein/peptide antibody; DAS-C, Disease Activity Score 28 using C-reactive protein; EDSS, Expanded Disability Status Scale; MS, multiple sclerosis; RA, rheumatoid arthritis; RF, rheumatoid factor; sd, standard deviation; SLE, systemic lupus erythematosus; SLE-DAI, SLE Disease Activity Index.

[*1] Information about the date of onset could not be obtained for 17 RA patients.

[*2] Information about treatment and proton pump inhibitor usage could not be obtained for five RA patients.

[*3] Information about the presence of interstitial lung disease for six RA patients.

[*4] DAS-C could not be obtained for 11 RA patients.

[*5] Information about antibiotics usage could not be obtained for a SLE patient.



Tomofuji Y, *et al*. *Ann Rheum Dis* 2022; 781:278–288. doi: 10.1136/annrheumdis-2021-221267

**Supplementary Table 2. Detection ratio of the viral clades.**

| | Sequencing batch 1 | Sequencing batch 2 | Sequencing batch 3 | Sequencing batch 4 | Sequencing batch 5 |
|---|---|---|---|---|---|
| Number of samples | 29 | 99 | 109 | 103 | 136 |
| Detection ratio (%) | | | | | |
| *Autographiviridae* | 41.4 | 29.3 | 30.3 | 21.4 | 25.0 |
| crAss-like phage | 72.4 | 62.6 | 64.2 | 66.0 | 63.2 |
| *Herelleviridae* | 65.5 | 42.4 | 33.0 | 21.4 | 38.2 |
| *Microviridae* | 55.2 | 51.5 | 45.9 | 24.3 | 62.5 |
| *Myoviridae* | 100.0 | 100.0 | 100.0 | 100.0 | 100.0 |
| *Phycodnaviridae* | 86.2 | 53.5 | 61.5 | 44.7 | 63.2 |
| *Podoviridae* | 96.6 | 93.9 | 98.2 | 95.1 | 97.1 |
| *Siphoviridae* | 100.0 | 100.0 | 100.0 | 100.0 | 100.0 |

**Supplementary Table 3. Association of age and sex to the viral abundances.**

Age ($N$ = 476)

| Viruses | Effect size | SE | *P* |
|---|---|---|---|
| *Autographiviridae* | $3.8 \times 10^{-4}$ | 0.0020 | 0.85 |
| crAss-like phage | 0.0064 | 0.0036 | 0.076 |
| *Herelleviridae* | 0.0031 | 0.0021 | 0.13 |
| *Microviridae* | $3.4 \times 10^{-5}$ | 0.0025 | 0.99 |
| *Myoviridae* | 0.0016 | $8.3 \times 10^{-4}$ | 0.047 |
| *Phycodnaviridae* | −0.0016 | 0.0027 | 0.54 |
| *Podoviridae* | 0.0026 | 0.0021 | 0.21 |
| *Siphoviridae* | $7.8 \times 10^{-4}$ | $4.5 \times 10^{-4}$ | 0.087 |

Male ($N$ = 476)

| Viruses | Effect size | SE | *P* |
|---|---|---|---|
| *Autographiviridae* | −0.026 | 0.056 | 0.64 |
| crAss-like phage | −0.024 | 0.10 | 0.81 |
| *Herelleviridae* | 0.029 | 0.059 | 0.63 |
| *Microviridae* | 0.047 | 0.072 | 0.51 |
| *Myoviridae* | 0.035 | 0.024 | 0.14 |
| *Phycodnaviridae* | 0.040 | 0.077 | 0.61 |
| *Podoviridae* | −0.027 | 0.059 | 0.64 |
| *Siphoviridae* | −0.0060 | 0.013 | 0.64 |

SE, standard error.



Tomofuji Y, *et al. Ann Rheum Dis* 2022; 781:278–288. doi: 10.1136/annrheumdis-2021-221267

**Supplementary Table 4. Sub-analysis of the crAss-like phage abundance for RA.**

| Case–control comparison | | | |
|---|---|---|---|
| Sample set | Effect size | SE | *P* |
| All | −0.476 | 0.173 | 0.0060 |
| Remove male | −0.568 | 0.197 | 0.0040 |
| Remove non-new onset patients | −0.385 | 0.200 | 0.055 |
| Remove patients with proton pump inhibitors | −0.441 | 0.183 | 0.016 |
| Remove patients with steroids | −0.475 | 0.183 | 0.010 |
| Remove patients with treatment for RA | −0.520 | 0.211 | 0.014 |
| Comparison within case | | | |
| Objective | Effect size | SE | *P* |
| Not treated | −0.008 | 0.207 | 0.97 |
| New onset | 0.313 | 0.271 | 0.25 |
| DAS-C > 4.1 | 0.018 | 0.219 | 0.93 |
| CRP > 1.0 mg / L ($N_{\text{CRP high}}$ = 52 vs $N_{\text{CRP low}}$ = 52) | 0.291 | 0.227 | 0.20 |

CRP, C-reactive protein; DAS-C, Disease Activity Score 28 (DAS28) using C-reactive protein; SE, standard error; RA, rheumatoid arthritis.

**Supplementary Table 5. Sub-analysis of the viral abundance for SLE.**

**crAss-like phage**

| Case–control comparison | | | |
|---|---|---|---|
| Sample set | Effect size | SE | *P* |
| All | −0.514 | 0.206 | 0.012 |
| Remove male | −0.484 | 0.218 | 0.027 |
| Remove non-new onset patients | −0.946 | 0.356 | 0.0078 |
| Remove patients with antibiotics | −0.372 | 0.225 | 0.099 |
| Remove patients with proton pump inhibitors | −0.724 | 0.319 | 0.023 |
| Remove patients with steroids | −0.799 | 0.353 | 0.023 |
| Remove patients with treatment for SLE | −0.945 | 0.399 | 0.018 |
| Comparison within case | | | |
| Objective | Effect size | SE | *P* |
| Not treated | −0.806 | 0.564 | 0.15 |
| New onset | −0.593 | 0.580 | 0.31 |
| SLE-DAI ≥ 8 | −1.147 | 0.634 | 0.070 |

***Podoviridae***

| Case–control comparison | | | |
|---|---|---|---|
| Sample set | Effect size | SE | *P* |
| All | −0.947 | 0.330 | 0.0041 |
| Remove male | −0.653 | 0.357 | 0.067 |
| Remove non-new onset patients | −1.231 | 0.469 | 0.0087 |
| Remove patients with antibiotics | −0.601 | 0.371 | 0.11 |
| Remove patients with proton pump inhibitors | −0.591 | 0.519 | 0.26 |
| Remove patients with steroids | −1.238 | 0.492 | 0.012 |
| Remove patients with treatment for SLE | −1.350 | 0.519 | 0.0093 |
| Comparison within case | | | |
| Objective | Effect size | SE | *P* |
| Not treated | −0.289 | 0.593 | 0.63 |
| New onset | −0.165 | 0.573 | 0.77 |
| SLE-DAI ≥ 8 | 0.313 | 0.736 | 0.67 |

SE, standard error; SLE, systemic lupus erythematosus; SLE-DAI, SLE Disease Activity Index.



Tomofuji Y, *et al. Ann Rheum Dis* 2022; 781:278–288. doi: 10.1136/annrheumdis-2021-221267

**Supplementary Table 6. Sub-analysis of the crAss-like phage abundance for combined autoimmune diseases (RA, SLE, and MS).**

| Case–control comparison | | | |
|---|---|---|---|
| Sample set | Effect size | SE | *P* |
| All | −0.429 | 0.126 | $6.5 \times 10^{-4}$ |
| Remove male | −0.464 | 0.140 | $9.2 \times 10^{-4}$ |
| Remove non-new onset patients | −0.534 | 0.166 | 0.0013 |
| Remove patients with antibiotics | −0.387 | 0.131 | 0.0031 |
| Remove patients with proton pump inhibitors | −0.417 | 0.140 | 0.0029 |
| Remove patients with steroids | −0.406 | 0.147 | 0.0057 |
| Remove patients with treatment for combined autoimmune diseases | −0.549 | 0.180 | 0.0022 |
| Remove sequencing batch 4 | −0.499 | 0.137 | $2.6 \times 10^{-4}$ |
| Case–control comparison (Per sequencing batch analysis) | | | |
| Sequencing batch | Effect size | SE | *P* |
| Batch 1 | −0.988 | 0.551 | 0.073 |
| Batch 2 | −0.270 | 0.248 | 0.28 |
| Batch 3 | −0.437 | 0.296 | 0.14 |
| Batch 4 | 0.375 | 0.463 | 0.42 |
| Batch 5 | −0.451 | 0.269 | 0.094 |
| Comparison within the case | | | |
| Objective | Effect size | SE | *P* |
| Not treated | −0.087 | 0.179 | 0.63 |
| New onset | −0.007 | 0.204 | 0.97 |

SE, standard error.

**Supplementary Table 7. Meta-analysis for the association between combined autoimmune diseases (RA, SLE, and MS) and the viral abundances.**

| | Fixed-effect meta-analysis | | | Random effect meta-analysis | | | | |
|---|---|---|---|---|---|---|---|---|
| Viruses | Effect size | SE | *P* | Effect size | SE | *P* | *Q* | *Q-P* |
| *Autographiviridae* | 0.027 | 0.241 | 0.91 | 0.027 | 0.241 | 0.91 | 0.51 | 0.77 |
| crAss-like phage | −0.392 | 0.139 | 0.0047 | −0.392 | 0.139 | 0.0047 | 3.03 | 0.22 |
| *Herelleviridae* | −0.474 | 0.267 | 0.076 | −0.474 | 0.267 | 0.076 | 3.71 | 0.16 |
| *Microviridae* | 0.302 | 0.193 | 0.12 | 0.302 | 0.193 | 0.12 | 1.32 | 0.52 |
| *Myoviridae* | 0.169 | 0.569 | 0.77 | 0.030 | 0.688 | 0.97 | 2.08 | 0.35 |
| *Phycodnaviridae* | −0.131 | 0.194 | 0.50 | −0.150 | 0.225 | 0.51 | 2.36 | 0.31 |
| *Podoviridae* | −0.215 | 0.256 | 0.40 | −0.215 | 0.256 | 0.40 | 1.75 | 0.42 |
| *Siphoviridae* | −1.746 | 1.127 | 0.12 | −1.746 | 1.127 | 0.12 | 1.36 | 0.51 |

SE, standard error.

**Supplementary Table 8. Case–control comparison of the absence/presence state of crAss-like phages for combined autoimmune diseases (RA, SLE, and MS).**

| Viruses | Effect size | SE | *P* | *N* detection in case | *N* detection in control |
|---|---|---|---|---|---|
| crAss-like phage | −0.734 | 0.259 | 0.0046 | 113 (60.4%) | 194 (67.1%) |

SE, standard error.

**Supplementary Table 9. Case–control comparison of *crAssphage* (NC_024711.1) and other crAss-like phages for combined autoimmune diseases (RA, SLE, and MS).**

| Viruses | Effect size | SE | *P* |
|---|---|---|---|
| *crAssphage* (NC_024711.1) | −0.378 | 0.130 | 0.0036 |
| Other crAss-like phages | −0.374 | 0.159 | 0.019 |

SE, standard error.



Tomofuji Y, *et al. Ann Rheum Dis* 2022; 781:278–288. doi: 10.1136/annrheumdis-2021-221267

**Supplementary Table 10. Virus-bacterium pairs with significant association.**

| Viruses | Bacteria | Level of bacteria | Effect size | SE | $P_{\text{virus-bacterium}}$ | $q$ |
|---|---|---|---|---|---|---|
| *Podoviridae* | *Faecalibacterium sp.* | Species (L7) | 0.192 | 0.035 | $7.9 \times 10^{-8}$ | $5.1 \times 10^{-4}$ |
| *Podoviridae* | *Faecalibacterium cf. prausnitzii* | Species (L7) | 0.186 | 0.038 | $1.3 \times 10^{-6}$ | $4.2 \times 10^{-3}$ |

SE, standard error.



Tomofuji Y, *et al. Ann Rheum Dis* 2022; 781:278–288. doi: 10.1136/annrheumdis-2021-221267

**Supplementary Table 11. Sub-analysis for the virus-bacterium association analysis.**

***Podoviridae* and *Faecalibacterium sp.***

| Sample set | Effect size | SE | $P_{\text{virus-bacterium}}$ |
|---|---|---|---|
| All | 0.192 | 0.035 | $7.9 \times 10^{-8}$ |
| Remove male | 0.204 | 0.044 | $4.7 \times 10^{-6}$ |
| Remove non-new onset patients | 0.180 | 0.040 | $7.3 \times 10^{-6}$ |
| Only HC | 0.194 | 0.048 | $6.2 \times 10^{-5}$ |
| Remove patients with antibiotics | 0.179 | 0.035 | $5.8 \times 10^{-7}$ |
| Remove patients with proton pump inhibitors | 0.192 | 0.036 | $2.1 \times 10^{-7}$ |
| Remove patients with steroids | 0.188 | 0.038 | $8.4 \times 10^{-7}$ |
| Remove patients with treatment for combined autoimmune diseases (RA, SLE, and MS) | 0.198 | 0.041 | $1.6 \times 10^{-6}$ |

***Podoviridae* and *Faecalibacterium cf. prausnitzii***

| Sample set | Effect size | SE | $P_{\text{virus-bacterium}}$ |
|---|---|---|---|
| All | 0.186 | 0.038 | $1.3 \times 10^{-6}$ |
| Remove male | 0.191 | 0.047 | $5.7 \times 10^{-5}$ |
| Remove non-new onset patients | 0.189 | 0.042 | $1.1 \times 10^{-5}$ |
| Only HC | 0.209 | 0.051 | $6.0 \times 10^{-5}$ |
| Remove patients with antibiotics | 0.181 | 0.038 | $2.4 \times 10^{-6}$ |
| Remove patients with proton pump inhibitors | 0.200 | 0.040 | $6.4 \times 10^{-7}$ |
| Remove patients with steroids | 0.196 | 0.040 | $1.8 \times 10^{-6}$ |
| Remove patients with treatment for combined autoimmune diseases (RA, SLE, and MS) | 0.206 | 0.044 | $3.6 \times 10^{-6}$ |

HC, healthy control; MS, multiple sclerosis; RA, rheumatoid arthritis; SE, standard error; SLE, systemic lupus erythematosus.



Tomofuji Y, *et al. Ann Rheum Dis* 2022; 781:278–288. doi: 10.1136/annrheumdis-2021-221267
