# Supplementary material for: Whole gut virome analysis of 476 Japanese revealed a link between phage and autoimmune disease
Source: Ann Rheum Dis. 2021 Dec 8;81(2):278–88. doi: 10.1136/annrheumdis-2021-221267 (PMC8761997; doi:10.1136/annrheumdis-2021-221267)
Supplement: Supplementary data [file annrheumdis-2021-221267supp002.pdf]

Supplementary file. Full result of the virus-bacterium association test.

Viruses	Bacteria	Effect size	SE	*P*	*q*
Autographiviridae	L2_Deinococcus.Thermus	-0.008	0.012	4.95.E-01	9.55.E-01
crAss_like_phage	L2_Deinococcus.Thermus	-0.029	0.013	2.72.E-02	6.58.E-01
Herelleviridae	L2_Deinococcus.Thermus	-0.009	0.012	4.70.E-01	9.50.E-01
Microviridae	L2_Deinococcus.Thermus	0.028	0.012	2.05.E-02	6.01.E-01
Myoviridae	L2_Deinococcus.Thermus	0.008	0.014	5.44.E-01	9.60.E-01
Phycodnaviridae	L2_Deinococcus.Thermus	-0.003	0.012	8.14.E-01	9.93.E-01
Podoviridae	L2_Deinococcus.Thermus	0.005	0.014	7.40.E-01	9.88.E-01
Siphoviridae	L2_Deinococcus.Thermus	-0.005	0.013	6.77.E-01	9.80.E-01
Autographiviridae	L2_Verrucomicrobia	-0.009	0.039	8.17.E-01	9.93.E-01
crAss_like_phage	L2_Verrucomicrobia	0.014	0.043	7.51.E-01	9.89.E-01
Herelleviridae	L2_Verrucomicrobia	0.036	0.040	3.74.E-01	9.15.E-01
Microviridae	L2_Verrucomicrobia	-0.077	0.040	5.41.E-02	7.23.E-01
Myoviridae	L2_Verrucomicrobia	0.040	0.045	3.76.E-01	9.15.E-01
Phycodnaviridae	L2_Verrucomicrobia	0.054	0.040	1.72.E-01	8.54.E-01
Podoviridae	L2_Verrucomicrobia	0.090	0.046	5.14.E-02	7.22.E-01
Siphoviridae	L2_Verrucomicrobia	-0.011	0.041	7.95.E-01	9.92.E-01
Autographiviridae	L2_p_bacterium_LF.3	0.004	0.039	9.22.E-01	9.98.E-01
crAss_like_phage	L2_p_bacterium_LF.3	-0.031	0.043	4.74.E-01	9.52.E-01
Herelleviridae	L2_p_bacterium_LF.3	0.009	0.040	8.27.E-01	9.93.E-01
Microviridae	L2_p_bacterium_LF.3	-0.006	0.040	8.84.E-01	9.96.E-01
Myoviridae	L2_p_bacterium_LF.3	0.000	0.045	9.97.E-01	9.99.E-01
Phycodnaviridae	L2_p_bacterium_LF.3	-0.025	0.040	5.26.E-01	9.57.E-01
Podoviridae	L2_p_bacterium_LF.3	-0.024	0.046	6.00.E-01	9.70.E-01
Siphoviridae	L2_p_bacterium_LF.3	-0.028	0.041	4.92.E-01	9.55.E-01
Autographiviridae	L2_Euryarchaeota	0.049	0.036	1.71.E-01	8.50.E-01
crAss_like_phage	L2_Euryarchaeota	-0.031	0.040	4.35.E-01	9.26.E-01
Herelleviridae	L2_Euryarchaeota	0.071	0.037	5.81.E-02	7.24.E-01
Microviridae	L2_Euryarchaeota	0.048	0.037	2.00.E-01	8.71.E-01
Myoviridae	L2_Euryarchaeota	0.006	0.042	8.82.E-01	9.96.E-01
Phycodnaviridae	L2_Euryarchaeota	-0.018	0.037	6.18.E-01	9.75.E-01
Podoviridae	L2_Euryarchaeota	-0.016	0.043	7.18.E-01	9.86.E-01
Siphoviridae	L2_Euryarchaeota	-0.017	0.038	6.55.E-01	9.79.E-01
Autographiviridae	L2_Bacteroidetes	-0.013	0.015	3.86.E-01	9.15.E-01
crAss_like_phage	L2_Bacteroidetes	-0.026	0.017	1.18.E-01	7.86.E-01
Herelleviridae	L2_Bacteroidetes	-0.043	0.015	5.30.E-03	4.28.E-01
Microviridae	L2_Bacteroidetes	-0.018	0.016	2.39.E-01	8.85.E-01
Myoviridae	L2_Bacteroidetes	-0.025	0.017	1.48.E-01	8.20.E-01
Phycodnaviridae	L2_Bacteroidetes	-0.043	0.015	5.35.E-03	4.28.E-01
Podoviridae	L2_Bacteroidetes	-0.007	0.018	6.98.E-01	9.81.E-01
Siphoviridae	L2_Bacteroidetes	-0.042	0.016	9.38.E-03	4.94.E-01
Autographiviridae	L2_Actinobacteria	0.006	0.023	7.92.E-01	9.92.E-01
crAss_like_phage	L2_Actinobacteria	-0.043	0.025	9.44.E-02	7.64.E-01
Herelleviridae	L2_Actinobacteria	0.019	0.024	4.27.E-01	9.24.E-01
Microviridae	L2_Actinobacteria	0.000	0.024	9.96.E-01	9.99.E-01
Myoviridae	L2_Actinobacteria	-0.040	0.027	1.32.E-01	8.03.E-01
Phycodnaviridae	L2_Actinobacteria	0.005	0.024	8.47.E-01	9.95.E-01
Podoviridae	L2_Actinobacteria	-0.044	0.028	1.14.E-01	7.85.E-01
Siphoviridae	L2_Actinobacteria	-0.009	0.025	7.17.E-01	9.86.E-01
Autographiviridae	L2_Fusobacteria	-0.039	0.037	2.97.E-01	9.15.E-01
crAss_like_phage	L2_Fusobacteria	-0.091	0.041	2.80.E-02	6.58.E-01
Herelleviridae	L2_Fusobacteria	0.040	0.039	3.08.E-01	9.15.E-01
Microviridae	L2_Fusobacteria	0.001	0.039	9.71.E-01	9.99.E-01
Myoviridae	L2_Fusobacteria	0.069	0.043	1.16.E-01	7.85.E-01
Phycodnaviridae	L2_Fusobacteria	0.048	0.038	2.14.E-01	8.78.E-01
Podoviridae	L2_Fusobacteria	0.110	0.045	1.42.E-02	5.51.E-01
Siphoviridae	L2_Fusobacteria	-0.022	0.040	5.76.E-01	9.62.E-01
Autographiviridae	L2_Synergistetes	0.017	0.036	6.48.E-01	9.79.E-01
crAss_like_phage	L2_Synergistetes	0.023	0.040	5.70.E-01	9.61.E-01
Herelleviridae	L2_Synergistetes	0.044	0.038	2.44.E-01	8.88.E-01
Microviridae	L2_Synergistetes	-0.004	0.038	9.15.E-01	9.98.E-01
Myoviridae	L2_Synergistetes	0.017	0.042	6.89.E-01	9.80.E-01
Phycodnaviridae	L2_Synergistetes	0.021	0.037	5.82.E-01	9.64.E-01
Podoviridae	L2_Synergistetes	0.044	0.044	3.16.E-01	9.15.E-01

Siphoviridae	L2_Synergistetes	0.016	0.039	6.90.E-01	9.80.E-01
Autographiviridae	L2_Candidatus_Saccharibacteria	-0.026	0.037	4.76.E-01	9.52.E-01
crAss_like_phage	L2_Candidatus_Saccharibacteria	-0.008	0.041	8.44.E-01	9.95.E-01
Herelleviridae	L2_Candidatus_Saccharibacteria	-0.019	0.038	6.28.E-01	9.76.E-01
Microviridae	L2_Candidatus_Saccharibacteria	-0.008	0.039	8.45.E-01	9.95.E-01
Myoviridae	L2_Candidatus_Saccharibacteria	-0.054	0.043	2.13.E-01	8.78.E-01
Phycodnaviridae	L2_Candidatus_Saccharibacteria	-0.034	0.038	3.73.E-01	9.15.E-01
Podoviridae	L2_Candidatus_Saccharibacteria	-0.075	0.044	8.98.E-02	7.58.E-01
Siphoviridae	L2_Candidatus_Saccharibacteria	-0.049	0.039	2.17.E-01	8.79.E-01
Autographiviridae	L2_Proteobacteria	0.044	0.031	1.61.E-01	8.38.E-01
crAss_like_phage	L2_Proteobacteria	-0.026	0.034	4.44.E-01	9.32.E-01
Herelleviridae	L2_Proteobacteria	-0.015	0.032	6.51.E-01	9.79.E-01
Microviridae	L2_Proteobacteria	0.034	0.032	2.92.E-01	9.15.E-01
Myoviridae	L2_Proteobacteria	0.139	0.035	1.06.E-04	1.70.E-01
Phycodnaviridae	L2_Proteobacteria	0.021	0.032	5.14.E-01	9.57.E-01
Podoviridae	L2_Proteobacteria	0.014	0.037	7.06.E-01	9.83.E-01
Siphoviridae	L2_Proteobacteria	-0.001	0.033	9.83.E-01	9.99.E-01
Autographiviridae	L2_Firmicutes	0.014	0.024	5.65.E-01	9.61.E-01
crAss_like_phage	L2_Firmicutes	-0.001	0.027	9.69.E-01	9.99.E-01
Herelleviridae	L2_Firmicutes	-0.002	0.025	9.51.E-01	9.98.E-01
Microviridae	L2_Firmicutes	0.000	0.025	9.90.E-01	9.99.E-01
Myoviridae	L2_Firmicutes	0.089	0.028	1.51.E-03	3.12.E-01
Phycodnaviridae	L2_Firmicutes	0.019	0.025	4.43.E-01	9.32.E-01
Podoviridae	L2_Firmicutes	-0.006	0.029	8.46.E-01	9.95.E-01
Siphoviridae	L2_Firmicutes	-0.045	0.026	8.02.E-02	7.45.E-01
Autographiviridae	L2_Spirochaetes	-0.021	0.040	5.88.E-01	9.67.E-01
crAss_like_phage	L2_Spirochaetes	-0.039	0.044	3.70.E-01	9.15.E-01
Herelleviridae	L2_Spirochaetes	0.022	0.041	5.90.E-01	9.68.E-01
Microviridae	L2_Spirochaetes	-0.004	0.041	9.13.E-01	9.98.E-01
Myoviridae	L2_Spirochaetes	0.038	0.046	4.16.E-01	9.18.E-01
Phycodnaviridae	L2_Spirochaetes	-0.014	0.041	7.39.E-01	9.88.E-01
Podoviridae	L2_Spirochaetes	0.045	0.048	3.45.E-01	9.15.E-01
Siphoviridae	L2_Spirochaetes	-0.037	0.042	3.79.E-01	9.15.E-01
Autographiviridae	L3_Deinococci	-0.008	0.012	4.95.E-01	9.55.E-01
crAss_like_phage	L3_Deinococci	-0.029	0.013	2.72.E-02	6.58.E-01
Herelleviridae	L3_Deinococci	-0.009	0.012	4.70.E-01	9.50.E-01
Microviridae	L3_Deinococci	0.028	0.012	2.05.E-02	6.01.E-01
Myoviridae	L3_Deinococci	0.008	0.014	5.44.E-01	9.60.E-01
Phycodnaviridae	L3_Deinococci	-0.003	0.012	8.14.E-01	9.93.E-01
Podoviridae	L3_Deinococci	0.005	0.014	7.40.E-01	9.88.E-01
Siphoviridae	L3_Deinococci	-0.005	0.013	6.77.E-01	9.80.E-01
Autographiviridae	L3_Methanobacteria	0.048	0.036	1.84.E-01	8.60.E-01
crAss_like_phage	L3_Methanobacteria	-0.028	0.040	4.82.E-01	9.53.E-01
Herelleviridae	L3_Methanobacteria	0.067	0.037	7.32.E-02	7.26.E-01
Microviridae	L3_Methanobacteria	0.049	0.037	1.90.E-01	8.61.E-01
Myoviridae	L3_Methanobacteria	0.009	0.042	8.35.E-01	9.94.E-01
Phycodnaviridae	L3_Methanobacteria	-0.022	0.037	5.53.E-01	9.60.E-01
Podoviridae	L3_Methanobacteria	-0.017	0.043	7.02.E-01	9.81.E-01
Siphoviridae	L3_Methanobacteria	-0.017	0.038	6.63.E-01	9.79.E-01
Autographiviridae	L3_Bacteroidia	-0.013	0.015	3.86.E-01	9.15.E-01
crAss_like_phage	L3_Bacteroidia	-0.026	0.017	1.18.E-01	7.86.E-01
Herelleviridae	L3_Bacteroidia	-0.043	0.015	5.31.E-03	4.28.E-01
Microviridae	L3_Bacteroidia	-0.018	0.016	2.39.E-01	8.85.E-01
Myoviridae	L3_Bacteroidia	-0.025	0.017	1.49.E-01	8.22.E-01
Phycodnaviridae	L3_Bacteroidia	-0.043	0.015	5.35.E-03	4.28.E-01
Podoviridae	L3_Bacteroidia	-0.007	0.018	6.98.E-01	9.81.E-01
Siphoviridae	L3_Bacteroidia	-0.042	0.016	9.43.E-03	4.94.E-01
Autographiviridae	L3_Betaproteobacteria	0.055	0.034	1.04.E-01	7.82.E-01
crAss_like_phage	L3_Betaproteobacteria	0.043	0.038	2.58.E-01	8.95.E-01
Herelleviridae	L3_Betaproteobacteria	-0.046	0.035	1.97.E-01	8.66.E-01
Microviridae	L3_Betaproteobacteria	0.004	0.035	9.04.E-01	9.97.E-01
Myoviridae	L3_Betaproteobacteria	0.025	0.040	5.34.E-01	9.59.E-01
Phycodnaviridae	L3_Betaproteobacteria	0.000	0.035	9.95.E-01	9.99.E-01
Podoviridae	L3_Betaproteobacteria	-0.042	0.041	3.00.E-01	9.15.E-01
Siphoviridae	L3_Betaproteobacteria	-0.016	0.036	6.59.E-01	9.79.E-01

Autographiviridae	L3_Flavobacteriia	0.026	0.020	2.03.E-01	8.75.E-01
crAss_like_phage	L3_Flavobacteriia	-0.005	0.023	8.14.E-01	9.93.E-01
Herelleviridae	L3_Flavobacteriia	-0.026	0.021	2.15.E-01	8.79.E-01
Microviridae	L3_Flavobacteriia	0.009	0.021	6.60.E-01	9.79.E-01
Myoviridae	L3_Flavobacteriia	-0.026	0.024	2.72.E-01	9.02.E-01
Phycodnaviridae	L3_Flavobacteriia	0.009	0.021	6.66.E-01	9.80.E-01
Podoviridae	L3_Flavobacteriia	0.018	0.025	4.59.E-01	9.43.E-01
Siphoviridae	L3_Flavobacteriia	-0.039	0.022	7.22.E-02	7.25.E-01
Autographiviridae	L3_Synergistia	0.017	0.036	6.48.E-01	9.79.E-01
crAss_like_phage	L3_Synergistia	0.023	0.040	5.70.E-01	9.61.E-01
Herelleviridae	L3_Synergistia	0.044	0.038	2.44.E-01	8.88.E-01
Microviridae	L3_Synergistia	-0.004	0.038	9.15.E-01	9.98.E-01
Myoviridae	L3_Synergistia	0.017	0.042	6.89.E-01	9.80.E-01
Phycodnaviridae	L3_Synergistia	0.021	0.037	5.82.E-01	9.64.E-01
Podoviridae	L3_Synergistia	0.044	0.044	3.16.E-01	9.15.E-01
Siphoviridae	L3_Synergistia	0.016	0.039	6.90.E-01	9.80.E-01
Autographiviridae	L3_Spirochaetia	-0.021	0.040	5.88.E-01	9.67.E-01
crAss_like_phage	L3_Spirochaetia	-0.039	0.044	3.70.E-01	9.15.E-01
Herelleviridae	L3_Spirochaetia	0.022	0.041	5.90.E-01	9.68.E-01
Microviridae	L3_Spirochaetia	-0.004	0.041	9.13.E-01	9.98.E-01
Myoviridae	L3_Spirochaetia	0.038	0.046	4.16.E-01	9.18.E-01
Phycodnaviridae	L3_Spirochaetia	-0.014	0.041	7.39.E-01	9.88.E-01
Podoviridae	L3_Spirochaetia	0.045	0.048	3.45.E-01	9.15.E-01
Siphoviridae	L3_Spirochaetia	-0.037	0.042	3.79.E-01	9.15.E-01
Autographiviridae	L3_Verrucomicrobiae	-0.009	0.039	8.18.E-01	9.93.E-01
crAss_like_phage	L3_Verrucomicrobiae	0.013	0.043	7.69.E-01	9.91.E-01
Herelleviridae	L3_Verrucomicrobiae	0.024	0.040	5.51.E-01	9.60.E-01
Microviridae	L3_Verrucomicrobiae	-0.074	0.040	6.67.E-02	7.24.E-01
Myoviridae	L3_Verrucomicrobiae	0.041	0.045	3.64.E-01	9.15.E-01
Phycodnaviridae	L3_Verrucomicrobiae	0.054	0.040	1.77.E-01	8.58.E-01
Podoviridae	L3_Verrucomicrobiae	0.092	0.046	4.68.E-02	7.22.E-01
Siphoviridae	L3_Verrucomicrobiae	-0.004	0.041	9.28.E-01	9.98.E-01
Autographiviridae	L3_c_Firmicutes_bacterium	-0.024	0.024	3.27.E-01	9.15.E-01
crAss_like_phage	L3_c_Firmicutes_bacterium	0.032	0.027	2.29.E-01	8.82.E-01
Herelleviridae	L3_c_Firmicutes_bacterium	0.001	0.025	9.83.E-01	9.99.E-01
Microviridae	L3_c_Firmicutes_bacterium	0.023	0.025	3.74.E-01	9.15.E-01
Myoviridae	L3_c_Firmicutes_bacterium	-0.019	0.028	4.98.E-01	9.55.E-01
Phycodnaviridae	L3_c_Firmicutes_bacterium	0.030	0.025	2.31.E-01	8.82.E-01
Podoviridae	L3_c_Firmicutes_bacterium	0.008	0.029	7.78.E-01	9.92.E-01
Siphoviridae	L3_c_Firmicutes_bacterium	0.007	0.026	7.90.E-01	9.92.E-01
Autographiviridae	L3_c_bacterium_LF.3	0.004	0.039	9.22.E-01	9.98.E-01
crAss_like_phage	L3_c_bacterium_LF.3	-0.031	0.043	4.74.E-01	9.52.E-01
Herelleviridae	L3_c_bacterium_LF.3	0.009	0.040	8.27.E-01	9.93.E-01
Microviridae	L3_c_bacterium_LF.3	-0.006	0.040	8.84.E-01	9.96.E-01
Myoviridae	L3_c_bacterium_LF.3	0.000	0.045	9.97.E-01	9.99.E-01
Phycodnaviridae	L3_c_bacterium_LF.3	-0.025	0.040	5.26.E-01	9.57.E-01
Podoviridae	L3_c_bacterium_LF.3	-0.024	0.046	6.00.E-01	9.70.E-01
Siphoviridae	L3_c_bacterium_LF.3	-0.028	0.041	4.92.E-01	9.55.E-01
Autographiviridae	L3_Bacilli	0.035	0.023	1.34.E-01	8.06.E-01
crAss_like_phage	L3_Bacilli	0.000	0.026	9.88.E-01	9.99.E-01
Herelleviridae	L3_Bacilli	0.022	0.024	3.54.E-01	9.15.E-01
Microviridae	L3_Bacilli	0.003	0.024	9.05.E-01	9.97.E-01
Myoviridae	L3_Bacilli	0.016	0.027	5.47.E-01	9.60.E-01
Phycodnaviridae	L3_Bacilli	0.006	0.024	7.99.E-01	9.92.E-01
Podoviridae	L3_Bacilli	0.052	0.028	6.08.E-02	7.24.E-01
Siphoviridae	L3_Bacilli	0.043	0.025	8.28.E-02	7.49.E-01
Autographiviridae	L3_Alphaproteobacteria	0.044	0.039	2.56.E-01	8.95.E-01
crAss_like_phage	L3_Alphaproteobacteria	-0.111	0.042	9.26.E-03	4.94.E-01
Herelleviridae	L3_Alphaproteobacteria	-0.038	0.040	3.43.E-01	9.15.E-01
Microviridae	L3_Alphaproteobacteria	0.009	0.040	8.15.E-01	9.93.E-01
Myoviridae	L3_Alphaproteobacteria	-0.001	0.045	9.86.E-01	9.99.E-01
Phycodnaviridae	L3_Alphaproteobacteria	-0.064	0.040	1.06.E-01	7.82.E-01
Podoviridae	L3_Alphaproteobacteria	-0.029	0.046	5.27.E-01	9.57.E-01
Siphoviridae	L3_Alphaproteobacteria	-0.029	0.041	4.79.E-01	9.53.E-01
Autographiviridae	L3_Deltaproteobacteria	0.053	0.036	1.34.E-01	8.06.E-01

crAss_like_phage	L3_Deltaproteobacteria	-0.016	0.039	6.88.E-01	9.80.E-01
Herelleviridae	L3_Deltaproteobacteria	-0.028	0.037	4.48.E-01	9.35.E-01
Microviridae	L3_Deltaproteobacteria	0.013	0.037	7.35.E-01	9.88.E-01
Myoviridae	L3_Deltaproteobacteria	0.013	0.041	7.54.E-01	9.89.E-01
Phycodnaviridae	L3_Deltaproteobacteria	-0.011	0.037	7.55.E-01	9.89.E-01
Podoviridae	L3_Deltaproteobacteria	-0.024	0.043	5.74.E-01	9.62.E-01
Siphoviridae	L3_Deltaproteobacteria	-0.020	0.038	6.01.E-01	9.71.E-01
Autographiviridae	L3_Gammaproteobacteria	-0.001	0.024	9.69.E-01	9.98.E-01
crAss_like_phage	L3_Gammaproteobacteria	-0.024	0.027	3.74.E-01	9.15.E-01
Herelleviridae	L3_Gammaproteobacteria	-0.002	0.025	9.21.E-01	9.98.E-01
Microviridae	L3_Gammaproteobacteria	0.039	0.025	1.19.E-01	7.91.E-01
Myoviridae	L3_Gammaproteobacteria	0.028	0.028	3.17.E-01	9.15.E-01
Phycodnaviridae	L3_Gammaproteobacteria	0.027	0.025	2.75.E-01	9.05.E-01
Podoviridae	L3_Gammaproteobacteria	-0.020	0.029	4.98.E-01	9.55.E-01
Siphoviridae	L3_Gammaproteobacteria	0.023	0.026	3.76.E-01	9.15.E-01
Autographiviridae	L3_c_Bacteroidetes_bacterium	-0.045	0.040	2.69.E-01	9.01.E-01
crAss_like_phage	L3_c_Bacteroidetes_bacterium	-0.012	0.045	7.81.E-01	9.92.E-01
Herelleviridae	L3_c_Bacteroidetes_bacterium	0.018	0.042	6.59.E-01	9.79.E-01
Microviridae	L3_c_Bacteroidetes_bacterium	0.047	0.042	2.60.E-01	8.95.E-01
Myoviridae	L3_c_Bacteroidetes_bacterium	-0.032	0.047	4.99.E-01	9.55.E-01
Phycodnaviridae	L3_c_Bacteroidetes_bacterium	0.046	0.041	2.65.E-01	9.01.E-01
Podoviridae	L3_c_Bacteroidetes_bacterium	0.053	0.048	2.72.E-01	9.02.E-01
Siphoviridae	L3_c_Bacteroidetes_bacterium	0.036	0.043	4.08.E-01	9.17.E-01
Autographiviridae	L3_Tissierellia	0.056	0.038	1.42.E-01	8.12.E-01
crAss_like_phage	L3_Tissierellia	0.008	0.042	8.46.E-01	9.95.E-01
Herelleviridae	L3_Tissierellia	0.035	0.040	3.83.E-01	9.15.E-01
Microviridae	L3_Tissierellia	0.047	0.040	2.41.E-01	8.85.E-01
Myoviridae	L3_Tissierellia	-0.004	0.045	9.24.E-01	9.98.E-01
Phycodnaviridae	L3_Tissierellia	0.005	0.039	9.04.E-01	9.97.E-01
Podoviridae	L3_Tissierellia	0.007	0.046	8.71.E-01	9.96.E-01
Siphoviridae	L3_Tissierellia	0.001	0.041	9.82.E-01	9.99.E-01
Autographiviridae	L3_Erysipelotrichia	0.040	0.041	3.34.E-01	9.15.E-01
crAss_like_phage	L3_Erysipelotrichia	0.008	0.045	8.67.E-01	9.96.E-01
Herelleviridae	L3_Erysipelotrichia	0.001	0.043	9.88.E-01	9.99.E-01
Microviridae	L3_Erysipelotrichia	0.054	0.043	2.09.E-01	8.76.E-01
Myoviridae	L3_Erysipelotrichia	0.007	0.048	8.91.E-01	9.96.E-01
Phycodnaviridae	L3_Erysipelotrichia	-0.005	0.042	9.14.E-01	9.98.E-01
Podoviridae	L3_Erysipelotrichia	-0.096	0.049	5.26.E-02	7.22.E-01
Siphoviridae	L3_Erysipelotrichia	-0.028	0.044	5.31.E-01	9.57.E-01
Autographiviridae	L3_c_Proteobacteria_bacterium	-0.012	0.039	7.63.E-01	9.91.E-01
crAss_like_phage	L3_c_Proteobacteria_bacterium	0.030	0.043	4.86.E-01	9.54.E-01
Herelleviridae	L3_c_Proteobacteria_bacterium	0.046	0.040	2.55.E-01	8.95.E-01
Microviridae	L3_c_Proteobacteria_bacterium	-0.060	0.041	1.41.E-01	8.10.E-01
Myoviridae	L3_c_Proteobacteria_bacterium	0.012	0.045	7.83.E-01	9.92.E-01
Phycodnaviridae	L3_c_Proteobacteria_bacterium	0.008	0.040	8.51.E-01	9.95.E-01
Podoviridae	L3_c_Proteobacteria_bacterium	-0.126	0.046	6.84.E-03	4.51.E-01
Siphoviridae	L3_c_Proteobacteria_bacterium	-0.008	0.042	8.52.E-01	9.95.E-01
Autographiviridae	L3_Coriobacteriia	0.023	0.034	5.01.E-01	9.55.E-01
crAss_like_phage	L3_Coriobacteriia	-0.031	0.038	4.12.E-01	9.18.E-01
Herelleviridae	L3_Coriobacteriia	0.040	0.035	2.56.E-01	8.95.E-01
Microviridae	L3_Coriobacteriia	-0.040	0.035	2.54.E-01	8.95.E-01
Myoviridae	L3_Coriobacteriia	-0.081	0.039	4.04.E-02	7.04.E-01
Phycodnaviridae	L3_Coriobacteriia	0.047	0.035	1.75.E-01	8.56.E-01
Podoviridae	L3_Coriobacteriia	-0.046	0.041	2.58.E-01	8.95.E-01
Siphoviridae	L3_Coriobacteriia	-0.053	0.036	1.45.E-01	8.15.E-01
Autographiviridae	L3_Actinobacteria	-0.009	0.021	6.59.E-01	9.79.E-01
crAss_like_phage	L3_Actinobacteria	-0.026	0.023	2.64.E-01	9.01.E-01
Herelleviridae	L3_Actinobacteria	0.009	0.022	6.91.E-01	9.80.E-01
Microviridae	L3_Actinobacteria	0.044	0.022	4.60.E-02	7.22.E-01
Myoviridae	L3_Actinobacteria	0.006	0.025	8.11.E-01	9.93.E-01
Phycodnaviridae	L3_Actinobacteria	-0.004	0.022	8.65.E-01	9.96.E-01
Podoviridae	L3_Actinobacteria	0.009	0.025	7.33.E-01	9.88.E-01
Siphoviridae	L3_Actinobacteria	0.029	0.023	1.96.E-01	8.66.E-01
Autographiviridae	L3_Negativicutes	0.017	0.040	6.75.E-01	9.80.E-01
crAss_like_phage	L3_Negativicutes	-0.033	0.044	4.47.E-01	9.34.E-01

Herelleviridae	L3_Negativicutes	-0.072	0.041	7.95.E-02	7.43.E-01
Microviridae	L3_Negativicutes	-0.060	0.041	1.45.E-01	8.15.E-01
Myoviridae	L3_Negativicutes	0.077	0.046	9.21.E-02	7.59.E-01
Phycodnaviridae	L3_Negativicutes	-0.030	0.041	4.62.E-01	9.44.E-01
Podoviridae	L3_Negativicutes	0.015	0.047	7.59.E-01	9.91.E-01
Siphoviridae	L3_Negativicutes	-0.020	0.042	6.33.E-01	9.76.E-01
Autographiviridae	L3_Epsilonproteobacteria	-0.002	0.029	9.47.E-01	9.98.E-01
crAss_like_phage	L3_Epsilonproteobacteria	0.010	0.032	7.59.E-01	9.91.E-01
Herelleviridae	L3_Epsilonproteobacteria	-0.002	0.030	9.42.E-01	9.98.E-01
Microviridae	L3_Epsilonproteobacteria	-0.053	0.030	7.78.E-02	7.42.E-01
Myoviridae	L3_Epsilonproteobacteria	0.014	0.034	6.86.E-01	9.80.E-01
Phycodnaviridae	L3_Epsilonproteobacteria	0.000	0.030	9.88.E-01	9.99.E-01
Podoviridae	L3_Epsilonproteobacteria	-0.043	0.035	2.20.E-01	8.79.E-01
Siphoviridae	L3_Epsilonproteobacteria	-0.004	0.031	8.96.E-01	9.97.E-01
Autographiviridae	L3_Clostridia	0.018	0.019	3.59.E-01	9.15.E-01
crAss_like_phage	L3_Clostridia	0.014	0.021	5.22.E-01	9.57.E-01
Herelleviridae	L3_Clostridia	0.017	0.020	3.96.E-01	9.15.E-01
Microviridae	L3_Clostridia	-0.005	0.020	8.15.E-01	9.93.E-01
Myoviridae	L3_Clostridia	0.004	0.022	8.49.E-01	9.95.E-01
Phycodnaviridae	L3_Clostridia	0.034	0.020	8.29.E-02	7.49.E-01
Podoviridae	L3_Clostridia	-0.006	0.023	8.11.E-01	9.93.E-01
Siphoviridae	L3_Clostridia	-0.022	0.021	2.92.E-01	9.15.E-01
Autographiviridae	L3_Fusobacteriia	-0.039	0.037	2.97.E-01	9.15.E-01
crAss_like_phage	L3_Fusobacteriia	-0.091	0.041	2.80.E-02	6.58.E-01
Herelleviridae	L3_Fusobacteriia	0.040	0.039	3.08.E-01	9.15.E-01
Microviridae	L3_Fusobacteriia	0.001	0.039	9.71.E-01	9.99.E-01
Myoviridae	L3_Fusobacteriia	0.069	0.043	1.16.E-01	7.85.E-01
Phycodnaviridae	L3_Fusobacteriia	0.048	0.038	2.14.E-01	8.78.E-01
Podoviridae	L3_Fusobacteriia	0.110	0.045	1.42.E-02	5.51.E-01
Siphoviridae	L3_Fusobacteriia	-0.022	0.040	5.76.E-01	9.62.E-01
Autographiviridae	L4_Pasteurellales	-0.003	0.032	9.37.E-01	9.98.E-01
crAss_like_phage	L4_Pasteurellales	-0.051	0.035	1.47.E-01	8.16.E-01
Herelleviridae	L4_Pasteurellales	0.060	0.033	7.06.E-02	7.24.E-01
Microviridae	L4_Pasteurellales	0.016	0.033	6.31.E-01	9.76.E-01
Myoviridae	L4_Pasteurellales	0.102	0.037	5.34.E-03	4.28.E-01
Phycodnaviridae	L4_Pasteurellales	0.032	0.033	3.29.E-01	9.15.E-01
Podoviridae	L4_Pasteurellales	0.007	0.038	8.50.E-01	9.95.E-01
Siphoviridae	L4_Pasteurellales	0.001	0.034	9.86.E-01	9.99.E-01
Autographiviridae	L4_Coriobacteriales	0.023	0.033	4.82.E-01	9.53.E-01
crAss_like_phage	L4_Coriobacteriales	-0.037	0.036	3.10.E-01	9.15.E-01
Herelleviridae	L4_Coriobacteriales	0.017	0.034	6.18.E-01	9.75.E-01
Microviridae	L4_Coriobacteriales	-0.032	0.034	3.45.E-01	9.15.E-01
Myoviridae	L4_Coriobacteriales	-0.038	0.038	3.22.E-01	9.15.E-01
Phycodnaviridae	L4_Coriobacteriales	0.031	0.034	3.53.E-01	9.15.E-01
Podoviridae	L4_Coriobacteriales	-0.051	0.039	1.97.E-01	8.66.E-01
Siphoviridae	L4_Coriobacteriales	-0.048	0.035	1.74.E-01	8.56.E-01
Autographiviridae	L4_Selenomonadales	-0.036	0.037	3.32.E-01	9.15.E-01
crAss_like_phage	L4_Selenomonadales	-0.018	0.041	6.59.E-01	9.79.E-01
Herelleviridae	L4_Selenomonadales	-0.034	0.038	3.78.E-01	9.15.E-01
Microviridae	L4_Selenomonadales	-0.026	0.039	4.93.E-01	9.55.E-01
Myoviridae	L4_Selenomonadales	0.066	0.043	1.25.E-01	7.94.E-01
Phycodnaviridae	L4_Selenomonadales	-0.030	0.038	4.23.E-01	9.23.E-01
Podoviridae	L4_Selenomonadales	-0.058	0.044	1.93.E-01	8.62.E-01
Siphoviridae	L4_Selenomonadales	-0.025	0.040	5.31.E-01	9.57.E-01
Autographiviridae	L4_o_Bacteroidetes_bacterium	-0.045	0.040	2.69.E-01	9.01.E-01
crAss_like_phage	L4_o_Bacteroidetes_bacterium	-0.012	0.045	7.81.E-01	9.92.E-01
Herelleviridae	L4_o_Bacteroidetes_bacterium	0.018	0.042	6.59.E-01	9.79.E-01
Microviridae	L4_o_Bacteroidetes_bacterium	0.047	0.042	2.60.E-01	8.95.E-01
Myoviridae	L4_o_Bacteroidetes_bacterium	-0.032	0.047	4.99.E-01	9.55.E-01
Phycodnaviridae	L4_o_Bacteroidetes_bacterium	0.046	0.041	2.65.E-01	9.01.E-01
Podoviridae	L4_o_Bacteroidetes_bacterium	0.053	0.048	2.72.E-01	9.02.E-01
Siphoviridae	L4_o_Bacteroidetes_bacterium	0.036	0.043	4.08.E-01	9.17.E-01
Autographiviridae	L4_Tissierellales	0.053	0.038	1.60.E-01	8.38.E-01
crAss_like_phage	L4_Tissierellales	0.007	0.042	8.68.E-01	9.96.E-01
Herelleviridae	L4_Tissierellales	0.037	0.039	3.55.E-01	9.15.E-01

Microviridae	L4_Tissierellales	0.055	0.040	1.67.E-01	8.44.E-01
Myoviridae	L4_Tissierellales	0.000	0.044	9.92.E-01	9.99.E-01
Phycodnaviridae	L4_Tissierellales	0.015	0.039	7.07.E-01	9.83.E-01
Podoviridae	L4_Tissierellales	0.007	0.046	8.75.E-01	9.96.E-01
Siphoviridae	L4_Tissierellales	0.002	0.041	9.60.E-01	9.98.E-01
Autographiviridae	L4_o_Firmicutes_bacterium	-0.024	0.024	3.27.E-01	9.15.E-01
crAss_like_phage	L4_o_Firmicutes_bacterium	0.032	0.027	2.29.E-01	8.82.E-01
Herelleviridae	L4_o_Firmicutes_bacterium	0.001	0.025	9.83.E-01	9.99.E-01
Microviridae	L4_o_Firmicutes_bacterium	0.023	0.025	3.74.E-01	9.15.E-01
Myoviridae	L4_o_Firmicutes_bacterium	-0.019	0.028	4.98.E-01	9.55.E-01
Phycodnaviridae	L4_o_Firmicutes_bacterium	0.030	0.025	2.31.E-01	8.82.E-01
Podoviridae	L4_o_Firmicutes_bacterium	0.008	0.029	7.78.E-01	9.92.E-01
Siphoviridae	L4_o_Firmicutes_bacterium	0.007	0.026	7.90.E-01	9.92.E-01
Autographiviridae	L4_Eggerthellales	0.005	0.034	8.77.E-01	9.96.E-01
crAss_like_phage	L4_Eggerthellales	-0.027	0.038	4.78.E-01	9.52.E-01
Herelleviridae	L4_Eggerthellales	0.058	0.036	1.04.E-01	7.82.E-01
Microviridae	L4_Eggerthellales	-0.031	0.036	3.83.E-01	9.15.E-01
Myoviridae	L4_Eggerthellales	-0.047	0.040	2.38.E-01	8.85.E-01
Phycodnaviridae	L4_Eggerthellales	0.006	0.035	8.64.E-01	9.96.E-01
Podoviridae	L4_Eggerthellales	0.057	0.041	1.65.E-01	8.43.E-01
Siphoviridae	L4_Eggerthellales	-0.067	0.037	6.82.E-02	7.24.E-01
Autographiviridae	L4_Desulfovibrionales	0.054	0.036	1.32.E-01	8.03.E-01
crAss_like_phage	L4_Desulfovibrionales	-0.007	0.040	8.57.E-01	9.96.E-01
Herelleviridae	L4_Desulfovibrionales	-0.033	0.037	3.80.E-01	9.15.E-01
Microviridae	L4_Desulfovibrionales	0.015	0.037	6.90.E-01	9.80.E-01
Myoviridae	L4_Desulfovibrionales	0.018	0.042	6.70.E-01	9.80.E-01
Phycodnaviridae	L4_Desulfovibrionales	-0.016	0.037	6.59.E-01	9.79.E-01
Podoviridae	L4_Desulfovibrionales	-0.019	0.043	6.61.E-01	9.79.E-01
Siphoviridae	L4_Desulfovibrionales	-0.022	0.038	5.60.E-01	9.61.E-01
Autographiviridae	L4_Acidaminococcales	0.040	0.039	2.99.E-01	9.15.E-01
crAss_like_phage	L4_Acidaminococcales	0.019	0.043	6.62.E-01	9.79.E-01
Herelleviridae	L4_Acidaminococcales	0.026	0.040	5.24.E-01	9.57.E-01
Microviridae	L4_Acidaminococcales	0.019	0.040	6.35.E-01	9.76.E-01
Myoviridae	L4_Acidaminococcales	0.163	0.044	2.70.E-04	1.92.E-01
Phycodnaviridae	L4_Acidaminococcales	0.064	0.040	1.11.E-01	7.82.E-01
Podoviridae	L4_Acidaminococcales	0.085	0.046	6.86.E-02	7.24.E-01
Siphoviridae	L4_Acidaminococcales	0.057	0.041	1.68.E-01	8.44.E-01
Autographiviridae	L4_Fusobacteriales	-0.039	0.037	2.97.E-01	9.15.E-01
crAss_like_phage	L4_Fusobacteriales	-0.091	0.041	2.80.E-02	6.58.E-01
Herelleviridae	L4_Fusobacteriales	0.040	0.039	3.08.E-01	9.15.E-01
Microviridae	L4_Fusobacteriales	0.001	0.039	9.71.E-01	9.99.E-01
Myoviridae	L4_Fusobacteriales	0.069	0.043	1.16.E-01	7.85.E-01
Phycodnaviridae	L4_Fusobacteriales	0.048	0.038	2.14.E-01	8.78.E-01
Podoviridae	L4_Fusobacteriales	0.110	0.045	1.42.E-02	5.51.E-01
Siphoviridae	L4_Fusobacteriales	-0.022	0.040	5.76.E-01	9.62.E-01
Autographiviridae	L4_Verrucomicrobiales	-0.009	0.039	8.18.E-01	9.93.E-01
crAss_like_phage	L4_Verrucomicrobiales	0.013	0.043	7.69.E-01	9.91.E-01
Herelleviridae	L4_Verrucomicrobiales	0.024	0.040	5.51.E-01	9.60.E-01
Microviridae	L4_Verrucomicrobiales	-0.074	0.040	6.67.E-02	7.24.E-01
Myoviridae	L4_Verrucomicrobiales	0.041	0.045	3.64.E-01	9.15.E-01
Phycodnaviridae	L4_Verrucomicrobiales	0.054	0.040	1.77.E-01	8.58.E-01
Podoviridae	L4_Verrucomicrobiales	0.092	0.046	4.68.E-02	7.22.E-01
Siphoviridae	L4_Verrucomicrobiales	-0.004	0.041	9.28.E-01	9.98.E-01
Autographiviridae	L4_Corynebacteriales	-0.002	0.037	9.61.E-01	9.98.E-01
crAss_like_phage	L4_Corynebacteriales	0.080	0.040	4.97.E-02	7.22.E-01
Herelleviridae	L4_Corynebacteriales	-0.015	0.038	7.02.E-01	9.81.E-01
Microviridae	L4_Corynebacteriales	0.071	0.038	6.41.E-02	7.24.E-01
Myoviridae	L4_Corynebacteriales	0.074	0.043	8.21.E-02	7.48.E-01
Phycodnaviridae	L4_Corynebacteriales	-0.039	0.038	3.06.E-01	9.15.E-01
Podoviridae	L4_Corynebacteriales	0.071	0.044	1.07.E-01	7.82.E-01
Siphoviridae	L4_Corynebacteriales	0.011	0.039	7.85.E-01	9.92.E-01
Autographiviridae	L4_Clostridiales	0.018	0.019	3.59.E-01	9.15.E-01
crAss_like_phage	L4_Clostridiales	0.014	0.021	5.22.E-01	9.57.E-01
Herelleviridae	L4_Clostridiales	0.017	0.020	3.96.E-01	9.15.E-01
Microviridae	L4_Clostridiales	-0.005	0.020	8.15.E-01	9.93.E-01

Myoviridae	L4_Clostridiales	0.004	0.022	8.49.E-01	9.95.E-01
Phycodnaviridae	L4_Clostridiales	0.034	0.020	8.29.E-02	7.49.E-01
Podoviridae	L4_Clostridiales	-0.006	0.023	8.11.E-01	9.93.E-01
Siphoviridae	L4_Clostridiales	-0.022	0.021	2.92.E-01	9.15.E-01
Autographiviridae	L4_Aeromonadales	-0.013	0.040	7.48.E-01	9.89.E-01
crAss_like_phage	L4_Aeromonadales	0.025	0.044	5.74.E-01	9.62.E-01
Herelleviridae	L4_Aeromonadales	-0.073	0.041	7.41.E-02	7.28.E-01
Microviridae	L4_Aeromonadales	0.033	0.041	4.31.E-01	9.25.E-01
Myoviridae	L4_Aeromonadales	0.007	0.046	8.78.E-01	9.96.E-01
Phycodnaviridae	L4_Aeromonadales	0.017	0.041	6.78.E-01	9.80.E-01
Podoviridae	L4_Aeromonadales	-0.082	0.047	8.51.E-02	7.50.E-01
Siphoviridae	L4_Aeromonadales	0.022	0.042	6.06.E-01	9.73.E-01
Autographiviridae	L4_Campylobacterales	-0.002	0.029	9.49.E-01	9.98.E-01
crAss_like_phage	L4_Campylobacterales	0.010	0.032	7.58.E-01	9.90.E-01
Herelleviridae	L4_Campylobacterales	-0.002	0.030	9.39.E-01	9.98.E-01
Microviridae	L4_Campylobacterales	-0.053	0.030	7.80.E-02	7.42.E-01
Myoviridae	L4_Campylobacterales	0.014	0.034	6.83.E-01	9.80.E-01
Phycodnaviridae	L4_Campylobacterales	0.000	0.030	9.90.E-01	9.99.E-01
Podoviridae	L4_Campylobacterales	-0.043	0.035	2.20.E-01	8.79.E-01
Siphoviridae	L4_Campylobacterales	-0.004	0.031	8.97.E-01	9.97.E-01
Autographiviridae	L4_Synergistales	0.017	0.036	6.48.E-01	9.79.E-01
crAss_like_phage	L4_Synergistales	0.023	0.040	5.70.E-01	9.61.E-01
Herelleviridae	L4_Synergistales	0.044	0.038	2.44.E-01	8.88.E-01
Microviridae	L4_Synergistales	-0.004	0.038	9.15.E-01	9.98.E-01
Myoviridae	L4_Synergistales	0.017	0.042	6.89.E-01	9.80.E-01
Phycodnaviridae	L4_Synergistales	0.021	0.037	5.82.E-01	9.64.E-01
Podoviridae	L4_Synergistales	0.044	0.044	3.16.E-01	9.15.E-01
Siphoviridae	L4_Synergistales	0.016	0.039	6.90.E-01	9.80.E-01
Autographiviridae	L4_Burkholderiales	0.053	0.034	1.20.E-01	7.91.E-01
crAss_like_phage	L4_Burkholderiales	0.036	0.038	3.41.E-01	9.15.E-01
Herelleviridae	L4_Burkholderiales	-0.047	0.036	1.89.E-01	8.61.E-01
Microviridae	L4_Burkholderiales	-0.002	0.036	9.45.E-01	9.98.E-01
Myoviridae	L4_Burkholderiales	0.021	0.040	5.90.E-01	9.68.E-01
Phycodnaviridae	L4_Burkholderiales	-0.004	0.035	9.15.E-01	9.98.E-01
Podoviridae	L4_Burkholderiales	-0.048	0.041	2.40.E-01	8.85.E-01
Siphoviridae	L4_Burkholderiales	-0.019	0.037	6.03.E-01	9.72.E-01
Autographiviridae	L4_Bifidobacteriales	-0.006	0.023	7.76.E-01	9.92.E-01
crAss_like_phage	L4_Bifidobacteriales	-0.027	0.025	2.74.E-01	9.04.E-01
Herelleviridae	L4_Bifidobacteriales	0.013	0.023	5.73.E-01	9.61.E-01
Microviridae	L4_Bifidobacteriales	0.055	0.023	1.80.E-02	5.86.E-01
Myoviridae	L4_Bifidobacteriales	0.027	0.026	3.06.E-01	9.15.E-01
Phycodnaviridae	L4_Bifidobacteriales	0.003	0.023	8.87.E-01	9.96.E-01
Podoviridae	L4_Bifidobacteriales	0.024	0.027	3.82.E-01	9.15.E-01
Siphoviridae	L4_Bifidobacteriales	0.047	0.024	4.89.E-02	7.22.E-01
Autographiviridae	L4_Rhodospirillales	0.035	0.038	3.58.E-01	9.15.E-01
crAss_like_phage	L4_Rhodospirillales	-0.113	0.042	7.38.E-03	4.64.E-01
Herelleviridae	L4_Rhodospirillales	-0.059	0.040	1.40.E-01	8.10.E-01
Microviridae	L4_Rhodospirillales	0.020	0.040	6.16.E-01	9.75.E-01
Myoviridae	L4_Rhodospirillales	-0.043	0.045	3.40.E-01	9.15.E-01
Phycodnaviridae	L4_Rhodospirillales	-0.052	0.039	1.84.E-01	8.60.E-01
Podoviridae	L4_Rhodospirillales	-0.032	0.046	4.94.E-01	9.55.E-01
Siphoviridae	L4_Rhodospirillales	-0.021	0.041	6.15.E-01	9.75.E-01
Autographiviridae	L4_Lactobacillales	0.023	0.023	3.06.E-01	9.15.E-01
crAss_like_phage	L4_Lactobacillales	-0.008	0.025	7.50.E-01	9.89.E-01
Herelleviridae	L4_Lactobacillales	0.032	0.024	1.75.E-01	8.56.E-01
Microviridae	L4_Lactobacillales	0.006	0.024	8.09.E-01	9.93.E-01
Myoviridae	L4_Lactobacillales	0.007	0.026	7.87.E-01	9.92.E-01
Phycodnaviridae	L4_Lactobacillales	-0.005	0.023	8.44.E-01	9.95.E-01
Podoviridae	L4_Lactobacillales	0.047	0.027	8.47.E-02	7.50.E-01
Siphoviridae	L4_Lactobacillales	0.033	0.024	1.77.E-01	8.58.E-01
Autographiviridae	L4_Erysipelotrichales	0.040	0.041	3.34.E-01	9.15.E-01
crAss_like_phage	L4_Erysipelotrichales	0.008	0.045	8.67.E-01	9.96.E-01
Herelleviridae	L4_Erysipelotrichales	0.001	0.043	9.88.E-01	9.99.E-01
Microviridae	L4_Erysipelotrichales	0.054	0.043	2.09.E-01	8.76.E-01
Myoviridae	L4_Erysipelotrichales	0.007	0.048	8.91.E-01	9.96.E-01

Phycodnaviridae	L4_Erysipelotrichales	-0.005	0.042	9.14.E-01	9.98.E-01
Podoviridae	L4_Erysipelotrichales	-0.096	0.049	5.26.E-02	7.22.E-01
Siphoviridae	L4_Erysipelotrichales	-0.028	0.044	5.31.E-01	9.57.E-01
Autographiviridae	L4_Propionibacteriales	-0.012	0.040	7.54.E-01	9.89.E-01
crAss_like_phage	L4_Propionibacteriales	-0.082	0.044	6.06.E-02	7.24.E-01
Herelleviridae	L4_Propionibacteriales	0.041	0.041	3.14.E-01	9.15.E-01
Microviridae	L4_Propionibacteriales	-0.004	0.041	9.31.E-01	9.98.E-01
Myoviridae	L4_Propionibacteriales	0.036	0.046	4.40.E-01	9.30.E-01
Phycodnaviridae	L4_Propionibacteriales	0.049	0.041	2.25.E-01	8.82.E-01
Podoviridae	L4_Propionibacteriales	-0.005	0.047	9.09.E-01	9.98.E-01
Siphoviridae	L4_Propionibacteriales	0.002	0.042	9.53.E-01	9.98.E-01
Autographiviridae	L4_Bacteroidales	-0.013	0.015	3.86.E-01	9.15.E-01
crAss_like_phage	L4_Bacteroidales	-0.026	0.017	1.18.E-01	7.86.E-01
Herelleviridae	L4_Bacteroidales	-0.043	0.015	5.31.E-03	4.28.E-01
Microviridae	L4_Bacteroidales	-0.018	0.016	2.39.E-01	8.85.E-01
Myoviridae	L4_Bacteroidales	-0.025	0.017	1.49.E-01	8.22.E-01
Phycodnaviridae	L4_Bacteroidales	-0.043	0.015	5.35.E-03	4.28.E-01
Podoviridae	L4_Bacteroidales	-0.007	0.018	6.98.E-01	9.81.E-01
Siphoviridae	L4_Bacteroidales	-0.042	0.016	9.43.E-03	4.94.E-01
Autographiviridae	L4_Bacillales	0.000	0.044	9.98.E-01	9.99.E-01
crAss_like_phage	L4_Bacillales	0.073	0.048	1.29.E-01	7.99.E-01
Herelleviridae	L4_Bacillales	-0.029	0.045	5.20.E-01	9.57.E-01
Microviridae	L4_Bacillales	0.056	0.045	2.18.E-01	8.79.E-01
Myoviridae	L4_Bacillales	0.088	0.050	8.17.E-02	7.47.E-01
Phycodnaviridae	L4_Bacillales	0.043	0.045	3.37.E-01	9.15.E-01
Podoviridae	L4_Bacillales	0.054	0.052	2.98.E-01	9.15.E-01
Siphoviridae	L4_Bacillales	0.062	0.046	1.80.E-01	8.60.E-01
Autographiviridae	L4_Enterobacterales	-0.002	0.019	9.22.E-01	9.98.E-01
crAss_like_phage	L4_Enterobacterales	0.026	0.021	2.15.E-01	8.79.E-01
Herelleviridae	L4_Enterobacterales	-0.022	0.020	2.71.E-01	9.02.E-01
Microviridae	L4_Enterobacterales	0.015	0.020	4.42.E-01	9.32.E-01
Myoviridae	L4_Enterobacterales	-0.006	0.022	8.04.E-01	9.93.E-01
Phycodnaviridae	L4_Enterobacterales	0.005	0.020	7.99.E-01	9.92.E-01
Podoviridae	L4_Enterobacterales	-0.036	0.023	1.15.E-01	7.85.E-01
Siphoviridae	L4_Enterobacterales	0.013	0.021	5.25.E-01	9.57.E-01
Autographiviridae	L4_o_bacterium_LF.3	0.004	0.039	9.22.E-01	9.98.E-01
crAss_like_phage	L4_o_bacterium_LF.3	-0.031	0.043	4.74.E-01	9.52.E-01
Herelleviridae	L4_o_bacterium_LF.3	0.009	0.040	8.27.E-01	9.93.E-01
Microviridae	L4_o_bacterium_LF.3	-0.006	0.040	8.84.E-01	9.96.E-01
Myoviridae	L4_o_bacterium_LF.3	0.000	0.045	9.97.E-01	9.99.E-01
Phycodnaviridae	L4_o_bacterium_LF.3	-0.025	0.040	5.26.E-01	9.57.E-01
Podoviridae	L4_o_bacterium_LF.3	-0.024	0.046	6.00.E-01	9.70.E-01
Siphoviridae	L4_o_bacterium_LF.3	-0.028	0.041	4.92.E-01	9.55.E-01
Autographiviridae	L4_Brachyspirales	-0.032	0.039	4.17.E-01	9.19.E-01
crAss_like_phage	L4_Brachyspirales	-0.034	0.043	4.30.E-01	9.25.E-01
Herelleviridae	L4_Brachyspirales	-0.005	0.041	8.95.E-01	9.97.E-01
Microviridae	L4_Brachyspirales	0.054	0.041	1.87.E-01	8.61.E-01
Myoviridae	L4_Brachyspirales	-0.021	0.045	6.47.E-01	9.79.E-01
Phycodnaviridae	L4_Brachyspirales	0.021	0.040	6.02.E-01	9.71.E-01
Podoviridae	L4_Brachyspirales	-0.060	0.047	1.97.E-01	8.66.E-01
Siphoviridae	L4_Brachyspirales	-0.018	0.042	6.68.E-01	9.80.E-01
Autographiviridae	L4_Veillonellales	0.048	0.040	2.32.E-01	8.82.E-01
crAss_like_phage	L4_Veillonellales	-0.003	0.045	9.38.E-01	9.98.E-01
Herelleviridae	L4_Veillonellales	-0.099	0.042	1.76.E-02	5.86.E-01
Microviridae	L4_Veillonellales	-0.071	0.042	8.99.E-02	7.58.E-01
Myoviridae	L4_Veillonellales	-0.070	0.047	1.36.E-01	8.08.E-01
Phycodnaviridae	L4_Veillonellales	-0.098	0.041	1.82.E-02	5.86.E-01
Podoviridae	L4_Veillonellales	-0.085	0.048	7.86.E-02	7.43.E-01
Siphoviridae	L4_Veillonellales	-0.054	0.043	2.08.E-01	8.76.E-01
Autographiviridae	L4_Thermales	0.000	0.008	9.51.E-01	9.98.E-01
crAss_like_phage	L4_Thermales	-0.014	0.009	1.05.E-01	7.82.E-01
Herelleviridae	L4_Thermales	-0.003	0.008	7.17.E-01	9.86.E-01
Microviridae	L4_Thermales	0.008	0.008	3.32.E-01	9.15.E-01
Myoviridae	L4_Thermales	-0.007	0.009	4.55.E-01	9.39.E-01
Phycodnaviridae	L4_Thermales	-0.005	0.008	5.73.E-01	9.61.E-01

Podoviridae	L4_Thermales	-0.010	0.009	3.04.E-01	9.15.E-01
Siphoviridae	L4_Thermales	-0.011	0.008	1.84.E-01	8.60.E-01
Autographiviridae	L4_Micrococcales	0.047	0.028	9.74.E-02	7.74.E-01
crAss_like_phage	L4_Micrococcales	0.023	0.031	4.68.E-01	9.50.E-01
Herelleviridae	L4_Micrococcales	0.011	0.030	7.23.E-01	9.86.E-01
Microviridae	L4_Micrococcales	0.013	0.030	6.59.E-01	9.79.E-01
Myoviridae	L4_Micrococcales	0.103	0.033	1.73.E-03	3.25.E-01
Phycodnaviridae	L4_Micrococcales	0.003	0.029	9.10.E-01	9.98.E-01
Podoviridae	L4_Micrococcales	0.060	0.034	8.12.E-02	7.47.E-01
Siphoviridae	L4_Micrococcales	0.015	0.030	6.34.E-01	9.76.E-01
Autographiviridae	L4_Flavobacteriales	0.026	0.020	2.03.E-01	8.75.E-01
crAss_like_phage	L4_Flavobacteriales	-0.005	0.023	8.14.E-01	9.93.E-01
Herelleviridae	L4_Flavobacteriales	-0.026	0.021	2.15.E-01	8.79.E-01
Microviridae	L4_Flavobacteriales	0.009	0.021	6.60.E-01	9.79.E-01
Myoviridae	L4_Flavobacteriales	-0.026	0.024	2.72.E-01	9.02.E-01
Phycodnaviridae	L4_Flavobacteriales	0.009	0.021	6.66.E-01	9.80.E-01
Podoviridae	L4_Flavobacteriales	0.018	0.025	4.59.E-01	9.43.E-01
Siphoviridae	L4_Flavobacteriales	-0.039	0.022	7.21.E-02	7.25.E-01
Autographiviridae	L4_Methanobacteriales	0.048	0.036	1.84.E-01	8.60.E-01
crAss_like_phage	L4_Methanobacteriales	-0.028	0.040	4.82.E-01	9.53.E-01
Herelleviridae	L4_Methanobacteriales	0.067	0.037	7.32.E-02	7.26.E-01
Microviridae	L4_Methanobacteriales	0.049	0.037	1.90.E-01	8.61.E-01
Myoviridae	L4_Methanobacteriales	0.009	0.042	8.35.E-01	9.94.E-01
Phycodnaviridae	L4_Methanobacteriales	-0.022	0.037	5.53.E-01	9.60.E-01
Podoviridae	L4_Methanobacteriales	-0.017	0.043	7.02.E-01	9.81.E-01
Siphoviridae	L4_Methanobacteriales	-0.017	0.038	6.63.E-01	9.79.E-01
Autographiviridae	L4_Pseudomonadales	-0.063	0.043	1.41.E-01	8.10.E-01
crAss_like_phage	L4_Pseudomonadales	-0.102	0.047	3.03.E-02	6.66.E-01
Herelleviridae	L4_Pseudomonadales	-0.031	0.044	4.89.E-01	9.55.E-01
Microviridae	L4_Pseudomonadales	0.033	0.044	4.61.E-01	9.43.E-01
Myoviridae	L4_Pseudomonadales	0.033	0.049	5.11.E-01	9.57.E-01
Phycodnaviridae	L4_Pseudomonadales	0.026	0.044	5.52.E-01	9.60.E-01
Podoviridae	L4_Pseudomonadales	0.016	0.051	7.55.E-01	9.89.E-01
Siphoviridae	L4_Pseudomonadales	0.027	0.046	5.51.E-01	9.60.E-01
Autographiviridae	L4_Actinomycetales	-0.054	0.027	5.10.E-02	7.22.E-01
crAss_like_phage	L4_Actinomycetales	-0.012	0.030	6.91.E-01	9.80.E-01
Herelleviridae	L4_Actinomycetales	-0.065	0.028	2.16.E-02	6.09.E-01
Microviridae	L4_Actinomycetales	0.008	0.029	7.74.E-01	9.92.E-01
Myoviridae	L4_Actinomycetales	-0.035	0.032	2.70.E-01	9.02.E-01
Phycodnaviridae	L4_Actinomycetales	-0.024	0.028	3.87.E-01	9.15.E-01
Podoviridae	L4_Actinomycetales	-0.013	0.033	7.04.E-01	9.81.E-01
Siphoviridae	L4_Actinomycetales	-0.005	0.029	8.72.E-01	9.96.E-01
Autographiviridae	L4_o_Proteobacteria_bacterium	-0.012	0.039	7.63.E-01	9.91.E-01
crAss_like_phage	L4_o_Proteobacteria_bacterium	0.030	0.043	4.86.E-01	9.54.E-01
Herelleviridae	L4_o_Proteobacteria_bacterium	0.046	0.040	2.55.E-01	8.95.E-01
Microviridae	L4_o_Proteobacteria_bacterium	-0.060	0.041	1.41.E-01	8.10.E-01
Myoviridae	L4_o_Proteobacteria_bacterium	0.012	0.045	7.83.E-01	9.92.E-01
Phycodnaviridae	L4_o_Proteobacteria_bacterium	0.008	0.040	8.51.E-01	9.95.E-01
Podoviridae	L4_o_Proteobacteria_bacterium	-0.126	0.046	6.84.E-03	4.51.E-01
Siphoviridae	L4_o_Proteobacteria_bacterium	-0.008	0.042	8.52.E-01	9.95.E-01
Autographiviridae	L5_Peptoniphilaceae	0.052	0.038	1.75.E-01	8.56.E-01
crAss_like_phage	L5_Peptoniphilaceae	0.007	0.042	8.61.E-01	9.96.E-01
Herelleviridae	L5_Peptoniphilaceae	0.036	0.040	3.71.E-01	9.15.E-01
Microviridae	L5_Peptoniphilaceae	0.051	0.040	1.96.E-01	8.66.E-01
Myoviridae	L5_Peptoniphilaceae	-0.001	0.044	9.80.E-01	9.99.E-01
Phycodnaviridae	L5_Peptoniphilaceae	0.014	0.039	7.16.E-01	9.86.E-01
Podoviridae	L5_Peptoniphilaceae	0.007	0.046	8.74.E-01	9.96.E-01
Siphoviridae	L5_Peptoniphilaceae	0.003	0.041	9.44.E-01	9.98.E-01
Autographiviridae	L5_Oscillospiraceae	0.005	0.028	8.54.E-01	9.96.E-01
crAss_like_phage	L5_Oscillospiraceae	-0.004	0.031	8.89.E-01	9.96.E-01
Herelleviridae	L5_Oscillospiraceae	-0.030	0.029	2.96.E-01	9.15.E-01
Microviridae	L5_Oscillospiraceae	-0.009	0.029	7.64.E-01	9.91.E-01
Myoviridae	L5_Oscillospiraceae	-0.080	0.032	1.21.E-02	5.36.E-01
Phycodnaviridae	L5_Oscillospiraceae	-0.002	0.028	9.54.E-01	9.98.E-01
Podoviridae	L5_Oscillospiraceae	0.006	0.033	8.59.E-01	9.96.E-01

Siphoviridae	L5_Oscillospiraceae	0.027	0.030	3.56.E-01	9.15.E-01
Autographiviridae	L5_Bacteroidaceae	0.006	0.016	7.03.E-01	9.81.E-01
crAss_like_phage	L5_Bacteroidaceae	-0.034	0.018	5.42.E-02	7.23.E-01
Herelleviridae	L5_Bacteroidaceae	-0.029	0.017	8.30.E-02	7.49.E-01
Microviridae	L5_Bacteroidaceae	-0.018	0.017	2.76.E-01	9.05.E-01
Myoviridae	L5_Bacteroidaceae	0.011	0.019	5.50.E-01	9.60.E-01
Phycodnaviridae	L5_Bacteroidaceae	-0.010	0.016	5.47.E-01	9.60.E-01
Podoviridae	L5_Bacteroidaceae	-0.026	0.019	1.76.E-01	8.58.E-01
Siphoviridae	L5_Bacteroidaceae	-0.025	0.017	1.37.E-01	8.09.E-01
Autographiviridae	L5_Synergistaceae	0.017	0.036	6.48.E-01	9.79.E-01
crAss_like_phage	L5_Synergistaceae	0.023	0.040	5.70.E-01	9.61.E-01
Herelleviridae	L5_Synergistaceae	0.044	0.038	2.44.E-01	8.88.E-01
Microviridae	L5_Synergistaceae	-0.004	0.038	9.15.E-01	9.98.E-01
Myoviridae	L5_Synergistaceae	0.017	0.042	6.89.E-01	9.80.E-01
Phycodnaviridae	L5_Synergistaceae	0.021	0.037	5.82.E-01	9.64.E-01
Podoviridae	L5_Synergistaceae	0.044	0.044	3.16.E-01	9.15.E-01
Siphoviridae	L5_Synergistaceae	0.016	0.039	6.90.E-01	9.80.E-01
Autographiviridae	L5_Enterobacteriaceae	0.000	0.019	9.88.E-01	9.99.E-01
crAss_like_phage	L5_Enterobacteriaceae	0.036	0.021	9.18.E-02	7.59.E-01
Herelleviridae	L5_Enterobacteriaceae	-0.019	0.020	3.34.E-01	9.15.E-01
Microviridae	L5_Enterobacteriaceae	0.008	0.020	6.98.E-01	9.81.E-01
Myoviridae	L5_Enterobacteriaceae	0.000	0.022	9.99.E-01	9.99.E-01
Phycodnaviridae	L5_Enterobacteriaceae	0.000	0.020	9.87.E-01	9.99.E-01
Podoviridae	L5_Enterobacteriaceae	-0.037	0.023	1.12.E-01	7.83.E-01
Siphoviridae	L5_Enterobacteriaceae	0.016	0.021	4.30.E-01	9.25.E-01
Autographiviridae	L5_Ruminococcaceae	0.009	0.028	7.56.E-01	9.89.E-01
crAss_like_phage	L5_Ruminococcaceae	0.059	0.030	5.17.E-02	7.22.E-01
Herelleviridae	L5_Ruminococcaceae	-0.018	0.029	5.31.E-01	9.57.E-01
Microviridae	L5_Ruminococcaceae	0.011	0.029	6.99.E-01	9.81.E-01
Myoviridae	L5_Ruminococcaceae	0.054	0.032	9.27.E-02	7.60.E-01
Phycodnaviridae	L5_Ruminococcaceae	0.012	0.028	6.84.E-01	9.80.E-01
Podoviridae	L5_Ruminococcaceae	0.051	0.033	1.27.E-01	7.99.E-01
Siphoviridae	L5_Ruminococcaceae	-0.007	0.030	8.00.E-01	9.92.E-01
Autographiviridae	L5_Selenomonadaceae	-0.036	0.037	3.28.E-01	9.15.E-01
crAss_like_phage	L5_Selenomonadaceae	-0.020	0.041	6.33.E-01	9.76.E-01
Herelleviridae	L5_Selenomonadaceae	-0.034	0.038	3.76.E-01	9.15.E-01
Microviridae	L5_Selenomonadaceae	-0.026	0.039	4.93.E-01	9.55.E-01
Myoviridae	L5_Selenomonadaceae	0.067	0.043	1.21.E-01	7.93.E-01
Phycodnaviridae	L5_Selenomonadaceae	-0.030	0.038	4.31.E-01	9.25.E-01
Podoviridae	L5_Selenomonadaceae	-0.058	0.044	1.90.E-01	8.61.E-01
Siphoviridae	L5_Selenomonadaceae	-0.025	0.040	5.33.E-01	9.58.E-01
Autographiviridae	L5_f_bacterium_LF.3	0.004	0.039	9.22.E-01	9.98.E-01
crAss_like_phage	L5_f_bacterium_LF.3	-0.031	0.043	4.74.E-01	9.52.E-01
Herelleviridae	L5_f_bacterium_LF.3	0.009	0.040	8.27.E-01	9.93.E-01
Microviridae	L5_f_bacterium_LF.3	-0.006	0.040	8.84.E-01	9.96.E-01
Myoviridae	L5_f_bacterium_LF.3	0.000	0.045	9.97.E-01	9.99.E-01
Phycodnaviridae	L5_f_bacterium_LF.3	-0.025	0.040	5.26.E-01	9.57.E-01
Podoviridae	L5_f_bacterium_LF.3	-0.024	0.046	6.00.E-01	9.70.E-01
Siphoviridae	L5_f_bacterium_LF.3	-0.028	0.041	4.92.E-01	9.55.E-01
Autographiviridae	L5_Propionibacteriaceae	-0.003	0.036	9.27.E-01	9.98.E-01
crAss_like_phage	L5_Propionibacteriaceae	-0.087	0.039	2.66.E-02	6.58.E-01
Herelleviridae	L5_Propionibacteriaceae	-0.029	0.037	4.39.E-01	9.30.E-01
Microviridae	L5_Propionibacteriaceae	-0.056	0.037	1.32.E-01	8.03.E-01
Myoviridae	L5_Propionibacteriaceae	0.015	0.041	7.11.E-01	9.85.E-01
Phycodnaviridae	L5_Propionibacteriaceae	0.024	0.037	5.07.E-01	9.57.E-01
Podoviridae	L5_Propionibacteriaceae	-0.042	0.043	3.30.E-01	9.15.E-01
Siphoviridae	L5_Propionibacteriaceae	-0.014	0.038	7.08.E-01	9.83.E-01
Autographiviridae	L5_Comamonadaceae	-0.090	0.024	2.38.E-04	1.92.E-01
crAss_like_phage	L5_Comamonadaceae	-0.018	0.027	5.11.E-01	9.57.E-01
Herelleviridae	L5_Comamonadaceae	-0.026	0.026	3.19.E-01	9.15.E-01
Microviridae	L5_Comamonadaceae	0.038	0.026	1.40.E-01	8.10.E-01
Myoviridae	L5_Comamonadaceae	0.069	0.029	1.58.E-02	5.77.E-01
Phycodnaviridae	L5_Comamonadaceae	-0.015	0.025	5.55.E-01	9.61.E-01
Podoviridae	L5_Comamonadaceae	0.005	0.030	8.68.E-01	9.96.E-01
Siphoviridae	L5_Comamonadaceae	-0.039	0.026	1.44.E-01	8.12.E-01

Autographiviridae	L5_Leuconostocaceae	-0.015	0.039	6.95.E-01	9.81.E-01
crAss_like_phage	L5_Leuconostocaceae	-0.005	0.043	9.13.E-01	9.98.E-01
Herelleviridae	L5_Leuconostocaceae	0.040	0.040	3.28.E-01	9.15.E-01
Microviridae	L5_Leuconostocaceae	0.011	0.041	7.83.E-01	9.92.E-01
Myoviridae	L5_Leuconostocaceae	-0.054	0.045	2.28.E-01	8.82.E-01
Phycodnaviridae	L5_Leuconostocaceae	0.023	0.040	5.72.E-01	9.61.E-01
Podoviridae	L5_Leuconostocaceae	-0.004	0.047	9.34.E-01	9.98.E-01
Siphoviridae	L5_Leuconostocaceae	0.028	0.042	4.94.E-01	9.55.E-01
Autographiviridae	L5_f_Proteobacteria_bacterium	-0.012	0.039	7.63.E-01	9.91.E-01
crAss_like_phage	L5_f_Proteobacteria_bacterium	0.030	0.043	4.86.E-01	9.54.E-01
Herelleviridae	L5_f_Proteobacteria_bacterium	0.046	0.040	2.55.E-01	8.95.E-01
Microviridae	L5_f_Proteobacteria_bacterium	-0.060	0.041	1.41.E-01	8.10.E-01
Myoviridae	L5_f_Proteobacteria_bacterium	0.012	0.045	7.83.E-01	9.92.E-01
Phycodnaviridae	L5_f_Proteobacteria_bacterium	0.008	0.040	8.51.E-01	9.95.E-01
Podoviridae	L5_f_Proteobacteria_bacterium	-0.126	0.046	6.84.E-03	4.51.E-01
Siphoviridae	L5_f_Proteobacteria_bacterium	-0.008	0.042	8.52.E-01	9.95.E-01
Autographiviridae	L5_f_Bacteroides_pectinophilus	-0.004	0.032	9.07.E-01	9.97.E-01
crAss_like_phage	L5_f_Bacteroides_pectinophilus	-0.050	0.036	1.59.E-01	8.38.E-01
Herelleviridae	L5_f_Bacteroides_pectinophilus	0.002	0.034	9.53.E-01	9.98.E-01
Microviridae	L5_f_Bacteroides_pectinophilus	-0.062	0.034	6.46.E-02	7.24.E-01
Myoviridae	L5_f_Bacteroides_pectinophilus	-0.030	0.038	4.29.E-01	9.25.E-01
Phycodnaviridae	L5_f_Bacteroides_pectinophilus	-0.017	0.033	6.05.E-01	9.73.E-01
Podoviridae	L5_f_Bacteroides_pectinophilus	-0.039	0.039	3.20.E-01	9.15.E-01
Siphoviridae	L5_f_Bacteroides_pectinophilus	-0.075	0.034	2.99.E-02	6.66.E-01
Autographiviridae	L5_Pasteurellaceae	-0.003	0.032	9.37.E-01	9.98.E-01
crAss_like_phage	L5_Pasteurellaceae	-0.051	0.035	1.47.E-01	8.16.E-01
Herelleviridae	L5_Pasteurellaceae	0.060	0.033	7.06.E-02	7.24.E-01
Microviridae	L5_Pasteurellaceae	0.016	0.033	6.31.E-01	9.76.E-01
Myoviridae	L5_Pasteurellaceae	0.102	0.037	5.34.E-03	4.28.E-01
Phycodnaviridae	L5_Pasteurellaceae	0.032	0.033	3.29.E-01	9.15.E-01
Podoviridae	L5_Pasteurellaceae	0.007	0.038	8.50.E-01	9.95.E-01
Siphoviridae	L5_Pasteurellaceae	0.001	0.034	9.86.E-01	9.99.E-01
Autographiviridae	L5_f_Gemella	-0.034	0.027	2.03.E-01	8.75.E-01
crAss_like_phage	L5_f_Gemella	0.046	0.030	1.23.E-01	7.94.E-01
Herelleviridae	L5_f_Gemella	-0.045	0.028	1.04.E-01	7.82.E-01
Microviridae	L5_f_Gemella	0.015	0.028	5.96.E-01	9.69.E-01
Myoviridae	L5_f_Gemella	-0.009	0.031	7.69.E-01	9.91.E-01
Phycodnaviridae	L5_f_Gemella	-0.023	0.028	4.10.E-01	9.17.E-01
Podoviridae	L5_f_Gemella	0.033	0.032	3.14.E-01	9.15.E-01
Siphoviridae	L5_f_Gemella	0.041	0.029	1.50.E-01	8.22.E-01
Autographiviridae	L5_Veillonellaceae	0.048	0.040	2.32.E-01	8.82.E-01
crAss_like_phage	L5_Veillonellaceae	-0.003	0.045	9.38.E-01	9.98.E-01
Herelleviridae	L5_Veillonellaceae	-0.099	0.042	1.76.E-02	5.86.E-01
Microviridae	L5_Veillonellaceae	-0.071	0.042	8.99.E-02	7.58.E-01
Myoviridae	L5_Veillonellaceae	-0.070	0.047	1.36.E-01	8.08.E-01
Phycodnaviridae	L5_Veillonellaceae	-0.098	0.041	1.82.E-02	5.86.E-01
Podoviridae	L5_Veillonellaceae	-0.085	0.048	7.86.E-02	7.43.E-01
Siphoviridae	L5_Veillonellaceae	-0.054	0.043	2.08.E-01	8.76.E-01
Autographiviridae	L5_Morganellaceae	-0.012	0.031	6.92.E-01	9.81.E-01
crAss_like_phage	L5_Morganellaceae	-0.012	0.034	7.26.E-01	9.87.E-01
Herelleviridae	L5_Morganellaceae	0.012	0.032	7.07.E-01	9.83.E-01
Microviridae	L5_Morganellaceae	0.073	0.032	2.25.E-02	6.19.E-01
Myoviridae	L5_Morganellaceae	0.030	0.036	3.98.E-01	9.15.E-01
Phycodnaviridae	L5_Morganellaceae	0.028	0.031	3.80.E-01	9.15.E-01
Podoviridae	L5_Morganellaceae	0.035	0.037	3.42.E-01	9.15.E-01
Siphoviridae	L5_Morganellaceae	-0.037	0.033	2.65.E-01	9.01.E-01
Autographiviridae	L5_Desulfovibrionaceae	0.056	0.036	1.17.E-01	7.86.E-01
crAss_like_phage	L5_Desulfovibrionaceae	-0.009	0.040	8.25.E-01	9.93.E-01
Herelleviridae	L5_Desulfovibrionaceae	-0.030	0.037	4.13.E-01	9.18.E-01
Microviridae	L5_Desulfovibrionaceae	0.017	0.037	6.58.E-01	9.79.E-01
Myoviridae	L5_Desulfovibrionaceae	0.018	0.042	6.71.E-01	9.80.E-01
Phycodnaviridae	L5_Desulfovibrionaceae	-0.016	0.037	6.68.E-01	9.80.E-01
Podoviridae	L5_Desulfovibrionaceae	-0.020	0.043	6.44.E-01	9.79.E-01
Siphoviridae	L5_Desulfovibrionaceae	-0.022	0.038	5.69.E-01	9.61.E-01
Autographiviridae	L5_Porphyromonadaceae	0.006	0.027	8.36.E-01	9.94.E-01

crAss_like_phage	L5_Porphyromonadaceae	0.031	0.029	2.88.E-01	9.15.E-01
Herelleviridae	L5_Porphyromonadaceae	0.045	0.028	1.04.E-01	7.82.E-01
Microviridae	L5_Porphyromonadaceae	0.004	0.028	8.74.E-01	9.96.E-01
Myoviridae	L5_Porphyromonadaceae	-0.054	0.031	8.13.E-02	7.47.E-01
Phycodnaviridae	L5_Porphyromonadaceae	-0.012	0.027	6.52.E-01	9.79.E-01
Podoviridae	L5_Porphyromonadaceae	-0.002	0.032	9.51.E-01	9.98.E-01
Siphoviridae	L5_Porphyromonadaceae	0.030	0.028	2.99.E-01	9.15.E-01
Autographiviridae	L5_f_Fenollaria	0.031	0.042	4.61.E-01	9.43.E-01
crAss_like_phage	L5_f_Fenollaria	-0.002	0.046	9.63.E-01	9.98.E-01
Herelleviridae	L5_f_Fenollaria	0.084	0.043	5.12.E-02	7.22.E-01
Microviridae	L5_f_Fenollaria	0.021	0.043	6.28.E-01	9.76.E-01
Myoviridae	L5_f_Fenollaria	-0.050	0.048	2.96.E-01	9.15.E-01
Phycodnaviridae	L5_f_Fenollaria	-0.041	0.043	3.40.E-01	9.15.E-01
Podoviridae	L5_f_Fenollaria	-0.013	0.050	7.95.E-01	9.92.E-01
Siphoviridae	L5_f_Fenollaria	0.031	0.044	4.82.E-01	9.53.E-01
Autographiviridae	L5_Methanobacteriaceae	0.048	0.036	1.84.E-01	8.60.E-01
crAss_like_phage	L5_Methanobacteriaceae	-0.028	0.040	4.82.E-01	9.53.E-01
Herelleviridae	L5_Methanobacteriaceae	0.067	0.037	7.32.E-02	7.26.E-01
Microviridae	L5_Methanobacteriaceae	0.049	0.037	1.90.E-01	8.61.E-01
Myoviridae	L5_Methanobacteriaceae	0.009	0.042	8.35.E-01	9.94.E-01
Phycodnaviridae	L5_Methanobacteriaceae	-0.022	0.037	5.53.E-01	9.60.E-01
Podoviridae	L5_Methanobacteriaceae	-0.017	0.043	7.02.E-01	9.81.E-01
Siphoviridae	L5_Methanobacteriaceae	-0.017	0.038	6.63.E-01	9.79.E-01
Autographiviridae	L5_Campylobacteraceae	-0.002	0.028	9.56.E-01	9.98.E-01
crAss_like_phage	L5_Campylobacteraceae	0.001	0.031	9.80.E-01	9.99.E-01
Herelleviridae	L5_Campylobacteraceae	0.016	0.029	5.86.E-01	9.65.E-01
Microviridae	L5_Campylobacteraceae	-0.034	0.029	2.39.E-01	8.85.E-01
Myoviridae	L5_Campylobacteraceae	-0.005	0.032	8.84.E-01	9.96.E-01
Phycodnaviridae	L5_Campylobacteraceae	-0.001	0.029	9.59.E-01	9.98.E-01
Podoviridae	L5_Campylobacteraceae	-0.033	0.033	3.17.E-01	9.15.E-01
Siphoviridae	L5_Campylobacteraceae	-0.015	0.030	6.19.E-01	9.75.E-01
Autographiviridae	L5_Streptococcaceae	-0.006	0.025	7.99.E-01	9.92.E-01
crAss_like_phage	L5_Streptococcaceae	0.018	0.027	5.06.E-01	9.57.E-01
Herelleviridae	L5_Streptococcaceae	0.028	0.025	2.69.E-01	9.01.E-01
Microviridae	L5_Streptococcaceae	0.031	0.026	2.19.E-01	8.79.E-01
Myoviridae	L5_Streptococcaceae	0.030	0.028	2.98.E-01	9.15.E-01
Phycodnaviridae	L5_Streptococcaceae	0.000	0.025	9.92.E-01	9.99.E-01
Podoviridae	L5_Streptococcaceae	0.043	0.029	1.48.E-01	8.22.E-01
Siphoviridae	L5_Streptococcaceae	0.039	0.026	1.33.E-01	8.05.E-01
Autographiviridae	L5_Microbacteriaceae	-0.010	0.018	5.75.E-01	9.62.E-01
crAss_like_phage	L5_Microbacteriaceae	-0.001	0.020	9.46.E-01	9.98.E-01
Herelleviridae	L5_Microbacteriaceae	0.018	0.018	3.19.E-01	9.15.E-01
Microviridae	L5_Microbacteriaceae	0.005	0.018	7.76.E-01	9.92.E-01
Myoviridae	L5_Microbacteriaceae	0.003	0.021	8.71.E-01	9.96.E-01
Phycodnaviridae	L5_Microbacteriaceae	0.017	0.018	3.42.E-01	9.15.E-01
Podoviridae	L5_Microbacteriaceae	-0.006	0.021	7.90.E-01	9.92.E-01
Siphoviridae	L5_Microbacteriaceae	-0.027	0.019	1.54.E-01	8.26.E-01
Autographiviridae	L5_Corynebacteriaceae	-0.005	0.036	8.86.E-01	9.96.E-01
crAss_like_phage	L5_Corynebacteriaceae	0.076	0.040	5.77.E-02	7.24.E-01
Herelleviridae	L5_Corynebacteriaceae	-0.007	0.038	8.49.E-01	9.95.E-01
Microviridae	L5_Corynebacteriaceae	0.073	0.038	5.28.E-02	7.22.E-01
Myoviridae	L5_Corynebacteriaceae	0.057	0.042	1.76.E-01	8.58.E-01
Phycodnaviridae	L5_Corynebacteriaceae	-0.039	0.037	2.92.E-01	9.15.E-01
Podoviridae	L5_Corynebacteriaceae	0.065	0.043	1.33.E-01	8.05.E-01
Siphoviridae	L5_Corynebacteriaceae	0.003	0.039	9.35.E-01	9.98.E-01
Autographiviridae	L5_Clostridiaceae	-0.001	0.031	9.68.E-01	9.98.E-01
crAss_like_phage	L5_Clostridiaceae	0.020	0.034	5.56.E-01	9.61.E-01
Herelleviridae	L5_Clostridiaceae	0.074	0.032	2.06.E-02	6.02.E-01
Microviridae	L5_Clostridiaceae	0.002	0.032	9.50.E-01	9.98.E-01
Myoviridae	L5_Clostridiaceae	0.038	0.036	2.91.E-01	9.15.E-01
Phycodnaviridae	L5_Clostridiaceae	0.048	0.032	1.31.E-01	8.00.E-01
Podoviridae	L5_Clostridiaceae	-0.006	0.037	8.74.E-01	9.96.E-01
Siphoviridae	L5_Clostridiaceae	0.000	0.033	9.99.E-01	9.99.E-01
Autographiviridae	L5_Acetobacteraceae	0.051	0.039	1.88.E-01	8.61.E-01
crAss_like_phage	L5_Acetobacteraceae	-0.123	0.043	3.98.E-03	4.05.E-01

Herelleviridae	L5_Acetobacteraceae	-0.019	0.040	6.31.E-01	9.76.E-01
Microviridae	L5_Acetobacteraceae	0.057	0.040	1.63.E-01	8.38.E-01
Myoviridae	L5_Acetobacteraceae	-0.027	0.045	5.54.E-01	9.61.E-01
Phycodnaviridae	L5_Acetobacteraceae	-0.044	0.040	2.68.E-01	9.01.E-01
Podoviridae	L5_Acetobacteraceae	-0.083	0.047	7.42.E-02	7.28.E-01
Siphoviridae	L5_Acetobacteraceae	-0.057	0.042	1.70.E-01	8.49.E-01
Autographiviridae	L5_f_Burkholderiales_bacterium	-0.005	0.038	8.91.E-01	9.96.E-01
crAss_like_phage	L5_f_Burkholderiales_bacterium	0.014	0.042	7.37.E-01	9.88.E-01
Herelleviridae	L5_f_Burkholderiales_bacterium	0.074	0.039	5.76.E-02	7.24.E-01
Microviridae	L5_f_Burkholderiales_bacterium	-0.045	0.039	2.49.E-01	8.94.E-01
Myoviridae	L5_f_Burkholderiales_bacterium	0.028	0.044	5.26.E-01	9.57.E-01
Phycodnaviridae	L5_f_Burkholderiales_bacterium	0.015	0.039	6.97.E-01	9.81.E-01
Podoviridae	L5_f_Burkholderiales_bacterium	-0.056	0.045	2.18.E-01	8.79.E-01
Siphoviridae	L5_f_Burkholderiales_bacterium	-0.026	0.040	5.18.E-01	9.57.E-01
Autographiviridae	L5_Staphylococcaceae	-0.014	0.040	7.19.E-01	9.86.E-01
crAss_like_phage	L5_Staphylococcaceae	-0.065	0.044	1.37.E-01	8.10.E-01
Herelleviridae	L5_Staphylococcaceae	-0.020	0.041	6.27.E-01	9.76.E-01
Microviridae	L5_Staphylococcaceae	-0.011	0.042	7.85.E-01	9.92.E-01
Myoviridae	L5_Staphylococcaceae	-0.023	0.046	6.13.E-01	9.75.E-01
Phycodnaviridae	L5_Staphylococcaceae	-0.023	0.041	5.80.E-01	9.64.E-01
Podoviridae	L5_Staphylococcaceae	-0.021	0.048	6.60.E-01	9.79.E-01
Siphoviridae	L5_Staphylococcaceae	-0.051	0.043	2.35.E-01	8.85.E-01
Autographiviridae	L5_Erysipelotrichaceae	0.040	0.041	3.34.E-01	9.15.E-01
crAss_like_phage	L5_Erysipelotrichaceae	0.008	0.045	8.67.E-01	9.96.E-01
Herelleviridae	L5_Erysipelotrichaceae	0.001	0.043	9.88.E-01	9.99.E-01
Microviridae	L5_Erysipelotrichaceae	0.054	0.043	2.09.E-01	8.76.E-01
Myoviridae	L5_Erysipelotrichaceae	0.007	0.048	8.91.E-01	9.96.E-01
Phycodnaviridae	L5_Erysipelotrichaceae	-0.005	0.042	9.14.E-01	9.98.E-01
Podoviridae	L5_Erysipelotrichaceae	-0.096	0.049	5.26.E-02	7.22.E-01
Siphoviridae	L5_Erysipelotrichaceae	-0.028	0.044	5.31.E-01	9.57.E-01
Autographiviridae	L5_Moraxellaceae	-0.054	0.044	2.18.E-01	8.79.E-01
crAss_like_phage	L5_Moraxellaceae	-0.045	0.049	3.52.E-01	9.15.E-01
Herelleviridae	L5_Moraxellaceae	-0.024	0.046	6.02.E-01	9.71.E-01
Microviridae	L5_Moraxellaceae	-0.002	0.046	9.72.E-01	9.99.E-01
Myoviridae	L5_Moraxellaceae	0.058	0.051	2.56.E-01	8.95.E-01
Phycodnaviridae	L5_Moraxellaceae	0.008	0.045	8.67.E-01	9.96.E-01
Podoviridae	L5_Moraxellaceae	0.034	0.053	5.24.E-01	9.57.E-01
Siphoviridae	L5_Moraxellaceae	0.044	0.047	3.47.E-01	9.15.E-01
Autographiviridae	L5_Intestinimonas	-0.004	0.027	8.74.E-01	9.96.E-01
crAss_like_phage	L5_Intestinimonas	0.010	0.029	7.24.E-01	9.86.E-01
Herelleviridae	L5_Intestinimonas	0.004	0.028	8.95.E-01	9.97.E-01
Microviridae	L5_Intestinimonas	0.043	0.028	1.23.E-01	7.94.E-01
Myoviridae	L5_Intestinimonas	0.069	0.031	2.56.E-02	6.50.E-01
Phycodnaviridae	L5_Intestinimonas	0.021	0.027	4.35.E-01	9.26.E-01
Podoviridae	L5_Intestinimonas	0.007	0.032	8.20.E-01	9.93.E-01
Siphoviridae	L5_Intestinimonas	0.005	0.028	8.56.E-01	9.96.E-01
Autographiviridae	L5_Akkermansiaceae	-0.009	0.039	8.18.E-01	9.93.E-01
crAss_like_phage	L5_Akkermansiaceae	0.013	0.043	7.69.E-01	9.91.E-01
Herelleviridae	L5_Akkermansiaceae	0.024	0.040	5.51.E-01	9.60.E-01
Microviridae	L5_Akkermansiaceae	-0.074	0.040	6.67.E-02	7.24.E-01
Myoviridae	L5_Akkermansiaceae	0.041	0.045	3.64.E-01	9.15.E-01
Phycodnaviridae	L5_Akkermansiaceae	0.054	0.040	1.77.E-01	8.58.E-01
Podoviridae	L5_Akkermansiaceae	0.092	0.046	4.68.E-02	7.22.E-01
Siphoviridae	L5_Akkermansiaceae	-0.004	0.041	9.28.E-01	9.98.E-01
Autographiviridae	L5_Actinomycetaceae	-0.054	0.027	5.10.E-02	7.22.E-01
crAss_like_phage	L5_Actinomycetaceae	-0.012	0.030	6.91.E-01	9.80.E-01
Herelleviridae	L5_Actinomycetaceae	-0.065	0.028	2.16.E-02	6.09.E-01
Microviridae	L5_Actinomycetaceae	0.008	0.029	7.74.E-01	9.92.E-01
Myoviridae	L5_Actinomycetaceae	-0.035	0.032	2.70.E-01	9.02.E-01
Phycodnaviridae	L5_Actinomycetaceae	-0.024	0.028	3.87.E-01	9.15.E-01
Podoviridae	L5_Actinomycetaceae	-0.013	0.033	7.04.E-01	9.81.E-01
Siphoviridae	L5_Actinomycetaceae	-0.005	0.029	8.72.E-01	9.96.E-01
Autographiviridae	L5_Oxalobacteraceae	-0.069	0.035	5.10.E-02	7.22.E-01
crAss_like_phage	L5_Oxalobacteraceae	0.002	0.039	9.53.E-01	9.98.E-01
Herelleviridae	L5_Oxalobacteraceae	-0.056	0.036	1.27.E-01	7.99.E-01

Microviridae	L5_Oxalobacteraceae	0.027	0.037	4.61.E-01	9.43.E-01
Myoviridae	L5_Oxalobacteraceae	-0.013	0.041	7.55.E-01	9.89.E-01
Phycodnaviridae	L5_Oxalobacteraceae	-0.055	0.036	1.31.E-01	8.00.E-01
Podoviridae	L5_Oxalobacteraceae	-0.061	0.042	1.51.E-01	8.22.E-01
Siphoviridae	L5_Oxalobacteraceae	-0.041	0.038	2.73.E-01	9.04.E-01
Autographiviridae	L5_Atopobiaceae	-0.002	0.038	9.52.E-01	9.98.E-01
crAss_like_phage	L5_Atopobiaceae	0.005	0.042	9.03.E-01	9.97.E-01
Herelleviridae	L5_Atopobiaceae	-0.028	0.039	4.78.E-01	9.52.E-01
Microviridae	L5_Atopobiaceae	-0.084	0.039	3.20.E-02	6.75.E-01
Myoviridae	L5_Atopobiaceae	0.010	0.044	8.23.E-01	9.93.E-01
Phycodnaviridae	L5_Atopobiaceae	-0.051	0.039	1.90.E-01	8.61.E-01
Podoviridae	L5_Atopobiaceae	-0.020	0.045	6.52.E-01	9.79.E-01
Siphoviridae	L5_Atopobiaceae	-0.057	0.040	1.59.E-01	8.38.E-01
Autographiviridae	L5_Micrococcaceae	0.049	0.030	1.05.E-01	7.82.E-01
crAss_like_phage	L5_Micrococcaceae	0.023	0.034	5.00.E-01	9.55.E-01
Herelleviridae	L5_Micrococcaceae	0.006	0.032	8.61.E-01	9.96.E-01
Microviridae	L5_Micrococcaceae	0.011	0.032	7.22.E-01	9.86.E-01
Myoviridae	L5_Micrococcaceae	0.118	0.035	8.31.E-04	2.79.E-01
Phycodnaviridae	L5_Micrococcaceae	0.000	0.031	9.98.E-01	9.99.E-01
Podoviridae	L5_Micrococcaceae	0.065	0.036	7.45.E-02	7.29.E-01
Siphoviridae	L5_Micrococcaceae	0.033	0.033	3.10.E-01	9.15.E-01
Autographiviridae	L5_Flavobacteriaceae	0.026	0.020	2.05.E-01	8.76.E-01
crAss_like_phage	L5_Flavobacteriaceae	-0.005	0.023	8.26.E-01	9.93.E-01
Herelleviridae	L5_Flavobacteriaceae	-0.026	0.021	2.15.E-01	8.79.E-01
Microviridae	L5_Flavobacteriaceae	0.009	0.021	6.58.E-01	9.79.E-01
Myoviridae	L5_Flavobacteriaceae	-0.026	0.024	2.75.E-01	9.05.E-01
Phycodnaviridae	L5_Flavobacteriaceae	0.009	0.021	6.78.E-01	9.80.E-01
Podoviridae	L5_Flavobacteriaceae	0.019	0.025	4.51.E-01	9.38.E-01
Siphoviridae	L5_Flavobacteriaceae	-0.039	0.022	7.37.E-02	7.26.E-01
Autographiviridae	L5_Helicobacteraceae	-0.006	0.032	8.61.E-01	9.96.E-01
crAss_like_phage	L5_Helicobacteraceae	-0.018	0.035	6.06.E-01	9.73.E-01
Herelleviridae	L5_Helicobacteraceae	-0.008	0.033	8.01.E-01	9.92.E-01
Microviridae	L5_Helicobacteraceae	-0.052	0.033	1.13.E-01	7.84.E-01
Myoviridae	L5_Helicobacteraceae	-0.007	0.037	8.41.E-01	9.95.E-01
Phycodnaviridae	L5_Helicobacteraceae	0.058	0.032	7.44.E-02	7.28.E-01
Podoviridae	L5_Helicobacteraceae	-0.018	0.038	6.39.E-01	9.78.E-01
Siphoviridae	L5_Helicobacteraceae	-0.004	0.034	9.02.E-01	9.97.E-01
Autographiviridae	L5_Fusobacteriaceae	-0.038	0.037	3.11.E-01	9.15.E-01
crAss_like_phage	L5_Fusobacteriaceae	-0.091	0.041	2.78.E-02	6.58.E-01
Herelleviridae	L5_Fusobacteriaceae	0.040	0.039	3.00.E-01	9.15.E-01
Microviridae	L5_Fusobacteriaceae	0.001	0.039	9.73.E-01	9.99.E-01
Myoviridae	L5_Fusobacteriaceae	0.069	0.043	1.11.E-01	7.82.E-01
Phycodnaviridae	L5_Fusobacteriaceae	0.046	0.038	2.28.E-01	8.82.E-01
Podoviridae	L5_Fusobacteriaceae	0.111	0.045	1.34.E-02	5.51.E-01
Siphoviridae	L5_Fusobacteriaceae	-0.021	0.040	6.05.E-01	9.73.E-01
Autographiviridae	L5_Barnesiellaceae	-0.064	0.033	5.57.E-02	7.24.E-01
crAss_like_phage	L5_Barnesiellaceae	0.032	0.037	3.91.E-01	9.15.E-01
Herelleviridae	L5_Barnesiellaceae	0.008	0.035	8.26.E-01	9.93.E-01
Microviridae	L5_Barnesiellaceae	0.047	0.035	1.80.E-01	8.60.E-01
Myoviridae	L5_Barnesiellaceae	0.003	0.039	9.31.E-01	9.98.E-01
Phycodnaviridae	L5_Barnesiellaceae	-0.017	0.034	6.29.E-01	9.76.E-01
Podoviridae	L5_Barnesiellaceae	-0.064	0.040	1.14.E-01	7.85.E-01
Siphoviridae	L5_Barnesiellaceae	0.038	0.036	2.89.E-01	9.15.E-01
Autographiviridae	L5_Bacillaceae	0.011	0.044	8.10.E-01	9.93.E-01
crAss_like_phage	L5_Bacillaceae	0.082	0.049	9.49.E-02	7.64.E-01
Herelleviridae	L5_Bacillaceae	-0.027	0.046	5.58.E-01	9.61.E-01
Microviridae	L5_Bacillaceae	0.095	0.046	3.91.E-02	7.03.E-01
Myoviridae	L5_Bacillaceae	0.112	0.051	2.98.E-02	6.66.E-01
Phycodnaviridae	L5_Bacillaceae	0.064	0.045	1.61.E-01	8.38.E-01
Podoviridae	L5_Bacillaceae	0.061	0.053	2.49.E-01	8.94.E-01
Siphoviridae	L5_Bacillaceae	0.093	0.047	4.83.E-02	7.22.E-01
Autographiviridae	L5_Bifidobacteriaceae	-0.006	0.023	7.76.E-01	9.92.E-01
crAss_like_phage	L5_Bifidobacteriaceae	-0.027	0.025	2.74.E-01	9.04.E-01
Herelleviridae	L5_Bifidobacteriaceae	0.013	0.023	5.73.E-01	9.61.E-01
Microviridae	L5_Bifidobacteriaceae	0.055	0.023	1.80.E-02	5.86.E-01

Myoviridae	L5_Bifidobacteriaceae	0.027	0.026	3.06.E-01	9.15.E-01
Phycodnaviridae	L5_Bifidobacteriaceae	0.003	0.023	8.87.E-01	9.96.E-01
Podoviridae	L5_Bifidobacteriaceae	0.024	0.027	3.82.E-01	9.15.E-01
Siphoviridae	L5_Bifidobacteriaceae	0.047	0.024	4.89.E-02	7.22.E-01
Autographiviridae	L5_Eubacteriaceae	0.031	0.033	3.44.E-01	9.15.E-01
crAss_like_phage	L5_Eubacteriaceae	-0.050	0.036	1.67.E-01	8.44.E-01
Herelleviridae	L5_Eubacteriaceae	0.045	0.034	1.80.E-01	8.60.E-01
Microviridae	L5_Eubacteriaceae	-0.013	0.034	6.98.E-01	9.81.E-01
Myoviridae	L5_Eubacteriaceae	-0.046	0.038	2.28.E-01	8.82.E-01
Phycodnaviridae	L5_Eubacteriaceae	-0.006	0.034	8.62.E-01	9.96.E-01
Podoviridae	L5_Eubacteriaceae	-0.127	0.039	1.17.E-03	2.88.E-01
Siphoviridae	L5_Eubacteriaceae	0.001	0.035	9.80.E-01	9.99.E-01
Autographiviridae	L5_Carnobacteriaceae	-0.033	0.025	1.86.E-01	8.61.E-01
crAss_like_phage	L5_Carnobacteriaceae	0.021	0.028	4.54.E-01	9.39.E-01
Herelleviridae	L5_Carnobacteriaceae	-0.069	0.026	7.70.E-03	4.66.E-01
Microviridae	L5_Carnobacteriaceae	0.012	0.026	6.36.E-01	9.77.E-01
Myoviridae	L5_Carnobacteriaceae	-0.064	0.029	2.66.E-02	6.58.E-01
Phycodnaviridae	L5_Carnobacteriaceae	-0.035	0.026	1.76.E-01	8.58.E-01
Podoviridae	L5_Carnobacteriaceae	0.061	0.030	4.21.E-02	7.16.E-01
Siphoviridae	L5_Carnobacteriaceae	0.013	0.027	6.26.E-01	9.76.E-01
Autographiviridae	L5_Lactobacillaceae	-0.039	0.028	1.62.E-01	8.38.E-01
crAss_like_phage	L5_Lactobacillaceae	-0.021	0.031	4.98.E-01	9.55.E-01
Herelleviridae	L5_Lactobacillaceae	0.025	0.029	3.88.E-01	9.15.E-01
Microviridae	L5_Lactobacillaceae	-0.015	0.029	6.03.E-01	9.72.E-01
Myoviridae	L5_Lactobacillaceae	-0.009	0.033	7.82.E-01	9.92.E-01
Phycodnaviridae	L5_Lactobacillaceae	0.034	0.029	2.35.E-01	8.85.E-01
Podoviridae	L5_Lactobacillaceae	-0.005	0.034	8.84.E-01	9.96.E-01
Siphoviridae	L5_Lactobacillaceae	0.066	0.030	2.81.E-02	6.59.E-01
Autographiviridae	L5_Nocardioidaceae	-0.045	0.038	2.37.E-01	8.85.E-01
crAss_like_phage	L5_Nocardioidaceae	-0.031	0.042	4.58.E-01	9.42.E-01
Herelleviridae	L5_Nocardioidaceae	0.069	0.039	7.99.E-02	7.44.E-01
Microviridae	L5_Nocardioidaceae	0.033	0.040	4.01.E-01	9.15.E-01
Myoviridae	L5_Nocardioidaceae	-0.011	0.044	7.97.E-01	9.92.E-01
Phycodnaviridae	L5_Nocardioidaceae	0.013	0.039	7.46.E-01	9.89.E-01
Podoviridae	L5_Nocardioidaceae	0.118	0.045	9.62.E-03	4.94.E-01
Siphoviridae	L5_Nocardioidaceae	0.041	0.041	3.11.E-01	9.15.E-01
Autographiviridae	L5_Eggerthellaceae	0.005	0.034	8.77.E-01	9.96.E-01
crAss_like_phage	L5_Eggerthellaceae	-0.027	0.038	4.78.E-01	9.52.E-01
Herelleviridae	L5_Eggerthellaceae	0.058	0.036	1.04.E-01	7.82.E-01
Microviridae	L5_Eggerthellaceae	-0.031	0.036	3.83.E-01	9.15.E-01
Myoviridae	L5_Eggerthellaceae	-0.047	0.040	2.38.E-01	8.85.E-01
Phycodnaviridae	L5_Eggerthellaceae	0.006	0.035	8.64.E-01	9.96.E-01
Podoviridae	L5_Eggerthellaceae	0.057	0.041	1.65.E-01	8.43.E-01
Siphoviridae	L5_Eggerthellaceae	-0.067	0.037	6.82.E-02	7.24.E-01
Autographiviridae	L5_f_butyrate.producing_bacterium	-0.030	0.035	3.89.E-01	9.15.E-01
crAss_like_phage	L5_f_butyrate.producing_bacterium	-0.035	0.039	3.68.E-01	9.15.E-01
Herelleviridae	L5_f_butyrate.producing_bacterium	-0.051	0.036	1.63.E-01	8.38.E-01
Microviridae	L5_f_butyrate.producing_bacterium	-0.042	0.037	2.54.E-01	8.95.E-01
Myoviridae	L5_f_butyrate.producing_bacterium	0.008	0.041	8.49.E-01	9.95.E-01
Phycodnaviridae	L5_f_butyrate.producing_bacterium	-0.023	0.036	5.27.E-01	9.57.E-01
Podoviridae	L5_f_butyrate.producing_bacterium	0.010	0.042	8.11.E-01	9.93.E-01
Siphoviridae	L5_f_butyrate.producing_bacterium	-0.016	0.038	6.71.E-01	9.80.E-01
Autographiviridae	L5_Clostridiales_Family_XIII_Incertae_Sedis	0.047	0.038	2.19.E-01	8.79.E-01
crAss_like_phage	L5_Clostridiales_Family_XIII_Incertae_Sedis	-0.014	0.042	7.47.E-01	9.89.E-01
Herelleviridae	L5_Clostridiales_Family_XIII_Incertae_Sedis	-0.042	0.040	2.92.E-01	9.15.E-01
Microviridae	L5_Clostridiales_Family_XIII_Incertae_Sedis	0.064	0.040	1.07.E-01	7.82.E-01
Myoviridae	L5_Clostridiales_Family_XIII_Incertae_Sedis	0.002	0.045	9.66.E-01	9.98.E-01
Phycodnaviridae	L5_Clostridiales_Family_XIII_Incertae_Sedis	0.026	0.039	5.11.E-01	9.57.E-01
Podoviridae	L5_Clostridiales_Family_XIII_Incertae_Sedis	0.020	0.046	6.57.E-01	9.79.E-01
Siphoviridae	L5_Clostridiales_Family_XIII_Incertae_Sedis	0.012	0.041	7.69.E-01	9.91.E-01
Autographiviridae	L5_Christensenellaceae	0.013	0.031	6.75.E-01	9.80.E-01
crAss_like_phage	L5_Christensenellaceae	-0.034	0.034	3.24.E-01	9.15.E-01
Herelleviridae	L5_Christensenellaceae	-0.055	0.032	8.73.E-02	7.52.E-01
Microviridae	L5_Christensenellaceae	0.018	0.032	5.70.E-01	9.61.E-01
Myoviridae	L5_Christensenellaceae	0.017	0.036	6.43.E-01	9.79.E-01

Phycodnaviridae	L5_Christensenellaceae	-0.069	0.032	3.03.E-02	6.66.E-01
Podoviridae	L5_Christensenellaceae	0.009	0.037	8.07.E-01	9.93.E-01
Siphoviridae	L5_Christensenellaceae	-0.010	0.033	7.51.E-01	9.89.E-01
Autographiviridae	L5_Succinivibrionaceae	-0.003	0.041	9.39.E-01	9.98.E-01
crAss_like_phage	L5_Succinivibrionaceae	0.015	0.045	7.44.E-01	9.89.E-01
Herelleviridae	L5_Succinivibrionaceae	-0.029	0.043	5.02.E-01	9.55.E-01
Microviridae	L5_Succinivibrionaceae	0.007	0.043	8.64.E-01	9.96.E-01
Myoviridae	L5_Succinivibrionaceae	-0.008	0.048	8.66.E-01	9.96.E-01
Phycodnaviridae	L5_Succinivibrionaceae	0.050	0.042	2.35.E-01	8.85.E-01
Podoviridae	L5_Succinivibrionaceae	-0.027	0.049	5.91.E-01	9.68.E-01
Siphoviridae	L5_Succinivibrionaceae	0.014	0.044	7.45.E-01	9.89.E-01
Autographiviridae	L5_Odoribacteraceae	-0.006	0.019	7.60.E-01	9.91.E-01
crAss_like_phage	L5_Odoribacteraceae	0.020	0.021	3.41.E-01	9.15.E-01
Herelleviridae	L5_Odoribacteraceae	0.005	0.020	8.10.E-01	9.93.E-01
Microviridae	L5_Odoribacteraceae	0.019	0.020	3.24.E-01	9.15.E-01
Myoviridae	L5_Odoribacteraceae	-0.026	0.022	2.40.E-01	8.85.E-01
Phycodnaviridae	L5_Odoribacteraceae	0.044	0.019	2.21.E-02	6.19.E-01
Podoviridae	L5_Odoribacteraceae	0.013	0.023	5.77.E-01	9.63.E-01
Siphoviridae	L5_Odoribacteraceae	0.020	0.020	3.31.E-01	9.15.E-01
Autographiviridae	L5_f_Clostridiales_bacterium	0.022	0.027	4.20.E-01	9.20.E-01
crAss_like_phage	L5_f_Clostridiales_bacterium	0.027	0.030	3.59.E-01	9.15.E-01
Herelleviridae	L5_f_Clostridiales_bacterium	0.012	0.028	6.61.E-01	9.79.E-01
Microviridae	L5_f_Clostridiales_bacterium	-0.016	0.028	5.79.E-01	9.64.E-01
Myoviridae	L5_f_Clostridiales_bacterium	0.092	0.031	3.14.E-03	3.87.E-01
Phycodnaviridae	L5_f_Clostridiales_bacterium	-0.017	0.028	5.46.E-01	9.60.E-01
Podoviridae	L5_f_Clostridiales_bacterium	-0.081	0.032	1.20.E-02	5.36.E-01
Siphoviridae	L5_f_Clostridiales_bacterium	0.036	0.029	2.08.E-01	8.76.E-01
Autographiviridae	L5_Coriobacteriaceae	0.015	0.033	6.41.E-01	9.79.E-01
crAss_like_phage	L5_Coriobacteriaceae	-0.038	0.036	2.97.E-01	9.15.E-01
Herelleviridae	L5_Coriobacteriaceae	0.020	0.034	5.51.E-01	9.60.E-01
Microviridae	L5_Coriobacteriaceae	-0.045	0.034	1.87.E-01	8.61.E-01
Myoviridae	L5_Coriobacteriaceae	-0.043	0.038	2.60.E-01	8.95.E-01
Phycodnaviridae	L5_Coriobacteriaceae	0.032	0.034	3.40.E-01	9.15.E-01
Podoviridae	L5_Coriobacteriaceae	-0.038	0.039	3.40.E-01	9.15.E-01
Siphoviridae	L5_Coriobacteriaceae	-0.044	0.035	2.16.E-01	8.79.E-01
Autographiviridae	L5_Aeromonadaceae	-0.013	0.041	7.44.E-01	9.89.E-01
crAss_like_phage	L5_Aeromonadaceae	0.025	0.045	5.84.E-01	9.65.E-01
Herelleviridae	L5_Aeromonadaceae	-0.084	0.042	4.60.E-02	7.22.E-01
Microviridae	L5_Aeromonadaceae	0.021	0.042	6.17.E-01	9.75.E-01
Myoviridae	L5_Aeromonadaceae	0.014	0.047	7.66.E-01	9.91.E-01
Phycodnaviridae	L5_Aeromonadaceae	-0.057	0.042	1.76.E-01	8.57.E-01
Podoviridae	L5_Aeromonadaceae	-0.093	0.049	5.73.E-02	7.24.E-01
Siphoviridae	L5_Aeromonadaceae	0.016	0.043	7.07.E-01	9.83.E-01
Autographiviridae	L5_Rhodospirillaceae	0.042	0.039	2.87.E-01	9.15.E-01
crAss_like_phage	L5_Rhodospirillaceae	-0.083	0.043	5.44.E-02	7.23.E-01
Herelleviridae	L5_Rhodospirillaceae	-0.049	0.041	2.29.E-01	8.82.E-01
Microviridae	L5_Rhodospirillaceae	-0.002	0.041	9.62.E-01	9.98.E-01
Myoviridae	L5_Rhodospirillaceae	-0.061	0.046	1.82.E-01	8.60.E-01
Phycodnaviridae	L5_Rhodospirillaceae	-0.042	0.040	3.01.E-01	9.15.E-01
Podoviridae	L5_Rhodospirillaceae	0.013	0.047	7.88.E-01	9.92.E-01
Siphoviridae	L5_Rhodospirillaceae	0.006	0.042	8.90.E-01	9.96.E-01
Autographiviridae	L5_f_Bacteroidetes_bacterium	-0.045	0.040	2.69.E-01	9.01.E-01
crAss_like_phage	L5_f_Bacteroidetes_bacterium	-0.012	0.045	7.81.E-01	9.92.E-01
Herelleviridae	L5_f_Bacteroidetes_bacterium	0.018	0.042	6.59.E-01	9.79.E-01
Microviridae	L5_f_Bacteroidetes_bacterium	0.047	0.042	2.60.E-01	8.95.E-01
Myoviridae	L5_f_Bacteroidetes_bacterium	-0.032	0.047	4.99.E-01	9.55.E-01
Phycodnaviridae	L5_f_Bacteroidetes_bacterium	0.046	0.041	2.65.E-01	9.01.E-01
Podoviridae	L5_f_Bacteroidetes_bacterium	0.053	0.048	2.72.E-01	9.02.E-01
Siphoviridae	L5_f_Bacteroidetes_bacterium	0.036	0.043	4.08.E-01	9.17.E-01
Autographiviridae	L5_Lachnospiraceae	0.008	0.027	7.54.E-01	9.89.E-01
crAss_like_phage	L5_Lachnospiraceae	-0.043	0.029	1.42.E-01	8.12.E-01
Herelleviridae	L5_Lachnospiraceae	-0.023	0.028	4.17.E-01	9.19.E-01
Microviridae	L5_Lachnospiraceae	-0.035	0.028	2.05.E-01	8.76.E-01
Myoviridae	L5_Lachnospiraceae	-0.050	0.031	1.04.E-01	7.82.E-01
Phycodnaviridae	L5_Lachnospiraceae	0.042	0.027	1.26.E-01	7.98.E-01

Podoviridae	L5_Lachnospiraceae	-0.011	0.032	7.20.E-01	9.86.E-01
Siphoviridae	L5_Lachnospiraceae	-0.027	0.028	3.35.E-01	9.15.E-01
Autographiviridae	L5_Aerococcaceae	0.023	0.037	5.25.E-01	9.57.E-01
crAss_like_phage	L5_Aerococcaceae	-0.040	0.041	3.24.E-01	9.15.E-01
Herelleviridae	L5_Aerococcaceae	-0.021	0.038	5.85.E-01	9.65.E-01
Microviridae	L5_Aerococcaceae	0.058	0.038	1.27.E-01	7.98.E-01
Myoviridae	L5_Aerococcaceae	0.035	0.043	4.07.E-01	9.17.E-01
Phycodnaviridae	L5_Aerococcaceae	-0.064	0.038	9.02.E-02	7.58.E-01
Podoviridae	L5_Aerococcaceae	0.022	0.044	6.14.E-01	9.75.E-01
Siphoviridae	L5_Aerococcaceae	-0.011	0.039	7.78.E-01	9.92.E-01
Autographiviridae	L5_Brachyspiraceae	-0.032	0.039	4.17.E-01	9.19.E-01
crAss_like_phage	L5_Brachyspiraceae	-0.034	0.043	4.30.E-01	9.25.E-01
Herelleviridae	L5_Brachyspiraceae	-0.005	0.041	8.95.E-01	9.97.E-01
Microviridae	L5_Brachyspiraceae	0.054	0.041	1.87.E-01	8.61.E-01
Myoviridae	L5_Brachyspiraceae	-0.021	0.045	6.47.E-01	9.79.E-01
Phycodnaviridae	L5_Brachyspiraceae	0.021	0.040	6.02.E-01	9.71.E-01
Podoviridae	L5_Brachyspiraceae	-0.060	0.047	1.97.E-01	8.66.E-01
Siphoviridae	L5_Brachyspiraceae	-0.018	0.042	6.68.E-01	9.80.E-01
Autographiviridae	L5_Prevotellaceae	0.010	0.031	7.56.E-01	9.89.E-01
crAss_like_phage	L5_Prevotellaceae	-0.015	0.034	6.52.E-01	9.79.E-01
Herelleviridae	L5_Prevotellaceae	-0.011	0.032	7.41.E-01	9.89.E-01
Microviridae	L5_Prevotellaceae	-0.010	0.032	7.50.E-01	9.89.E-01
Myoviridae	L5_Prevotellaceae	-0.018	0.036	6.15.E-01	9.75.E-01
Phycodnaviridae	L5_Prevotellaceae	-0.029	0.032	3.71.E-01	9.15.E-01
Podoviridae	L5_Prevotellaceae	0.034	0.037	3.55.E-01	9.15.E-01
Siphoviridae	L5_Prevotellaceae	0.041	0.033	2.13.E-01	8.78.E-01
Autographiviridae	L5_Thermaceae	0.000	0.008	9.51.E-01	9.98.E-01
crAss_like_phage	L5_Thermaceae	-0.014	0.009	1.05.E-01	7.82.E-01
Herelleviridae	L5_Thermaceae	-0.003	0.008	7.17.E-01	9.86.E-01
Microviridae	L5_Thermaceae	0.008	0.008	3.32.E-01	9.15.E-01
Myoviridae	L5_Thermaceae	-0.007	0.009	4.55.E-01	9.39.E-01
Phycodnaviridae	L5_Thermaceae	-0.005	0.008	5.73.E-01	9.61.E-01
Podoviridae	L5_Thermaceae	-0.010	0.009	3.04.E-01	9.15.E-01
Siphoviridae	L5_Thermaceae	-0.011	0.008	1.84.E-01	8.60.E-01
Autographiviridae	L5_Rikenellaceae	0.010	0.026	6.84.E-01	9.80.E-01
crAss_like_phage	L5_Rikenellaceae	0.045	0.028	1.07.E-01	7.82.E-01
Herelleviridae	L5_Rikenellaceae	0.026	0.026	3.21.E-01	9.15.E-01
Microviridae	L5_Rikenellaceae	0.004	0.027	8.84.E-01	9.96.E-01
Myoviridae	L5_Rikenellaceae	-0.021	0.030	4.88.E-01	9.54.E-01
Phycodnaviridae	L5_Rikenellaceae	-0.022	0.026	3.91.E-01	9.15.E-01
Podoviridae	L5_Rikenellaceae	0.063	0.030	4.02.E-02	7.03.E-01
Siphoviridae	L5_Rikenellaceae	-0.017	0.027	5.45.E-01	9.60.E-01
Autographiviridae	L5_Acidaminococcaceae	0.040	0.039	2.99.E-01	9.15.E-01
crAss_like_phage	L5_Acidaminococcaceae	0.019	0.043	6.62.E-01	9.79.E-01
Herelleviridae	L5_Acidaminococcaceae	0.026	0.040	5.24.E-01	9.57.E-01
Microviridae	L5_Acidaminococcaceae	0.019	0.040	6.35.E-01	9.76.E-01
Myoviridae	L5_Acidaminococcaceae	0.163	0.044	2.70.E-04	1.92.E-01
Phycodnaviridae	L5_Acidaminococcaceae	0.064	0.040	1.11.E-01	7.82.E-01
Podoviridae	L5_Acidaminococcaceae	0.085	0.046	6.86.E-02	7.24.E-01
Siphoviridae	L5_Acidaminococcaceae	0.057	0.041	1.68.E-01	8.44.E-01
Autographiviridae	L5_Tannerellaceae	-0.007	0.025	7.89.E-01	9.92.E-01
crAss_like_phage	L5_Tannerellaceae	0.005	0.027	8.48.E-01	9.95.E-01
Herelleviridae	L5_Tannerellaceae	0.006	0.026	8.19.E-01	9.93.E-01
Microviridae	L5_Tannerellaceae	-0.019	0.026	4.75.E-01	9.52.E-01
Myoviridae	L5_Tannerellaceae	-0.071	0.029	1.41.E-02	5.51.E-01
Phycodnaviridae	L5_Tannerellaceae	0.024	0.026	3.55.E-01	9.15.E-01
Podoviridae	L5_Tannerellaceae	0.026	0.030	3.77.E-01	9.15.E-01
Siphoviridae	L5_Tannerellaceae	-0.002	0.027	9.35.E-01	9.98.E-01
Autographiviridae	L5_Peptostreptococcaceae	0.014	0.032	6.58.E-01	9.79.E-01
crAss_like_phage	L5_Peptostreptococcaceae	-0.057	0.035	1.01.E-01	7.79.E-01
Herelleviridae	L5_Peptostreptococcaceae	0.026	0.033	4.35.E-01	9.26.E-01
Microviridae	L5_Peptostreptococcaceae	-0.055	0.033	9.61.E-02	7.69.E-01
Myoviridae	L5_Peptostreptococcaceae	0.049	0.037	1.82.E-01	8.60.E-01
Phycodnaviridae	L5_Peptostreptococcaceae	0.039	0.032	2.22.E-01	8.80.E-01
Podoviridae	L5_Peptostreptococcaceae	0.011	0.038	7.75.E-01	9.92.E-01

Siphoviridae	L5_Peptostreptococcaceae	0.014	0.034	6.72.E-01	9.80.E-01
Autographiviridae	L5_f_Firmicutes_bacterium	-0.024	0.024	3.27.E-01	9.15.E-01
crAss_like_phage	L5_f_Firmicutes_bacterium	0.032	0.027	2.29.E-01	8.82.E-01
Herelleviridae	L5_f_Firmicutes_bacterium	0.001	0.025	9.83.E-01	9.99.E-01
Microviridae	L5_f_Firmicutes_bacterium	0.023	0.025	3.74.E-01	9.15.E-01
Myoviridae	L5_f_Firmicutes_bacterium	-0.019	0.028	4.98.E-01	9.55.E-01
Phycodnaviridae	L5_f_Firmicutes_bacterium	0.030	0.025	2.31.E-01	8.82.E-01
Podoviridae	L5_f_Firmicutes_bacterium	0.008	0.029	7.78.E-01	9.92.E-01
Siphoviridae	L5_f_Firmicutes_bacterium	0.007	0.026	7.90.E-01	9.92.E-01
Autographiviridae	L5_Sutterellaceae	0.058	0.035	9.73.E-02	7.74.E-01
crAss_like_phage	L5_Sutterellaceae	0.043	0.039	2.70.E-01	9.02.E-01
Herelleviridae	L5_Sutterellaceae	-0.031	0.036	3.91.E-01	9.15.E-01
Microviridae	L5_Sutterellaceae	-0.003	0.036	9.36.E-01	9.98.E-01
Myoviridae	L5_Sutterellaceae	0.033	0.041	4.14.E-01	9.18.E-01
Phycodnaviridae	L5_Sutterellaceae	0.002	0.036	9.52.E-01	9.98.E-01
Podoviridae	L5_Sutterellaceae	-0.014	0.042	7.36.E-01	9.88.E-01
Siphoviridae	L5_Sutterellaceae	-0.004	0.037	9.06.E-01	9.97.E-01
Autographiviridae	L5_Peptococcaceae	-0.001	0.035	9.76.E-01	9.99.E-01
crAss_like_phage	L5_Peptococcaceae	-0.046	0.038	2.29.E-01	8.82.E-01
Herelleviridae	L5_Peptococcaceae	0.003	0.036	9.32.E-01	9.98.E-01
Microviridae	L5_Peptococcaceae	0.012	0.036	7.42.E-01	9.89.E-01
Myoviridae	L5_Peptococcaceae	0.075	0.040	6.06.E-02	7.24.E-01
Phycodnaviridae	L5_Peptococcaceae	-0.037	0.036	2.93.E-01	9.15.E-01
Podoviridae	L5_Peptococcaceae	0.003	0.042	9.48.E-01	9.98.E-01
Siphoviridae	L5_Peptococcaceae	0.033	0.037	3.70.E-01	9.15.E-01
Autographiviridae	L5_f_Pseudoflavonifractor	-0.005	0.026	8.41.E-01	9.95.E-01
crAss_like_phage	L5_f_Pseudoflavonifractor	-0.075	0.029	9.49.E-03	4.94.E-01
Herelleviridae	L5_f_Pseudoflavonifractor	-0.025	0.027	3.68.E-01	9.15.E-01
Microviridae	L5_f_Pseudoflavonifractor	-0.003	0.027	9.18.E-01	9.98.E-01
Myoviridae	L5_f_Pseudoflavonifractor	-0.003	0.030	9.09.E-01	9.98.E-01
Phycodnaviridae	L5_f_Pseudoflavonifractor	-0.033	0.027	2.25.E-01	8.82.E-01
Podoviridae	L5_f_Pseudoflavonifractor	-0.028	0.031	3.78.E-01	9.15.E-01
Siphoviridae	L5_f_Pseudoflavonifractor	0.009	0.028	7.44.E-01	9.89.E-01
Autographiviridae	L5_Pseudomonadaceae	-0.052	0.039	1.85.E-01	8.61.E-01
crAss_like_phage	L5_Pseudomonadaceae	-0.072	0.043	9.48.E-02	7.64.E-01
Herelleviridae	L5_Pseudomonadaceae	-0.044	0.041	2.83.E-01	9.13.E-01
Microviridae	L5_Pseudomonadaceae	0.033	0.041	4.27.E-01	9.24.E-01
Myoviridae	L5_Pseudomonadaceae	0.002	0.046	9.63.E-01	9.98.E-01
Phycodnaviridae	L5_Pseudomonadaceae	0.007	0.040	8.70.E-01	9.96.E-01
Podoviridae	L5_Pseudomonadaceae	0.010	0.047	8.38.E-01	9.94.E-01
Siphoviridae	L5_Pseudomonadaceae	-0.011	0.042	7.93.E-01	9.92.E-01
Autographiviridae	L5_Burkholderiaceae	0.020	0.021	3.36.E-01	9.15.E-01
crAss_like_phage	L5_Burkholderiaceae	0.035	0.023	1.23.E-01	7.94.E-01
Herelleviridae	L5_Burkholderiaceae	-0.007	0.022	7.43.E-01	9.89.E-01
Microviridae	L5_Burkholderiaceae	-0.030	0.022	1.67.E-01	8.44.E-01
Myoviridae	L5_Burkholderiaceae	-0.010	0.024	6.69.E-01	9.80.E-01
Phycodnaviridae	L5_Burkholderiaceae	-0.056	0.021	8.02.E-03	4.75.E-01
Podoviridae	L5_Burkholderiaceae	-0.024	0.025	3.28.E-01	9.15.E-01
Siphoviridae	L5_Burkholderiaceae	-0.031	0.022	1.64.E-01	8.39.E-01
Autographiviridae	L5_Enterococcaceae	0.065	0.036	7.03.E-02	7.24.E-01
crAss_like_phage	L5_Enterococcaceae	-0.049	0.040	2.21.E-01	8.79.E-01
Herelleviridae	L5_Enterococcaceae	0.030	0.038	4.18.E-01	9.19.E-01
Microviridae	L5_Enterococcaceae	-0.016	0.038	6.80.E-01	9.80.E-01
Myoviridae	L5_Enterococcaceae	0.098	0.042	1.93.E-02	5.90.E-01
Phycodnaviridae	L5_Enterococcaceae	0.002	0.037	9.58.E-01	9.98.E-01
Podoviridae	L5_Enterococcaceae	0.047	0.043	2.77.E-01	9.05.E-01
Siphoviridae	L5_Enterococcaceae	-0.057	0.039	1.41.E-01	8.10.E-01
Autographiviridae	L6_Haemophilus	-0.009	0.033	7.86.E-01	9.92.E-01
crAss_like_phage	L6_Haemophilus	-0.044	0.036	2.21.E-01	8.79.E-01
Herelleviridae	L6_Haemophilus	0.070	0.034	3.83.E-02	7.03.E-01
Microviridae	L6_Haemophilus	0.016	0.034	6.46.E-01	9.79.E-01
Myoviridae	L6_Haemophilus	0.115	0.038	2.30.E-03	3.52.E-01
Phycodnaviridae	L6_Haemophilus	0.030	0.034	3.75.E-01	9.15.E-01
Podoviridae	L6_Haemophilus	0.008	0.039	8.38.E-01	9.94.E-01
Siphoviridae	L6_Haemophilus	-0.010	0.035	7.82.E-01	9.92.E-01

Autographiviridae	L6_Ralstonia	-0.001	0.014	9.69.E-01	9.98.E-01
crAss_like_phage	L6_Ralstonia	-0.010	0.015	5.31.E-01	9.57.E-01
Herelleviridae	L6_Ralstonia	0.005	0.014	7.52.E-01	9.89.E-01
Microviridae	L6_Ralstonia	0.005	0.014	7.43.E-01	9.89.E-01
Myoviridae	L6_Ralstonia	-0.005	0.016	7.69.E-01	9.91.E-01
Phycodnaviridae	L6_Ralstonia	-0.013	0.014	3.47.E-01	9.15.E-01
Podoviridae	L6_Ralstonia	-0.034	0.017	3.83.E-02	7.03.E-01
Siphoviridae	L6_Ralstonia	-0.017	0.015	2.41.E-01	8.85.E-01
Autographiviridae	L6_Helicobacter	-0.001	0.032	9.83.E-01	9.99.E-01
crAss_like_phage	L6_Helicobacter	-0.015	0.035	6.76.E-01	9.80.E-01
Herelleviridae	L6_Helicobacter	-0.004	0.033	8.99.E-01	9.97.E-01
Microviridae	L6_Helicobacter	-0.053	0.033	1.09.E-01	7.82.E-01
Myoviridae	L6_Helicobacter	-0.009	0.037	8.00.E-01	9.92.E-01
Phycodnaviridae	L6_Helicobacter	0.066	0.032	4.27.E-02	7.19.E-01
Podoviridae	L6_Helicobacter	-0.018	0.038	6.32.E-01	9.76.E-01
Siphoviridae	L6_Helicobacter	-0.003	0.034	9.24.E-01	9.98.E-01
Autographiviridae	L6_Alistipes	0.010	0.026	7.01.E-01	9.81.E-01
crAss_like_phage	L6_Alistipes	0.046	0.028	1.03.E-01	7.82.E-01
Herelleviridae	L6_Alistipes	0.026	0.027	3.29.E-01	9.15.E-01
Microviridae	L6_Alistipes	0.003	0.027	8.98.E-01	9.97.E-01
Myoviridae	L6_Alistipes	-0.020	0.030	4.98.E-01	9.55.E-01
Phycodnaviridae	L6_Alistipes	-0.020	0.026	4.42.E-01	9.32.E-01
Podoviridae	L6_Alistipes	0.065	0.031	3.43.E-02	6.83.E-01
Siphoviridae	L6_Alistipes	-0.014	0.027	6.05.E-01	9.73.E-01
Autographiviridae	L6_g_Clostridium_leptum	-0.004	0.036	9.10.E-01	9.98.E-01
crAss_like_phage	L6_g_Clostridium_leptum	0.070	0.040	7.90.E-02	7.43.E-01
Herelleviridae	L6_g_Clostridium_leptum	-0.078	0.037	3.62.E-02	6.90.E-01
Microviridae	L6_g_Clostridium_leptum	-0.053	0.037	1.60.E-01	8.38.E-01
Myoviridae	L6_g_Clostridium_leptum	-0.004	0.042	9.28.E-01	9.98.E-01
Phycodnaviridae	L6_g_Clostridium_leptum	-0.005	0.037	8.87.E-01	9.96.E-01
Podoviridae	L6_g_Clostridium_leptum	-0.006	0.043	8.98.E-01	9.97.E-01
Siphoviridae	L6_g_Clostridium_leptum	-0.011	0.038	7.77.E-01	9.92.E-01
Autographiviridae	L6_Acetivibrio	-0.032	0.039	4.10.E-01	9.17.E-01
crAss_like_phage	L6_Acetivibrio	0.011	0.043	7.95.E-01	9.92.E-01
Herelleviridae	L6_Acetivibrio	-0.115	0.040	4.37.E-03	4.24.E-01
Microviridae	L6_Acetivibrio	0.053	0.040	1.90.E-01	8.61.E-01
Myoviridae	L6_Acetivibrio	0.006	0.045	8.91.E-01	9.96.E-01
Phycodnaviridae	L6_Acetivibrio	-0.035	0.040	3.79.E-01	9.15.E-01
Podoviridae	L6_Acetivibrio	0.044	0.047	3.50.E-01	9.15.E-01
Siphoviridae	L6_Acetivibrio	-0.024	0.041	5.56.E-01	9.61.E-01
Autographiviridae	L6_Raoultella	0.036	0.027	1.84.E-01	8.60.E-01
crAss_like_phage	L6_Raoultella	0.050	0.030	9.19.E-02	7.59.E-01
Herelleviridae	L6_Raoultella	-0.034	0.028	2.21.E-01	8.79.E-01
Microviridae	L6_Raoultella	-0.057	0.028	4.43.E-02	7.22.E-01
Myoviridae	L6_Raoultella	-0.040	0.031	1.97.E-01	8.66.E-01
Phycodnaviridae	L6_Raoultella	-0.047	0.028	8.92.E-02	7.57.E-01
Podoviridae	L6_Raoultella	0.030	0.032	3.60.E-01	9.15.E-01
Siphoviridae	L6_Raoultella	0.008	0.029	7.72.E-01	9.92.E-01
Autographiviridae	L6_Synergistes	0.063	0.036	7.90.E-02	7.43.E-01
crAss_like_phage	L6_Synergistes	-0.011	0.040	7.87.E-01	9.92.E-01
Herelleviridae	L6_Synergistes	0.035	0.038	3.55.E-01	9.15.E-01
Microviridae	L6_Synergistes	0.031	0.038	4.15.E-01	9.18.E-01
Myoviridae	L6_Synergistes	-0.003	0.042	9.41.E-01	9.98.E-01
Phycodnaviridae	L6_Synergistes	-0.028	0.037	4.57.E-01	9.41.E-01
Podoviridae	L6_Synergistes	-0.004	0.043	9.24.E-01	9.98.E-01
Siphoviridae	L6_Synergistes	0.020	0.039	6.12.E-01	9.75.E-01
Autographiviridae	L6_Marvinbryantia	-0.008	0.038	8.27.E-01	9.93.E-01
crAss_like_phage	L6_Marvinbryantia	-0.023	0.043	5.85.E-01	9.65.E-01
Herelleviridae	L6_Marvinbryantia	-0.030	0.040	4.52.E-01	9.38.E-01
Microviridae	L6_Marvinbryantia	-0.078	0.040	5.02.E-02	7.22.E-01
Myoviridae	L6_Marvinbryantia	-0.007	0.045	8.73.E-01	9.96.E-01
Phycodnaviridae	L6_Marvinbryantia	0.013	0.040	7.39.E-01	9.88.E-01
Podoviridae	L6_Marvinbryantia	-0.072	0.046	1.17.E-01	7.86.E-01
Siphoviridae	L6_Marvinbryantia	-0.038	0.041	3.55.E-01	9.15.E-01
Autographiviridae	L6_Subdoligranulum	-0.015	0.026	5.68.E-01	9.61.E-01

crAss_like_phage	L6_Subdoligranulum	0.010	0.029	7.33.E-01	9.88.E-01
Herelleviridae	L6_Subdoligranulum	-0.025	0.027	3.63.E-01	9.15.E-01
Microviridae	L6_Subdoligranulum	-0.068	0.027	1.38.E-02	5.51.E-01
Myoviridae	L6_Subdoligranulum	-0.054	0.031	7.78.E-02	7.42.E-01
Phycodnaviridae	L6_Subdoligranulum	-0.054	0.027	4.81.E-02	7.22.E-01
Podoviridae	L6_Subdoligranulum	0.012	0.032	7.12.E-01	9.85.E-01
Siphoviridae	L6_Subdoligranulum	-0.019	0.028	5.00.E-01	9.55.E-01
Autographiviridae	L6_Odoribacter	-0.011	0.021	5.92.E-01	9.68.E-01
crAss_like_phage	L6_Odoribacter	0.027	0.023	2.40.E-01	8.85.E-01
Herelleviridae	L6_Odoribacter	0.006	0.022	7.66.E-01	9.91.E-01
Microviridae	L6_Odoribacter	0.018	0.022	4.06.E-01	9.17.E-01
Myoviridae	L6_Odoribacter	-0.026	0.024	2.86.E-01	9.15.E-01
Phycodnaviridae	L6_Odoribacter	0.050	0.021	1.97.E-02	5.90.E-01
Podoviridae	L6_Odoribacter	0.031	0.025	2.22.E-01	8.80.E-01
Siphoviridae	L6_Odoribacter	0.009	0.022	6.95.E-01	9.81.E-01
Autographiviridae	L6_g_bacterium_LF.3	0.004	0.039	9.22.E-01	9.98.E-01
crAss_like_phage	L6_g_bacterium_LF.3	-0.031	0.043	4.74.E-01	9.52.E-01
Herelleviridae	L6_g_bacterium_LF.3	0.009	0.040	8.27.E-01	9.93.E-01
Microviridae	L6_g_bacterium_LF.3	-0.006	0.040	8.84.E-01	9.96.E-01
Myoviridae	L6_g_bacterium_LF.3	0.000	0.045	9.97.E-01	9.99.E-01
Phycodnaviridae	L6_g_bacterium_LF.3	-0.025	0.040	5.26.E-01	9.57.E-01
Podoviridae	L6_g_bacterium_LF.3	-0.024	0.046	6.00.E-01	9.70.E-01
Siphoviridae	L6_g_bacterium_LF.3	-0.028	0.041	4.92.E-01	9.55.E-01
Autographiviridae	L6_Succinatimonas	-0.006	0.041	8.75.E-01	9.96.E-01
crAss_like_phage	L6_Succinatimonas	0.004	0.046	9.24.E-01	9.98.E-01
Herelleviridae	L6_Succinatimonas	-0.019	0.043	6.57.E-01	9.79.E-01
Microviridae	L6_Succinatimonas	0.014	0.043	7.48.E-01	9.89.E-01
Myoviridae	L6_Succinatimonas	-0.015	0.048	7.56.E-01	9.89.E-01
Phycodnaviridae	L6_Succinatimonas	0.045	0.042	2.86.E-01	9.15.E-01
Podoviridae	L6_Succinatimonas	-0.023	0.049	6.40.E-01	9.79.E-01
Siphoviridae	L6_Succinatimonas	0.008	0.044	8.59.E-01	9.96.E-01
Autographiviridae	L6_Anaerotruncus	-0.031	0.024	1.97.E-01	8.66.E-01
crAss_like_phage	L6_Anaerotruncus	0.016	0.027	5.45.E-01	9.60.E-01
Herelleviridae	L6_Anaerotruncus	-0.023	0.025	3.64.E-01	9.15.E-01
Microviridae	L6_Anaerotruncus	-0.001	0.025	9.62.E-01	9.98.E-01
Myoviridae	L6_Anaerotruncus	0.037	0.028	1.87.E-01	8.61.E-01
Phycodnaviridae	L6_Anaerotruncus	0.018	0.025	4.60.E-01	9.43.E-01
Podoviridae	L6_Anaerotruncus	0.027	0.029	3.46.E-01	9.15.E-01
Siphoviridae	L6_Anaerotruncus	0.024	0.026	3.62.E-01	9.15.E-01
Autographiviridae	L6_g_Eubacterium_sulci	0.010	0.035	7.81.E-01	9.92.E-01
crAss_like_phage	L6_g_Eubacterium_sulci	0.008	0.039	8.45.E-01	9.95.E-01
Herelleviridae	L6_g_Eubacterium_sulci	-0.046	0.037	2.08.E-01	8.76.E-01
Microviridae	L6_g_Eubacterium_sulci	0.033	0.037	3.65.E-01	9.15.E-01
Myoviridae	L6_g_Eubacterium_sulci	-0.027	0.041	5.15.E-01	9.57.E-01
Phycodnaviridae	L6_g_Eubacterium_sulci	-0.013	0.036	7.20.E-01	9.86.E-01
Podoviridae	L6_g_Eubacterium_sulci	0.010	0.042	8.15.E-01	9.93.E-01
Siphoviridae	L6_g_Eubacterium_sulci	-0.045	0.038	2.38.E-01	8.85.E-01
Autographiviridae	L6_Negativicoccus	0.015	0.039	6.97.E-01	9.81.E-01
crAss_like_phage	L6_Negativicoccus	-0.107	0.043	1.27.E-02	5.43.E-01
Herelleviridae	L6_Negativicoccus	0.008	0.040	8.38.E-01	9.94.E-01
Microviridae	L6_Negativicoccus	-0.055	0.040	1.73.E-01	8.55.E-01
Myoviridae	L6_Negativicoccus	0.046	0.045	3.08.E-01	9.15.E-01
Phycodnaviridae	L6_Negativicoccus	0.024	0.040	5.45.E-01	9.60.E-01
Podoviridae	L6_Negativicoccus	-0.048	0.047	3.05.E-01	9.15.E-01
Siphoviridae	L6_Negativicoccus	0.019	0.042	6.48.E-01	9.79.E-01
Autographiviridae	L6_Actinobaculum	0.024	0.038	5.25.E-01	9.57.E-01
crAss_like_phage	L6_Actinobaculum	0.033	0.042	4.43.E-01	9.32.E-01
Herelleviridae	L6_Actinobaculum	-0.042	0.040	2.99.E-01	9.15.E-01
Microviridae	L6_Actinobaculum	-0.038	0.040	3.41.E-01	9.15.E-01
Myoviridae	L6_Actinobaculum	-0.084	0.045	6.03.E-02	7.24.E-01
Phycodnaviridae	L6_Actinobaculum	0.048	0.039	2.23.E-01	8.80.E-01
Podoviridae	L6_Actinobaculum	0.050	0.046	2.83.E-01	9.13.E-01
Siphoviridae	L6_Actinobaculum	-0.026	0.041	5.26.E-01	9.57.E-01
Autographiviridae	L6_Coprobacillus	-0.006	0.041	8.84.E-01	9.96.E-01
crAss_like_phage	L6_Coprobacillus	-0.017	0.045	7.08.E-01	9.83.E-01

Herelleviridae	L6_Coprobacillus	0.005	0.043	9.03.E-01	9.97.E-01
Microviridae	L6_Coprobacillus	0.058	0.043	1.75.E-01	8.56.E-01
Myoviridae	L6_Coprobacillus	0.008	0.048	8.64.E-01	9.96.E-01
Phycodnaviridae	L6_Coprobacillus	-0.006	0.042	8.81.E-01	9.96.E-01
Podoviridae	L6_Coprobacillus	-0.022	0.049	6.57.E-01	9.79.E-01
Siphoviridae	L6_Coprobacillus	0.013	0.044	7.69.E-01	9.91.E-01
Autographiviridae	L6_Ruminococcus	0.068	0.032	3.67.E-02	6.90.E-01
crAss_like_phage	L6_Ruminococcus	0.029	0.036	4.27.E-01	9.24.E-01
Herelleviridae	L6_Ruminococcus	0.022	0.034	5.25.E-01	9.57.E-01
Microviridae	L6_Ruminococcus	-0.027	0.034	4.34.E-01	9.26.E-01
Myoviridae	L6_Ruminococcus	-0.055	0.038	1.49.E-01	8.22.E-01
Phycodnaviridae	L6_Ruminococcus	0.017	0.033	6.11.E-01	9.75.E-01
Podoviridae	L6_Ruminococcus	-0.054	0.039	1.66.E-01	8.44.E-01
Siphoviridae	L6_Ruminococcus	-0.051	0.035	1.42.E-01	8.12.E-01
Autographiviridae	L6_Paraclostridium	0.023	0.028	4.12.E-01	9.18.E-01
crAss_like_phage	L6_Paraclostridium	0.000	0.031	9.96.E-01	9.99.E-01
Herelleviridae	L6_Paraclostridium	0.014	0.029	6.17.E-01	9.75.E-01
Microviridae	L6_Paraclostridium	-0.039	0.029	1.81.E-01	8.60.E-01
Myoviridae	L6_Paraclostridium	-0.033	0.032	2.99.E-01	9.15.E-01
Phycodnaviridae	L6_Paraclostridium	0.030	0.028	2.95.E-01	9.15.E-01
Podoviridae	L6_Paraclostridium	0.032	0.033	3.32.E-01	9.15.E-01
Siphoviridae	L6_Paraclostridium	0.042	0.030	1.52.E-01	8.24.E-01
Autographiviridae	L6_g_Clostridium_hiranonis	-0.020	0.037	5.77.E-01	9.63.E-01
crAss_like_phage	L6_g_Clostridium_hiranonis	-0.002	0.041	9.55.E-01	9.98.E-01
Herelleviridae	L6_g_Clostridium_hiranonis	0.023	0.038	5.53.E-01	9.60.E-01
Microviridae	L6_g_Clostridium_hiranonis	0.063	0.038	9.85.E-02	7.78.E-01
Myoviridae	L6_g_Clostridium_hiranonis	-0.054	0.043	2.08.E-01	8.76.E-01
Phycodnaviridae	L6_g_Clostridium_hiranonis	0.026	0.038	4.99.E-01	9.55.E-01
Podoviridae	L6_g_Clostridium_hiranonis	-0.041	0.044	3.47.E-01	9.15.E-01
Siphoviridae	L6_g_Clostridium_hiranonis	0.081	0.039	3.88.E-02	7.03.E-01
Autographiviridae	L6_g_Proteobacteria_bacterium	-0.012	0.039	7.63.E-01	9.91.E-01
crAss_like_phage	L6_g_Proteobacteria_bacterium	0.030	0.043	4.86.E-01	9.54.E-01
Herelleviridae	L6_g_Proteobacteria_bacterium	0.046	0.040	2.55.E-01	8.95.E-01
Microviridae	L6_g_Proteobacteria_bacterium	-0.060	0.041	1.41.E-01	8.10.E-01
Myoviridae	L6_g_Proteobacteria_bacterium	0.012	0.045	7.83.E-01	9.92.E-01
Phycodnaviridae	L6_g_Proteobacteria_bacterium	0.008	0.040	8.51.E-01	9.95.E-01
Podoviridae	L6_g_Proteobacteria_bacterium	-0.126	0.046	6.84.E-03	4.51.E-01
Siphoviridae	L6_g_Proteobacteria_bacterium	-0.008	0.042	8.52.E-01	9.95.E-01
Autographiviridae	L6_g_Firmicutes_bacterium	-0.024	0.024	3.27.E-01	9.15.E-01
crAss_like_phage	L6_g_Firmicutes_bacterium	0.032	0.027	2.29.E-01	8.82.E-01
Herelleviridae	L6_g_Firmicutes_bacterium	0.001	0.025	9.83.E-01	9.99.E-01
Microviridae	L6_g_Firmicutes_bacterium	0.023	0.025	3.74.E-01	9.15.E-01
Myoviridae	L6_g_Firmicutes_bacterium	-0.019	0.028	4.98.E-01	9.55.E-01
Phycodnaviridae	L6_g_Firmicutes_bacterium	0.030	0.025	2.31.E-01	8.82.E-01
Podoviridae	L6_g_Firmicutes_bacterium	0.008	0.029	7.78.E-01	9.92.E-01
Siphoviridae	L6_g_Firmicutes_bacterium	0.007	0.026	7.90.E-01	9.92.E-01
Autographiviridae	L6_Providencia	0.033	0.034	3.30.E-01	9.15.E-01
crAss_like_phage	L6_Providencia	0.009	0.038	8.15.E-01	9.93.E-01
Herelleviridae	L6_Providencia	0.028	0.036	4.26.E-01	9.24.E-01
Microviridae	L6_Providencia	0.031	0.036	3.85.E-01	9.15.E-01
Myoviridae	L6_Providencia	0.010	0.040	8.11.E-01	9.93.E-01
Phycodnaviridae	L6_Providencia	0.047	0.035	1.77.E-01	8.58.E-01
Podoviridae	L6_Providencia	0.107	0.041	8.78.E-03	4.93.E-01
Siphoviridae	L6_Providencia	-0.052	0.036	1.51.E-01	8.22.E-01
Autographiviridae	L6_Veillonella	-0.002	0.033	9.56.E-01	9.98.E-01
crAss_like_phage	L6_Veillonella	-0.033	0.036	3.60.E-01	9.15.E-01
Herelleviridae	L6_Veillonella	-0.081	0.034	1.80.E-02	5.86.E-01
Microviridae	L6_Veillonella	-0.027	0.034	4.36.E-01	9.26.E-01
Myoviridae	L6_Veillonella	-0.071	0.038	6.42.E-02	7.24.E-01
Phycodnaviridae	L6_Veillonella	-0.041	0.034	2.22.E-01	8.80.E-01
Podoviridae	L6_Veillonella	-0.106	0.039	6.89.E-03	4.51.E-01
Siphoviridae	L6_Veillonella	-0.057	0.035	1.03.E-01	7.82.E-01
Autographiviridae	L6_g_Bacteroides_pectinophilus	-0.004	0.032	9.07.E-01	9.97.E-01
crAss_like_phage	L6_g_Bacteroides_pectinophilus	-0.050	0.036	1.59.E-01	8.38.E-01
Herelleviridae	L6_g_Bacteroides_pectinophilus	0.002	0.034	9.53.E-01	9.98.E-01

Microviridae	L6_g_Bacteroides_pectinophilus	-0.062	0.034	6.46.E-02	7.24.E-01
Myoviridae	L6_g_Bacteroides_pectinophilus	-0.030	0.038	4.29.E-01	9.25.E-01
Phycodnaviridae	L6_g_Bacteroides_pectinophilus	-0.017	0.033	6.05.E-01	9.73.E-01
Podoviridae	L6_g_Bacteroides_pectinophilus	-0.039	0.039	3.20.E-01	9.15.E-01
Siphoviridae	L6_g_Bacteroides_pectinophilus	-0.075	0.034	2.99.E-02	6.66.E-01
Autographiviridae	L6_g_Erysipelotrichaceae_bacterium	0.023	0.033	4.77.E-01	9.52.E-01
crAss_like_phage	L6_g_Erysipelotrichaceae_bacterium	-0.008	0.036	8.23.E-01	9.93.E-01
Herelleviridae	L6_g_Erysipelotrichaceae_bacterium	-0.033	0.034	3.30.E-01	9.15.E-01
Microviridae	L6_g_Erysipelotrichaceae_bacterium	0.027	0.034	4.26.E-01	9.24.E-01
Myoviridae	L6_g_Erysipelotrichaceae_bacterium	-0.004	0.038	9.06.E-01	9.97.E-01
Phycodnaviridae	L6_g_Erysipelotrichaceae_bacterium	0.003	0.033	9.33.E-01	9.98.E-01
Podoviridae	L6_g_Erysipelotrichaceae_bacterium	-0.063	0.039	1.06.E-01	7.82.E-01
Siphoviridae	L6_g_Erysipelotrichaceae_bacterium	0.016	0.035	6.55.E-01	9.79.E-01
Autographiviridae	L6_Anaerovorax	0.036	0.038	3.41.E-01	9.15.E-01
crAss_like_phage	L6_Anaerovorax	-0.039	0.042	3.54.E-01	9.15.E-01
Herelleviridae	L6_Anaerovorax	0.019	0.039	6.33.E-01	9.76.E-01
Microviridae	L6_Anaerovorax	-0.019	0.039	6.34.E-01	9.76.E-01
Myoviridae	L6_Anaerovorax	-0.021	0.044	6.37.E-01	9.77.E-01
Phycodnaviridae	L6_Anaerovorax	0.045	0.039	2.47.E-01	8.92.E-01
Podoviridae	L6_Anaerovorax	-0.022	0.045	6.30.E-01	9.76.E-01
Siphoviridae	L6_Anaerovorax	0.053	0.040	1.93.E-01	8.61.E-01
Autographiviridae	L6_Anaeroglobus	0.079	0.041	5.67.E-02	7.24.E-01
crAss_like_phage	L6_Anaeroglobus	0.012	0.046	7.92.E-01	9.92.E-01
Herelleviridae	L6_Anaeroglobus	-0.053	0.043	2.20.E-01	8.79.E-01
Microviridae	L6_Anaeroglobus	-0.080	0.043	6.41.E-02	7.24.E-01
Myoviridae	L6_Anaeroglobus	-0.047	0.048	3.27.E-01	9.15.E-01
Phycodnaviridae	L6_Anaeroglobus	-0.001	0.043	9.74.E-01	9.99.E-01
Podoviridae	L6_Anaeroglobus	0.017	0.050	7.32.E-01	9.88.E-01
Siphoviridae	L6_Anaeroglobus	-0.014	0.044	7.51.E-01	9.89.E-01
Autographiviridae	L6_Romboutsia	0.010	0.028	7.26.E-01	9.87.E-01
crAss_like_phage	L6_Romboutsia	0.023	0.031	4.50.E-01	9.37.E-01
Herelleviridae	L6_Romboutsia	0.043	0.029	1.35.E-01	8.06.E-01
Microviridae	L6_Romboutsia	-0.042	0.029	1.42.E-01	8.12.E-01
Myoviridae	L6_Romboutsia	-0.051	0.032	1.13.E-01	7.84.E-01
Phycodnaviridae	L6_Romboutsia	-0.021	0.028	4.66.E-01	9.46.E-01
Podoviridae	L6_Romboutsia	0.008	0.033	8.17.E-01	9.93.E-01
Siphoviridae	L6_Romboutsia	0.001	0.030	9.68.E-01	9.98.E-01
Autographiviridae	L6_Collinsella	0.016	0.034	6.30.E-01	9.76.E-01
crAss_like_phage	L6_Collinsella	-0.041	0.038	2.73.E-01	9.04.E-01
Herelleviridae	L6_Collinsella	0.033	0.035	3.55.E-01	9.15.E-01
Microviridae	L6_Collinsella	-0.034	0.036	3.40.E-01	9.15.E-01
Myoviridae	L6_Collinsella	-0.034	0.040	3.87.E-01	9.15.E-01
Phycodnaviridae	L6_Collinsella	0.032	0.035	3.60.E-01	9.15.E-01
Podoviridae	L6_Collinsella	-0.037	0.041	3.73.E-01	9.15.E-01
Siphoviridae	L6_Collinsella	-0.033	0.036	3.60.E-01	9.15.E-01
Autographiviridae	L6_g_Peptostreptococcaceae_bacterium	0.025	0.028	3.89.E-01	9.15.E-01
crAss_like_phage	L6_g_Peptostreptococcaceae_bacterium	0.005	0.032	8.66.E-01	9.96.E-01
Herelleviridae	L6_g_Peptostreptococcaceae_bacterium	0.047	0.030	1.15.E-01	7.85.E-01
Microviridae	L6_g_Peptostreptococcaceae_bacterium	-0.051	0.030	8.49.E-02	7.50.E-01
Myoviridae	L6_g_Peptostreptococcaceae_bacterium	-0.039	0.033	2.35.E-01	8.85.E-01
Phycodnaviridae	L6_g_Peptostreptococcaceae_bacterium	0.005	0.029	8.74.E-01	9.96.E-01
Podoviridae	L6_g_Peptostreptococcaceae_bacterium	0.053	0.034	1.24.E-01	7.94.E-01
Siphoviridae	L6_g_Peptostreptococcaceae_bacterium	0.019	0.030	5.30.E-01	9.57.E-01
Autographiviridae	L6_Aeromicrobium	0.003	0.032	9.17.E-01	9.98.E-01
crAss_like_phage	L6_Aeromicrobium	0.013	0.035	7.01.E-01	9.81.E-01
Herelleviridae	L6_Aeromicrobium	0.062	0.033	5.95.E-02	7.24.E-01
Microviridae	L6_Aeromicrobium	0.015	0.033	6.45.E-01	9.79.E-01
Myoviridae	L6_Aeromicrobium	-0.090	0.037	1.37.E-02	5.51.E-01
Phycodnaviridae	L6_Aeromicrobium	-0.005	0.033	8.78.E-01	9.96.E-01
Podoviridae	L6_Aeromicrobium	0.077	0.038	4.13.E-02	7.09.E-01
Siphoviridae	L6_Aeromicrobium	0.021	0.034	5.31.E-01	9.57.E-01
Autographiviridae	L6_Terrisporobacter	0.026	0.029	3.66.E-01	9.15.E-01
crAss_like_phage	L6_Terrisporobacter	-0.003	0.032	9.14.E-01	9.98.E-01
Herelleviridae	L6_Terrisporobacter	0.043	0.030	1.47.E-01	8.16.E-01
Microviridae	L6_Terrisporobacter	-0.099	0.030	8.69.E-04	2.79.E-01

Myoviridae	L6_Terrisporobacter	-0.013	0.033	6.91.E-01	9.80.E-01
Phycodnaviridae	L6_Terrisporobacter	0.037	0.030	2.10.E-01	8.76.E-01
Podoviridae	L6_Terrisporobacter	0.047	0.034	1.73.E-01	8.55.E-01
Siphoviridae	L6_Terrisporobacter	0.005	0.031	8.62.E-01	9.96.E-01
Autographiviridae	L6_Christensenella	0.013	0.031	6.75.E-01	9.80.E-01
crAss_like_phage	L6_Christensenella	-0.034	0.034	3.24.E-01	9.15.E-01
Herelleviridae	L6_Christensenella	-0.055	0.032	8.73.E-02	7.52.E-01
Microviridae	L6_Christensenella	0.018	0.032	5.70.E-01	9.61.E-01
Myoviridae	L6_Christensenella	0.017	0.036	6.43.E-01	9.79.E-01
Phycodnaviridae	L6_Christensenella	-0.069	0.032	3.03.E-02	6.66.E-01
Podoviridae	L6_Christensenella	0.009	0.037	8.07.E-01	9.93.E-01
Siphoviridae	L6_Christensenella	-0.010	0.033	7.51.E-01	9.89.E-01
Autographiviridae	L6_Aggregatibacter	-0.009	0.033	7.89.E-01	9.92.E-01
crAss_like_phage	L6_Aggregatibacter	-0.019	0.036	5.93.E-01	9.69.E-01
Herelleviridae	L6_Aggregatibacter	0.064	0.034	6.22.E-02	7.24.E-01
Microviridae	L6_Aggregatibacter	0.002	0.034	9.44.E-01	9.98.E-01
Myoviridae	L6_Aggregatibacter	0.092	0.038	1.57.E-02	5.77.E-01
Phycodnaviridae	L6_Aggregatibacter	-0.027	0.034	4.29.E-01	9.25.E-01
Podoviridae	L6_Aggregatibacter	0.011	0.039	7.75.E-01	9.92.E-01
Siphoviridae	L6_Aggregatibacter	-0.011	0.035	7.64.E-01	9.91.E-01
Autographiviridae	L6_g_Lachnospiraceae_bacterium	-0.001	0.035	9.68.E-01	9.98.E-01
crAss_like_phage	L6_g_Lachnospiraceae_bacterium	-0.038	0.038	3.21.E-01	9.15.E-01
Herelleviridae	L6_g_Lachnospiraceae_bacterium	0.026	0.036	4.65.E-01	9.45.E-01
Microviridae	L6_g_Lachnospiraceae_bacterium	-0.005	0.036	8.85.E-01	9.96.E-01
Myoviridae	L6_g_Lachnospiraceae_bacterium	-0.032	0.040	4.24.E-01	9.23.E-01
Phycodnaviridae	L6_g_Lachnospiraceae_bacterium	0.069	0.035	5.30.E-02	7.22.E-01
Podoviridae	L6_g_Lachnospiraceae_bacterium	0.002	0.041	9.53.E-01	9.98.E-01
Siphoviridae	L6_g_Lachnospiraceae_bacterium	0.034	0.037	3.52.E-01	9.15.E-01
Autographiviridae	L6_Lachnospira	-0.007	0.034	8.38.E-01	9.94.E-01
crAss_like_phage	L6_Lachnospira	-0.011	0.038	7.79.E-01	9.92.E-01
Herelleviridae	L6_Lachnospira	0.018	0.036	6.12.E-01	9.75.E-01
Microviridae	L6_Lachnospira	-0.019	0.036	5.99.E-01	9.70.E-01
Myoviridae	L6_Lachnospira	-0.052	0.040	1.92.E-01	8.61.E-01
Phycodnaviridae	L6_Lachnospira	-0.025	0.035	4.83.E-01	9.54.E-01
Podoviridae	L6_Lachnospira	-0.035	0.041	4.00.E-01	9.15.E-01
Siphoviridae	L6_Lachnospira	-0.007	0.037	8.45.E-01	9.95.E-01
Autographiviridae	L6_Ruminiclostridium	0.007	0.029	8.15.E-01	9.93.E-01
crAss_like_phage	L6_Ruminiclostridium	0.031	0.032	3.30.E-01	9.15.E-01
Herelleviridae	L6_Ruminiclostridium	-0.043	0.030	1.44.E-01	8.12.E-01
Microviridae	L6_Ruminiclostridium	-0.011	0.030	7.16.E-01	9.86.E-01
Myoviridae	L6_Ruminiclostridium	0.036	0.033	2.72.E-01	9.02.E-01
Phycodnaviridae	L6_Ruminiclostridium	0.022	0.029	4.46.E-01	9.34.E-01
Podoviridae	L6_Ruminiclostridium	-0.022	0.034	5.27.E-01	9.57.E-01
Siphoviridae	L6_Ruminiclostridium	0.002	0.031	9.47.E-01	9.98.E-01
Autographiviridae	L6_Oribacterium	-0.018	0.039	6.53.E-01	9.79.E-01
crAss_like_phage	L6_Oribacterium	-0.018	0.043	6.81.E-01	9.80.E-01
Herelleviridae	L6_Oribacterium	-0.051	0.041	2.07.E-01	8.76.E-01
Microviridae	L6_Oribacterium	0.086	0.040	3.41.E-02	6.82.E-01
Myoviridae	L6_Oribacterium	0.078	0.045	8.54.E-02	7.50.E-01
Phycodnaviridae	L6_Oribacterium	-0.052	0.040	1.99.E-01	8.69.E-01
Podoviridae	L6_Oribacterium	0.089	0.047	5.76.E-02	7.24.E-01
Siphoviridae	L6_Oribacterium	0.000	0.042	9.95.E-01	9.99.E-01
Autographiviridae	L6_Fusobacterium	-0.038	0.037	3.12.E-01	9.15.E-01
crAss_like_phage	L6_Fusobacterium	-0.091	0.041	2.79.E-02	6.58.E-01
Herelleviridae	L6_Fusobacterium	0.040	0.039	3.00.E-01	9.15.E-01
Microviridae	L6_Fusobacterium	0.001	0.039	9.71.E-01	9.99.E-01
Myoviridae	L6_Fusobacterium	0.069	0.043	1.11.E-01	7.82.E-01
Phycodnaviridae	L6_Fusobacterium	0.046	0.038	2.28.E-01	8.82.E-01
Podoviridae	L6_Fusobacterium	0.111	0.045	1.34.E-02	5.51.E-01
Siphoviridae	L6_Fusobacterium	-0.021	0.040	6.05.E-01	9.73.E-01
Autographiviridae	L6_Fenollaria	0.031	0.042	4.61.E-01	9.43.E-01
crAss_like_phage	L6_Fenollaria	-0.002	0.046	9.63.E-01	9.98.E-01
Herelleviridae	L6_Fenollaria	0.084	0.043	5.12.E-02	7.22.E-01
Microviridae	L6_Fenollaria	0.021	0.043	6.28.E-01	9.76.E-01
Myoviridae	L6_Fenollaria	-0.050	0.048	2.96.E-01	9.15.E-01

Phycodnaviridae	L6_Fenollaria	-0.041	0.043	3.40.E-01	9.15.E-01
Podoviridae	L6_Fenollaria	-0.013	0.050	7.95.E-01	9.92.E-01
Siphoviridae	L6_Fenollaria	0.031	0.044	4.82.E-01	9.53.E-01
Autographiviridae	L6_Porphyromonas	0.015	0.030	6.30.E-01	9.76.E-01
crAss_like_phage	L6_Porphyromonas	0.041	0.034	2.21.E-01	8.79.E-01
Herelleviridae	L6_Porphyromonas	0.028	0.032	3.67.E-01	9.15.E-01
Microviridae	L6_Porphyromonas	-0.013	0.032	6.84.E-01	9.80.E-01
Myoviridae	L6_Porphyromonas	-0.070	0.035	4.60.E-02	7.22.E-01
Phycodnaviridae	L6_Porphyromonas	-0.005	0.031	8.66.E-01	9.96.E-01
Podoviridae	L6_Porphyromonas	-0.001	0.036	9.69.E-01	9.98.E-01
Siphoviridae	L6_Porphyromonas	0.015	0.032	6.35.E-01	9.76.E-01
Autographiviridae	L6_Gabonia	-0.011	0.037	7.67.E-01	9.91.E-01
crAss_like_phage	L6_Gabonia	0.015	0.041	7.18.E-01	9.86.E-01
Herelleviridae	L6_Gabonia	0.075	0.039	5.36.E-02	7.22.E-01
Microviridae	L6_Gabonia	-0.027	0.039	4.97.E-01	9.55.E-01
Myoviridae	L6_Gabonia	0.007	0.044	8.66.E-01	9.96.E-01
Phycodnaviridae	L6_Gabonia	0.003	0.038	9.46.E-01	9.98.E-01
Podoviridae	L6_Gabonia	0.003	0.045	9.52.E-01	9.98.E-01
Siphoviridae	L6_Gabonia	-0.004	0.040	9.13.E-01	9.98.E-01
Autographiviridae	L6_Citrobacter	-0.012	0.027	6.41.E-01	9.79.E-01
crAss_like_phage	L6_Citrobacter	0.015	0.029	5.99.E-01	9.70.E-01
Herelleviridae	L6_Citrobacter	0.023	0.028	4.15.E-01	9.18.E-01
Microviridae	L6_Citrobacter	-0.009	0.028	7.48.E-01	9.89.E-01
Myoviridae	L6_Citrobacter	-0.027	0.031	3.82.E-01	9.15.E-01
Phycodnaviridae	L6_Citrobacter	-0.013	0.027	6.30.E-01	9.76.E-01
Podoviridae	L6_Citrobacter	-0.004	0.032	8.95.E-01	9.97.E-01
Siphoviridae	L6_Citrobacter	0.016	0.028	5.85.E-01	9.65.E-01
Autographiviridae	L6_Sutterella	0.055	0.038	1.46.E-01	8.16.E-01
crAss_like_phage	L6_Sutterella	0.037	0.042	3.78.E-01	9.15.E-01
Herelleviridae	L6_Sutterella	-0.064	0.039	1.02.E-01	7.81.E-01
Microviridae	L6_Sutterella	0.005	0.040	8.95.E-01	9.97.E-01
Myoviridae	L6_Sutterella	0.022	0.044	6.24.E-01	9.76.E-01
Phycodnaviridae	L6_Sutterella	-0.005	0.039	8.88.E-01	9.96.E-01
Podoviridae	L6_Sutterella	0.030	0.046	5.15.E-01	9.57.E-01
Siphoviridae	L6_Sutterella	0.018	0.041	6.64.E-01	9.79.E-01
Autographiviridae	L6_Lachnoclostridium	-0.001	0.030	9.73.E-01	9.99.E-01
crAss_like_phage	L6_Lachnoclostridium	0.005	0.033	8.84.E-01	9.96.E-01
Herelleviridae	L6_Lachnoclostridium	0.024	0.031	4.41.E-01	9.32.E-01
Microviridae	L6_Lachnoclostridium	0.026	0.031	4.02.E-01	9.16.E-01
Myoviridae	L6_Lachnoclostridium	-0.019	0.035	5.93.E-01	9.69.E-01
Phycodnaviridae	L6_Lachnoclostridium	0.093	0.031	2.46.E-03	3.52.E-01
Podoviridae	L6_Lachnoclostridium	0.064	0.036	7.38.E-02	7.27.E-01
Siphoviridae	L6_Lachnoclostridium	0.041	0.032	2.05.E-01	8.76.E-01
Autographiviridae	L6_Parvimonas	-0.013	0.040	7.36.E-01	9.88.E-01
crAss_like_phage	L6_Parvimonas	-0.012	0.044	7.81.E-01	9.92.E-01
Herelleviridae	L6_Parvimonas	-0.052	0.041	2.10.E-01	8.76.E-01
Microviridae	L6_Parvimonas	0.109	0.041	8.36.E-03	4.75.E-01
Myoviridae	L6_Parvimonas	0.026	0.046	5.73.E-01	9.61.E-01
Phycodnaviridae	L6_Parvimonas	0.052	0.041	2.01.E-01	8.74.E-01
Podoviridae	L6_Parvimonas	0.067	0.048	1.62.E-01	8.38.E-01
Siphoviridae	L6_Parvimonas	-0.010	0.042	8.23.E-01	9.93.E-01
Autographiviridae	L6_Anaerostipes	-0.086	0.038	2.35.E-02	6.28.E-01
crAss_like_phage	L6_Anaerostipes	-0.100	0.042	1.70.E-02	5.86.E-01
Herelleviridae	L6_Anaerostipes	0.043	0.039	2.74.E-01	9.04.E-01
Microviridae	L6_Anaerostipes	-0.065	0.039	1.00.E-01	7.79.E-01
Myoviridae	L6_Anaerostipes	-0.063	0.044	1.54.E-01	8.26.E-01
Phycodnaviridae	L6_Anaerostipes	0.067	0.039	8.32.E-02	7.50.E-01
Podoviridae	L6_Anaerostipes	-0.058	0.045	1.99.E-01	8.69.E-01
Siphoviridae	L6_Anaerostipes	-0.036	0.041	3.76.E-01	9.15.E-01
Autographiviridae	L6_Peptoniphilus	0.034	0.038	3.81.E-01	9.15.E-01
crAss_like_phage	L6_Peptoniphilus	0.000	0.043	9.98.E-01	9.99.E-01
Herelleviridae	L6_Peptoniphilus	0.058	0.040	1.46.E-01	8.15.E-01
Microviridae	L6_Peptoniphilus	0.028	0.040	4.81.E-01	9.53.E-01
Myoviridae	L6_Peptoniphilus	-0.035	0.045	4.32.E-01	9.25.E-01
Phycodnaviridae	L6_Peptoniphilus	-0.017	0.040	6.65.E-01	9.79.E-01

Podoviridae	L6_Peptoniphilus	-0.006	0.046	9.00.E-01	9.97.E-01
Siphoviridae	L6_Peptoniphilus	0.008	0.041	8.55.E-01	9.96.E-01
Autographiviridae	L6_Staphylococcus	-0.013	0.040	7.39.E-01	9.88.E-01
crAss_like_phage	L6_Staphylococcus	-0.063	0.044	1.53.E-01	8.24.E-01
Herelleviridae	L6_Staphylococcus	-0.020	0.041	6.26.E-01	9.76.E-01
Microviridae	L6_Staphylococcus	-0.015	0.042	7.20.E-01	9.86.E-01
Myoviridae	L6_Staphylococcus	-0.025	0.046	5.93.E-01	9.69.E-01
Phycodnaviridae	L6_Staphylococcus	-0.026	0.041	5.30.E-01	9.57.E-01
Podoviridae	L6_Staphylococcus	-0.026	0.048	5.81.E-01	9.64.E-01
Siphoviridae	L6_Staphylococcus	-0.050	0.043	2.40.E-01	8.85.E-01
Autographiviridae	L6_Drancourtella	0.070	0.036	5.73.E-02	7.24.E-01
crAss_like_phage	L6_Drancourtella	0.017	0.040	6.80.E-01	9.80.E-01
Herelleviridae	L6_Drancourtella	-0.002	0.038	9.61.E-01	9.98.E-01
Microviridae	L6_Drancourtella	0.016	0.038	6.83.E-01	9.80.E-01
Myoviridae	L6_Drancourtella	0.132	0.042	1.77.E-03	3.25.E-01
Phycodnaviridae	L6_Drancourtella	-0.036	0.038	3.38.E-01	9.15.E-01
Podoviridae	L6_Drancourtella	-0.028	0.044	5.30.E-01	9.57.E-01
Siphoviridae	L6_Drancourtella	0.012	0.039	7.60.E-01	9.91.E-01
Autographiviridae	L6_Lachnotalea	-0.020	0.026	4.52.E-01	9.38.E-01
crAss_like_phage	L6_Lachnotalea	0.009	0.029	7.55.E-01	9.89.E-01
Herelleviridae	L6_Lachnotalea	0.039	0.027	1.49.E-01	8.22.E-01
Microviridae	L6_Lachnotalea	0.041	0.027	1.30.E-01	7.99.E-01
Myoviridae	L6_Lachnotalea	-0.004	0.030	8.87.E-01	9.96.E-01
Phycodnaviridae	L6_Lachnotalea	-0.023	0.027	4.01.E-01	9.15.E-01
Podoviridae	L6_Lachnotalea	-0.006	0.031	8.38.E-01	9.94.E-01
Siphoviridae	L6_Lachnotalea	-0.015	0.028	6.02.E-01	9.71.E-01
Autographiviridae	L6_g_Intestinimonas_massiliensis	-0.004	0.027	8.74.E-01	9.96.E-01
crAss_like_phage	L6_g_Intestinimonas_massiliensis	0.010	0.029	7.24.E-01	9.86.E-01
Herelleviridae	L6_g_Intestinimonas_massiliensis	0.004	0.028	8.95.E-01	9.97.E-01
Microviridae	L6_g_Intestinimonas_massiliensis	0.043	0.028	1.23.E-01	7.94.E-01
Myoviridae	L6_g_Intestinimonas_massiliensis	0.069	0.031	2.56.E-02	6.50.E-01
Phycodnaviridae	L6_g_Intestinimonas_massiliensis	0.021	0.027	4.35.E-01	9.26.E-01
Podoviridae	L6_g_Intestinimonas_massiliensis	0.007	0.032	8.20.E-01	9.93.E-01
Siphoviridae	L6_g_Intestinimonas_massiliensis	0.005	0.028	8.56.E-01	9.96.E-01
Autographiviridae	L6_Pediococcus	0.005	0.039	9.07.E-01	9.97.E-01
crAss_like_phage	L6_Pediococcus	0.013	0.043	7.70.E-01	9.91.E-01
Herelleviridae	L6_Pediococcus	0.028	0.040	4.94.E-01	9.55.E-01
Microviridae	L6_Pediococcus	0.039	0.040	3.33.E-01	9.15.E-01
Myoviridae	L6_Pediococcus	-0.037	0.045	4.13.E-01	9.18.E-01
Phycodnaviridae	L6_Pediococcus	0.074	0.040	6.27.E-02	7.24.E-01
Podoviridae	L6_Pediococcus	0.015	0.046	7.50.E-01	9.89.E-01
Siphoviridae	L6_Pediococcus	0.097	0.041	1.87.E-02	5.90.E-01
Autographiviridae	L6_Enterobacter	-0.036	0.022	1.01.E-01	7.79.E-01
crAss_like_phage	L6_Enterobacter	0.041	0.024	9.32.E-02	7.60.E-01
Herelleviridae	L6_Enterobacter	0.006	0.023	7.85.E-01	9.92.E-01
Microviridae	L6_Enterobacter	0.031	0.023	1.74.E-01	8.56.E-01
Myoviridae	L6_Enterobacter	-0.010	0.026	7.09.E-01	9.84.E-01
Phycodnaviridae	L6_Enterobacter	-0.030	0.023	1.92.E-01	8.61.E-01
Podoviridae	L6_Enterobacter	0.004	0.027	8.79.E-01	9.96.E-01
Siphoviridae	L6_Enterobacter	0.012	0.024	6.15.E-01	9.75.E-01
Autographiviridae	L6_Dielma	-0.068	0.038	7.15.E-02	7.24.E-01
crAss_like_phage	L6_Dielma	0.026	0.042	5.26.E-01	9.57.E-01
Herelleviridae	L6_Dielma	0.011	0.039	7.84.E-01	9.92.E-01
Microviridae	L6_Dielma	0.008	0.039	8.43.E-01	9.95.E-01
Myoviridae	L6_Dielma	0.015	0.044	7.40.E-01	9.88.E-01
Phycodnaviridae	L6_Dielma	0.070	0.039	7.01.E-02	7.24.E-01
Podoviridae	L6_Dielma	0.048	0.045	2.90.E-01	9.15.E-01
Siphoviridae	L6_Dielma	0.089	0.040	2.75.E-02	6.58.E-01
Autographiviridae	L6_Mediterraneibacter	-0.007	0.039	8.53.E-01	9.95.E-01
crAss_like_phage	L6_Mediterraneibacter	0.057	0.043	1.89.E-01	8.61.E-01
Herelleviridae	L6_Mediterraneibacter	0.063	0.041	1.24.E-01	7.94.E-01
Microviridae	L6_Mediterraneibacter	0.002	0.041	9.63.E-01	9.98.E-01
Myoviridae	L6_Mediterraneibacter	-0.004	0.046	9.33.E-01	9.98.E-01
Phycodnaviridae	L6_Mediterraneibacter	-0.014	0.040	7.33.E-01	9.88.E-01
Podoviridae	L6_Mediterraneibacter	-0.021	0.047	6.58.E-01	9.79.E-01

Siphoviridae	L6_Mediterraneibacter	0.127	0.042	2.41.E-03	3.52.E-01
Autographiviridae	L6_g_Eubacterium_rectale	0.013	0.035	7.15.E-01	9.86.E-01
crAss_like_phage	L6_g_Eubacterium_rectale	-0.023	0.039	5.53.E-01	9.60.E-01
Herelleviridae	L6_g_Eubacterium_rectale	-0.006	0.037	8.72.E-01	9.96.E-01
Microviridae	L6_g_Eubacterium_rectale	-0.052	0.037	1.61.E-01	8.38.E-01
Myoviridae	L6_g_Eubacterium_rectale	-0.009	0.041	8.18.E-01	9.93.E-01
Phycodnaviridae	L6_g_Eubacterium_rectale	-0.015	0.036	6.74.E-01	9.80.E-01
Podoviridae	L6_g_Eubacterium_rectale	-0.026	0.043	5.34.E-01	9.59.E-01
Siphoviridae	L6_g_Eubacterium_rectale	-0.067	0.038	7.61.E-02	7.32.E-01
Autographiviridae	L6_Alloprevotella	0.017	0.037	6.48.E-01	9.79.E-01
crAss_like_phage	L6_Alloprevotella	-0.053	0.041	1.98.E-01	8.68.E-01
Herelleviridae	L6_Alloprevotella	-0.061	0.038	1.09.E-01	7.82.E-01
Microviridae	L6_Alloprevotella	0.004	0.038	9.27.E-01	9.98.E-01
Myoviridae	L6_Alloprevotella	-0.084	0.043	4.99.E-02	7.22.E-01
Phycodnaviridae	L6_Alloprevotella	-0.014	0.038	7.17.E-01	9.86.E-01
Podoviridae	L6_Alloprevotella	-0.053	0.044	2.36.E-01	8.85.E-01
Siphoviridae	L6_Alloprevotella	-0.114	0.039	3.65.E-03	4.02.E-01
Autographiviridae	L6_g_Clostridiaceae_bacterium	0.008	0.027	7.60.E-01	9.91.E-01
crAss_like_phage	L6_g_Clostridiaceae_bacterium	-0.017	0.030	5.64.E-01	9.61.E-01
Herelleviridae	L6_g_Clostridiaceae_bacterium	-0.015	0.028	6.03.E-01	9.72.E-01
Microviridae	L6_g_Clostridiaceae_bacterium	0.010	0.028	7.35.E-01	9.88.E-01
Myoviridae	L6_g_Clostridiaceae_bacterium	0.029	0.032	3.59.E-01	9.15.E-01
Phycodnaviridae	L6_g_Clostridiaceae_bacterium	-0.059	0.028	3.37.E-02	6.82.E-01
Podoviridae	L6_g_Clostridiaceae_bacterium	-0.024	0.033	4.55.E-01	9.39.E-01
Siphoviridae	L6_g_Clostridiaceae_bacterium	0.028	0.029	3.41.E-01	9.15.E-01
Autographiviridae	L6_Paraprevotella	0.018	0.035	6.18.E-01	9.75.E-01
crAss_like_phage	L6_Paraprevotella	-0.041	0.039	2.98.E-01	9.15.E-01
Herelleviridae	L6_Paraprevotella	-0.025	0.037	4.99.E-01	9.55.E-01
Microviridae	L6_Paraprevotella	-0.048	0.037	1.94.E-01	8.62.E-01
Myoviridae	L6_Paraprevotella	0.052	0.041	2.04.E-01	8.76.E-01
Phycodnaviridae	L6_Paraprevotella	-0.024	0.036	5.02.E-01	9.55.E-01
Podoviridae	L6_Paraprevotella	0.035	0.042	4.05.E-01	9.17.E-01
Siphoviridae	L6_Paraprevotella	0.057	0.038	1.27.E-01	7.99.E-01
Autographiviridae	L6_Olsenella	0.019	0.038	6.14.E-01	9.75.E-01
crAss_like_phage	L6_Olsenella	-0.046	0.042	2.73.E-01	9.03.E-01
Herelleviridae	L6_Olsenella	-0.037	0.040	3.47.E-01	9.15.E-01
Microviridae	L6_Olsenella	-0.049	0.040	2.21.E-01	8.79.E-01
Myoviridae	L6_Olsenella	0.027	0.044	5.40.E-01	9.60.E-01
Phycodnaviridae	L6_Olsenella	-0.051	0.039	1.92.E-01	8.61.E-01
Podoviridae	L6_Olsenella	-0.097	0.046	3.41.E-02	6.82.E-01
Siphoviridae	L6_Olsenella	-0.026	0.041	5.30.E-01	9.57.E-01
Autographiviridae	L6_Intestinibacter	0.002	0.039	9.58.E-01	9.98.E-01
crAss_like_phage	L6_Intestinibacter	-0.083	0.043	5.32.E-02	7.22.E-01
Herelleviridae	L6_Intestinibacter	0.027	0.040	5.08.E-01	9.57.E-01
Microviridae	L6_Intestinibacter	-0.030	0.040	4.65.E-01	9.45.E-01
Myoviridae	L6_Intestinibacter	0.030	0.045	5.10.E-01	9.57.E-01
Phycodnaviridae	L6_Intestinibacter	0.028	0.040	4.89.E-01	9.55.E-01
Podoviridae	L6_Intestinibacter	-0.004	0.046	9.32.E-01	9.98.E-01
Siphoviridae	L6_Intestinibacter	0.013	0.041	7.62.E-01	9.91.E-01
Autographiviridae	L6_Johnsonella	-0.042	0.035	2.41.E-01	8.85.E-01
crAss_like_phage	L6_Johnsonella	0.058	0.039	1.41.E-01	8.10.E-01
Herelleviridae	L6_Johnsonella	-0.054	0.037	1.38.E-01	8.10.E-01
Microviridae	L6_Johnsonella	0.037	0.037	3.19.E-01	9.15.E-01
Myoviridae	L6_Johnsonella	0.026	0.041	5.20.E-01	9.57.E-01
Phycodnaviridae	L6_Johnsonella	0.022	0.036	5.41.E-01	9.60.E-01
Podoviridae	L6_Johnsonella	0.086	0.042	4.37.E-02	7.22.E-01
Siphoviridae	L6_Johnsonella	0.014	0.038	7.04.E-01	9.81.E-01
Autographiviridae	L6_Bacteroides	0.006	0.016	7.03.E-01	9.81.E-01
crAss_like_phage	L6_Bacteroides	-0.034	0.018	5.42.E-02	7.23.E-01
Herelleviridae	L6_Bacteroides	-0.029	0.017	8.30.E-02	7.49.E-01
Microviridae	L6_Bacteroides	-0.018	0.017	2.76.E-01	9.05.E-01
Myoviridae	L6_Bacteroides	0.011	0.019	5.50.E-01	9.60.E-01
Phycodnaviridae	L6_Bacteroides	-0.010	0.016	5.47.E-01	9.60.E-01
Podoviridae	L6_Bacteroides	-0.026	0.019	1.76.E-01	8.58.E-01
Siphoviridae	L6_Bacteroides	-0.025	0.017	1.37.E-01	8.09.E-01

Autographiviridae	L6_Culturomica	0.011	0.027	6.91.E-01	9.80.E-01
crAss_like_phage	L6_Culturomica	-0.036	0.030	2.22.E-01	8.80.E-01
Herelleviridae	L6_Culturomica	-0.002	0.028	9.54.E-01	9.98.E-01
Microviridae	L6_Culturomica	0.021	0.028	4.47.E-01	9.34.E-01
Myoviridae	L6_Culturomica	0.035	0.031	2.63.E-01	9.00.E-01
Phycodnaviridae	L6_Culturomica	0.051	0.028	6.45.E-02	7.24.E-01
Podoviridae	L6_Culturomica	-0.019	0.032	5.51.E-01	9.60.E-01
Siphoviridae	L6_Culturomica	-0.007	0.029	8.02.E-01	9.92.E-01
Autographiviridae	L6_Weissella	-0.024	0.041	5.57.E-01	9.61.E-01
crAss_like_phage	L6_Weissella	-0.035	0.045	4.40.E-01	9.31.E-01
Herelleviridae	L6_Weissella	0.021	0.042	6.18.E-01	9.75.E-01
Microviridae	L6_Weissella	0.006	0.042	8.89.E-01	9.96.E-01
Myoviridae	L6_Weissella	-0.108	0.047	2.14.E-02	6.06.E-01
Phycodnaviridae	L6_Weissella	-0.013	0.042	7.61.E-01	9.91.E-01
Podoviridae	L6_Weissella	-0.053	0.049	2.80.E-01	9.12.E-01
Siphoviridae	L6_Weissella	-0.040	0.043	3.52.E-01	9.15.E-01
Autographiviridae	L6_Actinomyces	-0.055	0.027	4.65.E-02	7.22.E-01
crAss_like_phage	L6_Actinomyces	-0.013	0.030	6.64.E-01	9.79.E-01
Herelleviridae	L6_Actinomyces	-0.066	0.028	1.99.E-02	5.93.E-01
Microviridae	L6_Actinomyces	0.008	0.029	7.74.E-01	9.92.E-01
Myoviridae	L6_Actinomyces	-0.033	0.032	3.03.E-01	9.15.E-01
Phycodnaviridae	L6_Actinomyces	-0.024	0.028	3.88.E-01	9.15.E-01
Podoviridae	L6_Actinomyces	-0.014	0.033	6.62.E-01	9.79.E-01
Siphoviridae	L6_Actinomyces	-0.003	0.029	9.17.E-01	9.98.E-01
Autographiviridae	L6_Peptostreptococcus	0.035	0.041	3.95.E-01	9.15.E-01
crAss_like_phage	L6_Peptostreptococcus	0.045	0.046	3.20.E-01	9.15.E-01
Herelleviridae	L6_Peptostreptococcus	0.017	0.043	6.91.E-01	9.80.E-01
Microviridae	L6_Peptostreptococcus	-0.029	0.043	5.03.E-01	9.55.E-01
Myoviridae	L6_Peptostreptococcus	-0.023	0.048	6.35.E-01	9.76.E-01
Phycodnaviridae	L6_Peptostreptococcus	-0.010	0.042	8.12.E-01	9.93.E-01
Podoviridae	L6_Peptostreptococcus	0.011	0.050	8.30.E-01	9.94.E-01
Siphoviridae	L6_Peptostreptococcus	0.003	0.044	9.39.E-01	9.98.E-01
Autographiviridae	L6_g_Ruminococcaceae_bacterium	-0.035	0.029	2.27.E-01	8.82.E-01
crAss_like_phage	L6_g_Ruminococcaceae_bacterium	0.017	0.032	5.98.E-01	9.69.E-01
Herelleviridae	L6_g_Ruminococcaceae_bacterium	-0.013	0.030	6.64.E-01	9.79.E-01
Microviridae	L6_g_Ruminococcaceae_bacterium	0.004	0.030	8.82.E-01	9.96.E-01
Myoviridae	L6_g_Ruminococcaceae_bacterium	0.044	0.034	1.96.E-01	8.66.E-01
Phycodnaviridae	L6_g_Ruminococcaceae_bacterium	-0.055	0.030	6.44.E-02	7.24.E-01
Podoviridae	L6_g_Ruminococcaceae_bacterium	0.013	0.035	7.03.E-01	9.81.E-01
Siphoviridae	L6_g_Ruminococcaceae_bacterium	-0.033	0.031	2.94.E-01	9.15.E-01
Autographiviridae	L6_Klebsiella	-0.003	0.024	8.99.E-01	9.97.E-01
crAss_like_phage	L6_Klebsiella	-0.009	0.026	7.38.E-01	9.88.E-01
Herelleviridae	L6_Klebsiella	-0.010	0.025	6.76.E-01	9.80.E-01
Microviridae	L6_Klebsiella	-0.006	0.025	8.05.E-01	9.93.E-01
Myoviridae	L6_Klebsiella	-0.048	0.027	7.88.E-02	7.43.E-01
Phycodnaviridae	L6_Klebsiella	-0.015	0.024	5.41.E-01	9.60.E-01
Podoviridae	L6_Klebsiella	-0.021	0.028	4.63.E-01	9.45.E-01
Siphoviridae	L6_Klebsiella	-0.021	0.025	3.96.E-01	9.15.E-01
Autographiviridae	L6_Harryflintia	0.000	0.025	9.97.E-01	9.99.E-01
crAss_like_phage	L6_Harryflintia	0.004	0.028	8.85.E-01	9.96.E-01
Herelleviridae	L6_Harryflintia	-0.024	0.026	3.52.E-01	9.15.E-01
Microviridae	L6_Harryflintia	0.016	0.026	5.30.E-01	9.57.E-01
Myoviridae	L6_Harryflintia	0.031	0.029	2.92.E-01	9.15.E-01
Phycodnaviridae	L6_Harryflintia	-0.034	0.026	1.83.E-01	8.60.E-01
Podoviridae	L6_Harryflintia	-0.031	0.030	3.02.E-01	9.15.E-01
Siphoviridae	L6_Harryflintia	-0.031	0.027	2.41.E-01	8.85.E-01
Autographiviridae	L6_Rothia	0.052	0.030	8.79.E-02	7.54.E-01
crAss_like_phage	L6_Rothia	0.030	0.033	3.74.E-01	9.15.E-01
Herelleviridae	L6_Rothia	0.008	0.031	7.92.E-01	9.92.E-01
Microviridae	L6_Rothia	0.019	0.032	5.51.E-01	9.60.E-01
Myoviridae	L6_Rothia	0.115	0.035	9.79.E-04	2.79.E-01
Phycodnaviridae	L6_Rothia	-0.001	0.031	9.78.E-01	9.99.E-01
Podoviridae	L6_Rothia	0.058	0.036	1.08.E-01	7.82.E-01
Siphoviridae	L6_Rothia	0.030	0.032	3.49.E-01	9.15.E-01
Autographiviridae	L6_Campylobacter	-0.002	0.028	9.57.E-01	9.98.E-01

crAss_like_phage	L6_Campylobacter	0.001	0.031	9.78.E-01	9.99.E-01
Herelleviridae	L6_Campylobacter	0.016	0.029	5.87.E-01	9.67.E-01
Microviridae	L6_Campylobacter	-0.034	0.029	2.39.E-01	8.85.E-01
Myoviridae	L6_Campylobacter	-0.005	0.032	8.83.E-01	9.96.E-01
Phycodnaviridae	L6_Campylobacter	-0.001	0.029	9.59.E-01	9.98.E-01
Podoviridae	L6_Campylobacter	-0.033	0.033	3.17.E-01	9.15.E-01
Siphoviridae	L6_Campylobacter	-0.015	0.030	6.17.E-01	9.75.E-01
Autographiviridae	L6_Peptoanaerobacter	0.009	0.040	8.12.E-01	9.93.E-01
crAss_like_phage	L6_Peptoanaerobacter	-0.097	0.044	2.70.E-02	6.58.E-01
Herelleviridae	L6_Peptoanaerobacter	-0.012	0.042	7.70.E-01	9.91.E-01
Microviridae	L6_Peptoanaerobacter	0.027	0.042	5.10.E-01	9.57.E-01
Myoviridae	L6_Peptoanaerobacter	0.040	0.046	3.83.E-01	9.15.E-01
Phycodnaviridae	L6_Peptoanaerobacter	-0.018	0.041	6.62.E-01	9.79.E-01
Podoviridae	L6_Peptoanaerobacter	0.063	0.048	1.91.E-01	8.61.E-01
Siphoviridae	L6_Peptoanaerobacter	0.025	0.043	5.53.E-01	9.60.E-01
Autographiviridae	L6_Mitsuokella	-0.057	0.039	1.42.E-01	8.10.E-01
crAss_like_phage	L6_Mitsuokella	-0.017	0.043	6.86.E-01	9.80.E-01
Herelleviridae	L6_Mitsuokella	-0.086	0.040	3.24.E-02	6.75.E-01
Microviridae	L6_Mitsuokella	-0.001	0.041	9.76.E-01	9.99.E-01
Myoviridae	L6_Mitsuokella	0.057	0.045	2.10.E-01	8.76.E-01
Phycodnaviridae	L6_Mitsuokella	-0.003	0.040	9.49.E-01	9.98.E-01
Podoviridae	L6_Mitsuokella	-0.052	0.047	2.68.E-01	9.01.E-01
Siphoviridae	L6_Mitsuokella	0.012	0.042	7.65.E-01	9.91.E-01
Autographiviridae	L6_Leuconostoc	0.002	0.039	9.62.E-01	9.98.E-01
crAss_like_phage	L6_Leuconostoc	0.006	0.044	8.88.E-01	9.96.E-01
Herelleviridae	L6_Leuconostoc	0.057	0.041	1.64.E-01	8.39.E-01
Microviridae	L6_Leuconostoc	0.018	0.041	6.64.E-01	9.79.E-01
Myoviridae	L6_Leuconostoc	-0.031	0.046	4.93.E-01	9.55.E-01
Phycodnaviridae	L6_Leuconostoc	0.021	0.040	5.96.E-01	9.69.E-01
Podoviridae	L6_Leuconostoc	-0.013	0.047	7.87.E-01	9.92.E-01
Siphoviridae	L6_Leuconostoc	0.044	0.042	2.93.E-01	9.15.E-01
Autographiviridae	L6_Cronobacter	-0.042	0.023	6.32.E-02	7.24.E-01
crAss_like_phage	L6_Cronobacter	0.025	0.025	3.09.E-01	9.15.E-01
Herelleviridae	L6_Cronobacter	-0.050	0.023	3.38.E-02	6.82.E-01
Microviridae	L6_Cronobacter	0.000	0.024	9.95.E-01	9.99.E-01
Myoviridae	L6_Cronobacter	0.001	0.026	9.55.E-01	9.98.E-01
Phycodnaviridae	L6_Cronobacter	-0.042	0.023	7.06.E-02	7.24.E-01
Podoviridae	L6_Cronobacter	-0.012	0.027	6.72.E-01	9.80.E-01
Siphoviridae	L6_Cronobacter	-0.001	0.024	9.55.E-01	9.98.E-01
Autographiviridae	L6_Cryptobacterium	-0.002	0.041	9.54.E-01	9.98.E-01
crAss_like_phage	L6_Cryptobacterium	-0.036	0.046	4.38.E-01	9.29.E-01
Herelleviridae	L6_Cryptobacterium	0.047	0.043	2.71.E-01	9.02.E-01
Microviridae	L6_Cryptobacterium	-0.039	0.043	3.70.E-01	9.15.E-01
Myoviridae	L6_Cryptobacterium	0.034	0.048	4.87.E-01	9.54.E-01
Phycodnaviridae	L6_Cryptobacterium	0.001	0.043	9.87.E-01	9.99.E-01
Podoviridae	L6_Cryptobacterium	-0.077	0.050	1.22.E-01	7.93.E-01
Siphoviridae	L6_Cryptobacterium	-0.094	0.044	3.38.E-02	6.82.E-01
Autographiviridae	L6_Coprococcus	0.026	0.039	5.08.E-01	9.57.E-01
crAss_like_phage	L6_Coprococcus	-0.037	0.044	4.03.E-01	9.16.E-01
Herelleviridae	L6_Coprococcus	0.078	0.041	5.74.E-02	7.24.E-01
Microviridae	L6_Coprococcus	0.014	0.041	7.34.E-01	9.88.E-01
Myoviridae	L6_Coprococcus	-0.086	0.046	5.97.E-02	7.24.E-01
Phycodnaviridae	L6_Coprococcus	0.026	0.041	5.21.E-01	9.57.E-01
Podoviridae	L6_Coprococcus	-0.024	0.047	6.10.E-01	9.75.E-01
Siphoviridae	L6_Coprococcus	0.002	0.042	9.62.E-01	9.98.E-01
Autographiviridae	L6_Turicibacter	0.050	0.035	1.55.E-01	8.26.E-01
crAss_like_phage	L6_Turicibacter	0.000	0.039	9.96.E-01	9.99.E-01
Herelleviridae	L6_Turicibacter	0.094	0.036	8.92.E-03	4.93.E-01
Microviridae	L6_Turicibacter	0.052	0.036	1.53.E-01	8.24.E-01
Myoviridae	L6_Turicibacter	-0.041	0.040	3.11.E-01	9.15.E-01
Phycodnaviridae	L6_Turicibacter	0.056	0.036	1.17.E-01	7.86.E-01
Podoviridae	L6_Turicibacter	0.018	0.042	6.62.E-01	9.79.E-01
Siphoviridae	L6_Turicibacter	-0.039	0.037	2.96.E-01	9.15.E-01
Autographiviridae	L6_Desulfotomaculum	0.004	0.034	9.09.E-01	9.98.E-01
crAss_like_phage	L6_Desulfotomaculum	-0.044	0.038	2.46.E-01	8.92.E-01

Herelleviridae	L6_Desulfotomaculum	-0.014	0.035	6.98.E-01	9.81.E-01
Microviridae	L6_Desulfotomaculum	0.017	0.036	6.26.E-01	9.76.E-01
Myoviridae	L6_Desulfotomaculum	0.064	0.040	1.05.E-01	7.82.E-01
Phycodnaviridae	L6_Desulfotomaculum	-0.045	0.035	1.97.E-01	8.66.E-01
Podoviridae	L6_Desulfotomaculum	0.002	0.041	9.60.E-01	9.98.E-01
Siphoviridae	L6_Desulfotomaculum	0.032	0.036	3.84.E-01	9.15.E-01
Autographiviridae	L6_Stomatobaculum	0.028	0.041	4.93.E-01	9.55.E-01
crAss_like_phage	L6_Stomatobaculum	0.078	0.045	8.38.E-02	7.50.E-01
Herelleviridae	L6_Stomatobaculum	-0.036	0.042	3.89.E-01	9.15.E-01
Microviridae	L6_Stomatobaculum	-0.013	0.042	7.61.E-01	9.91.E-01
Myoviridae	L6_Stomatobaculum	0.093	0.047	4.94.E-02	7.22.E-01
Phycodnaviridae	L6_Stomatobaculum	-0.027	0.042	5.18.E-01	9.57.E-01
Podoviridae	L6_Stomatobaculum	0.009	0.049	8.52.E-01	9.95.E-01
Siphoviridae	L6_Stomatobaculum	-0.028	0.044	5.26.E-01	9.57.E-01
Autographiviridae	L6_Riemerella	0.021	0.021	3.33.E-01	9.15.E-01
crAss_like_phage	L6_Riemerella	-0.007	0.024	7.71.E-01	9.92.E-01
Herelleviridae	L6_Riemerella	-0.027	0.022	2.29.E-01	8.82.E-01
Microviridae	L6_Riemerella	-0.009	0.022	6.96.E-01	9.81.E-01
Myoviridae	L6_Riemerella	-0.015	0.025	5.44.E-01	9.60.E-01
Phycodnaviridae	L6_Riemerella	0.002	0.022	9.25.E-01	9.98.E-01
Podoviridae	L6_Riemerella	0.030	0.026	2.40.E-01	8.85.E-01
Siphoviridae	L6_Riemerella	-0.025	0.023	2.70.E-01	9.02.E-01
Autographiviridae	L6_Megasphaera	0.032	0.042	4.43.E-01	9.32.E-01
crAss_like_phage	L6_Megasphaera	0.007	0.047	8.86.E-01	9.96.E-01
Herelleviridae	L6_Megasphaera	-0.037	0.044	3.94.E-01	9.15.E-01
Microviridae	L6_Megasphaera	-0.023	0.044	6.05.E-01	9.73.E-01
Myoviridae	L6_Megasphaera	0.007	0.049	8.90.E-01	9.96.E-01
Phycodnaviridae	L6_Megasphaera	-0.054	0.043	2.14.E-01	8.78.E-01
Podoviridae	L6_Megasphaera	-0.027	0.051	5.98.E-01	9.69.E-01
Siphoviridae	L6_Megasphaera	0.030	0.045	5.06.E-01	9.57.E-01
Autographiviridae	L6_Selenomonas	-0.020	0.043	6.37.E-01	9.77.E-01
crAss_like_phage	L6_Selenomonas	-0.048	0.047	3.11.E-01	9.15.E-01
Herelleviridae	L6_Selenomonas	-0.095	0.044	3.11.E-02	6.70.E-01
Microviridae	L6_Selenomonas	-0.009	0.044	8.43.E-01	9.95.E-01
Myoviridae	L6_Selenomonas	0.097	0.049	4.91.E-02	7.22.E-01
Phycodnaviridae	L6_Selenomonas	0.031	0.044	4.85.E-01	9.54.E-01
Podoviridae	L6_Selenomonas	-0.017	0.051	7.40.E-01	9.88.E-01
Siphoviridae	L6_Selenomonas	-0.040	0.045	3.82.E-01	9.15.E-01
Autographiviridae	L6_Bilophila	0.056	0.037	1.34.E-01	8.06.E-01
crAss_like_phage	L6_Bilophila	-0.019	0.041	6.37.E-01	9.77.E-01
Herelleviridae	L6_Bilophila	-0.028	0.039	4.64.E-01	9.45.E-01
Microviridae	L6_Bilophila	0.030	0.039	4.35.E-01	9.26.E-01
Myoviridae	L6_Bilophila	0.035	0.043	4.13.E-01	9.18.E-01
Phycodnaviridae	L6_Bilophila	0.003	0.038	9.42.E-01	9.98.E-01
Podoviridae	L6_Bilophila	-0.002	0.045	9.73.E-01	9.99.E-01
Siphoviridae	L6_Bilophila	-0.005	0.040	8.96.E-01	9.97.E-01
Autographiviridae	L6_Delftia	-0.026	0.020	2.10.E-01	8.76.E-01
crAss_like_phage	L6_Delftia	-0.062	0.022	5.57.E-03	4.30.E-01
Herelleviridae	L6_Delftia	-0.020	0.021	3.48.E-01	9.15.E-01
Microviridae	L6_Delftia	-0.001	0.021	9.72.E-01	9.99.E-01
Myoviridae	L6_Delftia	0.027	0.024	2.57.E-01	8.95.E-01
Phycodnaviridae	L6_Delftia	-0.005	0.021	8.30.E-01	9.94.E-01
Podoviridae	L6_Delftia	-0.045	0.024	6.87.E-02	7.24.E-01
Siphoviridae	L6_Delftia	-0.059	0.022	7.06.E-03	4.53.E-01
Autographiviridae	L6_Flavonifractor	0.031	0.031	3.08.E-01	9.15.E-01
crAss_like_phage	L6_Flavonifractor	-0.059	0.034	8.06.E-02	7.47.E-01
Herelleviridae	L6_Flavonifractor	-0.061	0.032	5.36.E-02	7.22.E-01
Microviridae	L6_Flavonifractor	0.001	0.032	9.66.E-01	9.98.E-01
Myoviridae	L6_Flavonifractor	-0.036	0.036	3.13.E-01	9.15.E-01
Phycodnaviridae	L6_Flavonifractor	0.031	0.031	3.24.E-01	9.15.E-01
Podoviridae	L6_Flavonifractor	-0.021	0.037	5.66.E-01	9.61.E-01
Siphoviridae	L6_Flavonifractor	-0.005	0.033	8.89.E-01	9.96.E-01
Autographiviridae	L6_Gemmiger	-0.061	0.035	8.61.E-02	7.52.E-01
crAss_like_phage	L6_Gemmiger	0.076	0.039	5.25.E-02	7.22.E-01
Herelleviridae	L6_Gemmiger	-0.038	0.037	3.02.E-01	9.15.E-01

Microviridae	L6_Gemmiger	-0.025	0.037	4.92.E-01	9.55.E-01
Myoviridae	L6_Gemmiger	-0.035	0.041	4.01.E-01	9.15.E-01
Phycodnaviridae	L6_Gemmiger	0.032	0.036	3.86.E-01	9.15.E-01
Podoviridae	L6_Gemmiger	0.037	0.043	3.88.E-01	9.15.E-01
Siphoviridae	L6_Gemmiger	0.010	0.038	7.93.E-01	9.92.E-01
Autographiviridae	L6_Anaerofustis	0.021	0.035	5.58.E-01	9.61.E-01
crAss_like_phage	L6_Anaerofustis	0.013	0.039	7.37.E-01	9.88.E-01
Herelleviridae	L6_Anaerofustis	0.035	0.037	3.39.E-01	9.15.E-01
Microviridae	L6_Anaerofustis	-0.013	0.037	7.33.E-01	9.88.E-01
Myoviridae	L6_Anaerofustis	-0.046	0.041	2.57.E-01	8.95.E-01
Phycodnaviridae	L6_Anaerofustis	-0.052	0.036	1.55.E-01	8.26.E-01
Podoviridae	L6_Anaerofustis	0.029	0.042	4.93.E-01	9.55.E-01
Siphoviridae	L6_Anaerofustis	0.031	0.038	4.18.E-01	9.19.E-01
Autographiviridae	L6_Blautia	-0.014	0.030	6.35.E-01	9.76.E-01
crAss_like_phage	L6_Blautia	-0.033	0.033	3.18.E-01	9.15.E-01
Herelleviridae	L6_Blautia	-0.006	0.031	8.55.E-01	9.96.E-01
Microviridae	L6_Blautia	0.016	0.031	6.19.E-01	9.75.E-01
Myoviridae	L6_Blautia	-0.045	0.035	2.02.E-01	8.75.E-01
Phycodnaviridae	L6_Blautia	0.047	0.031	1.30.E-01	7.99.E-01
Podoviridae	L6_Blautia	-0.003	0.036	9.34.E-01	9.98.E-01
Siphoviridae	L6_Blautia	0.011	0.032	7.37.E-01	9.88.E-01
Autographiviridae	L6_Gardnerella	0.049	0.039	2.19.E-01	8.79.E-01
crAss_like_phage	L6_Gardnerella	0.001	0.044	9.87.E-01	9.99.E-01
Herelleviridae	L6_Gardnerella	0.010	0.041	8.07.E-01	9.93.E-01
Microviridae	L6_Gardnerella	0.012	0.041	7.67.E-01	9.91.E-01
Myoviridae	L6_Gardnerella	0.051	0.046	2.68.E-01	9.01.E-01
Phycodnaviridae	L6_Gardnerella	0.045	0.041	2.67.E-01	9.01.E-01
Podoviridae	L6_Gardnerella	0.038	0.047	4.24.E-01	9.23.E-01
Siphoviridae	L6_Gardnerella	-0.044	0.042	2.99.E-01	9.15.E-01
Autographiviridae	L6_Fusicatenibacter	-0.035	0.035	3.20.E-01	9.15.E-01
crAss_like_phage	L6_Fusicatenibacter	-0.082	0.038	3.22.E-02	6.75.E-01
Herelleviridae	L6_Fusicatenibacter	-0.008	0.036	8.26.E-01	9.93.E-01
Microviridae	L6_Fusicatenibacter	-0.025	0.036	5.00.E-01	9.55.E-01
Myoviridae	L6_Fusicatenibacter	-0.020	0.040	6.17.E-01	9.75.E-01
Phycodnaviridae	L6_Fusicatenibacter	0.018	0.036	6.11.E-01	9.75.E-01
Podoviridae	L6_Fusicatenibacter	0.017	0.042	6.88.E-01	9.80.E-01
Siphoviridae	L6_Fusicatenibacter	0.001	0.037	9.72.E-01	9.99.E-01
Autographiviridae	L6_Actinobacillus	0.002	0.012	8.48.E-01	9.95.E-01
crAss_like_phage	L6_Actinobacillus	-0.028	0.014	4.09.E-02	7.06.E-01
Herelleviridae	L6_Actinobacillus	-0.018	0.013	1.60.E-01	8.38.E-01
Microviridae	L6_Actinobacillus	0.038	0.013	3.61.E-03	4.02.E-01
Myoviridae	L6_Actinobacillus	-0.012	0.014	4.01.E-01	9.15.E-01
Phycodnaviridae	L6_Actinobacillus	0.002	0.013	8.74.E-01	9.96.E-01
Podoviridae	L6_Actinobacillus	-0.014	0.015	3.42.E-01	9.15.E-01
Siphoviridae	L6_Actinobacillus	-0.039	0.013	2.97.E-03	3.87.E-01
Autographiviridae	L6_Butyricimonas	0.007	0.022	7.49.E-01	9.89.E-01
crAss_like_phage	L6_Butyricimonas	-0.010	0.024	6.83.E-01	9.80.E-01
Herelleviridae	L6_Butyricimonas	0.010	0.023	6.58.E-01	9.79.E-01
Microviridae	L6_Butyricimonas	0.024	0.023	3.04.E-01	9.15.E-01
Myoviridae	L6_Butyricimonas	0.011	0.025	6.73.E-01	9.80.E-01
Phycodnaviridae	L6_Butyricimonas	-0.019	0.023	4.09.E-01	9.17.E-01
Podoviridae	L6_Butyricimonas	-0.031	0.026	2.45.E-01	8.91.E-01
Siphoviridae	L6_Butyricimonas	0.065	0.023	5.41.E-03	4.28.E-01
Autographiviridae	L6_Alloscardovia	-0.038	0.041	3.54.E-01	9.15.E-01
crAss_like_phage	L6_Alloscardovia	-0.025	0.045	5.86.E-01	9.65.E-01
Herelleviridae	L6_Alloscardovia	0.060	0.043	1.58.E-01	8.38.E-01
Microviridae	L6_Alloscardovia	0.024	0.043	5.74.E-01	9.62.E-01
Myoviridae	L6_Alloscardovia	-0.027	0.048	5.67.E-01	9.61.E-01
Phycodnaviridae	L6_Alloscardovia	0.053	0.042	2.12.E-01	8.78.E-01
Podoviridae	L6_Alloscardovia	-0.057	0.049	2.47.E-01	8.92.E-01
Siphoviridae	L6_Alloscardovia	-0.029	0.044	5.12.E-01	9.57.E-01
Autographiviridae	L6_Murdochiella	0.027	0.033	4.16.E-01	9.18.E-01
crAss_like_phage	L6_Murdochiella	0.024	0.037	5.14.E-01	9.57.E-01
Herelleviridae	L6_Murdochiella	0.017	0.035	6.34.E-01	9.76.E-01
Microviridae	L6_Murdochiella	-0.005	0.035	8.94.E-01	9.97.E-01

Myoviridae	L6_Murdochiella	0.084	0.039	3.01.E-02	6.66.E-01
Phycodnaviridae	L6_Murdochiella	0.023	0.034	5.12.E-01	9.57.E-01
Podoviridae	L6_Murdochiella	0.057	0.040	1.57.E-01	8.33.E-01
Siphoviridae	L6_Murdochiella	-0.002	0.036	9.62.E-01	9.98.E-01
Autographiviridae	L6_Anaerococcus	0.076	0.040	5.98.E-02	7.24.E-01
crAss_like_phage	L6_Anaerococcus	0.025	0.045	5.68.E-01	9.61.E-01
Herelleviridae	L6_Anaerococcus	0.080	0.042	5.66.E-02	7.24.E-01
Microviridae	L6_Anaerococcus	-0.004	0.042	9.18.E-01	9.98.E-01
Myoviridae	L6_Anaerococcus	-0.033	0.047	4.78.E-01	9.52.E-01
Phycodnaviridae	L6_Anaerococcus	0.002	0.041	9.60.E-01	9.98.E-01
Podoviridae	L6_Anaerococcus	-0.046	0.048	3.43.E-01	9.15.E-01
Siphoviridae	L6_Anaerococcus	-0.003	0.043	9.36.E-01	9.98.E-01
Autographiviridae	L6_Oscillibacter	0.007	0.027	7.89.E-01	9.92.E-01
crAss_like_phage	L6_Oscillibacter	-0.005	0.030	8.81.E-01	9.96.E-01
Herelleviridae	L6_Oscillibacter	-0.027	0.028	3.36.E-01	9.15.E-01
Microviridae	L6_Oscillibacter	-0.008	0.028	7.88.E-01	9.92.E-01
Myoviridae	L6_Oscillibacter	-0.077	0.031	1.54.E-02	5.72.E-01
Phycodnaviridae	L6_Oscillibacter	-0.001	0.028	9.82.E-01	9.99.E-01
Podoviridae	L6_Oscillibacter	0.001	0.033	9.76.E-01	9.99.E-01
Siphoviridae	L6_Oscillibacter	0.021	0.029	4.82.E-01	9.53.E-01
Autographiviridae	L6_Neglecta	-0.038	0.032	2.36.E-01	8.85.E-01
crAss_like_phage	L6_Neglecta	0.047	0.035	1.88.E-01	8.61.E-01
Herelleviridae	L6_Neglecta	-0.068	0.033	4.27.E-02	7.19.E-01
Microviridae	L6_Neglecta	0.079	0.033	1.86.E-02	5.90.E-01
Myoviridae	L6_Neglecta	-0.014	0.037	7.00.E-01	9.81.E-01
Phycodnaviridae	L6_Neglecta	0.005	0.033	8.89.E-01	9.96.E-01
Podoviridae	L6_Neglecta	-0.021	0.039	5.92.E-01	9.68.E-01
Siphoviridae	L6_Neglecta	-0.047	0.034	1.74.E-01	8.56.E-01
Autographiviridae	L6_Agathobaculum	-0.061	0.038	1.10.E-01	7.82.E-01
crAss_like_phage	L6_Agathobaculum	0.064	0.042	1.32.E-01	8.03.E-01
Herelleviridae	L6_Agathobaculum	0.059	0.040	1.37.E-01	8.09.E-01
Microviridae	L6_Agathobaculum	-0.032	0.040	4.20.E-01	9.20.E-01
Myoviridae	L6_Agathobaculum	0.071	0.045	1.12.E-01	7.83.E-01
Phycodnaviridae	L6_Agathobaculum	-0.003	0.040	9.34.E-01	9.98.E-01
Podoviridae	L6_Agathobaculum	0.087	0.046	6.08.E-02	7.24.E-01
Siphoviridae	L6_Agathobaculum	0.105	0.041	1.03.E-02	5.13.E-01
Autographiviridae	L6_Cloacibacillus	0.080	0.038	3.70.E-02	6.91.E-01
crAss_like_phage	L6_Cloacibacillus	-0.024	0.042	5.66.E-01	9.61.E-01
Herelleviridae	L6_Cloacibacillus	-0.004	0.040	9.11.E-01	9.98.E-01
Microviridae	L6_Cloacibacillus	0.022	0.040	5.90.E-01	9.68.E-01
Myoviridae	L6_Cloacibacillus	-0.017	0.045	7.06.E-01	9.82.E-01
Phycodnaviridae	L6_Cloacibacillus	-0.046	0.039	2.46.E-01	8.92.E-01
Podoviridae	L6_Cloacibacillus	-0.072	0.046	1.20.E-01	7.91.E-01
Siphoviridae	L6_Cloacibacillus	-0.007	0.041	8.74.E-01	9.96.E-01
Autographiviridae	L6_Leclercia	0.005	0.031	8.79.E-01	9.96.E-01
crAss_like_phage	L6_Leclercia	-0.003	0.034	9.33.E-01	9.98.E-01
Herelleviridae	L6_Leclercia	-0.018	0.032	5.68.E-01	9.61.E-01
Microviridae	L6_Leclercia	-0.002	0.032	9.40.E-01	9.98.E-01
Myoviridae	L6_Leclercia	-0.001	0.036	9.71.E-01	9.99.E-01
Phycodnaviridae	L6_Leclercia	-0.008	0.032	7.95.E-01	9.92.E-01
Podoviridae	L6_Leclercia	0.109	0.037	3.33.E-03	3.95.E-01
Siphoviridae	L6_Leclercia	0.013	0.033	7.00.E-01	9.81.E-01
Autographiviridae	L6_Scardovia	-0.042	0.039	2.83.E-01	9.13.E-01
crAss_like_phage	L6_Scardovia	-0.042	0.044	3.33.E-01	9.15.E-01
Herelleviridae	L6_Scardovia	0.056	0.041	1.71.E-01	8.50.E-01
Microviridae	L6_Scardovia	0.041	0.041	3.24.E-01	9.15.E-01
Myoviridae	L6_Scardovia	-0.054	0.046	2.38.E-01	8.85.E-01
Phycodnaviridae	L6_Scardovia	0.012	0.041	7.74.E-01	9.92.E-01
Podoviridae	L6_Scardovia	-0.089	0.047	6.02.E-02	7.24.E-01
Siphoviridae	L6_Scardovia	0.009	0.042	8.40.E-01	9.95.E-01
Autographiviridae	L6_Methanobrevibacter	0.045	0.036	2.08.E-01	8.76.E-01
crAss_like_phage	L6_Methanobrevibacter	-0.029	0.040	4.62.E-01	9.44.E-01
Herelleviridae	L6_Methanobrevibacter	0.070	0.037	6.23.E-02	7.24.E-01
Microviridae	L6_Methanobrevibacter	0.041	0.038	2.81.E-01	9.12.E-01
Myoviridae	L6_Methanobrevibacter	0.011	0.042	8.01.E-01	9.92.E-01

Phycodnaviridae	L6_Methanobrevibacter	-0.025	0.037	4.98.E-01	9.55.E-01
Podoviridae	L6_Methanobrevibacter	-0.024	0.043	5.73.E-01	9.61.E-01
Siphoviridae	L6_Methanobrevibacter	-0.018	0.039	6.33.E-01	9.76.E-01
Autographiviridae	L6_Sellimonas	0.064	0.036	7.51.E-02	7.30.E-01
crAss_like_phage	L6_Sellimonas	0.020	0.040	6.16.E-01	9.75.E-01
Herelleviridae	L6_Sellimonas	0.005	0.037	8.98.E-01	9.97.E-01
Microviridae	L6_Sellimonas	0.004	0.037	9.20.E-01	9.98.E-01
Myoviridae	L6_Sellimonas	0.125	0.041	2.58.E-03	3.52.E-01
Phycodnaviridae	L6_Sellimonas	-0.048	0.037	1.92.E-01	8.61.E-01
Podoviridae	L6_Sellimonas	-0.066	0.043	1.22.E-01	7.93.E-01
Siphoviridae	L6_Sellimonas	0.019	0.038	6.24.E-01	9.76.E-01
Autographiviridae	L6_Meiothermus	0.000	0.008	9.95.E-01	9.99.E-01
crAss_like_phage	L6_Meiothermus	-0.014	0.009	1.09.E-01	7.82.E-01
Herelleviridae	L6_Meiothermus	-0.003	0.008	7.12.E-01	9.85.E-01
Microviridae	L6_Meiothermus	0.007	0.008	3.69.E-01	9.15.E-01
Myoviridae	L6_Meiothermus	-0.007	0.009	4.21.E-01	9.20.E-01
Phycodnaviridae	L6_Meiothermus	-0.004	0.008	5.85.E-01	9.65.E-01
Podoviridae	L6_Meiothermus	-0.010	0.009	2.71.E-01	9.02.E-01
Siphoviridae	L6_Meiothermus	-0.011	0.008	1.78.E-01	8.59.E-01
Autographiviridae	L6_Lachnoanaerobaculum	-0.026	0.040	5.04.E-01	9.56.E-01
crAss_like_phage	L6_Lachnoanaerobaculum	0.061	0.044	1.63.E-01	8.38.E-01
Herelleviridae	L6_Lachnoanaerobaculum	0.025	0.041	5.42.E-01	9.60.E-01
Microviridae	L6_Lachnoanaerobaculum	0.098	0.041	1.70.E-02	5.86.E-01
Myoviridae	L6_Lachnoanaerobaculum	0.142	0.046	1.97.E-03	3.37.E-01
Phycodnaviridae	L6_Lachnoanaerobaculum	-0.007	0.041	8.62.E-01	9.96.E-01
Podoviridae	L6_Lachnoanaerobaculum	0.082	0.047	8.51.E-02	7.50.E-01
Siphoviridae	L6_Lachnoanaerobaculum	0.083	0.042	4.88.E-02	7.22.E-01
Autographiviridae	L6_Acinetobacter	-0.054	0.044	2.19.E-01	8.79.E-01
crAss_like_phage	L6_Acinetobacter	-0.048	0.049	3.24.E-01	9.15.E-01
Herelleviridae	L6_Acinetobacter	-0.031	0.046	5.04.E-01	9.56.E-01
Microviridae	L6_Acinetobacter	-0.001	0.046	9.77.E-01	9.99.E-01
Myoviridae	L6_Acinetobacter	0.058	0.051	2.59.E-01	8.95.E-01
Phycodnaviridae	L6_Acinetobacter	0.010	0.045	8.33.E-01	9.94.E-01
Podoviridae	L6_Acinetobacter	0.034	0.053	5.20.E-01	9.57.E-01
Siphoviridae	L6_Acinetobacter	0.043	0.047	3.66.E-01	9.15.E-01
Autographiviridae	L6_g_butyrate.producing_bacterium	-0.030	0.035	3.89.E-01	9.15.E-01
crAss_like_phage	L6_g_butyrate.producing_bacterium	-0.035	0.039	3.68.E-01	9.15.E-01
Herelleviridae	L6_g_butyrate.producing_bacterium	-0.051	0.036	1.63.E-01	8.38.E-01
Microviridae	L6_g_butyrate.producing_bacterium	-0.042	0.037	2.54.E-01	8.95.E-01
Myoviridae	L6_g_butyrate.producing_bacterium	0.008	0.041	8.49.E-01	9.95.E-01
Phycodnaviridae	L6_g_butyrate.producing_bacterium	-0.023	0.036	5.27.E-01	9.57.E-01
Podoviridae	L6_g_butyrate.producing_bacterium	0.010	0.042	8.11.E-01	9.93.E-01
Siphoviridae	L6_g_butyrate.producing_bacterium	-0.016	0.038	6.71.E-01	9.80.E-01
Autographiviridae	L6_Parasutterella	0.000	0.038	9.90.E-01	9.99.E-01
crAss_like_phage	L6_Parasutterella	0.013	0.042	7.53.E-01	9.89.E-01
Herelleviridae	L6_Parasutterella	0.058	0.040	1.43.E-01	8.12.E-01
Microviridae	L6_Parasutterella	-0.074	0.040	6.16.E-02	7.24.E-01
Myoviridae	L6_Parasutterella	0.034	0.044	4.50.E-01	9.37.E-01
Phycodnaviridae	L6_Parasutterella	0.018	0.039	6.46.E-01	9.79.E-01
Podoviridae	L6_Parasutterella	-0.027	0.046	5.53.E-01	9.60.E-01
Siphoviridae	L6_Parasutterella	-0.024	0.041	5.65.E-01	9.61.E-01
Autographiviridae	L6_Hungatella	-0.006	0.029	8.50.E-01	9.95.E-01
crAss_like_phage	L6_Hungatella	0.014	0.032	6.55.E-01	9.79.E-01
Herelleviridae	L6_Hungatella	0.056	0.030	6.74.E-02	7.24.E-01
Microviridae	L6_Hungatella	-0.013	0.031	6.79.E-01	9.80.E-01
Myoviridae	L6_Hungatella	0.033	0.034	3.30.E-01	9.15.E-01
Phycodnaviridae	L6_Hungatella	0.056	0.030	6.04.E-02	7.24.E-01
Podoviridae	L6_Hungatella	-0.012	0.035	7.36.E-01	9.88.E-01
Siphoviridae	L6_Hungatella	-0.002	0.031	9.45.E-01	9.98.E-01
Autographiviridae	L6_Slackia	0.008	0.039	8.41.E-01	9.95.E-01
crAss_like_phage	L6_Slackia	0.007	0.044	8.79.E-01	9.96.E-01
Herelleviridae	L6_Slackia	-0.108	0.041	8.17.E-03	4.75.E-01
Microviridae	L6_Slackia	0.019	0.041	6.44.E-01	9.79.E-01
Myoviridae	L6_Slackia	-0.043	0.046	3.49.E-01	9.15.E-01
Phycodnaviridae	L6_Slackia	0.037	0.040	3.64.E-01	9.15.E-01

Podoviridae	L6_Slackia	0.011	0.047	8.22.E-01	9.93.E-01
Siphoviridae	L6_Slackia	-0.008	0.042	8.56.E-01	9.96.E-01
Autographiviridae	L6_Holdemanella	0.083	0.039	3.27.E-02	6.75.E-01
crAss_like_phage	L6_Holdemanella	0.042	0.043	3.33.E-01	9.15.E-01
Herelleviridae	L6_Holdemanella	0.008	0.040	8.34.E-01	9.94.E-01
Microviridae	L6_Holdemanella	0.068	0.040	9.10.E-02	7.59.E-01
Myoviridae	L6_Holdemanella	-0.029	0.045	5.14.E-01	9.57.E-01
Phycodnaviridae	L6_Holdemanella	-0.003	0.040	9.40.E-01	9.98.E-01
Podoviridae	L6_Holdemanella	-0.034	0.047	4.65.E-01	9.45.E-01
Siphoviridae	L6_Holdemanella	0.081	0.041	5.01.E-02	7.22.E-01
Autographiviridae	L6_Azospirillum	0.031	0.039	4.30.E-01	9.25.E-01
crAss_like_phage	L6_Azospirillum	-0.087	0.043	4.59.E-02	7.22.E-01
Herelleviridae	L6_Azospirillum	-0.040	0.041	3.25.E-01	9.15.E-01
Microviridae	L6_Azospirillum	-0.004	0.041	9.32.E-01	9.98.E-01
Myoviridae	L6_Azospirillum	-0.088	0.046	5.41.E-02	7.23.E-01
Phycodnaviridae	L6_Azospirillum	-0.037	0.040	3.64.E-01	9.15.E-01
Podoviridae	L6_Azospirillum	0.000	0.047	9.98.E-01	9.99.E-01
Siphoviridae	L6_Azospirillum	0.002	0.042	9.55.E-01	9.98.E-01
Autographiviridae	L6_Faecalicatena	0.030	0.033	3.69.E-01	9.15.E-01
crAss_like_phage	L6_Faecalicatena	0.034	0.037	3.58.E-01	9.15.E-01
Herelleviridae	L6_Faecalicatena	-0.040	0.034	2.51.E-01	8.95.E-01
Microviridae	L6_Faecalicatena	-0.058	0.034	9.31.E-02	7.60.E-01
Myoviridae	L6_Faecalicatena	0.027	0.039	4.82.E-01	9.53.E-01
Phycodnaviridae	L6_Faecalicatena	-0.015	0.034	6.54.E-01	9.79.E-01
Podoviridae	L6_Faecalicatena	0.014	0.040	7.35.E-01	9.88.E-01
Siphoviridae	L6_Faecalicatena	-0.031	0.035	3.76.E-01	9.15.E-01
Autographiviridae	L6_Gabonibacter	0.008	0.038	8.37.E-01	9.94.E-01
crAss_like_phage	L6_Gabonibacter	0.018	0.042	6.69.E-01	9.80.E-01
Herelleviridae	L6_Gabonibacter	0.022	0.039	5.69.E-01	9.61.E-01
Microviridae	L6_Gabonibacter	-0.019	0.039	6.22.E-01	9.76.E-01
Myoviridae	L6_Gabonibacter	-0.033	0.044	4.45.E-01	9.34.E-01
Phycodnaviridae	L6_Gabonibacter	0.057	0.038	1.41.E-01	8.10.E-01
Podoviridae	L6_Gabonibacter	-0.053	0.045	2.43.E-01	8.87.E-01
Siphoviridae	L6_Gabonibacter	0.025	0.040	5.37.E-01	9.60.E-01
Autographiviridae	L6_Peptoclostridium	-0.060	0.038	1.21.E-01	7.93.E-01
crAss_like_phage	L6_Peptoclostridium	-0.011	0.043	7.91.E-01	9.92.E-01
Herelleviridae	L6_Peptoclostridium	-0.042	0.040	2.89.E-01	9.15.E-01
Microviridae	L6_Peptoclostridium	-0.051	0.040	2.08.E-01	8.76.E-01
Myoviridae	L6_Peptoclostridium	0.036	0.045	4.27.E-01	9.24.E-01
Phycodnaviridae	L6_Peptoclostridium	0.015	0.040	6.97.E-01	9.81.E-01
Podoviridae	L6_Peptoclostridium	-0.036	0.046	4.31.E-01	9.25.E-01
Siphoviridae	L6_Peptoclostridium	-0.027	0.041	5.10.E-01	9.57.E-01
Autographiviridae	L6_Clostridioides	0.005	0.026	8.60.E-01	9.96.E-01
crAss_like_phage	L6_Clostridioides	-0.022	0.028	4.31.E-01	9.25.E-01
Herelleviridae	L6_Clostridioides	0.031	0.027	2.53.E-01	8.95.E-01
Microviridae	L6_Clostridioides	-0.056	0.027	3.55.E-02	6.90.E-01
Myoviridae	L6_Clostridioides	0.019	0.030	5.22.E-01	9.57.E-01
Phycodnaviridae	L6_Clostridioides	0.003	0.026	9.00.E-01	9.97.E-01
Podoviridae	L6_Clostridioides	-0.009	0.031	7.69.E-01	9.91.E-01
Siphoviridae	L6_Clostridioides	-0.002	0.028	9.49.E-01	9.98.E-01
Autographiviridae	L6_Gemella	-0.034	0.027	2.03.E-01	8.75.E-01
crAss_like_phage	L6_Gemella	0.046	0.030	1.23.E-01	7.94.E-01
Herelleviridae	L6_Gemella	-0.045	0.028	1.04.E-01	7.82.E-01
Microviridae	L6_Gemella	0.015	0.028	5.96.E-01	9.69.E-01
Myoviridae	L6_Gemella	-0.009	0.031	7.69.E-01	9.91.E-01
Phycodnaviridae	L6_Gemella	-0.023	0.028	4.10.E-01	9.17.E-01
Podoviridae	L6_Gemella	0.033	0.032	3.14.E-01	9.15.E-01
Siphoviridae	L6_Gemella	0.041	0.029	1.50.E-01	8.22.E-01
Autographiviridae	L6_g_Burkholderiales_bacterium	-0.005	0.038	8.91.E-01	9.96.E-01
crAss_like_phage	L6_g_Burkholderiales_bacterium	0.014	0.042	7.37.E-01	9.88.E-01
Herelleviridae	L6_g_Burkholderiales_bacterium	0.074	0.039	5.76.E-02	7.24.E-01
Microviridae	L6_g_Burkholderiales_bacterium	-0.045	0.039	2.49.E-01	8.94.E-01
Myoviridae	L6_g_Burkholderiales_bacterium	0.028	0.044	5.26.E-01	9.57.E-01
Phycodnaviridae	L6_g_Burkholderiales_bacterium	0.015	0.039	6.97.E-01	9.81.E-01
Podoviridae	L6_g_Burkholderiales_bacterium	-0.056	0.045	2.18.E-01	8.79.E-01

Siphoviridae	L6_g_Burkholderiales_bacterium	-0.026	0.040	5.18.E-01	9.57.E-01
Autographiviridae	L6_g_Oscillospiraceae_bacterium	0.014	0.031	6.50.E-01	9.79.E-01
crAss_like_phage	L6_g_Oscillospiraceae_bacterium	-0.014	0.034	6.89.E-01	9.80.E-01
Herelleviridae	L6_g_Oscillospiraceae_bacterium	-0.050	0.032	1.17.E-01	7.86.E-01
Microviridae	L6_g_Oscillospiraceae_bacterium	-0.017	0.032	6.06.E-01	9.73.E-01
Myoviridae	L6_g_Oscillospiraceae_bacterium	-0.060	0.036	9.19.E-02	7.59.E-01
Phycodnaviridae	L6_g_Oscillospiraceae_bacterium	-0.005	0.032	8.66.E-01	9.96.E-01
Podoviridae	L6_g_Oscillospiraceae_bacterium	0.032	0.037	3.88.E-01	9.15.E-01
Siphoviridae	L6_g_Oscillospiraceae_bacterium	0.054	0.033	1.00.E-01	7.79.E-01
Autographiviridae	L6_Absiella	-0.056	0.037	1.30.E-01	8.00.E-01
crAss_like_phage	L6_Absiella	0.027	0.041	5.10.E-01	9.57.E-01
Herelleviridae	L6_Absiella	-0.019	0.038	6.20.E-01	9.75.E-01
Microviridae	L6_Absiella	0.006	0.038	8.72.E-01	9.96.E-01
Myoviridae	L6_Absiella	0.000	0.043	9.93.E-01	9.99.E-01
Phycodnaviridae	L6_Absiella	-0.006	0.038	8.72.E-01	9.96.E-01
Podoviridae	L6_Absiella	-0.081	0.044	6.73.E-02	7.24.E-01
Siphoviridae	L6_Absiella	0.034	0.039	3.87.E-01	9.15.E-01
Autographiviridae	L6_Curtobacterium	0.006	0.011	5.78.E-01	9.64.E-01
crAss_like_phage	L6_Curtobacterium	0.003	0.012	7.74.E-01	9.92.E-01
Herelleviridae	L6_Curtobacterium	0.006	0.011	5.95.E-01	9.69.E-01
Microviridae	L6_Curtobacterium	-0.003	0.011	8.06.E-01	9.93.E-01
Myoviridae	L6_Curtobacterium	-0.011	0.012	3.93.E-01	9.15.E-01
Phycodnaviridae	L6_Curtobacterium	-0.003	0.011	7.95.E-01	9.92.E-01
Podoviridae	L6_Curtobacterium	-0.008	0.013	5.21.E-01	9.57.E-01
Siphoviridae	L6_Curtobacterium	-0.011	0.011	3.37.E-01	9.15.E-01
Autographiviridae	L6_Lactobacillus	-0.029	0.028	2.92.E-01	9.15.E-01
crAss_like_phage	L6_Lactobacillus	-0.009	0.031	7.60.E-01	9.91.E-01
Herelleviridae	L6_Lactobacillus	0.029	0.029	3.25.E-01	9.15.E-01
Microviridae	L6_Lactobacillus	-0.024	0.029	4.09.E-01	9.17.E-01
Myoviridae	L6_Lactobacillus	-0.017	0.032	5.96.E-01	9.69.E-01
Phycodnaviridae	L6_Lactobacillus	0.032	0.029	2.60.E-01	8.95.E-01
Podoviridae	L6_Lactobacillus	0.001	0.033	9.68.E-01	9.98.E-01
Siphoviridae	L6_Lactobacillus	0.039	0.030	1.93.E-01	8.62.E-01
Autographiviridae	L6_Eubacterium	0.031	0.033	3.48.E-01	9.15.E-01
crAss_like_phage	L6_Eubacterium	-0.050	0.036	1.68.E-01	8.44.E-01
Herelleviridae	L6_Eubacterium	0.046	0.034	1.80.E-01	8.60.E-01
Microviridae	L6_Eubacterium	-0.014	0.034	6.87.E-01	9.80.E-01
Myoviridae	L6_Eubacterium	-0.045	0.038	2.37.E-01	8.85.E-01
Phycodnaviridae	L6_Eubacterium	-0.006	0.034	8.51.E-01	9.95.E-01
Podoviridae	L6_Eubacterium	-0.128	0.039	1.05.E-03	2.79.E-01
Siphoviridae	L6_Eubacterium	0.001	0.035	9.83.E-01	9.99.E-01
Autographiviridae	L6_Butyricicoccus	-0.021	0.034	5.39.E-01	9.60.E-01
crAss_like_phage	L6_Butyricicoccus	-0.013	0.038	7.23.E-01	9.86.E-01
Herelleviridae	L6_Butyricicoccus	0.018	0.035	6.20.E-01	9.75.E-01
Microviridae	L6_Butyricicoccus	-0.024	0.036	4.93.E-01	9.55.E-01
Myoviridae	L6_Butyricicoccus	0.074	0.039	6.05.E-02	7.24.E-01
Phycodnaviridae	L6_Butyricicoccus	-0.007	0.035	8.38.E-01	9.94.E-01
Podoviridae	L6_Butyricicoccus	0.055	0.041	1.79.E-01	8.60.E-01
Siphoviridae	L6_Butyricicoccus	0.068	0.036	6.07.E-02	7.24.E-01
Autographiviridae	L6_Enorma	-0.023	0.038	5.49.E-01	9.60.E-01
crAss_like_phage	L6_Enorma	-0.047	0.042	2.62.E-01	9.00.E-01
Herelleviridae	L6_Enorma	-0.044	0.040	2.63.E-01	9.00.E-01
Microviridae	L6_Enorma	-0.042	0.040	2.91.E-01	9.15.E-01
Myoviridae	L6_Enorma	0.010	0.044	8.13.E-01	9.93.E-01
Phycodnaviridae	L6_Enorma	-0.072	0.039	6.52.E-02	7.24.E-01
Podoviridae	L6_Enorma	0.038	0.046	4.12.E-01	9.18.E-01
Siphoviridae	L6_Enorma	-0.100	0.041	1.38.E-02	5.51.E-01
Autographiviridae	L6_Dakarella	-0.056	0.045	2.12.E-01	8.78.E-01
crAss_like_phage	L6_Dakarella	0.043	0.050	3.92.E-01	9.15.E-01
Herelleviridae	L6_Dakarella	-0.039	0.047	4.07.E-01	9.17.E-01
Microviridae	L6_Dakarella	-0.042	0.047	3.71.E-01	9.15.E-01
Myoviridae	L6_Dakarella	0.053	0.052	3.13.E-01	9.15.E-01
Phycodnaviridae	L6_Dakarella	0.036	0.046	4.29.E-01	9.25.E-01
Podoviridae	L6_Dakarella	-0.128	0.054	1.76.E-02	5.86.E-01
Siphoviridae	L6_Dakarella	0.000	0.048	9.93.E-01	9.99.E-01

Autographiviridae	L6_Enterorhabdus	-0.009	0.035	7.91.E-01	9.92.E-01
crAss_like_phage	L6_Enterorhabdus	0.026	0.039	5.12.E-01	9.57.E-01
Herelleviridae	L6_Enterorhabdus	0.011	0.037	7.64.E-01	9.91.E-01
Microviridae	L6_Enterorhabdus	-0.020	0.037	5.81.E-01	9.64.E-01
Myoviridae	L6_Enterorhabdus	-0.061	0.041	1.33.E-01	8.05.E-01
Phycodnaviridae	L6_Enterorhabdus	0.086	0.036	1.75.E-02	5.86.E-01
Podoviridae	L6_Enterorhabdus	0.005	0.042	9.10.E-01	9.98.E-01
Siphoviridae	L6_Enterorhabdus	0.037	0.038	3.29.E-01	9.15.E-01
Autographiviridae	L6_Clostridium	0.007	0.032	8.23.E-01	9.93.E-01
crAss_like_phage	L6_Clostridium	0.009	0.035	8.05.E-01	9.93.E-01
Herelleviridae	L6_Clostridium	0.050	0.033	1.26.E-01	7.98.E-01
Microviridae	L6_Clostridium	0.011	0.033	7.40.E-01	9.88.E-01
Myoviridae	L6_Clostridium	0.011	0.037	7.67.E-01	9.91.E-01
Phycodnaviridae	L6_Clostridium	0.049	0.032	1.31.E-01	8.00.E-01
Podoviridae	L6_Clostridium	-0.008	0.038	8.23.E-01	9.93.E-01
Siphoviridae	L6_Clostridium	-0.024	0.034	4.86.E-01	9.54.E-01
Autographiviridae	L6_Pseudomonas	-0.052	0.039	1.85.E-01	8.61.E-01
crAss_like_phage	L6_Pseudomonas	-0.072	0.043	9.48.E-02	7.64.E-01
Herelleviridae	L6_Pseudomonas	-0.044	0.041	2.83.E-01	9.13.E-01
Microviridae	L6_Pseudomonas	0.033	0.041	4.27.E-01	9.24.E-01
Myoviridae	L6_Pseudomonas	0.002	0.046	9.63.E-01	9.98.E-01
Phycodnaviridae	L6_Pseudomonas	0.007	0.040	8.70.E-01	9.96.E-01
Podoviridae	L6_Pseudomonas	0.010	0.047	8.38.E-01	9.94.E-01
Siphoviridae	L6_Pseudomonas	-0.011	0.042	7.93.E-01	9.92.E-01
Autographiviridae	L6_Coprobacter	0.000	0.037	9.97.E-01	9.99.E-01
crAss_like_phage	L6_Coprobacter	-0.026	0.041	5.25.E-01	9.57.E-01
Herelleviridae	L6_Coprobacter	0.063	0.038	9.96.E-02	7.79.E-01
Microviridae	L6_Coprobacter	0.015	0.038	6.88.E-01	9.80.E-01
Myoviridae	L6_Coprobacter	0.020	0.043	6.33.E-01	9.76.E-01
Phycodnaviridae	L6_Coprobacter	0.013	0.038	7.34.E-01	9.88.E-01
Podoviridae	L6_Coprobacter	-0.020	0.044	6.51.E-01	9.79.E-01
Siphoviridae	L6_Coprobacter	-0.017	0.039	6.65.E-01	9.79.E-01
Autographiviridae	L6_Parabacteroides	-0.007	0.026	7.91.E-01	9.92.E-01
crAss_like_phage	L6_Parabacteroides	0.002	0.028	9.35.E-01	9.98.E-01
Herelleviridae	L6_Parabacteroides	0.005	0.027	8.64.E-01	9.96.E-01
Microviridae	L6_Parabacteroides	-0.022	0.027	4.21.E-01	9.20.E-01
Myoviridae	L6_Parabacteroides	-0.079	0.030	8.25.E-03	4.75.E-01
Phycodnaviridae	L6_Parabacteroides	0.024	0.026	3.67.E-01	9.15.E-01
Podoviridae	L6_Parabacteroides	0.028	0.031	3.64.E-01	9.15.E-01
Siphoviridae	L6_Parabacteroides	-0.004	0.027	8.98.E-01	9.97.E-01
Autographiviridae	L6_Corynebacterium	-0.004	0.036	9.02.E-01	9.97.E-01
crAss_like_phage	L6_Corynebacterium	0.076	0.040	5.89.E-02	7.24.E-01
Herelleviridae	L6_Corynebacterium	-0.007	0.038	8.50.E-01	9.95.E-01
Microviridae	L6_Corynebacterium	0.074	0.038	5.09.E-02	7.22.E-01
Myoviridae	L6_Corynebacterium	0.058	0.042	1.70.E-01	8.49.E-01
Phycodnaviridae	L6_Corynebacterium	-0.040	0.037	2.82.E-01	9.13.E-01
Podoviridae	L6_Corynebacterium	0.065	0.043	1.34.E-01	8.06.E-01
Siphoviridae	L6_Corynebacterium	0.003	0.039	9.33.E-01	9.98.E-01
Autographiviridae	L6_Catenibacterium	0.008	0.040	8.39.E-01	9.94.E-01
crAss_like_phage	L6_Catenibacterium	-0.017	0.044	7.05.E-01	9.82.E-01
Herelleviridae	L6_Catenibacterium	0.005	0.042	9.05.E-01	9.97.E-01
Microviridae	L6_Catenibacterium	0.021	0.042	6.10.E-01	9.75.E-01
Myoviridae	L6_Catenibacterium	0.043	0.046	3.53.E-01	9.15.E-01
Phycodnaviridae	L6_Catenibacterium	-0.039	0.041	3.38.E-01	9.15.E-01
Podoviridae	L6_Catenibacterium	0.011	0.048	8.20.E-01	9.93.E-01
Siphoviridae	L6_Catenibacterium	-0.057	0.043	1.81.E-01	8.60.E-01
Autographiviridae	L6_Dorea	0.044	0.035	2.13.E-01	8.78.E-01
crAss_like_phage	L6_Dorea	0.002	0.039	9.65.E-01	9.98.E-01
Herelleviridae	L6_Dorea	-0.124	0.036	6.77.E-04	2.55.E-01
Microviridae	L6_Dorea	-0.114	0.036	1.85.E-03	3.29.E-01
Myoviridae	L6_Dorea	-0.074	0.041	6.94.E-02	7.24.E-01
Phycodnaviridae	L6_Dorea	-0.043	0.036	2.42.E-01	8.85.E-01
Podoviridae	L6_Dorea	0.056	0.042	1.87.E-01	8.61.E-01
Siphoviridae	L6_Dorea	-0.039	0.038	2.99.E-01	9.15.E-01
Autographiviridae	L6_Erysipelatoclostridium	-0.017	0.030	5.58.E-01	9.61.E-01

crAss_like_phage	L6_Erysipelatoclostridium	0.033	0.033	3.19.E-01	9.15.E-01
Herelleviridae	L6_Erysipelatoclostridium	-0.014	0.031	6.55.E-01	9.79.E-01
Microviridae	L6_Erysipelatoclostridium	0.006	0.031	8.53.E-01	9.95.E-01
Myoviridae	L6_Erysipelatoclostridium	0.023	0.034	5.11.E-01	9.57.E-01
Phycodnaviridae	L6_Erysipelatoclostridium	-0.007	0.030	8.28.E-01	9.94.E-01
Podoviridae	L6_Erysipelatoclostridium	-0.025	0.036	4.84.E-01	9.54.E-01
Siphoviridae	L6_Erysipelatoclostridium	0.016	0.032	6.07.E-01	9.74.E-01
Autographiviridae	L6_Butyrivibrio	-0.006	0.033	8.60.E-01	9.96.E-01
crAss_like_phage	L6_Butyrivibrio	-0.013	0.037	7.25.E-01	9.87.E-01
Herelleviridae	L6_Butyrivibrio	0.004	0.035	9.06.E-01	9.97.E-01
Microviridae	L6_Butyrivibrio	-0.078	0.035	2.42.E-02	6.37.E-01
Myoviridae	L6_Butyrivibrio	-0.068	0.039	8.11.E-02	7.47.E-01
Phycodnaviridae	L6_Butyrivibrio	0.013	0.034	7.12.E-01	9.85.E-01
Podoviridae	L6_Butyrivibrio	-0.007	0.040	8.67.E-01	9.96.E-01
Siphoviridae	L6_Butyrivibrio	-0.046	0.036	2.01.E-01	8.74.E-01
Autographiviridae	L6_Bifidobacterium	-0.002	0.023	9.41.E-01	9.98.E-01
crAss_like_phage	L6_Bifidobacterium	-0.033	0.025	1.88.E-01	8.61.E-01
Herelleviridae	L6_Bifidobacterium	0.015	0.023	5.33.E-01	9.58.E-01
Microviridae	L6_Bifidobacterium	0.059	0.023	1.15.E-02	5.36.E-01
Myoviridae	L6_Bifidobacterium	0.025	0.026	3.33.E-01	9.15.E-01
Phycodnaviridae	L6_Bifidobacterium	0.008	0.023	7.28.E-01	9.88.E-01
Podoviridae	L6_Bifidobacterium	0.026	0.027	3.42.E-01	9.15.E-01
Siphoviridae	L6_Bifidobacterium	0.050	0.024	3.91.E-02	7.03.E-01
Autographiviridae	L6_Atopobium	-0.029	0.033	3.73.E-01	9.15.E-01
crAss_like_phage	L6_Atopobium	0.041	0.036	2.54.E-01	8.95.E-01
Herelleviridae	L6_Atopobium	-0.028	0.034	4.19.E-01	9.20.E-01
Microviridae	L6_Atopobium	-0.062	0.034	6.75.E-02	7.24.E-01
Myoviridae	L6_Atopobium	-0.019	0.038	6.12.E-01	9.75.E-01
Phycodnaviridae	L6_Atopobium	0.020	0.034	5.62.E-01	9.61.E-01
Podoviridae	L6_Atopobium	0.038	0.039	3.36.E-01	9.15.E-01
Siphoviridae	L6_Atopobium	-0.064	0.035	6.97.E-02	7.24.E-01
Autographiviridae	L6_Desulfovibrio	-0.005	0.036	8.83.E-01	9.96.E-01
crAss_like_phage	L6_Desulfovibrio	0.020	0.040	6.06.E-01	9.73.E-01
Herelleviridae	L6_Desulfovibrio	0.036	0.037	3.29.E-01	9.15.E-01
Microviridae	L6_Desulfovibrio	-0.014	0.037	7.13.E-01	9.85.E-01
Myoviridae	L6_Desulfovibrio	-0.024	0.042	5.60.E-01	9.61.E-01
Phycodnaviridae	L6_Desulfovibrio	-0.021	0.037	5.61.E-01	9.61.E-01
Podoviridae	L6_Desulfovibrio	-0.039	0.043	3.60.E-01	9.15.E-01
Siphoviridae	L6_Desulfovibrio	-0.057	0.038	1.40.E-01	8.10.E-01
Autographiviridae	L6_Faecalibacterium	-0.013	0.032	6.78.E-01	9.80.E-01
crAss_like_phage	L6_Faecalibacterium	-0.003	0.035	9.27.E-01	9.98.E-01
Herelleviridae	L6_Faecalibacterium	-0.040	0.033	2.32.E-01	8.82.E-01
Microviridae	L6_Faecalibacterium	0.057	0.033	8.74.E-02	7.52.E-01
Myoviridae	L6_Faecalibacterium	0.126	0.037	6.38.E-04	2.55.E-01
Phycodnaviridae	L6_Faecalibacterium	-0.002	0.033	9.56.E-01	9.98.E-01
Podoviridae	L6_Faecalibacterium	0.142	0.038	1.98.E-04	1.92.E-01
Siphoviridae	L6_Faecalibacterium	-0.008	0.034	8.12.E-01	9.93.E-01
Autographiviridae	L6_Roseburia	0.017	0.035	6.34.E-01	9.76.E-01
crAss_like_phage	L6_Roseburia	-0.015	0.039	7.02.E-01	9.81.E-01
Herelleviridae	L6_Roseburia	-0.045	0.037	2.18.E-01	8.79.E-01
Microviridae	L6_Roseburia	0.002	0.037	9.47.E-01	9.98.E-01
Myoviridae	L6_Roseburia	-0.100	0.041	1.44.E-02	5.53.E-01
Phycodnaviridae	L6_Roseburia	-0.052	0.036	1.51.E-01	8.22.E-01
Podoviridae	L6_Roseburia	-0.040	0.042	3.43.E-01	9.15.E-01
Siphoviridae	L6_Roseburia	-0.102	0.037	6.60.E-03	4.51.E-01
Autographiviridae	L6_Massilioclostridium	0.013	0.037	7.26.E-01	9.87.E-01
crAss_like_phage	L6_Massilioclostridium	0.067	0.041	1.01.E-01	7.79.E-01
Herelleviridae	L6_Massilioclostridium	-0.007	0.039	8.48.E-01	9.95.E-01
Microviridae	L6_Massilioclostridium	-0.037	0.039	3.36.E-01	9.15.E-01
Myoviridae	L6_Massilioclostridium	0.017	0.043	6.90.E-01	9.80.E-01
Phycodnaviridae	L6_Massilioclostridium	-0.024	0.038	5.29.E-01	9.57.E-01
Podoviridae	L6_Massilioclostridium	0.040	0.044	3.67.E-01	9.15.E-01
Siphoviridae	L6_Massilioclostridium	0.042	0.040	2.85.E-01	9.15.E-01
Autographiviridae	L6_Solobacterium	0.004	0.039	9.15.E-01	9.98.E-01
crAss_like_phage	L6_Solobacterium	0.031	0.043	4.74.E-01	9.52.E-01

Herelleviridae	L6_Solobacterium	-0.010	0.041	8.11.E-01	9.93.E-01
Microviridae	L6_Solobacterium	0.144	0.040	4.01.E-04	2.15.E-01
Myoviridae	L6_Solobacterium	0.069	0.046	1.29.E-01	7.99.E-01
Phycodnaviridae	L6_Solobacterium	0.026	0.040	5.21.E-01	9.57.E-01
Podoviridae	L6_Solobacterium	-0.009	0.047	8.57.E-01	9.96.E-01
Siphoviridae	L6_Solobacterium	-0.009	0.042	8.25.E-01	9.93.E-01
Autographiviridae	L6_Holdemania	-0.007	0.035	8.51.E-01	9.95.E-01
crAss_like_phage	L6_Holdemania	-0.062	0.039	1.10.E-01	7.82.E-01
Herelleviridae	L6_Holdemania	-0.010	0.037	7.77.E-01	9.92.E-01
Microviridae	L6_Holdemania	-0.001	0.037	9.81.E-01	9.99.E-01
Myoviridae	L6_Holdemania	-0.019	0.041	6.43.E-01	9.79.E-01
Phycodnaviridae	L6_Holdemania	0.019	0.036	5.96.E-01	9.69.E-01
Podoviridae	L6_Holdemania	0.052	0.042	2.19.E-01	8.79.E-01
Siphoviridae	L6_Holdemania	0.045	0.038	2.38.E-01	8.85.E-01
Autographiviridae	L6_Gordonibacter	0.030	0.036	3.98.E-01	9.15.E-01
crAss_like_phage	L6_Gordonibacter	-0.041	0.039	2.95.E-01	9.15.E-01
Herelleviridae	L6_Gordonibacter	0.014	0.037	7.11.E-01	9.85.E-01
Microviridae	L6_Gordonibacter	-0.026	0.037	4.80.E-01	9.53.E-01
Myoviridae	L6_Gordonibacter	-0.061	0.041	1.43.E-01	8.12.E-01
Phycodnaviridae	L6_Gordonibacter	0.017	0.037	6.44.E-01	9.79.E-01
Podoviridae	L6_Gordonibacter	0.009	0.043	8.31.E-01	9.94.E-01
Siphoviridae	L6_Gordonibacter	0.018	0.038	6.44.E-01	9.79.E-01
Autographiviridae	L6_Barnesiella	-0.080	0.035	2.13.E-02	6.06.E-01
crAss_like_phage	L6_Barnesiella	0.034	0.038	3.84.E-01	9.15.E-01
Herelleviridae	L6_Barnesiella	0.004	0.036	9.02.E-01	9.97.E-01
Microviridae	L6_Barnesiella	0.051	0.036	1.61.E-01	8.38.E-01
Myoviridae	L6_Barnesiella	-0.004	0.040	9.19.E-01	9.98.E-01
Phycodnaviridae	L6_Barnesiella	-0.007	0.036	8.36.E-01	9.94.E-01
Podoviridae	L6_Barnesiella	-0.065	0.042	1.21.E-01	7.93.E-01
Siphoviridae	L6_Barnesiella	0.051	0.037	1.67.E-01	8.44.E-01
Autographiviridae	L6_Bacillus	0.013	0.044	7.75.E-01	9.92.E-01
crAss_like_phage	L6_Bacillus	0.080	0.049	1.01.E-01	7.79.E-01
Herelleviridae	L6_Bacillus	-0.030	0.046	5.21.E-01	9.57.E-01
Microviridae	L6_Bacillus	0.092	0.046	4.69.E-02	7.22.E-01
Myoviridae	L6_Bacillus	0.108	0.051	3.55.E-02	6.90.E-01
Phycodnaviridae	L6_Bacillus	0.062	0.045	1.73.E-01	8.55.E-01
Podoviridae	L6_Bacillus	0.061	0.053	2.52.E-01	8.95.E-01
Siphoviridae	L6_Bacillus	0.093	0.047	4.82.E-02	7.22.E-01
Autographiviridae	L6_g_Bacteroidetes_bacterium	-0.045	0.040	2.69.E-01	9.01.E-01
crAss_like_phage	L6_g_Bacteroidetes_bacterium	-0.012	0.045	7.81.E-01	9.92.E-01
Herelleviridae	L6_g_Bacteroidetes_bacterium	0.018	0.042	6.59.E-01	9.79.E-01
Microviridae	L6_g_Bacteroidetes_bacterium	0.047	0.042	2.60.E-01	8.95.E-01
Myoviridae	L6_g_Bacteroidetes_bacterium	-0.032	0.047	4.99.E-01	9.55.E-01
Phycodnaviridae	L6_g_Bacteroidetes_bacterium	0.046	0.041	2.65.E-01	9.01.E-01
Podoviridae	L6_g_Bacteroidetes_bacterium	0.053	0.048	2.72.E-01	9.02.E-01
Siphoviridae	L6_g_Bacteroidetes_bacterium	0.036	0.043	4.08.E-01	9.17.E-01
Autographiviridae	L6_Senegalimassilia	-0.017	0.038	6.52.E-01	9.79.E-01
crAss_like_phage	L6_Senegalimassilia	-0.064	0.042	1.29.E-01	7.99.E-01
Herelleviridae	L6_Senegalimassilia	-0.057	0.039	1.52.E-01	8.24.E-01
Microviridae	L6_Senegalimassilia	0.035	0.040	3.78.E-01	9.15.E-01
Myoviridae	L6_Senegalimassilia	0.009	0.044	8.33.E-01	9.94.E-01
Phycodnaviridae	L6_Senegalimassilia	0.067	0.039	8.42.E-02	7.50.E-01
Podoviridae	L6_Senegalimassilia	-0.042	0.046	3.61.E-01	9.15.E-01
Siphoviridae	L6_Senegalimassilia	-0.019	0.041	6.35.E-01	9.76.E-01
Autographiviridae	L6_Propionibacterium	0.007	0.036	8.42.E-01	9.95.E-01
crAss_like_phage	L6_Propionibacterium	-0.056	0.040	1.58.E-01	8.35.E-01
Herelleviridae	L6_Propionibacterium	-0.026	0.037	4.82.E-01	9.53.E-01
Microviridae	L6_Propionibacterium	-0.049	0.037	1.92.E-01	8.61.E-01
Myoviridae	L6_Propionibacterium	0.002	0.042	9.65.E-01	9.98.E-01
Phycodnaviridae	L6_Propionibacterium	0.002	0.037	9.63.E-01	9.98.E-01
Podoviridae	L6_Propionibacterium	-0.038	0.043	3.74.E-01	9.15.E-01
Siphoviridae	L6_Propionibacterium	-0.022	0.038	5.66.E-01	9.61.E-01
Autographiviridae	L6_Acidaminococcus	0.001	0.044	9.75.E-01	9.99.E-01
crAss_like_phage	L6_Acidaminococcus	-0.097	0.048	4.46.E-02	7.22.E-01
Herelleviridae	L6_Acidaminococcus	-0.013	0.045	7.68.E-01	9.91.E-01

Microviridae	L6_Acidaminococcus	-0.013	0.045	7.79.E-01	9.92.E-01
Myoviridae	L6_Acidaminococcus	-0.007	0.051	8.85.E-01	9.96.E-01
Phycodnaviridae	L6_Acidaminococcus	0.035	0.045	4.39.E-01	9.30.E-01
Podoviridae	L6_Acidaminococcus	-0.027	0.052	6.07.E-01	9.74.E-01
Siphoviridae	L6_Acidaminococcus	0.006	0.047	9.06.E-01	9.97.E-01
Autographiviridae	L6_Escherichia	0.022	0.032	4.92.E-01	9.55.E-01
crAss_like_phage	L6_Escherichia	0.022	0.035	5.30.E-01	9.57.E-01
Herelleviridae	L6_Escherichia	0.014	0.033	6.63.E-01	9.79.E-01
Microviridae	L6_Escherichia	0.005	0.033	8.85.E-01	9.96.E-01
Myoviridae	L6_Escherichia	0.057	0.037	1.18.E-01	7.86.E-01
Phycodnaviridae	L6_Escherichia	0.000	0.033	9.93.E-01	9.99.E-01
Podoviridae	L6_Escherichia	-0.050	0.038	1.89.E-01	8.61.E-01
Siphoviridae	L6_Escherichia	0.029	0.034	3.85.E-01	9.15.E-01
Autographiviridae	L6_g_Eubacterium_siraeum	-0.025	0.033	4.64.E-01	9.45.E-01
crAss_like_phage	L6_g_Eubacterium_siraeum	0.061	0.037	9.83.E-02	7.78.E-01
Herelleviridae	L6_g_Eubacterium_siraeum	0.067	0.035	5.29.E-02	7.22.E-01
Microviridae	L6_g_Eubacterium_siraeum	0.013	0.035	7.13.E-01	9.85.E-01
Myoviridae	L6_g_Eubacterium_siraeum	0.053	0.039	1.75.E-01	8.56.E-01
Phycodnaviridae	L6_g_Eubacterium_siraeum	0.024	0.034	4.88.E-01	9.54.E-01
Podoviridae	L6_g_Eubacterium_siraeum	0.032	0.040	4.23.E-01	9.23.E-01
Siphoviridae	L6_g_Eubacterium_siraeum	-0.016	0.036	6.50.E-01	9.79.E-01
Autographiviridae	L6_Megamonas	-0.020	0.038	5.93.E-01	9.69.E-01
crAss_like_phage	L6_Megamonas	0.000	0.042	9.91.E-01	9.99.E-01
Herelleviridae	L6_Megamonas	-0.013	0.039	7.39.E-01	9.88.E-01
Microviridae	L6_Megamonas	-0.027	0.039	4.84.E-01	9.54.E-01
Myoviridae	L6_Megamonas	0.056	0.044	2.03.E-01	8.75.E-01
Phycodnaviridae	L6_Megamonas	-0.030	0.039	4.41.E-01	9.31.E-01
Podoviridae	L6_Megamonas	-0.034	0.045	4.56.E-01	9.39.E-01
Siphoviridae	L6_Megamonas	-0.038	0.040	3.38.E-01	9.15.E-01
Autographiviridae	L6_Granulicatella	-0.034	0.026	1.83.E-01	8.60.E-01
crAss_like_phage	L6_Granulicatella	0.031	0.028	2.67.E-01	9.01.E-01
Herelleviridae	L6_Granulicatella	-0.086	0.026	1.25.E-03	2.90.E-01
Microviridae	L6_Granulicatella	0.015	0.027	5.81.E-01	9.64.E-01
Myoviridae	L6_Granulicatella	-0.057	0.030	5.74.E-02	7.24.E-01
Phycodnaviridae	L6_Granulicatella	-0.027	0.026	3.10.E-01	9.15.E-01
Podoviridae	L6_Granulicatella	0.072	0.031	1.85.E-02	5.90.E-01
Siphoviridae	L6_Granulicatella	0.028	0.027	3.03.E-01	9.15.E-01
Autographiviridae	L6_Fournierella	-0.057	0.036	1.10.E-01	7.82.E-01
crAss_like_phage	L6_Fournierella	-0.034	0.040	3.89.E-01	9.15.E-01
Herelleviridae	L6_Fournierella	-0.013	0.037	7.36.E-01	9.88.E-01
Microviridae	L6_Fournierella	-0.009	0.037	8.13.E-01	9.93.E-01
Myoviridae	L6_Fournierella	0.006	0.042	8.82.E-01	9.96.E-01
Phycodnaviridae	L6_Fournierella	-0.026	0.037	4.76.E-01	9.52.E-01
Podoviridae	L6_Fournierella	0.016	0.043	7.14.E-01	9.86.E-01
Siphoviridae	L6_Fournierella	0.026	0.038	5.07.E-01	9.57.E-01
Autographiviridae	L6_g_Clostridiales_bacterium	0.022	0.027	4.20.E-01	9.20.E-01
crAss_like_phage	L6_g_Clostridiales_bacterium	0.027	0.030	3.59.E-01	9.15.E-01
Herelleviridae	L6_g_Clostridiales_bacterium	0.012	0.028	6.61.E-01	9.79.E-01
Microviridae	L6_g_Clostridiales_bacterium	-0.016	0.028	5.79.E-01	9.64.E-01
Myoviridae	L6_g_Clostridiales_bacterium	0.092	0.031	3.14.E-03	3.87.E-01
Phycodnaviridae	L6_g_Clostridiales_bacterium	-0.017	0.028	5.46.E-01	9.60.E-01
Podoviridae	L6_g_Clostridiales_bacterium	-0.081	0.032	1.20.E-02	5.36.E-01
Siphoviridae	L6_g_Clostridiales_bacterium	0.036	0.029	2.08.E-01	8.76.E-01
Autographiviridae	L6_Aeromonas	-0.017	0.041	6.81.E-01	9.80.E-01
crAss_like_phage	L6_Aeromonas	0.021	0.045	6.42.E-01	9.79.E-01
Herelleviridae	L6_Aeromonas	-0.085	0.042	4.46.E-02	7.22.E-01
Microviridae	L6_Aeromonas	0.021	0.042	6.27.E-01	9.76.E-01
Myoviridae	L6_Aeromonas	0.012	0.047	7.99.E-01	9.92.E-01
Phycodnaviridae	L6_Aeromonas	-0.060	0.042	1.50.E-01	8.22.E-01
Podoviridae	L6_Aeromonas	-0.084	0.049	8.67.E-02	7.52.E-01
Siphoviridae	L6_Aeromonas	0.017	0.044	6.93.E-01	9.81.E-01
Autographiviridae	L6_Faecalicoccus	-0.035	0.037	3.49.E-01	9.15.E-01
crAss_like_phage	L6_Faecalicoccus	0.001	0.041	9.85.E-01	9.99.E-01
Herelleviridae	L6_Faecalicoccus	-0.034	0.038	3.75.E-01	9.15.E-01
Microviridae	L6_Faecalicoccus	-0.045	0.038	2.38.E-01	8.85.E-01

Myoviridae	L6_Faecalicoccus	-0.057	0.043	1.85.E-01	8.61.E-01
Phycodnaviridae	L6_Faecalicoccus	-0.037	0.038	3.29.E-01	9.15.E-01
Podoviridae	L6_Faecalicoccus	-0.010	0.044	8.20.E-01	9.93.E-01
Siphoviridae	L6_Faecalicoccus	-0.003	0.039	9.44.E-01	9.98.E-01
Autographiviridae	L6_Catonella	0.023	0.043	5.91.E-01	9.68.E-01
crAss_like_phage	L6_Catonella	-0.060	0.048	2.09.E-01	8.76.E-01
Herelleviridae	L6_Catonella	-0.014	0.045	7.54.E-01	9.89.E-01
Microviridae	L6_Catonella	0.004	0.045	9.29.E-01	9.98.E-01
Myoviridae	L6_Catonella	0.060	0.050	2.29.E-01	8.82.E-01
Phycodnaviridae	L6_Catonella	0.033	0.044	4.60.E-01	9.43.E-01
Podoviridae	L6_Catonella	0.149	0.051	3.93.E-03	4.05.E-01
Siphoviridae	L6_Catonella	0.055	0.046	2.32.E-01	8.82.E-01
Autographiviridae	L6_Anaerobutyricum	0.003	0.037	9.36.E-01	9.98.E-01
crAss_like_phage	L6_Anaerobutyricum	-0.038	0.041	3.63.E-01	9.15.E-01
Herelleviridae	L6_Anaerobutyricum	0.061	0.039	1.14.E-01	7.85.E-01
Microviridae	L6_Anaerobutyricum	0.031	0.039	4.31.E-01	9.25.E-01
Myoviridae	L6_Anaerobutyricum	0.000	0.043	9.93.E-01	9.99.E-01
Phycodnaviridae	L6_Anaerobutyricum	0.022	0.038	5.72.E-01	9.61.E-01
Podoviridae	L6_Anaerobutyricum	-0.048	0.045	2.88.E-01	9.15.E-01
Siphoviridae	L6_Anaerobutyricum	-0.009	0.040	8.31.E-01	9.94.E-01
Autographiviridae	L6_Acetobacter	0.044	0.041	2.87.E-01	9.15.E-01
crAss_like_phage	L6_Acetobacter	-0.102	0.045	2.49.E-02	6.47.E-01
Herelleviridae	L6_Acetobacter	-0.019	0.043	6.54.E-01	9.79.E-01
Microviridae	L6_Acetobacter	0.029	0.043	5.01.E-01	9.55.E-01
Myoviridae	L6_Acetobacter	0.017	0.048	7.23.E-01	9.86.E-01
Phycodnaviridae	L6_Acetobacter	-0.024	0.042	5.75.E-01	9.62.E-01
Podoviridae	L6_Acetobacter	-0.091	0.049	6.53.E-02	7.24.E-01
Siphoviridae	L6_Acetobacter	-0.018	0.044	6.89.E-01	9.80.E-01
Autographiviridae	L6_Oxalobacter	-0.064	0.035	6.63.E-02	7.24.E-01
crAss_like_phage	L6_Oxalobacter	0.002	0.039	9.48.E-01	9.98.E-01
Herelleviridae	L6_Oxalobacter	-0.061	0.036	9.33.E-02	7.60.E-01
Microviridae	L6_Oxalobacter	0.038	0.036	3.01.E-01	9.15.E-01
Myoviridae	L6_Oxalobacter	0.002	0.040	9.54.E-01	9.98.E-01
Phycodnaviridae	L6_Oxalobacter	-0.052	0.036	1.50.E-01	8.22.E-01
Podoviridae	L6_Oxalobacter	-0.047	0.042	2.64.E-01	9.01.E-01
Siphoviridae	L6_Oxalobacter	-0.039	0.037	2.98.E-01	9.15.E-01
Autographiviridae	L6_Tannerella	0.010	0.025	6.92.E-01	9.80.E-01
crAss_like_phage	L6_Tannerella	0.035	0.028	2.08.E-01	8.76.E-01
Herelleviridae	L6_Tannerella	0.047	0.026	7.18.E-02	7.25.E-01
Microviridae	L6_Tannerella	0.025	0.026	3.39.E-01	9.15.E-01
Myoviridae	L6_Tannerella	0.040	0.029	1.74.E-01	8.56.E-01
Phycodnaviridae	L6_Tannerella	-0.006	0.026	8.08.E-01	9.93.E-01
Podoviridae	L6_Tannerella	-0.001	0.030	9.66.E-01	9.98.E-01
Siphoviridae	L6_Tannerella	0.013	0.027	6.24.E-01	9.76.E-01
Autographiviridae	L6_Pseudoflavonifractor	-0.016	0.027	5.37.E-01	9.60.E-01
crAss_like_phage	L6_Pseudoflavonifractor	-0.064	0.029	2.94.E-02	6.66.E-01
Herelleviridae	L6_Pseudoflavonifractor	-0.004	0.028	8.92.E-01	9.96.E-01
Microviridae	L6_Pseudoflavonifractor	-0.006	0.028	8.37.E-01	9.94.E-01
Myoviridae	L6_Pseudoflavonifractor	-0.005	0.031	8.67.E-01	9.96.E-01
Phycodnaviridae	L6_Pseudoflavonifractor	-0.053	0.027	5.28.E-02	7.22.E-01
Podoviridae	L6_Pseudoflavonifractor	-0.028	0.032	3.81.E-01	9.15.E-01
Siphoviridae	L6_Pseudoflavonifractor	-0.017	0.029	5.50.E-01	9.60.E-01
Autographiviridae	L6_Caecibacter	0.006	0.044	8.85.E-01	9.96.E-01
crAss_like_phage	L6_Caecibacter	-0.031	0.049	5.20.E-01	9.57.E-01
Herelleviridae	L6_Caecibacter	-0.089	0.046	5.10.E-02	7.22.E-01
Microviridae	L6_Caecibacter	0.004	0.046	9.38.E-01	9.98.E-01
Myoviridae	L6_Caecibacter	0.018	0.051	7.25.E-01	9.87.E-01
Phycodnaviridae	L6_Caecibacter	-0.044	0.045	3.33.E-01	9.15.E-01
Podoviridae	L6_Caecibacter	-0.060	0.053	2.54.E-01	8.95.E-01
Siphoviridae	L6_Caecibacter	0.004	0.047	9.35.E-01	9.98.E-01
Autographiviridae	L6_Lactococcus	0.057	0.040	1.55.E-01	8.26.E-01
crAss_like_phage	L6_Lactococcus	-0.023	0.044	6.05.E-01	9.73.E-01
Herelleviridae	L6_Lactococcus	0.004	0.042	9.14.E-01	9.98.E-01
Microviridae	L6_Lactococcus	0.040	0.042	3.40.E-01	9.15.E-01
Myoviridae	L6_Lactococcus	0.020	0.046	6.73.E-01	9.80.E-01

Phycodnaviridae	L6_Lactococcus	-0.018	0.041	6.61.E-01	9.79.E-01
Podoviridae	L6_Lactococcus	0.031	0.048	5.21.E-01	9.57.E-01
Siphoviridae	L6_Lactococcus	-0.023	0.043	5.97.E-01	9.69.E-01
Autographiviridae	L6_Tyzzerella	-0.015	0.037	6.76.E-01	9.80.E-01
crAss_like_phage	L6_Tyzzerella	-0.006	0.041	8.89.E-01	9.96.E-01
Herelleviridae	L6_Tyzzerella	0.071	0.038	6.23.E-02	7.24.E-01
Microviridae	L6_Tyzzerella	0.004	0.038	9.09.E-01	9.98.E-01
Myoviridae	L6_Tyzzerella	-0.007	0.043	8.67.E-01	9.96.E-01
Phycodnaviridae	L6_Tyzzerella	-0.030	0.038	4.31.E-01	9.25.E-01
Podoviridae	L6_Tyzzerella	0.024	0.044	5.94.E-01	9.69.E-01
Siphoviridae	L6_Tyzzerella	0.018	0.039	6.53.E-01	9.79.E-01
Autographiviridae	L6_Rikenella	0.041	0.029	1.56.E-01	8.30.E-01
crAss_like_phage	L6_Rikenella	-0.012	0.032	6.98.E-01	9.81.E-01
Herelleviridae	L6_Rikenella	0.025	0.030	4.14.E-01	9.18.E-01
Microviridae	L6_Rikenella	0.003	0.030	9.24.E-01	9.98.E-01
Myoviridae	L6_Rikenella	-0.009	0.034	7.85.E-01	9.92.E-01
Phycodnaviridae	L6_Rikenella	-0.037	0.030	2.19.E-01	8.79.E-01
Podoviridae	L6_Rikenella	-0.024	0.035	5.00.E-01	9.55.E-01
Siphoviridae	L6_Rikenella	-0.057	0.031	6.35.E-02	7.24.E-01
Autographiviridae	L6_Eggerthella	-0.001	0.037	9.89.E-01	9.99.E-01
crAss_like_phage	L6_Eggerthella	-0.037	0.040	3.58.E-01	9.15.E-01
Herelleviridae	L6_Eggerthella	0.042	0.038	2.74.E-01	9.04.E-01
Microviridae	L6_Eggerthella	-0.025	0.038	5.16.E-01	9.57.E-01
Myoviridae	L6_Eggerthella	-0.049	0.042	2.49.E-01	8.94.E-01
Phycodnaviridae	L6_Eggerthella	0.010	0.038	7.96.E-01	9.92.E-01
Podoviridae	L6_Eggerthella	0.058	0.044	1.83.E-01	8.60.E-01
Siphoviridae	L6_Eggerthella	-0.097	0.039	1.27.E-02	5.43.E-01
Autographiviridae	L6_Adlercreutzia	0.014	0.036	6.97.E-01	9.81.E-01
crAss_like_phage	L6_Adlercreutzia	-0.039	0.039	3.27.E-01	9.15.E-01
Herelleviridae	L6_Adlercreutzia	-0.014	0.037	7.15.E-01	9.86.E-01
Microviridae	L6_Adlercreutzia	-0.006	0.037	8.78.E-01	9.96.E-01
Myoviridae	L6_Adlercreutzia	-0.015	0.041	7.12.E-01	9.85.E-01
Phycodnaviridae	L6_Adlercreutzia	0.035	0.037	3.35.E-01	9.15.E-01
Podoviridae	L6_Adlercreutzia	-0.023	0.043	5.90.E-01	9.68.E-01
Siphoviridae	L6_Adlercreutzia	0.056	0.038	1.44.E-01	8.12.E-01
Autographiviridae	L6_Asaccharobacter	0.006	0.035	8.60.E-01	9.96.E-01
crAss_like_phage	L6_Asaccharobacter	0.010	0.038	7.98.E-01	9.92.E-01
Herelleviridae	L6_Asaccharobacter	0.026	0.036	4.74.E-01	9.52.E-01
Microviridae	L6_Asaccharobacter	-0.029	0.036	4.25.E-01	9.23.E-01
Myoviridae	L6_Asaccharobacter	-0.005	0.040	9.05.E-01	9.97.E-01
Phycodnaviridae	L6_Asaccharobacter	0.023	0.036	5.12.E-01	9.57.E-01
Podoviridae	L6_Asaccharobacter	-0.021	0.042	6.20.E-01	9.75.E-01
Siphoviridae	L6_Asaccharobacter	0.078	0.037	3.48.E-02	6.89.E-01
Autographiviridae	L6_Anaerotignum	-0.006	0.041	8.83.E-01	9.96.E-01
crAss_like_phage	L6_Anaerotignum	-0.044	0.045	3.35.E-01	9.15.E-01
Herelleviridae	L6_Anaerotignum	-0.029	0.043	4.94.E-01	9.55.E-01
Microviridae	L6_Anaerotignum	0.009	0.043	8.38.E-01	9.94.E-01
Myoviridae	L6_Anaerotignum	0.084	0.048	7.96.E-02	7.43.E-01
Phycodnaviridae	L6_Anaerotignum	0.015	0.042	7.14.E-01	9.86.E-01
Podoviridae	L6_Anaerotignum	-0.048	0.049	3.29.E-01	9.15.E-01
Siphoviridae	L6_Anaerotignum	-0.004	0.044	9.30.E-01	9.98.E-01
Autographiviridae	L6_Enterococcus	0.065	0.036	7.15.E-02	7.24.E-01
crAss_like_phage	L6_Enterococcus	-0.049	0.040	2.21.E-01	8.79.E-01
Herelleviridae	L6_Enterococcus	0.030	0.038	4.18.E-01	9.19.E-01
Microviridae	L6_Enterococcus	-0.016	0.038	6.75.E-01	9.80.E-01
Myoviridae	L6_Enterococcus	0.098	0.042	1.94.E-02	5.90.E-01
Phycodnaviridae	L6_Enterococcus	0.002	0.037	9.55.E-01	9.98.E-01
Podoviridae	L6_Enterococcus	0.047	0.043	2.77.E-01	9.05.E-01
Siphoviridae	L6_Enterococcus	-0.057	0.039	1.42.E-01	8.10.E-01
Autographiviridae	L6_Eisenbergiella	-0.036	0.033	2.79.E-01	9.10.E-01
crAss_like_phage	L6_Eisenbergiella	-0.026	0.037	4.86.E-01	9.54.E-01
Herelleviridae	L6_Eisenbergiella	0.019	0.035	5.89.E-01	9.68.E-01
Microviridae	L6_Eisenbergiella	0.045	0.035	1.92.E-01	8.61.E-01
Myoviridae	L6_Eisenbergiella	-0.013	0.039	7.44.E-01	9.89.E-01
Phycodnaviridae	L6_Eisenbergiella	0.029	0.034	4.01.E-01	9.15.E-01

Podoviridae	L6_Eisenbergiella	0.034	0.040	3.92.E-01	9.15.E-01
Siphoviridae	L6_Eisenbergiella	0.001	0.036	9.88.E-01	9.99.E-01
Autographiviridae	L6_Prevotella	-0.010	0.032	7.58.E-01	9.90.E-01
crAss_like_phage	L6_Prevotella	0.009	0.036	7.99.E-01	9.92.E-01
Herelleviridae	L6_Prevotella	0.006	0.034	8.54.E-01	9.96.E-01
Microviridae	L6_Prevotella	-0.005	0.034	8.89.E-01	9.96.E-01
Myoviridae	L6_Prevotella	-0.027	0.038	4.73.E-01	9.52.E-01
Phycodnaviridae	L6_Prevotella	-0.016	0.033	6.26.E-01	9.76.E-01
Podoviridae	L6_Prevotella	0.017	0.039	6.70.E-01	9.80.E-01
Siphoviridae	L6_Prevotella	0.029	0.035	4.04.E-01	9.16.E-01
Autographiviridae	L6_Streptococcus	-0.009	0.025	7.09.E-01	9.84.E-01
crAss_like_phage	L6_Streptococcus	0.022	0.028	4.30.E-01	9.25.E-01
Herelleviridae	L6_Streptococcus	0.032	0.026	2.23.E-01	8.80.E-01
Microviridae	L6_Streptococcus	0.024	0.026	3.54.E-01	9.15.E-01
Myoviridae	L6_Streptococcus	0.027	0.029	3.52.E-01	9.15.E-01
Phycodnaviridae	L6_Streptococcus	0.001	0.026	9.81.E-01	9.99.E-01
Podoviridae	L6_Streptococcus	0.033	0.030	2.71.E-01	9.02.E-01
Siphoviridae	L6_Streptococcus	0.039	0.027	1.43.E-01	8.12.E-01
Autographiviridae	L6_Dialister	0.018	0.044	6.84.E-01	9.80.E-01
crAss_like_phage	L6_Dialister	-0.017	0.049	7.22.E-01	9.86.E-01
Herelleviridae	L6_Dialister	-0.073	0.046	1.14.E-01	7.85.E-01
Microviridae	L6_Dialister	-0.023	0.046	6.23.E-01	9.76.E-01
Myoviridae	L6_Dialister	-0.048	0.052	3.57.E-01	9.15.E-01
Phycodnaviridae	L6_Dialister	-0.019	0.046	6.82.E-01	9.80.E-01
Podoviridae	L6_Dialister	0.016	0.053	7.70.E-01	9.91.E-01
Siphoviridae	L6_Dialister	-0.004	0.047	9.34.E-01	9.98.E-01
Autographiviridae	L6_Brachyspira	-0.032	0.039	4.17.E-01	9.19.E-01
crAss_like_phage	L6_Brachyspira	-0.034	0.043	4.30.E-01	9.25.E-01
Herelleviridae	L6_Brachyspira	-0.005	0.041	8.95.E-01	9.97.E-01
Microviridae	L6_Brachyspira	0.054	0.041	1.87.E-01	8.61.E-01
Myoviridae	L6_Brachyspira	-0.021	0.045	6.47.E-01	9.79.E-01
Phycodnaviridae	L6_Brachyspira	0.021	0.040	6.02.E-01	9.71.E-01
Podoviridae	L6_Brachyspira	-0.060	0.047	1.97.E-01	8.66.E-01
Siphoviridae	L6_Brachyspira	-0.018	0.042	6.68.E-01	9.80.E-01
Autographiviridae	L6_Akkermansia	-0.009	0.039	8.18.E-01	9.93.E-01
crAss_like_phage	L6_Akkermansia	0.013	0.043	7.69.E-01	9.91.E-01
Herelleviridae	L6_Akkermansia	0.024	0.040	5.51.E-01	9.60.E-01
Microviridae	L6_Akkermansia	-0.074	0.040	6.67.E-02	7.24.E-01
Myoviridae	L6_Akkermansia	0.041	0.045	3.64.E-01	9.15.E-01
Phycodnaviridae	L6_Akkermansia	0.054	0.040	1.77.E-01	8.58.E-01
Podoviridae	L6_Akkermansia	0.092	0.046	4.68.E-02	7.22.E-01
Siphoviridae	L6_Akkermansia	-0.004	0.041	9.28.E-01	9.98.E-01
Autographiviridae	L6_Faecalitalea	-0.018	0.034	6.03.E-01	9.72.E-01
crAss_like_phage	L6_Faecalitalea	0.017	0.038	6.44.E-01	9.79.E-01
Herelleviridae	L6_Faecalitalea	0.011	0.035	7.47.E-01	9.89.E-01
Microviridae	L6_Faecalitalea	-0.031	0.035	3.82.E-01	9.15.E-01
Myoviridae	L6_Faecalitalea	-0.016	0.039	6.77.E-01	9.80.E-01
Phycodnaviridae	L6_Faecalitalea	-0.001	0.035	9.76.E-01	9.99.E-01
Podoviridae	L6_Faecalitalea	-0.066	0.041	1.04.E-01	7.82.E-01
Siphoviridae	L6_Faecalitalea	-0.051	0.036	1.63.E-01	8.38.E-01
Autographiviridae	L6_Phascolarctobacterium	0.023	0.039	5.67.E-01	9.61.E-01
crAss_like_phage	L6_Phascolarctobacterium	0.079	0.044	7.07.E-02	7.24.E-01
Herelleviridae	L6_Phascolarctobacterium	0.035	0.041	3.94.E-01	9.15.E-01
Microviridae	L6_Phascolarctobacterium	0.035	0.041	3.91.E-01	9.15.E-01
Myoviridae	L6_Phascolarctobacterium	0.159	0.045	4.94.E-04	2.44.E-01
Phycodnaviridae	L6_Phascolarctobacterium	0.054	0.041	1.79.E-01	8.60.E-01
Podoviridae	L6_Phascolarctobacterium	0.110	0.047	1.96.E-02	5.90.E-01
Siphoviridae	L6_Phascolarctobacterium	0.053	0.042	2.10.E-01	8.76.E-01
Autographiviridae	L6_Emergencia	0.038	0.036	2.99.E-01	9.15.E-01
crAss_like_phage	L6_Emergencia	0.010	0.040	7.96.E-01	9.92.E-01
Herelleviridae	L6_Emergencia	-0.026	0.038	4.83.E-01	9.54.E-01
Microviridae	L6_Emergencia	0.022	0.038	5.63.E-01	9.61.E-01
Myoviridae	L6_Emergencia	0.024	0.042	5.61.E-01	9.61.E-01
Phycodnaviridae	L6_Emergencia	0.028	0.037	4.59.E-01	9.43.E-01
Podoviridae	L6_Emergencia	0.017	0.043	6.97.E-01	9.81.E-01

Siphoviridae	L6_Emergencia	0.062	0.039	1.08.E-01	7.82.E-01
Autographiviridae	L7_Clostridium_dakarense	0.024	0.028	3.84.E-01	9.15.E-01
crAss_like_phage	L7_Clostridium_dakarense	0.029	0.031	3.45.E-01	9.15.E-01
Herelleviridae	L7_Clostridium_dakarense	0.033	0.029	2.54.E-01	8.95.E-01
Microviridae	L7_Clostridium_dakarense	-0.027	0.029	3.55.E-01	9.15.E-01
Myoviridae	L7_Clostridium_dakarense	-0.071	0.032	2.73.E-02	6.58.E-01
Phycodnaviridae	L7_Clostridium_dakarense	-0.001	0.029	9.70.E-01	9.99.E-01
Podoviridae	L7_Clostridium_dakarense	0.014	0.034	6.77.E-01	9.80.E-01
Siphoviridae	L7_Clostridium_dakarense	0.034	0.030	2.56.E-01	8.95.E-01
Autographiviridae	L7_Actinomyces_oris	-0.025	0.037	5.01.E-01	9.55.E-01
crAss_like_phage	L7_Actinomyces_oris	0.014	0.041	7.33.E-01	9.88.E-01
Herelleviridae	L7_Actinomyces_oris	-0.063	0.038	1.00.E-01	7.79.E-01
Microviridae	L7_Actinomyces_oris	-0.007	0.038	8.61.E-01	9.96.E-01
Myoviridae	L7_Actinomyces_oris	0.023	0.043	5.91.E-01	9.68.E-01
Phycodnaviridae	L7_Actinomyces_oris	-0.011	0.038	7.77.E-01	9.92.E-01
Podoviridae	L7_Actinomyces_oris	-0.077	0.044	8.16.E-02	7.47.E-01
Siphoviridae	L7_Actinomyces_oris	-0.022	0.039	5.74.E-01	9.62.E-01
Autographiviridae	L7_Clostridium_innocuum	0.016	0.031	5.95.E-01	9.69.E-01
crAss_like_phage	L7_Clostridium_innocuum	0.022	0.034	5.08.E-01	9.57.E-01
Herelleviridae	L7_Clostridium_innocuum	0.027	0.032	3.91.E-01	9.15.E-01
Microviridae	L7_Clostridium_innocuum	0.003	0.032	9.25.E-01	9.98.E-01
Myoviridae	L7_Clostridium_innocuum	-0.037	0.036	3.02.E-01	9.15.E-01
Phycodnaviridae	L7_Clostridium_innocuum	-0.020	0.031	5.28.E-01	9.57.E-01
Podoviridae	L7_Clostridium_innocuum	0.025	0.037	5.00.E-01	9.55.E-01
Siphoviridae	L7_Clostridium_innocuum	0.017	0.033	6.00.E-01	9.71.E-01
Autographiviridae	L7_Clostridiaceae_bacterium	0.008	0.027	7.60.E-01	9.91.E-01
crAss_like_phage	L7_Clostridiaceae_bacterium	-0.017	0.030	5.64.E-01	9.61.E-01
Herelleviridae	L7_Clostridiaceae_bacterium	-0.015	0.028	6.03.E-01	9.72.E-01
Microviridae	L7_Clostridiaceae_bacterium	0.010	0.028	7.35.E-01	9.88.E-01
Myoviridae	L7_Clostridiaceae_bacterium	0.029	0.032	3.59.E-01	9.15.E-01
Phycodnaviridae	L7_Clostridiaceae_bacterium	-0.059	0.028	3.37.E-02	6.82.E-01
Podoviridae	L7_Clostridiaceae_bacterium	-0.024	0.033	4.55.E-01	9.39.E-01
Siphoviridae	L7_Clostridiaceae_bacterium	0.028	0.029	3.41.E-01	9.15.E-01
Autographiviridae	L7_Coprobacter_fastidiosus	0.000	0.037	9.97.E-01	9.99.E-01
crAss_like_phage	L7_Coprobacter_fastidiosus	-0.026	0.041	5.25.E-01	9.57.E-01
Herelleviridae	L7_Coprobacter_fastidiosus	0.063	0.038	9.96.E-02	7.79.E-01
Microviridae	L7_Coprobacter_fastidiosus	0.015	0.038	6.88.E-01	9.80.E-01
Myoviridae	L7_Coprobacter_fastidiosus	0.020	0.043	6.33.E-01	9.76.E-01
Phycodnaviridae	L7_Coprobacter_fastidiosus	0.013	0.038	7.34.E-01	9.88.E-01
Podoviridae	L7_Coprobacter_fastidiosus	-0.020	0.044	6.51.E-01	9.79.E-01
Siphoviridae	L7_Coprobacter_fastidiosus	-0.017	0.039	6.65.E-01	9.79.E-01
Autographiviridae	L7_Roseburia_sp.	0.002	0.036	9.66.E-01	9.98.E-01
crAss_like_phage	L7_Roseburia_sp.	-0.011	0.040	7.75.E-01	9.92.E-01
Herelleviridae	L7_Roseburia_sp.	-0.031	0.037	4.05.E-01	9.17.E-01
Microviridae	L7_Roseburia_sp.	-0.007	0.037	8.42.E-01	9.95.E-01
Myoviridae	L7_Roseburia_sp.	-0.062	0.042	1.38.E-01	8.10.E-01
Phycodnaviridae	L7_Roseburia_sp.	-0.067	0.037	7.13.E-02	7.24.E-01
Podoviridae	L7_Roseburia_sp.	-0.027	0.043	5.26.E-01	9.57.E-01
Siphoviridae	L7_Roseburia_sp.	-0.130	0.038	6.50.E-04	2.55.E-01
Autographiviridae	L7_Bifidobacterium_breve	0.027	0.031	3.81.E-01	9.15.E-01
crAss_like_phage	L7_Bifidobacterium_breve	0.006	0.034	8.52.E-01	9.95.E-01
Herelleviridae	L7_Bifidobacterium_breve	0.001	0.032	9.77.E-01	9.99.E-01
Microviridae	L7_Bifidobacterium_breve	0.069	0.032	3.12.E-02	6.70.E-01
Myoviridae	L7_Bifidobacterium_breve	-0.023	0.036	5.15.E-01	9.57.E-01
Phycodnaviridae	L7_Bifidobacterium_breve	0.031	0.032	3.22.E-01	9.15.E-01
Podoviridae	L7_Bifidobacterium_breve	0.093	0.037	1.19.E-02	5.36.E-01
Siphoviridae	L7_Bifidobacterium_breve	0.002	0.033	9.61.E-01	9.98.E-01
Autographiviridae	L7_Bacteroides_caccae	0.010	0.032	7.61.E-01	9.91.E-01
crAss_like_phage	L7_Bacteroides_caccae	0.024	0.035	4.87.E-01	9.54.E-01
Herelleviridae	L7_Bacteroides_caccae	0.026	0.033	4.39.E-01	9.30.E-01
Microviridae	L7_Bacteroides_caccae	-0.007	0.033	8.33.E-01	9.94.E-01
Myoviridae	L7_Bacteroides_caccae	-0.016	0.037	6.56.E-01	9.79.E-01
Phycodnaviridae	L7_Bacteroides_caccae	0.029	0.033	3.67.E-01	9.15.E-01
Podoviridae	L7_Bacteroides_caccae	0.008	0.038	8.38.E-01	9.94.E-01
Siphoviridae	L7_Bacteroides_caccae	0.054	0.034	1.11.E-01	7.82.E-01

Autographiviridae	L7_Faecalibacterium_prausnitzii	-0.004	0.034	9.05.E-01	9.97.E-01
crAss_like_phage	L7_Faecalibacterium_prausnitzii	-0.011	0.037	7.73.E-01	9.92.E-01
Herelleviridae	L7_Faecalibacterium_prausnitzii	-0.054	0.035	1.24.E-01	7.94.E-01
Microviridae	L7_Faecalibacterium_prausnitzii	0.077	0.035	2.94.E-02	6.66.E-01
Myoviridae	L7_Faecalibacterium_prausnitzii	0.121	0.039	2.00.E-03	3.37.E-01
Phycodnaviridae	L7_Faecalibacterium_prausnitzii	0.016	0.035	6.35.E-01	9.76.E-01
Podoviridae	L7_Faecalibacterium_prausnitzii	0.100	0.040	1.40.E-02	5.51.E-01
Siphoviridae	L7_Faecalibacterium_prausnitzii	-0.010	0.036	7.89.E-01	9.92.E-01
Autographiviridae	L7_Johnsonella_ignava	-0.042	0.035	2.41.E-01	8.85.E-01
crAss_like_phage	L7_Johnsonella_ignava	0.058	0.039	1.41.E-01	8.10.E-01
Herelleviridae	L7_Johnsonella_ignava	-0.054	0.037	1.38.E-01	8.10.E-01
Microviridae	L7_Johnsonella_ignava	0.037	0.037	3.19.E-01	9.15.E-01
Myoviridae	L7_Johnsonella_ignava	0.026	0.041	5.20.E-01	9.57.E-01
Phycodnaviridae	L7_Johnsonella_ignava	0.022	0.036	5.41.E-01	9.60.E-01
Podoviridae	L7_Johnsonella_ignava	0.086	0.042	4.37.E-02	7.22.E-01
Siphoviridae	L7_Johnsonella_ignava	0.014	0.038	7.04.E-01	9.81.E-01
Autographiviridae	L7_Prevotella_sp.	-0.033	0.033	3.16.E-01	9.15.E-01
crAss_like_phage	L7_Prevotella_sp.	-0.011	0.037	7.55.E-01	9.89.E-01
Herelleviridae	L7_Prevotella_sp.	0.006	0.034	8.53.E-01	9.95.E-01
Microviridae	L7_Prevotella_sp.	-0.032	0.034	3.57.E-01	9.15.E-01
Myoviridae	L7_Prevotella_sp.	-0.012	0.038	7.57.E-01	9.90.E-01
Phycodnaviridae	L7_Prevotella_sp.	0.029	0.034	3.97.E-01	9.15.E-01
Podoviridae	L7_Prevotella_sp.	0.005	0.040	9.02.E-01	9.97.E-01
Siphoviridae	L7_Prevotella_sp.	0.019	0.035	5.83.E-01	9.64.E-01
Autographiviridae	L7_Butyricicoccus_pullicaecorum	0.017	0.043	6.93.E-01	9.81.E-01
crAss_like_phage	L7_Butyricicoccus_pullicaecorum	-0.063	0.047	1.82.E-01	8.60.E-01
Herelleviridae	L7_Butyricicoccus_pullicaecorum	-0.014	0.044	7.55.E-01	9.89.E-01
Microviridae	L7_Butyricicoccus_pullicaecorum	0.026	0.044	5.63.E-01	9.61.E-01
Myoviridae	L7_Butyricicoccus_pullicaecorum	0.019	0.049	7.04.E-01	9.81.E-01
Phycodnaviridae	L7_Butyricicoccus_pullicaecorum	-0.045	0.044	3.00.E-01	9.15.E-01
Podoviridae	L7_Butyricicoccus_pullicaecorum	-0.058	0.051	2.53.E-01	8.95.E-01
Siphoviridae	L7_Butyricicoccus_pullicaecorum	0.040	0.045	3.84.E-01	9.15.E-01
Autographiviridae	L7_Klebsiella_sp.	-0.020	0.029	5.07.E-01	9.57.E-01
crAss_like_phage	L7_Klebsiella_sp.	-0.041	0.032	2.13.E-01	8.78.E-01
Herelleviridae	L7_Klebsiella_sp.	-0.044	0.031	1.53.E-01	8.24.E-01
Microviridae	L7_Klebsiella_sp.	-0.011	0.031	7.19.E-01	9.86.E-01
Myoviridae	L7_Klebsiella_sp.	-0.063	0.034	6.60.E-02	7.24.E-01
Phycodnaviridae	L7_Klebsiella_sp.	-0.023	0.030	4.42.E-01	9.32.E-01
Podoviridae	L7_Klebsiella_sp.	-0.014	0.035	6.85.E-01	9.80.E-01
Siphoviridae	L7_Klebsiella_sp.	-0.019	0.031	5.47.E-01	9.60.E-01
Autographiviridae	L7_Klebsiella_quasipneumoniae	0.015	0.026	5.57.E-01	9.61.E-01
crAss_like_phage	L7_Klebsiella_quasipneumoniae	0.052	0.029	7.13.E-02	7.24.E-01
Herelleviridae	L7_Klebsiella_quasipneumoniae	-0.009	0.027	7.36.E-01	9.88.E-01
Microviridae	L7_Klebsiella_quasipneumoniae	-0.004	0.027	8.92.E-01	9.96.E-01
Myoviridae	L7_Klebsiella_quasipneumoniae	-0.046	0.030	1.30.E-01	8.00.E-01
Phycodnaviridae	L7_Klebsiella_quasipneumoniae	0.013	0.027	6.30.E-01	9.76.E-01
Podoviridae	L7_Klebsiella_quasipneumoniae	0.006	0.031	8.58.E-01	9.96.E-01
Siphoviridae	L7_Klebsiella_quasipneumoniae	-0.022	0.028	4.22.E-01	9.22.E-01
Autographiviridae	L7_Eubacterium_sp.	0.060	0.035	8.92.E-02	7.57.E-01
crAss_like_phage	L7_Eubacterium_sp.	-0.032	0.039	4.18.E-01	9.19.E-01
Herelleviridae	L7_Eubacterium_sp.	0.041	0.037	2.68.E-01	9.01.E-01
Microviridae	L7_Eubacterium_sp.	-0.022	0.037	5.47.E-01	9.60.E-01
Myoviridae	L7_Eubacterium_sp.	-0.016	0.041	7.03.E-01	9.81.E-01
Phycodnaviridae	L7_Eubacterium_sp.	0.004	0.036	9.22.E-01	9.98.E-01
Podoviridae	L7_Eubacterium_sp.	-0.157	0.042	1.84.E-04	1.92.E-01
Siphoviridae	L7_Eubacterium_sp.	0.006	0.038	8.79.E-01	9.96.E-01
Autographiviridae	L7_Ruminiclostridium_sp.	0.033	0.031	2.97.E-01	9.15.E-01
crAss_like_phage	L7_Ruminiclostridium_sp.	0.031	0.035	3.67.E-01	9.15.E-01
Herelleviridae	L7_Ruminiclostridium_sp.	-0.036	0.032	2.73.E-01	9.04.E-01
Microviridae	L7_Ruminiclostridium_sp.	-0.029	0.033	3.70.E-01	9.15.E-01
Myoviridae	L7_Ruminiclostridium_sp.	0.059	0.036	1.03.E-01	7.82.E-01
Phycodnaviridae	L7_Ruminiclostridium_sp.	0.034	0.032	2.86.E-01	9.15.E-01
Podoviridae	L7_Ruminiclostridium_sp.	-0.017	0.037	6.56.E-01	9.79.E-01
Siphoviridae	L7_Ruminiclostridium_sp.	-0.006	0.033	8.58.E-01	9.96.E-01
Autographiviridae	L7_Bacteroides_finegoldii	-0.002	0.033	9.61.E-01	9.98.E-01

crAss_like_phage	L7_Bacteroides_finegoldii	-0.019	0.037	6.03.E-01	9.72.E-01
Herelleviridae	L7_Bacteroides_finegoldii	0.062	0.034	6.94.E-02	7.24.E-01
Microviridae	L7_Bacteroides_finegoldii	-0.003	0.035	9.25.E-01	9.98.E-01
Myoviridae	L7_Bacteroides_finegoldii	0.027	0.038	4.91.E-01	9.55.E-01
Phycodnaviridae	L7_Bacteroides_finegoldii	0.055	0.034	1.09.E-01	7.82.E-01
Podoviridae	L7_Bacteroides_finegoldii	0.084	0.040	3.39.E-02	6.82.E-01
Siphoviridae	L7_Bacteroides_finegoldii	0.019	0.035	5.94.E-01	9.69.E-01
Autographiviridae	L7_Blautia_wexlerae	0.050	0.036	1.68.E-01	8.44.E-01
crAss_like_phage	L7_Blautia_wexlerae	0.001	0.040	9.76.E-01	9.99.E-01
Herelleviridae	L7_Blautia_wexlerae	-0.042	0.038	2.66.E-01	9.01.E-01
Microviridae	L7_Blautia_wexlerae	-0.071	0.038	6.10.E-02	7.24.E-01
Myoviridae	L7_Blautia_wexlerae	-0.001	0.042	9.89.E-01	9.99.E-01
Phycodnaviridae	L7_Blautia_wexlerae	0.023	0.037	5.38.E-01	9.60.E-01
Podoviridae	L7_Blautia_wexlerae	-0.040	0.043	3.55.E-01	9.15.E-01
Siphoviridae	L7_Blautia_wexlerae	-0.083	0.039	3.24.E-02	6.75.E-01
Autographiviridae	L7_Providencia_alcalifaciens	0.069	0.036	5.71.E-02	7.24.E-01
crAss_like_phage	L7_Providencia_alcalifaciens	0.028	0.040	4.83.E-01	9.54.E-01
Herelleviridae	L7_Providencia_alcalifaciens	-0.012	0.038	7.45.E-01	9.89.E-01
Microviridae	L7_Providencia_alcalifaciens	0.052	0.038	1.64.E-01	8.40.E-01
Myoviridae	L7_Providencia_alcalifaciens	0.017	0.042	6.83.E-01	9.80.E-01
Phycodnaviridae	L7_Providencia_alcalifaciens	0.026	0.037	4.93.E-01	9.55.E-01
Podoviridae	L7_Providencia_alcalifaciens	0.121	0.043	5.26.E-03	4.28.E-01
Siphoviridae	L7_Providencia_alcalifaciens	-0.055	0.039	1.55.E-01	8.26.E-01
Autographiviridae	L7_Megasphaera_elsdenii	0.000	0.040	9.93.E-01	9.99.E-01
crAss_like_phage	L7_Megasphaera_elsdenii	0.017	0.044	7.04.E-01	9.81.E-01
Herelleviridae	L7_Megasphaera_elsdenii	-0.042	0.041	3.05.E-01	9.15.E-01
Microviridae	L7_Megasphaera_elsdenii	-0.045	0.041	2.81.E-01	9.12.E-01
Myoviridae	L7_Megasphaera_elsdenii	-0.020	0.046	6.67.E-01	9.80.E-01
Phycodnaviridae	L7_Megasphaera_elsdenii	-0.016	0.041	6.97.E-01	9.81.E-01
Podoviridae	L7_Megasphaera_elsdenii	-0.076	0.047	1.11.E-01	7.82.E-01
Siphoviridae	L7_Megasphaera_elsdenii	0.020	0.042	6.41.E-01	9.79.E-01
Autographiviridae	L7_Megasphaera_massiliensis	0.070	0.042	9.26.E-02	7.60.E-01
crAss_like_phage	L7_Megasphaera_massiliensis	0.016	0.046	7.36.E-01	9.88.E-01
Herelleviridae	L7_Megasphaera_massiliensis	-0.017	0.043	6.91.E-01	9.80.E-01
Microviridae	L7_Megasphaera_massiliensis	0.025	0.043	5.59.E-01	9.61.E-01
Myoviridae	L7_Megasphaera_massiliensis	-0.042	0.048	3.91.E-01	9.15.E-01
Phycodnaviridae	L7_Megasphaera_massiliensis	-0.055	0.043	2.01.E-01	8.74.E-01
Podoviridae	L7_Megasphaera_massiliensis	-0.041	0.050	4.11.E-01	9.18.E-01
Siphoviridae	L7_Megasphaera_massiliensis	0.004	0.045	9.36.E-01	9.98.E-01
Autographiviridae	L7_Alistipes_senegalensis	0.033	0.029	2.55.E-01	8.95.E-01
crAss_like_phage	L7_Alistipes_senegalensis	0.042	0.032	1.80.E-01	8.60.E-01
Herelleviridae	L7_Alistipes_senegalensis	0.025	0.030	3.94.E-01	9.15.E-01
Microviridae	L7_Alistipes_senegalensis	-0.005	0.030	8.78.E-01	9.96.E-01
Myoviridae	L7_Alistipes_senegalensis	-0.046	0.033	1.67.E-01	8.44.E-01
Phycodnaviridae	L7_Alistipes_senegalensis	-0.012	0.029	6.78.E-01	9.80.E-01
Podoviridae	L7_Alistipes_senegalensis	0.042	0.034	2.19.E-01	8.79.E-01
Siphoviridae	L7_Alistipes_senegalensis	0.000	0.031	9.93.E-01	9.99.E-01
Autographiviridae	L7_Acetivibrio_sp.	-0.032	0.039	4.05.E-01	9.17.E-01
crAss_like_phage	L7_Acetivibrio_sp.	0.011	0.043	7.90.E-01	9.92.E-01
Herelleviridae	L7_Acetivibrio_sp.	-0.114	0.040	4.43.E-03	4.24.E-01
Microviridae	L7_Acetivibrio_sp.	0.053	0.040	1.92.E-01	8.61.E-01
Myoviridae	L7_Acetivibrio_sp.	0.006	0.045	8.94.E-01	9.97.E-01
Phycodnaviridae	L7_Acetivibrio_sp.	-0.035	0.040	3.80.E-01	9.15.E-01
Podoviridae	L7_Acetivibrio_sp.	0.044	0.047	3.48.E-01	9.15.E-01
Siphoviridae	L7_Acetivibrio_sp.	-0.025	0.042	5.53.E-01	9.60.E-01
Autographiviridae	L7_Olsenella_sp.	-0.004	0.038	9.20.E-01	9.98.E-01
crAss_like_phage	L7_Olsenella_sp.	-0.050	0.041	2.26.E-01	8.82.E-01
Herelleviridae	L7_Olsenella_sp.	-0.035	0.039	3.72.E-01	9.15.E-01
Microviridae	L7_Olsenella_sp.	-0.051	0.039	1.92.E-01	8.61.E-01
Myoviridae	L7_Olsenella_sp.	0.017	0.044	7.03.E-01	9.81.E-01
Phycodnaviridae	L7_Olsenella_sp.	-0.039	0.038	3.09.E-01	9.15.E-01
Podoviridae	L7_Olsenella_sp.	-0.066	0.045	1.43.E-01	8.12.E-01
Siphoviridae	L7_Olsenella_sp.	-0.042	0.040	2.99.E-01	9.15.E-01
Autographiviridae	L7_Clostridium_butyricum	0.090	0.045	4.46.E-02	7.22.E-01
crAss_like_phage	L7_Clostridium_butyricum	0.038	0.050	4.39.E-01	9.30.E-01

Herelleviridae	L7_Clostridium_butyricum	-0.025	0.047	5.97.E-01	9.69.E-01
Microviridae	L7_Clostridium_butyricum	-0.022	0.047	6.40.E-01	9.79.E-01
Myoviridae	L7_Clostridium_butyricum	0.065	0.052	2.13.E-01	8.78.E-01
Phycodnaviridae	L7_Clostridium_butyricum	0.058	0.046	2.12.E-01	8.78.E-01
Podoviridae	L7_Clostridium_butyricum	-0.080	0.054	1.38.E-01	8.10.E-01
Siphoviridae	L7_Clostridium_butyricum	-0.040	0.048	4.04.E-01	9.17.E-01
Autographiviridae	L7_Enorma_timonensis	0.003	0.036	9.29.E-01	9.98.E-01
crAss_like_phage	L7_Enorma_timonensis	-0.025	0.040	5.36.E-01	9.60.E-01
Herelleviridae	L7_Enorma_timonensis	-0.036	0.037	3.36.E-01	9.15.E-01
Microviridae	L7_Enorma_timonensis	-0.059	0.037	1.13.E-01	7.84.E-01
Myoviridae	L7_Enorma_timonensis	-0.004	0.042	9.19.E-01	9.98.E-01
Phycodnaviridae	L7_Enorma_timonensis	-0.043	0.037	2.42.E-01	8.86.E-01
Podoviridae	L7_Enorma_timonensis	0.031	0.043	4.74.E-01	9.52.E-01
Siphoviridae	L7_Enorma_timonensis	-0.059	0.038	1.24.E-01	7.94.E-01
Autographiviridae	L7_Rothia_dentocariosa	0.058	0.033	8.61.E-02	7.52.E-01
crAss_like_phage	L7_Rothia_dentocariosa	-0.006	0.037	8.64.E-01	9.96.E-01
Herelleviridae	L7_Rothia_dentocariosa	-0.007	0.035	8.41.E-01	9.95.E-01
Microviridae	L7_Rothia_dentocariosa	-0.090	0.035	1.01.E-02	5.11.E-01
Myoviridae	L7_Rothia_dentocariosa	0.042	0.039	2.77.E-01	9.05.E-01
Phycodnaviridae	L7_Rothia_dentocariosa	0.042	0.034	2.27.E-01	8.82.E-01
Podoviridae	L7_Rothia_dentocariosa	0.010	0.040	8.02.E-01	9.92.E-01
Siphoviridae	L7_Rothia_dentocariosa	0.005	0.036	8.88.E-01	9.96.E-01
Autographiviridae	L7_Anaerotignum_lactatifermentans	-0.006	0.041	8.83.E-01	9.96.E-01
crAss_like_phage	L7_Anaerotignum_lactatifermentans	-0.044	0.045	3.35.E-01	9.15.E-01
Herelleviridae	L7_Anaerotignum_lactatifermentans	-0.029	0.043	4.94.E-01	9.55.E-01
Microviridae	L7_Anaerotignum_lactatifermentans	0.009	0.043	8.38.E-01	9.94.E-01
Myoviridae	L7_Anaerotignum_lactatifermentans	0.084	0.048	7.96.E-02	7.43.E-01
Phycodnaviridae	L7_Anaerotignum_lactatifermentans	0.015	0.042	7.14.E-01	9.86.E-01
Podoviridae	L7_Anaerotignum_lactatifermentans	-0.048	0.049	3.29.E-01	9.15.E-01
Siphoviridae	L7_Anaerotignum_lactatifermentans	-0.004	0.044	9.30.E-01	9.98.E-01
Autographiviridae	L7_Alistipes_obesi	-0.035	0.031	2.55.E-01	8.95.E-01
crAss_like_phage	L7_Alistipes_obesi	0.003	0.034	9.27.E-01	9.98.E-01
Herelleviridae	L7_Alistipes_obesi	0.053	0.032	1.00.E-01	7.79.E-01
Microviridae	L7_Alistipes_obesi	0.000	0.032	9.88.E-01	9.99.E-01
Myoviridae	L7_Alistipes_obesi	0.050	0.036	1.63.E-01	8.38.E-01
Phycodnaviridae	L7_Alistipes_obesi	0.004	0.032	9.01.E-01	9.97.E-01
Podoviridae	L7_Alistipes_obesi	0.031	0.037	4.08.E-01	9.17.E-01
Siphoviridae	L7_Alistipes_obesi	0.046	0.033	1.63.E-01	8.38.E-01
Autographiviridae	L7_Enterobacter_hormaechei	-0.031	0.028	2.68.E-01	9.01.E-01
crAss_like_phage	L7_Enterobacter_hormaechei	0.048	0.031	1.17.E-01	7.86.E-01
Herelleviridae	L7_Enterobacter_hormaechei	0.026	0.029	3.76.E-01	9.15.E-01
Microviridae	L7_Enterobacter_hormaechei	0.052	0.029	7.29.E-02	7.26.E-01
Myoviridae	L7_Enterobacter_hormaechei	0.027	0.032	4.08.E-01	9.17.E-01
Phycodnaviridae	L7_Enterobacter_hormaechei	-0.020	0.028	4.85.E-01	9.54.E-01
Podoviridae	L7_Enterobacter_hormaechei	0.023	0.033	4.95.E-01	9.55.E-01
Siphoviridae	L7_Enterobacter_hormaechei	0.041	0.030	1.66.E-01	8.44.E-01
Autographiviridae	L7_Clostridium_cf.	-0.003	0.033	9.26.E-01	9.98.E-01
crAss_like_phage	L7_Clostridium_cf.	-0.041	0.036	2.58.E-01	8.95.E-01
Herelleviridae	L7_Clostridium_cf.	-0.065	0.034	5.90.E-02	7.24.E-01
Microviridae	L7_Clostridium_cf.	0.005	0.034	8.82.E-01	9.96.E-01
Myoviridae	L7_Clostridium_cf.	0.007	0.038	8.58.E-01	9.96.E-01
Phycodnaviridae	L7_Clostridium_cf.	-0.028	0.034	4.10.E-01	9.17.E-01
Podoviridae	L7_Clostridium_cf.	-0.039	0.040	3.24.E-01	9.15.E-01
Siphoviridae	L7_Clostridium_cf.	-0.016	0.035	6.58.E-01	9.79.E-01
Autographiviridae	L7_Sutterella_parvirubra	-0.017	0.041	6.73.E-01	9.80.E-01
crAss_like_phage	L7_Sutterella_parvirubra	-0.011	0.046	8.14.E-01	9.93.E-01
Herelleviridae	L7_Sutterella_parvirubra	-0.037	0.043	3.93.E-01	9.15.E-01
Microviridae	L7_Sutterella_parvirubra	-0.002	0.043	9.61.E-01	9.98.E-01
Myoviridae	L7_Sutterella_parvirubra	-0.048	0.048	3.16.E-01	9.15.E-01
Phycodnaviridae	L7_Sutterella_parvirubra	-0.026	0.043	5.44.E-01	9.60.E-01
Podoviridae	L7_Sutterella_parvirubra	0.033	0.050	5.07.E-01	9.57.E-01
Siphoviridae	L7_Sutterella_parvirubra	-0.079	0.044	7.26.E-02	7.26.E-01
Autographiviridae	L7_Ruminococcaceae_bacterium	-0.035	0.029	2.27.E-01	8.82.E-01
crAss_like_phage	L7_Ruminococcaceae_bacterium	0.017	0.032	5.98.E-01	9.69.E-01
Herelleviridae	L7_Ruminococcaceae_bacterium	-0.013	0.030	6.64.E-01	9.79.E-01

Microviridae	L7_Ruminococcaceae_bacterium	0.004	0.030	8.82.E-01	9.96.E-01
Myoviridae	L7_Ruminococcaceae_bacterium	0.044	0.034	1.96.E-01	8.66.E-01
Phycodnaviridae	L7_Ruminococcaceae_bacterium	-0.055	0.030	6.44.E-02	7.24.E-01
Podoviridae	L7_Ruminococcaceae_bacterium	0.013	0.035	7.03.E-01	9.81.E-01
Siphoviridae	L7_Ruminococcaceae_bacterium	-0.033	0.031	2.94.E-01	9.15.E-01
Autographiviridae	L7_Parabacteroides_johnsonii	0.008	0.037	8.37.E-01	9.94.E-01
crAss_like_phage	L7_Parabacteroides_johnsonii	-0.032	0.041	4.32.E-01	9.25.E-01
Herelleviridae	L7_Parabacteroides_johnsonii	0.056	0.039	1.47.E-01	8.17.E-01
Microviridae	L7_Parabacteroides_johnsonii	0.016	0.039	6.78.E-01	9.80.E-01
Myoviridae	L7_Parabacteroides_johnsonii	0.015	0.043	7.31.E-01	9.88.E-01
Phycodnaviridae	L7_Parabacteroides_johnsonii	-0.034	0.038	3.75.E-01	9.15.E-01
Podoviridae	L7_Parabacteroides_johnsonii	0.014	0.045	7.63.E-01	9.91.E-01
Siphoviridae	L7_Parabacteroides_johnsonii	-0.025	0.040	5.34.E-01	9.59.E-01
Autographiviridae	L7_Terrisporobacter_glycolicus	0.027	0.029	3.40.E-01	9.15.E-01
crAss_like_phage	L7_Terrisporobacter_glycolicus	-0.006	0.032	8.44.E-01	9.95.E-01
Herelleviridae	L7_Terrisporobacter_glycolicus	0.040	0.030	1.82.E-01	8.60.E-01
Microviridae	L7_Terrisporobacter_glycolicus	-0.095	0.029	1.31.E-03	2.90.E-01
Myoviridae	L7_Terrisporobacter_glycolicus	-0.019	0.033	5.76.E-01	9.63.E-01
Phycodnaviridae	L7_Terrisporobacter_glycolicus	0.034	0.029	2.47.E-01	8.92.E-01
Podoviridae	L7_Terrisporobacter_glycolicus	0.046	0.034	1.79.E-01	8.60.E-01
Siphoviridae	L7_Terrisporobacter_glycolicus	0.007	0.031	8.08.E-01	9.93.E-01
Autographiviridae	L7_Staphylococcus_aureus	0.015	0.038	6.99.E-01	9.81.E-01
crAss_like_phage	L7_Staphylococcus_aureus	-0.035	0.042	4.02.E-01	9.15.E-01
Herelleviridae	L7_Staphylococcus_aureus	-0.048	0.039	2.25.E-01	8.82.E-01
Microviridae	L7_Staphylococcus_aureus	-0.026	0.040	5.14.E-01	9.57.E-01
Myoviridae	L7_Staphylococcus_aureus	-0.079	0.044	7.35.E-02	7.26.E-01
Phycodnaviridae	L7_Staphylococcus_aureus	-0.064	0.039	9.82.E-02	7.78.E-01
Podoviridae	L7_Staphylococcus_aureus	-0.019	0.046	6.81.E-01	9.80.E-01
Siphoviridae	L7_Staphylococcus_aureus	-0.067	0.040	9.96.E-02	7.79.E-01
Autographiviridae	L7_Butyrivibrio_crossotus	-0.010	0.033	7.64.E-01	9.91.E-01
crAss_like_phage	L7_Butyrivibrio_crossotus	-0.027	0.036	4.59.E-01	9.43.E-01
Herelleviridae	L7_Butyrivibrio_crossotus	-0.024	0.034	4.77.E-01	9.52.E-01
Microviridae	L7_Butyrivibrio_crossotus	-0.073	0.034	3.21.E-02	6.75.E-01
Myoviridae	L7_Butyrivibrio_crossotus	-0.064	0.038	9.11.E-02	7.59.E-01
Phycodnaviridae	L7_Butyrivibrio_crossotus	-0.043	0.033	1.97.E-01	8.66.E-01
Podoviridae	L7_Butyrivibrio_crossotus	-0.011	0.039	7.76.E-01	9.92.E-01
Siphoviridae	L7_Butyrivibrio_crossotus	-0.053	0.035	1.31.E-01	8.00.E-01
Autographiviridae	L7_Pediococcus_acidilactici	0.003	0.040	9.34.E-01	9.98.E-01
crAss_like_phage	L7_Pediococcus_acidilactici	-0.005	0.044	9.11.E-01	9.98.E-01
Herelleviridae	L7_Pediococcus_acidilactici	0.012	0.042	7.78.E-01	9.92.E-01
Microviridae	L7_Pediococcus_acidilactici	0.041	0.042	3.23.E-01	9.15.E-01
Myoviridae	L7_Pediococcus_acidilactici	-0.035	0.047	4.52.E-01	9.38.E-01
Phycodnaviridae	L7_Pediococcus_acidilactici	0.055	0.041	1.86.E-01	8.61.E-01
Podoviridae	L7_Pediococcus_acidilactici	0.008	0.048	8.64.E-01	9.96.E-01
Siphoviridae	L7_Pediococcus_acidilactici	0.077	0.043	7.13.E-02	7.24.E-01
Autographiviridae	L7_Lactobacillus_johnsonii	-0.082	0.034	1.66.E-02	5.86.E-01
crAss_like_phage	L7_Lactobacillus_johnsonii	-0.026	0.038	4.95.E-01	9.55.E-01
Herelleviridae	L7_Lactobacillus_johnsonii	-0.020	0.036	5.70.E-01	9.61.E-01
Microviridae	L7_Lactobacillus_johnsonii	-0.020	0.036	5.82.E-01	9.64.E-01
Myoviridae	L7_Lactobacillus_johnsonii	-0.063	0.040	1.17.E-01	7.86.E-01
Phycodnaviridae	L7_Lactobacillus_johnsonii	0.012	0.035	7.32.E-01	9.88.E-01
Podoviridae	L7_Lactobacillus_johnsonii	-0.014	0.041	7.44.E-01	9.89.E-01
Siphoviridae	L7_Lactobacillus_johnsonii	-0.046	0.037	2.13.E-01	8.78.E-01
Autographiviridae	L7_Clostridium_leptum	-0.001	0.036	9.86.E-01	9.99.E-01
crAss_like_phage	L7_Clostridium_leptum	0.069	0.040	8.20.E-02	7.48.E-01
Herelleviridae	L7_Clostridium_leptum	-0.077	0.037	3.93.E-02	7.03.E-01
Microviridae	L7_Clostridium_leptum	-0.049	0.038	1.95.E-01	8.66.E-01
Myoviridae	L7_Clostridium_leptum	-0.004	0.042	9.30.E-01	9.98.E-01
Phycodnaviridae	L7_Clostridium_leptum	-0.008	0.037	8.38.E-01	9.94.E-01
Podoviridae	L7_Clostridium_leptum	-0.002	0.043	9.62.E-01	9.98.E-01
Siphoviridae	L7_Clostridium_leptum	-0.006	0.039	8.71.E-01	9.96.E-01
Autographiviridae	L7_Streptococcus_equinus	-0.016	0.040	6.87.E-01	9.80.E-01
crAss_like_phage	L7_Streptococcus_equinus	0.007	0.044	8.68.E-01	9.96.E-01
Herelleviridae	L7_Streptococcus_equinus	0.027	0.042	5.13.E-01	9.57.E-01
Microviridae	L7_Streptococcus_equinus	0.019	0.042	6.56.E-01	9.79.E-01

Myoviridae	L7_Streptococcus_equinus	-0.107	0.046	2.08.E-02	6.04.E-01
Phycodnaviridae	L7_Streptococcus_equinus	-0.043	0.041	3.01.E-01	9.15.E-01
Podoviridae	L7_Streptococcus_equinus	-0.013	0.048	7.83.E-01	9.92.E-01
Siphoviridae	L7_Streptococcus_equinus	-0.003	0.043	9.49.E-01	9.98.E-01
Autographiviridae	L7_Anaerovorax_sp.	0.036	0.038	3.50.E-01	9.15.E-01
crAss_like_phage	L7_Anaerovorax_sp.	-0.041	0.042	3.30.E-01	9.15.E-01
Herelleviridae	L7_Anaerovorax_sp.	0.020	0.040	6.23.E-01	9.76.E-01
Microviridae	L7_Anaerovorax_sp.	-0.020	0.040	6.12.E-01	9.75.E-01
Myoviridae	L7_Anaerovorax_sp.	-0.019	0.044	6.62.E-01	9.79.E-01
Phycodnaviridae	L7_Anaerovorax_sp.	0.045	0.039	2.54.E-01	8.95.E-01
Podoviridae	L7_Anaerovorax_sp.	-0.022	0.046	6.24.E-01	9.76.E-01
Siphoviridae	L7_Anaerovorax_sp.	0.053	0.041	1.94.E-01	8.65.E-01
Autographiviridae	L7_Prevotella_multiformis	-0.031	0.039	4.35.E-01	9.26.E-01
crAss_like_phage	L7_Prevotella_multiformis	-0.003	0.043	9.53.E-01	9.98.E-01
Herelleviridae	L7_Prevotella_multiformis	0.037	0.041	3.59.E-01	9.15.E-01
Microviridae	L7_Prevotella_multiformis	0.012	0.041	7.72.E-01	9.92.E-01
Myoviridae	L7_Prevotella_multiformis	-0.001	0.046	9.87.E-01	9.99.E-01
Phycodnaviridae	L7_Prevotella_multiformis	0.036	0.040	3.76.E-01	9.15.E-01
Podoviridae	L7_Prevotella_multiformis	0.003	0.047	9.53.E-01	9.98.E-01
Siphoviridae	L7_Prevotella_multiformis	-0.012	0.042	7.74.E-01	9.92.E-01
Autographiviridae	L7_Sutterella_wadsworthensis	0.095	0.039	1.45.E-02	5.54.E-01
crAss_like_phage	L7_Sutterella_wadsworthensis	0.056	0.043	1.92.E-01	8.61.E-01
Herelleviridae	L7_Sutterella_wadsworthensis	-0.051	0.041	2.12.E-01	8.78.E-01
Microviridae	L7_Sutterella_wadsworthensis	-0.027	0.041	5.12.E-01	9.57.E-01
Myoviridae	L7_Sutterella_wadsworthensis	-0.035	0.045	4.43.E-01	9.32.E-01
Phycodnaviridae	L7_Sutterella_wadsworthensis	0.006	0.040	8.77.E-01	9.96.E-01
Podoviridae	L7_Sutterella_wadsworthensis	0.045	0.047	3.39.E-01	9.15.E-01
Siphoviridae	L7_Sutterella_wadsworthensis	0.001	0.042	9.72.E-01	9.99.E-01
Autographiviridae	L7_Lactobacillus_fermentum	-0.008	0.030	7.86.E-01	9.92.E-01
crAss_like_phage	L7_Lactobacillus_fermentum	-0.028	0.034	4.09.E-01	9.17.E-01
Herelleviridae	L7_Lactobacillus_fermentum	0.021	0.032	5.03.E-01	9.55.E-01
Microviridae	L7_Lactobacillus_fermentum	-0.030	0.032	3.37.E-01	9.15.E-01
Myoviridae	L7_Lactobacillus_fermentum	0.032	0.035	3.67.E-01	9.15.E-01
Phycodnaviridae	L7_Lactobacillus_fermentum	0.049	0.031	1.17.E-01	7.86.E-01
Podoviridae	L7_Lactobacillus_fermentum	-0.047	0.036	1.95.E-01	8.66.E-01
Siphoviridae	L7_Lactobacillus_fermentum	0.001	0.032	9.86.E-01	9.99.E-01
Autographiviridae	L7_Bifidobacterium_saeculare	-0.074	0.038	4.98.E-02	7.22.E-01
crAss_like_phage	L7_Bifidobacterium_saeculare	-0.064	0.042	1.28.E-01	7.99.E-01
Herelleviridae	L7_Bifidobacterium_saeculare	-0.082	0.039	3.76.E-02	6.99.E-01
Microviridae	L7_Bifidobacterium_saeculare	-0.024	0.039	5.35.E-01	9.59.E-01
Myoviridae	L7_Bifidobacterium_saeculare	-0.095	0.044	3.11.E-02	6.70.E-01
Phycodnaviridae	L7_Bifidobacterium_saeculare	0.023	0.039	5.52.E-01	9.60.E-01
Podoviridae	L7_Bifidobacterium_saeculare	-0.029	0.045	5.24.E-01	9.57.E-01
Siphoviridae	L7_Bifidobacterium_saeculare	-0.025	0.040	5.40.E-01	9.60.E-01
Autographiviridae	L7_Cryptobacterium_sp.	0.023	0.041	5.79.E-01	9.64.E-01
crAss_like_phage	L7_Cryptobacterium_sp.	-0.001	0.045	9.83.E-01	9.99.E-01
Herelleviridae	L7_Cryptobacterium_sp.	0.048	0.042	2.57.E-01	8.95.E-01
Microviridae	L7_Cryptobacterium_sp.	-0.048	0.042	2.62.E-01	9.00.E-01
Myoviridae	L7_Cryptobacterium_sp.	0.060	0.047	2.02.E-01	8.75.E-01
Phycodnaviridae	L7_Cryptobacterium_sp.	-0.014	0.042	7.41.E-01	9.89.E-01
Podoviridae	L7_Cryptobacterium_sp.	-0.071	0.049	1.44.E-01	8.12.E-01
Siphoviridae	L7_Cryptobacterium_sp.	-0.063	0.043	1.47.E-01	8.16.E-01
Autographiviridae	L7_Collinsella_massiliensis	-0.006	0.036	8.69.E-01	9.96.E-01
crAss_like_phage	L7_Collinsella_massiliensis	-0.024	0.040	5.57.E-01	9.61.E-01
Herelleviridae	L7_Collinsella_massiliensis	-0.010	0.038	7.95.E-01	9.92.E-01
Microviridae	L7_Collinsella_massiliensis	-0.079	0.038	3.62.E-02	6.90.E-01
Myoviridae	L7_Collinsella_massiliensis	0.010	0.042	8.22.E-01	9.93.E-01
Phycodnaviridae	L7_Collinsella_massiliensis	-0.038	0.037	3.14.E-01	9.15.E-01
Podoviridae	L7_Collinsella_massiliensis	0.019	0.044	6.56.E-01	9.79.E-01
Siphoviridae	L7_Collinsella_massiliensis	-0.040	0.039	3.02.E-01	9.15.E-01
Autographiviridae	L7_Parabacteroides_merdae	-0.004	0.034	9.06.E-01	9.97.E-01
crAss_like_phage	L7_Parabacteroides_merdae	-0.032	0.038	3.91.E-01	9.15.E-01
Herelleviridae	L7_Parabacteroides_merdae	0.017	0.036	6.29.E-01	9.76.E-01
Microviridae	L7_Parabacteroides_merdae	0.056	0.036	1.19.E-01	7.89.E-01
Myoviridae	L7_Parabacteroides_merdae	-0.003	0.040	9.44.E-01	9.98.E-01

Phycodnaviridae	L7_Parabacteroides_merdae	0.087	0.035	1.33.E-02	5.51.E-01
Podoviridae	L7_Parabacteroides_merdae	0.048	0.041	2.41.E-01	8.85.E-01
Siphoviridae	L7_Parabacteroides_merdae	0.037	0.037	3.18.E-01	9.15.E-01
Autographiviridae	L7_Actinobacillus_pleuropneumoniae	0.000	0.011	9.97.E-01	9.99.E-01
crAss_like_phage	L7_Actinobacillus_pleuropneumoniae	-0.016	0.013	2.06.E-01	8.76.E-01
Herelleviridae	L7_Actinobacillus_pleuropneumoniae	-0.015	0.012	1.90.E-01	8.61.E-01
Microviridae	L7_Actinobacillus_pleuropneumoniae	0.030	0.012	1.14.E-02	5.36.E-01
Myoviridae	L7_Actinobacillus_pleuropneumoniae	0.006	0.013	6.46.E-01	9.79.E-01
Phycodnaviridae	L7_Actinobacillus_pleuropneumoniae	0.002	0.012	8.70.E-01	9.96.E-01
Podoviridae	L7_Actinobacillus_pleuropneumoniae	-0.009	0.014	5.03.E-01	9.55.E-01
Siphoviridae	L7_Actinobacillus_pleuropneumoniae	-0.029	0.012	1.66.E-02	5.86.E-01
Autographiviridae	L7_Faecalitalea_cylindroides	-0.018	0.034	6.03.E-01	9.72.E-01
crAss_like_phage	L7_Faecalitalea_cylindroides	0.017	0.038	6.44.E-01	9.79.E-01
Herelleviridae	L7_Faecalitalea_cylindroides	0.011	0.035	7.47.E-01	9.89.E-01
Microviridae	L7_Faecalitalea_cylindroides	-0.031	0.035	3.82.E-01	9.15.E-01
Myoviridae	L7_Faecalitalea_cylindroides	-0.016	0.039	6.77.E-01	9.80.E-01
Phycodnaviridae	L7_Faecalitalea_cylindroides	-0.001	0.035	9.76.E-01	9.99.E-01
Podoviridae	L7_Faecalitalea_cylindroides	-0.066	0.041	1.04.E-01	7.82.E-01
Siphoviridae	L7_Faecalitalea_cylindroides	-0.051	0.036	1.63.E-01	8.38.E-01
Autographiviridae	L7_Turicibacter_sp.	0.108	0.037	3.58.E-03	4.02.E-01
crAss_like_phage	L7_Turicibacter_sp.	0.065	0.041	1.15.E-01	7.85.E-01
Herelleviridae	L7_Turicibacter_sp.	0.043	0.039	2.70.E-01	9.02.E-01
Microviridae	L7_Turicibacter_sp.	0.012	0.039	7.62.E-01	9.91.E-01
Myoviridae	L7_Turicibacter_sp.	0.015	0.043	7.20.E-01	9.86.E-01
Phycodnaviridae	L7_Turicibacter_sp.	0.009	0.038	8.13.E-01	9.93.E-01
Podoviridae	L7_Turicibacter_sp.	0.035	0.045	4.32.E-01	9.25.E-01
Siphoviridae	L7_Turicibacter_sp.	0.000	0.040	9.96.E-01	9.99.E-01
Autographiviridae	L7_Streptococcus_suis	-0.014	0.040	7.17.E-01	9.86.E-01
crAss_like_phage	L7_Streptococcus_suis	-0.076	0.044	8.49.E-02	7.50.E-01
Herelleviridae	L7_Streptococcus_suis	0.050	0.041	2.31.E-01	8.82.E-01
Microviridae	L7_Streptococcus_suis	-0.039	0.042	3.44.E-01	9.15.E-01
Myoviridae	L7_Streptococcus_suis	-0.034	0.046	4.67.E-01	9.49.E-01
Phycodnaviridae	L7_Streptococcus_suis	0.011	0.041	7.95.E-01	9.92.E-01
Podoviridae	L7_Streptococcus_suis	-0.030	0.048	5.30.E-01	9.57.E-01
Siphoviridae	L7_Streptococcus_suis	-0.040	0.043	3.52.E-01	9.15.E-01
Autographiviridae	L7_Barnesiella_intestinihominis	-0.062	0.036	8.55.E-02	7.51.E-01
crAss_like_phage	L7_Barnesiella_intestinihominis	0.023	0.040	5.65.E-01	9.61.E-01
Herelleviridae	L7_Barnesiella_intestinihominis	0.018	0.038	6.27.E-01	9.76.E-01
Microviridae	L7_Barnesiella_intestinihominis	0.076	0.038	4.46.E-02	7.22.E-01
Myoviridae	L7_Barnesiella_intestinihominis	-0.028	0.042	5.08.E-01	9.57.E-01
Phycodnaviridae	L7_Barnesiella_intestinihominis	-0.001	0.037	9.85.E-01	9.99.E-01
Podoviridae	L7_Barnesiella_intestinihominis	-0.031	0.043	4.78.E-01	9.52.E-01
Siphoviridae	L7_Barnesiella_intestinihominis	0.066	0.039	8.81.E-02	7.54.E-01
Autographiviridae	L7_Anaerotruncus_sp.	-0.063	0.027	2.23.E-02	6.19.E-01
crAss_like_phage	L7_Anaerotruncus_sp.	0.019	0.030	5.34.E-01	9.59.E-01
Herelleviridae	L7_Anaerotruncus_sp.	0.019	0.028	5.07.E-01	9.57.E-01
Microviridae	L7_Anaerotruncus_sp.	0.031	0.029	2.80.E-01	9.11.E-01
Myoviridae	L7_Anaerotruncus_sp.	0.019	0.032	5.50.E-01	9.60.E-01
Phycodnaviridae	L7_Anaerotruncus_sp.	0.041	0.028	1.50.E-01	8.22.E-01
Podoviridae	L7_Anaerotruncus_sp.	0.029	0.033	3.83.E-01	9.15.E-01
Siphoviridae	L7_Anaerotruncus_sp.	0.012	0.029	6.92.E-01	9.81.E-01
Autographiviridae	L7_Coprobacillus_cateniformis	-0.006	0.043	8.80.E-01	9.96.E-01
crAss_like_phage	L7_Coprobacillus_cateniformis	0.070	0.047	1.36.E-01	8.07.E-01
Herelleviridae	L7_Coprobacillus_cateniformis	0.039	0.044	3.74.E-01	9.15.E-01
Microviridae	L7_Coprobacillus_cateniformis	0.044	0.044	3.26.E-01	9.15.E-01
Myoviridae	L7_Coprobacillus_cateniformis	-0.108	0.049	2.87.E-02	6.66.E-01
Phycodnaviridae	L7_Coprobacillus_cateniformis	-0.030	0.044	4.96.E-01	9.55.E-01
Podoviridae	L7_Coprobacillus_cateniformis	0.004	0.051	9.42.E-01	9.98.E-01
Siphoviridae	L7_Coprobacillus_cateniformis	0.045	0.045	3.23.E-01	9.15.E-01
Autographiviridae	L7_Staphylococcus_sp.	-0.009	0.040	8.24.E-01	9.93.E-01
crAss_like_phage	L7_Staphylococcus_sp.	-0.097	0.044	2.70.E-02	6.58.E-01
Herelleviridae	L7_Staphylococcus_sp.	0.035	0.041	3.92.E-01	9.15.E-01
Microviridae	L7_Staphylococcus_sp.	-0.006	0.042	8.82.E-01	9.96.E-01
Myoviridae	L7_Staphylococcus_sp.	-0.005	0.046	9.16.E-01	9.98.E-01
Phycodnaviridae	L7_Staphylococcus_sp.	-0.004	0.041	9.22.E-01	9.98.E-01

Podoviridae	L7_Staphylococcus_sp.	-0.049	0.048	3.05.E-01	9.15.E-01
Siphoviridae	L7_Staphylococcus_sp.	-0.011	0.043	7.90.E-01	9.92.E-01
Autographiviridae	L7_Klebsiella_pneumoniae	0.011	0.024	6.39.E-01	9.78.E-01
crAss_like_phage	L7_Klebsiella_pneumoniae	-0.001	0.027	9.80.E-01	9.99.E-01
Herelleviridae	L7_Klebsiella_pneumoniae	-0.002	0.025	9.47.E-01	9.98.E-01
Microviridae	L7_Klebsiella_pneumoniae	0.009	0.025	7.21.E-01	9.86.E-01
Myoviridae	L7_Klebsiella_pneumoniae	-0.057	0.028	3.98.E-02	7.03.E-01
Phycodnaviridae	L7_Klebsiella_pneumoniae	0.007	0.025	7.83.E-01	9.92.E-01
Podoviridae	L7_Klebsiella_pneumoniae	-0.004	0.029	8.91.E-01	9.96.E-01
Siphoviridae	L7_Klebsiella_pneumoniae	-0.015	0.026	5.70.E-01	9.61.E-01
Autographiviridae	L7_Lactobacillus_crispatus	-0.071	0.038	5.98.E-02	7.24.E-01
crAss_like_phage	L7_Lactobacillus_crispatus	0.009	0.042	8.24.E-01	9.93.E-01
Herelleviridae	L7_Lactobacillus_crispatus	-0.015	0.039	7.03.E-01	9.81.E-01
Microviridae	L7_Lactobacillus_crispatus	-0.033	0.039	4.05.E-01	9.17.E-01
Myoviridae	L7_Lactobacillus_crispatus	0.003	0.044	9.44.E-01	9.98.E-01
Phycodnaviridae	L7_Lactobacillus_crispatus	-0.001	0.039	9.78.E-01	9.99.E-01
Podoviridae	L7_Lactobacillus_crispatus	-0.057	0.045	2.06.E-01	8.76.E-01
Siphoviridae	L7_Lactobacillus_crispatus	0.107	0.040	7.71.E-03	4.66.E-01
Autographiviridae	L7_Peptostreptococcus_anaerobius	0.000	0.041	9.91.E-01	9.99.E-01
crAss_like_phage	L7_Peptostreptococcus_anaerobius	0.093	0.045	3.86.E-02	7.03.E-01
Herelleviridae	L7_Peptostreptococcus_anaerobius	0.005	0.042	9.01.E-01	9.97.E-01
Microviridae	L7_Peptostreptococcus_anaerobius	-0.059	0.042	1.68.E-01	8.44.E-01
Myoviridae	L7_Peptostreptococcus_anaerobius	-0.022	0.047	6.50.E-01	9.79.E-01
Phycodnaviridae	L7_Peptostreptococcus_anaerobius	-0.004	0.042	9.33.E-01	9.98.E-01
Podoviridae	L7_Peptostreptococcus_anaerobius	-0.019	0.049	6.96.E-01	9.81.E-01
Siphoviridae	L7_Peptostreptococcus_anaerobius	-0.006	0.044	9.00.E-01	9.97.E-01
Autographiviridae	L7_Oscillospiraceae_bacterium	0.014	0.031	6.50.E-01	9.79.E-01
crAss_like_phage	L7_Oscillospiraceae_bacterium	-0.014	0.034	6.89.E-01	9.80.E-01
Herelleviridae	L7_Oscillospiraceae_bacterium	-0.050	0.032	1.17.E-01	7.86.E-01
Microviridae	L7_Oscillospiraceae_bacterium	-0.017	0.032	6.06.E-01	9.73.E-01
Myoviridae	L7_Oscillospiraceae_bacterium	-0.060	0.036	9.19.E-02	7.59.E-01
Phycodnaviridae	L7_Oscillospiraceae_bacterium	-0.005	0.032	8.66.E-01	9.96.E-01
Podoviridae	L7_Oscillospiraceae_bacterium	0.032	0.037	3.88.E-01	9.15.E-01
Siphoviridae	L7_Oscillospiraceae_bacterium	0.054	0.033	1.00.E-01	7.79.E-01
Autographiviridae	L7_Odoribacter_laneus	0.004	0.041	9.30.E-01	9.98.E-01
crAss_like_phage	L7_Odoribacter_laneus	0.033	0.045	4.66.E-01	9.46.E-01
Herelleviridae	L7_Odoribacter_laneus	0.056	0.042	1.82.E-01	8.60.E-01
Microviridae	L7_Odoribacter_laneus	0.027	0.042	5.28.E-01	9.57.E-01
Myoviridae	L7_Odoribacter_laneus	-0.024	0.047	6.15.E-01	9.75.E-01
Phycodnaviridae	L7_Odoribacter_laneus	0.010	0.042	8.04.E-01	9.93.E-01
Podoviridae	L7_Odoribacter_laneus	0.029	0.049	5.59.E-01	9.61.E-01
Siphoviridae	L7_Odoribacter_laneus	0.004	0.044	9.26.E-01	9.98.E-01
Autographiviridae	L7_Paraclostridium_bifermentans	0.023	0.028	4.12.E-01	9.18.E-01
crAss_like_phage	L7_Paraclostridium_bifermentans	0.000	0.031	9.96.E-01	9.99.E-01
Herelleviridae	L7_Paraclostridium_bifermentans	0.014	0.029	6.17.E-01	9.75.E-01
Microviridae	L7_Paraclostridium_bifermentans	-0.039	0.029	1.81.E-01	8.60.E-01
Myoviridae	L7_Paraclostridium_bifermentans	-0.033	0.032	2.99.E-01	9.15.E-01
Phycodnaviridae	L7_Paraclostridium_bifermentans	0.030	0.028	2.95.E-01	9.15.E-01
Podoviridae	L7_Paraclostridium_bifermentans	0.032	0.033	3.32.E-01	9.15.E-01
Siphoviridae	L7_Paraclostridium_bifermentans	0.042	0.030	1.52.E-01	8.24.E-01
Autographiviridae	L7_Lactobacillus_amylovorus	-0.006	0.038	8.75.E-01	9.96.E-01
crAss_like_phage	L7_Lactobacillus_amylovorus	-0.013	0.042	7.53.E-01	9.89.E-01
Herelleviridae	L7_Lactobacillus_amylovorus	-0.032	0.040	4.19.E-01	9.20.E-01
Microviridae	L7_Lactobacillus_amylovorus	-0.063	0.040	1.16.E-01	7.85.E-01
Myoviridae	L7_Lactobacillus_amylovorus	-0.071	0.044	1.11.E-01	7.82.E-01
Phycodnaviridae	L7_Lactobacillus_amylovorus	-0.056	0.039	1.54.E-01	8.26.E-01
Podoviridae	L7_Lactobacillus_amylovorus	-0.054	0.046	2.36.E-01	8.85.E-01
Siphoviridae	L7_Lactobacillus_amylovorus	-0.039	0.041	3.41.E-01	9.15.E-01
Autographiviridae	L7_Roseburia_inulinivorans	0.048	0.038	2.02.E-01	8.75.E-01
crAss_like_phage	L7_Roseburia_inulinivorans	-0.033	0.042	4.31.E-01	9.25.E-01
Herelleviridae	L7_Roseburia_inulinivorans	-0.008	0.039	8.47.E-01	9.95.E-01
Microviridae	L7_Roseburia_inulinivorans	0.003	0.039	9.30.E-01	9.98.E-01
Myoviridae	L7_Roseburia_inulinivorans	-0.068	0.044	1.18.E-01	7.86.E-01
Phycodnaviridae	L7_Roseburia_inulinivorans	-0.012	0.039	7.61.E-01	9.91.E-01
Podoviridae	L7_Roseburia_inulinivorans	-0.059	0.045	1.94.E-01	8.62.E-01

Siphoviridae	L7_Roseburia_inulinivorans	-0.051	0.040	2.04.E-01	8.76.E-01
Autographiviridae	L7_Anaerotruncus_colihominis	-0.005	0.027	8.57.E-01	9.96.E-01
crAss_like_phage	L7_Anaerotruncus_colihominis	0.026	0.030	3.79.E-01	9.15.E-01
Herelleviridae	L7_Anaerotruncus_colihominis	-0.026	0.028	3.54.E-01	9.15.E-01
Microviridae	L7_Anaerotruncus_colihominis	-0.002	0.028	9.39.E-01	9.98.E-01
Myoviridae	L7_Anaerotruncus_colihominis	0.034	0.031	2.84.E-01	9.15.E-01
Phycodnaviridae	L7_Anaerotruncus_colihominis	0.014	0.028	6.12.E-01	9.75.E-01
Podoviridae	L7_Anaerotruncus_colihominis	0.020	0.032	5.42.E-01	9.60.E-01
Siphoviridae	L7_Anaerotruncus_colihominis	0.026	0.029	3.67.E-01	9.15.E-01
Autographiviridae	L7_Roseburia_hominis	-0.027	0.034	4.25.E-01	9.23.E-01
crAss_like_phage	L7_Roseburia_hominis	-0.078	0.037	3.59.E-02	6.90.E-01
Herelleviridae	L7_Roseburia_hominis	-0.031	0.035	3.77.E-01	9.15.E-01
Microviridae	L7_Roseburia_hominis	0.005	0.035	8.80.E-01	9.96.E-01
Myoviridae	L7_Roseburia_hominis	-0.066	0.039	9.00.E-02	7.58.E-01
Phycodnaviridae	L7_Roseburia_hominis	-0.011	0.035	7.56.E-01	9.89.E-01
Podoviridae	L7_Roseburia_hominis	-0.079	0.040	4.97.E-02	7.22.E-01
Siphoviridae	L7_Roseburia_hominis	-0.004	0.036	9.12.E-01	9.98.E-01
Autographiviridae	L7_Enterococcus_casseliflavus	-0.002	0.037	9.57.E-01	9.98.E-01
crAss_like_phage	L7_Enterococcus_casseliflavus	-0.064	0.040	1.12.E-01	7.83.E-01
Herelleviridae	L7_Enterococcus_casseliflavus	0.063	0.038	9.51.E-02	7.64.E-01
Microviridae	L7_Enterococcus_casseliflavus	-0.011	0.038	7.75.E-01	9.92.E-01
Myoviridae	L7_Enterococcus_casseliflavus	-0.026	0.042	5.47.E-01	9.60.E-01
Phycodnaviridae	L7_Enterococcus_casseliflavus	0.024	0.038	5.27.E-01	9.57.E-01
Podoviridae	L7_Enterococcus_casseliflavus	0.078	0.044	7.56.E-02	7.30.E-01
Siphoviridae	L7_Enterococcus_casseliflavus	0.020	0.039	6.15.E-01	9.75.E-01
Autographiviridae	L7_Streptococcus_intermedius	-0.045	0.027	9.15.E-02	7.59.E-01
crAss_like_phage	L7_Streptococcus_intermedius	0.041	0.029	1.62.E-01	8.38.E-01
Herelleviridae	L7_Streptococcus_intermedius	0.020	0.028	4.79.E-01	9.53.E-01
Microviridae	L7_Streptococcus_intermedius	0.043	0.028	1.22.E-01	7.93.E-01
Myoviridae	L7_Streptococcus_intermedius	0.056	0.031	7.20.E-02	7.25.E-01
Phycodnaviridae	L7_Streptococcus_intermedius	0.036	0.027	1.88.E-01	8.61.E-01
Podoviridae	L7_Streptococcus_intermedius	0.030	0.032	3.52.E-01	9.15.E-01
Siphoviridae	L7_Streptococcus_intermedius	0.030	0.028	2.97.E-01	9.15.E-01
Autographiviridae	L7_Bilophila_sp.	0.066	0.038	8.14.E-02	7.47.E-01
crAss_like_phage	L7_Bilophila_sp.	-0.002	0.042	9.60.E-01	9.98.E-01
Herelleviridae	L7_Bilophila_sp.	-0.025	0.039	5.19.E-01	9.57.E-01
Microviridae	L7_Bilophila_sp.	0.034	0.039	3.86.E-01	9.15.E-01
Myoviridae	L7_Bilophila_sp.	0.026	0.044	5.58.E-01	9.61.E-01
Phycodnaviridae	L7_Bilophila_sp.	0.003	0.039	9.46.E-01	9.98.E-01
Podoviridae	L7_Bilophila_sp.	-0.014	0.045	7.50.E-01	9.89.E-01
Siphoviridae	L7_Bilophila_sp.	-0.010	0.040	7.98.E-01	9.92.E-01
Autographiviridae	L7_Akkermansia_muciniphila	0.003	0.040	9.46.E-01	9.98.E-01
crAss_like_phage	L7_Akkermansia_muciniphila	-0.006	0.044	8.98.E-01	9.97.E-01
Herelleviridae	L7_Akkermansia_muciniphila	0.027	0.041	5.18.E-01	9.57.E-01
Microviridae	L7_Akkermansia_muciniphila	-0.079	0.041	5.72.E-02	7.24.E-01
Myoviridae	L7_Akkermansia_muciniphila	0.036	0.046	4.43.E-01	9.32.E-01
Phycodnaviridae	L7_Akkermansia_muciniphila	0.033	0.041	4.15.E-01	9.18.E-01
Podoviridae	L7_Akkermansia_muciniphila	0.085	0.048	7.56.E-02	7.30.E-01
Siphoviridae	L7_Akkermansia_muciniphila	-0.011	0.043	7.91.E-01	9.92.E-01
Autographiviridae	L7_Eubacterium_ventriosum	0.076	0.036	3.62.E-02	6.90.E-01
crAss_like_phage	L7_Eubacterium_ventriosum	-0.012	0.040	7.62.E-01	9.91.E-01
Herelleviridae	L7_Eubacterium_ventriosum	0.087	0.038	2.10.E-02	6.06.E-01
Microviridae	L7_Eubacterium_ventriosum	0.021	0.038	5.83.E-01	9.64.E-01
Myoviridae	L7_Eubacterium_ventriosum	-0.043	0.042	3.07.E-01	9.15.E-01
Phycodnaviridae	L7_Eubacterium_ventriosum	-0.018	0.037	6.26.E-01	9.76.E-01
Podoviridae	L7_Eubacterium_ventriosum	-0.013	0.044	7.62.E-01	9.91.E-01
Siphoviridae	L7_Eubacterium_ventriosum	-0.010	0.039	8.00.E-01	9.92.E-01
Autographiviridae	L7_Slackia_piriformis	0.030	0.039	4.45.E-01	9.33.E-01
crAss_like_phage	L7_Slackia_piriformis	-0.022	0.043	6.15.E-01	9.75.E-01
Herelleviridae	L7_Slackia_piriformis	-0.066	0.040	1.02.E-01	7.81.E-01
Microviridae	L7_Slackia_piriformis	0.048	0.041	2.41.E-01	8.85.E-01
Myoviridae	L7_Slackia_piriformis	-0.028	0.045	5.34.E-01	9.59.E-01
Phycodnaviridae	L7_Slackia_piriformis	0.037	0.040	3.50.E-01	9.15.E-01
Podoviridae	L7_Slackia_piriformis	0.028	0.047	5.55.E-01	9.61.E-01
Siphoviridae	L7_Slackia_piriformis	-0.039	0.042	3.45.E-01	9.15.E-01

Autographiviridae	L7_Lactobacillus_mucosae	-0.021	0.038	5.80.E-01	9.64.E-01
crAss_like_phage	L7_Lactobacillus_mucosae	-0.063	0.042	1.33.E-01	8.05.E-01
Herelleviridae	L7_Lactobacillus_mucosae	-0.071	0.039	6.92.E-02	7.24.E-01
Microviridae	L7_Lactobacillus_mucosae	0.001	0.039	9.79.E-01	9.99.E-01
Myoviridae	L7_Lactobacillus_mucosae	-0.031	0.044	4.79.E-01	9.53.E-01
Phycodnaviridae	L7_Lactobacillus_mucosae	0.019	0.039	6.23.E-01	9.76.E-01
Podoviridae	L7_Lactobacillus_mucosae	-0.026	0.045	5.60.E-01	9.61.E-01
Siphoviridae	L7_Lactobacillus_mucosae	-0.062	0.040	1.27.E-01	7.98.E-01
Autographiviridae	L7_Dielma_fastidiosa	-0.068	0.038	7.15.E-02	7.24.E-01
crAss_like_phage	L7_Dielma_fastidiosa	0.026	0.042	5.26.E-01	9.57.E-01
Herelleviridae	L7_Dielma_fastidiosa	0.011	0.039	7.84.E-01	9.92.E-01
Microviridae	L7_Dielma_fastidiosa	0.008	0.039	8.43.E-01	9.95.E-01
Myoviridae	L7_Dielma_fastidiosa	0.015	0.044	7.40.E-01	9.88.E-01
Phycodnaviridae	L7_Dielma_fastidiosa	0.070	0.039	7.01.E-02	7.24.E-01
Podoviridae	L7_Dielma_fastidiosa	0.048	0.045	2.90.E-01	9.15.E-01
Siphoviridae	L7_Dielma_fastidiosa	0.089	0.040	2.75.E-02	6.58.E-01
Autographiviridae	L7_Actinomyces_graevenitzii	-0.082	0.038	3.24.E-02	6.75.E-01
crAss_like_phage	L7_Actinomyces_graevenitzii	-0.011	0.042	7.88.E-01	9.92.E-01
Herelleviridae	L7_Actinomyces_graevenitzii	-0.028	0.040	4.91.E-01	9.55.E-01
Microviridae	L7_Actinomyces_graevenitzii	-0.052	0.040	1.92.E-01	8.61.E-01
Myoviridae	L7_Actinomyces_graevenitzii	0.007	0.045	8.79.E-01	9.96.E-01
Phycodnaviridae	L7_Actinomyces_graevenitzii	0.043	0.039	2.76.E-01	9.05.E-01
Podoviridae	L7_Actinomyces_graevenitzii	0.011	0.046	8.06.E-01	9.93.E-01
Siphoviridae	L7_Actinomyces_graevenitzii	-0.081	0.041	4.81.E-02	7.22.E-01
Autographiviridae	L7_Enterobacter_sp.	-0.043	0.023	5.90.E-02	7.24.E-01
crAss_like_phage	L7_Enterobacter_sp.	0.037	0.025	1.36.E-01	8.07.E-01
Herelleviridae	L7_Enterobacter_sp.	0.013	0.024	5.83.E-01	9.64.E-01
Microviridae	L7_Enterobacter_sp.	0.034	0.024	1.51.E-01	8.22.E-01
Myoviridae	L7_Enterobacter_sp.	-0.009	0.026	7.46.E-01	9.89.E-01
Phycodnaviridae	L7_Enterobacter_sp.	-0.016	0.023	5.02.E-01	9.55.E-01
Podoviridae	L7_Enterobacter_sp.	0.011	0.027	6.73.E-01	9.80.E-01
Siphoviridae	L7_Enterobacter_sp.	0.006	0.024	8.08.E-01	9.93.E-01
Autographiviridae	L7_Coprococcus_sp.	0.031	0.041	4.53.E-01	9.38.E-01
crAss_like_phage	L7_Coprococcus_sp.	-0.036	0.045	4.17.E-01	9.19.E-01
Herelleviridae	L7_Coprococcus_sp.	0.077	0.042	6.91.E-02	7.24.E-01
Microviridae	L7_Coprococcus_sp.	0.036	0.042	3.89.E-01	9.15.E-01
Myoviridae	L7_Coprococcus_sp.	-0.034	0.047	4.67.E-01	9.47.E-01
Phycodnaviridae	L7_Coprococcus_sp.	-0.010	0.042	8.13.E-01	9.93.E-01
Podoviridae	L7_Coprococcus_sp.	-0.036	0.049	4.58.E-01	9.41.E-01
Siphoviridae	L7_Coprococcus_sp.	0.022	0.043	6.16.E-01	9.75.E-01
Autographiviridae	L7_Roseburia_intestinalis	0.050	0.039	2.03.E-01	8.75.E-01
crAss_like_phage	L7_Roseburia_intestinalis	0.037	0.043	3.94.E-01	9.15.E-01
Herelleviridae	L7_Roseburia_intestinalis	-0.023	0.041	5.76.E-01	9.62.E-01
Microviridae	L7_Roseburia_intestinalis	-0.013	0.041	7.43.E-01	9.89.E-01
Myoviridae	L7_Roseburia_intestinalis	-0.079	0.046	8.49.E-02	7.50.E-01
Phycodnaviridae	L7_Roseburia_intestinalis	0.007	0.040	8.55.E-01	9.96.E-01
Podoviridae	L7_Roseburia_intestinalis	-0.019	0.047	6.91.E-01	9.80.E-01
Siphoviridae	L7_Roseburia_intestinalis	-0.017	0.042	6.81.E-01	9.80.E-01
Autographiviridae	L7_Eubacterium_eligens	-0.071	0.038	5.87.E-02	7.24.E-01
crAss_like_phage	L7_Eubacterium_eligens	-0.068	0.041	9.97.E-02	7.79.E-01
Herelleviridae	L7_Eubacterium_eligens	0.077	0.039	4.82.E-02	7.22.E-01
Microviridae	L7_Eubacterium_eligens	0.069	0.039	7.90.E-02	7.43.E-01
Myoviridae	L7_Eubacterium_eligens	-0.073	0.044	9.54.E-02	7.65.E-01
Phycodnaviridae	L7_Eubacterium_eligens	0.014	0.039	7.10.E-01	9.84.E-01
Podoviridae	L7_Eubacterium_eligens	-0.047	0.045	3.00.E-01	9.15.E-01
Siphoviridae	L7_Eubacterium_eligens	0.007	0.040	8.65.E-01	9.96.E-01
Autographiviridae	L7_Leclercia_adecarboxylata	0.005	0.031	8.79.E-01	9.96.E-01
crAss_like_phage	L7_Leclercia_adecarboxylata	-0.003	0.034	9.33.E-01	9.98.E-01
Herelleviridae	L7_Leclercia_adecarboxylata	-0.018	0.032	5.68.E-01	9.61.E-01
Microviridae	L7_Leclercia_adecarboxylata	-0.002	0.032	9.40.E-01	9.98.E-01
Myoviridae	L7_Leclercia_adecarboxylata	-0.001	0.036	9.71.E-01	9.99.E-01
Phycodnaviridae	L7_Leclercia_adecarboxylata	-0.008	0.032	7.95.E-01	9.92.E-01
Podoviridae	L7_Leclercia_adecarboxylata	0.109	0.037	3.33.E-03	3.95.E-01
Siphoviridae	L7_Leclercia_adecarboxylata	0.013	0.033	7.00.E-01	9.81.E-01
Autographiviridae	L7_Adlercreutzia_equolifaciens	0.014	0.036	6.97.E-01	9.81.E-01

crAss_like_phage	L7_Adlercreutzia_equolifaciens	-0.039	0.039	3.27.E-01	9.15.E-01
Herelleviridae	L7_Adlercreutzia_equolifaciens	-0.014	0.037	7.15.E-01	9.86.E-01
Microviridae	L7_Adlercreutzia_equolifaciens	-0.006	0.037	8.78.E-01	9.96.E-01
Myoviridae	L7_Adlercreutzia_equolifaciens	-0.015	0.041	7.12.E-01	9.85.E-01
Phycodnaviridae	L7_Adlercreutzia_equolifaciens	0.035	0.037	3.35.E-01	9.15.E-01
Podoviridae	L7_Adlercreutzia_equolifaciens	-0.023	0.043	5.90.E-01	9.68.E-01
Siphoviridae	L7_Adlercreutzia_equolifaciens	0.056	0.038	1.44.E-01	8.12.E-01
Autographiviridae	L7_Collinsella_sp.	0.019	0.034	5.86.E-01	9.65.E-01
crAss_like_phage	L7_Collinsella_sp.	-0.041	0.038	2.77.E-01	9.05.E-01
Herelleviridae	L7_Collinsella_sp.	0.042	0.036	2.39.E-01	8.85.E-01
Microviridae	L7_Collinsella_sp.	-0.031	0.036	3.90.E-01	9.15.E-01
Myoviridae	L7_Collinsella_sp.	-0.049	0.040	2.17.E-01	8.79.E-01
Phycodnaviridae	L7_Collinsella_sp.	0.026	0.035	4.68.E-01	9.50.E-01
Podoviridae	L7_Collinsella_sp.	-0.035	0.041	3.99.E-01	9.15.E-01
Siphoviridae	L7_Collinsella_sp.	-0.036	0.037	3.28.E-01	9.15.E-01
Autographiviridae	L7_Veillonella_seminalis	0.022	0.045	6.20.E-01	9.75.E-01
crAss_like_phage	L7_Veillonella_seminalis	0.044	0.050	3.78.E-01	9.15.E-01
Herelleviridae	L7_Veillonella_seminalis	-0.091	0.047	5.09.E-02	7.22.E-01
Microviridae	L7_Veillonella_seminalis	-0.049	0.047	2.98.E-01	9.15.E-01
Myoviridae	L7_Veillonella_seminalis	-0.072	0.052	1.67.E-01	8.44.E-01
Phycodnaviridae	L7_Veillonella_seminalis	-0.062	0.046	1.82.E-01	8.60.E-01
Podoviridae	L7_Veillonella_seminalis	-0.120	0.054	2.69.E-02	6.58.E-01
Siphoviridae	L7_Veillonella_seminalis	-0.068	0.048	1.61.E-01	8.38.E-01
Autographiviridae	L7_Aeromonas_caviae	-0.026	0.040	5.20.E-01	9.57.E-01
crAss_like_phage	L7_Aeromonas_caviae	-0.071	0.044	1.04.E-01	7.82.E-01
Herelleviridae	L7_Aeromonas_caviae	-0.098	0.041	1.78.E-02	5.86.E-01
Microviridae	L7_Aeromonas_caviae	-0.012	0.041	7.70.E-01	9.91.E-01
Myoviridae	L7_Aeromonas_caviae	-0.084	0.046	6.91.E-02	7.24.E-01
Phycodnaviridae	L7_Aeromonas_caviae	0.049	0.041	2.34.E-01	8.85.E-01
Podoviridae	L7_Aeromonas_caviae	-0.051	0.048	2.87.E-01	9.15.E-01
Siphoviridae	L7_Aeromonas_caviae	0.042	0.043	3.26.E-01	9.15.E-01
Autographiviridae	L7_Clostridium_spiroforme	-0.019	0.040	6.31.E-01	9.76.E-01
crAss_like_phage	L7_Clostridium_spiroforme	-0.042	0.044	3.42.E-01	9.15.E-01
Herelleviridae	L7_Clostridium_spiroforme	-0.074	0.041	7.54.E-02	7.30.E-01
Microviridae	L7_Clostridium_spiroforme	-0.025	0.042	5.47.E-01	9.60.E-01
Myoviridae	L7_Clostridium_spiroforme	0.008	0.047	8.62.E-01	9.96.E-01
Phycodnaviridae	L7_Clostridium_spiroforme	-0.034	0.041	4.09.E-01	9.17.E-01
Podoviridae	L7_Clostridium_spiroforme	-0.052	0.048	2.77.E-01	9.05.E-01
Siphoviridae	L7_Clostridium_spiroforme	0.060	0.043	1.61.E-01	8.38.E-01
Autographiviridae	L7_Enterococcus_hirae	0.061	0.041	1.39.E-01	8.10.E-01
crAss_like_phage	L7_Enterococcus_hirae	-0.031	0.046	4.95.E-01	9.55.E-01
Herelleviridae	L7_Enterococcus_hirae	0.001	0.043	9.75.E-01	9.99.E-01
Microviridae	L7_Enterococcus_hirae	-0.054	0.043	2.11.E-01	8.78.E-01
Myoviridae	L7_Enterococcus_hirae	0.013	0.048	7.91.E-01	9.92.E-01
Phycodnaviridae	L7_Enterococcus_hirae	-0.001	0.043	9.87.E-01	9.99.E-01
Podoviridae	L7_Enterococcus_hirae	0.015	0.050	7.58.E-01	9.90.E-01
Siphoviridae	L7_Enterococcus_hirae	-0.085	0.044	5.46.E-02	7.23.E-01
Autographiviridae	L7_Gabonibacter_massiliensis	0.008	0.038	8.37.E-01	9.94.E-01
crAss_like_phage	L7_Gabonibacter_massiliensis	0.018	0.042	6.69.E-01	9.80.E-01
Herelleviridae	L7_Gabonibacter_massiliensis	0.022	0.039	5.69.E-01	9.61.E-01
Microviridae	L7_Gabonibacter_massiliensis	-0.019	0.039	6.22.E-01	9.76.E-01
Myoviridae	L7_Gabonibacter_massiliensis	-0.033	0.044	4.45.E-01	9.34.E-01
Phycodnaviridae	L7_Gabonibacter_massiliensis	0.057	0.038	1.41.E-01	8.10.E-01
Podoviridae	L7_Gabonibacter_massiliensis	-0.053	0.045	2.43.E-01	8.87.E-01
Siphoviridae	L7_Gabonibacter_massiliensis	0.025	0.040	5.37.E-01	9.60.E-01
Autographiviridae	L7_Catenibacterium_sp.	0.005	0.040	9.08.E-01	9.98.E-01
crAss_like_phage	L7_Catenibacterium_sp.	-0.012	0.045	7.81.E-01	9.92.E-01
Herelleviridae	L7_Catenibacterium_sp.	-0.006	0.042	8.95.E-01	9.97.E-01
Microviridae	L7_Catenibacterium_sp.	0.024	0.042	5.69.E-01	9.61.E-01
Myoviridae	L7_Catenibacterium_sp.	0.046	0.047	3.26.E-01	9.15.E-01
Phycodnaviridae	L7_Catenibacterium_sp.	-0.026	0.042	5.35.E-01	9.59.E-01
Podoviridae	L7_Catenibacterium_sp.	-0.001	0.049	9.86.E-01	9.99.E-01
Siphoviridae	L7_Catenibacterium_sp.	-0.045	0.043	3.04.E-01	9.15.E-01
Autographiviridae	L7_Clostridium_bolteae	0.020	0.035	5.60.E-01	9.61.E-01
crAss_like_phage	L7_Clostridium_bolteae	-0.016	0.039	6.79.E-01	9.80.E-01

Herelleviridae	L7_Clostridium_bolteae	0.037	0.036	3.12.E-01	9.15.E-01
Microviridae	L7_Clostridium_bolteae	0.006	0.036	8.64.E-01	9.96.E-01
Myoviridae	L7_Clostridium_bolteae	0.000	0.041	9.97.E-01	9.99.E-01
Phycodnaviridae	L7_Clostridium_bolteae	0.057	0.036	1.12.E-01	7.83.E-01
Podoviridae	L7_Clostridium_bolteae	0.083	0.042	4.87.E-02	7.22.E-01
Siphoviridae	L7_Clostridium_bolteae	-0.004	0.037	9.13.E-01	9.98.E-01
Autographiviridae	L7_Butyricimonas_virosa	0.008	0.022	7.03.E-01	9.81.E-01
crAss_like_phage	L7_Butyricimonas_virosa	-0.015	0.024	5.50.E-01	9.60.E-01
Herelleviridae	L7_Butyricimonas_virosa	0.006	0.023	7.90.E-01	9.92.E-01
Microviridae	L7_Butyricimonas_virosa	0.023	0.023	3.09.E-01	9.15.E-01
Myoviridae	L7_Butyricimonas_virosa	0.008	0.026	7.65.E-01	9.91.E-01
Phycodnaviridae	L7_Butyricimonas_virosa	-0.022	0.023	3.43.E-01	9.15.E-01
Podoviridae	L7_Butyricimonas_virosa	-0.032	0.026	2.33.E-01	8.83.E-01
Siphoviridae	L7_Butyricimonas_virosa	0.068	0.023	3.76.E-03	4.02.E-01
Autographiviridae	L7_Blautia_sp.	-0.013	0.032	6.89.E-01	9.80.E-01
crAss_like_phage	L7_Blautia_sp.	-0.016	0.035	6.46.E-01	9.79.E-01
Herelleviridae	L7_Blautia_sp.	-0.011	0.033	7.41.E-01	9.89.E-01
Microviridae	L7_Blautia_sp.	0.013	0.033	6.98.E-01	9.81.E-01
Myoviridae	L7_Blautia_sp.	-0.001	0.037	9.80.E-01	9.99.E-01
Phycodnaviridae	L7_Blautia_sp.	0.025	0.033	4.53.E-01	9.38.E-01
Podoviridae	L7_Blautia_sp.	0.035	0.038	3.61.E-01	9.15.E-01
Siphoviridae	L7_Blautia_sp.	0.038	0.034	2.69.E-01	9.01.E-01
Autographiviridae	L7_Holdemania_massiliensis	-0.058	0.040	1.47.E-01	8.16.E-01
crAss_like_phage	L7_Holdemania_massiliensis	-0.010	0.044	8.16.E-01	9.93.E-01
Herelleviridae	L7_Holdemania_massiliensis	0.004	0.042	9.23.E-01	9.98.E-01
Microviridae	L7_Holdemania_massiliensis	0.067	0.042	1.10.E-01	7.82.E-01
Myoviridae	L7_Holdemania_massiliensis	0.003	0.046	9.52.E-01	9.98.E-01
Phycodnaviridae	L7_Holdemania_massiliensis	-0.004	0.041	9.24.E-01	9.98.E-01
Podoviridae	L7_Holdemania_massiliensis	-0.014	0.048	7.73.E-01	9.92.E-01
Siphoviridae	L7_Holdemania_massiliensis	0.068	0.043	1.12.E-01	7.83.E-01
Autographiviridae	L7_Bacteroides_fragilis	-0.019	0.029	5.13.E-01	9.57.E-01
crAss_like_phage	L7_Bacteroides_fragilis	-0.059	0.032	6.93.E-02	7.24.E-01
Herelleviridae	L7_Bacteroides_fragilis	-0.019	0.031	5.42.E-01	9.60.E-01
Microviridae	L7_Bacteroides_fragilis	0.002	0.031	9.41.E-01	9.98.E-01
Myoviridae	L7_Bacteroides_fragilis	-0.052	0.034	1.27.E-01	7.98.E-01
Phycodnaviridae	L7_Bacteroides_fragilis	0.018	0.030	5.41.E-01	9.60.E-01
Podoviridae	L7_Bacteroides_fragilis	-0.080	0.035	2.28.E-02	6.19.E-01
Siphoviridae	L7_Bacteroides_fragilis	0.009	0.031	7.69.E-01	9.91.E-01
Autographiviridae	L7_Rikenella_microfusus	0.041	0.029	1.56.E-01	8.30.E-01
crAss_like_phage	L7_Rikenella_microfusus	-0.012	0.032	6.98.E-01	9.81.E-01
Herelleviridae	L7_Rikenella_microfusus	0.025	0.030	4.14.E-01	9.18.E-01
Microviridae	L7_Rikenella_microfusus	0.003	0.030	9.24.E-01	9.98.E-01
Myoviridae	L7_Rikenella_microfusus	-0.009	0.034	7.85.E-01	9.92.E-01
Phycodnaviridae	L7_Rikenella_microfusus	-0.037	0.030	2.19.E-01	8.79.E-01
Podoviridae	L7_Rikenella_microfusus	-0.024	0.035	5.00.E-01	9.55.E-01
Siphoviridae	L7_Rikenella_microfusus	-0.057	0.031	6.35.E-02	7.24.E-01
Autographiviridae	L7_Mitsuokella_jalaludinii	-0.019	0.040	6.28.E-01	9.76.E-01
crAss_like_phage	L7_Mitsuokella_jalaludinii	-0.036	0.044	4.07.E-01	9.17.E-01
Herelleviridae	L7_Mitsuokella_jalaludinii	-0.062	0.041	1.29.E-01	7.99.E-01
Microviridae	L7_Mitsuokella_jalaludinii	-0.005	0.041	8.98.E-01	9.97.E-01
Myoviridae	L7_Mitsuokella_jalaludinii	0.072	0.046	1.18.E-01	7.89.E-01
Phycodnaviridae	L7_Mitsuokella_jalaludinii	0.005	0.041	8.99.E-01	9.97.E-01
Podoviridae	L7_Mitsuokella_jalaludinii	-0.099	0.047	3.63.E-02	6.90.E-01
Siphoviridae	L7_Mitsuokella_jalaludinii	0.020	0.042	6.40.E-01	9.79.E-01
Autographiviridae	L7_Clostridium_citroniae	-0.008	0.036	8.27.E-01	9.93.E-01
crAss_like_phage	L7_Clostridium_citroniae	0.017	0.040	6.76.E-01	9.80.E-01
Herelleviridae	L7_Clostridium_citroniae	0.037	0.038	3.22.E-01	9.15.E-01
Microviridae	L7_Clostridium_citroniae	-0.019	0.038	6.21.E-01	9.76.E-01
Myoviridae	L7_Clostridium_citroniae	-0.001	0.042	9.75.E-01	9.99.E-01
Phycodnaviridae	L7_Clostridium_citroniae	0.002	0.037	9.60.E-01	9.98.E-01
Podoviridae	L7_Clostridium_citroniae	0.096	0.043	2.75.E-02	6.58.E-01
Siphoviridae	L7_Clostridium_citroniae	-0.031	0.039	4.32.E-01	9.25.E-01
Autographiviridae	L7_bacterium_LF.3	0.004	0.039	9.22.E-01	9.98.E-01
crAss_like_phage	L7_bacterium_LF.3	-0.031	0.043	4.74.E-01	9.52.E-01
Herelleviridae	L7_bacterium_LF.3	0.009	0.040	8.27.E-01	9.93.E-01

Microviridae	L7_bacterium_LF.3	-0.006	0.040	8.84.E-01	9.96.E-01
Myoviridae	L7_bacterium_LF.3	0.000	0.045	9.97.E-01	9.99.E-01
Phycodnaviridae	L7_bacterium_LF.3	-0.025	0.040	5.26.E-01	9.57.E-01
Podoviridae	L7_bacterium_LF.3	-0.024	0.046	6.00.E-01	9.70.E-01
Siphoviridae	L7_bacterium_LF.3	-0.028	0.041	4.92.E-01	9.55.E-01
Autographiviridae	L7_Negativicoccus_succinicivorans	0.015	0.039	6.97.E-01	9.81.E-01
crAss_like_phage	L7_Negativicoccus_succinicivorans	-0.107	0.043	1.27.E-02	5.43.E-01
Herelleviridae	L7_Negativicoccus_succinicivorans	0.008	0.040	8.38.E-01	9.94.E-01
Microviridae	L7_Negativicoccus_succinicivorans	-0.055	0.040	1.73.E-01	8.55.E-01
Myoviridae	L7_Negativicoccus_succinicivorans	0.046	0.045	3.08.E-01	9.15.E-01
Phycodnaviridae	L7_Negativicoccus_succinicivorans	0.024	0.040	5.45.E-01	9.60.E-01
Podoviridae	L7_Negativicoccus_succinicivorans	-0.048	0.047	3.05.E-01	9.15.E-01
Siphoviridae	L7_Negativicoccus_succinicivorans	0.019	0.042	6.48.E-01	9.79.E-01
Autographiviridae	L7_Oxalobacter_formigenes	-0.064	0.035	6.63.E-02	7.24.E-01
crAss_like_phage	L7_Oxalobacter_formigenes	0.002	0.039	9.48.E-01	9.98.E-01
Herelleviridae	L7_Oxalobacter_formigenes	-0.061	0.036	9.33.E-02	7.60.E-01
Microviridae	L7_Oxalobacter_formigenes	0.038	0.036	3.01.E-01	9.15.E-01
Myoviridae	L7_Oxalobacter_formigenes	0.002	0.040	9.54.E-01	9.98.E-01
Phycodnaviridae	L7_Oxalobacter_formigenes	-0.052	0.036	1.50.E-01	8.22.E-01
Podoviridae	L7_Oxalobacter_formigenes	-0.047	0.042	2.64.E-01	9.01.E-01
Siphoviridae	L7_Oxalobacter_formigenes	-0.039	0.037	2.98.E-01	9.15.E-01
Autographiviridae	L7_Gardnerella_vaginalis	0.049	0.039	2.19.E-01	8.79.E-01
crAss_like_phage	L7_Gardnerella_vaginalis	0.001	0.044	9.87.E-01	9.99.E-01
Herelleviridae	L7_Gardnerella_vaginalis	0.010	0.041	8.07.E-01	9.93.E-01
Microviridae	L7_Gardnerella_vaginalis	0.012	0.041	7.67.E-01	9.91.E-01
Myoviridae	L7_Gardnerella_vaginalis	0.051	0.046	2.68.E-01	9.01.E-01
Phycodnaviridae	L7_Gardnerella_vaginalis	0.045	0.041	2.67.E-01	9.01.E-01
Podoviridae	L7_Gardnerella_vaginalis	0.038	0.047	4.24.E-01	9.23.E-01
Siphoviridae	L7_Gardnerella_vaginalis	-0.044	0.042	2.99.E-01	9.15.E-01
Autographiviridae	L7_Streptococcus_timonensis	0.024	0.027	3.73.E-01	9.15.E-01
crAss_like_phage	L7_Streptococcus_timonensis	0.011	0.030	7.12.E-01	9.85.E-01
Herelleviridae	L7_Streptococcus_timonensis	-0.012	0.028	6.83.E-01	9.80.E-01
Microviridae	L7_Streptococcus_timonensis	-0.017	0.029	5.47.E-01	9.60.E-01
Myoviridae	L7_Streptococcus_timonensis	0.012	0.032	7.10.E-01	9.84.E-01
Phycodnaviridae	L7_Streptococcus_timonensis	0.027	0.028	3.43.E-01	9.15.E-01
Podoviridae	L7_Streptococcus_timonensis	0.001	0.033	9.83.E-01	9.99.E-01
Siphoviridae	L7_Streptococcus_timonensis	0.042	0.029	1.55.E-01	8.26.E-01
Autographiviridae	L7_Sellimonas_intestinalis	0.064	0.036	7.51.E-02	7.30.E-01
crAss_like_phage	L7_Sellimonas_intestinalis	0.020	0.040	6.16.E-01	9.75.E-01
Herelleviridae	L7_Sellimonas_intestinalis	0.005	0.037	8.98.E-01	9.97.E-01
Microviridae	L7_Sellimonas_intestinalis	0.004	0.037	9.20.E-01	9.98.E-01
Myoviridae	L7_Sellimonas_intestinalis	0.125	0.041	2.58.E-03	3.52.E-01
Phycodnaviridae	L7_Sellimonas_intestinalis	-0.048	0.037	1.92.E-01	8.61.E-01
Podoviridae	L7_Sellimonas_intestinalis	-0.066	0.043	1.22.E-01	7.93.E-01
Siphoviridae	L7_Sellimonas_intestinalis	0.019	0.038	6.24.E-01	9.76.E-01
Autographiviridae	L7_Streptococcus_pneumoniae	0.015	0.023	5.21.E-01	9.57.E-01
crAss_like_phage	L7_Streptococcus_pneumoniae	-0.011	0.026	6.66.E-01	9.80.E-01
Herelleviridae	L7_Streptococcus_pneumoniae	-0.005	0.024	8.42.E-01	9.95.E-01
Microviridae	L7_Streptococcus_pneumoniae	-0.016	0.024	5.11.E-01	9.57.E-01
Myoviridae	L7_Streptococcus_pneumoniae	0.009	0.027	7.35.E-01	9.88.E-01
Phycodnaviridae	L7_Streptococcus_pneumoniae	-0.016	0.024	4.99.E-01	9.55.E-01
Podoviridae	L7_Streptococcus_pneumoniae	0.014	0.028	6.12.E-01	9.75.E-01
Siphoviridae	L7_Streptococcus_pneumoniae	-0.056	0.025	2.38.E-02	6.32.E-01
Autographiviridae	L7_Haemophilus_parainfluenzae	-0.010	0.033	7.59.E-01	9.91.E-01
crAss_like_phage	L7_Haemophilus_parainfluenzae	-0.030	0.036	4.06.E-01	9.17.E-01
Herelleviridae	L7_Haemophilus_parainfluenzae	0.069	0.034	4.23.E-02	7.18.E-01
Microviridae	L7_Haemophilus_parainfluenzae	0.005	0.034	8.77.E-01	9.96.E-01
Myoviridae	L7_Haemophilus_parainfluenzae	0.093	0.038	1.35.E-02	5.51.E-01
Phycodnaviridae	L7_Haemophilus_parainfluenzae	0.022	0.033	5.07.E-01	9.57.E-01
Podoviridae	L7_Haemophilus_parainfluenzae	0.010	0.039	7.97.E-01	9.92.E-01
Siphoviridae	L7_Haemophilus_parainfluenzae	-0.014	0.035	6.83.E-01	9.80.E-01
Autographiviridae	L7_Clostridium_hiranonis	-0.020	0.037	5.77.E-01	9.63.E-01
crAss_like_phage	L7_Clostridium_hiranonis	-0.002	0.041	9.55.E-01	9.98.E-01
Herelleviridae	L7_Clostridium_hiranonis	0.023	0.038	5.53.E-01	9.60.E-01
Microviridae	L7_Clostridium_hiranonis	0.063	0.038	9.85.E-02	7.78.E-01

Myoviridae	L7_Clostridium_hiranonis	-0.054	0.043	2.08.E-01	8.76.E-01
Phycodnaviridae	L7_Clostridium_hiranonis	0.026	0.038	4.99.E-01	9.55.E-01
Podoviridae	L7_Clostridium_hiranonis	-0.041	0.044	3.47.E-01	9.15.E-01
Siphoviridae	L7_Clostridium_hiranonis	0.081	0.039	3.88.E-02	7.03.E-01
Autographiviridae	L7_Bacteroides_oleiciplenus	-0.033	0.028	2.42.E-01	8.86.E-01
crAss_like_phage	L7_Bacteroides_oleiciplenus	0.034	0.031	2.86.E-01	9.15.E-01
Herelleviridae	L7_Bacteroides_oleiciplenus	-0.016	0.030	5.90.E-01	9.68.E-01
Microviridae	L7_Bacteroides_oleiciplenus	0.008	0.030	7.98.E-01	9.92.E-01
Myoviridae	L7_Bacteroides_oleiciplenus	0.072	0.033	2.92.E-02	6.66.E-01
Phycodnaviridae	L7_Bacteroides_oleiciplenus	-0.024	0.029	4.15.E-01	9.18.E-01
Podoviridae	L7_Bacteroides_oleiciplenus	-0.024	0.034	4.75.E-01	9.52.E-01
Siphoviridae	L7_Bacteroides_oleiciplenus	0.023	0.030	4.49.E-01	9.36.E-01
Autographiviridae	L7_Paraprevotella_clara	0.027	0.036	4.48.E-01	9.35.E-01
crAss_like_phage	L7_Paraprevotella_clara	-0.034	0.039	3.87.E-01	9.15.E-01
Herelleviridae	L7_Paraprevotella_clara	-0.022	0.037	5.58.E-01	9.61.E-01
Microviridae	L7_Paraprevotella_clara	-0.056	0.037	1.29.E-01	7.99.E-01
Myoviridae	L7_Paraprevotella_clara	0.090	0.041	2.89.E-02	6.66.E-01
Phycodnaviridae	L7_Paraprevotella_clara	-0.032	0.037	3.88.E-01	9.15.E-01
Podoviridae	L7_Paraprevotella_clara	0.034	0.043	4.21.E-01	9.20.E-01
Siphoviridae	L7_Paraprevotella_clara	0.066	0.038	8.10.E-02	7.47.E-01
Autographiviridae	L7_Ruminococcus_lactaris	0.018	0.042	6.74.E-01	9.80.E-01
crAss_like_phage	L7_Ruminococcus_lactaris	-0.072	0.046	1.20.E-01	7.91.E-01
Herelleviridae	L7_Ruminococcus_lactaris	-0.005	0.043	9.12.E-01	9.98.E-01
Microviridae	L7_Ruminococcus_lactaris	-0.009	0.044	8.43.E-01	9.95.E-01
Myoviridae	L7_Ruminococcus_lactaris	-0.047	0.049	3.31.E-01	9.15.E-01
Phycodnaviridae	L7_Ruminococcus_lactaris	-0.032	0.043	4.55.E-01	9.39.E-01
Podoviridae	L7_Ruminococcus_lactaris	-0.005	0.050	9.27.E-01	9.98.E-01
Siphoviridae	L7_Ruminococcus_lactaris	-0.056	0.045	2.12.E-01	8.78.E-01
Autographiviridae	L7_Bifidobacterium_boum	-0.002	0.038	9.58.E-01	9.98.E-01
crAss_like_phage	L7_Bifidobacterium_boum	0.045	0.042	2.78.E-01	9.08.E-01
Herelleviridae	L7_Bifidobacterium_boum	0.043	0.039	2.80.E-01	9.12.E-01
Microviridae	L7_Bifidobacterium_boum	-0.038	0.039	3.41.E-01	9.15.E-01
Myoviridae	L7_Bifidobacterium_boum	-0.007	0.044	8.79.E-01	9.96.E-01
Phycodnaviridae	L7_Bifidobacterium_boum	-0.007	0.039	8.63.E-01	9.96.E-01
Podoviridae	L7_Bifidobacterium_boum	0.098	0.045	3.07.E-02	6.69.E-01
Siphoviridae	L7_Bifidobacterium_boum	0.032	0.041	4.35.E-01	9.26.E-01
Autographiviridae	L7_Bacteroides_pyogenes	0.021	0.034	5.31.E-01	9.57.E-01
crAss_like_phage	L7_Bacteroides_pyogenes	0.052	0.037	1.68.E-01	8.44.E-01
Herelleviridae	L7_Bacteroides_pyogenes	0.006	0.035	8.66.E-01	9.96.E-01
Microviridae	L7_Bacteroides_pyogenes	-0.054	0.035	1.25.E-01	7.94.E-01
Myoviridae	L7_Bacteroides_pyogenes	0.044	0.039	2.62.E-01	9.00.E-01
Phycodnaviridae	L7_Bacteroides_pyogenes	-0.033	0.035	3.44.E-01	9.15.E-01
Podoviridae	L7_Bacteroides_pyogenes	-0.015	0.041	7.16.E-01	9.86.E-01
Siphoviridae	L7_Bacteroides_pyogenes	-0.014	0.036	7.00.E-01	9.81.E-01
Autographiviridae	L7_Anaerostipes_hadrus	-0.036	0.035	3.07.E-01	9.15.E-01
crAss_like_phage	L7_Anaerostipes_hadrus	-0.038	0.039	3.34.E-01	9.15.E-01
Herelleviridae	L7_Anaerostipes_hadrus	0.074	0.036	4.29.E-02	7.20.E-01
Microviridae	L7_Anaerostipes_hadrus	-0.033	0.037	3.67.E-01	9.15.E-01
Myoviridae	L7_Anaerostipes_hadrus	-0.062	0.041	1.29.E-01	7.99.E-01
Phycodnaviridae	L7_Anaerostipes_hadrus	0.041	0.036	2.58.E-01	8.95.E-01
Podoviridae	L7_Anaerostipes_hadrus	-0.022	0.042	6.11.E-01	9.75.E-01
Siphoviridae	L7_Anaerostipes_hadrus	-0.016	0.038	6.74.E-01	9.80.E-01
Autographiviridae	L7_Escherichia_fergusonii	0.016	0.031	6.10.E-01	9.75.E-01
crAss_like_phage	L7_Escherichia_fergusonii	0.025	0.035	4.81.E-01	9.53.E-01
Herelleviridae	L7_Escherichia_fergusonii	0.016	0.033	6.16.E-01	9.75.E-01
Microviridae	L7_Escherichia_fergusonii	0.016	0.033	6.17.E-01	9.75.E-01
Myoviridae	L7_Escherichia_fergusonii	0.057	0.036	1.21.E-01	7.93.E-01
Phycodnaviridae	L7_Escherichia_fergusonii	0.030	0.032	3.60.E-01	9.15.E-01
Podoviridae	L7_Escherichia_fergusonii	-0.027	0.038	4.70.E-01	9.50.E-01
Siphoviridae	L7_Escherichia_fergusonii	0.036	0.034	2.89.E-01	9.15.E-01
Autographiviridae	L7_Dorea_formicigenerans	0.019	0.035	5.97.E-01	9.69.E-01
crAss_like_phage	L7_Dorea_formicigenerans	-0.023	0.039	5.53.E-01	9.60.E-01
Herelleviridae	L7_Dorea_formicigenerans	-0.043	0.037	2.40.E-01	8.85.E-01
Microviridae	L7_Dorea_formicigenerans	-0.035	0.037	3.40.E-01	9.15.E-01
Myoviridae	L7_Dorea_formicigenerans	-0.035	0.041	3.94.E-01	9.15.E-01

Phycodnaviridae	L7_Dorea_formicigenerans	-0.030	0.036	4.03.E-01	9.16.E-01
Podoviridae	L7_Dorea_formicigenerans	0.044	0.042	2.99.E-01	9.15.E-01
Siphoviridae	L7_Dorea_formicigenerans	0.025	0.038	5.11.E-01	9.57.E-01
Autographiviridae	L7_Neglecta_timonensis	-0.038	0.032	2.36.E-01	8.85.E-01
crAss_like_phage	L7_Neglecta_timonensis	0.047	0.035	1.88.E-01	8.61.E-01
Herelleviridae	L7_Neglecta_timonensis	-0.068	0.033	4.27.E-02	7.19.E-01
Microviridae	L7_Neglecta_timonensis	0.079	0.033	1.86.E-02	5.90.E-01
Myoviridae	L7_Neglecta_timonensis	-0.014	0.037	7.00.E-01	9.81.E-01
Phycodnaviridae	L7_Neglecta_timonensis	0.005	0.033	8.89.E-01	9.96.E-01
Podoviridae	L7_Neglecta_timonensis	-0.021	0.039	5.92.E-01	9.68.E-01
Siphoviridae	L7_Neglecta_timonensis	-0.047	0.034	1.74.E-01	8.56.E-01
Autographiviridae	L7_Desulfotomaculum_sp.	0.005	0.034	8.85.E-01	9.96.E-01
crAss_like_phage	L7_Desulfotomaculum_sp.	-0.042	0.038	2.63.E-01	9.01.E-01
Herelleviridae	L7_Desulfotomaculum_sp.	-0.013	0.035	7.07.E-01	9.83.E-01
Microviridae	L7_Desulfotomaculum_sp.	0.018	0.036	6.13.E-01	9.75.E-01
Myoviridae	L7_Desulfotomaculum_sp.	0.065	0.040	1.00.E-01	7.79.E-01
Phycodnaviridae	L7_Desulfotomaculum_sp.	-0.048	0.035	1.74.E-01	8.56.E-01
Podoviridae	L7_Desulfotomaculum_sp.	0.002	0.041	9.56.E-01	9.98.E-01
Siphoviridae	L7_Desulfotomaculum_sp.	0.031	0.036	3.90.E-01	9.15.E-01
Autographiviridae	L7_Acidaminococcus_fermentans	-0.050	0.038	1.89.E-01	8.61.E-01
crAss_like_phage	L7_Acidaminococcus_fermentans	-0.088	0.042	3.80.E-02	7.02.E-01
Herelleviridae	L7_Acidaminococcus_fermentans	-0.027	0.040	5.03.E-01	9.55.E-01
Microviridae	L7_Acidaminococcus_fermentans	0.024	0.040	5.52.E-01	9.60.E-01
Myoviridae	L7_Acidaminococcus_fermentans	0.048	0.044	2.81.E-01	9.13.E-01
Phycodnaviridae	L7_Acidaminococcus_fermentans	0.032	0.039	4.13.E-01	9.18.E-01
Podoviridae	L7_Acidaminococcus_fermentans	-0.083	0.046	7.07.E-02	7.24.E-01
Siphoviridae	L7_Acidaminococcus_fermentans	0.036	0.041	3.79.E-01	9.15.E-01
Autographiviridae	L7_Coprococcus_eutactus	0.047	0.030	1.26.E-01	7.98.E-01
crAss_like_phage	L7_Coprococcus_eutactus	0.020	0.034	5.61.E-01	9.61.E-01
Herelleviridae	L7_Coprococcus_eutactus	-0.022	0.032	4.84.E-01	9.54.E-01
Microviridae	L7_Coprococcus_eutactus	0.023	0.032	4.61.E-01	9.43.E-01
Myoviridae	L7_Coprococcus_eutactus	-0.030	0.035	3.96.E-01	9.15.E-01
Phycodnaviridae	L7_Coprococcus_eutactus	-0.057	0.031	6.66.E-02	7.24.E-01
Podoviridae	L7_Coprococcus_eutactus	0.014	0.037	7.04.E-01	9.81.E-01
Siphoviridae	L7_Coprococcus_eutactus	-0.011	0.033	7.40.E-01	9.88.E-01
Autographiviridae	L7_Oscillibacter_sp.	0.006	0.027	8.18.E-01	9.93.E-01
crAss_like_phage	L7_Oscillibacter_sp.	-0.005	0.030	8.74.E-01	9.96.E-01
Herelleviridae	L7_Oscillibacter_sp.	-0.028	0.028	3.25.E-01	9.15.E-01
Microviridae	L7_Oscillibacter_sp.	-0.007	0.029	7.95.E-01	9.92.E-01
Myoviridae	L7_Oscillibacter_sp.	-0.077	0.032	1.52.E-02	5.69.E-01
Phycodnaviridae	L7_Oscillibacter_sp.	-0.001	0.028	9.74.E-01	9.99.E-01
Podoviridae	L7_Oscillibacter_sp.	0.001	0.033	9.65.E-01	9.98.E-01
Siphoviridae	L7_Oscillibacter_sp.	0.021	0.029	4.65.E-01	9.45.E-01
Autographiviridae	L7_Roseburia_faecis	0.023	0.040	5.62.E-01	9.61.E-01
crAss_like_phage	L7_Roseburia_faecis	-0.064	0.045	1.51.E-01	8.22.E-01
Herelleviridae	L7_Roseburia_faecis	-0.003	0.042	9.41.E-01	9.98.E-01
Microviridae	L7_Roseburia_faecis	-0.072	0.042	8.88.E-02	7.56.E-01
Myoviridae	L7_Roseburia_faecis	-0.069	0.047	1.41.E-01	8.10.E-01
Phycodnaviridae	L7_Roseburia_faecis	-0.096	0.041	2.04.E-02	6.01.E-01
Podoviridae	L7_Roseburia_faecis	-0.029	0.049	5.49.E-01	9.60.E-01
Siphoviridae	L7_Roseburia_faecis	-0.103	0.043	1.76.E-02	5.86.E-01
Autographiviridae	L7_Enterobacter_cloacae	-0.026	0.022	2.39.E-01	8.85.E-01
crAss_like_phage	L7_Enterobacter_cloacae	0.040	0.025	1.09.E-01	7.82.E-01
Herelleviridae	L7_Enterobacter_cloacae	-0.002	0.023	9.35.E-01	9.98.E-01
Microviridae	L7_Enterobacter_cloacae	0.028	0.023	2.37.E-01	8.85.E-01
Myoviridae	L7_Enterobacter_cloacae	-0.013	0.026	6.11.E-01	9.75.E-01
Phycodnaviridae	L7_Enterobacter_cloacae	-0.035	0.023	1.34.E-01	8.06.E-01
Podoviridae	L7_Enterobacter_cloacae	0.001	0.027	9.64.E-01	9.98.E-01
Siphoviridae	L7_Enterobacter_cloacae	0.011	0.024	6.37.E-01	9.77.E-01
Autographiviridae	L7_Streptococcus_agalactiae	0.040	0.033	2.29.E-01	8.82.E-01
crAss_like_phage	L7_Streptococcus_agalactiae	-0.033	0.037	3.68.E-01	9.15.E-01
Herelleviridae	L7_Streptococcus_agalactiae	0.031	0.035	3.72.E-01	9.15.E-01
Microviridae	L7_Streptococcus_agalactiae	-0.050	0.035	1.51.E-01	8.22.E-01
Myoviridae	L7_Streptococcus_agalactiae	-0.030	0.039	4.31.E-01	9.25.E-01
Phycodnaviridae	L7_Streptococcus_agalactiae	0.020	0.034	5.51.E-01	9.60.E-01

Podoviridae	L7_Streptococcus_agalactiae	0.014	0.040	7.23.E-01	9.86.E-01
Siphoviridae	L7_Streptococcus_agalactiae	-0.043	0.035	2.28.E-01	8.82.E-01
Autographiviridae	L7_Marvinbryantia_formatexigens	-0.008	0.038	8.27.E-01	9.93.E-01
crAss_like_phage	L7_Marvinbryantia_formatexigens	-0.023	0.043	5.85.E-01	9.65.E-01
Herelleviridae	L7_Marvinbryantia_formatexigens	-0.030	0.040	4.52.E-01	9.38.E-01
Microviridae	L7_Marvinbryantia_formatexigens	-0.078	0.040	5.02.E-02	7.22.E-01
Myoviridae	L7_Marvinbryantia_formatexigens	-0.007	0.045	8.73.E-01	9.96.E-01
Phycodnaviridae	L7_Marvinbryantia_formatexigens	0.013	0.040	7.39.E-01	9.88.E-01
Podoviridae	L7_Marvinbryantia_formatexigens	-0.072	0.046	1.17.E-01	7.86.E-01
Siphoviridae	L7_Marvinbryantia_formatexigens	-0.038	0.041	3.55.E-01	9.15.E-01
Autographiviridae	L7_Bacteroides_stercoris	0.031	0.034	3.68.E-01	9.15.E-01
crAss_like_phage	L7_Bacteroides_stercoris	0.030	0.038	4.25.E-01	9.23.E-01
Herelleviridae	L7_Bacteroides_stercoris	0.052	0.035	1.44.E-01	8.12.E-01
Microviridae	L7_Bacteroides_stercoris	0.012	0.036	7.26.E-01	9.87.E-01
Myoviridae	L7_Bacteroides_stercoris	0.049	0.040	2.12.E-01	8.78.E-01
Phycodnaviridae	L7_Bacteroides_stercoris	0.006	0.035	8.71.E-01	9.96.E-01
Podoviridae	L7_Bacteroides_stercoris	0.080	0.041	5.15.E-02	7.22.E-01
Siphoviridae	L7_Bacteroides_stercoris	0.054	0.036	1.36.E-01	8.07.E-01
Autographiviridae	L7_Aeromonas_sp.	-0.018	0.040	6.54.E-01	9.79.E-01
crAss_like_phage	L7_Aeromonas_sp.	-0.014	0.044	7.47.E-01	9.89.E-01
Herelleviridae	L7_Aeromonas_sp.	-0.081	0.041	5.02.E-02	7.22.E-01
Microviridae	L7_Aeromonas_sp.	0.030	0.042	4.69.E-01	9.50.E-01
Myoviridae	L7_Aeromonas_sp.	0.067	0.046	1.51.E-01	8.22.E-01
Phycodnaviridae	L7_Aeromonas_sp.	0.020	0.041	6.20.E-01	9.75.E-01
Podoviridae	L7_Aeromonas_sp.	-0.006	0.048	8.97.E-01	9.97.E-01
Siphoviridae	L7_Aeromonas_sp.	-0.013	0.043	7.70.E-01	9.91.E-01
Autographiviridae	L7_Azospirillum_sp.	0.034	0.040	3.92.E-01	9.15.E-01
crAss_like_phage	L7_Azospirillum_sp.	-0.082	0.044	6.09.E-02	7.24.E-01
Herelleviridae	L7_Azospirillum_sp.	-0.047	0.041	2.58.E-01	8.95.E-01
Microviridae	L7_Azospirillum_sp.	0.001	0.041	9.86.E-01	9.99.E-01
Myoviridae	L7_Azospirillum_sp.	-0.091	0.046	4.71.E-02	7.22.E-01
Phycodnaviridae	L7_Azospirillum_sp.	-0.034	0.041	3.98.E-01	9.15.E-01
Podoviridae	L7_Azospirillum_sp.	0.003	0.047	9.43.E-01	9.98.E-01
Siphoviridae	L7_Azospirillum_sp.	0.004	0.042	9.20.E-01	9.98.E-01
Autographiviridae	L7_Campylobacter_gracilis	-0.025	0.034	4.52.E-01	9.38.E-01
crAss_like_phage	L7_Campylobacter_gracilis	-0.029	0.037	4.43.E-01	9.32.E-01
Herelleviridae	L7_Campylobacter_gracilis	0.048	0.035	1.66.E-01	8.44.E-01
Microviridae	L7_Campylobacter_gracilis	-0.002	0.035	9.48.E-01	9.98.E-01
Myoviridae	L7_Campylobacter_gracilis	-0.014	0.039	7.18.E-01	9.86.E-01
Phycodnaviridae	L7_Campylobacter_gracilis	0.000	0.035	9.94.E-01	9.99.E-01
Podoviridae	L7_Campylobacter_gracilis	-0.055	0.040	1.72.E-01	8.53.E-01
Siphoviridae	L7_Campylobacter_gracilis	0.039	0.036	2.75.E-01	9.05.E-01
Autographiviridae	L7_Bacteroides_faecis	0.071	0.032	2.65.E-02	6.58.E-01
crAss_like_phage	L7_Bacteroides_faecis	0.010	0.035	7.69.E-01	9.91.E-01
Herelleviridae	L7_Bacteroides_faecis	-0.045	0.033	1.75.E-01	8.56.E-01
Microviridae	L7_Bacteroides_faecis	0.060	0.033	7.28.E-02	7.26.E-01
Myoviridae	L7_Bacteroides_faecis	-0.025	0.037	5.06.E-01	9.57.E-01
Phycodnaviridae	L7_Bacteroides_faecis	-0.020	0.033	5.35.E-01	9.59.E-01
Podoviridae	L7_Bacteroides_faecis	-0.094	0.038	1.46.E-02	5.54.E-01
Siphoviridae	L7_Bacteroides_faecis	0.028	0.034	4.12.E-01	9.18.E-01
Autographiviridae	L7_Erysipelatoclostridium_sp.	-0.018	0.039	6.39.E-01	9.78.E-01
crAss_like_phage	L7_Erysipelatoclostridium_sp.	-0.020	0.043	6.45.E-01	9.79.E-01
Herelleviridae	L7_Erysipelatoclostridium_sp.	-0.006	0.041	8.91.E-01	9.96.E-01
Microviridae	L7_Erysipelatoclostridium_sp.	-0.045	0.041	2.72.E-01	9.02.E-01
Myoviridae	L7_Erysipelatoclostridium_sp.	0.043	0.045	3.42.E-01	9.15.E-01
Phycodnaviridae	L7_Erysipelatoclostridium_sp.	0.025	0.040	5.38.E-01	9.60.E-01
Podoviridae	L7_Erysipelatoclostridium_sp.	0.020	0.047	6.77.E-01	9.80.E-01
Siphoviridae	L7_Erysipelatoclostridium_sp.	0.070	0.042	9.14.E-02	7.59.E-01
Autographiviridae	L7_Desulfovibrio_piger	0.027	0.038	4.74.E-01	9.52.E-01
crAss_like_phage	L7_Desulfovibrio_piger	-0.001	0.042	9.84.E-01	9.99.E-01
Herelleviridae	L7_Desulfovibrio_piger	0.029	0.039	4.55.E-01	9.39.E-01
Microviridae	L7_Desulfovibrio_piger	0.051	0.039	1.93.E-01	8.62.E-01
Myoviridae	L7_Desulfovibrio_piger	-0.014	0.044	7.43.E-01	9.89.E-01
Phycodnaviridae	L7_Desulfovibrio_piger	-0.062	0.039	1.09.E-01	7.82.E-01
Podoviridae	L7_Desulfovibrio_piger	-0.009	0.045	8.36.E-01	9.94.E-01

Siphoviridae	L7_Desulfovibrio_piger	-0.050	0.040	2.17.E-01	8.79.E-01
Autographiviridae	L7_Haemophilus_sputorum	0.000	0.034	9.92.E-01	9.99.E-01
crAss_like_phage	L7_Haemophilus_sputorum	-0.061	0.038	1.07.E-01	7.82.E-01
Herelleviridae	L7_Haemophilus_sputorum	0.008	0.036	8.27.E-01	9.93.E-01
Microviridae	L7_Haemophilus_sputorum	0.048	0.036	1.78.E-01	8.59.E-01
Myoviridae	L7_Haemophilus_sputorum	0.127	0.040	1.44.E-03	3.07.E-01
Phycodnaviridae	L7_Haemophilus_sputorum	0.055	0.035	1.22.E-01	7.93.E-01
Podoviridae	L7_Haemophilus_sputorum	0.002	0.041	9.59.E-01	9.98.E-01
Siphoviridae	L7_Haemophilus_sputorum	0.006	0.037	8.65.E-01	9.96.E-01
Autographiviridae	L7_Phascolarctobacterium_succinatutens	-0.041	0.042	3.23.E-01	9.15.E-01
crAss_like_phage	L7_Phascolarctobacterium_succinatutens	0.059	0.046	2.01.E-01	8.74.E-01
Herelleviridae	L7_Phascolarctobacterium_succinatutens	-0.038	0.043	3.80.E-01	9.15.E-01
Microviridae	L7_Phascolarctobacterium_succinatutens	0.026	0.044	5.47.E-01	9.60.E-01
Myoviridae	L7_Phascolarctobacterium_succinatutens	0.033	0.049	4.95.E-01	9.55.E-01
Phycodnaviridae	L7_Phascolarctobacterium_succinatutens	0.043	0.043	3.12.E-01	9.15.E-01
Podoviridae	L7_Phascolarctobacterium_succinatutens	0.023	0.050	6.47.E-01	9.79.E-01
Siphoviridae	L7_Phascolarctobacterium_succinatutens	-0.030	0.045	5.08.E-01	9.57.E-01
Autographiviridae	L7_Olsenella_uli	-0.019	0.040	6.36.E-01	9.76.E-01
crAss_like_phage	L7_Olsenella_uli	0.033	0.044	4.52.E-01	9.38.E-01
Herelleviridae	L7_Olsenella_uli	-0.016	0.041	7.03.E-01	9.81.E-01
Microviridae	L7_Olsenella_uli	-0.002	0.041	9.60.E-01	9.98.E-01
Myoviridae	L7_Olsenella_uli	0.050	0.046	2.75.E-01	9.05.E-01
Phycodnaviridae	L7_Olsenella_uli	0.003	0.041	9.47.E-01	9.98.E-01
Podoviridae	L7_Olsenella_uli	-0.031	0.048	5.08.E-01	9.57.E-01
Siphoviridae	L7_Olsenella_uli	0.037	0.042	3.85.E-01	9.15.E-01
Autographiviridae	L7_Dorea_sp.	0.027	0.037	4.52.E-01	9.38.E-01
crAss_like_phage	L7_Dorea_sp.	0.032	0.040	4.27.E-01	9.25.E-01
Herelleviridae	L7_Dorea_sp.	-0.026	0.038	4.88.E-01	9.55.E-01
Microviridae	L7_Dorea_sp.	-0.038	0.038	3.20.E-01	9.15.E-01
Myoviridae	L7_Dorea_sp.	-0.064	0.042	1.30.E-01	7.99.E-01
Phycodnaviridae	L7_Dorea_sp.	-0.012	0.038	7.44.E-01	9.89.E-01
Podoviridae	L7_Dorea_sp.	-0.006	0.044	8.94.E-01	9.97.E-01
Siphoviridae	L7_Dorea_sp.	-0.022	0.039	5.77.E-01	9.63.E-01
Autographiviridae	L7_Eggerthella_lenta	0.032	0.033	3.31.E-01	9.15.E-01
crAss_like_phage	L7_Eggerthella_lenta	-0.032	0.036	3.81.E-01	9.15.E-01
Herelleviridae	L7_Eggerthella_lenta	0.001	0.034	9.73.E-01	9.99.E-01
Microviridae	L7_Eggerthella_lenta	-0.066	0.034	5.24.E-02	7.22.E-01
Myoviridae	L7_Eggerthella_lenta	-0.045	0.038	2.33.E-01	8.84.E-01
Phycodnaviridae	L7_Eggerthella_lenta	-0.007	0.034	8.37.E-01	9.94.E-01
Podoviridae	L7_Eggerthella_lenta	0.042	0.039	2.80.E-01	9.11.E-01
Siphoviridae	L7_Eggerthella_lenta	-0.086	0.035	1.39.E-02	5.51.E-01
Autographiviridae	L7_Clostridium_paraputrificum	0.014	0.040	7.25.E-01	9.87.E-01
crAss_like_phage	L7_Clostridium_paraputrificum	0.030	0.044	4.92.E-01	9.55.E-01
Herelleviridae	L7_Clostridium_paraputrificum	-0.045	0.041	2.75.E-01	9.05.E-01
Microviridae	L7_Clostridium_paraputrificum	-0.042	0.041	3.16.E-01	9.15.E-01
Myoviridae	L7_Clostridium_paraputrificum	-0.031	0.046	4.96.E-01	9.55.E-01
Phycodnaviridae	L7_Clostridium_paraputrificum	0.006	0.041	8.79.E-01	9.96.E-01
Podoviridae	L7_Clostridium_paraputrificum	-0.083	0.048	8.27.E-02	7.49.E-01
Siphoviridae	L7_Clostridium_paraputrificum	-0.008	0.043	8.60.E-01	9.96.E-01
Autographiviridae	L7_Parabacteroides_gordonii	0.010	0.034	7.56.E-01	9.89.E-01
crAss_like_phage	L7_Parabacteroides_gordonii	-0.008	0.037	8.19.E-01	9.93.E-01
Herelleviridae	L7_Parabacteroides_gordonii	0.000	0.035	9.90.E-01	9.99.E-01
Microviridae	L7_Parabacteroides_gordonii	0.002	0.035	9.47.E-01	9.98.E-01
Myoviridae	L7_Parabacteroides_gordonii	-0.031	0.039	4.29.E-01	9.25.E-01
Phycodnaviridae	L7_Parabacteroides_gordonii	0.062	0.034	7.09.E-02	7.24.E-01
Podoviridae	L7_Parabacteroides_gordonii	-0.012	0.040	7.65.E-01	9.91.E-01
Siphoviridae	L7_Parabacteroides_gordonii	0.018	0.036	6.14.E-01	9.75.E-01
Autographiviridae	L7_Scardovia_wiggsiae	-0.029	0.041	4.83.E-01	9.54.E-01
crAss_like_phage	L7_Scardovia_wiggsiae	-0.039	0.046	3.99.E-01	9.15.E-01
Herelleviridae	L7_Scardovia_wiggsiae	0.046	0.043	2.81.E-01	9.13.E-01
Microviridae	L7_Scardovia_wiggsiae	0.008	0.043	8.47.E-01	9.95.E-01
Myoviridae	L7_Scardovia_wiggsiae	-0.063	0.048	1.89.E-01	8.61.E-01
Phycodnaviridae	L7_Scardovia_wiggsiae	0.011	0.043	7.89.E-01	9.92.E-01
Podoviridae	L7_Scardovia_wiggsiae	-0.118	0.049	1.75.E-02	5.86.E-01
Siphoviridae	L7_Scardovia_wiggsiae	0.007	0.044	8.70.E-01	9.96.E-01

Autographiviridae	L7_Peptostreptococcaceae_bacterium	0.025	0.028	3.89.E-01	9.15.E-01
crAss_like_phage	L7_Peptostreptococcaceae_bacterium	0.005	0.032	8.66.E-01	9.96.E-01
Herelleviridae	L7_Peptostreptococcaceae_bacterium	0.047	0.030	1.15.E-01	7.85.E-01
Microviridae	L7_Peptostreptococcaceae_bacterium	-0.051	0.030	8.49.E-02	7.50.E-01
Myoviridae	L7_Peptostreptococcaceae_bacterium	-0.039	0.033	2.35.E-01	8.85.E-01
Phycodnaviridae	L7_Peptostreptococcaceae_bacterium	0.005	0.029	8.74.E-01	9.96.E-01
Podoviridae	L7_Peptostreptococcaceae_bacterium	0.053	0.034	1.24.E-01	7.94.E-01
Siphoviridae	L7_Peptostreptococcaceae_bacterium	0.019	0.030	5.30.E-01	9.57.E-01
Autographiviridae	L7_Bifidobacterium_merycicum	0.027	0.032	4.03.E-01	9.16.E-01
crAss_like_phage	L7_Bifidobacterium_merycicum	-0.065	0.035	6.63.E-02	7.24.E-01
Herelleviridae	L7_Bifidobacterium_merycicum	0.006	0.033	8.53.E-01	9.95.E-01
Microviridae	L7_Bifidobacterium_merycicum	0.010	0.033	7.73.E-01	9.92.E-01
Myoviridae	L7_Bifidobacterium_merycicum	-0.007	0.037	8.51.E-01	9.95.E-01
Phycodnaviridae	L7_Bifidobacterium_merycicum	0.015	0.033	6.52.E-01	9.79.E-01
Podoviridae	L7_Bifidobacterium_merycicum	0.008	0.038	8.30.E-01	9.94.E-01
Siphoviridae	L7_Bifidobacterium_merycicum	0.028	0.034	4.09.E-01	9.17.E-01
Autographiviridae	L7_Gemella_haemolysans	-0.038	0.030	2.11.E-01	8.78.E-01
crAss_like_phage	L7_Gemella_haemolysans	0.050	0.034	1.38.E-01	8.10.E-01
Herelleviridae	L7_Gemella_haemolysans	-0.057	0.032	7.35.E-02	7.26.E-01
Microviridae	L7_Gemella_haemolysans	-0.011	0.032	7.39.E-01	9.88.E-01
Myoviridae	L7_Gemella_haemolysans	-0.008	0.035	8.14.E-01	9.93.E-01
Phycodnaviridae	L7_Gemella_haemolysans	-0.057	0.031	6.64.E-02	7.24.E-01
Podoviridae	L7_Gemella_haemolysans	0.024	0.037	5.10.E-01	9.57.E-01
Siphoviridae	L7_Gemella_haemolysans	0.003	0.033	9.18.E-01	9.98.E-01
Autographiviridae	L7_Enterococcus_raffinosus	0.057	0.034	1.01.E-01	7.79.E-01
crAss_like_phage	L7_Enterococcus_raffinosus	-0.059	0.038	1.22.E-01	7.93.E-01
Herelleviridae	L7_Enterococcus_raffinosus	-0.030	0.036	4.09.E-01	9.17.E-01
Microviridae	L7_Enterococcus_raffinosus	-0.053	0.036	1.37.E-01	8.10.E-01
Myoviridae	L7_Enterococcus_raffinosus	0.023	0.040	5.66.E-01	9.61.E-01
Phycodnaviridae	L7_Enterococcus_raffinosus	-0.026	0.035	4.61.E-01	9.43.E-01
Podoviridae	L7_Enterococcus_raffinosus	0.023	0.041	5.79.E-01	9.64.E-01
Siphoviridae	L7_Enterococcus_raffinosus	-0.020	0.037	5.92.E-01	9.68.E-01
Autographiviridae	L7_Bacteroides_barnesiae	0.011	0.030	7.12.E-01	9.85.E-01
crAss_like_phage	L7_Bacteroides_barnesiae	-0.028	0.033	4.02.E-01	9.16.E-01
Herelleviridae	L7_Bacteroides_barnesiae	0.034	0.031	2.82.E-01	9.13.E-01
Microviridae	L7_Bacteroides_barnesiae	0.011	0.031	7.36.E-01	9.88.E-01
Myoviridae	L7_Bacteroides_barnesiae	0.041	0.035	2.42.E-01	8.85.E-01
Phycodnaviridae	L7_Bacteroides_barnesiae	-0.006	0.031	8.42.E-01	9.95.E-01
Podoviridae	L7_Bacteroides_barnesiae	0.024	0.036	5.05.E-01	9.57.E-01
Siphoviridae	L7_Bacteroides_barnesiae	-0.022	0.032	4.89.E-01	9.55.E-01
Autographiviridae	L7_Pseudoflavonifractor_sp.	-0.018	0.029	5.37.E-01	9.60.E-01
crAss_like_phage	L7_Pseudoflavonifractor_sp.	-0.059	0.032	7.09.E-02	7.24.E-01
Herelleviridae	L7_Pseudoflavonifractor_sp.	0.015	0.031	6.19.E-01	9.75.E-01
Microviridae	L7_Pseudoflavonifractor_sp.	-0.011	0.031	7.13.E-01	9.85.E-01
Myoviridae	L7_Pseudoflavonifractor_sp.	-0.003	0.034	9.26.E-01	9.98.E-01
Phycodnaviridae	L7_Pseudoflavonifractor_sp.	-0.064	0.030	3.32.E-02	6.82.E-01
Podoviridae	L7_Pseudoflavonifractor_sp.	-0.023	0.035	5.09.E-01	9.57.E-01
Siphoviridae	L7_Pseudoflavonifractor_sp.	-0.039	0.031	2.13.E-01	8.78.E-01
Autographiviridae	L7_Streptococcus_thermophilus	-0.024	0.042	5.68.E-01	9.61.E-01
crAss_like_phage	L7_Streptococcus_thermophilus	0.029	0.047	5.34.E-01	9.59.E-01
Herelleviridae	L7_Streptococcus_thermophilus	0.057	0.044	1.96.E-01	8.66.E-01
Microviridae	L7_Streptococcus_thermophilus	0.040	0.044	3.65.E-01	9.15.E-01
Myoviridae	L7_Streptococcus_thermophilus	0.122	0.049	1.25.E-02	5.43.E-01
Phycodnaviridae	L7_Streptococcus_thermophilus	0.009	0.043	8.43.E-01	9.95.E-01
Podoviridae	L7_Streptococcus_thermophilus	0.099	0.050	4.98.E-02	7.22.E-01
Siphoviridae	L7_Streptococcus_thermophilus	0.078	0.045	8.49.E-02	7.50.E-01
Autographiviridae	L7_Veillonella_parvula	-0.008	0.032	8.01.E-01	9.92.E-01
crAss_like_phage	L7_Veillonella_parvula	-0.079	0.035	2.27.E-02	6.19.E-01
Herelleviridae	L7_Veillonella_parvula	-0.035	0.033	2.79.E-01	9.10.E-01
Microviridae	L7_Veillonella_parvula	0.004	0.033	9.10.E-01	9.98.E-01
Myoviridae	L7_Veillonella_parvula	-0.008	0.037	8.20.E-01	9.93.E-01
Phycodnaviridae	L7_Veillonella_parvula	0.022	0.032	4.98.E-01	9.55.E-01
Podoviridae	L7_Veillonella_parvula	-0.075	0.038	4.70.E-02	7.22.E-01
Siphoviridae	L7_Veillonella_parvula	-0.045	0.034	1.85.E-01	8.61.E-01
Autographiviridae	L7_Eubacterium_siraeum	-0.025	0.033	4.55.E-01	9.39.E-01

crAss_like_phage	L7_Eubacterium_siraeum	0.058	0.037	1.17.E-01	7.86.E-01
Herelleviridae	L7_Eubacterium_siraeum	0.068	0.035	4.94.E-02	7.22.E-01
Microviridae	L7_Eubacterium_siraeum	0.012	0.035	7.30.E-01	9.88.E-01
Myoviridae	L7_Eubacterium_siraeum	0.055	0.039	1.55.E-01	8.26.E-01
Phycodnaviridae	L7_Eubacterium_siraeum	0.022	0.034	5.23.E-01	9.57.E-01
Podoviridae	L7_Eubacterium_siraeum	0.031	0.040	4.37.E-01	9.27.E-01
Siphoviridae	L7_Eubacterium_siraeum	-0.018	0.036	6.21.E-01	9.76.E-01
Autographiviridae	L7_Lachnoclostridium_sp.	0.017	0.034	6.15.E-01	9.75.E-01
crAss_like_phage	L7_Lachnoclostridium_sp.	0.028	0.037	4.48.E-01	9.36.E-01
Herelleviridae	L7_Lachnoclostridium_sp.	0.055	0.035	1.13.E-01	7.84.E-01
Microviridae	L7_Lachnoclostridium_sp.	0.030	0.035	3.97.E-01	9.15.E-01
Myoviridae	L7_Lachnoclostridium_sp.	-0.038	0.039	3.33.E-01	9.15.E-01
Phycodnaviridae	L7_Lachnoclostridium_sp.	0.071	0.034	3.95.E-02	7.03.E-01
Podoviridae	L7_Lachnoclostridium_sp.	0.020	0.040	6.14.E-01	9.75.E-01
Siphoviridae	L7_Lachnoclostridium_sp.	0.052	0.036	1.47.E-01	8.17.E-01
Autographiviridae	L7_Streptococcus_gallolyticus	-0.022	0.039	5.72.E-01	9.61.E-01
crAss_like_phage	L7_Streptococcus_gallolyticus	0.027	0.044	5.39.E-01	9.60.E-01
Herelleviridae	L7_Streptococcus_gallolyticus	0.029	0.041	4.71.E-01	9.51.E-01
Microviridae	L7_Streptococcus_gallolyticus	-0.003	0.041	9.43.E-01	9.98.E-01
Myoviridae	L7_Streptococcus_gallolyticus	-0.054	0.046	2.34.E-01	8.85.E-01
Phycodnaviridae	L7_Streptococcus_gallolyticus	-0.050	0.040	2.20.E-01	8.79.E-01
Podoviridae	L7_Streptococcus_gallolyticus	-0.022	0.047	6.45.E-01	9.79.E-01
Siphoviridae	L7_Streptococcus_gallolyticus	-0.005	0.042	9.07.E-01	9.97.E-01
Autographiviridae	L7_Bacillus_subtilis	0.021	0.044	6.35.E-01	9.76.E-01
crAss_like_phage	L7_Bacillus_subtilis	0.068	0.049	1.61.E-01	8.38.E-01
Herelleviridae	L7_Bacillus_subtilis	-0.042	0.046	3.63.E-01	9.15.E-01
Microviridae	L7_Bacillus_subtilis	0.055	0.046	2.32.E-01	8.82.E-01
Myoviridae	L7_Bacillus_subtilis	0.087	0.051	9.08.E-02	7.59.E-01
Phycodnaviridae	L7_Bacillus_subtilis	0.084	0.045	6.36.E-02	7.24.E-01
Podoviridae	L7_Bacillus_subtilis	0.055	0.053	3.04.E-01	9.15.E-01
Siphoviridae	L7_Bacillus_subtilis	0.080	0.047	9.14.E-02	7.59.E-01
Autographiviridae	L7_Parabacteroides_goldsteinii	0.007	0.033	8.32.E-01	9.94.E-01
crAss_like_phage	L7_Parabacteroides_goldsteinii	-0.028	0.037	4.44.E-01	9.33.E-01
Herelleviridae	L7_Parabacteroides_goldsteinii	0.006	0.035	8.68.E-01	9.96.E-01
Microviridae	L7_Parabacteroides_goldsteinii	-0.058	0.035	9.31.E-02	7.60.E-01
Myoviridae	L7_Parabacteroides_goldsteinii	0.018	0.039	6.43.E-01	9.79.E-01
Phycodnaviridae	L7_Parabacteroides_goldsteinii	0.006	0.034	8.62.E-01	9.96.E-01
Podoviridae	L7_Parabacteroides_goldsteinii	-0.019	0.040	6.44.E-01	9.79.E-01
Siphoviridae	L7_Parabacteroides_goldsteinii	-0.034	0.036	3.48.E-01	9.15.E-01
Autographiviridae	L7_Lachnoanaerobaculum_sp.	-0.024	0.040	5.45.E-01	9.60.E-01
crAss_like_phage	L7_Lachnoanaerobaculum_sp.	0.055	0.044	2.07.E-01	8.76.E-01
Herelleviridae	L7_Lachnoanaerobaculum_sp.	0.029	0.041	4.84.E-01	9.54.E-01
Microviridae	L7_Lachnoanaerobaculum_sp.	0.107	0.041	9.60.E-03	4.94.E-01
Myoviridae	L7_Lachnoanaerobaculum_sp.	0.112	0.046	1.42.E-02	5.51.E-01
Phycodnaviridae	L7_Lachnoanaerobaculum_sp.	-0.014	0.041	7.39.E-01	9.88.E-01
Podoviridae	L7_Lachnoanaerobaculum_sp.	0.117	0.047	1.33.E-02	5.51.E-01
Siphoviridae	L7_Lachnoanaerobaculum_sp.	0.103	0.042	1.47.E-02	5.56.E-01
Autographiviridae	L7_Ruminococcus_torques	0.054	0.038	1.55.E-01	8.26.E-01
crAss_like_phage	L7_Ruminococcus_torques	0.026	0.042	5.32.E-01	9.57.E-01
Herelleviridae	L7_Ruminococcus_torques	-0.055	0.039	1.59.E-01	8.38.E-01
Microviridae	L7_Ruminococcus_torques	-0.044	0.039	2.61.E-01	8.99.E-01
Myoviridae	L7_Ruminococcus_torques	-0.095	0.044	2.95.E-02	6.66.E-01
Phycodnaviridae	L7_Ruminococcus_torques	-0.040	0.039	3.07.E-01	9.15.E-01
Podoviridae	L7_Ruminococcus_torques	0.047	0.045	3.04.E-01	9.15.E-01
Siphoviridae	L7_Ruminococcus_torques	0.014	0.040	7.26.E-01	9.87.E-01
Autographiviridae	L7_Alistipes_ihumii	0.000	0.033	9.94.E-01	9.99.E-01
crAss_like_phage	L7_Alistipes_ihumii	0.042	0.036	2.45.E-01	8.90.E-01
Herelleviridae	L7_Alistipes_ihumii	0.002	0.034	9.56.E-01	9.98.E-01
Microviridae	L7_Alistipes_ihumii	-0.016	0.034	6.34.E-01	9.76.E-01
Myoviridae	L7_Alistipes_ihumii	0.040	0.038	2.92.E-01	9.15.E-01
Phycodnaviridae	L7_Alistipes_ihumii	-0.031	0.034	3.56.E-01	9.15.E-01
Podoviridae	L7_Alistipes_ihumii	-0.031	0.039	4.31.E-01	9.25.E-01
Siphoviridae	L7_Alistipes_ihumii	-0.038	0.035	2.83.E-01	9.13.E-01
Autographiviridae	L7_Ruminococcus_flavefaciens	0.029	0.030	3.33.E-01	9.15.E-01
crAss_like_phage	L7_Ruminococcus_flavefaciens	0.027	0.033	4.14.E-01	9.18.E-01

Herelleviridae	L7_Ruminococcus_flavefaciens	-0.014	0.031	6.53.E-01	9.79.E-01
Microviridae	L7_Ruminococcus_flavefaciens	0.044	0.031	1.62.E-01	8.38.E-01
Myoviridae	L7_Ruminococcus_flavefaciens	0.035	0.035	3.22.E-01	9.15.E-01
Phycodnaviridae	L7_Ruminococcus_flavefaciens	0.067	0.031	3.18.E-02	6.75.E-01
Podoviridae	L7_Ruminococcus_flavefaciens	-0.022	0.036	5.52.E-01	9.60.E-01
Siphoviridae	L7_Ruminococcus_flavefaciens	0.077	0.032	1.72.E-02	5.86.E-01
Autographiviridae	L7_Bacillus_cereus	0.031	0.040	4.47.E-01	9.34.E-01
crAss_like_phage	L7_Bacillus_cereus	0.012	0.045	7.93.E-01	9.92.E-01
Herelleviridae	L7_Bacillus_cereus	0.014	0.042	7.41.E-01	9.89.E-01
Microviridae	L7_Bacillus_cereus	0.021	0.042	6.20.E-01	9.75.E-01
Myoviridae	L7_Bacillus_cereus	-0.010	0.047	8.36.E-01	9.94.E-01
Phycodnaviridae	L7_Bacillus_cereus	0.007	0.041	8.64.E-01	9.96.E-01
Podoviridae	L7_Bacillus_cereus	0.031	0.048	5.28.E-01	9.57.E-01
Siphoviridae	L7_Bacillus_cereus	0.030	0.043	4.88.E-01	9.54.E-01
Autographiviridae	L7_Bifidobacterium_adolescentis	0.045	0.030	1.41.E-01	8.10.E-01
crAss_like_phage	L7_Bifidobacterium_adolescentis	-0.026	0.034	4.47.E-01	9.34.E-01
Herelleviridae	L7_Bifidobacterium_adolescentis	0.022	0.032	4.82.E-01	9.53.E-01
Microviridae	L7_Bifidobacterium_adolescentis	0.005	0.032	8.66.E-01	9.96.E-01
Myoviridae	L7_Bifidobacterium_adolescentis	-0.053	0.035	1.31.E-01	8.00.E-01
Phycodnaviridae	L7_Bifidobacterium_adolescentis	0.008	0.031	8.00.E-01	9.92.E-01
Podoviridae	L7_Bifidobacterium_adolescentis	0.008	0.036	8.23.E-01	9.93.E-01
Siphoviridae	L7_Bifidobacterium_adolescentis	0.030	0.032	3.53.E-01	9.15.E-01
Autographiviridae	L7_Enterobacter_ludwigii	0.019	0.035	5.99.E-01	9.70.E-01
crAss_like_phage	L7_Enterobacter_ludwigii	0.048	0.039	2.25.E-01	8.82.E-01
Herelleviridae	L7_Enterobacter_ludwigii	0.006	0.037	8.66.E-01	9.96.E-01
Microviridae	L7_Enterobacter_ludwigii	0.030	0.037	4.15.E-01	9.18.E-01
Myoviridae	L7_Enterobacter_ludwigii	-0.051	0.041	2.13.E-01	8.78.E-01
Phycodnaviridae	L7_Enterobacter_ludwigii	-0.055	0.036	1.34.E-01	8.06.E-01
Podoviridae	L7_Enterobacter_ludwigii	-0.002	0.043	9.66.E-01	9.98.E-01
Siphoviridae	L7_Enterobacter_ludwigii	-0.003	0.038	9.36.E-01	9.98.E-01
Autographiviridae	L7_Actinomyces_odontolyticus	-0.016	0.033	6.24.E-01	9.76.E-01
crAss_like_phage	L7_Actinomyces_odontolyticus	-0.022	0.036	5.36.E-01	9.60.E-01
Herelleviridae	L7_Actinomyces_odontolyticus	-0.042	0.034	2.12.E-01	8.78.E-01
Microviridae	L7_Actinomyces_odontolyticus	-0.034	0.034	3.18.E-01	9.15.E-01
Myoviridae	L7_Actinomyces_odontolyticus	-0.081	0.038	3.09.E-02	6.70.E-01
Phycodnaviridae	L7_Actinomyces_odontolyticus	-0.043	0.033	2.03.E-01	8.75.E-01
Podoviridae	L7_Actinomyces_odontolyticus	-0.034	0.039	3.86.E-01	9.15.E-01
Siphoviridae	L7_Actinomyces_odontolyticus	0.019	0.035	5.88.E-01	9.67.E-01
Autographiviridae	L7_Bacteroides_acidifaciens	-0.006	0.029	8.37.E-01	9.94.E-01
crAss_like_phage	L7_Bacteroides_acidifaciens	0.001	0.032	9.86.E-01	9.99.E-01
Herelleviridae	L7_Bacteroides_acidifaciens	0.026	0.030	3.94.E-01	9.15.E-01
Microviridae	L7_Bacteroides_acidifaciens	-0.083	0.030	5.48.E-03	4.29.E-01
Myoviridae	L7_Bacteroides_acidifaciens	0.011	0.033	7.52.E-01	9.89.E-01
Phycodnaviridae	L7_Bacteroides_acidifaciens	-0.006	0.030	8.41.E-01	9.95.E-01
Podoviridae	L7_Bacteroides_acidifaciens	0.039	0.034	2.59.E-01	8.95.E-01
Siphoviridae	L7_Bacteroides_acidifaciens	0.019	0.031	5.45.E-01	9.60.E-01
Autographiviridae	L7_Prevotella_copri	0.000	0.035	9.97.E-01	9.99.E-01
crAss_like_phage	L7_Prevotella_copri	0.030	0.039	4.42.E-01	9.32.E-01
Herelleviridae	L7_Prevotella_copri	-0.025	0.036	4.99.E-01	9.55.E-01
Microviridae	L7_Prevotella_copri	0.010	0.037	7.84.E-01	9.92.E-01
Myoviridae	L7_Prevotella_copri	-0.040	0.041	3.25.E-01	9.15.E-01
Phycodnaviridae	L7_Prevotella_copri	-0.065	0.036	6.90.E-02	7.24.E-01
Podoviridae	L7_Prevotella_copri	0.035	0.042	4.11.E-01	9.18.E-01
Siphoviridae	L7_Prevotella_copri	0.022	0.038	5.53.E-01	9.60.E-01
Autographiviridae	L7_Collinsella_intestinalis	-0.005	0.039	8.99.E-01	9.97.E-01
crAss_like_phage	L7_Collinsella_intestinalis	-0.056	0.043	1.88.E-01	8.61.E-01
Herelleviridae	L7_Collinsella_intestinalis	0.014	0.040	7.23.E-01	9.86.E-01
Microviridae	L7_Collinsella_intestinalis	-0.055	0.040	1.75.E-01	8.56.E-01
Myoviridae	L7_Collinsella_intestinalis	-0.023	0.045	6.14.E-01	9.75.E-01
Phycodnaviridae	L7_Collinsella_intestinalis	0.033	0.040	4.02.E-01	9.15.E-01
Podoviridae	L7_Collinsella_intestinalis	-0.027	0.046	5.63.E-01	9.61.E-01
Siphoviridae	L7_Collinsella_intestinalis	-0.038	0.041	3.63.E-01	9.15.E-01
Autographiviridae	L7_Bifidobacterium_animalis	0.016	0.045	7.18.E-01	9.86.E-01
crAss_like_phage	L7_Bifidobacterium_animalis	-0.067	0.049	1.75.E-01	8.56.E-01
Herelleviridae	L7_Bifidobacterium_animalis	0.010	0.046	8.34.E-01	9.94.E-01

Microviridae	L7_Bifidobacterium_animalis	-0.070	0.046	1.33.E-01	8.05.E-01
Myoviridae	L7_Bifidobacterium_animalis	0.040	0.052	4.38.E-01	9.29.E-01
Phycodnaviridae	L7_Bifidobacterium_animalis	0.057	0.046	2.16.E-01	8.79.E-01
Podoviridae	L7_Bifidobacterium_animalis	0.036	0.054	5.03.E-01	9.55.E-01
Siphoviridae	L7_Bifidobacterium_animalis	0.027	0.048	5.71.E-01	9.61.E-01
Autographiviridae	L7_Megamonas_funiformis	-0.010	0.038	7.83.E-01	9.92.E-01
crAss_like_phage	L7_Megamonas_funiformis	-0.002	0.042	9.68.E-01	9.98.E-01
Herelleviridae	L7_Megamonas_funiformis	-0.012	0.039	7.67.E-01	9.91.E-01
Microviridae	L7_Megamonas_funiformis	-0.034	0.039	3.82.E-01	9.15.E-01
Myoviridae	L7_Megamonas_funiformis	0.055	0.044	2.07.E-01	8.76.E-01
Phycodnaviridae	L7_Megamonas_funiformis	-0.037	0.039	3.36.E-01	9.15.E-01
Podoviridae	L7_Megamonas_funiformis	-0.044	0.045	3.33.E-01	9.15.E-01
Siphoviridae	L7_Megamonas_funiformis	-0.034	0.040	3.94.E-01	9.15.E-01
Autographiviridae	L7_Weissella_cibaria	0.017	0.042	6.78.E-01	9.80.E-01
crAss_like_phage	L7_Weissella_cibaria	-0.020	0.046	6.62.E-01	9.79.E-01
Herelleviridae	L7_Weissella_cibaria	0.048	0.043	2.65.E-01	9.01.E-01
Microviridae	L7_Weissella_cibaria	0.025	0.044	5.69.E-01	9.61.E-01
Myoviridae	L7_Weissella_cibaria	-0.094	0.048	5.11.E-02	7.22.E-01
Phycodnaviridae	L7_Weissella_cibaria	-0.019	0.043	6.62.E-01	9.79.E-01
Podoviridae	L7_Weissella_cibaria	-0.053	0.050	2.87.E-01	9.15.E-01
Siphoviridae	L7_Weissella_cibaria	-0.023	0.045	6.10.E-01	9.75.E-01
Autographiviridae	L7_Acinetobacter_baumannii	-0.049	0.043	2.48.E-01	8.94.E-01
crAss_like_phage	L7_Acinetobacter_baumannii	-0.019	0.047	6.94.E-01	9.81.E-01
Herelleviridae	L7_Acinetobacter_baumannii	-0.040	0.044	3.63.E-01	9.15.E-01
Microviridae	L7_Acinetobacter_baumannii	-0.053	0.044	2.29.E-01	8.82.E-01
Myoviridae	L7_Acinetobacter_baumannii	0.113	0.049	2.25.E-02	6.19.E-01
Phycodnaviridae	L7_Acinetobacter_baumannii	0.043	0.044	3.24.E-01	9.15.E-01
Podoviridae	L7_Acinetobacter_baumannii	0.063	0.051	2.20.E-01	8.79.E-01
Siphoviridae	L7_Acinetobacter_baumannii	0.045	0.046	3.22.E-01	9.15.E-01
Autographiviridae	L7_Subdoligranulum_sp.	-0.021	0.028	4.47.E-01	9.34.E-01
crAss_like_phage	L7_Subdoligranulum_sp.	0.026	0.031	3.96.E-01	9.15.E-01
Herelleviridae	L7_Subdoligranulum_sp.	-0.014	0.029	6.42.E-01	9.79.E-01
Microviridae	L7_Subdoligranulum_sp.	-0.044	0.029	1.28.E-01	7.99.E-01
Myoviridae	L7_Subdoligranulum_sp.	-0.042	0.033	1.92.E-01	8.61.E-01
Phycodnaviridae	L7_Subdoligranulum_sp.	-0.051	0.029	7.47.E-02	7.29.E-01
Podoviridae	L7_Subdoligranulum_sp.	0.045	0.034	1.79.E-01	8.60.E-01
Siphoviridae	L7_Subdoligranulum_sp.	-0.010	0.030	7.36.E-01	9.88.E-01
Autographiviridae	L7_Paraprevotella_xylaniphila	-0.007	0.036	8.47.E-01	9.95.E-01
crAss_like_phage	L7_Paraprevotella_xylaniphila	-0.035	0.040	3.82.E-01	9.15.E-01
Herelleviridae	L7_Paraprevotella_xylaniphila	-0.021	0.037	5.73.E-01	9.61.E-01
Microviridae	L7_Paraprevotella_xylaniphila	-0.005	0.038	8.92.E-01	9.96.E-01
Myoviridae	L7_Paraprevotella_xylaniphila	-0.020	0.042	6.41.E-01	9.79.E-01
Phycodnaviridae	L7_Paraprevotella_xylaniphila	0.001	0.037	9.86.E-01	9.99.E-01
Podoviridae	L7_Paraprevotella_xylaniphila	0.024	0.043	5.86.E-01	9.66.E-01
Siphoviridae	L7_Paraprevotella_xylaniphila	0.030	0.039	4.36.E-01	9.26.E-01
Autographiviridae	L7_Dialister_invisus	0.028	0.043	5.17.E-01	9.57.E-01
crAss_like_phage	L7_Dialister_invisus	0.002	0.047	9.64.E-01	9.98.E-01
Herelleviridae	L7_Dialister_invisus	-0.055	0.045	2.21.E-01	8.79.E-01
Microviridae	L7_Dialister_invisus	-0.040	0.045	3.66.E-01	9.15.E-01
Myoviridae	L7_Dialister_invisus	-0.016	0.050	7.42.E-01	9.89.E-01
Phycodnaviridae	L7_Dialister_invisus	-0.011	0.044	8.12.E-01	9.93.E-01
Podoviridae	L7_Dialister_invisus	0.045	0.051	3.77.E-01	9.15.E-01
Siphoviridae	L7_Dialister_invisus	0.027	0.046	5.56.E-01	9.61.E-01
Autographiviridae	L7_Dialister_succinatiphilus	-0.012	0.043	7.86.E-01	9.92.E-01
crAss_like_phage	L7_Dialister_succinatiphilus	-0.054	0.048	2.54.E-01	8.95.E-01
Herelleviridae	L7_Dialister_succinatiphilus	-0.091	0.045	4.17.E-02	7.14.E-01
Microviridae	L7_Dialister_succinatiphilus	-0.030	0.045	5.07.E-01	9.57.E-01
Myoviridae	L7_Dialister_succinatiphilus	-0.055	0.050	2.69.E-01	9.02.E-01
Phycodnaviridae	L7_Dialister_succinatiphilus	-0.043	0.044	3.33.E-01	9.15.E-01
Podoviridae	L7_Dialister_succinatiphilus	-0.063	0.052	2.26.E-01	8.82.E-01
Siphoviridae	L7_Dialister_succinatiphilus	-0.011	0.046	8.13.E-01	9.93.E-01
Autographiviridae	L7_Burkholderiales_bacterium	-0.005	0.038	8.91.E-01	9.96.E-01
crAss_like_phage	L7_Burkholderiales_bacterium	0.014	0.042	7.37.E-01	9.88.E-01
Herelleviridae	L7_Burkholderiales_bacterium	0.074	0.039	5.76.E-02	7.24.E-01
Microviridae	L7_Burkholderiales_bacterium	-0.045	0.039	2.49.E-01	8.94.E-01

Myoviridae	L7_Burkholderiales_bacterium	0.028	0.044	5.26.E-01	9.57.E-01
Phycodnaviridae	L7_Burkholderiales_bacterium	0.015	0.039	6.97.E-01	9.81.E-01
Podoviridae	L7_Burkholderiales_bacterium	-0.056	0.045	2.18.E-01	8.79.E-01
Siphoviridae	L7_Burkholderiales_bacterium	-0.026	0.040	5.18.E-01	9.57.E-01
Autographiviridae	L7_Odoribacter_splanchnicus	-0.028	0.023	2.24.E-01	8.82.E-01
crAss_like_phage	L7_Odoribacter_splanchnicus	0.042	0.026	1.06.E-01	7.82.E-01
Herelleviridae	L7_Odoribacter_splanchnicus	0.012	0.024	6.14.E-01	9.75.E-01
Microviridae	L7_Odoribacter_splanchnicus	0.016	0.024	5.00.E-01	9.55.E-01
Myoviridae	L7_Odoribacter_splanchnicus	-0.017	0.027	5.43.E-01	9.60.E-01
Phycodnaviridae	L7_Odoribacter_splanchnicus	0.046	0.024	5.53.E-02	7.24.E-01
Podoviridae	L7_Odoribacter_splanchnicus	0.031	0.028	2.69.E-01	9.01.E-01
Siphoviridae	L7_Odoribacter_splanchnicus	0.016	0.025	5.14.E-01	9.57.E-01
Autographiviridae	L7_Streptococcus_macedonicus	0.017	0.039	6.59.E-01	9.79.E-01
crAss_like_phage	L7_Streptococcus_macedonicus	0.015	0.043	7.25.E-01	9.87.E-01
Herelleviridae	L7_Streptococcus_macedonicus	0.079	0.040	4.73.E-02	7.22.E-01
Microviridae	L7_Streptococcus_macedonicus	0.047	0.040	2.43.E-01	8.88.E-01
Myoviridae	L7_Streptococcus_macedonicus	0.033	0.045	4.65.E-01	9.45.E-01
Phycodnaviridae	L7_Streptococcus_macedonicus	0.000	0.040	9.99.E-01	9.99.E-01
Podoviridae	L7_Streptococcus_macedonicus	0.052	0.046	2.60.E-01	8.95.E-01
Siphoviridae	L7_Streptococcus_macedonicus	0.058	0.041	1.58.E-01	8.35.E-01
Autographiviridae	L7_Enterococcus_sp.	0.084	0.037	2.47.E-02	6.45.E-01
crAss_like_phage	L7_Enterococcus_sp.	-0.054	0.042	1.91.E-01	8.61.E-01
Herelleviridae	L7_Enterococcus_sp.	0.024	0.039	5.31.E-01	9.57.E-01
Microviridae	L7_Enterococcus_sp.	-0.010	0.039	7.97.E-01	9.92.E-01
Myoviridae	L7_Enterococcus_sp.	0.028	0.044	5.27.E-01	9.57.E-01
Phycodnaviridae	L7_Enterococcus_sp.	0.010	0.039	7.91.E-01	9.92.E-01
Podoviridae	L7_Enterococcus_sp.	0.008	0.045	8.67.E-01	9.96.E-01
Siphoviridae	L7_Enterococcus_sp.	-0.069	0.040	8.49.E-02	7.50.E-01
Autographiviridae	L7_Sutterella_sp.	-0.004	0.042	9.26.E-01	9.98.E-01
crAss_like_phage	L7_Sutterella_sp.	0.000	0.046	9.96.E-01	9.99.E-01
Herelleviridae	L7_Sutterella_sp.	-0.006	0.043	8.89.E-01	9.96.E-01
Microviridae	L7_Sutterella_sp.	0.060	0.043	1.62.E-01	8.38.E-01
Myoviridae	L7_Sutterella_sp.	0.067	0.048	1.64.E-01	8.40.E-01
Phycodnaviridae	L7_Sutterella_sp.	0.024	0.043	5.67.E-01	9.61.E-01
Podoviridae	L7_Sutterella_sp.	0.029	0.050	5.57.E-01	9.61.E-01
Siphoviridae	L7_Sutterella_sp.	0.035	0.044	4.36.E-01	9.26.E-01
Autographiviridae	L7_Cloacibacillus_evryensis	0.080	0.038	3.70.E-02	6.91.E-01
crAss_like_phage	L7_Cloacibacillus_evryensis	-0.024	0.042	5.66.E-01	9.61.E-01
Herelleviridae	L7_Cloacibacillus_evryensis	-0.004	0.040	9.11.E-01	9.98.E-01
Microviridae	L7_Cloacibacillus_evryensis	0.022	0.040	5.90.E-01	9.68.E-01
Myoviridae	L7_Cloacibacillus_evryensis	-0.017	0.045	7.06.E-01	9.82.E-01
Phycodnaviridae	L7_Cloacibacillus_evryensis	-0.046	0.039	2.46.E-01	8.92.E-01
Podoviridae	L7_Cloacibacillus_evryensis	-0.072	0.046	1.20.E-01	7.91.E-01
Siphoviridae	L7_Cloacibacillus_evryensis	-0.007	0.041	8.74.E-01	9.96.E-01
Autographiviridae	L7_Gordonibacter_pamelaeae	0.030	0.036	3.98.E-01	9.15.E-01
crAss_like_phage	L7_Gordonibacter_pamelaeae	-0.041	0.039	2.95.E-01	9.15.E-01
Herelleviridae	L7_Gordonibacter_pamelaeae	0.014	0.037	7.11.E-01	9.85.E-01
Microviridae	L7_Gordonibacter_pamelaeae	-0.026	0.037	4.80.E-01	9.53.E-01
Myoviridae	L7_Gordonibacter_pamelaeae	-0.061	0.041	1.43.E-01	8.12.E-01
Phycodnaviridae	L7_Gordonibacter_pamelaeae	0.017	0.037	6.44.E-01	9.79.E-01
Podoviridae	L7_Gordonibacter_pamelaeae	0.009	0.043	8.31.E-01	9.94.E-01
Siphoviridae	L7_Gordonibacter_pamelaeae	0.018	0.038	6.44.E-01	9.79.E-01
Autographiviridae	L7_Bacteroides_plebeius	0.007	0.036	8.44.E-01	9.95.E-01
crAss_like_phage	L7_Bacteroides_plebeius	-0.026	0.039	5.06.E-01	9.57.E-01
Herelleviridae	L7_Bacteroides_plebeius	-0.060	0.037	1.05.E-01	7.82.E-01
Microviridae	L7_Bacteroides_plebeius	-0.011	0.037	7.72.E-01	9.92.E-01
Myoviridae	L7_Bacteroides_plebeius	0.025	0.041	5.54.E-01	9.60.E-01
Phycodnaviridae	L7_Bacteroides_plebeius	-0.068	0.036	6.47.E-02	7.24.E-01
Podoviridae	L7_Bacteroides_plebeius	0.079	0.043	6.49.E-02	7.24.E-01
Siphoviridae	L7_Bacteroides_plebeius	0.013	0.038	7.37.E-01	9.88.E-01
Autographiviridae	L7_Gabonia_massiliensis	-0.011	0.037	7.67.E-01	9.91.E-01
crAss_like_phage	L7_Gabonia_massiliensis	0.015	0.041	7.18.E-01	9.86.E-01
Herelleviridae	L7_Gabonia_massiliensis	0.075	0.039	5.36.E-02	7.22.E-01
Microviridae	L7_Gabonia_massiliensis	-0.027	0.039	4.97.E-01	9.55.E-01
Myoviridae	L7_Gabonia_massiliensis	0.007	0.044	8.66.E-01	9.96.E-01

Phycodnaviridae	L7_Gabonia_massiliensis	0.003	0.038	9.46.E-01	9.98.E-01
Podoviridae	L7_Gabonia_massiliensis	0.003	0.045	9.52.E-01	9.98.E-01
Siphoviridae	L7_Gabonia_massiliensis	-0.004	0.040	9.13.E-01	9.98.E-01
Autographiviridae	L7_Bifidobacterium_pullorum	-0.016	0.039	6.77.E-01	9.80.E-01
crAss_like_phage	L7_Bifidobacterium_pullorum	0.009	0.043	8.29.E-01	9.94.E-01
Herelleviridae	L7_Bifidobacterium_pullorum	0.036	0.041	3.78.E-01	9.15.E-01
Microviridae	L7_Bifidobacterium_pullorum	0.051	0.041	2.15.E-01	8.79.E-01
Myoviridae	L7_Bifidobacterium_pullorum	-0.011	0.045	8.10.E-01	9.93.E-01
Phycodnaviridae	L7_Bifidobacterium_pullorum	-0.008	0.040	8.51.E-01	9.95.E-01
Podoviridae	L7_Bifidobacterium_pullorum	0.021	0.047	6.58.E-01	9.79.E-01
Siphoviridae	L7_Bifidobacterium_pullorum	-0.014	0.042	7.43.E-01	9.89.E-01
Autographiviridae	L7_Peptoclostridium_sp.	-0.078	0.039	4.48.E-02	7.22.E-01
crAss_like_phage	L7_Peptoclostridium_sp.	-0.013	0.043	7.54.E-01	9.89.E-01
Herelleviridae	L7_Peptoclostridium_sp.	-0.037	0.041	3.67.E-01	9.15.E-01
Microviridae	L7_Peptoclostridium_sp.	-0.055	0.041	1.74.E-01	8.56.E-01
Myoviridae	L7_Peptoclostridium_sp.	0.034	0.045	4.53.E-01	9.38.E-01
Phycodnaviridae	L7_Peptoclostridium_sp.	0.009	0.040	8.26.E-01	9.93.E-01
Podoviridae	L7_Peptoclostridium_sp.	-0.034	0.047	4.64.E-01	9.45.E-01
Siphoviridae	L7_Peptoclostridium_sp.	-0.029	0.042	4.79.E-01	9.53.E-01
Autographiviridae	L7_Turicibacter_sanguinis	0.045	0.035	2.03.E-01	8.75.E-01
crAss_like_phage	L7_Turicibacter_sanguinis	-0.002	0.039	9.63.E-01	9.98.E-01
Herelleviridae	L7_Turicibacter_sanguinis	0.097	0.036	7.72.E-03	4.66.E-01
Microviridae	L7_Turicibacter_sanguinis	0.051	0.036	1.65.E-01	8.43.E-01
Myoviridae	L7_Turicibacter_sanguinis	-0.043	0.041	2.95.E-01	9.15.E-01
Phycodnaviridae	L7_Turicibacter_sanguinis	0.057	0.036	1.11.E-01	7.82.E-01
Podoviridae	L7_Turicibacter_sanguinis	0.018	0.042	6.76.E-01	9.80.E-01
Siphoviridae	L7_Turicibacter_sanguinis	-0.038	0.037	3.09.E-01	9.15.E-01
Autographiviridae	L7_Acidaminococcus_intestini	0.012	0.044	7.84.E-01	9.92.E-01
crAss_like_phage	L7_Acidaminococcus_intestini	-0.093	0.049	5.55.E-02	7.24.E-01
Herelleviridae	L7_Acidaminococcus_intestini	-0.002	0.046	9.63.E-01	9.98.E-01
Microviridae	L7_Acidaminococcus_intestini	-0.034	0.046	4.60.E-01	9.43.E-01
Myoviridae	L7_Acidaminococcus_intestini	-0.018	0.051	7.21.E-01	9.86.E-01
Phycodnaviridae	L7_Acidaminococcus_intestini	0.022	0.045	6.20.E-01	9.75.E-01
Podoviridae	L7_Acidaminococcus_intestini	-0.008	0.053	8.74.E-01	9.96.E-01
Siphoviridae	L7_Acidaminococcus_intestini	-0.002	0.047	9.66.E-01	9.98.E-01
Autographiviridae	L7_Bacteroides_stercorirosoris	0.047	0.028	9.03.E-02	7.58.E-01
crAss_like_phage	L7_Bacteroides_stercorirosoris	0.026	0.031	3.98.E-01	9.15.E-01
Herelleviridae	L7_Bacteroides_stercorirosoris	0.010	0.029	7.22.E-01	9.86.E-01
Microviridae	L7_Bacteroides_stercorirosoris	0.026	0.029	3.72.E-01	9.15.E-01
Myoviridae	L7_Bacteroides_stercorirosoris	0.019	0.033	5.50.E-01	9.60.E-01
Phycodnaviridae	L7_Bacteroides_stercorirosoris	-0.046	0.029	1.11.E-01	7.82.E-01
Podoviridae	L7_Bacteroides_stercorirosoris	-0.004	0.034	9.02.E-01	9.97.E-01
Siphoviridae	L7_Bacteroides_stercorirosoris	-0.067	0.030	2.60.E-02	6.56.E-01
Autographiviridae	L7_Megasphaera_micronuciformis	-0.016	0.034	6.40.E-01	9.79.E-01
crAss_like_phage	L7_Megasphaera_micronuciformis	-0.034	0.037	3.63.E-01	9.15.E-01
Herelleviridae	L7_Megasphaera_micronuciformis	0.015	0.035	6.78.E-01	9.80.E-01
Microviridae	L7_Megasphaera_micronuciformis	-0.008	0.035	8.17.E-01	9.93.E-01
Myoviridae	L7_Megasphaera_micronuciformis	-0.023	0.039	5.61.E-01	9.61.E-01
Phycodnaviridae	L7_Megasphaera_micronuciformis	0.005	0.035	8.74.E-01	9.96.E-01
Podoviridae	L7_Megasphaera_micronuciformis	0.015	0.040	7.15.E-01	9.86.E-01
Siphoviridae	L7_Megasphaera_micronuciformis	0.030	0.036	4.11.E-01	9.18.E-01
Autographiviridae	L7_Prevotella_corporis	0.044	0.024	6.59.E-02	7.24.E-01
crAss_like_phage	L7_Prevotella_corporis	-0.006	0.026	8.19.E-01	9.93.E-01
Herelleviridae	L7_Prevotella_corporis	-0.020	0.025	4.11.E-01	9.18.E-01
Microviridae	L7_Prevotella_corporis	0.003	0.025	9.16.E-01	9.98.E-01
Myoviridae	L7_Prevotella_corporis	-0.027	0.028	3.25.E-01	9.15.E-01
Phycodnaviridae	L7_Prevotella_corporis	0.006	0.024	8.14.E-01	9.93.E-01
Podoviridae	L7_Prevotella_corporis	-0.008	0.028	7.70.E-01	9.91.E-01
Siphoviridae	L7_Prevotella_corporis	-0.015	0.025	5.47.E-01	9.60.E-01
Autographiviridae	L7_Porphyromonas_somerae	0.040	0.023	8.65.E-02	7.52.E-01
crAss_like_phage	L7_Porphyromonas_somerae	0.032	0.026	2.16.E-01	8.79.E-01
Herelleviridae	L7_Porphyromonas_somerae	-0.016	0.024	5.13.E-01	9.57.E-01
Microviridae	L7_Porphyromonas_somerae	-0.001	0.024	9.59.E-01	9.98.E-01
Myoviridae	L7_Porphyromonas_somerae	0.010	0.027	7.00.E-01	9.81.E-01
Phycodnaviridae	L7_Porphyromonas_somerae	-0.026	0.024	2.82.E-01	9.13.E-01

Podoviridae	L7_Porphyromonas_somerae	-0.027	0.028	3.37.E-01	9.15.E-01
Siphoviridae	L7_Porphyromonas_somerae	-0.018	0.025	4.58.E-01	9.42.E-01
Autographiviridae	L7_Streptococcus_lutetiensis	0.018	0.043	6.69.E-01	9.80.E-01
crAss_like_phage	L7_Streptococcus_lutetiensis	0.045	0.047	3.37.E-01	9.15.E-01
Herelleviridae	L7_Streptococcus_lutetiensis	0.068	0.044	1.22.E-01	7.93.E-01
Microviridae	L7_Streptococcus_lutetiensis	0.019	0.044	6.62.E-01	9.79.E-01
Myoviridae	L7_Streptococcus_lutetiensis	-0.042	0.049	3.91.E-01	9.15.E-01
Phycodnaviridae	L7_Streptococcus_lutetiensis	0.018	0.044	6.77.E-01	9.80.E-01
Podoviridae	L7_Streptococcus_lutetiensis	-0.087	0.051	8.83.E-02	7.54.E-01
Siphoviridae	L7_Streptococcus_lutetiensis	0.064	0.045	1.59.E-01	8.38.E-01
Autographiviridae	L7_Lactobacillus_ruminis	0.041	0.040	3.01.E-01	9.15.E-01
crAss_like_phage	L7_Lactobacillus_ruminis	-0.035	0.044	4.21.E-01	9.20.E-01
Herelleviridae	L7_Lactobacillus_ruminis	0.005	0.041	9.00.E-01	9.97.E-01
Microviridae	L7_Lactobacillus_ruminis	-0.108	0.041	8.95.E-03	4.93.E-01
Myoviridae	L7_Lactobacillus_ruminis	-0.048	0.046	3.00.E-01	9.15.E-01
Phycodnaviridae	L7_Lactobacillus_ruminis	0.002	0.041	9.61.E-01	9.98.E-01
Podoviridae	L7_Lactobacillus_ruminis	-0.004	0.048	9.31.E-01	9.98.E-01
Siphoviridae	L7_Lactobacillus_ruminis	-0.035	0.043	4.11.E-01	9.17.E-01
Autographiviridae	L7_Collinsella_ihuae	0.024	0.039	5.39.E-01	9.60.E-01
crAss_like_phage	L7_Collinsella_ihuae	0.009	0.043	8.36.E-01	9.94.E-01
Herelleviridae	L7_Collinsella_ihuae	-0.027	0.041	5.11.E-01	9.57.E-01
Microviridae	L7_Collinsella_ihuae	-0.071	0.041	8.25.E-02	7.49.E-01
Myoviridae	L7_Collinsella_ihuae	0.003	0.046	9.42.E-01	9.98.E-01
Phycodnaviridae	L7_Collinsella_ihuae	0.018	0.040	6.55.E-01	9.79.E-01
Podoviridae	L7_Collinsella_ihuae	0.050	0.047	2.91.E-01	9.15.E-01
Siphoviridae	L7_Collinsella_ihuae	-0.026	0.042	5.40.E-01	9.60.E-01
Autographiviridae	L7_Lactobacillus_plantarum	-0.010	0.039	8.04.E-01	9.93.E-01
crAss_like_phage	L7_Lactobacillus_plantarum	0.022	0.043	6.11.E-01	9.75.E-01
Herelleviridae	L7_Lactobacillus_plantarum	0.013	0.041	7.53.E-01	9.89.E-01
Microviridae	L7_Lactobacillus_plantarum	0.052	0.041	1.96.E-01	8.66.E-01
Myoviridae	L7_Lactobacillus_plantarum	0.047	0.045	2.99.E-01	9.15.E-01
Phycodnaviridae	L7_Lactobacillus_plantarum	0.023	0.040	5.66.E-01	9.61.E-01
Podoviridae	L7_Lactobacillus_plantarum	0.011	0.047	8.15.E-01	9.93.E-01
Siphoviridae	L7_Lactobacillus_plantarum	0.060	0.042	1.53.E-01	8.24.E-01
Autographiviridae	L7_Bacteroides_sp.	-0.007	0.019	7.18.E-01	9.86.E-01
crAss_like_phage	L7_Bacteroides_sp.	-0.039	0.021	7.15.E-02	7.24.E-01
Herelleviridae	L7_Bacteroides_sp.	-0.004	0.020	8.32.E-01	9.94.E-01
Microviridae	L7_Bacteroides_sp.	-0.028	0.020	1.69.E-01	8.46.E-01
Myoviridae	L7_Bacteroides_sp.	-0.019	0.023	3.90.E-01	9.15.E-01
Phycodnaviridae	L7_Bacteroides_sp.	-0.016	0.020	4.14.E-01	9.18.E-01
Podoviridae	L7_Bacteroides_sp.	-0.065	0.023	5.13.E-03	4.28.E-01
Siphoviridae	L7_Bacteroides_sp.	-0.062	0.021	2.60.E-03	3.52.E-01
Autographiviridae	L7_Rothia_mucilaginosa	0.058	0.033	7.96.E-02	7.43.E-01
crAss_like_phage	L7_Rothia_mucilaginosa	0.026	0.036	4.71.E-01	9.51.E-01
Herelleviridae	L7_Rothia_mucilaginosa	0.046	0.034	1.82.E-01	8.60.E-01
Microviridae	L7_Rothia_mucilaginosa	-0.011	0.034	7.49.E-01	9.89.E-01
Myoviridae	L7_Rothia_mucilaginosa	0.098	0.038	9.91.E-03	5.05.E-01
Phycodnaviridae	L7_Rothia_mucilaginosa	-0.010	0.034	7.72.E-01	9.92.E-01
Podoviridae	L7_Rothia_mucilaginosa	0.030	0.040	4.50.E-01	9.37.E-01
Siphoviridae	L7_Rothia_mucilaginosa	0.038	0.035	2.86.E-01	9.15.E-01
Autographiviridae	L7_Escherichia_sp.	0.009	0.031	7.82.E-01	9.92.E-01
crAss_like_phage	L7_Escherichia_sp.	0.028	0.035	4.18.E-01	9.19.E-01
Herelleviridae	L7_Escherichia_sp.	0.014	0.033	6.66.E-01	9.80.E-01
Microviridae	L7_Escherichia_sp.	0.025	0.033	4.52.E-01	9.38.E-01
Myoviridae	L7_Escherichia_sp.	0.070	0.036	5.48.E-02	7.23.E-01
Phycodnaviridae	L7_Escherichia_sp.	0.020	0.032	5.38.E-01	9.60.E-01
Podoviridae	L7_Escherichia_sp.	-0.016	0.038	6.72.E-01	9.80.E-01
Siphoviridae	L7_Escherichia_sp.	0.032	0.034	3.43.E-01	9.15.E-01
Autographiviridae	L7_Bifidobacterium_saguini	0.026	0.037	4.86.E-01	9.54.E-01
crAss_like_phage	L7_Bifidobacterium_saguini	0.065	0.040	1.08.E-01	7.82.E-01
Herelleviridae	L7_Bifidobacterium_saguini	-0.009	0.038	8.16.E-01	9.93.E-01
Microviridae	L7_Bifidobacterium_saguini	0.073	0.038	5.52.E-02	7.24.E-01
Myoviridae	L7_Bifidobacterium_saguini	0.059	0.042	1.67.E-01	8.44.E-01
Phycodnaviridae	L7_Bifidobacterium_saguini	0.000	0.038	9.96.E-01	9.99.E-01
Podoviridae	L7_Bifidobacterium_saguini	0.036	0.044	4.12.E-01	9.18.E-01

Siphoviridae	L7_Bifidobacterium_saguini	0.012	0.039	7.51.E-01	9.89.E-01
Autographiviridae	L7_Bifidobacterium_gallinarum	-0.046	0.038	2.31.E-01	8.82.E-01
crAss_like_phage	L7_Bifidobacterium_gallinarum	0.033	0.042	4.39.E-01	9.30.E-01
Herelleviridae	L7_Bifidobacterium_gallinarum	0.028	0.040	4.85.E-01	9.54.E-01
Microviridae	L7_Bifidobacterium_gallinarum	0.048	0.040	2.30.E-01	8.82.E-01
Myoviridae	L7_Bifidobacterium_gallinarum	-0.037	0.044	4.01.E-01	9.15.E-01
Phycodnaviridae	L7_Bifidobacterium_gallinarum	0.045	0.039	2.49.E-01	8.94.E-01
Podoviridae	L7_Bifidobacterium_gallinarum	0.025	0.046	5.84.E-01	9.65.E-01
Siphoviridae	L7_Bifidobacterium_gallinarum	0.029	0.041	4.73.E-01	9.52.E-01
Autographiviridae	L7_Meiothermus_ruber	0.000	0.008	9.83.E-01	9.99.E-01
crAss_like_phage	L7_Meiothermus_ruber	-0.014	0.009	1.09.E-01	7.82.E-01
Herelleviridae	L7_Meiothermus_ruber	-0.003	0.008	7.19.E-01	9.86.E-01
Microviridae	L7_Meiothermus_ruber	0.007	0.008	3.76.E-01	9.15.E-01
Myoviridae	L7_Meiothermus_ruber	-0.007	0.009	4.21.E-01	9.20.E-01
Phycodnaviridae	L7_Meiothermus_ruber	-0.004	0.008	5.84.E-01	9.65.E-01
Podoviridae	L7_Meiothermus_ruber	-0.010	0.009	2.68.E-01	9.01.E-01
Siphoviridae	L7_Meiothermus_ruber	-0.011	0.008	1.78.E-01	8.59.E-01
Autographiviridae	L7_Clostridium_saccharogumia	0.039	0.040	3.32.E-01	9.15.E-01
crAss_like_phage	L7_Clostridium_saccharogumia	0.048	0.045	2.82.E-01	9.13.E-01
Herelleviridae	L7_Clostridium_saccharogumia	-0.024	0.042	5.62.E-01	9.61.E-01
Microviridae	L7_Clostridium_saccharogumia	-0.052	0.042	2.16.E-01	8.79.E-01
Myoviridae	L7_Clostridium_saccharogumia	0.141	0.047	2.63.E-03	3.52.E-01
Phycodnaviridae	L7_Clostridium_saccharogumia	0.041	0.041	3.24.E-01	9.15.E-01
Podoviridae	L7_Clostridium_saccharogumia	0.010	0.049	8.29.E-01	9.94.E-01
Siphoviridae	L7_Clostridium_saccharogumia	0.012	0.043	7.73.E-01	9.92.E-01
Autographiviridae	L7_Lactobacillus_reuteri	-0.047	0.038	2.11.E-01	8.78.E-01
crAss_like_phage	L7_Lactobacillus_reuteri	-0.048	0.042	2.56.E-01	8.95.E-01
Herelleviridae	L7_Lactobacillus_reuteri	0.014	0.039	7.25.E-01	9.87.E-01
Microviridae	L7_Lactobacillus_reuteri	-0.086	0.039	2.87.E-02	6.66.E-01
Myoviridae	L7_Lactobacillus_reuteri	-0.112	0.044	1.05.E-02	5.13.E-01
Phycodnaviridae	L7_Lactobacillus_reuteri	0.014	0.039	7.27.E-01	9.87.E-01
Podoviridae	L7_Lactobacillus_reuteri	-0.015	0.045	7.49.E-01	9.89.E-01
Siphoviridae	L7_Lactobacillus_reuteri	0.021	0.040	6.08.E-01	9.75.E-01
Autographiviridae	L7_Romboutsia_sp.	0.007	0.028	8.15.E-01	9.93.E-01
crAss_like_phage	L7_Romboutsia_sp.	0.026	0.031	4.07.E-01	9.17.E-01
Herelleviridae	L7_Romboutsia_sp.	0.044	0.029	1.33.E-01	8.05.E-01
Microviridae	L7_Romboutsia_sp.	-0.050	0.029	8.88.E-02	7.56.E-01
Myoviridae	L7_Romboutsia_sp.	-0.046	0.033	1.60.E-01	8.38.E-01
Phycodnaviridae	L7_Romboutsia_sp.	-0.024	0.029	4.06.E-01	9.17.E-01
Podoviridae	L7_Romboutsia_sp.	0.004	0.034	8.96.E-01	9.97.E-01
Siphoviridae	L7_Romboutsia_sp.	-0.010	0.030	7.35.E-01	9.88.E-01
Autographiviridae	L7_Bifidobacterium_sp.	-0.010	0.032	7.53.E-01	9.89.E-01
crAss_like_phage	L7_Bifidobacterium_sp.	0.004	0.036	9.14.E-01	9.98.E-01
Herelleviridae	L7_Bifidobacterium_sp.	0.008	0.034	8.06.E-01	9.93.E-01
Microviridae	L7_Bifidobacterium_sp.	0.061	0.034	7.05.E-02	7.24.E-01
Myoviridae	L7_Bifidobacterium_sp.	0.025	0.038	5.01.E-01	9.55.E-01
Phycodnaviridae	L7_Bifidobacterium_sp.	-0.006	0.033	8.60.E-01	9.96.E-01
Podoviridae	L7_Bifidobacterium_sp.	0.015	0.039	6.97.E-01	9.81.E-01
Siphoviridae	L7_Bifidobacterium_sp.	0.000	0.035	9.93.E-01	9.99.E-01
Autographiviridae	L7_Bacillus_sp.	-0.035	0.045	4.41.E-01	9.31.E-01
crAss_like_phage	L7_Bacillus_sp.	0.077	0.049	1.21.E-01	7.93.E-01
Herelleviridae	L7_Bacillus_sp.	-0.006	0.047	9.03.E-01	9.97.E-01
Microviridae	L7_Bacillus_sp.	-0.009	0.047	8.39.E-01	9.94.E-01
Myoviridae	L7_Bacillus_sp.	0.042	0.052	4.15.E-01	9.18.E-01
Phycodnaviridae	L7_Bacillus_sp.	0.056	0.046	2.26.E-01	8.82.E-01
Podoviridae	L7_Bacillus_sp.	0.018	0.054	7.35.E-01	9.88.E-01
Siphoviridae	L7_Bacillus_sp.	0.015	0.048	7.61.E-01	9.91.E-01
Autographiviridae	L7_Eggerthella_sp.	-0.012	0.038	7.52.E-01	9.89.E-01
crAss_like_phage	L7_Eggerthella_sp.	-0.039	0.042	3.55.E-01	9.15.E-01
Herelleviridae	L7_Eggerthella_sp.	0.045	0.039	2.51.E-01	8.95.E-01
Microviridae	L7_Eggerthella_sp.	-0.009	0.040	8.14.E-01	9.93.E-01
Myoviridae	L7_Eggerthella_sp.	-0.034	0.044	4.46.E-01	9.34.E-01
Phycodnaviridae	L7_Eggerthella_sp.	0.014	0.039	7.21.E-01	9.86.E-01
Podoviridae	L7_Eggerthella_sp.	0.048	0.046	2.98.E-01	9.15.E-01
Siphoviridae	L7_Eggerthella_sp.	-0.083	0.040	4.01.E-02	7.03.E-01

Autographiviridae	L7_Bacteroides_vulgatus	0.022	0.032	4.76.E-01	9.52.E-01
crAss_like_phage	L7_Bacteroides_vulgatus	-0.040	0.035	2.47.E-01	8.93.E-01
Herelleviridae	L7_Bacteroides_vulgatus	-0.041	0.033	2.16.E-01	8.79.E-01
Microviridae	L7_Bacteroides_vulgatus	-0.011	0.033	7.39.E-01	9.88.E-01
Myoviridae	L7_Bacteroides_vulgatus	-0.037	0.037	3.17.E-01	9.15.E-01
Phycodnaviridae	L7_Bacteroides_vulgatus	0.012	0.032	7.18.E-01	9.86.E-01
Podoviridae	L7_Bacteroides_vulgatus	-0.002	0.038	9.68.E-01	9.98.E-01
Siphoviridae	L7_Bacteroides_vulgatus	-0.014	0.034	6.84.E-01	9.80.E-01
Autographiviridae	L7_Bacteroides_fluxus	0.036	0.033	2.85.E-01	9.15.E-01
crAss_like_phage	L7_Bacteroides_fluxus	-0.004	0.037	9.21.E-01	9.98.E-01
Herelleviridae	L7_Bacteroides_fluxus	-0.010	0.035	7.65.E-01	9.91.E-01
Microviridae	L7_Bacteroides_fluxus	0.033	0.035	3.40.E-01	9.15.E-01
Myoviridae	L7_Bacteroides_fluxus	0.047	0.039	2.24.E-01	8.82.E-01
Phycodnaviridae	L7_Bacteroides_fluxus	0.022	0.034	5.20.E-01	9.57.E-01
Podoviridae	L7_Bacteroides_fluxus	0.049	0.040	2.21.E-01	8.79.E-01
Siphoviridae	L7_Bacteroides_fluxus	0.098	0.035	5.76.E-03	4.40.E-01
Autographiviridae	L7_Acetobacter_sp.	0.058	0.038	1.29.E-01	7.99.E-01
crAss_like_phage	L7_Acetobacter_sp.	-0.090	0.042	3.39.E-02	6.82.E-01
Herelleviridae	L7_Acetobacter_sp.	-0.004	0.040	9.11.E-01	9.98.E-01
Microviridae	L7_Acetobacter_sp.	0.029	0.040	4.69.E-01	9.50.E-01
Myoviridae	L7_Acetobacter_sp.	0.029	0.044	5.16.E-01	9.57.E-01
Phycodnaviridae	L7_Acetobacter_sp.	-0.017	0.039	6.64.E-01	9.79.E-01
Podoviridae	L7_Acetobacter_sp.	-0.048	0.046	2.94.E-01	9.15.E-01
Siphoviridae	L7_Acetobacter_sp.	-0.007	0.041	8.72.E-01	9.96.E-01
Autographiviridae	L7_Intestinimonas_massiliensis	-0.004	0.027	8.74.E-01	9.96.E-01
crAss_like_phage	L7_Intestinimonas_massiliensis	0.010	0.029	7.24.E-01	9.86.E-01
Herelleviridae	L7_Intestinimonas_massiliensis	0.004	0.028	8.95.E-01	9.97.E-01
Microviridae	L7_Intestinimonas_massiliensis	0.043	0.028	1.23.E-01	7.94.E-01
Myoviridae	L7_Intestinimonas_massiliensis	0.069	0.031	2.56.E-02	6.50.E-01
Phycodnaviridae	L7_Intestinimonas_massiliensis	0.021	0.027	4.35.E-01	9.26.E-01
Podoviridae	L7_Intestinimonas_massiliensis	0.007	0.032	8.20.E-01	9.93.E-01
Siphoviridae	L7_Intestinimonas_massiliensis	0.005	0.028	8.56.E-01	9.96.E-01
Autographiviridae	L7_Ruminococcus_bromii	0.029	0.038	4.42.E-01	9.32.E-01
crAss_like_phage	L7_Ruminococcus_bromii	0.064	0.042	1.24.E-01	7.94.E-01
Herelleviridae	L7_Ruminococcus_bromii	0.025	0.039	5.31.E-01	9.57.E-01
Microviridae	L7_Ruminococcus_bromii	0.071	0.039	6.97.E-02	7.24.E-01
Myoviridae	L7_Ruminococcus_bromii	0.025	0.044	5.77.E-01	9.63.E-01
Phycodnaviridae	L7_Ruminococcus_bromii	0.009	0.039	8.23.E-01	9.93.E-01
Podoviridae	L7_Ruminococcus_bromii	0.023	0.045	6.18.E-01	9.75.E-01
Siphoviridae	L7_Ruminococcus_bromii	-0.016	0.040	6.95.E-01	9.81.E-01
Autographiviridae	L7_Blautia_obeum	0.006	0.033	8.61.E-01	9.96.E-01
crAss_like_phage	L7_Blautia_obeum	-0.012	0.036	7.33.E-01	9.88.E-01
Herelleviridae	L7_Blautia_obeum	-0.016	0.034	6.35.E-01	9.76.E-01
Microviridae	L7_Blautia_obeum	-0.059	0.034	8.61.E-02	7.52.E-01
Myoviridae	L7_Blautia_obeum	-0.043	0.038	2.66.E-01	9.01.E-01
Phycodnaviridae	L7_Blautia_obeum	-0.030	0.034	3.73.E-01	9.15.E-01
Podoviridae	L7_Blautia_obeum	-0.099	0.039	1.15.E-02	5.36.E-01
Siphoviridae	L7_Blautia_obeum	-0.073	0.035	3.66.E-02	6.90.E-01
Autographiviridae	L7_Anaeroglobus_geminatus	0.079	0.041	5.67.E-02	7.24.E-01
crAss_like_phage	L7_Anaeroglobus_geminatus	0.012	0.046	7.92.E-01	9.92.E-01
Herelleviridae	L7_Anaeroglobus_geminatus	-0.053	0.043	2.20.E-01	8.79.E-01
Microviridae	L7_Anaeroglobus_geminatus	-0.080	0.043	6.41.E-02	7.24.E-01
Myoviridae	L7_Anaeroglobus_geminatus	-0.047	0.048	3.27.E-01	9.15.E-01
Phycodnaviridae	L7_Anaeroglobus_geminatus	-0.001	0.043	9.74.E-01	9.99.E-01
Podoviridae	L7_Anaeroglobus_geminatus	0.017	0.050	7.32.E-01	9.88.E-01
Siphoviridae	L7_Anaeroglobus_geminatus	-0.014	0.044	7.51.E-01	9.89.E-01
Autographiviridae	L7_Fusobacterium_mortiferum	-0.005	0.043	9.05.E-01	9.97.E-01
crAss_like_phage	L7_Fusobacterium_mortiferum	-0.051	0.047	2.85.E-01	9.15.E-01
Herelleviridae	L7_Fusobacterium_mortiferum	0.015	0.045	7.32.E-01	9.88.E-01
Microviridae	L7_Fusobacterium_mortiferum	-0.002	0.045	9.68.E-01	9.98.E-01
Myoviridae	L7_Fusobacterium_mortiferum	0.049	0.050	3.26.E-01	9.15.E-01
Phycodnaviridae	L7_Fusobacterium_mortiferum	0.105	0.044	1.70.E-02	5.86.E-01
Podoviridae	L7_Fusobacterium_mortiferum	0.049	0.051	3.37.E-01	9.15.E-01
Siphoviridae	L7_Fusobacterium_mortiferum	-0.010	0.046	8.31.E-01	9.94.E-01
Autographiviridae	L7_Catenibacterium_mitsuokai	0.019	0.040	6.33.E-01	9.76.E-01

crAss_like_phage	L7_Catenibacterium_mitsuokai	-0.011	0.044	7.99.E-01	9.92.E-01
Herelleviridae	L7_Catenibacterium_mitsuokai	0.033	0.042	4.33.E-01	9.26.E-01
Microviridae	L7_Catenibacterium_mitsuokai	-0.006	0.042	8.93.E-01	9.97.E-01
Myoviridae	L7_Catenibacterium_mitsuokai	0.057	0.046	2.20.E-01	8.79.E-01
Phycodnaviridae	L7_Catenibacterium_mitsuokai	-0.072	0.041	8.14.E-02	7.47.E-01
Podoviridae	L7_Catenibacterium_mitsuokai	0.026	0.048	5.90.E-01	9.68.E-01
Siphoviridae	L7_Catenibacterium_mitsuokai	-0.039	0.043	3.64.E-01	9.15.E-01
Autographiviridae	L7_Acinetobacter_sp.	-0.059	0.044	1.81.E-01	8.60.E-01
crAss_like_phage	L7_Acinetobacter_sp.	-0.057	0.048	2.38.E-01	8.85.E-01
Herelleviridae	L7_Acinetobacter_sp.	-0.028	0.046	5.39.E-01	9.60.E-01
Microviridae	L7_Acinetobacter_sp.	0.001	0.046	9.75.E-01	9.99.E-01
Myoviridae	L7_Acinetobacter_sp.	0.011	0.051	8.33.E-01	9.94.E-01
Phycodnaviridae	L7_Acinetobacter_sp.	0.029	0.045	5.21.E-01	9.57.E-01
Podoviridae	L7_Acinetobacter_sp.	0.015	0.053	7.80.E-01	9.92.E-01
Siphoviridae	L7_Acinetobacter_sp.	0.008	0.047	8.73.E-01	9.96.E-01
Autographiviridae	L7_Granulicatella_adiacens	-0.034	0.026	1.97.E-01	8.66.E-01
crAss_like_phage	L7_Granulicatella_adiacens	0.025	0.029	3.95.E-01	9.15.E-01
Herelleviridae	L7_Granulicatella_adiacens	-0.089	0.027	1.09.E-03	2.79.E-01
Microviridae	L7_Granulicatella_adiacens	0.010	0.028	7.16.E-01	9.86.E-01
Myoviridae	L7_Granulicatella_adiacens	-0.059	0.031	5.34.E-02	7.22.E-01
Phycodnaviridae	L7_Granulicatella_adiacens	-0.031	0.027	2.60.E-01	8.95.E-01
Podoviridae	L7_Granulicatella_adiacens	0.070	0.032	2.69.E-02	6.58.E-01
Siphoviridae	L7_Granulicatella_adiacens	0.034	0.028	2.26.E-01	8.82.E-01
Autographiviridae	L7_Lachnospira_sp.	0.011	0.033	7.31.E-01	9.88.E-01
crAss_like_phage	L7_Lachnospira_sp.	0.015	0.037	6.79.E-01	9.80.E-01
Herelleviridae	L7_Lachnospira_sp.	0.057	0.035	1.01.E-01	7.79.E-01
Microviridae	L7_Lachnospira_sp.	0.009	0.035	8.07.E-01	9.93.E-01
Myoviridae	L7_Lachnospira_sp.	-0.001	0.039	9.87.E-01	9.99.E-01
Phycodnaviridae	L7_Lachnospira_sp.	0.019	0.034	5.78.E-01	9.64.E-01
Podoviridae	L7_Lachnospira_sp.	0.037	0.040	3.54.E-01	9.15.E-01
Siphoviridae	L7_Lachnospira_sp.	0.057	0.036	1.07.E-01	7.82.E-01
Autographiviridae	L7_Mediterraneibacter_massiliensis	-0.007	0.039	8.53.E-01	9.95.E-01
crAss_like_phage	L7_Mediterraneibacter_massiliensis	0.057	0.043	1.89.E-01	8.61.E-01
Herelleviridae	L7_Mediterraneibacter_massiliensis	0.063	0.041	1.24.E-01	7.94.E-01
Microviridae	L7_Mediterraneibacter_massiliensis	0.002	0.041	9.63.E-01	9.98.E-01
Myoviridae	L7_Mediterraneibacter_massiliensis	-0.004	0.046	9.33.E-01	9.98.E-01
Phycodnaviridae	L7_Mediterraneibacter_massiliensis	-0.014	0.040	7.33.E-01	9.88.E-01
Podoviridae	L7_Mediterraneibacter_massiliensis	-0.021	0.047	6.58.E-01	9.79.E-01
Siphoviridae	L7_Mediterraneibacter_massiliensis	0.127	0.042	2.41.E-03	3.52.E-01
Autographiviridae	L7_Actinobaculum_sp.	0.022	0.039	5.72.E-01	9.61.E-01
crAss_like_phage	L7_Actinobaculum_sp.	0.036	0.043	4.00.E-01	9.15.E-01
Herelleviridae	L7_Actinobaculum_sp.	-0.037	0.040	3.64.E-01	9.15.E-01
Microviridae	L7_Actinobaculum_sp.	-0.038	0.040	3.50.E-01	9.15.E-01
Myoviridae	L7_Actinobaculum_sp.	-0.080	0.045	7.59.E-02	7.31.E-01
Phycodnaviridae	L7_Actinobaculum_sp.	0.048	0.040	2.24.E-01	8.82.E-01
Podoviridae	L7_Actinobaculum_sp.	0.056	0.047	2.29.E-01	8.82.E-01
Siphoviridae	L7_Actinobaculum_sp.	-0.020	0.041	6.34.E-01	9.76.E-01
Autographiviridae	L7_Collinsella_aerofaciens	0.002	0.035	9.63.E-01	9.98.E-01
crAss_like_phage	L7_Collinsella_aerofaciens	-0.003	0.039	9.45.E-01	9.98.E-01
Herelleviridae	L7_Collinsella_aerofaciens	0.052	0.036	1.57.E-01	8.33.E-01
Microviridae	L7_Collinsella_aerofaciens	-0.021	0.037	5.68.E-01	9.61.E-01
Myoviridae	L7_Collinsella_aerofaciens	-0.046	0.041	2.65.E-01	9.01.E-01
Phycodnaviridae	L7_Collinsella_aerofaciens	0.035	0.036	3.33.E-01	9.15.E-01
Podoviridae	L7_Collinsella_aerofaciens	-0.034	0.042	4.24.E-01	9.23.E-01
Siphoviridae	L7_Collinsella_aerofaciens	0.001	0.038	9.81.E-01	9.99.E-01
Autographiviridae	L7_Butyricicoccus_sp.	-0.033	0.033	3.22.E-01	9.15.E-01
crAss_like_phage	L7_Butyricicoccus_sp.	0.000	0.037	9.92.E-01	9.99.E-01
Herelleviridae	L7_Butyricicoccus_sp.	0.011	0.034	7.43.E-01	9.89.E-01
Microviridae	L7_Butyricicoccus_sp.	-0.046	0.034	1.86.E-01	8.61.E-01
Myoviridae	L7_Butyricicoccus_sp.	0.071	0.038	6.35.E-02	7.24.E-01
Phycodnaviridae	L7_Butyricicoccus_sp.	-0.006	0.034	8.59.E-01	9.96.E-01
Podoviridae	L7_Butyricicoccus_sp.	0.071	0.040	7.53.E-02	7.30.E-01
Siphoviridae	L7_Butyricicoccus_sp.	0.072	0.035	4.05.E-02	7.04.E-01
Autographiviridae	L7_Eubacterium_dolichum	0.050	0.045	2.67.E-01	9.01.E-01
crAss_like_phage	L7_Eubacterium_dolichum	-0.034	0.050	5.03.E-01	9.55.E-01

Herelleviridae	L7_Eubacterium_dolichum	-0.003	0.047	9.45.E-01	9.98.E-01
Microviridae	L7_Eubacterium_dolichum	0.002	0.047	9.70.E-01	9.99.E-01
Myoviridae	L7_Eubacterium_dolichum	0.003	0.053	9.61.E-01	9.98.E-01
Phycodnaviridae	L7_Eubacterium_dolichum	-0.038	0.047	4.21.E-01	9.20.E-01
Podoviridae	L7_Eubacterium_dolichum	-0.112	0.054	3.94.E-02	7.03.E-01
Siphoviridae	L7_Eubacterium_dolichum	-0.013	0.049	7.90.E-01	9.92.E-01
Autographiviridae	L7_Megamonas_rupellensis	-0.044	0.038	2.43.E-01	8.87.E-01
crAss_like_phage	L7_Megamonas_rupellensis	-0.002	0.042	9.66.E-01	9.98.E-01
Herelleviridae	L7_Megamonas_rupellensis	-0.009	0.039	8.25.E-01	9.93.E-01
Microviridae	L7_Megamonas_rupellensis	-0.019	0.039	6.26.E-01	9.76.E-01
Myoviridae	L7_Megamonas_rupellensis	0.060	0.044	1.69.E-01	8.48.E-01
Phycodnaviridae	L7_Megamonas_rupellensis	-0.016	0.039	6.81.E-01	9.80.E-01
Podoviridae	L7_Megamonas_rupellensis	-0.017	0.045	7.07.E-01	9.83.E-01
Siphoviridae	L7_Megamonas_rupellensis	-0.032	0.040	4.25.E-01	9.23.E-01
Autographiviridae	L7_Agathobaculum_desmolans	-0.067	0.040	9.57.E-02	7.66.E-01
crAss_like_phage	L7_Agathobaculum_desmolans	0.021	0.045	6.41.E-01	9.79.E-01
Herelleviridae	L7_Agathobaculum_desmolans	-0.023	0.042	5.86.E-01	9.65.E-01
Microviridae	L7_Agathobaculum_desmolans	-0.064	0.042	1.28.E-01	7.99.E-01
Myoviridae	L7_Agathobaculum_desmolans	0.018	0.047	7.05.E-01	9.82.E-01
Phycodnaviridae	L7_Agathobaculum_desmolans	-0.003	0.042	9.35.E-01	9.98.E-01
Podoviridae	L7_Agathobaculum_desmolans	0.034	0.049	4.85.E-01	9.54.E-01
Siphoviridae	L7_Agathobaculum_desmolans	0.055	0.043	2.04.E-01	8.76.E-01
Autographiviridae	L7_Dialister_sp.	0.012	0.045	7.96.E-01	9.92.E-01
crAss_like_phage	L7_Dialister_sp.	-0.074	0.049	1.37.E-01	8.09.E-01
Herelleviridae	L7_Dialister_sp.	-0.054	0.047	2.48.E-01	8.94.E-01
Microviridae	L7_Dialister_sp.	0.021	0.047	6.48.E-01	9.79.E-01
Myoviridae	L7_Dialister_sp.	-0.065	0.052	2.10.E-01	8.76.E-01
Phycodnaviridae	L7_Dialister_sp.	-0.028	0.046	5.46.E-01	9.60.E-01
Podoviridae	L7_Dialister_sp.	-0.093	0.054	8.42.E-02	7.50.E-01
Siphoviridae	L7_Dialister_sp.	-0.043	0.048	3.66.E-01	9.15.E-01
Autographiviridae	L7_Clostridium_hathewayi	-0.034	0.037	3.55.E-01	9.15.E-01
crAss_like_phage	L7_Clostridium_hathewayi	-0.028	0.041	5.01.E-01	9.55.E-01
Herelleviridae	L7_Clostridium_hathewayi	-0.007	0.038	8.56.E-01	9.96.E-01
Microviridae	L7_Clostridium_hathewayi	0.068	0.038	7.76.E-02	7.42.E-01
Myoviridae	L7_Clostridium_hathewayi	0.017	0.043	6.94.E-01	9.81.E-01
Phycodnaviridae	L7_Clostridium_hathewayi	-0.024	0.038	5.24.E-01	9.57.E-01
Podoviridae	L7_Clostridium_hathewayi	-0.021	0.044	6.34.E-01	9.76.E-01
Siphoviridae	L7_Clostridium_hathewayi	-0.016	0.040	6.80.E-01	9.80.E-01
Autographiviridae	L7_Bacteroides_eggerthii	0.043	0.033	2.00.E-01	8.74.E-01
crAss_like_phage	L7_Bacteroides_eggerthii	0.071	0.037	5.46.E-02	7.23.E-01
Herelleviridae	L7_Bacteroides_eggerthii	-0.003	0.035	9.41.E-01	9.98.E-01
Microviridae	L7_Bacteroides_eggerthii	-0.008	0.035	8.10.E-01	9.93.E-01
Myoviridae	L7_Bacteroides_eggerthii	-0.011	0.039	7.84.E-01	9.92.E-01
Phycodnaviridae	L7_Bacteroides_eggerthii	0.022	0.034	5.32.E-01	9.57.E-01
Podoviridae	L7_Bacteroides_eggerthii	-0.042	0.040	2.99.E-01	9.15.E-01
Siphoviridae	L7_Bacteroides_eggerthii	-0.018	0.036	6.18.E-01	9.75.E-01
Autographiviridae	L7_Fenollaria_massiliensis	0.031	0.042	4.61.E-01	9.43.E-01
crAss_like_phage	L7_Fenollaria_massiliensis	-0.002	0.046	9.63.E-01	9.98.E-01
Herelleviridae	L7_Fenollaria_massiliensis	0.084	0.043	5.12.E-02	7.22.E-01
Microviridae	L7_Fenollaria_massiliensis	0.021	0.043	6.28.E-01	9.76.E-01
Myoviridae	L7_Fenollaria_massiliensis	-0.050	0.048	2.96.E-01	9.15.E-01
Phycodnaviridae	L7_Fenollaria_massiliensis	-0.041	0.043	3.40.E-01	9.15.E-01
Podoviridae	L7_Fenollaria_massiliensis	-0.013	0.050	7.95.E-01	9.92.E-01
Siphoviridae	L7_Fenollaria_massiliensis	0.031	0.044	4.82.E-01	9.53.E-01
Autographiviridae	L7_Clostridium_phoceensis	0.051	0.032	1.20.E-01	7.91.E-01
crAss_like_phage	L7_Clostridium_phoceensis	0.036	0.036	3.16.E-01	9.15.E-01
Herelleviridae	L7_Clostridium_phoceensis	-0.010	0.034	7.65.E-01	9.91.E-01
Microviridae	L7_Clostridium_phoceensis	0.039	0.034	2.54.E-01	8.95.E-01
Myoviridae	L7_Clostridium_phoceensis	0.018	0.038	6.30.E-01	9.76.E-01
Phycodnaviridae	L7_Clostridium_phoceensis	0.039	0.033	2.39.E-01	8.85.E-01
Podoviridae	L7_Clostridium_phoceensis	0.139	0.038	3.20.E-04	2.06.E-01
Siphoviridae	L7_Clostridium_phoceensis	0.031	0.035	3.71.E-01	9.15.E-01
Autographiviridae	L7_Streptococcus_constellatus	-0.082	0.036	2.30.E-02	6.20.E-01
crAss_like_phage	L7_Streptococcus_constellatus	-0.036	0.040	3.63.E-01	9.15.E-01
Herelleviridae	L7_Streptococcus_constellatus	0.054	0.037	1.46.E-01	8.15.E-01

Microviridae	L7_Streptococcus_constellatus	0.072	0.037	5.42.E-02	7.23.E-01
Myoviridae	L7_Streptococcus_constellatus	0.042	0.042	3.20.E-01	9.15.E-01
Phycodnaviridae	L7_Streptococcus_constellatus	0.049	0.037	1.80.E-01	8.60.E-01
Podoviridae	L7_Streptococcus_constellatus	0.070	0.043	1.02.E-01	7.81.E-01
Siphoviridae	L7_Streptococcus_constellatus	0.022	0.038	5.70.E-01	9.61.E-01
Autographiviridae	L7_Ruminococcus_sp.	0.062	0.032	5.48.E-02	7.23.E-01
crAss_like_phage	L7_Ruminococcus_sp.	0.004	0.036	9.11.E-01	9.98.E-01
Herelleviridae	L7_Ruminococcus_sp.	0.001	0.034	9.68.E-01	9.98.E-01
Microviridae	L7_Ruminococcus_sp.	-0.037	0.034	2.75.E-01	9.05.E-01
Myoviridae	L7_Ruminococcus_sp.	-0.050	0.037	1.83.E-01	8.60.E-01
Phycodnaviridae	L7_Ruminococcus_sp.	0.000	0.033	9.92.E-01	9.99.E-01
Podoviridae	L7_Ruminococcus_sp.	-0.070	0.039	6.87.E-02	7.24.E-01
Siphoviridae	L7_Ruminococcus_sp.	-0.057	0.034	1.00.E-01	7.79.E-01
Autographiviridae	L7_Bacteroides_faecichinchillae	-0.025	0.028	3.76.E-01	9.15.E-01
crAss_like_phage	L7_Bacteroides_faecichinchillae	-0.023	0.031	4.60.E-01	9.43.E-01
Herelleviridae	L7_Bacteroides_faecichinchillae	-0.049	0.029	9.67.E-02	7.71.E-01
Microviridae	L7_Bacteroides_faecichinchillae	0.001	0.029	9.60.E-01	9.98.E-01
Myoviridae	L7_Bacteroides_faecichinchillae	-0.007	0.033	8.42.E-01	9.95.E-01
Phycodnaviridae	L7_Bacteroides_faecichinchillae	-0.061	0.029	3.59.E-02	6.90.E-01
Podoviridae	L7_Bacteroides_faecichinchillae	0.000	0.034	9.92.E-01	9.99.E-01
Siphoviridae	L7_Bacteroides_faecichinchillae	-0.001	0.030	9.66.E-01	9.98.E-01
Autographiviridae	L7_Clostridium_aldenense	0.033	0.037	3.63.E-01	9.15.E-01
crAss_like_phage	L7_Clostridium_aldenense	0.017	0.040	6.71.E-01	9.80.E-01
Herelleviridae	L7_Clostridium_aldenense	-0.042	0.038	2.67.E-01	9.01.E-01
Microviridae	L7_Clostridium_aldenense	-0.022	0.038	5.67.E-01	9.61.E-01
Myoviridae	L7_Clostridium_aldenense	0.010	0.043	8.22.E-01	9.93.E-01
Phycodnaviridae	L7_Clostridium_aldenense	-0.036	0.038	3.40.E-01	9.15.E-01
Podoviridae	L7_Clostridium_aldenense	0.011	0.044	7.98.E-01	9.92.E-01
Siphoviridae	L7_Clostridium_aldenense	-0.077	0.039	4.82.E-02	7.22.E-01
Autographiviridae	L7_Anaerostipes_sp.	-0.089	0.034	8.99.E-03	4.93.E-01
crAss_like_phage	L7_Anaerostipes_sp.	-0.060	0.037	1.09.E-01	7.82.E-01
Herelleviridae	L7_Anaerostipes_sp.	-0.062	0.035	7.76.E-02	7.42.E-01
Microviridae	L7_Anaerostipes_sp.	-0.060	0.035	8.96.E-02	7.58.E-01
Myoviridae	L7_Anaerostipes_sp.	-0.033	0.039	4.00.E-01	9.15.E-01
Phycodnaviridae	L7_Anaerostipes_sp.	-0.006	0.035	8.58.E-01	9.96.E-01
Podoviridae	L7_Anaerostipes_sp.	-0.092	0.041	2.42.E-02	6.37.E-01
Siphoviridae	L7_Anaerostipes_sp.	-0.035	0.036	3.32.E-01	9.15.E-01
Autographiviridae	L7_Prevotella_disiens	0.020	0.028	4.79.E-01	9.53.E-01
crAss_like_phage	L7_Prevotella_disiens	-0.033	0.031	2.86.E-01	9.15.E-01
Herelleviridae	L7_Prevotella_disiens	0.037	0.029	2.02.E-01	8.74.E-01
Microviridae	L7_Prevotella_disiens	-0.007	0.029	8.17.E-01	9.93.E-01
Myoviridae	L7_Prevotella_disiens	-0.015	0.033	6.49.E-01	9.79.E-01
Phycodnaviridae	L7_Prevotella_disiens	0.022	0.029	4.44.E-01	9.33.E-01
Podoviridae	L7_Prevotella_disiens	-0.019	0.034	5.74.E-01	9.62.E-01
Siphoviridae	L7_Prevotella_disiens	-0.013	0.030	6.56.E-01	9.79.E-01
Autographiviridae	L7_Lactobacillus_antri	-0.024	0.030	4.25.E-01	9.23.E-01
crAss_like_phage	L7_Lactobacillus_antri	0.024	0.033	4.69.E-01	9.50.E-01
Herelleviridae	L7_Lactobacillus_antri	0.008	0.031	7.98.E-01	9.92.E-01
Microviridae	L7_Lactobacillus_antri	0.002	0.032	9.53.E-01	9.98.E-01
Myoviridae	L7_Lactobacillus_antri	-0.038	0.035	2.75.E-01	9.05.E-01
Phycodnaviridae	L7_Lactobacillus_antri	0.032	0.031	3.03.E-01	9.15.E-01
Podoviridae	L7_Lactobacillus_antri	-0.042	0.036	2.43.E-01	8.87.E-01
Siphoviridae	L7_Lactobacillus_antri	-0.021	0.032	5.20.E-01	9.57.E-01
Autographiviridae	L7_Streptococcus_pyogenes	0.011	0.034	7.49.E-01	9.89.E-01
crAss_like_phage	L7_Streptococcus_pyogenes	0.005	0.038	9.01.E-01	9.97.E-01
Herelleviridae	L7_Streptococcus_pyogenes	-0.037	0.036	3.00.E-01	9.15.E-01
Microviridae	L7_Streptococcus_pyogenes	0.049	0.036	1.71.E-01	8.50.E-01
Myoviridae	L7_Streptococcus_pyogenes	-0.035	0.040	3.75.E-01	9.15.E-01
Phycodnaviridae	L7_Streptococcus_pyogenes	-0.003	0.035	9.43.E-01	9.98.E-01
Podoviridae	L7_Streptococcus_pyogenes	-0.047	0.041	2.54.E-01	8.95.E-01
Siphoviridae	L7_Streptococcus_pyogenes	-0.028	0.037	4.53.E-01	9.38.E-01
Autographiviridae	L7_Flavonifractor_plautii	0.050	0.031	1.14.E-01	7.85.E-01
crAss_like_phage	L7_Flavonifractor_plautii	-0.073	0.035	3.58.E-02	6.90.E-01
Herelleviridae	L7_Flavonifractor_plautii	-0.076	0.032	1.92.E-02	5.90.E-01
Microviridae	L7_Flavonifractor_plautii	-0.027	0.033	4.17.E-01	9.19.E-01

Myoviridae	L7_Flavonifractor_plautii	-0.066	0.036	6.85.E-02	7.24.E-01
Phycodnaviridae	L7_Flavonifractor_plautii	-0.032	0.032	3.20.E-01	9.15.E-01
Podoviridae	L7_Flavonifractor_plautii	-0.040	0.038	2.86.E-01	9.15.E-01
Siphoviridae	L7_Flavonifractor_plautii	-0.059	0.033	7.95.E-02	7.43.E-01
Autographiviridae	L7_Clostridium_clostridioforme	-0.016	0.031	5.94.E-01	9.69.E-01
crAss_like_phage	L7_Clostridium_clostridioforme	-0.002	0.034	9.63.E-01	9.98.E-01
Herelleviridae	L7_Clostridium_clostridioforme	0.025	0.032	4.35.E-01	9.26.E-01
Microviridae	L7_Clostridium_clostridioforme	0.020	0.032	5.21.E-01	9.57.E-01
Myoviridae	L7_Clostridium_clostridioforme	-0.033	0.036	3.50.E-01	9.15.E-01
Phycodnaviridae	L7_Clostridium_clostridioforme	0.051	0.031	1.03.E-01	7.82.E-01
Podoviridae	L7_Clostridium_clostridioforme	0.012	0.037	7.39.E-01	9.88.E-01
Siphoviridae	L7_Clostridium_clostridioforme	0.029	0.033	3.79.E-01	9.15.E-01
Autographiviridae	L7_Bacteroides_xylanisolvens	-0.012	0.029	6.71.E-01	9.80.E-01
crAss_like_phage	L7_Bacteroides_xylanisolvens	-0.029	0.032	3.66.E-01	9.15.E-01
Herelleviridae	L7_Bacteroides_xylanisolvens	0.011	0.030	7.12.E-01	9.85.E-01
Microviridae	L7_Bacteroides_xylanisolvens	0.004	0.030	9.01.E-01	9.97.E-01
Myoviridae	L7_Bacteroides_xylanisolvens	-0.029	0.034	3.96.E-01	9.15.E-01
Phycodnaviridae	L7_Bacteroides_xylanisolvens	0.051	0.030	8.52.E-02	7.50.E-01
Podoviridae	L7_Bacteroides_xylanisolvens	0.016	0.035	6.47.E-01	9.79.E-01
Siphoviridae	L7_Bacteroides_xylanisolvens	0.025	0.031	4.29.E-01	9.25.E-01
Autographiviridae	L7_Lactobacillus_gasseri	-0.046	0.034	1.67.E-01	8.44.E-01
crAss_like_phage	L7_Lactobacillus_gasseri	-0.006	0.037	8.74.E-01	9.96.E-01
Herelleviridae	L7_Lactobacillus_gasseri	-0.002	0.035	9.61.E-01	9.98.E-01
Microviridae	L7_Lactobacillus_gasseri	-0.041	0.035	2.39.E-01	8.85.E-01
Myoviridae	L7_Lactobacillus_gasseri	0.003	0.039	9.41.E-01	9.98.E-01
Phycodnaviridae	L7_Lactobacillus_gasseri	0.028	0.034	4.24.E-01	9.23.E-01
Podoviridae	L7_Lactobacillus_gasseri	-0.017	0.040	6.77.E-01	9.80.E-01
Siphoviridae	L7_Lactobacillus_gasseri	-0.032	0.036	3.67.E-01	9.15.E-01
Autographiviridae	L7_Streptococcus_oralis	-0.009	0.022	6.80.E-01	9.80.E-01
crAss_like_phage	L7_Streptococcus_oralis	0.012	0.025	6.22.E-01	9.76.E-01
Herelleviridae	L7_Streptococcus_oralis	-0.016	0.023	5.05.E-01	9.57.E-01
Microviridae	L7_Streptococcus_oralis	0.010	0.023	6.82.E-01	9.80.E-01
Myoviridae	L7_Streptococcus_oralis	0.008	0.026	7.67.E-01	9.91.E-01
Phycodnaviridae	L7_Streptococcus_oralis	-0.001	0.023	9.53.E-01	9.98.E-01
Podoviridae	L7_Streptococcus_oralis	0.041	0.027	1.26.E-01	7.98.E-01
Siphoviridae	L7_Streptococcus_oralis	0.001	0.024	9.55.E-01	9.98.E-01
Autographiviridae	L7_Lactobacillus_salivarius	-0.008	0.032	8.11.E-01	9.93.E-01
crAss_like_phage	L7_Lactobacillus_salivarius	-0.003	0.035	9.37.E-01	9.98.E-01
Herelleviridae	L7_Lactobacillus_salivarius	0.033	0.033	3.12.E-01	9.15.E-01
Microviridae	L7_Lactobacillus_salivarius	0.005	0.033	8.84.E-01	9.96.E-01
Myoviridae	L7_Lactobacillus_salivarius	-0.043	0.037	2.41.E-01	8.85.E-01
Phycodnaviridae	L7_Lactobacillus_salivarius	-0.010	0.033	7.70.E-01	9.91.E-01
Podoviridae	L7_Lactobacillus_salivarius	-0.045	0.038	2.34.E-01	8.85.E-01
Siphoviridae	L7_Lactobacillus_salivarius	0.024	0.034	4.70.E-01	9.50.E-01
Autographiviridae	L7_Catonella_morbi	0.023	0.043	5.91.E-01	9.68.E-01
crAss_like_phage	L7_Catonella_morbi	-0.060	0.048	2.09.E-01	8.76.E-01
Herelleviridae	L7_Catonella_morbi	-0.014	0.045	7.54.E-01	9.89.E-01
Microviridae	L7_Catonella_morbi	0.004	0.045	9.29.E-01	9.98.E-01
Myoviridae	L7_Catonella_morbi	0.060	0.050	2.29.E-01	8.82.E-01
Phycodnaviridae	L7_Catonella_morbi	0.033	0.044	4.60.E-01	9.43.E-01
Podoviridae	L7_Catonella_morbi	0.149	0.051	3.93.E-03	4.05.E-01
Siphoviridae	L7_Catonella_morbi	0.055	0.046	2.32.E-01	8.82.E-01
Autographiviridae	L7_Alistipes_putredinis	-0.028	0.034	4.10.E-01	9.17.E-01
crAss_like_phage	L7_Alistipes_putredinis	-0.042	0.038	2.64.E-01	9.01.E-01
Herelleviridae	L7_Alistipes_putredinis	0.014	0.036	6.86.E-01	9.80.E-01
Microviridae	L7_Alistipes_putredinis	0.016	0.036	6.56.E-01	9.79.E-01
Myoviridae	L7_Alistipes_putredinis	-0.012	0.040	7.62.E-01	9.91.E-01
Phycodnaviridae	L7_Alistipes_putredinis	0.019	0.035	5.96.E-01	9.69.E-01
Podoviridae	L7_Alistipes_putredinis	0.042	0.041	3.04.E-01	9.15.E-01
Siphoviridae	L7_Alistipes_putredinis	0.008	0.037	8.19.E-01	9.93.E-01
Autographiviridae	L7_Phascolarctobacterium_sp.	0.043	0.042	3.13.E-01	9.15.E-01
crAss_like_phage	L7_Phascolarctobacterium_sp.	0.039	0.047	4.10.E-01	9.17.E-01
Herelleviridae	L7_Phascolarctobacterium_sp.	0.055	0.044	2.11.E-01	8.78.E-01
Microviridae	L7_Phascolarctobacterium_sp.	0.027	0.044	5.41.E-01	9.60.E-01
Myoviridae	L7_Phascolarctobacterium_sp.	0.132	0.049	7.03.E-03	4.53.E-01

Phycodnaviridae	L7_Phascolarctobacterium_sp.	0.020	0.044	6.48.E-01	9.79.E-01
Podoviridae	L7_Phascolarctobacterium_sp.	0.098	0.051	5.29.E-02	7.22.E-01
Siphoviridae	L7_Phascolarctobacterium_sp.	0.052	0.045	2.47.E-01	8.92.E-01
Autographiviridae	L7_Methanobrevibacter_smithii	0.045	0.036	2.08.E-01	8.76.E-01
crAss_like_phage	L7_Methanobrevibacter_smithii	-0.029	0.040	4.62.E-01	9.44.E-01
Herelleviridae	L7_Methanobrevibacter_smithii	0.070	0.037	6.23.E-02	7.24.E-01
Microviridae	L7_Methanobrevibacter_smithii	0.041	0.038	2.81.E-01	9.12.E-01
Myoviridae	L7_Methanobrevibacter_smithii	0.011	0.042	8.01.E-01	9.92.E-01
Phycodnaviridae	L7_Methanobrevibacter_smithii	-0.025	0.037	4.98.E-01	9.55.E-01
Podoviridae	L7_Methanobrevibacter_smithii	-0.024	0.043	5.73.E-01	9.61.E-01
Siphoviridae	L7_Methanobrevibacter_smithii	-0.018	0.039	6.33.E-01	9.76.E-01
Autographiviridae	L7_Desulfovibrio_sp.	-0.009	0.037	8.02.E-01	9.92.E-01
crAss_like_phage	L7_Desulfovibrio_sp.	0.011	0.040	7.79.E-01	9.92.E-01
Herelleviridae	L7_Desulfovibrio_sp.	0.035	0.038	3.63.E-01	9.15.E-01
Microviridae	L7_Desulfovibrio_sp.	0.008	0.038	8.39.E-01	9.94.E-01
Myoviridae	L7_Desulfovibrio_sp.	-0.015	0.042	7.21.E-01	9.86.E-01
Phycodnaviridae	L7_Desulfovibrio_sp.	-0.004	0.038	9.18.E-01	9.98.E-01
Podoviridae	L7_Desulfovibrio_sp.	-0.039	0.044	3.69.E-01	9.15.E-01
Siphoviridae	L7_Desulfovibrio_sp.	-0.044	0.039	2.59.E-01	8.95.E-01
Autographiviridae	L7_Fusicatenibacter_sp.	0.021	0.038	5.79.E-01	9.64.E-01
crAss_like_phage	L7_Fusicatenibacter_sp.	0.036	0.042	3.90.E-01	9.15.E-01
Herelleviridae	L7_Fusicatenibacter_sp.	0.034	0.039	3.82.E-01	9.15.E-01
Microviridae	L7_Fusicatenibacter_sp.	-0.024	0.040	5.47.E-01	9.60.E-01
Myoviridae	L7_Fusicatenibacter_sp.	-0.036	0.044	4.18.E-01	9.19.E-01
Phycodnaviridae	L7_Fusicatenibacter_sp.	0.033	0.039	4.01.E-01	9.15.E-01
Podoviridae	L7_Fusicatenibacter_sp.	-0.022	0.046	6.26.E-01	9.76.E-01
Siphoviridae	L7_Fusicatenibacter_sp.	-0.010	0.041	8.12.E-01	9.93.E-01
Autographiviridae	L7_Collinsella_tanakaei	-0.003	0.038	9.27.E-01	9.98.E-01
crAss_like_phage	L7_Collinsella_tanakaei	-0.052	0.042	2.17.E-01	8.79.E-01
Herelleviridae	L7_Collinsella_tanakaei	0.013	0.040	7.50.E-01	9.89.E-01
Microviridae	L7_Collinsella_tanakaei	0.019	0.040	6.26.E-01	9.76.E-01
Myoviridae	L7_Collinsella_tanakaei	-0.050	0.044	2.62.E-01	9.00.E-01
Phycodnaviridae	L7_Collinsella_tanakaei	-0.019	0.039	6.38.E-01	9.78.E-01
Podoviridae	L7_Collinsella_tanakaei	-0.028	0.046	5.46.E-01	9.60.E-01
Siphoviridae	L7_Collinsella_tanakaei	-0.005	0.041	9.07.E-01	9.97.E-01
Autographiviridae	L7_Prevotella_buccalis	0.008	0.032	8.00.E-01	9.92.E-01
crAss_like_phage	L7_Prevotella_buccalis	0.049	0.035	1.60.E-01	8.38.E-01
Herelleviridae	L7_Prevotella_buccalis	-0.008	0.033	7.99.E-01	9.92.E-01
Microviridae	L7_Prevotella_buccalis	-0.019	0.033	5.58.E-01	9.61.E-01
Myoviridae	L7_Prevotella_buccalis	0.016	0.037	6.53.E-01	9.79.E-01
Phycodnaviridae	L7_Prevotella_buccalis	0.003	0.032	9.31.E-01	9.98.E-01
Podoviridae	L7_Prevotella_buccalis	0.004	0.038	9.25.E-01	9.98.E-01
Siphoviridae	L7_Prevotella_buccalis	-0.012	0.034	7.31.E-01	9.88.E-01
Autographiviridae	L7_Lactobacillus_paracasei	0.024	0.037	5.15.E-01	9.57.E-01
crAss_like_phage	L7_Lactobacillus_paracasei	0.014	0.041	7.21.E-01	9.86.E-01
Herelleviridae	L7_Lactobacillus_paracasei	0.043	0.038	2.57.E-01	8.95.E-01
Microviridae	L7_Lactobacillus_paracasei	0.004	0.038	9.18.E-01	9.98.E-01
Myoviridae	L7_Lactobacillus_paracasei	-0.011	0.043	8.05.E-01	9.93.E-01
Phycodnaviridae	L7_Lactobacillus_paracasei	0.046	0.038	2.23.E-01	8.80.E-01
Podoviridae	L7_Lactobacillus_paracasei	-0.043	0.044	3.28.E-01	9.15.E-01
Siphoviridae	L7_Lactobacillus_paracasei	0.031	0.039	4.32.E-01	9.25.E-01
Autographiviridae	L7_Bifidobacterium_longum	0.012	0.032	7.11.E-01	9.85.E-01
crAss_like_phage	L7_Bifidobacterium_longum	0.011	0.035	7.57.E-01	9.90.E-01
Herelleviridae	L7_Bifidobacterium_longum	-0.002	0.033	9.49.E-01	9.98.E-01
Microviridae	L7_Bifidobacterium_longum	0.090	0.033	6.70.E-03	4.51.E-01
Myoviridae	L7_Bifidobacterium_longum	0.016	0.037	6.68.E-01	9.80.E-01
Phycodnaviridae	L7_Bifidobacterium_longum	0.015	0.033	6.49.E-01	9.79.E-01
Podoviridae	L7_Bifidobacterium_longum	0.049	0.038	2.01.E-01	8.74.E-01
Siphoviridae	L7_Bifidobacterium_longum	0.042	0.034	2.23.E-01	8.81.E-01
Autographiviridae	L7_Harryflintia_acetispora	0.000	0.025	9.97.E-01	9.99.E-01
crAss_like_phage	L7_Harryflintia_acetispora	0.004	0.028	8.85.E-01	9.96.E-01
Herelleviridae	L7_Harryflintia_acetispora	-0.024	0.026	3.52.E-01	9.15.E-01
Microviridae	L7_Harryflintia_acetispora	0.016	0.026	5.30.E-01	9.57.E-01
Myoviridae	L7_Harryflintia_acetispora	0.031	0.029	2.92.E-01	9.15.E-01
Phycodnaviridae	L7_Harryflintia_acetispora	-0.034	0.026	1.83.E-01	8.60.E-01

Podoviridae	L7_Harryflintia_acetispora	-0.031	0.030	3.02.E-01	9.15.E-01
Siphoviridae	L7_Harryflintia_acetispora	-0.031	0.027	2.41.E-01	8.85.E-01
Autographiviridae	L7_Bacteroidetes_bacterium	-0.045	0.040	2.69.E-01	9.01.E-01
crAss_like_phage	L7_Bacteroidetes_bacterium	-0.012	0.045	7.81.E-01	9.92.E-01
Herelleviridae	L7_Bacteroidetes_bacterium	0.018	0.042	6.59.E-01	9.79.E-01
Microviridae	L7_Bacteroidetes_bacterium	0.047	0.042	2.60.E-01	8.95.E-01
Myoviridae	L7_Bacteroidetes_bacterium	-0.032	0.047	4.99.E-01	9.55.E-01
Phycodnaviridae	L7_Bacteroidetes_bacterium	0.046	0.041	2.65.E-01	9.01.E-01
Podoviridae	L7_Bacteroidetes_bacterium	0.053	0.048	2.72.E-01	9.02.E-01
Siphoviridae	L7_Bacteroidetes_bacterium	0.036	0.043	4.08.E-01	9.17.E-01
Autographiviridae	L7_butyrate.producing_bacterium	-0.030	0.035	3.89.E-01	9.15.E-01
crAss_like_phage	L7_butyrate.producing_bacterium	-0.035	0.039	3.68.E-01	9.15.E-01
Herelleviridae	L7_butyrate.producing_bacterium	-0.051	0.036	1.63.E-01	8.38.E-01
Microviridae	L7_butyrate.producing_bacterium	-0.042	0.037	2.54.E-01	8.95.E-01
Myoviridae	L7_butyrate.producing_bacterium	0.008	0.041	8.49.E-01	9.95.E-01
Phycodnaviridae	L7_butyrate.producing_bacterium	-0.023	0.036	5.27.E-01	9.57.E-01
Podoviridae	L7_butyrate.producing_bacterium	0.010	0.042	8.11.E-01	9.93.E-01
Siphoviridae	L7_butyrate.producing_bacterium	-0.016	0.038	6.71.E-01	9.80.E-01
Autographiviridae	L7_Fusobacterium_varium	-0.053	0.039	1.77.E-01	8.58.E-01
crAss_like_phage	L7_Fusobacterium_varium	-0.040	0.043	3.58.E-01	9.15.E-01
Herelleviridae	L7_Fusobacterium_varium	0.009	0.041	8.28.E-01	9.94.E-01
Microviridae	L7_Fusobacterium_varium	-0.075	0.041	6.56.E-02	7.24.E-01
Myoviridae	L7_Fusobacterium_varium	-0.018	0.045	6.97.E-01	9.81.E-01
Phycodnaviridae	L7_Fusobacterium_varium	0.020	0.040	6.23.E-01	9.76.E-01
Podoviridae	L7_Fusobacterium_varium	0.090	0.047	5.51.E-02	7.24.E-01
Siphoviridae	L7_Fusobacterium_varium	-0.034	0.042	4.18.E-01	9.19.E-01
Autographiviridae	L7_Tyzzerella_nexilis	-0.015	0.037	6.76.E-01	9.80.E-01
crAss_like_phage	L7_Tyzzerella_nexilis	-0.006	0.041	8.89.E-01	9.96.E-01
Herelleviridae	L7_Tyzzerella_nexilis	0.071	0.038	6.23.E-02	7.24.E-01
Microviridae	L7_Tyzzerella_nexilis	0.004	0.038	9.09.E-01	9.98.E-01
Myoviridae	L7_Tyzzerella_nexilis	-0.007	0.043	8.67.E-01	9.96.E-01
Phycodnaviridae	L7_Tyzzerella_nexilis	-0.030	0.038	4.31.E-01	9.25.E-01
Podoviridae	L7_Tyzzerella_nexilis	0.024	0.044	5.94.E-01	9.69.E-01
Siphoviridae	L7_Tyzzerella_nexilis	0.018	0.039	6.53.E-01	9.79.E-01
Autographiviridae	L7_Alistipes_timonensis	0.010	0.031	7.53.E-01	9.89.E-01
crAss_like_phage	L7_Alistipes_timonensis	0.020	0.034	5.51.E-01	9.60.E-01
Herelleviridae	L7_Alistipes_timonensis	0.048	0.032	1.40.E-01	8.10.E-01
Microviridae	L7_Alistipes_timonensis	0.021	0.032	5.16.E-01	9.57.E-01
Myoviridae	L7_Alistipes_timonensis	-0.083	0.036	2.12.E-02	6.06.E-01
Phycodnaviridae	L7_Alistipes_timonensis	-0.019	0.032	5.51.E-01	9.60.E-01
Podoviridae	L7_Alistipes_timonensis	0.080	0.037	3.06.E-02	6.69.E-01
Siphoviridae	L7_Alistipes_timonensis	0.024	0.033	4.73.E-01	9.52.E-01
Autographiviridae	L7_Weissella_confusa	-0.040	0.041	3.34.E-01	9.15.E-01
crAss_like_phage	L7_Weissella_confusa	-0.036	0.046	4.29.E-01	9.25.E-01
Herelleviridae	L7_Weissella_confusa	0.040	0.043	3.55.E-01	9.15.E-01
Microviridae	L7_Weissella_confusa	0.045	0.043	2.93.E-01	9.15.E-01
Myoviridae	L7_Weissella_confusa	-0.108	0.048	2.51.E-02	6.49.E-01
Phycodnaviridae	L7_Weissella_confusa	-0.066	0.043	1.20.E-01	7.91.E-01
Podoviridae	L7_Weissella_confusa	-0.009	0.050	8.54.E-01	9.95.E-01
Siphoviridae	L7_Weissella_confusa	-0.068	0.044	1.27.E-01	7.98.E-01
Autographiviridae	L7_Lactobacillus_oris	-0.031	0.032	3.29.E-01	9.15.E-01
crAss_like_phage	L7_Lactobacillus_oris	0.015	0.036	6.68.E-01	9.80.E-01
Herelleviridae	L7_Lactobacillus_oris	0.017	0.033	6.07.E-01	9.74.E-01
Microviridae	L7_Lactobacillus_oris	-0.009	0.034	7.86.E-01	9.92.E-01
Myoviridae	L7_Lactobacillus_oris	-0.051	0.037	1.70.E-01	8.49.E-01
Phycodnaviridae	L7_Lactobacillus_oris	0.030	0.033	3.68.E-01	9.15.E-01
Podoviridae	L7_Lactobacillus_oris	-0.029	0.039	4.56.E-01	9.40.E-01
Siphoviridae	L7_Lactobacillus_oris	-0.021	0.034	5.37.E-01	9.60.E-01
Autographiviridae	L7_Citrobacter_freundii	-0.058	0.027	3.27.E-02	6.75.E-01
crAss_like_phage	L7_Citrobacter_freundii	0.022	0.030	4.56.E-01	9.39.E-01
Herelleviridae	L7_Citrobacter_freundii	0.023	0.028	4.08.E-01	9.17.E-01
Microviridae	L7_Citrobacter_freundii	-0.021	0.028	4.48.E-01	9.35.E-01
Myoviridae	L7_Citrobacter_freundii	-0.034	0.031	2.87.E-01	9.15.E-01
Phycodnaviridae	L7_Citrobacter_freundii	0.016	0.028	5.69.E-01	9.61.E-01
Podoviridae	L7_Citrobacter_freundii	0.008	0.033	8.16.E-01	9.93.E-01

Siphoviridae	L7_Citrobacter_freundii	0.016	0.029	5.88.E-01	9.68.E-01
Autographiviridae	L7_Atopobium_sp.	-0.034	0.034	3.20.E-01	9.15.E-01
crAss_like_phage	L7_Atopobium_sp.	0.036	0.038	3.47.E-01	9.15.E-01
Herelleviridae	L7_Atopobium_sp.	-0.028	0.036	4.33.E-01	9.26.E-01
Microviridae	L7_Atopobium_sp.	-0.040	0.036	2.60.E-01	8.96.E-01
Myoviridae	L7_Atopobium_sp.	-0.039	0.040	3.28.E-01	9.15.E-01
Phycodnaviridae	L7_Atopobium_sp.	0.014	0.035	6.82.E-01	9.80.E-01
Podoviridae	L7_Atopobium_sp.	0.034	0.041	4.07.E-01	9.17.E-01
Siphoviridae	L7_Atopobium_sp.	-0.071	0.037	5.27.E-02	7.22.E-01
Autographiviridae	L7_Citrobacter_sp.	-0.037	0.027	1.78.E-01	8.59.E-01
crAss_like_phage	L7_Citrobacter_sp.	0.023	0.030	4.58.E-01	9.42.E-01
Herelleviridae	L7_Citrobacter_sp.	-0.004	0.029	8.78.E-01	9.96.E-01
Microviridae	L7_Citrobacter_sp.	0.007	0.029	7.96.E-01	9.92.E-01
Myoviridae	L7_Citrobacter_sp.	-0.024	0.032	4.44.E-01	9.33.E-01
Phycodnaviridae	L7_Citrobacter_sp.	-0.030	0.028	2.92.E-01	9.15.E-01
Podoviridae	L7_Citrobacter_sp.	-0.004	0.033	8.97.E-01	9.97.E-01
Siphoviridae	L7_Citrobacter_sp.	0.026	0.029	3.84.E-01	9.15.E-01
Autographiviridae	L7_Bacteroides_clarus	-0.009	0.031	7.67.E-01	9.91.E-01
crAss_like_phage	L7_Bacteroides_clarus	-0.044	0.034	1.94.E-01	8.65.E-01
Herelleviridae	L7_Bacteroides_clarus	-0.055	0.032	8.79.E-02	7.54.E-01
Microviridae	L7_Bacteroides_clarus	0.009	0.032	7.73.E-01	9.92.E-01
Myoviridae	L7_Bacteroides_clarus	-0.023	0.036	5.20.E-01	9.57.E-01
Phycodnaviridae	L7_Bacteroides_clarus	0.023	0.032	4.76.E-01	9.52.E-01
Podoviridae	L7_Bacteroides_clarus	0.007	0.037	8.46.E-01	9.95.E-01
Siphoviridae	L7_Bacteroides_clarus	-0.042	0.033	2.10.E-01	8.76.E-01
Autographiviridae	L7_Brachyspira_sp.	-0.007	0.040	8.56.E-01	9.96.E-01
crAss_like_phage	L7_Brachyspira_sp.	-0.002	0.044	9.64.E-01	9.98.E-01
Herelleviridae	L7_Brachyspira_sp.	-0.023	0.041	5.80.E-01	9.64.E-01
Microviridae	L7_Brachyspira_sp.	0.041	0.041	3.20.E-01	9.15.E-01
Myoviridae	L7_Brachyspira_sp.	-0.012	0.046	8.00.E-01	9.92.E-01
Phycodnaviridae	L7_Brachyspira_sp.	0.015	0.041	7.05.E-01	9.82.E-01
Podoviridae	L7_Brachyspira_sp.	0.017	0.048	7.23.E-01	9.86.E-01
Siphoviridae	L7_Brachyspira_sp.	0.041	0.043	3.39.E-01	9.15.E-01
Autographiviridae	L7_Bifidobacterium_dentium	-0.012	0.038	7.47.E-01	9.89.E-01
crAss_like_phage	L7_Bifidobacterium_dentium	0.004	0.042	9.25.E-01	9.98.E-01
Herelleviridae	L7_Bifidobacterium_dentium	0.031	0.039	4.29.E-01	9.25.E-01
Microviridae	L7_Bifidobacterium_dentium	-0.037	0.039	3.44.E-01	9.15.E-01
Myoviridae	L7_Bifidobacterium_dentium	-0.056	0.044	1.99.E-01	8.70.E-01
Phycodnaviridae	L7_Bifidobacterium_dentium	0.041	0.039	2.90.E-01	9.15.E-01
Podoviridae	L7_Bifidobacterium_dentium	-0.028	0.045	5.35.E-01	9.59.E-01
Siphoviridae	L7_Bifidobacterium_dentium	-0.050	0.040	2.16.E-01	8.79.E-01
Autographiviridae	L7_Emergencia_timonensis	0.038	0.036	2.99.E-01	9.15.E-01
crAss_like_phage	L7_Emergencia_timonensis	0.010	0.040	7.96.E-01	9.92.E-01
Herelleviridae	L7_Emergencia_timonensis	-0.026	0.038	4.83.E-01	9.54.E-01
Microviridae	L7_Emergencia_timonensis	0.022	0.038	5.63.E-01	9.61.E-01
Myoviridae	L7_Emergencia_timonensis	0.024	0.042	5.61.E-01	9.61.E-01
Phycodnaviridae	L7_Emergencia_timonensis	0.028	0.037	4.59.E-01	9.43.E-01
Podoviridae	L7_Emergencia_timonensis	0.017	0.043	6.97.E-01	9.81.E-01
Siphoviridae	L7_Emergencia_timonensis	0.062	0.039	1.08.E-01	7.82.E-01
Autographiviridae	L7_Clostridiales_bacterium	0.022	0.027	4.20.E-01	9.20.E-01
crAss_like_phage	L7_Clostridiales_bacterium	0.027	0.030	3.59.E-01	9.15.E-01
Herelleviridae	L7_Clostridiales_bacterium	0.012	0.028	6.61.E-01	9.79.E-01
Microviridae	L7_Clostridiales_bacterium	-0.016	0.028	5.79.E-01	9.64.E-01
Myoviridae	L7_Clostridiales_bacterium	0.092	0.031	3.14.E-03	3.87.E-01
Phycodnaviridae	L7_Clostridiales_bacterium	-0.017	0.028	5.46.E-01	9.60.E-01
Podoviridae	L7_Clostridiales_bacterium	-0.081	0.032	1.20.E-02	5.36.E-01
Siphoviridae	L7_Clostridiales_bacterium	0.036	0.029	2.08.E-01	8.76.E-01
Autographiviridae	L7_Holdemanella_biformis	0.083	0.039	3.27.E-02	6.75.E-01
crAss_like_phage	L7_Holdemanella_biformis	0.042	0.043	3.33.E-01	9.15.E-01
Herelleviridae	L7_Holdemanella_biformis	0.008	0.040	8.34.E-01	9.94.E-01
Microviridae	L7_Holdemanella_biformis	0.068	0.040	9.10.E-02	7.59.E-01
Myoviridae	L7_Holdemanella_biformis	-0.029	0.045	5.14.E-01	9.57.E-01
Phycodnaviridae	L7_Holdemanella_biformis	-0.003	0.040	9.40.E-01	9.98.E-01
Podoviridae	L7_Holdemanella_biformis	-0.034	0.047	4.65.E-01	9.45.E-01
Siphoviridae	L7_Holdemanella_biformis	0.081	0.041	5.01.E-02	7.22.E-01

Autographiviridae	L7_Faecalicoccus_pleomorphus	-0.035	0.037	3.49.E-01	9.15.E-01
crAss_like_phage	L7_Faecalicoccus_pleomorphus	0.001	0.041	9.85.E-01	9.99.E-01
Herelleviridae	L7_Faecalicoccus_pleomorphus	-0.034	0.038	3.75.E-01	9.15.E-01
Microviridae	L7_Faecalicoccus_pleomorphus	-0.045	0.038	2.38.E-01	8.85.E-01
Myoviridae	L7_Faecalicoccus_pleomorphus	-0.057	0.043	1.85.E-01	8.61.E-01
Phycodnaviridae	L7_Faecalicoccus_pleomorphus	-0.037	0.038	3.29.E-01	9.15.E-01
Podoviridae	L7_Faecalicoccus_pleomorphus	-0.010	0.044	8.20.E-01	9.93.E-01
Siphoviridae	L7_Faecalicoccus_pleomorphus	-0.003	0.039	9.44.E-01	9.98.E-01
Autographiviridae	L7_Odoribacter_sp.	0.042	0.027	1.22.E-01	7.93.E-01
crAss_like_phage	L7_Odoribacter_sp.	0.026	0.030	4.00.E-01	9.15.E-01
Herelleviridae	L7_Odoribacter_sp.	0.004	0.029	8.90.E-01	9.96.E-01
Microviridae	L7_Odoribacter_sp.	-0.025	0.029	3.88.E-01	9.15.E-01
Myoviridae	L7_Odoribacter_sp.	-0.046	0.032	1.52.E-01	8.24.E-01
Phycodnaviridae	L7_Odoribacter_sp.	0.031	0.028	2.76.E-01	9.05.E-01
Podoviridae	L7_Odoribacter_sp.	-0.014	0.033	6.76.E-01	9.80.E-01
Siphoviridae	L7_Odoribacter_sp.	-0.019	0.029	5.21.E-01	9.57.E-01
Autographiviridae	L7_Blautia_hydrogenotrophica	-0.058	0.039	1.38.E-01	8.10.E-01
crAss_like_phage	L7_Blautia_hydrogenotrophica	0.020	0.043	6.38.E-01	9.78.E-01
Herelleviridae	L7_Blautia_hydrogenotrophica	-0.013	0.041	7.43.E-01	9.89.E-01
Microviridae	L7_Blautia_hydrogenotrophica	0.062	0.041	1.24.E-01	7.94.E-01
Myoviridae	L7_Blautia_hydrogenotrophica	0.031	0.045	4.99.E-01	9.55.E-01
Phycodnaviridae	L7_Blautia_hydrogenotrophica	0.008	0.040	8.39.E-01	9.94.E-01
Podoviridae	L7_Blautia_hydrogenotrophica	-0.047	0.047	3.17.E-01	9.15.E-01
Siphoviridae	L7_Blautia_hydrogenotrophica	-0.002	0.042	9.56.E-01	9.98.E-01
Autographiviridae	L7_Prevotella_stercorea	-0.025	0.034	4.70.E-01	9.50.E-01
crAss_like_phage	L7_Prevotella_stercorea	0.008	0.038	8.39.E-01	9.94.E-01
Herelleviridae	L7_Prevotella_stercorea	-0.015	0.036	6.70.E-01	9.80.E-01
Microviridae	L7_Prevotella_stercorea	0.005	0.036	8.86.E-01	9.96.E-01
Myoviridae	L7_Prevotella_stercorea	0.053	0.040	1.84.E-01	8.60.E-01
Phycodnaviridae	L7_Prevotella_stercorea	0.019	0.035	6.00.E-01	9.70.E-01
Podoviridae	L7_Prevotella_stercorea	0.063	0.041	1.25.E-01	7.94.E-01
Siphoviridae	L7_Prevotella_stercorea	0.079	0.037	3.07.E-02	6.69.E-01
Autographiviridae	L7_Eubacterium_hallii	-0.010	0.038	7.84.E-01	9.92.E-01
crAss_like_phage	L7_Eubacterium_hallii	-0.036	0.042	3.89.E-01	9.15.E-01
Herelleviridae	L7_Eubacterium_hallii	0.058	0.039	1.35.E-01	8.07.E-01
Microviridae	L7_Eubacterium_hallii	0.033	0.039	3.99.E-01	9.15.E-01
Myoviridae	L7_Eubacterium_hallii	0.021	0.044	6.32.E-01	9.76.E-01
Phycodnaviridae	L7_Eubacterium_hallii	0.024	0.039	5.29.E-01	9.57.E-01
Podoviridae	L7_Eubacterium_hallii	-0.023	0.045	6.11.E-01	9.75.E-01
Siphoviridae	L7_Eubacterium_hallii	0.000	0.040	9.98.E-01	9.99.E-01
Autographiviridae	L7_Senegalimassilia_anaerobia	-0.017	0.038	6.52.E-01	9.79.E-01
crAss_like_phage	L7_Senegalimassilia_anaerobia	-0.064	0.042	1.29.E-01	7.99.E-01
Herelleviridae	L7_Senegalimassilia_anaerobia	-0.057	0.039	1.52.E-01	8.24.E-01
Microviridae	L7_Senegalimassilia_anaerobia	0.035	0.040	3.78.E-01	9.15.E-01
Myoviridae	L7_Senegalimassilia_anaerobia	0.009	0.044	8.33.E-01	9.94.E-01
Phycodnaviridae	L7_Senegalimassilia_anaerobia	0.067	0.039	8.42.E-02	7.50.E-01
Podoviridae	L7_Senegalimassilia_anaerobia	-0.042	0.046	3.61.E-01	9.15.E-01
Siphoviridae	L7_Senegalimassilia_anaerobia	-0.019	0.041	6.35.E-01	9.76.E-01
Autographiviridae	L7_Drancourtella_massiliensis	0.070	0.036	5.73.E-02	7.24.E-01
crAss_like_phage	L7_Drancourtella_massiliensis	0.017	0.040	6.80.E-01	9.80.E-01
Herelleviridae	L7_Drancourtella_massiliensis	-0.002	0.038	9.61.E-01	9.98.E-01
Microviridae	L7_Drancourtella_massiliensis	0.016	0.038	6.83.E-01	9.80.E-01
Myoviridae	L7_Drancourtella_massiliensis	0.132	0.042	1.77.E-03	3.25.E-01
Phycodnaviridae	L7_Drancourtella_massiliensis	-0.036	0.038	3.38.E-01	9.15.E-01
Podoviridae	L7_Drancourtella_massiliensis	-0.028	0.044	5.30.E-01	9.57.E-01
Siphoviridae	L7_Drancourtella_massiliensis	0.012	0.039	7.60.E-01	9.91.E-01
Autographiviridae	L7_Streptococcus_pseudopneumoniae	-0.012	0.025	6.39.E-01	9.79.E-01
crAss_like_phage	L7_Streptococcus_pseudopneumoniae	0.001	0.028	9.64.E-01	9.98.E-01
Herelleviridae	L7_Streptococcus_pseudopneumoniae	-0.027	0.026	2.97.E-01	9.15.E-01
Microviridae	L7_Streptococcus_pseudopneumoniae	-0.052	0.026	4.89.E-02	7.22.E-01
Myoviridae	L7_Streptococcus_pseudopneumoniae	-0.043	0.029	1.45.E-01	8.15.E-01
Phycodnaviridae	L7_Streptococcus_pseudopneumoniae	-0.013	0.026	6.25.E-01	9.76.E-01
Podoviridae	L7_Streptococcus_pseudopneumoniae	0.002	0.030	9.44.E-01	9.98.E-01
Siphoviridae	L7_Streptococcus_pseudopneumoniae	-0.019	0.027	4.77.E-01	9.52.E-01
Autographiviridae	L7_Barnesiella_sp.	-0.081	0.035	1.88.E-02	5.90.E-01

crAss_like_phage	L7_Barnesiella_sp.	0.036	0.038	3.44.E-01	9.15.E-01
Herelleviridae	L7_Barnesiella_sp.	-0.006	0.036	8.69.E-01	9.96.E-01
Microviridae	L7_Barnesiella_sp.	0.040	0.036	2.74.E-01	9.04.E-01
Myoviridae	L7_Barnesiella_sp.	0.009	0.040	8.32.E-01	9.94.E-01
Phycodnaviridae	L7_Barnesiella_sp.	-0.018	0.036	6.13.E-01	9.75.E-01
Podoviridae	L7_Barnesiella_sp.	-0.068	0.042	1.02.E-01	7.81.E-01
Siphoviridae	L7_Barnesiella_sp.	0.032	0.037	3.92.E-01	9.15.E-01
Autographiviridae	L7_Prevotella_marshii	0.001	0.035	9.76.E-01	9.99.E-01
crAss_like_phage	L7_Prevotella_marshii	0.008	0.039	8.29.E-01	9.94.E-01
Herelleviridae	L7_Prevotella_marshii	0.058	0.036	1.13.E-01	7.84.E-01
Microviridae	L7_Prevotella_marshii	-0.029	0.037	4.21.E-01	9.20.E-01
Myoviridae	L7_Prevotella_marshii	0.028	0.041	4.86.E-01	9.54.E-01
Phycodnaviridae	L7_Prevotella_marshii	-0.016	0.036	6.54.E-01	9.79.E-01
Podoviridae	L7_Prevotella_marshii	-0.030	0.042	4.78.E-01	9.52.E-01
Siphoviridae	L7_Prevotella_marshii	0.007	0.038	8.50.E-01	9.95.E-01
Autographiviridae	L7_Lactobacillus_kitasatonis	-0.010	0.037	7.84.E-01	9.92.E-01
crAss_like_phage	L7_Lactobacillus_kitasatonis	-0.035	0.040	3.84.E-01	9.15.E-01
Herelleviridae	L7_Lactobacillus_kitasatonis	0.009	0.038	8.17.E-01	9.93.E-01
Microviridae	L7_Lactobacillus_kitasatonis	-0.045	0.038	2.34.E-01	8.85.E-01
Myoviridae	L7_Lactobacillus_kitasatonis	-0.027	0.043	5.18.E-01	9.57.E-01
Phycodnaviridae	L7_Lactobacillus_kitasatonis	-0.038	0.038	3.06.E-01	9.15.E-01
Podoviridae	L7_Lactobacillus_kitasatonis	-0.075	0.044	8.65.E-02	7.52.E-01
Siphoviridae	L7_Lactobacillus_kitasatonis	-0.015	0.039	7.02.E-01	9.81.E-01
Autographiviridae	L7_Faecalibacterium_sp.	-0.038	0.030	2.08.E-01	8.76.E-01
crAss_like_phage	L7_Faecalibacterium_sp.	-0.003	0.034	9.38.E-01	9.98.E-01
Herelleviridae	L7_Faecalibacterium_sp.	-0.008	0.032	7.92.E-01	9.92.E-01
Microviridae	L7_Faecalibacterium_sp.	0.028	0.032	3.80.E-01	9.15.E-01
Myoviridae	L7_Faecalibacterium_sp.	0.110	0.035	1.67.E-03	3.25.E-01
Phycodnaviridae	L7_Faecalibacterium_sp.	-0.018	0.031	5.70.E-01	9.61.E-01
Podoviridae	L7_Faecalibacterium_sp.	0.192	0.035	7.90.E-08	5.07.E-04
Siphoviridae	L7_Faecalibacterium_sp.	0.012	0.032	7.16.E-01	9.86.E-01
Autographiviridae	L7_Bifidobacterium_moukalabense	-0.008	0.037	8.33.E-01	9.94.E-01
crAss_like_phage	L7_Bifidobacterium_moukalabense	-0.061	0.040	1.28.E-01	7.99.E-01
Herelleviridae	L7_Bifidobacterium_moukalabense	0.060	0.038	1.13.E-01	7.84.E-01
Microviridae	L7_Bifidobacterium_moukalabense	0.000	0.038	9.93.E-01	9.99.E-01
Myoviridae	L7_Bifidobacterium_moukalabense	-0.026	0.042	5.40.E-01	9.60.E-01
Phycodnaviridae	L7_Bifidobacterium_moukalabense	0.020	0.038	5.91.E-01	9.68.E-01
Podoviridae	L7_Bifidobacterium_moukalabense	0.000	0.044	9.96.E-01	9.99.E-01
Siphoviridae	L7_Bifidobacterium_moukalabense	-0.007	0.039	8.66.E-01	9.96.E-01
Autographiviridae	L7_Bacteroides_cellulosilyticus	-0.056	0.035	1.08.E-01	7.82.E-01
crAss_like_phage	L7_Bacteroides_cellulosilyticus	0.025	0.039	5.26.E-01	9.57.E-01
Herelleviridae	L7_Bacteroides_cellulosilyticus	0.152	0.036	2.29.E-05	4.89.E-02
Microviridae	L7_Bacteroides_cellulosilyticus	-0.012	0.036	7.42.E-01	9.89.E-01
Myoviridae	L7_Bacteroides_cellulosilyticus	-0.003	0.041	9.33.E-01	9.98.E-01
Phycodnaviridae	L7_Bacteroides_cellulosilyticus	0.058	0.036	1.06.E-01	7.82.E-01
Podoviridae	L7_Bacteroides_cellulosilyticus	-0.018	0.042	6.66.E-01	9.80.E-01
Siphoviridae	L7_Bacteroides_cellulosilyticus	-0.039	0.037	3.00.E-01	9.15.E-01
Autographiviridae	L7_Erysipelatoclostridium_ramosum	-0.055	0.032	8.79.E-02	7.54.E-01
crAss_like_phage	L7_Erysipelatoclostridium_ramosum	0.048	0.036	1.78.E-01	8.59.E-01
Herelleviridae	L7_Erysipelatoclostridium_ramosum	-0.012	0.033	7.19.E-01	9.86.E-01
Microviridae	L7_Erysipelatoclostridium_ramosum	0.019	0.034	5.73.E-01	9.61.E-01
Myoviridae	L7_Erysipelatoclostridium_ramosum	-0.033	0.037	3.73.E-01	9.15.E-01
Phycodnaviridae	L7_Erysipelatoclostridium_ramosum	-0.012	0.033	7.21.E-01	9.86.E-01
Podoviridae	L7_Erysipelatoclostridium_ramosum	-0.024	0.039	5.37.E-01	9.60.E-01
Siphoviridae	L7_Erysipelatoclostridium_ramosum	-0.012	0.034	7.27.E-01	9.87.E-01
Autographiviridae	L7_Streptococcus_pasteurianus	-0.006	0.041	8.91.E-01	9.96.E-01
crAss_like_phage	L7_Streptococcus_pasteurianus	0.022	0.046	6.38.E-01	9.78.E-01
Herelleviridae	L7_Streptococcus_pasteurianus	0.011	0.043	7.92.E-01	9.92.E-01
Microviridae	L7_Streptococcus_pasteurianus	-0.007	0.043	8.69.E-01	9.96.E-01
Myoviridae	L7_Streptococcus_pasteurianus	-0.086	0.048	7.20.E-02	7.25.E-01
Phycodnaviridae	L7_Streptococcus_pasteurianus	-0.048	0.042	2.54.E-01	8.95.E-01
Podoviridae	L7_Streptococcus_pasteurianus	-0.005	0.050	9.20.E-01	9.98.E-01
Siphoviridae	L7_Streptococcus_pasteurianus	-0.014	0.044	7.53.E-01	9.89.E-01
Autographiviridae	L7_Stomatobaculum_longum	0.028	0.041	4.93.E-01	9.55.E-01
crAss_like_phage	L7_Stomatobaculum_longum	0.078	0.045	8.38.E-02	7.50.E-01

Herelleviridae	L7_Stomatobaculum_longum	-0.036	0.042	3.89.E-01	9.15.E-01
Microviridae	L7_Stomatobaculum_longum	-0.013	0.042	7.61.E-01	9.91.E-01
Myoviridae	L7_Stomatobaculum_longum	0.093	0.047	4.94.E-02	7.22.E-01
Phycodnaviridae	L7_Stomatobaculum_longum	-0.027	0.042	5.18.E-01	9.57.E-01
Podoviridae	L7_Stomatobaculum_longum	0.009	0.049	8.52.E-01	9.95.E-01
Siphoviridae	L7_Stomatobaculum_longum	-0.028	0.044	5.26.E-01	9.57.E-01
Autographiviridae	L7_Intestinibacter_bartlettii	0.002	0.039	9.58.E-01	9.98.E-01
crAss_like_phage	L7_Intestinibacter_bartlettii	-0.083	0.043	5.32.E-02	7.22.E-01
Herelleviridae	L7_Intestinibacter_bartlettii	0.027	0.040	5.08.E-01	9.57.E-01
Microviridae	L7_Intestinibacter_bartlettii	-0.030	0.040	4.65.E-01	9.45.E-01
Myoviridae	L7_Intestinibacter_bartlettii	0.030	0.045	5.10.E-01	9.57.E-01
Phycodnaviridae	L7_Intestinibacter_bartlettii	0.028	0.040	4.89.E-01	9.55.E-01
Podoviridae	L7_Intestinibacter_bartlettii	-0.004	0.046	9.32.E-01	9.98.E-01
Siphoviridae	L7_Intestinibacter_bartlettii	0.013	0.041	7.62.E-01	9.91.E-01
Autographiviridae	L7_Prevotella_timonensis	0.028	0.035	4.35.E-01	9.26.E-01
crAss_like_phage	L7_Prevotella_timonensis	0.021	0.039	5.83.E-01	9.64.E-01
Herelleviridae	L7_Prevotella_timonensis	-0.001	0.037	9.82.E-01	9.99.E-01
Microviridae	L7_Prevotella_timonensis	-0.010	0.037	7.82.E-01	9.92.E-01
Myoviridae	L7_Prevotella_timonensis	-0.017	0.041	6.84.E-01	9.80.E-01
Phycodnaviridae	L7_Prevotella_timonensis	-0.057	0.036	1.12.E-01	7.83.E-01
Podoviridae	L7_Prevotella_timonensis	-0.060	0.042	1.56.E-01	8.29.E-01
Siphoviridae	L7_Prevotella_timonensis	-0.062	0.038	9.73.E-02	7.74.E-01
Autographiviridae	L7_Klebsiella_variicola	0.012	0.028	6.75.E-01	9.80.E-01
crAss_like_phage	L7_Klebsiella_variicola	0.036	0.031	2.50.E-01	8.95.E-01
Herelleviridae	L7_Klebsiella_variicola	-0.026	0.029	3.69.E-01	9.15.E-01
Microviridae	L7_Klebsiella_variicola	0.002	0.029	9.44.E-01	9.98.E-01
Myoviridae	L7_Klebsiella_variicola	-0.037	0.032	2.60.E-01	8.96.E-01
Phycodnaviridae	L7_Klebsiella_variicola	-0.029	0.029	3.20.E-01	9.15.E-01
Podoviridae	L7_Klebsiella_variicola	0.013	0.034	7.00.E-01	9.81.E-01
Siphoviridae	L7_Klebsiella_variicola	0.022	0.030	4.70.E-01	9.50.E-01
Autographiviridae	L7_Faecalicatena_contorta	0.030	0.033	3.69.E-01	9.15.E-01
crAss_like_phage	L7_Faecalicatena_contorta	0.034	0.037	3.58.E-01	9.15.E-01
Herelleviridae	L7_Faecalicatena_contorta	-0.040	0.034	2.51.E-01	8.95.E-01
Microviridae	L7_Faecalicatena_contorta	-0.058	0.034	9.31.E-02	7.60.E-01
Myoviridae	L7_Faecalicatena_contorta	0.027	0.039	4.82.E-01	9.53.E-01
Phycodnaviridae	L7_Faecalicatena_contorta	-0.015	0.034	6.54.E-01	9.79.E-01
Podoviridae	L7_Faecalicatena_contorta	0.014	0.040	7.35.E-01	9.88.E-01
Siphoviridae	L7_Faecalicatena_contorta	-0.031	0.035	3.76.E-01	9.15.E-01
Autographiviridae	L7_Clostridium_perfringens	-0.031	0.036	3.83.E-01	9.15.E-01
crAss_like_phage	L7_Clostridium_perfringens	0.012	0.039	7.66.E-01	9.91.E-01
Herelleviridae	L7_Clostridium_perfringens	0.051	0.037	1.68.E-01	8.44.E-01
Microviridae	L7_Clostridium_perfringens	-0.033	0.037	3.79.E-01	9.15.E-01
Myoviridae	L7_Clostridium_perfringens	-0.006	0.041	8.91.E-01	9.96.E-01
Phycodnaviridae	L7_Clostridium_perfringens	0.039	0.037	2.93.E-01	9.15.E-01
Podoviridae	L7_Clostridium_perfringens	0.002	0.043	9.54.E-01	9.98.E-01
Siphoviridae	L7_Clostridium_perfringens	-0.011	0.038	7.63.E-01	9.91.E-01
Autographiviridae	L7_Clostridium_disporicum	0.006	0.034	8.65.E-01	9.96.E-01
crAss_like_phage	L7_Clostridium_disporicum	0.000	0.038	1.00.E+00	1.00.E+00
Herelleviridae	L7_Clostridium_disporicum	0.048	0.036	1.83.E-01	8.60.E-01
Microviridae	L7_Clostridium_disporicum	-0.021	0.036	5.56.E-01	9.61.E-01
Myoviridae	L7_Clostridium_disporicum	-0.014	0.040	7.28.E-01	9.88.E-01
Phycodnaviridae	L7_Clostridium_disporicum	0.041	0.035	2.43.E-01	8.87.E-01
Podoviridae	L7_Clostridium_disporicum	0.006	0.041	8.85.E-01	9.96.E-01
Siphoviridae	L7_Clostridium_disporicum	-0.017	0.037	6.46.E-01	9.79.E-01
Autographiviridae	L7_Absiella_sp.	-0.060	0.037	1.04.E-01	7.82.E-01
crAss_like_phage	L7_Absiella_sp.	0.060	0.041	1.40.E-01	8.10.E-01
Herelleviridae	L7_Absiella_sp.	0.010	0.038	7.91.E-01	9.92.E-01
Microviridae	L7_Absiella_sp.	0.013	0.039	7.29.E-01	9.88.E-01
Myoviridae	L7_Absiella_sp.	0.010	0.043	8.22.E-01	9.93.E-01
Phycodnaviridae	L7_Absiella_sp.	-0.027	0.038	4.72.E-01	9.51.E-01
Podoviridae	L7_Absiella_sp.	-0.092	0.044	3.83.E-02	7.03.E-01
Siphoviridae	L7_Absiella_sp.	0.034	0.040	3.95.E-01	9.15.E-01
Autographiviridae	L7_Peptoanaerobacter_stomatis	0.011	0.040	7.82.E-01	9.92.E-01
crAss_like_phage	L7_Peptoanaerobacter_stomatis	-0.103	0.044	1.95.E-02	5.90.E-01
Herelleviridae	L7_Peptoanaerobacter_stomatis	-0.014	0.041	7.33.E-01	9.88.E-01

Microviridae	L7_Peptoanaerobacter_stomatis	0.024	0.042	5.60.E-01	9.61.E-01
Myoviridae	L7_Peptoanaerobacter_stomatis	0.041	0.046	3.80.E-01	9.15.E-01
Phycodnaviridae	L7_Peptoanaerobacter_stomatis	-0.021	0.041	6.17.E-01	9.75.E-01
Podoviridae	L7_Peptoanaerobacter_stomatis	0.061	0.048	2.03.E-01	8.75.E-01
Siphoviridae	L7_Peptoanaerobacter_stomatis	0.026	0.043	5.45.E-01	9.60.E-01
Autographiviridae	L7_Campylobacter_ureolyticus	0.022	0.021	2.89.E-01	9.15.E-01
crAss_like_phage	L7_Campylobacter_ureolyticus	0.026	0.023	2.51.E-01	8.95.E-01
Herelleviridae	L7_Campylobacter_ureolyticus	0.016	0.022	4.50.E-01	9.37.E-01
Microviridae	L7_Campylobacter_ureolyticus	0.006	0.022	7.66.E-01	9.91.E-01
Myoviridae	L7_Campylobacter_ureolyticus	-0.011	0.024	6.56.E-01	9.79.E-01
Phycodnaviridae	L7_Campylobacter_ureolyticus	-0.031	0.021	1.50.E-01	8.22.E-01
Podoviridae	L7_Campylobacter_ureolyticus	-0.019	0.025	4.42.E-01	9.32.E-01
Siphoviridae	L7_Campylobacter_ureolyticus	0.001	0.022	9.54.E-01	9.98.E-01
Autographiviridae	L7_Bacteroides_nordii	-0.007	0.029	7.99.E-01	9.92.E-01
crAss_like_phage	L7_Bacteroides_nordii	-0.001	0.032	9.81.E-01	9.99.E-01
Herelleviridae	L7_Bacteroides_nordii	-0.012	0.030	6.90.E-01	9.80.E-01
Microviridae	L7_Bacteroides_nordii	-0.050	0.030	9.78.E-02	7.77.E-01
Myoviridae	L7_Bacteroides_nordii	-0.016	0.034	6.33.E-01	9.76.E-01
Phycodnaviridae	L7_Bacteroides_nordii	0.026	0.030	3.81.E-01	9.15.E-01
Podoviridae	L7_Bacteroides_nordii	-0.037	0.035	2.85.E-01	9.15.E-01
Siphoviridae	L7_Bacteroides_nordii	0.000	0.031	9.93.E-01	9.99.E-01
Autographiviridae	L7_Bacteroides_thetaiotaomicron	-0.002	0.029	9.58.E-01	9.98.E-01
crAss_like_phage	L7_Bacteroides_thetaiotaomicron	0.003	0.032	9.24.E-01	9.98.E-01
Herelleviridae	L7_Bacteroides_thetaiotaomicron	-0.023	0.030	4.49.E-01	9.37.E-01
Microviridae	L7_Bacteroides_thetaiotaomicron	0.025	0.031	4.14.E-01	9.18.E-01
Myoviridae	L7_Bacteroides_thetaiotaomicron	0.042	0.034	2.17.E-01	8.79.E-01
Phycodnaviridae	L7_Bacteroides_thetaiotaomicron	0.008	0.030	7.96.E-01	9.92.E-01
Podoviridae	L7_Bacteroides_thetaiotaomicron	-0.017	0.035	6.21.E-01	9.76.E-01
Siphoviridae	L7_Bacteroides_thetaiotaomicron	0.026	0.031	4.13.E-01	9.18.E-01
Autographiviridae	L7_Enterococcus_avium	0.031	0.034	3.57.E-01	9.15.E-01
crAss_like_phage	L7_Enterococcus_avium	-0.065	0.037	7.89.E-02	7.43.E-01
Herelleviridae	L7_Enterococcus_avium	0.028	0.035	4.32.E-01	9.25.E-01
Microviridae	L7_Enterococcus_avium	-0.047	0.035	1.80.E-01	8.60.E-01
Myoviridae	L7_Enterococcus_avium	-0.024	0.039	5.44.E-01	9.60.E-01
Phycodnaviridae	L7_Enterococcus_avium	-0.022	0.035	5.19.E-01	9.57.E-01
Podoviridae	L7_Enterococcus_avium	0.036	0.040	3.76.E-01	9.15.E-01
Siphoviridae	L7_Enterococcus_avium	0.001	0.036	9.87.E-01	9.99.E-01
Autographiviridae	L7_Gemmiger_formicilis	-0.061	0.035	8.61.E-02	7.52.E-01
crAss_like_phage	L7_Gemmiger_formicilis	0.076	0.039	5.25.E-02	7.22.E-01
Herelleviridae	L7_Gemmiger_formicilis	-0.038	0.037	3.02.E-01	9.15.E-01
Microviridae	L7_Gemmiger_formicilis	-0.025	0.037	4.92.E-01	9.55.E-01
Myoviridae	L7_Gemmiger_formicilis	-0.035	0.041	4.01.E-01	9.15.E-01
Phycodnaviridae	L7_Gemmiger_formicilis	0.032	0.036	3.86.E-01	9.15.E-01
Podoviridae	L7_Gemmiger_formicilis	0.037	0.043	3.88.E-01	9.15.E-01
Siphoviridae	L7_Gemmiger_formicilis	0.010	0.038	7.93.E-01	9.92.E-01
Autographiviridae	L7_Raoultella_ornithinolytica	0.030	0.028	2.83.E-01	9.13.E-01
crAss_like_phage	L7_Raoultella_ornithinolytica	0.041	0.031	1.91.E-01	8.61.E-01
Herelleviridae	L7_Raoultella_ornithinolytica	-0.020	0.029	5.01.E-01	9.55.E-01
Microviridae	L7_Raoultella_ornithinolytica	-0.058	0.029	4.94.E-02	7.22.E-01
Myoviridae	L7_Raoultella_ornithinolytica	-0.033	0.033	3.10.E-01	9.15.E-01
Phycodnaviridae	L7_Raoultella_ornithinolytica	-0.038	0.029	1.95.E-01	8.66.E-01
Podoviridae	L7_Raoultella_ornithinolytica	0.046	0.034	1.69.E-01	8.48.E-01
Siphoviridae	L7_Raoultella_ornithinolytica	0.026	0.030	3.95.E-01	9.15.E-01
Autographiviridae	L7_Enterococcus_faecium	0.057	0.040	1.50.E-01	8.22.E-01
crAss_like_phage	L7_Enterococcus_faecium	0.034	0.044	4.39.E-01	9.30.E-01
Herelleviridae	L7_Enterococcus_faecium	0.038	0.041	3.59.E-01	9.15.E-01
Microviridae	L7_Enterococcus_faecium	-0.036	0.041	3.85.E-01	9.15.E-01
Myoviridae	L7_Enterococcus_faecium	0.082	0.046	7.58.E-02	7.31.E-01
Phycodnaviridae	L7_Enterococcus_faecium	-0.013	0.041	7.44.E-01	9.89.E-01
Podoviridae	L7_Enterococcus_faecium	-0.002	0.048	9.72.E-01	9.99.E-01
Siphoviridae	L7_Enterococcus_faecium	-0.045	0.042	2.94.E-01	9.15.E-01
Autographiviridae	L7_Flavonifractor_sp.	0.003	0.033	9.31.E-01	9.98.E-01
crAss_like_phage	L7_Flavonifractor_sp.	0.021	0.036	5.59.E-01	9.61.E-01
Herelleviridae	L7_Flavonifractor_sp.	-0.047	0.034	1.67.E-01	8.44.E-01
Microviridae	L7_Flavonifractor_sp.	0.065	0.034	5.71.E-02	7.24.E-01

Myoviridae	L7_Flavonifractor_sp.	0.026	0.038	4.88.E-01	9.54.E-01
Phycodnaviridae	L7_Flavonifractor_sp.	0.094	0.033	4.97.E-03	4.28.E-01
Podoviridae	L7_Flavonifractor_sp.	0.020	0.039	6.02.E-01	9.71.E-01
Siphoviridae	L7_Flavonifractor_sp.	0.079	0.035	2.28.E-02	6.19.E-01
Autographiviridae	L7_Delftia_sp.	-0.007	0.017	6.90.E-01	9.80.E-01
crAss_like_phage	L7_Delftia_sp.	-0.029	0.018	1.14.E-01	7.85.E-01
Herelleviridae	L7_Delftia_sp.	0.001	0.017	9.53.E-01	9.98.E-01
Microviridae	L7_Delftia_sp.	-0.002	0.017	9.24.E-01	9.98.E-01
Myoviridae	L7_Delftia_sp.	-0.004	0.019	8.17.E-01	9.93.E-01
Phycodnaviridae	L7_Delftia_sp.	0.001	0.017	9.64.E-01	9.98.E-01
Podoviridae	L7_Delftia_sp.	-0.011	0.020	5.86.E-01	9.65.E-01
Siphoviridae	L7_Delftia_sp.	-0.027	0.018	1.27.E-01	7.98.E-01
Autographiviridae	L7_Actinomyces_sp.	-0.053	0.029	6.96.E-02	7.24.E-01
crAss_like_phage	L7_Actinomyces_sp.	-0.014	0.033	6.63.E-01	9.79.E-01
Herelleviridae	L7_Actinomyces_sp.	-0.068	0.030	2.56.E-02	6.50.E-01
Microviridae	L7_Actinomyces_sp.	0.026	0.031	3.95.E-01	9.15.E-01
Myoviridae	L7_Actinomyces_sp.	-0.042	0.034	2.20.E-01	8.79.E-01
Phycodnaviridae	L7_Actinomyces_sp.	-0.035	0.030	2.52.E-01	8.95.E-01
Podoviridae	L7_Actinomyces_sp.	0.003	0.035	9.38.E-01	9.98.E-01
Siphoviridae	L7_Actinomyces_sp.	0.018	0.031	5.71.E-01	9.61.E-01
Autographiviridae	L7_Alistipes_sp.	0.007	0.029	8.06.E-01	9.93.E-01
crAss_like_phage	L7_Alistipes_sp.	0.086	0.031	6.22.E-03	4.51.E-01
Herelleviridae	L7_Alistipes_sp.	0.060	0.030	4.44.E-02	7.22.E-01
Microviridae	L7_Alistipes_sp.	-0.001	0.030	9.63.E-01	9.98.E-01
Myoviridae	L7_Alistipes_sp.	-0.015	0.033	6.63.E-01	9.79.E-01
Phycodnaviridae	L7_Alistipes_sp.	-0.048	0.029	1.02.E-01	7.81.E-01
Podoviridae	L7_Alistipes_sp.	0.057	0.034	9.54.E-02	7.65.E-01
Siphoviridae	L7_Alistipes_sp.	0.010	0.031	7.43.E-01	9.89.E-01
Autographiviridae	L7_Bacteroides_massiliensis	-0.005	0.037	8.82.E-01	9.96.E-01
crAss_like_phage	L7_Bacteroides_massiliensis	0.019	0.041	6.35.E-01	9.76.E-01
Herelleviridae	L7_Bacteroides_massiliensis	0.032	0.038	4.05.E-01	9.17.E-01
Microviridae	L7_Bacteroides_massiliensis	0.005	0.038	8.88.E-01	9.96.E-01
Myoviridae	L7_Bacteroides_massiliensis	0.087	0.042	4.00.E-02	7.03.E-01
Phycodnaviridae	L7_Bacteroides_massiliensis	-0.019	0.038	6.23.E-01	9.76.E-01
Podoviridae	L7_Bacteroides_massiliensis	0.066	0.044	1.35.E-01	8.06.E-01
Siphoviridae	L7_Bacteroides_massiliensis	0.035	0.039	3.74.E-01	9.15.E-01
Autographiviridae	L7_Streptococcus_salivarius	0.003	0.030	9.33.E-01	9.98.E-01
crAss_like_phage	L7_Streptococcus_salivarius	-0.001	0.033	9.80.E-01	9.99.E-01
Herelleviridae	L7_Streptococcus_salivarius	-0.002	0.031	9.53.E-01	9.98.E-01
Microviridae	L7_Streptococcus_salivarius	0.022	0.031	4.76.E-01	9.52.E-01
Myoviridae	L7_Streptococcus_salivarius	0.046	0.034	1.78.E-01	8.59.E-01
Phycodnaviridae	L7_Streptococcus_salivarius	0.043	0.030	1.53.E-01	8.25.E-01
Podoviridae	L7_Streptococcus_salivarius	0.012	0.036	7.28.E-01	9.88.E-01
Siphoviridae	L7_Streptococcus_salivarius	0.009	0.032	7.86.E-01	9.92.E-01
Autographiviridae	L7_Tannerella_sp.	0.010	0.025	6.77.E-01	9.80.E-01
crAss_like_phage	L7_Tannerella_sp.	0.034	0.028	2.22.E-01	8.80.E-01
Herelleviridae	L7_Tannerella_sp.	0.046	0.026	7.63.E-02	7.32.E-01
Microviridae	L7_Tannerella_sp.	0.025	0.026	3.40.E-01	9.15.E-01
Myoviridae	L7_Tannerella_sp.	0.041	0.029	1.56.E-01	8.29.E-01
Phycodnaviridae	L7_Tannerella_sp.	-0.007	0.026	7.93.E-01	9.92.E-01
Podoviridae	L7_Tannerella_sp.	-0.001	0.030	9.74.E-01	9.99.E-01
Siphoviridae	L7_Tannerella_sp.	0.015	0.027	5.65.E-01	9.61.E-01
Autographiviridae	L7_Christensenella_timonensis	0.013	0.031	6.75.E-01	9.80.E-01
crAss_like_phage	L7_Christensenella_timonensis	-0.034	0.034	3.24.E-01	9.15.E-01
Herelleviridae	L7_Christensenella_timonensis	-0.055	0.032	8.73.E-02	7.52.E-01
Microviridae	L7_Christensenella_timonensis	0.018	0.032	5.70.E-01	9.61.E-01
Myoviridae	L7_Christensenella_timonensis	0.017	0.036	6.43.E-01	9.79.E-01
Phycodnaviridae	L7_Christensenella_timonensis	-0.069	0.032	3.03.E-02	6.66.E-01
Podoviridae	L7_Christensenella_timonensis	0.009	0.037	8.07.E-01	9.93.E-01
Siphoviridae	L7_Christensenella_timonensis	-0.010	0.033	7.51.E-01	9.89.E-01
Autographiviridae	L7_Raoultella_planticola	0.073	0.029	1.26.E-02	5.43.E-01
crAss_like_phage	L7_Raoultella_planticola	0.051	0.032	1.16.E-01	7.85.E-01
Herelleviridae	L7_Raoultella_planticola	-0.028	0.030	3.57.E-01	9.15.E-01
Microviridae	L7_Raoultella_planticola	-0.027	0.030	3.80.E-01	9.15.E-01
Myoviridae	L7_Raoultella_planticola	-0.035	0.034	3.03.E-01	9.15.E-01

Phycodnaviridae	L7_Raoultella_planticola	-0.056	0.030	6.06.E-02	7.24.E-01
Podoviridae	L7_Raoultella_planticola	-0.031	0.035	3.84.E-01	9.15.E-01
Siphoviridae	L7_Raoultella_planticola	-0.021	0.031	4.94.E-01	9.55.E-01
Autographiviridae	L7_Prevotella_amnii	0.043	0.022	5.67.E-02	7.24.E-01
crAss_like_phage	L7_Prevotella_amnii	0.005	0.025	8.25.E-01	9.93.E-01
Herelleviridae	L7_Prevotella_amnii	0.003	0.023	8.84.E-01	9.96.E-01
Microviridae	L7_Prevotella_amnii	0.002	0.023	9.36.E-01	9.98.E-01
Myoviridae	L7_Prevotella_amnii	-0.007	0.026	7.91.E-01	9.92.E-01
Phycodnaviridae	L7_Prevotella_amnii	-0.017	0.023	4.72.E-01	9.51.E-01
Podoviridae	L7_Prevotella_amnii	0.012	0.027	6.53.E-01	9.79.E-01
Siphoviridae	L7_Prevotella_amnii	-0.020	0.024	3.98.E-01	9.15.E-01
Autographiviridae	L7_Akkermansia_sp.	-0.023	0.039	5.64.E-01	9.61.E-01
crAss_like_phage	L7_Akkermansia_sp.	0.016	0.044	7.11.E-01	9.85.E-01
Herelleviridae	L7_Akkermansia_sp.	0.031	0.041	4.45.E-01	9.33.E-01
Microviridae	L7_Akkermansia_sp.	-0.041	0.041	3.13.E-01	9.15.E-01
Myoviridae	L7_Akkermansia_sp.	0.052	0.046	2.54.E-01	8.95.E-01
Phycodnaviridae	L7_Akkermansia_sp.	0.039	0.041	3.31.E-01	9.15.E-01
Podoviridae	L7_Akkermansia_sp.	0.072	0.047	1.27.E-01	7.98.E-01
Siphoviridae	L7_Akkermansia_sp.	0.000	0.042	9.93.E-01	9.99.E-01
Autographiviridae	L7_Anaerostipes_caccae	-0.059	0.032	6.73.E-02	7.24.E-01
crAss_like_phage	L7_Anaerostipes_caccae	-0.040	0.035	2.57.E-01	8.95.E-01
Herelleviridae	L7_Anaerostipes_caccae	-0.009	0.033	7.98.E-01	9.92.E-01
Microviridae	L7_Anaerostipes_caccae	-0.035	0.033	2.95.E-01	9.15.E-01
Myoviridae	L7_Anaerostipes_caccae	0.008	0.037	8.35.E-01	9.94.E-01
Phycodnaviridae	L7_Anaerostipes_caccae	0.004	0.033	9.00.E-01	9.97.E-01
Podoviridae	L7_Anaerostipes_caccae	-0.061	0.038	1.16.E-01	7.86.E-01
Siphoviridae	L7_Anaerostipes_caccae	-0.047	0.034	1.67.E-01	8.44.E-01
Autographiviridae	L7_Alistipes_indistinctus	0.058	0.036	1.03.E-01	7.82.E-01
crAss_like_phage	L7_Alistipes_indistinctus	-0.001	0.040	9.78.E-01	9.99.E-01
Herelleviridae	L7_Alistipes_indistinctus	0.014	0.037	7.08.E-01	9.83.E-01
Microviridae	L7_Alistipes_indistinctus	-0.001	0.037	9.70.E-01	9.99.E-01
Myoviridae	L7_Alistipes_indistinctus	-0.010	0.042	8.13.E-01	9.93.E-01
Phycodnaviridae	L7_Alistipes_indistinctus	-0.061	0.037	9.47.E-02	7.64.E-01
Podoviridae	L7_Alistipes_indistinctus	-0.018	0.043	6.67.E-01	9.80.E-01
Siphoviridae	L7_Alistipes_indistinctus	-0.019	0.038	6.19.E-01	9.75.E-01
Autographiviridae	L7_Dorea_longicatena	0.038	0.039	3.31.E-01	9.15.E-01
crAss_like_phage	L7_Dorea_longicatena	0.017	0.043	6.88.E-01	9.80.E-01
Herelleviridae	L7_Dorea_longicatena	-0.130	0.040	1.27.E-03	2.90.E-01
Microviridae	L7_Dorea_longicatena	-0.114	0.040	4.89.E-03	4.28.E-01
Myoviridae	L7_Dorea_longicatena	-0.068	0.045	1.35.E-01	8.06.E-01
Phycodnaviridae	L7_Dorea_longicatena	-0.024	0.040	5.56.E-01	9.61.E-01
Podoviridae	L7_Dorea_longicatena	0.053	0.047	2.55.E-01	8.95.E-01
Siphoviridae	L7_Dorea_longicatena	-0.049	0.042	2.35.E-01	8.85.E-01
Autographiviridae	L7_Corynebacterium_argentoratense	0.022	0.045	6.16.E-01	9.75.E-01
crAss_like_phage	L7_Corynebacterium_argentoratense	0.058	0.049	2.36.E-01	8.85.E-01
Herelleviridae	L7_Corynebacterium_argentoratense	-0.015	0.046	7.50.E-01	9.89.E-01
Microviridae	L7_Corynebacterium_argentoratense	-0.080	0.046	8.47.E-02	7.50.E-01
Myoviridae	L7_Corynebacterium_argentoratense	0.114	0.052	2.80.E-02	6.58.E-01
Phycodnaviridae	L7_Corynebacterium_argentoratense	-0.043	0.046	3.45.E-01	9.15.E-01
Podoviridae	L7_Corynebacterium_argentoratense	0.111	0.053	3.78.E-02	7.01.E-01
Siphoviridae	L7_Corynebacterium_argentoratense	0.050	0.048	2.93.E-01	9.15.E-01
Autographiviridae	L7_Bacteroides_coprocola	0.068	0.039	8.17.E-02	7.47.E-01
crAss_like_phage	L7_Bacteroides_coprocola	0.075	0.043	8.39.E-02	7.50.E-01
Herelleviridae	L7_Bacteroides_coprocola	-0.003	0.041	9.39.E-01	9.98.E-01
Microviridae	L7_Bacteroides_coprocola	0.075	0.041	6.60.E-02	7.24.E-01
Myoviridae	L7_Bacteroides_coprocola	0.009	0.046	8.44.E-01	9.95.E-01
Phycodnaviridae	L7_Bacteroides_coprocola	-0.050	0.040	2.14.E-01	8.78.E-01
Podoviridae	L7_Bacteroides_coprocola	0.046	0.047	3.33.E-01	9.15.E-01
Siphoviridae	L7_Bacteroides_coprocola	-0.005	0.042	9.04.E-01	9.97.E-01
Autographiviridae	L7_Streptococcus_sanguinis	-0.008	0.025	7.45.E-01	9.89.E-01
crAss_like_phage	L7_Streptococcus_sanguinis	0.054	0.028	5.47.E-02	7.23.E-01
Herelleviridae	L7_Streptococcus_sanguinis	0.006	0.026	8.27.E-01	9.93.E-01
Microviridae	L7_Streptococcus_sanguinis	-0.028	0.026	2.93.E-01	9.15.E-01
Myoviridae	L7_Streptococcus_sanguinis	0.083	0.029	4.60.E-03	4.28.E-01
Phycodnaviridae	L7_Streptococcus_sanguinis	0.000	0.026	9.89.E-01	9.99.E-01

Podoviridae	L7_Streptococcus_sanguinis	-0.017	0.031	5.76.E-01	9.62.E-01
Siphoviridae	L7_Streptococcus_sanguinis	0.031	0.027	2.49.E-01	8.94.E-01
Autographiviridae	L7_Streptococcus_mitis	-0.016	0.025	5.32.E-01	9.57.E-01
crAss_like_phage	L7_Streptococcus_mitis	0.028	0.027	3.16.E-01	9.15.E-01
Herelleviridae	L7_Streptococcus_mitis	-0.014	0.026	5.96.E-01	9.69.E-01
Microviridae	L7_Streptococcus_mitis	-0.046	0.026	7.24.E-02	7.26.E-01
Myoviridae	L7_Streptococcus_mitis	-0.041	0.029	1.50.E-01	8.22.E-01
Phycodnaviridae	L7_Streptococcus_mitis	-0.008	0.025	7.48.E-01	9.89.E-01
Podoviridae	L7_Streptococcus_mitis	-0.013	0.030	6.68.E-01	9.80.E-01
Siphoviridae	L7_Streptococcus_mitis	-0.018	0.027	4.86.E-01	9.54.E-01
Autographiviridae	L7_Actinomyces_turicensis	0.036	0.042	3.91.E-01	9.15.E-01
crAss_like_phage	L7_Actinomyces_turicensis	0.003	0.047	9.51.E-01	9.98.E-01
Herelleviridae	L7_Actinomyces_turicensis	0.053	0.044	2.28.E-01	8.82.E-01
Microviridae	L7_Actinomyces_turicensis	-0.015	0.044	7.32.E-01	9.88.E-01
Myoviridae	L7_Actinomyces_turicensis	-0.071	0.049	1.47.E-01	8.16.E-01
Phycodnaviridae	L7_Actinomyces_turicensis	0.035	0.044	4.21.E-01	9.20.E-01
Podoviridae	L7_Actinomyces_turicensis	-0.021	0.051	6.85.E-01	9.80.E-01
Siphoviridae	L7_Actinomyces_turicensis	-0.026	0.045	5.71.E-01	9.61.E-01
Autographiviridae	L7_Bacteroides_salyersiae	-0.002	0.030	9.45.E-01	9.98.E-01
crAss_like_phage	L7_Bacteroides_salyersiae	-0.023	0.033	4.75.E-01	9.52.E-01
Herelleviridae	L7_Bacteroides_salyersiae	0.022	0.031	4.71.E-01	9.50.E-01
Microviridae	L7_Bacteroides_salyersiae	-0.038	0.031	2.16.E-01	8.79.E-01
Myoviridae	L7_Bacteroides_salyersiae	-0.062	0.034	7.05.E-02	7.24.E-01
Phycodnaviridae	L7_Bacteroides_salyersiae	-0.035	0.031	2.56.E-01	8.95.E-01
Podoviridae	L7_Bacteroides_salyersiae	-0.094	0.035	8.16.E-03	4.75.E-01
Siphoviridae	L7_Bacteroides_salyersiae	-0.025	0.032	4.34.E-01	9.26.E-01
Autographiviridae	L7_Mitsuokella_multacida	-0.047	0.039	2.33.E-01	8.84.E-01
crAss_like_phage	L7_Mitsuokella_multacida	0.006	0.043	8.98.E-01	9.97.E-01
Herelleviridae	L7_Mitsuokella_multacida	-0.083	0.041	4.06.E-02	7.05.E-01
Microviridae	L7_Mitsuokella_multacida	-0.009	0.041	8.27.E-01	9.93.E-01
Myoviridae	L7_Mitsuokella_multacida	0.037	0.045	4.16.E-01	9.19.E-01
Phycodnaviridae	L7_Mitsuokella_multacida	-0.009	0.040	8.19.E-01	9.93.E-01
Podoviridae	L7_Mitsuokella_multacida	-0.024	0.047	6.06.E-01	9.73.E-01
Siphoviridae	L7_Mitsuokella_multacida	0.017	0.042	6.84.E-01	9.80.E-01
Autographiviridae	L7_Eubacterium_ramulus	-0.002	0.039	9.61.E-01	9.98.E-01
crAss_like_phage	L7_Eubacterium_ramulus	0.040	0.044	3.62.E-01	9.15.E-01
Herelleviridae	L7_Eubacterium_ramulus	0.029	0.041	4.81.E-01	9.53.E-01
Microviridae	L7_Eubacterium_ramulus	-0.009	0.041	8.34.E-01	9.94.E-01
Myoviridae	L7_Eubacterium_ramulus	0.037	0.046	4.16.E-01	9.19.E-01
Phycodnaviridae	L7_Eubacterium_ramulus	-0.001	0.041	9.89.E-01	9.99.E-01
Podoviridae	L7_Eubacterium_ramulus	0.009	0.047	8.43.E-01	9.95.E-01
Siphoviridae	L7_Eubacterium_ramulus	0.012	0.042	7.78.E-01	9.92.E-01
Autographiviridae	L7_Veillonella_magna	0.007	0.042	8.73.E-01	9.96.E-01
crAss_like_phage	L7_Veillonella_magna	-0.022	0.047	6.35.E-01	9.76.E-01
Herelleviridae	L7_Veillonella_magna	-0.050	0.044	2.58.E-01	8.95.E-01
Microviridae	L7_Veillonella_magna	0.034	0.044	4.37.E-01	9.27.E-01
Myoviridae	L7_Veillonella_magna	-0.043	0.049	3.85.E-01	9.15.E-01
Phycodnaviridae	L7_Veillonella_magna	-0.050	0.044	2.49.E-01	8.94.E-01
Podoviridae	L7_Veillonella_magna	-0.079	0.051	1.20.E-01	7.91.E-01
Siphoviridae	L7_Veillonella_magna	-0.138	0.045	2.27.E-03	3.52.E-01
Autographiviridae	L7_Porphyromonas_sp.	-0.032	0.035	3.56.E-01	9.15.E-01
crAss_like_phage	L7_Porphyromonas_sp.	0.044	0.038	2.52.E-01	8.95.E-01
Herelleviridae	L7_Porphyromonas_sp.	0.037	0.036	3.04.E-01	9.15.E-01
Microviridae	L7_Porphyromonas_sp.	-0.017	0.036	6.41.E-01	9.79.E-01
Myoviridae	L7_Porphyromonas_sp.	-0.072	0.040	7.15.E-02	7.24.E-01
Phycodnaviridae	L7_Porphyromonas_sp.	0.029	0.035	4.16.E-01	9.19.E-01
Podoviridae	L7_Porphyromonas_sp.	0.065	0.041	1.15.E-01	7.85.E-01
Siphoviridae	L7_Porphyromonas_sp.	0.061	0.037	1.01.E-01	7.79.E-01
Autographiviridae	L7_Enterobacter_asburiae	-0.038	0.029	1.93.E-01	8.62.E-01
crAss_like_phage	L7_Enterobacter_asburiae	-0.018	0.032	5.69.E-01	9.61.E-01
Herelleviridae	L7_Enterobacter_asburiae	-0.016	0.030	5.96.E-01	9.69.E-01
Microviridae	L7_Enterobacter_asburiae	0.014	0.030	6.54.E-01	9.79.E-01
Myoviridae	L7_Enterobacter_asburiae	-0.013	0.034	7.05.E-01	9.82.E-01
Phycodnaviridae	L7_Enterobacter_asburiae	-0.055	0.030	6.67.E-02	7.24.E-01
Podoviridae	L7_Enterobacter_asburiae	0.024	0.035	4.89.E-01	9.55.E-01

Siphoviridae	L7_Enterobacter_asburiae	0.017	0.031	5.78.E-01	9.64.E-01
Autographiviridae	L7_Subdoligranulum_variabile	-0.005	0.030	8.79.E-01	9.96.E-01
crAss_like_phage	L7_Subdoligranulum_variabile	-0.011	0.034	7.48.E-01	9.89.E-01
Herelleviridae	L7_Subdoligranulum_variabile	-0.090	0.031	4.29.E-03	4.23.E-01
Microviridae	L7_Subdoligranulum_variabile	-0.058	0.032	6.77.E-02	7.24.E-01
Myoviridae	L7_Subdoligranulum_variabile	-0.043	0.035	2.25.E-01	8.82.E-01
Phycodnaviridae	L7_Subdoligranulum_variabile	-0.039	0.031	2.09.E-01	8.76.E-01
Podoviridae	L7_Subdoligranulum_variabile	-0.030	0.036	4.13.E-01	9.18.E-01
Siphoviridae	L7_Subdoligranulum_variabile	-0.017	0.032	5.93.E-01	9.69.E-01
Autographiviridae	L7_Bacteroides_coprophilus	0.005	0.036	9.01.E-01	9.97.E-01
crAss_like_phage	L7_Bacteroides_coprophilus	-0.010	0.040	8.09.E-01	9.93.E-01
Herelleviridae	L7_Bacteroides_coprophilus	-0.062	0.038	1.00.E-01	7.79.E-01
Microviridae	L7_Bacteroides_coprophilus	-0.043	0.038	2.54.E-01	8.95.E-01
Myoviridae	L7_Bacteroides_coprophilus	0.060	0.042	1.55.E-01	8.26.E-01
Phycodnaviridae	L7_Bacteroides_coprophilus	-0.047	0.037	2.06.E-01	8.76.E-01
Podoviridae	L7_Bacteroides_coprophilus	0.011	0.044	8.09.E-01	9.93.E-01
Siphoviridae	L7_Bacteroides_coprophilus	0.023	0.039	5.57.E-01	9.61.E-01
Autographiviridae	L7_Bifidobacterium_ruminantium	0.009	0.031	7.84.E-01	9.92.E-01
crAss_like_phage	L7_Bifidobacterium_ruminantium	0.007	0.035	8.32.E-01	9.94.E-01
Herelleviridae	L7_Bifidobacterium_ruminantium	0.028	0.033	3.85.E-01	9.15.E-01
Microviridae	L7_Bifidobacterium_ruminantium	-0.040	0.033	2.23.E-01	8.81.E-01
Myoviridae	L7_Bifidobacterium_ruminantium	-0.013	0.037	7.18.E-01	9.86.E-01
Phycodnaviridae	L7_Bifidobacterium_ruminantium	0.027	0.032	4.00.E-01	9.15.E-01
Podoviridae	L7_Bifidobacterium_ruminantium	0.050	0.038	1.86.E-01	8.61.E-01
Siphoviridae	L7_Bifidobacterium_ruminantium	0.052	0.034	1.19.E-01	7.89.E-01
Autographiviridae	L7_Clostridium_scindens	-0.001	0.037	9.89.E-01	9.99.E-01
crAss_like_phage	L7_Clostridium_scindens	0.034	0.041	4.04.E-01	9.16.E-01
Herelleviridae	L7_Clostridium_scindens	-0.023	0.039	5.52.E-01	9.60.E-01
Microviridae	L7_Clostridium_scindens	-0.028	0.039	4.70.E-01	9.50.E-01
Myoviridae	L7_Clostridium_scindens	0.018	0.043	6.78.E-01	9.80.E-01
Phycodnaviridae	L7_Clostridium_scindens	0.040	0.038	2.94.E-01	9.15.E-01
Podoviridae	L7_Clostridium_scindens	-0.005	0.045	9.17.E-01	9.98.E-01
Siphoviridae	L7_Clostridium_scindens	0.022	0.040	5.87.E-01	9.66.E-01
Autographiviridae	L7_Corynebacterium_durum	-0.022	0.037	5.56.E-01	9.61.E-01
crAss_like_phage	L7_Corynebacterium_durum	0.010	0.041	7.99.E-01	9.92.E-01
Herelleviridae	L7_Corynebacterium_durum	0.004	0.038	9.08.E-01	9.98.E-01
Microviridae	L7_Corynebacterium_durum	-0.036	0.039	3.55.E-01	9.15.E-01
Myoviridae	L7_Corynebacterium_durum	-0.014	0.043	7.44.E-01	9.89.E-01
Phycodnaviridae	L7_Corynebacterium_durum	-0.048	0.038	2.10.E-01	8.76.E-01
Podoviridae	L7_Corynebacterium_durum	-0.050	0.044	2.65.E-01	9.01.E-01
Siphoviridae	L7_Corynebacterium_durum	0.003	0.040	9.33.E-01	9.98.E-01
Autographiviridae	L7_Gemella_morbillorum	0.003	0.035	9.25.E-01	9.98.E-01
crAss_like_phage	L7_Gemella_morbillorum	0.016	0.039	6.79.E-01	9.80.E-01
Herelleviridae	L7_Gemella_morbillorum	-0.106	0.036	3.71.E-03	4.02.E-01
Microviridae	L7_Gemella_morbillorum	0.049	0.037	1.87.E-01	8.61.E-01
Myoviridae	L7_Gemella_morbillorum	-0.041	0.041	3.13.E-01	9.15.E-01
Phycodnaviridae	L7_Gemella_morbillorum	-0.068	0.036	6.22.E-02	7.24.E-01
Podoviridae	L7_Gemella_morbillorum	0.087	0.042	3.90.E-02	7.03.E-01
Siphoviridae	L7_Gemella_morbillorum	0.003	0.038	9.35.E-01	9.98.E-01
Autographiviridae	L7_Bifidobacterium_pseudolongum	0.015	0.044	7.30.E-01	9.88.E-01
crAss_like_phage	L7_Bifidobacterium_pseudolongum	-0.077	0.048	1.11.E-01	7.82.E-01
Herelleviridae	L7_Bifidobacterium_pseudolongum	0.012	0.045	7.94.E-01	9.92.E-01
Microviridae	L7_Bifidobacterium_pseudolongum	-0.048	0.045	2.92.E-01	9.15.E-01
Myoviridae	L7_Bifidobacterium_pseudolongum	-0.033	0.051	5.15.E-01	9.57.E-01
Phycodnaviridae	L7_Bifidobacterium_pseudolongum	0.009	0.045	8.36.E-01	9.94.E-01
Podoviridae	L7_Bifidobacterium_pseudolongum	-0.045	0.052	3.89.E-01	9.15.E-01
Siphoviridae	L7_Bifidobacterium_pseudolongum	-0.030	0.047	5.14.E-01	9.57.E-01
Autographiviridae	L7_Streptococcus_vestibularis	-0.025	0.029	3.86.E-01	9.15.E-01
crAss_like_phage	L7_Streptococcus_vestibularis	-0.007	0.032	8.29.E-01	9.94.E-01
Herelleviridae	L7_Streptococcus_vestibularis	0.055	0.030	6.76.E-02	7.24.E-01
Microviridae	L7_Streptococcus_vestibularis	-0.026	0.030	3.88.E-01	9.15.E-01
Myoviridae	L7_Streptococcus_vestibularis	-0.025	0.033	4.55.E-01	9.39.E-01
Phycodnaviridae	L7_Streptococcus_vestibularis	0.002	0.030	9.52.E-01	9.98.E-01
Podoviridae	L7_Streptococcus_vestibularis	0.052	0.034	1.35.E-01	8.06.E-01
Siphoviridae	L7_Streptococcus_vestibularis	-0.004	0.031	9.05.E-01	9.97.E-01

Autographiviridae	L7_Lactobacillus_vaginalis	0.002	0.031	9.47.E-01	9.98.E-01
crAss_like_phage	L7_Lactobacillus_vaginalis	0.029	0.035	4.04.E-01	9.16.E-01
Herelleviridae	L7_Lactobacillus_vaginalis	-0.002	0.032	9.50.E-01	9.98.E-01
Microviridae	L7_Lactobacillus_vaginalis	-0.032	0.033	3.21.E-01	9.15.E-01
Myoviridae	L7_Lactobacillus_vaginalis	-0.016	0.036	6.54.E-01	9.79.E-01
Phycodnaviridae	L7_Lactobacillus_vaginalis	0.012	0.032	7.18.E-01	9.86.E-01
Podoviridae	L7_Lactobacillus_vaginalis	-0.021	0.037	5.79.E-01	9.64.E-01
Siphoviridae	L7_Lactobacillus_vaginalis	-0.003	0.033	9.37.E-01	9.98.E-01
Autographiviridae	L7_Bifidobacterium_thermacidophilum	0.017	0.031	5.82.E-01	9.64.E-01
crAss_like_phage	L7_Bifidobacterium_thermacidophilum	-0.036	0.035	2.95.E-01	9.15.E-01
Herelleviridae	L7_Bifidobacterium_thermacidophilum	0.048	0.032	1.44.E-01	8.12.E-01
Microviridae	L7_Bifidobacterium_thermacidophilum	-0.001	0.033	9.82.E-01	9.99.E-01
Myoviridae	L7_Bifidobacterium_thermacidophilum	-0.027	0.036	4.62.E-01	9.44.E-01
Phycodnaviridae	L7_Bifidobacterium_thermacidophilum	0.012	0.032	7.08.E-01	9.83.E-01
Podoviridae	L7_Bifidobacterium_thermacidophilum	0.001	0.038	9.80.E-01	9.99.E-01
Siphoviridae	L7_Bifidobacterium_thermacidophilum	0.025	0.033	4.51.E-01	9.38.E-01
Autographiviridae	L7_Porphyromonas_asaccharolytica	0.072	0.034	3.54.E-02	6.90.E-01
crAss_like_phage	L7_Porphyromonas_asaccharolytica	0.032	0.038	4.01.E-01	9.15.E-01
Herelleviridae	L7_Porphyromonas_asaccharolytica	0.006	0.036	8.64.E-01	9.96.E-01
Microviridae	L7_Porphyromonas_asaccharolytica	0.000	0.036	9.96.E-01	9.99.E-01
Myoviridae	L7_Porphyromonas_asaccharolytica	-0.018	0.040	6.43.E-01	9.79.E-01
Phycodnaviridae	L7_Porphyromonas_asaccharolytica	-0.016	0.035	6.43.E-01	9.79.E-01
Podoviridae	L7_Porphyromonas_asaccharolytica	-0.006	0.041	8.87.E-01	9.96.E-01
Siphoviridae	L7_Porphyromonas_asaccharolytica	-0.026	0.037	4.82.E-01	9.53.E-01
Autographiviridae	L7_Bacteroides_salanitronis	0.021	0.031	5.03.E-01	9.55.E-01
crAss_like_phage	L7_Bacteroides_salanitronis	-0.047	0.034	1.70.E-01	8.48.E-01
Herelleviridae	L7_Bacteroides_salanitronis	-0.011	0.032	7.25.E-01	9.87.E-01
Microviridae	L7_Bacteroides_salanitronis	0.036	0.032	2.60.E-01	8.95.E-01
Myoviridae	L7_Bacteroides_salanitronis	0.025	0.036	4.93.E-01	9.55.E-01
Phycodnaviridae	L7_Bacteroides_salanitronis	0.000	0.032	9.93.E-01	9.99.E-01
Podoviridae	L7_Bacteroides_salanitronis	-0.023	0.037	5.39.E-01	9.60.E-01
Siphoviridae	L7_Bacteroides_salanitronis	-0.056	0.033	8.99.E-02	7.58.E-01
Autographiviridae	L7_Riemerella_columbina	0.016	0.021	4.50.E-01	9.37.E-01
crAss_like_phage	L7_Riemerella_columbina	0.000	0.024	9.97.E-01	9.99.E-01
Herelleviridae	L7_Riemerella_columbina	-0.035	0.022	1.11.E-01	7.82.E-01
Microviridae	L7_Riemerella_columbina	-0.015	0.022	4.93.E-01	9.55.E-01
Myoviridae	L7_Riemerella_columbina	-0.010	0.025	6.94.E-01	9.81.E-01
Phycodnaviridae	L7_Riemerella_columbina	-0.006	0.022	8.02.E-01	9.92.E-01
Podoviridae	L7_Riemerella_columbina	0.035	0.026	1.71.E-01	8.50.E-01
Siphoviridae	L7_Riemerella_columbina	-0.013	0.023	5.62.E-01	9.61.E-01
Autographiviridae	L7_Coprobacillus_sp.	-0.005	0.041	9.06.E-01	9.97.E-01
crAss_like_phage	L7_Coprobacillus_sp.	-0.026	0.045	5.62.E-01	9.61.E-01
Herelleviridae	L7_Coprobacillus_sp.	-0.005	0.042	9.00.E-01	9.97.E-01
Microviridae	L7_Coprobacillus_sp.	0.058	0.042	1.74.E-01	8.56.E-01
Myoviridae	L7_Coprobacillus_sp.	0.017	0.047	7.27.E-01	9.87.E-01
Phycodnaviridae	L7_Coprobacillus_sp.	-0.005	0.042	9.02.E-01	9.97.E-01
Podoviridae	L7_Coprobacillus_sp.	-0.025	0.049	6.14.E-01	9.75.E-01
Siphoviridae	L7_Coprobacillus_sp.	0.009	0.043	8.44.E-01	9.95.E-01
Autographiviridae	L7_Succinatimonas_hippei	-0.003	0.041	9.33.E-01	9.98.E-01
crAss_like_phage	L7_Succinatimonas_hippei	0.008	0.046	8.66.E-01	9.96.E-01
Herelleviridae	L7_Succinatimonas_hippei	-0.031	0.043	4.75.E-01	9.52.E-01
Microviridae	L7_Succinatimonas_hippei	0.014	0.043	7.45.E-01	9.89.E-01
Myoviridae	L7_Succinatimonas_hippei	-0.012	0.048	8.03.E-01	9.93.E-01
Phycodnaviridae	L7_Succinatimonas_hippei	0.046	0.042	2.80.E-01	9.12.E-01
Podoviridae	L7_Succinatimonas_hippei	-0.019	0.050	7.02.E-01	9.81.E-01
Siphoviridae	L7_Succinatimonas_hippei	0.008	0.044	8.55.E-01	9.96.E-01
Autographiviridae	L7_Veillonella_atypica	-0.019	0.031	5.45.E-01	9.60.E-01
crAss_like_phage	L7_Veillonella_atypica	-0.064	0.035	6.69.E-02	7.24.E-01
Herelleviridae	L7_Veillonella_atypica	-0.040	0.033	2.24.E-01	8.82.E-01
Microviridae	L7_Veillonella_atypica	-0.030	0.033	3.53.E-01	9.15.E-01
Myoviridae	L7_Veillonella_atypica	0.010	0.037	7.78.E-01	9.92.E-01
Phycodnaviridae	L7_Veillonella_atypica	-0.041	0.032	2.09.E-01	8.76.E-01
Podoviridae	L7_Veillonella_atypica	-0.011	0.038	7.71.E-01	9.92.E-01
Siphoviridae	L7_Veillonella_atypica	-0.003	0.034	9.21.E-01	9.98.E-01
Autographiviridae	L7_Dakarella_massiliensis	-0.056	0.045	2.12.E-01	8.78.E-01

crAss_like_phage	L7_Dakarella_massiliensis	0.043	0.050	3.92.E-01	9.15.E-01
Herelleviridae	L7_Dakarella_massiliensis	-0.039	0.047	4.07.E-01	9.17.E-01
Microviridae	L7_Dakarella_massiliensis	-0.042	0.047	3.71.E-01	9.15.E-01
Myoviridae	L7_Dakarella_massiliensis	0.053	0.052	3.13.E-01	9.15.E-01
Phycodnaviridae	L7_Dakarella_massiliensis	0.036	0.046	4.29.E-01	9.25.E-01
Podoviridae	L7_Dakarella_massiliensis	-0.128	0.054	1.76.E-02	5.86.E-01
Siphoviridae	L7_Dakarella_massiliensis	0.000	0.048	9.93.E-01	9.99.E-01
Autographiviridae	L7_Bifidobacterium_pseudocatenulatum	0.013	0.033	6.95.E-01	9.81.E-01
crAss_like_phage	L7_Bifidobacterium_pseudocatenulatum	-0.009	0.036	8.04.E-01	9.93.E-01
Herelleviridae	L7_Bifidobacterium_pseudocatenulatum	0.008	0.034	8.22.E-01	9.93.E-01
Microviridae	L7_Bifidobacterium_pseudocatenulatum	0.058	0.034	8.81.E-02	7.54.E-01
Myoviridae	L7_Bifidobacterium_pseudocatenulatum	0.065	0.038	8.68.E-02	7.52.E-01
Phycodnaviridae	L7_Bifidobacterium_pseudocatenulatum	-0.001	0.034	9.81.E-01	9.99.E-01
Podoviridae	L7_Bifidobacterium_pseudocatenulatum	0.033	0.039	4.04.E-01	9.17.E-01
Siphoviridae	L7_Bifidobacterium_pseudocatenulatum	0.043	0.035	2.26.E-01	8.82.E-01
Autographiviridae	L7_Streptococcus_sobrinus	0.002	0.035	9.63.E-01	9.98.E-01
crAss_like_phage	L7_Streptococcus_sobrinus	0.005	0.039	8.89.E-01	9.96.E-01
Herelleviridae	L7_Streptococcus_sobrinus	0.029	0.037	4.32.E-01	9.25.E-01
Microviridae	L7_Streptococcus_sobrinus	0.018	0.037	6.16.E-01	9.75.E-01
Myoviridae	L7_Streptococcus_sobrinus	-0.031	0.041	4.56.E-01	9.39.E-01
Phycodnaviridae	L7_Streptococcus_sobrinus	0.025	0.036	4.99.E-01	9.55.E-01
Podoviridae	L7_Streptococcus_sobrinus	-0.062	0.042	1.41.E-01	8.10.E-01
Siphoviridae	L7_Streptococcus_sobrinus	-0.032	0.038	4.02.E-01	9.15.E-01
Autographiviridae	L7_Ruminococcus_champanellensis	0.013	0.027	6.34.E-01	9.76.E-01
crAss_like_phage	L7_Ruminococcus_champanellensis	0.026	0.030	3.84.E-01	9.15.E-01
Herelleviridae	L7_Ruminococcus_champanellensis	-0.014	0.028	6.09.E-01	9.75.E-01
Microviridae	L7_Ruminococcus_champanellensis	0.013	0.028	6.56.E-01	9.79.E-01
Myoviridae	L7_Ruminococcus_champanellensis	-0.015	0.031	6.28.E-01	9.76.E-01
Phycodnaviridae	L7_Ruminococcus_champanellensis	0.003	0.028	9.16.E-01	9.98.E-01
Podoviridae	L7_Ruminococcus_champanellensis	0.021	0.032	5.20.E-01	9.57.E-01
Siphoviridae	L7_Ruminococcus_champanellensis	-0.007	0.029	8.10.E-01	9.93.E-01
Autographiviridae	L7_Streptococcus_sp.	0.024	0.024	3.18.E-01	9.15.E-01
crAss_like_phage	L7_Streptococcus_sp.	0.043	0.027	1.06.E-01	7.82.E-01
Herelleviridae	L7_Streptococcus_sp.	0.016	0.025	5.24.E-01	9.57.E-01
Microviridae	L7_Streptococcus_sp.	-0.003	0.025	9.01.E-01	9.97.E-01
Myoviridae	L7_Streptococcus_sp.	0.023	0.028	4.07.E-01	9.17.E-01
Phycodnaviridae	L7_Streptococcus_sp.	0.032	0.025	1.90.E-01	8.61.E-01
Podoviridae	L7_Streptococcus_sp.	0.037	0.029	1.99.E-01	8.69.E-01
Siphoviridae	L7_Streptococcus_sp.	0.031	0.026	2.35.E-01	8.85.E-01
Autographiviridae	L7_Blautia_schinkii	-0.012	0.032	7.08.E-01	9.83.E-01
crAss_like_phage	L7_Blautia_schinkii	0.011	0.035	7.49.E-01	9.89.E-01
Herelleviridae	L7_Blautia_schinkii	-0.032	0.033	3.38.E-01	9.15.E-01
Microviridae	L7_Blautia_schinkii	-0.030	0.033	3.67.E-01	9.15.E-01
Myoviridae	L7_Blautia_schinkii	0.077	0.037	3.59.E-02	6.90.E-01
Phycodnaviridae	L7_Blautia_schinkii	-0.016	0.032	6.12.E-01	9.75.E-01
Podoviridae	L7_Blautia_schinkii	-0.087	0.038	2.13.E-02	6.06.E-01
Siphoviridae	L7_Blautia_schinkii	-0.034	0.034	3.10.E-01	9.15.E-01
Autographiviridae	L7_Fusobacterium_sp.	0.026	0.042	5.39.E-01	9.60.E-01
crAss_like_phage	L7_Fusobacterium_sp.	0.011	0.046	8.11.E-01	9.93.E-01
Herelleviridae	L7_Fusobacterium_sp.	0.030	0.043	4.94.E-01	9.55.E-01
Microviridae	L7_Fusobacterium_sp.	0.020	0.043	6.39.E-01	9.78.E-01
Myoviridae	L7_Fusobacterium_sp.	0.077	0.048	1.10.E-01	7.82.E-01
Phycodnaviridae	L7_Fusobacterium_sp.	0.076	0.043	7.34.E-02	7.26.E-01
Podoviridae	L7_Fusobacterium_sp.	0.092	0.050	6.41.E-02	7.24.E-01
Siphoviridae	L7_Fusobacterium_sp.	0.006	0.044	8.96.E-01	9.97.E-01
Autographiviridae	L7_Lachnotalea_sp.	-0.020	0.026	4.52.E-01	9.38.E-01
crAss_like_phage	L7_Lachnotalea_sp.	0.009	0.029	7.55.E-01	9.89.E-01
Herelleviridae	L7_Lachnotalea_sp.	0.039	0.027	1.49.E-01	8.22.E-01
Microviridae	L7_Lachnotalea_sp.	0.041	0.027	1.30.E-01	7.99.E-01
Myoviridae	L7_Lachnotalea_sp.	-0.004	0.030	8.87.E-01	9.96.E-01
Phycodnaviridae	L7_Lachnotalea_sp.	-0.023	0.027	4.01.E-01	9.15.E-01
Podoviridae	L7_Lachnotalea_sp.	-0.006	0.031	8.38.E-01	9.94.E-01
Siphoviridae	L7_Lachnotalea_sp.	-0.015	0.028	6.02.E-01	9.71.E-01
Autographiviridae	L7_Eubacterium_rectale	0.012	0.036	7.27.E-01	9.87.E-01
crAss_like_phage	L7_Eubacterium_rectale	-0.023	0.039	5.53.E-01	9.60.E-01

Herelleviridae	L7_Eubacterium_rectale	-0.006	0.037	8.78.E-01	9.96.E-01
Microviridae	L7_Eubacterium_rectale	-0.052	0.037	1.62.E-01	8.38.E-01
Myoviridae	L7_Eubacterium_rectale	-0.009	0.041	8.29.E-01	9.94.E-01
Phycodnaviridae	L7_Eubacterium_rectale	-0.015	0.037	6.88.E-01	9.80.E-01
Podoviridae	L7_Eubacterium_rectale	-0.026	0.043	5.45.E-01	9.60.E-01
Siphoviridae	L7_Eubacterium_rectale	-0.067	0.038	8.01.E-02	7.45.E-01
Autographiviridae	L7_Streptococcus_gordonii	0.017	0.031	5.83.E-01	9.64.E-01
crAss_like_phage	L7_Streptococcus_gordonii	0.023	0.034	5.04.E-01	9.55.E-01
Herelleviridae	L7_Streptococcus_gordonii	0.009	0.032	7.86.E-01	9.92.E-01
Microviridae	L7_Streptococcus_gordonii	-0.010	0.032	7.47.E-01	9.89.E-01
Myoviridae	L7_Streptococcus_gordonii	0.030	0.036	3.96.E-01	9.15.E-01
Phycodnaviridae	L7_Streptococcus_gordonii	0.068	0.032	3.17.E-02	6.75.E-01
Podoviridae	L7_Streptococcus_gordonii	-0.018	0.037	6.26.E-01	9.76.E-01
Siphoviridae	L7_Streptococcus_gordonii	0.026	0.033	4.28.E-01	9.25.E-01
Autographiviridae	L7_Bifidobacterium_kashiwanohense	0.006	0.030	8.43.E-01	9.95.E-01
crAss_like_phage	L7_Bifidobacterium_kashiwanohense	-0.023	0.033	4.86.E-01	9.54.E-01
Herelleviridae	L7_Bifidobacterium_kashiwanohense	-0.009	0.031	7.81.E-01	9.92.E-01
Microviridae	L7_Bifidobacterium_kashiwanohense	0.079	0.031	1.21.E-02	5.36.E-01
Myoviridae	L7_Bifidobacterium_kashiwanohense	0.052	0.035	1.33.E-01	8.05.E-01
Phycodnaviridae	L7_Bifidobacterium_kashiwanohense	-0.002	0.031	9.37.E-01	9.98.E-01
Podoviridae	L7_Bifidobacterium_kashiwanohense	0.082	0.036	2.36.E-02	6.29.E-01
Siphoviridae	L7_Bifidobacterium_kashiwanohense	0.011	0.032	7.31.E-01	9.88.E-01
Autographiviridae	L7_Megasphaera_sp.	-0.013	0.042	7.52.E-01	9.89.E-01
crAss_like_phage	L7_Megasphaera_sp.	0.040	0.046	3.82.E-01	9.15.E-01
Herelleviridae	L7_Megasphaera_sp.	-0.032	0.043	4.60.E-01	9.43.E-01
Microviridae	L7_Megasphaera_sp.	-0.033	0.044	4.51.E-01	9.38.E-01
Myoviridae	L7_Megasphaera_sp.	0.078	0.049	1.07.E-01	7.82.E-01
Phycodnaviridae	L7_Megasphaera_sp.	-0.085	0.043	4.81.E-02	7.22.E-01
Podoviridae	L7_Megasphaera_sp.	-0.071	0.050	1.60.E-01	8.38.E-01
Siphoviridae	L7_Megasphaera_sp.	0.034	0.045	4.53.E-01	9.38.E-01
Autographiviridae	L7_Clostridium_methylpentosum	-0.036	0.032	2.61.E-01	8.97.E-01
crAss_like_phage	L7_Clostridium_methylpentosum	-0.009	0.036	8.00.E-01	9.92.E-01
Herelleviridae	L7_Clostridium_methylpentosum	-0.076	0.034	2.47.E-02	6.45.E-01
Microviridae	L7_Clostridium_methylpentosum	0.057	0.034	9.15.E-02	7.59.E-01
Myoviridae	L7_Clostridium_methylpentosum	0.025	0.038	5.04.E-01	9.56.E-01
Phycodnaviridae	L7_Clostridium_methylpentosum	-0.029	0.033	3.89.E-01	9.15.E-01
Podoviridae	L7_Clostridium_methylpentosum	-0.040	0.039	3.08.E-01	9.15.E-01
Siphoviridae	L7_Clostridium_methylpentosum	0.035	0.035	3.20.E-01	9.15.E-01
Autographiviridae	L7_Parabacteroides_distasonis	-0.029	0.030	3.19.E-01	9.15.E-01
crAss_like_phage	L7_Parabacteroides_distasonis	0.027	0.033	4.05.E-01	9.17.E-01
Herelleviridae	L7_Parabacteroides_distasonis	0.025	0.031	4.19.E-01	9.19.E-01
Microviridae	L7_Parabacteroides_distasonis	-0.026	0.031	3.99.E-01	9.15.E-01
Myoviridae	L7_Parabacteroides_distasonis	-0.094	0.034	6.10.E-03	4.51.E-01
Phycodnaviridae	L7_Parabacteroides_distasonis	0.011	0.030	7.20.E-01	9.86.E-01
Podoviridae	L7_Parabacteroides_distasonis	0.021	0.036	5.46.E-01	9.60.E-01
Siphoviridae	L7_Parabacteroides_distasonis	0.017	0.032	5.82.E-01	9.64.E-01
Autographiviridae	L7_Peptoniphilus_sp.	-0.010	0.039	7.90.E-01	9.92.E-01
crAss_like_phage	L7_Peptoniphilus_sp.	-0.026	0.043	5.49.E-01	9.60.E-01
Herelleviridae	L7_Peptoniphilus_sp.	0.065	0.040	1.05.E-01	7.82.E-01
Microviridae	L7_Peptoniphilus_sp.	0.034	0.040	3.94.E-01	9.15.E-01
Myoviridae	L7_Peptoniphilus_sp.	-0.056	0.045	2.13.E-01	8.78.E-01
Phycodnaviridae	L7_Peptoniphilus_sp.	-0.046	0.040	2.45.E-01	8.91.E-01
Podoviridae	L7_Peptoniphilus_sp.	-0.046	0.046	3.26.E-01	9.15.E-01
Siphoviridae	L7_Peptoniphilus_sp.	-0.024	0.041	5.69.E-01	9.61.E-01
Autographiviridae	L7_Lactococcus_garvieae	0.049	0.039	2.16.E-01	8.79.E-01
crAss_like_phage	L7_Lactococcus_garvieae	-0.014	0.043	7.50.E-01	9.89.E-01
Herelleviridae	L7_Lactococcus_garvieae	0.034	0.041	4.07.E-01	9.17.E-01
Microviridae	L7_Lactococcus_garvieae	0.018	0.041	6.53.E-01	9.79.E-01
Myoviridae	L7_Lactococcus_garvieae	-0.018	0.046	6.90.E-01	9.80.E-01
Phycodnaviridae	L7_Lactococcus_garvieae	0.023	0.040	5.72.E-01	9.61.E-01
Podoviridae	L7_Lactococcus_garvieae	0.080	0.047	8.86.E-02	7.56.E-01
Siphoviridae	L7_Lactococcus_garvieae	-0.043	0.042	3.12.E-01	9.15.E-01
Autographiviridae	L7_Clostridium_saudiense	0.011	0.036	7.54.E-01	9.89.E-01
crAss_like_phage	L7_Clostridium_saudiense	0.009	0.039	8.21.E-01	9.93.E-01
Herelleviridae	L7_Clostridium_saudiense	0.049	0.037	1.88.E-01	8.61.E-01

Microviridae	L7_Clostridium_saudiense	0.007	0.037	8.48.E-01	9.95.E-01
Myoviridae	L7_Clostridium_saudiense	-0.020	0.041	6.24.E-01	9.76.E-01
Phycodnaviridae	L7_Clostridium_saudiense	-0.008	0.037	8.17.E-01	9.93.E-01
Podoviridae	L7_Clostridium_saudiense	0.003	0.043	9.53.E-01	9.98.E-01
Siphoviridae	L7_Clostridium_saudiense	-0.026	0.038	4.96.E-01	9.55.E-01
Autographiviridae	L7_Lactobacillus_iners	0.096	0.040	1.72.E-02	5.86.E-01
crAss_like_phage	L7_Lactobacillus_iners	-0.038	0.044	3.95.E-01	9.15.E-01
Herelleviridae	L7_Lactobacillus_iners	-0.017	0.042	6.91.E-01	9.80.E-01
Microviridae	L7_Lactobacillus_iners	0.033	0.042	4.36.E-01	9.26.E-01
Myoviridae	L7_Lactobacillus_iners	0.028	0.047	5.49.E-01	9.60.E-01
Phycodnaviridae	L7_Lactobacillus_iners	0.010	0.041	8.08.E-01	9.93.E-01
Podoviridae	L7_Lactobacillus_iners	0.061	0.048	2.10.E-01	8.76.E-01
Siphoviridae	L7_Lactobacillus_iners	-0.036	0.043	4.08.E-01	9.17.E-01
Autographiviridae	L7_Lactococcus_lactis	0.057	0.041	1.63.E-01	8.38.E-01
crAss_like_phage	L7_Lactococcus_lactis	-0.020	0.045	6.63.E-01	9.79.E-01
Herelleviridae	L7_Lactococcus_lactis	-0.003	0.042	9.51.E-01	9.98.E-01
Microviridae	L7_Lactococcus_lactis	0.039	0.042	3.57.E-01	9.15.E-01
Myoviridae	L7_Lactococcus_lactis	0.023	0.047	6.34.E-01	9.76.E-01
Phycodnaviridae	L7_Lactococcus_lactis	-0.044	0.042	2.95.E-01	9.15.E-01
Podoviridae	L7_Lactococcus_lactis	0.020	0.049	6.87.E-01	9.80.E-01
Siphoviridae	L7_Lactococcus_lactis	-0.010	0.044	8.12.E-01	9.93.E-01
Autographiviridae	L7_Bilophila_wadsworthia	0.056	0.037	1.31.E-01	8.00.E-01
crAss_like_phage	L7_Bilophila_wadsworthia	-0.026	0.041	5.24.E-01	9.57.E-01
Herelleviridae	L7_Bilophila_wadsworthia	-0.025	0.039	5.16.E-01	9.57.E-01
Microviridae	L7_Bilophila_wadsworthia	0.031	0.039	4.24.E-01	9.23.E-01
Myoviridae	L7_Bilophila_wadsworthia	0.045	0.043	3.02.E-01	9.15.E-01
Phycodnaviridae	L7_Bilophila_wadsworthia	-0.003	0.038	9.31.E-01	9.98.E-01
Podoviridae	L7_Bilophila_wadsworthia	0.008	0.045	8.67.E-01	9.96.E-01
Siphoviridae	L7_Bilophila_wadsworthia	-0.001	0.040	9.89.E-01	9.99.E-01
Autographiviridae	L7_Ruminococcus_callidus	0.039	0.034	2.55.E-01	8.95.E-01
crAss_like_phage	L7_Ruminococcus_callidus	0.038	0.038	3.17.E-01	9.15.E-01
Herelleviridae	L7_Ruminococcus_callidus	0.016	0.035	6.43.E-01	9.79.E-01
Microviridae	L7_Ruminococcus_callidus	-0.007	0.035	8.43.E-01	9.95.E-01
Myoviridae	L7_Ruminococcus_callidus	0.034	0.039	3.95.E-01	9.15.E-01
Phycodnaviridae	L7_Ruminococcus_callidus	0.080	0.035	2.24.E-02	6.19.E-01
Podoviridae	L7_Ruminococcus_callidus	0.038	0.041	3.56.E-01	9.15.E-01
Siphoviridae	L7_Ruminococcus_callidus	0.098	0.036	6.84.E-03	4.51.E-01
Autographiviridae	L7_Enterococcus_faecalis	0.080	0.040	4.55.E-02	7.22.E-01
crAss_like_phage	L7_Enterococcus_faecalis	-0.045	0.044	3.15.E-01	9.15.E-01
Herelleviridae	L7_Enterococcus_faecalis	0.002	0.042	9.53.E-01	9.98.E-01
Microviridae	L7_Enterococcus_faecalis	0.018	0.042	6.63.E-01	9.79.E-01
Myoviridae	L7_Enterococcus_faecalis	0.161	0.046	5.45.E-04	2.50.E-01
Phycodnaviridae	L7_Enterococcus_faecalis	0.024	0.041	5.57.E-01	9.61.E-01
Podoviridae	L7_Enterococcus_faecalis	0.064	0.048	1.82.E-01	8.60.E-01
Siphoviridae	L7_Enterococcus_faecalis	-0.028	0.043	5.21.E-01	9.57.E-01
Autographiviridae	L7_Megamonas_hypermegale	-0.040	0.037	2.83.E-01	9.13.E-01
crAss_like_phage	L7_Megamonas_hypermegale	-0.015	0.041	7.16.E-01	9.86.E-01
Herelleviridae	L7_Megamonas_hypermegale	0.001	0.039	9.86.E-01	9.99.E-01
Microviridae	L7_Megamonas_hypermegale	-0.022	0.039	5.71.E-01	9.61.E-01
Myoviridae	L7_Megamonas_hypermegale	0.065	0.043	1.31.E-01	8.00.E-01
Phycodnaviridae	L7_Megamonas_hypermegale	-0.030	0.038	4.31.E-01	9.25.E-01
Podoviridae	L7_Megamonas_hypermegale	-0.020	0.045	6.50.E-01	9.79.E-01
Siphoviridae	L7_Megamonas_hypermegale	-0.014	0.040	7.20.E-01	9.86.E-01
Autographiviridae	L7_Bacteroides_timonensis	-0.092	0.036	1.13.E-02	5.36.E-01
crAss_like_phage	L7_Bacteroides_timonensis	-0.009	0.040	8.15.E-01	9.93.E-01
Herelleviridae	L7_Bacteroides_timonensis	0.080	0.037	3.36.E-02	6.82.E-01
Microviridae	L7_Bacteroides_timonensis	0.003	0.038	9.26.E-01	9.98.E-01
Myoviridae	L7_Bacteroides_timonensis	-0.031	0.042	4.62.E-01	9.44.E-01
Phycodnaviridae	L7_Bacteroides_timonensis	0.059	0.037	1.11.E-01	7.82.E-01
Podoviridae	L7_Bacteroides_timonensis	-0.021	0.043	6.23.E-01	9.76.E-01
Siphoviridae	L7_Bacteroides_timonensis	0.004	0.039	9.27.E-01	9.98.E-01
Autographiviridae	L7_Clostridioides_difficile	0.005	0.026	8.54.E-01	9.95.E-01
crAss_like_phage	L7_Clostridioides_difficile	-0.022	0.028	4.33.E-01	9.26.E-01
Herelleviridae	L7_Clostridioides_difficile	0.030	0.027	2.56.E-01	8.95.E-01
Microviridae	L7_Clostridioides_difficile	-0.056	0.027	3.64.E-02	6.90.E-01

Myoviridae	L7_Clostridioides_difficile	0.019	0.030	5.23.E-01	9.57.E-01
Phycodnaviridae	L7_Clostridioides_difficile	0.003	0.026	8.99.E-01	9.97.E-01
Podoviridae	L7_Clostridioides_difficile	-0.009	0.031	7.65.E-01	9.91.E-01
Siphoviridae	L7_Clostridioides_difficile	-0.002	0.028	9.51.E-01	9.98.E-01
Autographiviridae	L7_Gemella_sanguinis	-0.058	0.029	4.81.E-02	7.22.E-01
crAss_like_phage	L7_Gemella_sanguinis	0.037	0.033	2.57.E-01	8.95.E-01
Herelleviridae	L7_Gemella_sanguinis	-0.009	0.031	7.80.E-01	9.92.E-01
Microviridae	L7_Gemella_sanguinis	-0.010	0.031	7.39.E-01	9.88.E-01
Myoviridae	L7_Gemella_sanguinis	-0.018	0.034	5.96.E-01	9.69.E-01
Phycodnaviridae	L7_Gemella_sanguinis	0.018	0.030	5.44.E-01	9.60.E-01
Podoviridae	L7_Gemella_sanguinis	0.005	0.035	8.84.E-01	9.96.E-01
Siphoviridae	L7_Gemella_sanguinis	0.063	0.031	4.60.E-02	7.22.E-01
Autographiviridae	L7_Streptococcus_australis	0.033	0.030	2.81.E-01	9.13.E-01
crAss_like_phage	L7_Streptococcus_australis	0.050	0.033	1.34.E-01	8.06.E-01
Herelleviridae	L7_Streptococcus_australis	-0.002	0.031	9.48.E-01	9.98.E-01
Microviridae	L7_Streptococcus_australis	-0.013	0.032	6.71.E-01	9.80.E-01
Myoviridae	L7_Streptococcus_australis	0.082	0.035	1.91.E-02	5.90.E-01
Phycodnaviridae	L7_Streptococcus_australis	0.014	0.031	6.61.E-01	9.79.E-01
Podoviridae	L7_Streptococcus_australis	0.015	0.036	6.81.E-01	9.80.E-01
Siphoviridae	L7_Streptococcus_australis	0.083	0.032	1.04.E-02	5.13.E-01
Autographiviridae	L7_Lachnospira_pectinoschiza	0.000	0.039	9.96.E-01	9.99.E-01
crAss_like_phage	L7_Lachnospira_pectinoschiza	-0.067	0.044	1.25.E-01	7.94.E-01
Herelleviridae	L7_Lachnospira_pectinoschiza	-0.004	0.041	9.31.E-01	9.98.E-01
Microviridae	L7_Lachnospira_pectinoschiza	-0.044	0.041	2.89.E-01	9.15.E-01
Myoviridae	L7_Lachnospira_pectinoschiza	-0.088	0.046	5.35.E-02	7.22.E-01
Phycodnaviridae	L7_Lachnospira_pectinoschiza	-0.054	0.040	1.84.E-01	8.60.E-01
Podoviridae	L7_Lachnospira_pectinoschiza	-0.084	0.047	7.50.E-02	7.30.E-01
Siphoviridae	L7_Lachnospira_pectinoschiza	-0.051	0.042	2.28.E-01	8.82.E-01
Autographiviridae	L7_Alistipes_shahii	0.031	0.027	2.54.E-01	8.95.E-01
crAss_like_phage	L7_Alistipes_shahii	0.050	0.030	9.24.E-02	7.60.E-01
Herelleviridae	L7_Alistipes_shahii	0.081	0.028	3.58.E-03	4.02.E-01
Microviridae	L7_Alistipes_shahii	0.016	0.028	5.74.E-01	9.62.E-01
Myoviridae	L7_Alistipes_shahii	-0.020	0.031	5.15.E-01	9.57.E-01
Phycodnaviridae	L7_Alistipes_shahii	-0.006	0.028	8.29.E-01	9.94.E-01
Podoviridae	L7_Alistipes_shahii	0.048	0.032	1.39.E-01	8.10.E-01
Siphoviridae	L7_Alistipes_shahii	0.048	0.029	9.51.E-02	7.64.E-01
Autographiviridae	L7_Streptococcus_parasanguinis	0.015	0.025	5.59.E-01	9.61.E-01
crAss_like_phage	L7_Streptococcus_parasanguinis	0.065	0.027	1.92.E-02	5.90.E-01
Herelleviridae	L7_Streptococcus_parasanguinis	-0.005	0.026	8.34.E-01	9.94.E-01
Microviridae	L7_Streptococcus_parasanguinis	-0.022	0.026	3.90.E-01	9.15.E-01
Myoviridae	L7_Streptococcus_parasanguinis	0.073	0.029	1.20.E-02	5.36.E-01
Phycodnaviridae	L7_Streptococcus_parasanguinis	0.022	0.026	3.95.E-01	9.15.E-01
Podoviridae	L7_Streptococcus_parasanguinis	0.026	0.030	3.83.E-01	9.15.E-01
Siphoviridae	L7_Streptococcus_parasanguinis	0.036	0.027	1.75.E-01	8.56.E-01
Autographiviridae	L7_Erysipelotrichaceae_bacterium	0.023	0.033	4.77.E-01	9.52.E-01
crAss_like_phage	L7_Erysipelotrichaceae_bacterium	-0.008	0.036	8.23.E-01	9.93.E-01
Herelleviridae	L7_Erysipelotrichaceae_bacterium	-0.033	0.034	3.30.E-01	9.15.E-01
Microviridae	L7_Erysipelotrichaceae_bacterium	0.027	0.034	4.26.E-01	9.24.E-01
Myoviridae	L7_Erysipelotrichaceae_bacterium	-0.004	0.038	9.06.E-01	9.97.E-01
Phycodnaviridae	L7_Erysipelotrichaceae_bacterium	0.003	0.033	9.33.E-01	9.98.E-01
Podoviridae	L7_Erysipelotrichaceae_bacterium	-0.063	0.039	1.06.E-01	7.82.E-01
Siphoviridae	L7_Erysipelotrichaceae_bacterium	0.016	0.035	6.55.E-01	9.79.E-01
Autographiviridae	L7_Actinomyces_viscosus	-0.011	0.036	7.55.E-01	9.89.E-01
crAss_like_phage	L7_Actinomyces_viscosus	0.011	0.040	7.85.E-01	9.92.E-01
Herelleviridae	L7_Actinomyces_viscosus	-0.062	0.037	9.64.E-02	7.70.E-01
Microviridae	L7_Actinomyces_viscosus	-0.054	0.038	1.51.E-01	8.22.E-01
Myoviridae	L7_Actinomyces_viscosus	-0.013	0.042	7.64.E-01	9.91.E-01
Phycodnaviridae	L7_Actinomyces_viscosus	0.011	0.037	7.76.E-01	9.92.E-01
Podoviridae	L7_Actinomyces_viscosus	-0.045	0.043	2.94.E-01	9.15.E-01
Siphoviridae	L7_Actinomyces_viscosus	-0.033	0.039	3.87.E-01	9.15.E-01
Autographiviridae	L7_Faecalibacterium_cf.	-0.047	0.032	1.43.E-01	8.12.E-01
crAss_like_phage	L7_Faecalibacterium_cf.	0.011	0.036	7.53.E-01	9.89.E-01
Herelleviridae	L7_Faecalibacterium_cf.	-0.005	0.034	8.80.E-01	9.96.E-01
Microviridae	L7_Faecalibacterium_cf.	0.023	0.034	5.03.E-01	9.55.E-01
Myoviridae	L7_Faecalibacterium_cf.	0.123	0.037	1.04.E-03	2.79.E-01

Phycodnaviridae	L7_Faecalibacterium_cf.	-0.020	0.033	5.41.E-01	9.60.E-01
Podoviridae	L7_Faecalibacterium_cf.	0.186	0.038	1.31.E-06	4.20.E-03
Siphoviridae	L7_Faecalibacterium_cf.	0.019	0.035	5.85.E-01	9.65.E-01
Autographiviridae	L7_Fusicatenibacter_saccharivorans	-0.033	0.033	3.19.E-01	9.15.E-01
crAss_like_phage	L7_Fusicatenibacter_saccharivorans	-0.075	0.037	4.21.E-02	7.16.E-01
Herelleviridae	L7_Fusicatenibacter_saccharivorans	-0.029	0.035	3.98.E-01	9.15.E-01
Microviridae	L7_Fusicatenibacter_saccharivorans	-0.010	0.035	7.85.E-01	9.92.E-01
Myoviridae	L7_Fusicatenibacter_saccharivorans	-0.015	0.039	6.94.E-01	9.81.E-01
Phycodnaviridae	L7_Fusicatenibacter_saccharivorans	0.012	0.034	7.27.E-01	9.87.E-01
Podoviridae	L7_Fusicatenibacter_saccharivorans	0.025	0.040	5.28.E-01	9.57.E-01
Siphoviridae	L7_Fusicatenibacter_saccharivorans	0.008	0.036	8.21.E-01	9.93.E-01
Autographiviridae	L7_Alistipes_finegoldii	-0.011	0.033	7.38.E-01	9.88.E-01
crAss_like_phage	L7_Alistipes_finegoldii	0.067	0.036	6.69.E-02	7.24.E-01
Herelleviridae	L7_Alistipes_finegoldii	0.065	0.034	5.79.E-02	7.24.E-01
Microviridae	L7_Alistipes_finegoldii	0.017	0.034	6.19.E-01	9.75.E-01
Myoviridae	L7_Alistipes_finegoldii	-0.017	0.038	6.58.E-01	9.79.E-01
Phycodnaviridae	L7_Alistipes_finegoldii	-0.005	0.034	8.76.E-01	9.96.E-01
Podoviridae	L7_Alistipes_finegoldii	0.060	0.039	1.28.E-01	7.99.E-01
Siphoviridae	L7_Alistipes_finegoldii	0.017	0.035	6.32.E-01	9.76.E-01
Autographiviridae	L7_Firmicutes_bacterium	-0.024	0.024	3.27.E-01	9.15.E-01
crAss_like_phage	L7_Firmicutes_bacterium	0.032	0.027	2.29.E-01	8.82.E-01
Herelleviridae	L7_Firmicutes_bacterium	0.001	0.025	9.83.E-01	9.99.E-01
Microviridae	L7_Firmicutes_bacterium	0.023	0.025	3.74.E-01	9.15.E-01
Myoviridae	L7_Firmicutes_bacterium	-0.019	0.028	4.98.E-01	9.55.E-01
Phycodnaviridae	L7_Firmicutes_bacterium	0.030	0.025	2.31.E-01	8.82.E-01
Podoviridae	L7_Firmicutes_bacterium	0.008	0.029	7.78.E-01	9.92.E-01
Siphoviridae	L7_Firmicutes_bacterium	0.007	0.026	7.90.E-01	9.92.E-01
Autographiviridae	L7_Veillonella_sp.	-0.001	0.034	9.76.E-01	9.99.E-01
crAss_like_phage	L7_Veillonella_sp.	-0.037	0.038	3.35.E-01	9.15.E-01
Herelleviridae	L7_Veillonella_sp.	-0.086	0.036	1.57.E-02	5.77.E-01
Microviridae	L7_Veillonella_sp.	-0.036	0.036	3.22.E-01	9.15.E-01
Myoviridae	L7_Veillonella_sp.	-0.068	0.040	8.70.E-02	7.52.E-01
Phycodnaviridae	L7_Veillonella_sp.	-0.042	0.035	2.32.E-01	8.82.E-01
Podoviridae	L7_Veillonella_sp.	-0.113	0.041	6.09.E-03	4.51.E-01
Siphoviridae	L7_Veillonella_sp.	-0.039	0.037	2.87.E-01	9.15.E-01
Autographiviridae	L7_Pseudomonas_oryzihabitans	-0.031	0.037	4.13.E-01	9.18.E-01
crAss_like_phage	L7_Pseudomonas_oryzihabitans	-0.080	0.041	5.27.E-02	7.22.E-01
Herelleviridae	L7_Pseudomonas_oryzihabitans	-0.037	0.039	3.40.E-01	9.15.E-01
Microviridae	L7_Pseudomonas_oryzihabitans	0.055	0.039	1.58.E-01	8.35.E-01
Myoviridae	L7_Pseudomonas_oryzihabitans	0.026	0.044	5.43.E-01	9.60.E-01
Phycodnaviridae	L7_Pseudomonas_oryzihabitans	0.044	0.038	2.58.E-01	8.95.E-01
Podoviridae	L7_Pseudomonas_oryzihabitans	0.020	0.045	6.54.E-01	9.79.E-01
Siphoviridae	L7_Pseudomonas_oryzihabitans	0.013	0.040	7.47.E-01	9.89.E-01
Autographiviridae	L7_Solobacterium_moorei	0.004	0.039	9.15.E-01	9.98.E-01
crAss_like_phage	L7_Solobacterium_moorei	0.031	0.043	4.74.E-01	9.52.E-01
Herelleviridae	L7_Solobacterium_moorei	-0.010	0.041	8.11.E-01	9.93.E-01
Microviridae	L7_Solobacterium_moorei	0.144	0.040	4.01.E-04	2.15.E-01
Myoviridae	L7_Solobacterium_moorei	0.069	0.046	1.29.E-01	7.99.E-01
Phycodnaviridae	L7_Solobacterium_moorei	0.026	0.040	5.21.E-01	9.57.E-01
Podoviridae	L7_Solobacterium_moorei	-0.009	0.047	8.57.E-01	9.96.E-01
Siphoviridae	L7_Solobacterium_moorei	-0.009	0.042	8.25.E-01	9.93.E-01
Autographiviridae	L7_Coprococcus_catus	0.015	0.034	6.68.E-01	9.80.E-01
crAss_like_phage	L7_Coprococcus_catus	0.016	0.038	6.70.E-01	9.80.E-01
Herelleviridae	L7_Coprococcus_catus	0.031	0.036	3.76.E-01	9.15.E-01
Microviridae	L7_Coprococcus_catus	-0.016	0.036	6.56.E-01	9.79.E-01
Myoviridae	L7_Coprococcus_catus	-0.089	0.040	2.53.E-02	6.50.E-01
Phycodnaviridae	L7_Coprococcus_catus	0.026	0.035	4.56.E-01	9.39.E-01
Podoviridae	L7_Coprococcus_catus	-0.036	0.041	3.81.E-01	9.15.E-01
Siphoviridae	L7_Coprococcus_catus	-0.051	0.036	1.63.E-01	8.38.E-01
Autographiviridae	L7_Escherichia_coli	0.023	0.032	4.76.E-01	9.52.E-01
crAss_like_phage	L7_Escherichia_coli	0.017	0.035	6.20.E-01	9.75.E-01
Herelleviridae	L7_Escherichia_coli	0.012	0.033	7.24.E-01	9.86.E-01
Microviridae	L7_Escherichia_coli	0.002	0.033	9.50.E-01	9.98.E-01
Myoviridae	L7_Escherichia_coli	0.058	0.037	1.14.E-01	7.85.E-01
Phycodnaviridae	L7_Escherichia_coli	-0.002	0.033	9.47.E-01	9.98.E-01

Podoviridae	L7_Escherichia_coli	-0.056	0.038	1.44.E-01	8.12.E-01
Siphoviridae	L7_Escherichia_coli	0.030	0.034	3.75.E-01	9.15.E-01
Autographiviridae	L7_Lactobacillus_acidophilus	-0.027	0.039	4.91.E-01	9.55.E-01
crAss_like_phage	L7_Lactobacillus_acidophilus	-0.077	0.043	7.12.E-02	7.24.E-01
Herelleviridae	L7_Lactobacillus_acidophilus	-0.032	0.040	4.23.E-01	9.23.E-01
Microviridae	L7_Lactobacillus_acidophilus	-0.060	0.040	1.34.E-01	8.06.E-01
Myoviridae	L7_Lactobacillus_acidophilus	-0.060	0.045	1.80.E-01	8.60.E-01
Phycodnaviridae	L7_Lactobacillus_acidophilus	-0.006	0.040	8.88.E-01	9.96.E-01
Podoviridae	L7_Lactobacillus_acidophilus	0.028	0.046	5.41.E-01	9.60.E-01
Siphoviridae	L7_Lactobacillus_acidophilus	0.032	0.041	4.43.E-01	9.32.E-01
Autographiviridae	L7_Massilioclostridium_coli	0.013	0.037	7.26.E-01	9.87.E-01
crAss_like_phage	L7_Massilioclostridium_coli	0.067	0.041	1.01.E-01	7.79.E-01
Herelleviridae	L7_Massilioclostridium_coli	-0.007	0.039	8.48.E-01	9.95.E-01
Microviridae	L7_Massilioclostridium_coli	-0.037	0.039	3.36.E-01	9.15.E-01
Myoviridae	L7_Massilioclostridium_coli	0.017	0.043	6.90.E-01	9.80.E-01
Phycodnaviridae	L7_Massilioclostridium_coli	-0.024	0.038	5.29.E-01	9.57.E-01
Podoviridae	L7_Massilioclostridium_coli	0.040	0.044	3.67.E-01	9.15.E-01
Siphoviridae	L7_Massilioclostridium_coli	0.042	0.040	2.85.E-01	9.15.E-01
Autographiviridae	L7_Ruminococcus_gnavus	-0.029	0.032	3.66.E-01	9.15.E-01
crAss_like_phage	L7_Ruminococcus_gnavus	-0.035	0.035	3.21.E-01	9.15.E-01
Herelleviridae	L7_Ruminococcus_gnavus	-0.034	0.033	3.02.E-01	9.15.E-01
Microviridae	L7_Ruminococcus_gnavus	0.016	0.033	6.31.E-01	9.76.E-01
Myoviridae	L7_Ruminococcus_gnavus	-0.059	0.037	1.10.E-01	7.82.E-01
Phycodnaviridae	L7_Ruminococcus_gnavus	-0.001	0.032	9.76.E-01	9.99.E-01
Podoviridae	L7_Ruminococcus_gnavus	-0.060	0.038	1.11.E-01	7.82.E-01
Siphoviridae	L7_Ruminococcus_gnavus	0.030	0.034	3.70.E-01	9.15.E-01
Autographiviridae	L7_Veillonella_dispar	0.003	0.030	9.10.E-01	9.98.E-01
crAss_like_phage	L7_Veillonella_dispar	-0.088	0.033	7.30.E-03	4.64.E-01
Herelleviridae	L7_Veillonella_dispar	-0.049	0.031	1.12.E-01	7.83.E-01
Microviridae	L7_Veillonella_dispar	-0.006	0.031	8.37.E-01	9.94.E-01
Myoviridae	L7_Veillonella_dispar	0.021	0.035	5.48.E-01	9.60.E-01
Phycodnaviridae	L7_Veillonella_dispar	0.019	0.031	5.39.E-01	9.60.E-01
Podoviridae	L7_Veillonella_dispar	-0.039	0.036	2.68.E-01	9.01.E-01
Siphoviridae	L7_Veillonella_dispar	-0.009	0.032	7.88.E-01	9.92.E-01
Autographiviridae	L7_Eisenbergiella_massiliensis	-0.016	0.035	6.34.E-01	9.76.E-01
crAss_like_phage	L7_Eisenbergiella_massiliensis	-0.015	0.038	7.03.E-01	9.81.E-01
Herelleviridae	L7_Eisenbergiella_massiliensis	0.022	0.036	5.43.E-01	9.60.E-01
Microviridae	L7_Eisenbergiella_massiliensis	0.023	0.036	5.14.E-01	9.57.E-01
Myoviridae	L7_Eisenbergiella_massiliensis	0.039	0.040	3.27.E-01	9.15.E-01
Phycodnaviridae	L7_Eisenbergiella_massiliensis	0.046	0.035	1.98.E-01	8.68.E-01
Podoviridae	L7_Eisenbergiella_massiliensis	0.028	0.041	5.00.E-01	9.55.E-01
Siphoviridae	L7_Eisenbergiella_massiliensis	0.002	0.037	9.54.E-01	9.98.E-01
Autographiviridae	L7_Propionibacterium_acidifaciens	-0.028	0.041	4.93.E-01	9.55.E-01
crAss_like_phage	L7_Propionibacterium_acidifaciens	0.018	0.045	6.92.E-01	9.81.E-01
Herelleviridae	L7_Propionibacterium_acidifaciens	0.046	0.043	2.77.E-01	9.05.E-01
Microviridae	L7_Propionibacterium_acidifaciens	-0.018	0.043	6.83.E-01	9.80.E-01
Myoviridae	L7_Propionibacterium_acidifaciens	-0.050	0.048	2.91.E-01	9.15.E-01
Phycodnaviridae	L7_Propionibacterium_acidifaciens	-0.008	0.042	8.46.E-01	9.95.E-01
Podoviridae	L7_Propionibacterium_acidifaciens	-0.049	0.049	3.17.E-01	9.15.E-01
Siphoviridae	L7_Propionibacterium_acidifaciens	-0.023	0.044	5.99.E-01	9.70.E-01
Autographiviridae	L7_Enorma_massiliensis	-0.030	0.039	4.46.E-01	9.34.E-01
crAss_like_phage	L7_Enorma_massiliensis	-0.056	0.043	1.95.E-01	8.66.E-01
Herelleviridae	L7_Enorma_massiliensis	-0.054	0.041	1.90.E-01	8.61.E-01
Microviridae	L7_Enorma_massiliensis	-0.017	0.041	6.80.E-01	9.80.E-01
Myoviridae	L7_Enorma_massiliensis	0.006	0.046	9.02.E-01	9.97.E-01
Phycodnaviridae	L7_Enorma_massiliensis	-0.075	0.040	6.14.E-02	7.24.E-01
Podoviridae	L7_Enorma_massiliensis	0.040	0.047	3.95.E-01	9.15.E-01
Siphoviridae	L7_Enorma_massiliensis	-0.106	0.042	1.15.E-02	5.36.E-01
Autographiviridae	L7_Anaerotruncus_rubiinfantis	-0.096	0.029	9.91.E-04	2.79.E-01
crAss_like_phage	L7_Anaerotruncus_rubiinfantis	0.017	0.033	6.09.E-01	9.75.E-01
Herelleviridae	L7_Anaerotruncus_rubiinfantis	-0.041	0.031	1.84.E-01	8.60.E-01
Microviridae	L7_Anaerotruncus_rubiinfantis	0.007	0.031	8.25.E-01	9.93.E-01
Myoviridae	L7_Anaerotruncus_rubiinfantis	0.033	0.034	3.31.E-01	9.15.E-01
Phycodnaviridae	L7_Anaerotruncus_rubiinfantis	0.003	0.030	9.26.E-01	9.98.E-01
Podoviridae	L7_Anaerotruncus_rubiinfantis	-0.074	0.035	3.65.E-02	6.90.E-01

Siphoviridae	L7_Anaerotruncus_rubiinfantis	0.038	0.031	2.26.E-01	8.82.E-01
Autographiviridae	L7_Proteobacteria_bacterium	-0.012	0.039	7.63.E-01	9.91.E-01
crAss_like_phage	L7_Proteobacteria_bacterium	0.030	0.043	4.86.E-01	9.54.E-01
Herelleviridae	L7_Proteobacteria_bacterium	0.046	0.040	2.55.E-01	8.95.E-01
Microviridae	L7_Proteobacteria_bacterium	-0.060	0.041	1.41.E-01	8.10.E-01
Myoviridae	L7_Proteobacteria_bacterium	0.012	0.045	7.83.E-01	9.92.E-01
Phycodnaviridae	L7_Proteobacteria_bacterium	0.008	0.040	8.51.E-01	9.95.E-01
Podoviridae	L7_Proteobacteria_bacterium	-0.126	0.046	6.84.E-03	4.51.E-01
Siphoviridae	L7_Proteobacteria_bacterium	-0.008	0.042	8.52.E-01	9.95.E-01
Autographiviridae	L7_Porphyromonas_uenonis	0.093	0.035	8.36.E-03	4.75.E-01
crAss_like_phage	L7_Porphyromonas_uenonis	0.016	0.039	6.75.E-01	9.80.E-01
Herelleviridae	L7_Porphyromonas_uenonis	0.030	0.037	4.10.E-01	9.17.E-01
Microviridae	L7_Porphyromonas_uenonis	0.043	0.037	2.41.E-01	8.85.E-01
Myoviridae	L7_Porphyromonas_uenonis	-0.027	0.041	5.11.E-01	9.57.E-01
Phycodnaviridae	L7_Porphyromonas_uenonis	-0.042	0.036	2.52.E-01	8.95.E-01
Podoviridae	L7_Porphyromonas_uenonis	-0.004	0.042	9.22.E-01	9.98.E-01
Siphoviridae	L7_Porphyromonas_uenonis	0.006	0.038	8.66.E-01	9.96.E-01
Autographiviridae	L7_Delftia_acidovorans	-0.016	0.020	4.34.E-01	9.26.E-01
crAss_like_phage	L7_Delftia_acidovorans	-0.047	0.022	3.27.E-02	6.75.E-01
Herelleviridae	L7_Delftia_acidovorans	-0.011	0.021	5.79.E-01	9.64.E-01
Microviridae	L7_Delftia_acidovorans	-0.004	0.021	8.56.E-01	9.96.E-01
Myoviridae	L7_Delftia_acidovorans	0.021	0.023	3.70.E-01	9.15.E-01
Phycodnaviridae	L7_Delftia_acidovorans	-0.024	0.020	2.40.E-01	8.85.E-01
Podoviridae	L7_Delftia_acidovorans	-0.046	0.024	5.16.E-02	7.22.E-01
Siphoviridae	L7_Delftia_acidovorans	-0.054	0.021	1.04.E-02	5.13.E-01
Autographiviridae	L7_Bacteroides_dorei	0.022	0.032	4.88.E-01	9.54.E-01
crAss_like_phage	L7_Bacteroides_dorei	-0.004	0.036	9.19.E-01	9.98.E-01
Herelleviridae	L7_Bacteroides_dorei	0.014	0.033	6.87.E-01	9.80.E-01
Microviridae	L7_Bacteroides_dorei	-0.005	0.034	8.84.E-01	9.96.E-01
Myoviridae	L7_Bacteroides_dorei	0.019	0.037	6.08.E-01	9.75.E-01
Phycodnaviridae	L7_Bacteroides_dorei	-0.040	0.033	2.27.E-01	8.82.E-01
Podoviridae	L7_Bacteroides_dorei	-0.075	0.039	5.27.E-02	7.22.E-01
Siphoviridae	L7_Bacteroides_dorei	-0.081	0.034	1.80.E-02	5.86.E-01
Autographiviridae	L7_Lactobacillus_casei	0.020	0.036	5.83.E-01	9.64.E-01
crAss_like_phage	L7_Lactobacillus_casei	-0.003	0.040	9.36.E-01	9.98.E-01
Herelleviridae	L7_Lactobacillus_casei	0.061	0.037	1.02.E-01	7.81.E-01
Microviridae	L7_Lactobacillus_casei	-0.021	0.037	5.65.E-01	9.61.E-01
Myoviridae	L7_Lactobacillus_casei	0.001	0.042	9.86.E-01	9.99.E-01
Phycodnaviridae	L7_Lactobacillus_casei	0.036	0.037	3.34.E-01	9.15.E-01
Podoviridae	L7_Lactobacillus_casei	-0.011	0.043	7.94.E-01	9.92.E-01
Siphoviridae	L7_Lactobacillus_casei	-0.003	0.038	9.31.E-01	9.98.E-01
Autographiviridae	L7_Culturomica_massiliensis	0.011	0.027	6.91.E-01	9.80.E-01
crAss_like_phage	L7_Culturomica_massiliensis	-0.036	0.030	2.22.E-01	8.80.E-01
Herelleviridae	L7_Culturomica_massiliensis	-0.002	0.028	9.54.E-01	9.98.E-01
Microviridae	L7_Culturomica_massiliensis	0.021	0.028	4.47.E-01	9.34.E-01
Myoviridae	L7_Culturomica_massiliensis	0.035	0.031	2.63.E-01	9.00.E-01
Phycodnaviridae	L7_Culturomica_massiliensis	0.051	0.028	6.45.E-02	7.24.E-01
Podoviridae	L7_Culturomica_massiliensis	-0.019	0.032	5.51.E-01	9.60.E-01
Siphoviridae	L7_Culturomica_massiliensis	-0.007	0.029	8.02.E-01	9.92.E-01
Autographiviridae	L7_Butyrivibrio_sp.	0.003	0.036	9.40.E-01	9.98.E-01
crAss_like_phage	L7_Butyrivibrio_sp.	0.007	0.040	8.51.E-01	9.95.E-01
Herelleviridae	L7_Butyrivibrio_sp.	0.024	0.037	5.17.E-01	9.57.E-01
Microviridae	L7_Butyrivibrio_sp.	-0.004	0.037	9.22.E-01	9.98.E-01
Myoviridae	L7_Butyrivibrio_sp.	-0.085	0.041	4.11.E-02	7.06.E-01
Phycodnaviridae	L7_Butyrivibrio_sp.	0.069	0.037	6.12.E-02	7.24.E-01
Podoviridae	L7_Butyrivibrio_sp.	0.001	0.043	9.82.E-01	9.99.E-01
Siphoviridae	L7_Butyrivibrio_sp.	-0.027	0.038	4.76.E-01	9.52.E-01
Autographiviridae	L7_Anaerobutyricum_hallii	0.003	0.037	9.36.E-01	9.98.E-01
crAss_like_phage	L7_Anaerobutyricum_hallii	-0.038	0.041	3.63.E-01	9.15.E-01
Herelleviridae	L7_Anaerobutyricum_hallii	0.061	0.039	1.14.E-01	7.85.E-01
Microviridae	L7_Anaerobutyricum_hallii	0.031	0.039	4.31.E-01	9.25.E-01
Myoviridae	L7_Anaerobutyricum_hallii	0.000	0.043	9.93.E-01	9.99.E-01
Phycodnaviridae	L7_Anaerobutyricum_hallii	0.022	0.038	5.72.E-01	9.61.E-01
Podoviridae	L7_Anaerobutyricum_hallii	-0.048	0.045	2.88.E-01	9.15.E-01
Siphoviridae	L7_Anaerobutyricum_hallii	-0.009	0.040	8.31.E-01	9.94.E-01

Autographiviridae	L7_Absiella_dolichum	-0.018	0.039	6.41.E-01	9.79.E-01
crAss_like_phage	L7_Absiella_dolichum	-0.023	0.043	5.89.E-01	9.68.E-01
Herelleviridae	L7_Absiella_dolichum	-0.065	0.040	1.05.E-01	7.82.E-01
Microviridae	L7_Absiella_dolichum	0.020	0.040	6.26.E-01	9.76.E-01
Myoviridae	L7_Absiella_dolichum	0.003	0.045	9.38.E-01	9.98.E-01
Phycodnaviridae	L7_Absiella_dolichum	0.035	0.040	3.86.E-01	9.15.E-01
Podoviridae	L7_Absiella_dolichum	-0.034	0.046	4.68.E-01	9.50.E-01
Siphoviridae	L7_Absiella_dolichum	0.001	0.041	9.83.E-01	9.99.E-01
Autographiviridae	L7_Bacteroides_pectinophilus	-0.014	0.032	6.73.E-01	9.80.E-01
crAss_like_phage	L7_Bacteroides_pectinophilus	-0.060	0.036	9.50.E-02	7.64.E-01
Herelleviridae	L7_Bacteroides_pectinophilus	-0.003	0.034	9.27.E-01	9.98.E-01
Microviridae	L7_Bacteroides_pectinophilus	-0.060	0.034	7.33.E-02	7.26.E-01
Myoviridae	L7_Bacteroides_pectinophilus	-0.034	0.038	3.67.E-01	9.15.E-01
Phycodnaviridae	L7_Bacteroides_pectinophilus	-0.021	0.033	5.29.E-01	9.57.E-01
Podoviridae	L7_Bacteroides_pectinophilus	-0.051	0.039	1.92.E-01	8.61.E-01
Siphoviridae	L7_Bacteroides_pectinophilus	-0.079	0.034	2.31.E-02	6.21.E-01
Autographiviridae	L7_Leuconostoc_lactis	-0.016	0.042	6.98.E-01	9.81.E-01
crAss_like_phage	L7_Leuconostoc_lactis	-0.010	0.046	8.29.E-01	9.94.E-01
Herelleviridae	L7_Leuconostoc_lactis	0.065	0.043	1.34.E-01	8.06.E-01
Microviridae	L7_Leuconostoc_lactis	0.062	0.043	1.51.E-01	8.22.E-01
Myoviridae	L7_Leuconostoc_lactis	0.014	0.048	7.69.E-01	9.91.E-01
Phycodnaviridae	L7_Leuconostoc_lactis	0.006	0.043	8.84.E-01	9.96.E-01
Podoviridae	L7_Leuconostoc_lactis	-0.003	0.050	9.53.E-01	9.98.E-01
Siphoviridae	L7_Leuconostoc_lactis	0.082	0.044	6.64.E-02	7.24.E-01
Autographiviridae	L7_Actinomyces_naeslundii	-0.009	0.037	8.03.E-01	9.92.E-01
crAss_like_phage	L7_Actinomyces_naeslundii	-0.013	0.041	7.51.E-01	9.89.E-01
Herelleviridae	L7_Actinomyces_naeslundii	-0.016	0.038	6.74.E-01	9.80.E-01
Microviridae	L7_Actinomyces_naeslundii	-0.048	0.038	2.09.E-01	8.76.E-01
Myoviridae	L7_Actinomyces_naeslundii	0.003	0.043	9.38.E-01	9.98.E-01
Phycodnaviridae	L7_Actinomyces_naeslundii	-0.002	0.038	9.58.E-01	9.98.E-01
Podoviridae	L7_Actinomyces_naeslundii	-0.040	0.044	3.65.E-01	9.15.E-01
Siphoviridae	L7_Actinomyces_naeslundii	-0.025	0.039	5.26.E-01	9.57.E-01
Autographiviridae	L7_Desulfovibrio_desulfuricans	-0.019	0.040	6.30.E-01	9.76.E-01
crAss_like_phage	L7_Desulfovibrio_desulfuricans	0.019	0.044	6.57.E-01	9.79.E-01
Herelleviridae	L7_Desulfovibrio_desulfuricans	0.052	0.041	2.03.E-01	8.75.E-01
Microviridae	L7_Desulfovibrio_desulfuricans	-0.072	0.041	8.14.E-02	7.47.E-01
Myoviridae	L7_Desulfovibrio_desulfuricans	-0.010	0.046	8.27.E-01	9.93.E-01
Phycodnaviridae	L7_Desulfovibrio_desulfuricans	0.024	0.041	5.50.E-01	9.60.E-01
Podoviridae	L7_Desulfovibrio_desulfuricans	-0.008	0.047	8.71.E-01	9.96.E-01
Siphoviridae	L7_Desulfovibrio_desulfuricans	0.001	0.042	9.81.E-01	9.99.E-01
Autographiviridae	L7_Clostridium_asparagiforme	0.001	0.037	9.77.E-01	9.99.E-01
crAss_like_phage	L7_Clostridium_asparagiforme	-0.011	0.041	7.77.E-01	9.92.E-01
Herelleviridae	L7_Clostridium_asparagiforme	-0.032	0.038	3.99.E-01	9.15.E-01
Microviridae	L7_Clostridium_asparagiforme	0.009	0.038	8.17.E-01	9.93.E-01
Myoviridae	L7_Clostridium_asparagiforme	0.028	0.043	5.09.E-01	9.57.E-01
Phycodnaviridae	L7_Clostridium_asparagiforme	0.030	0.038	4.20.E-01	9.20.E-01
Podoviridae	L7_Clostridium_asparagiforme	0.070	0.044	1.13.E-01	7.84.E-01
Siphoviridae	L7_Clostridium_asparagiforme	-0.019	0.039	6.26.E-01	9.76.E-01
Autographiviridae	L7_Prevotella_bivia	0.030	0.029	3.00.E-01	9.15.E-01
crAss_like_phage	L7_Prevotella_bivia	0.008	0.032	8.04.E-01	9.93.E-01
Herelleviridae	L7_Prevotella_bivia	0.003	0.030	9.34.E-01	9.98.E-01
Microviridae	L7_Prevotella_bivia	0.007	0.030	8.19.E-01	9.93.E-01
Myoviridae	L7_Prevotella_bivia	-0.013	0.034	6.98.E-01	9.81.E-01
Phycodnaviridae	L7_Prevotella_bivia	0.005	0.030	8.78.E-01	9.96.E-01
Podoviridae	L7_Prevotella_bivia	0.003	0.035	9.29.E-01	9.98.E-01
Siphoviridae	L7_Prevotella_bivia	0.008	0.031	8.00.E-01	9.92.E-01
Autographiviridae	L7_Fournierella_massiliensis	-0.057	0.036	1.10.E-01	7.82.E-01
crAss_like_phage	L7_Fournierella_massiliensis	-0.034	0.040	3.89.E-01	9.15.E-01
Herelleviridae	L7_Fournierella_massiliensis	-0.013	0.037	7.36.E-01	9.88.E-01
Microviridae	L7_Fournierella_massiliensis	-0.009	0.037	8.13.E-01	9.93.E-01
Myoviridae	L7_Fournierella_massiliensis	0.006	0.042	8.82.E-01	9.96.E-01
Phycodnaviridae	L7_Fournierella_massiliensis	-0.026	0.037	4.76.E-01	9.52.E-01
Podoviridae	L7_Fournierella_massiliensis	0.016	0.043	7.14.E-01	9.86.E-01
Siphoviridae	L7_Fournierella_massiliensis	0.026	0.038	5.07.E-01	9.57.E-01
Autographiviridae	L7_Streptococcus_mutans	0.031	0.034	3.51.E-01	9.15.E-01

crAss_like_phage	L7_Streptococcus_mutans	0.015	0.037	6.85.E-01	9.80.E-01
Herelleviridae	L7_Streptococcus_mutans	-0.025	0.035	4.75.E-01	9.52.E-01
Microviridae	L7_Streptococcus_mutans	0.043	0.035	2.26.E-01	8.82.E-01
Myoviridae	L7_Streptococcus_mutans	0.003	0.039	9.39.E-01	9.98.E-01
Phycodnaviridae	L7_Streptococcus_mutans	0.054	0.035	1.22.E-01	7.93.E-01
Podoviridae	L7_Streptococcus_mutans	-0.041	0.040	3.06.E-01	9.15.E-01
Siphoviridae	L7_Streptococcus_mutans	0.022	0.036	5.50.E-01	9.60.E-01
Autographiviridae	L7_Clostridium_sp.	-0.004	0.031	8.94.E-01	9.97.E-01
crAss_like_phage	L7_Clostridium_sp.	0.021	0.034	5.42.E-01	9.60.E-01
Herelleviridae	L7_Clostridium_sp.	0.055	0.032	8.64.E-02	7.52.E-01
Microviridae	L7_Clostridium_sp.	0.007	0.032	8.18.E-01	9.93.E-01
Myoviridae	L7_Clostridium_sp.	0.018	0.036	6.16.E-01	9.75.E-01
Phycodnaviridae	L7_Clostridium_sp.	0.052	0.032	1.03.E-01	7.82.E-01
Podoviridae	L7_Clostridium_sp.	-0.029	0.037	4.34.E-01	9.26.E-01
Siphoviridae	L7_Clostridium_sp.	-0.009	0.033	7.93.E-01	9.92.E-01
Autographiviridae	L7_Ruminococcus_gauvreauii	-0.009	0.034	7.98.E-01	9.92.E-01
crAss_like_phage	L7_Ruminococcus_gauvreauii	0.005	0.038	8.99.E-01	9.97.E-01
Herelleviridae	L7_Ruminococcus_gauvreauii	-0.023	0.036	5.13.E-01	9.57.E-01
Microviridae	L7_Ruminococcus_gauvreauii	0.018	0.036	6.22.E-01	9.76.E-01
Myoviridae	L7_Ruminococcus_gauvreauii	-0.015	0.040	7.04.E-01	9.82.E-01
Phycodnaviridae	L7_Ruminococcus_gauvreauii	-0.027	0.035	4.46.E-01	9.34.E-01
Podoviridae	L7_Ruminococcus_gauvreauii	0.005	0.041	9.01.E-01	9.97.E-01
Siphoviridae	L7_Ruminococcus_gauvreauii	-0.031	0.037	4.03.E-01	9.16.E-01
Autographiviridae	L7_Klebsiella_michiganensis	-0.030	0.027	2.65.E-01	9.01.E-01
crAss_like_phage	L7_Klebsiella_michiganensis	-0.048	0.030	1.11.E-01	7.82.E-01
Herelleviridae	L7_Klebsiella_michiganensis	-0.031	0.028	2.66.E-01	9.01.E-01
Microviridae	L7_Klebsiella_michiganensis	-0.003	0.028	9.16.E-01	9.98.E-01
Myoviridae	L7_Klebsiella_michiganensis	-0.068	0.031	3.00.E-02	6.66.E-01
Phycodnaviridae	L7_Klebsiella_michiganensis	-0.015	0.028	5.94.E-01	9.69.E-01
Podoviridae	L7_Klebsiella_michiganensis	-0.024	0.033	4.70.E-01	9.50.E-01
Siphoviridae	L7_Klebsiella_michiganensis	-0.061	0.029	3.68.E-02	6.90.E-01
Autographiviridae	L7_Parasutterella_excrementihominis	0.000	0.038	9.90.E-01	9.99.E-01
crAss_like_phage	L7_Parasutterella_excrementihominis	0.013	0.042	7.53.E-01	9.89.E-01
Herelleviridae	L7_Parasutterella_excrementihominis	0.058	0.040	1.43.E-01	8.12.E-01
Microviridae	L7_Parasutterella_excrementihominis	-0.074	0.040	6.16.E-02	7.24.E-01
Myoviridae	L7_Parasutterella_excrementihominis	0.034	0.044	4.50.E-01	9.37.E-01
Phycodnaviridae	L7_Parasutterella_excrementihominis	0.018	0.039	6.46.E-01	9.79.E-01
Podoviridae	L7_Parasutterella_excrementihominis	-0.027	0.046	5.53.E-01	9.60.E-01
Siphoviridae	L7_Parasutterella_excrementihominis	-0.024	0.041	5.65.E-01	9.61.E-01
Autographiviridae	L7_Lactobacillus_rhamnosus	-0.012	0.034	7.18.E-01	9.86.E-01
crAss_like_phage	L7_Lactobacillus_rhamnosus	0.029	0.038	4.39.E-01	9.30.E-01
Herelleviridae	L7_Lactobacillus_rhamnosus	0.059	0.035	9.50.E-02	7.64.E-01
Microviridae	L7_Lactobacillus_rhamnosus	-0.026	0.036	4.65.E-01	9.45.E-01
Myoviridae	L7_Lactobacillus_rhamnosus	-0.020	0.040	6.15.E-01	9.75.E-01
Phycodnaviridae	L7_Lactobacillus_rhamnosus	0.031	0.035	3.70.E-01	9.15.E-01
Podoviridae	L7_Lactobacillus_rhamnosus	0.013	0.041	7.58.E-01	9.90.E-01
Siphoviridae	L7_Lactobacillus_rhamnosus	0.002	0.036	9.55.E-01	9.98.E-01
Autographiviridae	L7_Bifidobacterium_stercoris	0.036	0.031	2.38.E-01	8.85.E-01
crAss_like_phage	L7_Bifidobacterium_stercoris	-0.032	0.034	3.50.E-01	9.15.E-01
Herelleviridae	L7_Bifidobacterium_stercoris	0.018	0.032	5.81.E-01	9.64.E-01
Microviridae	L7_Bifidobacterium_stercoris	-0.011	0.032	7.40.E-01	9.88.E-01
Myoviridae	L7_Bifidobacterium_stercoris	-0.043	0.036	2.32.E-01	8.82.E-01
Phycodnaviridae	L7_Bifidobacterium_stercoris	0.002	0.032	9.39.E-01	9.98.E-01
Podoviridae	L7_Bifidobacterium_stercoris	-0.020	0.037	5.90.E-01	9.68.E-01
Siphoviridae	L7_Bifidobacterium_stercoris	0.029	0.033	3.74.E-01	9.15.E-01
Autographiviridae	L7_Bacteroides_rodentium	-0.005	0.031	8.69.E-01	9.96.E-01
crAss_like_phage	L7_Bacteroides_rodentium	-0.057	0.034	9.37.E-02	7.62.E-01
Herelleviridae	L7_Bacteroides_rodentium	-0.016	0.032	6.26.E-01	9.76.E-01
Microviridae	L7_Bacteroides_rodentium	0.005	0.032	8.88.E-01	9.96.E-01
Myoviridae	L7_Bacteroides_rodentium	-0.012	0.036	7.38.E-01	9.88.E-01
Phycodnaviridae	L7_Bacteroides_rodentium	-0.030	0.032	3.52.E-01	9.15.E-01
Podoviridae	L7_Bacteroides_rodentium	-0.045	0.037	2.30.E-01	8.82.E-01
Siphoviridae	L7_Bacteroides_rodentium	0.009	0.033	7.97.E-01	9.92.E-01
Autographiviridae	L7_Corynebacterium_sp.	-0.005	0.038	8.99.E-01	9.97.E-01
crAss_like_phage	L7_Corynebacterium_sp.	0.037	0.042	3.83.E-01	9.15.E-01

Herelleviridae	L7_Corynebacterium_sp.	0.003	0.040	9.31.E-01	9.98.E-01
Microviridae	L7_Corynebacterium_sp.	0.102	0.040	1.06.E-02	5.15.E-01
Myoviridae	L7_Corynebacterium_sp.	0.030	0.044	5.03.E-01	9.55.E-01
Phycodnaviridae	L7_Corynebacterium_sp.	0.016	0.039	6.93.E-01	9.81.E-01
Podoviridae	L7_Corynebacterium_sp.	0.050	0.046	2.74.E-01	9.04.E-01
Siphoviridae	L7_Corynebacterium_sp.	-0.035	0.041	3.95.E-01	9.15.E-01
Autographiviridae	L7_Lactobacillus_helveticus	-0.004	0.033	9.01.E-01	9.97.E-01
crAss_like_phage	L7_Lactobacillus_helveticus	0.014	0.036	6.88.E-01	9.80.E-01
Herelleviridae	L7_Lactobacillus_helveticus	-0.036	0.034	2.84.E-01	9.14.E-01
Microviridae	L7_Lactobacillus_helveticus	0.020	0.034	5.55.E-01	9.61.E-01
Myoviridae	L7_Lactobacillus_helveticus	0.036	0.038	3.48.E-01	9.15.E-01
Phycodnaviridae	L7_Lactobacillus_helveticus	-0.019	0.033	5.73.E-01	9.61.E-01
Podoviridae	L7_Lactobacillus_helveticus	0.000	0.039	9.93.E-01	9.99.E-01
Siphoviridae	L7_Lactobacillus_helveticus	-0.010	0.035	7.80.E-01	9.92.E-01
Autographiviridae	L7_Blautia_producta	-0.084	0.035	1.75.E-02	5.86.E-01
crAss_like_phage	L7_Blautia_producta	0.002	0.039	9.68.E-01	9.98.E-01
Herelleviridae	L7_Blautia_producta	-0.018	0.037	6.26.E-01	9.76.E-01
Microviridae	L7_Blautia_producta	-0.007	0.037	8.53.E-01	9.95.E-01
Myoviridae	L7_Blautia_producta	-0.024	0.041	5.62.E-01	9.61.E-01
Phycodnaviridae	L7_Blautia_producta	0.027	0.036	4.53.E-01	9.38.E-01
Podoviridae	L7_Blautia_producta	0.028	0.042	5.08.E-01	9.57.E-01
Siphoviridae	L7_Blautia_producta	-0.037	0.038	3.25.E-01	9.15.E-01
Autographiviridae	L7_Bifidobacterium_reuteri	0.019	0.031	5.52.E-01	9.60.E-01
crAss_like_phage	L7_Bifidobacterium_reuteri	0.056	0.034	1.02.E-01	7.81.E-01
Herelleviridae	L7_Bifidobacterium_reuteri	-0.049	0.032	1.28.E-01	7.99.E-01
Microviridae	L7_Bifidobacterium_reuteri	0.098	0.032	2.44.E-03	3.52.E-01
Myoviridae	L7_Bifidobacterium_reuteri	0.006	0.036	8.62.E-01	9.96.E-01
Phycodnaviridae	L7_Bifidobacterium_reuteri	0.006	0.032	8.62.E-01	9.96.E-01
Podoviridae	L7_Bifidobacterium_reuteri	0.089	0.037	1.75.E-02	5.86.E-01
Siphoviridae	L7_Bifidobacterium_reuteri	-0.016	0.033	6.35.E-01	9.76.E-01
Autographiviridae	L7_Streptococcus_infantarius	-0.022	0.037	5.59.E-01	9.61.E-01
crAss_like_phage	L7_Streptococcus_infantarius	0.024	0.041	5.56.E-01	9.61.E-01
Herelleviridae	L7_Streptococcus_infantarius	0.040	0.038	2.96.E-01	9.15.E-01
Microviridae	L7_Streptococcus_infantarius	0.051	0.038	1.83.E-01	8.60.E-01
Myoviridae	L7_Streptococcus_infantarius	0.002	0.043	9.59.E-01	9.98.E-01
Phycodnaviridae	L7_Streptococcus_infantarius	0.010	0.038	8.00.E-01	9.92.E-01
Podoviridae	L7_Streptococcus_infantarius	0.031	0.044	4.84.E-01	9.54.E-01
Siphoviridae	L7_Streptococcus_infantarius	0.055	0.039	1.61.E-01	8.38.E-01
Autographiviridae	L7_Caecibacter_massiliensis	0.006	0.044	8.85.E-01	9.96.E-01
crAss_like_phage	L7_Caecibacter_massiliensis	-0.031	0.049	5.20.E-01	9.57.E-01
Herelleviridae	L7_Caecibacter_massiliensis	-0.089	0.046	5.10.E-02	7.22.E-01
Microviridae	L7_Caecibacter_massiliensis	0.004	0.046	9.38.E-01	9.98.E-01
Myoviridae	L7_Caecibacter_massiliensis	0.018	0.051	7.25.E-01	9.87.E-01
Phycodnaviridae	L7_Caecibacter_massiliensis	-0.044	0.045	3.33.E-01	9.15.E-01
Podoviridae	L7_Caecibacter_massiliensis	-0.060	0.053	2.54.E-01	8.95.E-01
Siphoviridae	L7_Caecibacter_massiliensis	0.004	0.047	9.35.E-01	9.98.E-01
Autographiviridae	L7_Bacteroides_gallinarum	-0.037	0.030	2.19.E-01	8.79.E-01
crAss_like_phage	L7_Bacteroides_gallinarum	-0.002	0.033	9.62.E-01	9.98.E-01
Herelleviridae	L7_Bacteroides_gallinarum	0.011	0.031	7.20.E-01	9.86.E-01
Microviridae	L7_Bacteroides_gallinarum	0.023	0.032	4.70.E-01	9.50.E-01
Myoviridae	L7_Bacteroides_gallinarum	0.010	0.035	7.86.E-01	9.92.E-01
Phycodnaviridae	L7_Bacteroides_gallinarum	0.027	0.031	3.88.E-01	9.15.E-01
Podoviridae	L7_Bacteroides_gallinarum	-0.047	0.036	1.99.E-01	8.70.E-01
Siphoviridae	L7_Bacteroides_gallinarum	-0.002	0.032	9.55.E-01	9.98.E-01
Autographiviridae	L7_Eubacterium_sulci	0.010	0.035	7.81.E-01	9.92.E-01
crAss_like_phage	L7_Eubacterium_sulci	0.008	0.039	8.45.E-01	9.95.E-01
Herelleviridae	L7_Eubacterium_sulci	-0.046	0.037	2.08.E-01	8.76.E-01
Microviridae	L7_Eubacterium_sulci	0.033	0.037	3.65.E-01	9.15.E-01
Myoviridae	L7_Eubacterium_sulci	-0.027	0.041	5.15.E-01	9.57.E-01
Phycodnaviridae	L7_Eubacterium_sulci	-0.013	0.036	7.20.E-01	9.86.E-01
Podoviridae	L7_Eubacterium_sulci	0.010	0.042	8.15.E-01	9.93.E-01
Siphoviridae	L7_Eubacterium_sulci	-0.045	0.038	2.38.E-01	8.85.E-01
Autographiviridae	L7_Bacteroides_intestinalis	-0.003	0.028	9.15.E-01	9.98.E-01
crAss_like_phage	L7_Bacteroides_intestinalis	0.083	0.031	7.78.E-03	4.66.E-01
Herelleviridae	L7_Bacteroides_intestinalis	0.018	0.029	5.35.E-01	9.59.E-01

Microviridae	L7_Bacteroides_intestinalis	0.028	0.029	3.35.E-01	9.15.E-01
Myoviridae	L7_Bacteroides_intestinalis	0.016	0.033	6.25.E-01	9.76.E-01
Phycodnaviridae	L7_Bacteroides_intestinalis	0.015	0.029	5.97.E-01	9.69.E-01
Podoviridae	L7_Bacteroides_intestinalis	-0.038	0.034	2.66.E-01	9.01.E-01
Siphoviridae	L7_Bacteroides_intestinalis	0.019	0.030	5.37.E-01	9.60.E-01
Autographiviridae	L7_Clostridium_symbiosum	0.001	0.035	9.80.E-01	9.99.E-01
crAss_like_phage	L7_Clostridium_symbiosum	-0.016	0.038	6.74.E-01	9.80.E-01
Herelleviridae	L7_Clostridium_symbiosum	-0.074	0.036	3.92.E-02	7.03.E-01
Microviridae	L7_Clostridium_symbiosum	0.064	0.036	7.73.E-02	7.41.E-01
Myoviridae	L7_Clostridium_symbiosum	-0.054	0.040	1.83.E-01	8.60.E-01
Phycodnaviridae	L7_Clostridium_symbiosum	-0.024	0.036	5.07.E-01	9.57.E-01
Podoviridae	L7_Clostridium_symbiosum	0.001	0.042	9.74.E-01	9.99.E-01
Siphoviridae	L7_Clostridium_symbiosum	0.055	0.037	1.38.E-01	8.10.E-01
Autographiviridae	L7_Streptococcus_anginosus	-0.011	0.028	6.90.E-01	9.80.E-01
crAss_like_phage	L7_Streptococcus_anginosus	0.007	0.031	8.29.E-01	9.94.E-01
Herelleviridae	L7_Streptococcus_anginosus	0.079	0.029	6.86.E-03	4.51.E-01
Microviridae	L7_Streptococcus_anginosus	0.024	0.029	4.17.E-01	9.19.E-01
Myoviridae	L7_Streptococcus_anginosus	0.007	0.033	8.36.E-01	9.94.E-01
Phycodnaviridae	L7_Streptococcus_anginosus	0.049	0.029	9.14.E-02	7.59.E-01
Podoviridae	L7_Streptococcus_anginosus	0.047	0.034	1.65.E-01	8.42.E-01
Siphoviridae	L7_Streptococcus_anginosus	0.004	0.030	8.87.E-01	9.96.E-01
Autographiviridae	L7_Hungatella_hathewayi	-0.006	0.029	8.50.E-01	9.95.E-01
crAss_like_phage	L7_Hungatella_hathewayi	0.014	0.032	6.55.E-01	9.79.E-01
Herelleviridae	L7_Hungatella_hathewayi	0.056	0.030	6.74.E-02	7.24.E-01
Microviridae	L7_Hungatella_hathewayi	-0.013	0.031	6.79.E-01	9.80.E-01
Myoviridae	L7_Hungatella_hathewayi	0.033	0.034	3.30.E-01	9.15.E-01
Phycodnaviridae	L7_Hungatella_hathewayi	0.056	0.030	6.04.E-02	7.24.E-01
Podoviridae	L7_Hungatella_hathewayi	-0.012	0.035	7.36.E-01	9.88.E-01
Siphoviridae	L7_Hungatella_hathewayi	-0.002	0.031	9.45.E-01	9.98.E-01
Autographiviridae	L7_Bacteroides_uniformis	0.015	0.026	5.69.E-01	9.61.E-01
crAss_like_phage	L7_Bacteroides_uniformis	-0.014	0.029	6.25.E-01	9.76.E-01
Herelleviridae	L7_Bacteroides_uniformis	-0.034	0.027	2.05.E-01	8.76.E-01
Microviridae	L7_Bacteroides_uniformis	-0.005	0.027	8.53.E-01	9.95.E-01
Myoviridae	L7_Bacteroides_uniformis	0.036	0.030	2.31.E-01	8.82.E-01
Phycodnaviridae	L7_Bacteroides_uniformis	-0.023	0.027	3.90.E-01	9.15.E-01
Podoviridae	L7_Bacteroides_uniformis	0.017	0.031	5.83.E-01	9.64.E-01
Siphoviridae	L7_Bacteroides_uniformis	-0.010	0.028	7.19.E-01	9.86.E-01
Autographiviridae	L7_Coprococcus_comes	0.043	0.036	2.25.E-01	8.82.E-01
crAss_like_phage	L7_Coprococcus_comes	-0.035	0.039	3.80.E-01	9.15.E-01
Herelleviridae	L7_Coprococcus_comes	0.012	0.037	7.50.E-01	9.89.E-01
Microviridae	L7_Coprococcus_comes	-0.041	0.037	2.65.E-01	9.01.E-01
Myoviridae	L7_Coprococcus_comes	-0.061	0.041	1.40.E-01	8.10.E-01
Phycodnaviridae	L7_Coprococcus_comes	0.032	0.037	3.82.E-01	9.15.E-01
Podoviridae	L7_Coprococcus_comes	0.062	0.043	1.45.E-01	8.15.E-01
Siphoviridae	L7_Coprococcus_comes	-0.021	0.038	5.75.E-01	9.62.E-01
Autographiviridae	L7_Synergistes_sp.	0.087	0.037	1.94.E-02	5.90.E-01
crAss_like_phage	L7_Synergistes_sp.	-0.038	0.041	3.55.E-01	9.15.E-01
Herelleviridae	L7_Synergistes_sp.	0.022	0.039	5.70.E-01	9.61.E-01
Microviridae	L7_Synergistes_sp.	0.017	0.039	6.64.E-01	9.79.E-01
Myoviridae	L7_Synergistes_sp.	-0.039	0.043	3.76.E-01	9.15.E-01
Phycodnaviridae	L7_Synergistes_sp.	-0.037	0.038	3.40.E-01	9.15.E-01
Podoviridae	L7_Synergistes_sp.	-0.034	0.045	4.50.E-01	9.37.E-01
Siphoviridae	L7_Synergistes_sp.	-0.008	0.040	8.47.E-01	9.95.E-01
Autographiviridae	L7_Pseudoflavonifractor_capillosus	-0.005	0.026	8.41.E-01	9.95.E-01
crAss_like_phage	L7_Pseudoflavonifractor_capillosus	-0.075	0.029	9.49.E-03	4.94.E-01
Herelleviridae	L7_Pseudoflavonifractor_capillosus	-0.025	0.027	3.68.E-01	9.15.E-01
Microviridae	L7_Pseudoflavonifractor_capillosus	-0.003	0.027	9.18.E-01	9.98.E-01
Myoviridae	L7_Pseudoflavonifractor_capillosus	-0.003	0.030	9.09.E-01	9.98.E-01
Phycodnaviridae	L7_Pseudoflavonifractor_capillosus	-0.033	0.027	2.25.E-01	8.82.E-01
Podoviridae	L7_Pseudoflavonifractor_capillosus	-0.028	0.031	3.78.E-01	9.15.E-01
Siphoviridae	L7_Pseudoflavonifractor_capillosus	0.009	0.028	7.44.E-01	9.89.E-01
Autographiviridae	L7_Bacteroides_ovatus	0.029	0.031	3.53.E-01	9.15.E-01
crAss_like_phage	L7_Bacteroides_ovatus	0.003	0.034	9.22.E-01	9.98.E-01
Herelleviridae	L7_Bacteroides_ovatus	-0.021	0.032	5.16.E-01	9.57.E-01
Microviridae	L7_Bacteroides_ovatus	-0.052	0.032	1.07.E-01	7.82.E-01

Myoviridae	L7_Bacteroides_ovatus	-0.083	0.035	1.92.E-02	5.90.E-01
Phycodnaviridae	L7_Bacteroides_ovatus	0.048	0.031	1.28.E-01	7.99.E-01
Podoviridae	L7_Bacteroides_ovatus	-0.023	0.037	5.26.E-01	9.57.E-01
Siphoviridae	L7_Bacteroides_ovatus	0.004	0.033	9.13.E-01	9.98.E-01
Autographiviridae	L7_Alistipes_onderdonkii	-0.001	0.032	9.79.E-01	9.99.E-01
crAss_like_phage	L7_Alistipes_onderdonkii	0.037	0.036	3.06.E-01	9.15.E-01
Herelleviridae	L7_Alistipes_onderdonkii	0.033	0.034	3.25.E-01	9.15.E-01
Microviridae	L7_Alistipes_onderdonkii	0.016	0.034	6.34.E-01	9.76.E-01
Myoviridae	L7_Alistipes_onderdonkii	-0.038	0.038	3.08.E-01	9.15.E-01
Phycodnaviridae	L7_Alistipes_onderdonkii	0.014	0.033	6.75.E-01	9.80.E-01
Podoviridae	L7_Alistipes_onderdonkii	0.041	0.039	2.91.E-01	9.15.E-01
Siphoviridae	L7_Alistipes_onderdonkii	0.006	0.035	8.55.E-01	9.96.E-01
Autographiviridae	L7_Eubacterium_limosum	0.020	0.037	6.00.E-01	9.71.E-01
crAss_like_phage	L7_Eubacterium_limosum	0.018	0.041	6.69.E-01	9.80.E-01
Herelleviridae	L7_Eubacterium_limosum	-0.033	0.039	3.97.E-01	9.15.E-01
Microviridae	L7_Eubacterium_limosum	-0.001	0.039	9.80.E-01	9.99.E-01
Myoviridae	L7_Eubacterium_limosum	-0.036	0.043	4.07.E-01	9.17.E-01
Phycodnaviridae	L7_Eubacterium_limosum	-0.040	0.038	2.96.E-01	9.15.E-01
Podoviridae	L7_Eubacterium_limosum	0.048	0.045	2.83.E-01	9.13.E-01
Siphoviridae	L7_Eubacterium_limosum	-0.109	0.040	6.34.E-03	4.51.E-01
Autographiviridae	L7_Klebsiella_oxytoca	0.002	0.021	9.20.E-01	9.98.E-01
crAss_like_phage	L7_Klebsiella_oxytoca	0.017	0.023	4.78.E-01	9.52.E-01
Herelleviridae	L7_Klebsiella_oxytoca	-0.013	0.022	5.46.E-01	9.60.E-01
Microviridae	L7_Klebsiella_oxytoca	0.008	0.022	7.07.E-01	9.83.E-01
Myoviridae	L7_Klebsiella_oxytoca	-0.024	0.025	3.38.E-01	9.15.E-01
Phycodnaviridae	L7_Klebsiella_oxytoca	-0.024	0.022	2.66.E-01	9.01.E-01
Podoviridae	L7_Klebsiella_oxytoca	0.030	0.025	2.37.E-01	8.85.E-01
Siphoviridae	L7_Klebsiella_oxytoca	0.034	0.023	1.35.E-01	8.06.E-01
Autographiviridae	L7_Acidaminococcus_massiliensis	-0.051	0.039	1.87.E-01	8.61.E-01
crAss_like_phage	L7_Acidaminococcus_massiliensis	-0.007	0.043	8.71.E-01	9.96.E-01
Herelleviridae	L7_Acidaminococcus_massiliensis	-0.029	0.040	4.75.E-01	9.52.E-01
Microviridae	L7_Acidaminococcus_massiliensis	-0.010	0.040	8.12.E-01	9.93.E-01
Myoviridae	L7_Acidaminococcus_massiliensis	0.048	0.045	2.89.E-01	9.15.E-01
Phycodnaviridae	L7_Acidaminococcus_massiliensis	0.009	0.040	8.28.E-01	9.94.E-01
Podoviridae	L7_Acidaminococcus_massiliensis	-0.027	0.046	5.66.E-01	9.61.E-01
Siphoviridae	L7_Acidaminococcus_massiliensis	0.027	0.041	5.18.E-01	9.57.E-01
Autographiviridae	L7_Agathobaculum_butyriciproducens	-0.045	0.035	2.07.E-01	8.76.E-01
crAss_like_phage	L7_Agathobaculum_butyriciproducens	0.049	0.039	2.13.E-01	8.78.E-01
Herelleviridae	L7_Agathobaculum_butyriciproducens	0.046	0.037	2.15.E-01	8.79.E-01
Microviridae	L7_Agathobaculum_butyriciproducens	-0.023	0.037	5.34.E-01	9.59.E-01
Myoviridae	L7_Agathobaculum_butyriciproducens	0.053	0.041	2.01.E-01	8.74.E-01
Phycodnaviridae	L7_Agathobaculum_butyriciproducens	0.005	0.037	9.00.E-01	9.97.E-01
Podoviridae	L7_Agathobaculum_butyriciproducens	0.107	0.042	1.17.E-02	5.36.E-01
Siphoviridae	L7_Agathobaculum_butyriciproducens	0.083	0.038	2.93.E-02	6.66.E-01
Autographiviridae	L7_Curtobacterium_sp.	0.006	0.011	5.56.E-01	9.61.E-01
crAss_like_phage	L7_Curtobacterium_sp.	0.004	0.012	7.52.E-01	9.89.E-01
Herelleviridae	L7_Curtobacterium_sp.	0.006	0.011	5.97.E-01	9.69.E-01
Microviridae	L7_Curtobacterium_sp.	-0.003	0.011	7.98.E-01	9.92.E-01
Myoviridae	L7_Curtobacterium_sp.	-0.010	0.012	4.32.E-01	9.25.E-01
Phycodnaviridae	L7_Curtobacterium_sp.	-0.003	0.011	7.96.E-01	9.92.E-01
Podoviridae	L7_Curtobacterium_sp.	-0.008	0.013	5.21.E-01	9.57.E-01
Siphoviridae	L7_Curtobacterium_sp.	-0.011	0.011	3.50.E-01	9.15.E-01
Autographiviridae	L7_Clostridium_nexile	-0.013	0.038	7.41.E-01	9.89.E-01
crAss_like_phage	L7_Clostridium_nexile	-0.036	0.042	4.00.E-01	9.15.E-01
Herelleviridae	L7_Clostridium_nexile	0.051	0.040	1.98.E-01	8.68.E-01
Microviridae	L7_Clostridium_nexile	0.001	0.040	9.85.E-01	9.99.E-01
Myoviridae	L7_Clostridium_nexile	-0.064	0.044	1.47.E-01	8.17.E-01
Phycodnaviridae	L7_Clostridium_nexile	-0.028	0.039	4.69.E-01	9.50.E-01
Podoviridae	L7_Clostridium_nexile	-0.004	0.046	9.24.E-01	9.98.E-01
Siphoviridae	L7_Clostridium_nexile	-0.033	0.041	4.24.E-01	9.23.E-01
Autographiviridae	L7_Bifidobacterium_catenulatum	0.004	0.030	9.02.E-01	9.97.E-01
crAss_like_phage	L7_Bifidobacterium_catenulatum	-0.049	0.033	1.40.E-01	8.10.E-01
Herelleviridae	L7_Bifidobacterium_catenulatum	0.010	0.031	7.38.E-01	9.88.E-01
Microviridae	L7_Bifidobacterium_catenulatum	0.063	0.031	4.44.E-02	7.22.E-01
Myoviridae	L7_Bifidobacterium_catenulatum	0.021	0.035	5.48.E-01	9.60.E-01

Phycodnaviridae	L7_Bifidobacterium_catenulatum	0.013	0.031	6.85.E-01	9.80.E-01
Podoviridae	L7_Bifidobacterium_catenulatum	0.042	0.036	2.46.E-01	8.92.E-01
Siphoviridae	L7_Bifidobacterium_catenulatum	0.007	0.032	8.24.E-01	9.93.E-01
Autographiviridae	L7_Clostridium_celerecrescens	0.011	0.039	7.88.E-01	9.92.E-01
crAss_like_phage	L7_Clostridium_celerecrescens	0.057	0.043	1.92.E-01	8.61.E-01
Herelleviridae	L7_Clostridium_celerecrescens	-0.057	0.041	1.61.E-01	8.38.E-01
Microviridae	L7_Clostridium_celerecrescens	0.063	0.041	1.24.E-01	7.94.E-01
Myoviridae	L7_Clostridium_celerecrescens	0.072	0.046	1.17.E-01	7.86.E-01
Phycodnaviridae	L7_Clostridium_celerecrescens	0.075	0.040	6.25.E-02	7.24.E-01
Podoviridae	L7_Clostridium_celerecrescens	0.020	0.047	6.64.E-01	9.79.E-01
Siphoviridae	L7_Clostridium_celerecrescens	0.086	0.042	3.96.E-02	7.03.E-01
Autographiviridae	L7_Streptococcus_cristatus	-0.011	0.030	7.01.E-01	9.81.E-01
crAss_like_phage	L7_Streptococcus_cristatus	0.005	0.033	8.75.E-01	9.96.E-01
Herelleviridae	L7_Streptococcus_cristatus	-0.010	0.031	7.54.E-01	9.89.E-01
Microviridae	L7_Streptococcus_cristatus	-0.042	0.031	1.77.E-01	8.58.E-01
Myoviridae	L7_Streptococcus_cristatus	0.000	0.035	9.93.E-01	9.99.E-01
Phycodnaviridae	L7_Streptococcus_cristatus	-0.049	0.030	1.12.E-01	7.83.E-01
Podoviridae	L7_Streptococcus_cristatus	0.003	0.036	9.39.E-01	9.98.E-01
Siphoviridae	L7_Streptococcus_cristatus	0.021	0.032	5.05.E-01	9.57.E-01
Autographiviridae	L7_Aeromicrobium_massiliense	0.005	0.032	8.67.E-01	9.96.E-01
crAss_like_phage	L7_Aeromicrobium_massiliense	0.018	0.035	6.13.E-01	9.75.E-01
Herelleviridae	L7_Aeromicrobium_massiliense	0.058	0.033	7.95.E-02	7.43.E-01
Microviridae	L7_Aeromicrobium_massiliense	0.013	0.033	6.92.E-01	9.80.E-01
Myoviridae	L7_Aeromicrobium_massiliense	-0.089	0.037	1.48.E-02	5.56.E-01
Phycodnaviridae	L7_Aeromicrobium_massiliense	-0.009	0.033	7.89.E-01	9.92.E-01
Podoviridae	L7_Aeromicrobium_massiliense	0.078	0.038	4.10.E-02	7.06.E-01
Siphoviridae	L7_Aeromicrobium_massiliense	0.022	0.034	5.13.E-01	9.57.E-01
Autographiviridae	L7_Bifidobacterium_bifidum	-0.036	0.038	3.36.E-01	9.15.E-01
crAss_like_phage	L7_Bifidobacterium_bifidum	0.022	0.042	5.95.E-01	9.69.E-01
Herelleviridae	L7_Bifidobacterium_bifidum	0.045	0.039	2.48.E-01	8.94.E-01
Microviridae	L7_Bifidobacterium_bifidum	0.009	0.039	8.16.E-01	9.93.E-01
Myoviridae	L7_Bifidobacterium_bifidum	0.085	0.043	5.04.E-02	7.22.E-01
Phycodnaviridae	L7_Bifidobacterium_bifidum	-0.017	0.039	6.68.E-01	9.80.E-01
Podoviridae	L7_Bifidobacterium_bifidum	0.003	0.045	9.40.E-01	9.98.E-01
Siphoviridae	L7_Bifidobacterium_bifidum	0.062	0.040	1.20.E-01	7.91.E-01
Autographiviridae	L7_Prevotella_buccae	-0.021	0.041	6.15.E-01	9.75.E-01
crAss_like_phage	L7_Prevotella_buccae	0.018	0.045	6.85.E-01	9.80.E-01
Herelleviridae	L7_Prevotella_buccae	0.078	0.042	6.59.E-02	7.24.E-01
Microviridae	L7_Prevotella_buccae	0.066	0.042	1.22.E-01	7.93.E-01
Myoviridae	L7_Prevotella_buccae	0.095	0.047	4.60.E-02	7.22.E-01
Phycodnaviridae	L7_Prevotella_buccae	-0.014	0.042	7.35.E-01	9.88.E-01
Podoviridae	L7_Prevotella_buccae	-0.042	0.049	3.90.E-01	9.15.E-01
Siphoviridae	L7_Prevotella_buccae	-0.012	0.044	7.87.E-01	9.92.E-01
Autographiviridae	L7_Delftia_tsuruhatensis	0.007	0.016	6.80.E-01	9.80.E-01
crAss_like_phage	L7_Delftia_tsuruhatensis	-0.033	0.018	6.37.E-02	7.24.E-01
Herelleviridae	L7_Delftia_tsuruhatensis	-0.030	0.016	6.70.E-02	7.24.E-01
Microviridae	L7_Delftia_tsuruhatensis	-0.005	0.017	7.80.E-01	9.92.E-01
Myoviridae	L7_Delftia_tsuruhatensis	0.049	0.018	7.59.E-03	4.66.E-01
Phycodnaviridae	L7_Delftia_tsuruhatensis	0.015	0.016	3.48.E-01	9.15.E-01
Podoviridae	L7_Delftia_tsuruhatensis	-0.019	0.019	3.27.E-01	9.15.E-01
Siphoviridae	L7_Delftia_tsuruhatensis	-0.010	0.017	5.72.E-01	9.61.E-01
Autographiviridae	L7_Anaerofustis_stercorihominis	0.021	0.035	5.58.E-01	9.61.E-01
crAss_like_phage	L7_Anaerofustis_stercorihominis	0.013	0.039	7.37.E-01	9.88.E-01
Herelleviridae	L7_Anaerofustis_stercorihominis	0.035	0.037	3.39.E-01	9.15.E-01
Microviridae	L7_Anaerofustis_stercorihominis	-0.013	0.037	7.33.E-01	9.88.E-01
Myoviridae	L7_Anaerofustis_stercorihominis	-0.046	0.041	2.57.E-01	8.95.E-01
Phycodnaviridae	L7_Anaerofustis_stercorihominis	-0.052	0.036	1.55.E-01	8.26.E-01
Podoviridae	L7_Anaerofustis_stercorihominis	0.029	0.042	4.93.E-01	9.55.E-01
Siphoviridae	L7_Anaerofustis_stercorihominis	0.031	0.038	4.18.E-01	9.19.E-01
Autographiviridae	L7_Holdemania_filiformis	0.011	0.038	7.77.E-01	9.92.E-01
crAss_like_phage	L7_Holdemania_filiformis	-0.067	0.042	1.09.E-01	7.82.E-01
Herelleviridae	L7_Holdemania_filiformis	-0.022	0.039	5.70.E-01	9.61.E-01
Microviridae	L7_Holdemania_filiformis	-0.012	0.039	7.65.E-01	9.91.E-01
Myoviridae	L7_Holdemania_filiformis	-0.016	0.044	7.10.E-01	9.84.E-01
Phycodnaviridae	L7_Holdemania_filiformis	0.023	0.039	5.45.E-01	9.60.E-01

Podoviridae	L7_Holdemania_filiformis	0.085	0.045	5.98.E-02	7.24.E-01
Siphoviridae	L7_Holdemania_filiformis	0.020	0.040	6.19.E-01	9.75.E-01
Autographiviridae	L7_Eisenbergiella_sp.	-0.048	0.036	1.83.E-01	8.60.E-01
crAss_like_phage	L7_Eisenbergiella_sp.	-0.006	0.040	8.86.E-01	9.96.E-01
Herelleviridae	L7_Eisenbergiella_sp.	-0.001	0.037	9.88.E-01	9.99.E-01
Microviridae	L7_Eisenbergiella_sp.	0.079	0.037	3.51.E-02	6.90.E-01
Myoviridae	L7_Eisenbergiella_sp.	-0.082	0.041	4.94.E-02	7.22.E-01
Phycodnaviridae	L7_Eisenbergiella_sp.	-0.001	0.037	9.78.E-01	9.99.E-01
Podoviridae	L7_Eisenbergiella_sp.	0.016	0.043	7.13.E-01	9.86.E-01
Siphoviridae	L7_Eisenbergiella_sp.	0.029	0.038	4.43.E-01	9.32.E-01
Autographiviridae	L7_Prevotella_nanceiensis	0.026	0.041	5.35.E-01	9.59.E-01
crAss_like_phage	L7_Prevotella_nanceiensis	0.013	0.046	7.78.E-01	9.92.E-01
Herelleviridae	L7_Prevotella_nanceiensis	-0.022	0.043	6.12.E-01	9.75.E-01
Microviridae	L7_Prevotella_nanceiensis	0.014	0.043	7.38.E-01	9.88.E-01
Myoviridae	L7_Prevotella_nanceiensis	-0.020	0.048	6.74.E-01	9.80.E-01
Phycodnaviridae	L7_Prevotella_nanceiensis	0.017	0.043	6.83.E-01	9.80.E-01
Podoviridae	L7_Prevotella_nanceiensis	0.065	0.050	1.87.E-01	8.61.E-01
Siphoviridae	L7_Prevotella_nanceiensis	0.001	0.044	9.76.E-01	9.99.E-01
Autographiviridae	L7_Blautia_luti	0.040	0.035	2.53.E-01	8.95.E-01
crAss_like_phage	L7_Blautia_luti	0.002	0.038	9.66.E-01	9.98.E-01
Herelleviridae	L7_Blautia_luti	-0.002	0.036	9.55.E-01	9.98.E-01
Microviridae	L7_Blautia_luti	-0.009	0.036	8.05.E-01	9.93.E-01
Myoviridae	L7_Blautia_luti	-0.041	0.040	3.02.E-01	9.15.E-01
Phycodnaviridae	L7_Blautia_luti	0.000	0.036	9.97.E-01	9.99.E-01
Podoviridae	L7_Blautia_luti	-0.019	0.041	6.41.E-01	9.79.E-01
Siphoviridae	L7_Blautia_luti	-0.006	0.037	8.76.E-01	9.96.E-01
Autographiviridae	L7_Actinomyces_massiliensis	0.008	0.036	8.27.E-01	9.93.E-01
crAss_like_phage	L7_Actinomyces_massiliensis	-0.010	0.040	8.06.E-01	9.93.E-01
Herelleviridae	L7_Actinomyces_massiliensis	-0.081	0.037	2.98.E-02	6.66.E-01
Microviridae	L7_Actinomyces_massiliensis	-0.045	0.038	2.32.E-01	8.82.E-01
Myoviridae	L7_Actinomyces_massiliensis	-0.062	0.042	1.41.E-01	8.10.E-01
Phycodnaviridae	L7_Actinomyces_massiliensis	-0.020	0.037	5.98.E-01	9.69.E-01
Podoviridae	L7_Actinomyces_massiliensis	-0.027	0.043	5.33.E-01	9.58.E-01
Siphoviridae	L7_Actinomyces_massiliensis	-0.024	0.039	5.41.E-01	9.60.E-01
Autographiviridae	L7_Klebsiella_aerogenes	0.032	0.029	2.74.E-01	9.04.E-01
crAss_like_phage	L7_Klebsiella_aerogenes	0.015	0.032	6.47.E-01	9.79.E-01
Herelleviridae	L7_Klebsiella_aerogenes	-0.003	0.030	9.23.E-01	9.98.E-01
Microviridae	L7_Klebsiella_aerogenes	-0.026	0.030	3.92.E-01	9.15.E-01
Myoviridae	L7_Klebsiella_aerogenes	0.029	0.034	3.98.E-01	9.15.E-01
Phycodnaviridae	L7_Klebsiella_aerogenes	-0.003	0.030	9.32.E-01	9.98.E-01
Podoviridae	L7_Klebsiella_aerogenes	-0.038	0.035	2.72.E-01	9.02.E-01
Siphoviridae	L7_Klebsiella_aerogenes	0.023	0.031	4.52.E-01	9.38.E-01
Autographiviridae	L7_Bifidobacterium_scardovii	-0.082	0.039	3.51.E-02	6.90.E-01
crAss_like_phage	L7_Bifidobacterium_scardovii	0.009	0.043	8.43.E-01	9.95.E-01
Herelleviridae	L7_Bifidobacterium_scardovii	0.048	0.040	2.32.E-01	8.82.E-01
Microviridae	L7_Bifidobacterium_scardovii	0.005	0.041	9.04.E-01	9.97.E-01
Myoviridae	L7_Bifidobacterium_scardovii	-0.022	0.045	6.32.E-01	9.76.E-01
Phycodnaviridae	L7_Bifidobacterium_scardovii	0.014	0.040	7.34.E-01	9.88.E-01
Podoviridae	L7_Bifidobacterium_scardovii	0.015	0.047	7.46.E-01	9.89.E-01
Siphoviridae	L7_Bifidobacterium_scardovii	0.038	0.042	3.65.E-01	9.15.E-01
Autographiviridae	L7_Streptococcus_infantis	0.013	0.024	5.95.E-01	9.69.E-01
crAss_like_phage	L7_Streptococcus_infantis	0.003	0.027	9.11.E-01	9.98.E-01
Herelleviridae	L7_Streptococcus_infantis	-0.014	0.025	5.68.E-01	9.61.E-01
Microviridae	L7_Streptococcus_infantis	-0.017	0.025	5.08.E-01	9.57.E-01
Myoviridae	L7_Streptococcus_infantis	0.006	0.028	8.35.E-01	9.94.E-01
Phycodnaviridae	L7_Streptococcus_infantis	0.040	0.025	1.08.E-01	7.82.E-01
Podoviridae	L7_Streptococcus_infantis	0.029	0.029	3.20.E-01	9.15.E-01
Siphoviridae	L7_Streptococcus_infantis	0.058	0.026	2.29.E-02	6.19.E-01
Autographiviridae	L7_Asaccharobacter_celatus	0.006	0.035	8.60.E-01	9.96.E-01
crAss_like_phage	L7_Asaccharobacter_celatus	0.010	0.038	7.98.E-01	9.92.E-01
Herelleviridae	L7_Asaccharobacter_celatus	0.026	0.036	4.74.E-01	9.52.E-01
Microviridae	L7_Asaccharobacter_celatus	-0.029	0.036	4.25.E-01	9.23.E-01
Myoviridae	L7_Asaccharobacter_celatus	-0.005	0.040	9.05.E-01	9.97.E-01
Phycodnaviridae	L7_Asaccharobacter_celatus	0.023	0.036	5.12.E-01	9.57.E-01
Podoviridae	L7_Asaccharobacter_celatus	-0.021	0.042	6.20.E-01	9.75.E-01

Siphoviridae	L7_Asaccharobacter_celatus	0.078	0.037	3.48.E-02	6.89.E-01
Autographiviridae	L7_Acidaminococcus_sp.	0.015	0.044	7.37.E-01	9.88.E-01
crAss_like_phage	L7_Acidaminococcus_sp.	-0.093	0.049	5.70.E-02	7.24.E-01
Herelleviridae	L7_Acidaminococcus_sp.	0.006	0.046	8.95.E-01	9.97.E-01
Microviridae	L7_Acidaminococcus_sp.	-0.020	0.046	6.72.E-01	9.80.E-01
Myoviridae	L7_Acidaminococcus_sp.	-0.004	0.051	9.34.E-01	9.98.E-01
Phycodnaviridae	L7_Acidaminococcus_sp.	0.028	0.045	5.43.E-01	9.60.E-01
Podoviridae	L7_Acidaminococcus_sp.	-0.009	0.053	8.67.E-01	9.96.E-01
Siphoviridae	L7_Acidaminococcus_sp.	0.011	0.047	8.23.E-01	9.93.E-01
Autographiviridae	L7_Alloscardovia_omnicolens	-0.040	0.040	3.21.E-01	9.15.E-01
crAss_like_phage	L7_Alloscardovia_omnicolens	-0.037	0.044	3.97.E-01	9.15.E-01
Herelleviridae	L7_Alloscardovia_omnicolens	0.042	0.041	3.09.E-01	9.15.E-01
Microviridae	L7_Alloscardovia_omnicolens	0.024	0.041	5.71.E-01	9.61.E-01
Myoviridae	L7_Alloscardovia_omnicolens	-0.053	0.046	2.52.E-01	8.95.E-01
Phycodnaviridae	L7_Alloscardovia_omnicolens	0.022	0.041	5.97.E-01	9.69.E-01
Podoviridae	L7_Alloscardovia_omnicolens	-0.026	0.048	5.82.E-01	9.64.E-01
Siphoviridae	L7_Alloscardovia_omnicolens	-0.024	0.043	5.80.E-01	9.64.E-01
Autographiviridae	L7_Enterococcus_gallinarum	0.044	0.039	2.67.E-01	9.01.E-01
crAss_like_phage	L7_Enterococcus_gallinarum	-0.044	0.043	3.12.E-01	9.15.E-01
Herelleviridae	L7_Enterococcus_gallinarum	0.015	0.041	7.15.E-01	9.86.E-01
Microviridae	L7_Enterococcus_gallinarum	-0.018	0.041	6.64.E-01	9.79.E-01
Myoviridae	L7_Enterococcus_gallinarum	-0.038	0.046	4.00.E-01	9.15.E-01
Phycodnaviridae	L7_Enterococcus_gallinarum	0.015	0.040	7.18.E-01	9.86.E-01
Podoviridae	L7_Enterococcus_gallinarum	0.043	0.047	3.60.E-01	9.15.E-01
Siphoviridae	L7_Enterococcus_gallinarum	-0.040	0.042	3.43.E-01	9.15.E-01
Autographiviridae	L7_Lactobacillus_delbrueckii	0.008	0.043	8.45.E-01	9.95.E-01
crAss_like_phage	L7_Lactobacillus_delbrueckii	0.039	0.048	4.11.E-01	9.17.E-01
Herelleviridae	L7_Lactobacillus_delbrueckii	0.015	0.045	7.44.E-01	9.89.E-01
Microviridae	L7_Lactobacillus_delbrueckii	0.010	0.045	8.19.E-01	9.93.E-01
Myoviridae	L7_Lactobacillus_delbrueckii	0.021	0.050	6.70.E-01	9.80.E-01
Phycodnaviridae	L7_Lactobacillus_delbrueckii	-0.025	0.044	5.68.E-01	9.61.E-01
Podoviridae	L7_Lactobacillus_delbrueckii	0.060	0.052	2.46.E-01	8.92.E-01
Siphoviridae	L7_Lactobacillus_delbrueckii	0.058	0.046	2.08.E-01	8.76.E-01
Autographiviridae	L7_Parabacteroides_sp.	0.017	0.028	5.55.E-01	9.61.E-01
crAss_like_phage	L7_Parabacteroides_sp.	0.014	0.031	6.51.E-01	9.79.E-01
Herelleviridae	L7_Parabacteroides_sp.	0.025	0.029	3.99.E-01	9.15.E-01
Microviridae	L7_Parabacteroides_sp.	-0.060	0.029	4.00.E-02	7.03.E-01
Myoviridae	L7_Parabacteroides_sp.	-0.067	0.033	4.01.E-02	7.03.E-01
Phycodnaviridae	L7_Parabacteroides_sp.	-0.028	0.029	3.33.E-01	9.15.E-01
Podoviridae	L7_Parabacteroides_sp.	0.030	0.034	3.82.E-01	9.15.E-01
Siphoviridae	L7_Parabacteroides_sp.	-0.026	0.030	3.83.E-01	9.15.E-01
Autographiviridae	L7_Haemophilus_haemolyticus	-0.001	0.034	9.66.E-01	9.98.E-01
crAss_like_phage	L7_Haemophilus_haemolyticus	-0.008	0.038	8.27.E-01	9.93.E-01
Herelleviridae	L7_Haemophilus_haemolyticus	0.088	0.036	1.37.E-02	5.51.E-01
Microviridae	L7_Haemophilus_haemolyticus	0.016	0.036	6.46.E-01	9.79.E-01
Myoviridae	L7_Haemophilus_haemolyticus	0.114	0.040	4.25.E-03	4.23.E-01
Phycodnaviridae	L7_Haemophilus_haemolyticus	0.037	0.035	2.95.E-01	9.15.E-01
Podoviridae	L7_Haemophilus_haemolyticus	0.028	0.041	5.01.E-01	9.55.E-01
Siphoviridae	L7_Haemophilus_haemolyticus	0.000	0.037	9.96.E-01	9.99.E-01
Autographiviridae	L7_Actinomyces_dentalis	-0.043	0.040	2.82.E-01	9.13.E-01
crAss_like_phage	L7_Actinomyces_dentalis	-0.037	0.044	4.03.E-01	9.16.E-01
Herelleviridae	L7_Actinomyces_dentalis	-0.024	0.041	5.70.E-01	9.61.E-01
Microviridae	L7_Actinomyces_dentalis	-0.021	0.042	6.16.E-01	9.75.E-01
Myoviridae	L7_Actinomyces_dentalis	-0.027	0.046	5.60.E-01	9.61.E-01
Phycodnaviridae	L7_Actinomyces_dentalis	-0.010	0.041	8.17.E-01	9.93.E-01
Podoviridae	L7_Actinomyces_dentalis	-0.077	0.048	1.07.E-01	7.82.E-01
Siphoviridae	L7_Actinomyces_dentalis	-0.030	0.043	4.82.E-01	9.53.E-01
Autographiviridae	L7_Fusobacterium_ulcerans	-0.064	0.038	9.29.E-02	7.60.E-01
crAss_like_phage	L7_Fusobacterium_ulcerans	-0.009	0.042	8.32.E-01	9.94.E-01
Herelleviridae	L7_Fusobacterium_ulcerans	0.008	0.040	8.42.E-01	9.95.E-01
Microviridae	L7_Fusobacterium_ulcerans	-0.132	0.039	8.59.E-04	2.79.E-01
Myoviridae	L7_Fusobacterium_ulcerans	-0.029	0.045	5.20.E-01	9.57.E-01
Phycodnaviridae	L7_Fusobacterium_ulcerans	-0.001	0.039	9.79.E-01	9.99.E-01
Podoviridae	L7_Fusobacterium_ulcerans	0.081	0.046	7.80.E-02	7.42.E-01
Siphoviridae	L7_Fusobacterium_ulcerans	-0.022	0.041	5.91.E-01	9.68.E-01

Autographiviridae	L7_Blautia_hansenii	-0.039	0.038	3.03.E-01	9.15.E-01
crAss_like_phage	L7_Blautia_hansenii	-0.050	0.042	2.30.E-01	8.82.E-01
Herelleviridae	L7_Blautia_hansenii	0.005	0.039	8.93.E-01	9.97.E-01
Microviridae	L7_Blautia_hansenii	0.028	0.039	4.76.E-01	9.52.E-01
Myoviridae	L7_Blautia_hansenii	0.006	0.044	8.98.E-01	9.97.E-01
Phycodnaviridae	L7_Blautia_hansenii	-0.072	0.039	6.17.E-02	7.24.E-01
Podoviridae	L7_Blautia_hansenii	-0.062	0.045	1.71.E-01	8.50.E-01
Siphoviridae	L7_Blautia_hansenii	-0.007	0.040	8.70.E-01	9.96.E-01
Autographiviridae	L7_Lachnospiraceae_bacterium	-0.001	0.035	9.68.E-01	9.98.E-01
crAss_like_phage	L7_Lachnospiraceae_bacterium	-0.038	0.038	3.21.E-01	9.15.E-01
Herelleviridae	L7_Lachnospiraceae_bacterium	0.026	0.036	4.65.E-01	9.45.E-01
Microviridae	L7_Lachnospiraceae_bacterium	-0.005	0.036	8.85.E-01	9.96.E-01
Myoviridae	L7_Lachnospiraceae_bacterium	-0.032	0.040	4.24.E-01	9.23.E-01
Phycodnaviridae	L7_Lachnospiraceae_bacterium	0.069	0.035	5.30.E-02	7.22.E-01
Podoviridae	L7_Lachnospiraceae_bacterium	0.002	0.041	9.53.E-01	9.98.E-01
Siphoviridae	L7_Lachnospiraceae_bacterium	0.034	0.037	3.52.E-01	9.15.E-01
Autographiviridae	L7_Collinsella_stercoris	-0.028	0.037	4.49.E-01	9.36.E-01
crAss_like_phage	L7_Collinsella_stercoris	-0.028	0.041	4.95.E-01	9.55.E-01
Herelleviridae	L7_Collinsella_stercoris	0.004	0.039	9.22.E-01	9.98.E-01
Microviridae	L7_Collinsella_stercoris	-0.020	0.039	6.09.E-01	9.75.E-01
Myoviridae	L7_Collinsella_stercoris	-0.046	0.043	2.95.E-01	9.15.E-01
Phycodnaviridae	L7_Collinsella_stercoris	0.007	0.038	8.52.E-01	9.95.E-01
Podoviridae	L7_Collinsella_stercoris	-0.083	0.045	6.57.E-02	7.24.E-01
Siphoviridae	L7_Collinsella_stercoris	-0.017	0.040	6.75.E-01	9.80.E-01
Autographiviridae	L7_Bacteroides_sartorii	0.003	0.026	8.98.E-01	9.97.E-01
crAss_like_phage	L7_Bacteroides_sartorii	-0.024	0.029	3.99.E-01	9.15.E-01
Herelleviridae	L7_Bacteroides_sartorii	-0.030	0.027	2.57.E-01	8.95.E-01
Microviridae	L7_Bacteroides_sartorii	-0.018	0.027	4.93.E-01	9.55.E-01
Myoviridae	L7_Bacteroides_sartorii	-0.030	0.030	3.20.E-01	9.15.E-01
Phycodnaviridae	L7_Bacteroides_sartorii	-0.062	0.026	2.01.E-02	5.97.E-01
Podoviridae	L7_Bacteroides_sartorii	-0.062	0.031	4.54.E-02	7.22.E-01
Siphoviridae	L7_Bacteroides_sartorii	-0.006	0.028	8.28.E-01	9.94.E-01
Autographiviridae	L7_Clostridium_celatum	-0.033	0.036	3.49.E-01	9.15.E-01
crAss_like_phage	L7_Clostridium_celatum	0.014	0.039	7.22.E-01	9.86.E-01
Herelleviridae	L7_Clostridium_celatum	-0.006	0.037	8.65.E-01	9.96.E-01
Microviridae	L7_Clostridium_celatum	-0.054	0.037	1.46.E-01	8.15.E-01
Myoviridae	L7_Clostridium_celatum	-0.009	0.041	8.19.E-01	9.93.E-01
Phycodnaviridae	L7_Clostridium_celatum	0.036	0.037	3.20.E-01	9.15.E-01
Podoviridae	L7_Clostridium_celatum	0.014	0.043	7.48.E-01	9.89.E-01
Siphoviridae	L7_Clostridium_celatum	-0.045	0.038	2.33.E-01	8.84.E-01
Autographiviridae	L7_Bifidobacterium_angulatum	0.025	0.027	3.68.E-01	9.15.E-01
crAss_like_phage	L7_Bifidobacterium_angulatum	-0.035	0.030	2.49.E-01	8.94.E-01
Herelleviridae	L7_Bifidobacterium_angulatum	-0.003	0.028	9.25.E-01	9.98.E-01
Microviridae	L7_Bifidobacterium_angulatum	0.001	0.028	9.79.E-01	9.99.E-01
Myoviridae	L7_Bifidobacterium_angulatum	0.015	0.032	6.42.E-01	9.79.E-01
Phycodnaviridae	L7_Bifidobacterium_angulatum	0.011	0.028	6.89.E-01	9.80.E-01
Podoviridae	L7_Bifidobacterium_angulatum	0.060	0.033	6.50.E-02	7.24.E-01
Siphoviridae	L7_Bifidobacterium_angulatum	0.030	0.029	3.11.E-01	9.15.E-01
